# Specific impacts of beech and Norway spruce on the structure and diversity of the rhizosphere and soil microbial communities

**DOI:** 10.1038/srep27756

**Published:** 2016-06-15

**Authors:** S. Uroz, P. Oger, E. Tisserand, A. Cébron, M.-P. Turpault, M. Buée, W. De Boer, J. H. J. Leveau, P. Frey-Klett

**Affiliations:** 1 INRA-Université de Lorraine , UMR1136 « Interactions Arbres-Microorganismes », F-54280 Champenoux, France; 2 INRA UR 1138 “Biogéochimie des Ecosystèmes Forestiers”, Centre INRA de Nancy, Champenoux, France; 3 UMR5276 Laboratoire de Géologie de Lyon, Ecole Normale de Lyon, 46 allée d’Italie, 69364 Lyon Cedex 07, France; 4 CNRS, LIEC UMR7360 Faculté des Sciences et Technologies, 54506 Vandoeuvre-les-Nancy, France; 5 Université de Lorraine, LIEC UMR7360 Faculté des Sciences et Technologies, 54506 Vandoeuvre-les-Nancy, France; 6 Netherlands Institute of Ecology (NIOO-KNAW), Department of Microbial Ecology, Wageningen, The Netherlands; 7 Department of Plant Pathology, University of California, Davis CA 95616, USA

## Abstract

The impacts of plant species on the microbial communities and physico-chemical characteristics of soil are well documented for many herbs, grasses and legumes but much less so for tree species. Here, we investigate by rRNA and ITS amplicon sequencing the diversity of microorganisms from the three domains of life (Archaea, Bacteria and Eukaryota:Fungi) in soil samples taken from the forest experimental site of Breuil-Chenue (France). We discovered significant differences in the abundance, composition and structure of the microbial communities associated with two phylogenetically distant tree species of the same age, deciduous European beech (*Fagus sylvatica*) and coniferous Norway spruce (*Picea abies* Karst), planted in the same soil. Our results suggest a significant effect of tree species on soil microbiota though in different ways for each of the three microbial groups. Fungal and archaeal community structures and compositions are mainly determined according to tree species, whereas bacterial communities differ to a great degree between rhizosphere and bulk soils, regardless of the tree species. These results were confirmed by quantitative PCR, which revealed significant enrichment of specific bacterial genera, such as *Burkholderia* and *Collimonas*, known for their ability to weather minerals within the tree root vicinity.

Several studies have investigated the main biotic and abiotic factors determining the structure and functioning of soil microbial communities. In addition to impacts of soil type, soil age, soil mineralogy and soil pH, several studies have highlighted the strong link between plants and soil microorganisms^1^,^2^,^3^,^4^,^5^,^6^,^7^. Indeed, through litter degradation and the production of nutrients and protons in their rhizodeposits, plants modify the physical, chemical and biological properties of their soil environment^8^,^9^,^10^,^11^,^12^. For example, evergreen tree species such as Norway spruce (*Picea abies)* and Scots pine *(Pinus sylvestris*) are known for acidifying soils. Furthermore, nutrients and signaling molecules present in root exudates promote the selection of particular taxa and functions within the vicinity of the root system^4^,^13^. One hypothesis to explain this selection, known as the rhizosphere effect^14^, is that plants recruit indigenous microbial communities from the soil that are beneficial for improving plant health and nutrition and for preventing the establishment of pathogens.

Such a selective effect in the rhizosphere environment has been reported for several cultivated, desert and perennial plants^15^,^16^,^17^,^18^,^19^. Over the last decade, the development of high-density 16S rRNA microarray as well as monogenic and shotgun metagenomics has resulted in great advancements in our understanding of the structure and diversity of soil microbiomes^19^,^20^,^21^. Conversely, our knowledge of the functional capacities of rhizosphere microbial communities remains largely based on cultivation-dependent studies^15^,^22^. Nonetheless, functional screenings of culturable bacterial and fungal communities have revealed rhizosphere enrichment of microorganisms capable of improving plant nutrition by providing nitrogen and nutritive cations^15^. Cultivation-independent studies have also shown an increase in the relative abundance of specific functional genes related to nitrogen cycling, carbon fixation, phosphorus utilization, metal homeostasis and resistance in the rhizosphere^21^,^23^. Altogether, these results suggest that plants enrich nutritional helper microbial communities within the vicinity of their roots. Similar conclusions have been drawn about animal and human microbiomes, which strongly participate in key functions related to host defense, metabolism, and health^24^. Such functional complementation by the rhizosphere microbiome is particularly important for plants growing in nutrient-poor soils in which nutrients are scarce and not well accessible to roots, as is typically the case of forests developed on acidic and nutrient-poor soils. Under such conditions, trees have been shown to select symbiotic fungi and bacteria that are capable of mobilizing nutrients from minerals and organic matter, thereby supplying the trees with organic and inorganic nutrients^15^,^25^. However, this selective effect appears to be dependent on the host^15^ and remains poorly documented, particularly with regard to archaeal and bacterial communities.

The impact of plant host on the soil microbial communities has been widely investigated, with results showing that different plant species and genotypes (including genetically modified plants) can select specific microbial communities from the soil reservoir within their root vicinity^13^,^26^,^27^,^28^,^29^,^30^. Notably, this selection has been shown to change during plant development and root growth, indicating that such selection is altered according to the nutritional requirements or physiology of the plant^20^,^31^. This selective effect also appears to be dependent on the co-evolution history shared by plants and soil microbial communities^32^,^33^. However, most of the studies to date on the forest soil microbiome are organism specific, whereas few have simultaneously analyzed archaeal, bacterial and fungal communities^34^,^35^,^36^,^37^. As an example, Fierer *et al*.^34^ were among the first to identify archaeal, bacterial, fungal and even viral communities by applying cloning-sequencing, but their analysis only considered bulk soil samples. In addition, by analyzing archaea and bacteria, but not fungi, in soil from a temperate beech forest, Rasche *et al*.^35^ showed that both communities were impacted by seasonality and variations in nutrient availability. Finally, Urbanova *et al*.^36^ analyzed the soil stratification of both fungal and bacterial communities using high-throughput sequencing technology, determining how these two communities (archaeal communities were not considered) were distributed and whether this distribution varied for different tree species. However, none of these studies assessed differences in rhizosphere and bulk soil microbial communities in terms of structure, diversity and composition. Within this context, the aim of this study was to determine the structure, richness and abundance of archaeal, bacterial, and fungal communities in an acidic forest plantation and to assess how different tree species of the same age, growing in the same soil, impact these microbial communities. We hypothesized that tree species have specific effects on the composition and structure of soil microbial communities. An ideal site to test this hypothesis is the long-term observatory (LTO) forest site of Breuil-Chenue (Morvan, France), which is characterized by acidic and nutrient-poor soil conditions. The native forest was partially clear-felled in 1976 and replaced by mono-specific plantations. In this study, spatially independent samples were collected from soil in which two phylogenetically distant tree species, beech (B; *Fagus sylvatica* L.) and Norway spruce (NW; *Picea abies* Karst), were growing. To assess community structure, we performed archaeal and bacterial 16S rRNA and fungal 18S rRNA and ribosomal internal transcribed spacer (ITS) amplicon pyrosequencing using DNA isolated from the rhizosphere and bulk soil compartments to examine relationships among microbial diversity, tree species and soil characteristics. The same soil samples were also used for quantitative PCR to quantify the archaeal, bacterial and fungal community abundance as well as that of specific bacterial genera such as *Burkholderia* and *Collimonas* known for their efficacy in weathering minerals at this experimental site^15^,^18^,^38^.

## Results

### Chemical and microbial characterization of rhizosphere and bulk soils below beech and Norway spruce

The chemical analyses of bulk soil (BS) and rhizosphere soil (RS) samples are summarized in Table 1. A significantly lower pH was measured in the Norway spruce rhizosphere (pH = 3.6) compared to the bulk soil samples (pH 4.0; p = 0.01), though no difference in pH was observed for the beech bulk and rhizosphere soils (pH = 3.9 for both R and BS). Significantly more exchangeable potassium was measured in the Norway spruce (p = 0.007) and beech (p = 0.02) rhizospheres than in their corresponding bulk soil samples, and significantly more Fe^3+^ was observed in the Norway spruce rhizosphere compared to the bulk soil samples (p = 0.02). All the other soil parameters measured did not vary significantly between the two soil compartments (R vs BS) or between the two tree stands (beech vs Norway spruce).

**Figure 1:**
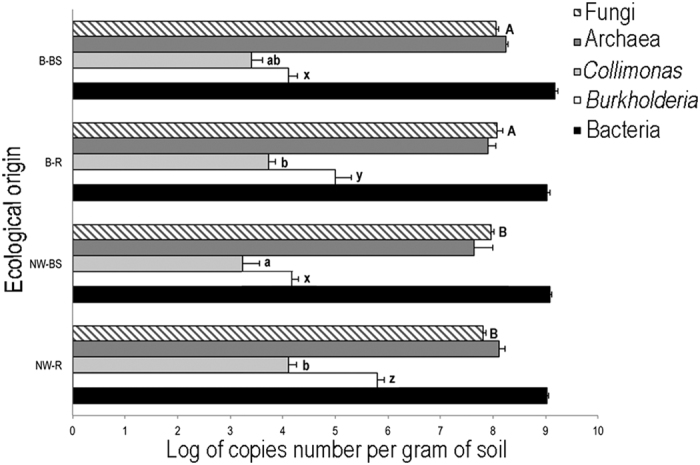
Quantification of specific taxa by quantitative PCR. Conditions presenting different letters are significantly different according to a one-factor ANOVA and Tukey post-hoc test (P < 0.05). Treatments are presented as follows: B-BS (beech bulk soil), B-R (beech rhizosphere), NW-BS (Norway spruce bulk soil), NW-R (Norway spruce rhizosphere).

At an average total bacterial population size of 9.05 ± 0.05 (expressed as log[16S rRNA gene copies per gram of soil]) did not differ significantly between the different tree stands or their soil compartments (Fig. 1). Archaea accounted for 1–23% of the total prokaryotes in the Norway spruce samples and 5.5 to 14% in the beech samples, though there was no significant difference in archaeal density according to tree species (average log value of 7.98 ± 0.11). Fungi were significantly more abundant in the beech stand (8.08 ± 0.09; p = 0.04) than in the Norway spruce stand (7.90 ± 0.07), but no rhizosphere effect was observed. Quantification of *Burkholderia* revealed a significant enrichment of this bacterial genus in the beech (B-R = 5 ± 0.30 vs B-BS = 4.10 ± 0.19; p = 0.029) and Norway spruce (NW-R = 5.80 ± 0.13 vs NW-BS = 4.24 ± 0.12; p = 0.0001) rhizospheres compared to the bulk soil samples, with significantly more *Burkholderia* detected in the Norway spruce rhizosphere than in the beech rhizosphere (p < 0.05). Quantification of *Collimonas* revealed an enrichment of this bacterial genus in the Norway spruce rhizosphere compartment (NW-R = 4.10 ± 0.16 vs NW-BS = 3.23 ± 0.33; p = 0.04), and analysis of the ratio (*Collimonas*/total bacterial community) revealed that these values were significantly higher in both the beech and Norway spruce rhizospheres than in the surrounding bulk soil.

### Pyrosequencing data analyses

We obtained a total of 440,000 reads for the beech and Norway spruce rhizosphere and bulk soil samples collected. After quality treatment, we obtained 26,386 reads for archaea, 203,476 reads for bacteria, and 24,540 and 14,607 reads for fungal ITS and 18S rRNA, respectively (Table S1). For each tree stand considered, the soil cores were not sampled to saturation because rarefaction analysis showed no significant differences between the rhizosphere and BS samples (Figure S1). For the same number of sequences, a higher number of archaeal and bacterial OTUs were observed in the beech BS samples. Conversely, a higher number of fungal OTUs were observed in the Norway spruce samples using both 18S rRNA and ITS meta-barcoding approaches. Notably, for all microbial communities considered, the Norway spruce BS and R samples harbored a similar number of OTUs. Non-parametric analyses also confirmed this trend. Although diversity (Shannon) and richness (Chao1) estimators did not permit differentiation among most of the microbial communities, a significantly higher Shannon value (H’) was observed for fungal ITS in the beech BS samples (H’ = 3.9) compared to the R samples (H’ = 3.1; p = 0.007) (Table S2).

### Taxonomic distribution and relationship with soil parameters

**Figure 2:**
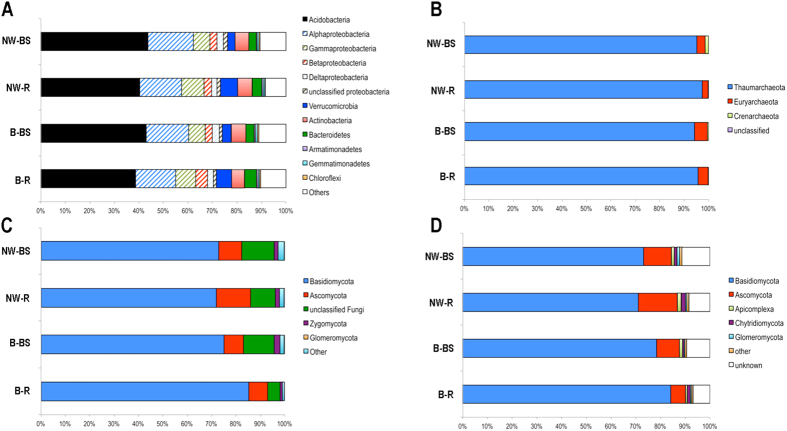
Relative distribution (%) of the main taxa detected among the archaeal, bacterial and fungal communities in the beech (B-BS, bulk soil; B-R, rhizosphere) and Norway spruce (NW-BS, bulk soil; NW-R, rhizosphere). (**A**) Bacteria, (**B**) Archaea, (**C**) Fungal communities based on ITS marker and (**D**) Fungal communities based on 18S rRNA gene marker. For the 18S rRNA results, the non fungal and apicomplexa sequences detected have been grouped in a category named other. Relative distribution was determined using the mean of 3 biological replicates for each tree species and soil compartment considered.

A total of 19 bacterial phyla were detected in the soil samples collected in the beech and Norway spruce stands, though only five presented an average abundance greater than 1%. Both tree stands appeared to be dominated by Acidobacteria (40.8% for beech and 42% for Norway spruce), Proteobacteria (32% for each), Actinobacteria (5.6% for beech and 5.8% for Norway spruce), Verrucomicrobia (5% for each) and Bacteroidetes (4.2% for beech and 3.5% for Norway spruce) (Fig. 2). Approximately 8-9% of the sequences from both stands remained unclassified.

Almost all of the archaeal sequences detected in the soil or rhizosphere samples belong to phylum Thaumarchaeota (96.5%). However, as Thaumarchaeota is a poorly characterized Archaeal phylum, the taxonomy of approx. 90% of the Thaumarchaeota sequences could not be determined (Fig. 2). The remaining 10% formed a cluster closely related to order Nitrososphaerales. The large majority of non-thaumarchaeal sequences are affiliated with Euryarchaeota (1.95% for Norway spruce and 3.92% for beech), particularly with the *Thermogymnomonas* genus of Thermoplasmatales. The remaining sequences (0.5%) are related to the Desulfurococcales family of Crenarchaeota. The diversity of Archaea appeared very limited, with the two largest OTUs regrouping 45% and 25.25% of all the archaeal sequences. Notably, the major 17 OTUs (represented by more than 10 sequences) accounted for approx. 99% of the archaeal sequences found.

Regarding fungal communities, dominant taxa were detected within the same range of abundance for both the ITS and 18rRNA approaches (Fig. 2). This analysis showed a dominance of Basidiomycota (72% for Norway spruce and 81% for beech) and Ascomycota (12% for spruce and 8.1% for beech). Notably, Zygomycota (1.6% for each stand) were detected only by the ITS approach, whereas phyla such as Chytridiomycota (1.46% for spruce and 0.9% for beech), Blastocladiomycota (0.15% for Norway spruce and 0.04% for beech) and Cryptomycota (0% for spruce and 0.2% for beech) were only detected using the 18S rRNA approach.

According to global Mantel tests, the relationships between soil parameters and the relative distribution of sequences among the archaeal, bacterial and fungal communities revealed no significant correlations when each tree stand was considered independently or when a combined analysis of the two tree stands was performed. However, a detailed analysis based Pearson correlations revealed significant correlations between the distributions of specific taxa among the archaeal, bacterial and fungal communities and certain soil parameters (Table 2).

### Tree species affects the structure and composition of soil microbial communities

According to ANOVA tests, no statistically significant differences were observed for bacteria at the phylum and class levels. This absence of tree species effect was confirmed by multivariate analysis, revealing that the rhizosphere and bulk soil samples grouped separately regardless of tree species (Figure S2). However, the bacterial communities did appear to be significantly impacted by tree species at lower phylogenetic levels. Indeed, at the order level, Sphingobacteriales and Enterobacteriales were significantly enriched in the beech soil samples (Sphingomonadales: 3.97% ± 0.35 vs 3.29% ± 0.41, p = 0.04; Enterobacteriales: 0.07% ± 0.009 vs 0.03% ± 0.01, p = 0,016), as observed at the family level for Enterobacteriaceae (B > NW; 0.07% ± 0.01 vs 0.03 ± 0.01, p = 0.016), Sphingomonadaceae (B > NW, 0.99% ± 0.14 vs 0.71% ± 0.11 p = 0.049), Xanthomonadaceae (B > NW, 0.66% ± 0.07 vs 0.20% ± 0.06; p = 0.001), and Sinobacteraceae (NW > B; 4.49% ± 0.73 vs 2.48% ± 0.29, p = 0.028). We further analyzed the bacterial communities at the genus level and observed that *Rhodanobacter, Steroidobacter* and *Flavitalea* varied in abundance depending on the tree species. *Rhodanobacter* was significantly enriched below beech (B = 0.21% ± 0.06 vs NW = 0.03% ± 0.01, p = 0.02) and *Steroidobacter* and *Flavitalea* below Norway spruce (*Steroidobacter*: NW = 4.24% ± 0.75 vs B = 2.31% ± 0.27, p = 0.035; *Flavitalea*: NW = 0.15% ± 0.03 vs B = 0.06% ± 0.01; p = 0.018). Notably, *Collimonas* was found to be a rare genus and was mainly detected in the beech soil pyrosequencing data. A total of 9,266 distinct OTUs (including singletons) were generated using a threshold of 97%; among them, 19 OTUs increased significantly in the Norway spruce as well as beech soil samples. This effect of tree species was also confirmed by an analysis of similarities (ANOSIM; R = 0.30 and p = 0.023) performed at the OTU level.

For archaea, no statistically significant differences were observed between the beech and Norway spruce samples at any taxonomic level (from phylum to OTU) based on ANOVA analyses. However, ANOSIM analysis revealed significant differences in term of structure between the two tree stands (R = 0.25 and p = 0.021). This tree species effect is presented in Figure S3. Notably, members of Thermoplasmatales were systematically enriched in the beech samples (3.58% and 4.51% of sequences for the rhizosphere and bulk soil, respectively) in comparison to the Norway spruce samples (2.31% and 1.72% of sequences for the rhizosphere and bulk soil, respectively).

For fungi, the 18S rRNA and ITS pyrosequencing approaches gave similar trends, even though specific taxa were only detected using only one of the two marker genes. The tree species effect was confirmed by multivariate analysis (Figures S4 and S5). At the phylum level, only the 18S rRNA approach revealed a significant enrichment of Basidiomycota below beech compared to Norway spruce (p < 0.01). At the class level, both approaches revealed a significant enrichment of Agaromycetes under beech, and more members of Tremellomycetes were found to be associated with Norway spruce. The 18S rRNA approach also revealed enrichment of Leotiomycetes and Saccharomycetes under Norway spruce. Significant differences were also observed at the order, family and genus levels, with several fungal genera appearing to be enriched below one tree species. For example, the beech stand showed enrichment in *Scleroderma, Russula, Dendrocollybia, Laccaria, Gyromitra,* and *Pleurocybella* sequences, whereas *Boletus, Cryptococcus, Asterotremella, Jaapia, Thelephora, Candida* and *Archaeorhizomyces* were enriched in the Norway spruce stand. Analyses performed at the OTU level revealed significant enrichment of sequences related to *Scleroderma citrinum*, *Russula spp.* and *Amanita rubescens* in the beech stand and of sequences related to *Atheliales* sp. and *Amanita* sp. in the Norway spruce stand. Notably, the 18S rRNA approach revealed enrichment of species not detected by the ITS approach or with no significant differences between the two tree species, such as *Xerocomellus sp., Russula exalbicans, Pyxidiophora arvernensis* and *Inocybe sp.* This host preference, or selectivity, was also confirmed by an analysis of similarities (ANOSIM) performed at the OTU level on 18S rRNA (R = 0.73 and p < 0.001) and ITS (R = 0.98 and p < 0.001) sequences, confirming the ANOVA results obtained at the different phylogenetic levels presented above.

### Structure and composition of rhizosphere and bulk soil microbial communities

To determine the selective effect of the rhizosphere habitat on bacterial, archaeal and fungal communities, the sequences generated from the Norway spruce and the beech soil samples were analyzed independently from the phylum to OTU levels. For each tree species, no significant difference between beech and Norway spruce bulk soil and rhizosphere samples was observed for bacteria at the phylum level (ANOVA and ANOSIM, p > 0.05); however, multivariate analysis separated the rhizosphere and bulk soil samples in each tree stand (Figure S2). At the class level, the beech rhizosphere samples were significantly enriched in Sphingobacteria (p = 0.037; B-R = 4.83% ± 0.06 vs B-BS = 3.22% ± 0.55) and Betaproteobacteria (p = 0.011; B-R = 4.87% ± 0.043 vs B-BS = 2.90% ± 0.10); in the Norway spruce soil samples, the rhizosphere was significantly enriched in Armatimonadia (NW-R = 0.24% ± 0.06 vs NW-BS = 0.058% ± 0.011, p = 0.039) and in Bacteroidetes in bulk soil (NW-BS = 0.058% ± 0.011 vs NW-R = 0.012% ± 0.011, p = 0.047). At the order level, the beech rhizosphere was significantly enriched in representatives of Burkholderiales (p = 0.015) and Sphingobacteriales (p = 0.03). Rhizobiales was significantly enriched in the bulk soil samples for both tree species (p = 0.021). At the family level, Xanthobacteraceae was significantly enriched in the Norway spruce soil (p = 0.03) and Armatimonadaceae in the rhizosphere (p = 0.025). Sphingomonadaceae (B-R > B-BS; p = 0.022), Comamonadaceae (B-R > B-BS; p = 0.006), Nocardiaceae (B-R > B-BS; p = 0.044) and Burkholderiales_incertae_sedis (B-R > B-BS; p = 0.001) were significantly enriched in the beech rhizosphere compared to the surrounding bulk soil. At the genus level, however, few genera were significantly different between the soil and rhizosphere habitats, with significant differences only observed in the Norway spruce samples, whereby *Pseudolabrys* was increased in the bulk soil (p = 0.036) and *Armatimonas* in the rhizosphere (p = 0.028). The *Burkholderia* genus was non-significantly enriched in the rhizosphere of both tree species (p > 0.05). At the OTU level, the rhizosphere and bulk soil bacterial communities were very similar (ANOSIM, p = 0.67 for both tree species), showing that the differences between the rhizosphere and bulk soil compartments were mainly due to variations in the abundance of specific taxa. Only few OTUs assigned to Acidobacteria, Gammaproteobacteria and Bacteroidetes appeared to be significantly structured according to habitat. This high similarity between rhizosphere and bulk soil taxa was also confirmed by ANOSIM analysis performed at the OTU level (p = 0.61 below beech and p = 0.80 below Norway spruce).

For archaeal communities, no statistically significant differences at any taxonomical level between the rhizosphere and bulk soil compartment were detected using ANOVA (p > 0.05 for each taxa tested) and ANOSIM (p = 0.86) analyses, and this absence of structure was confirmed by multivariate analysis (Figure S3). However, members of Methanosarcinales were more abundant in the beech and Norway spruce rhizospheres than in their related bulk soils (B-R = 0.32% and NW-R = 0.61% vs. B-BS = 0.11% and NW-BS = 0.04% of all sequences for the rhizosphere and bulk soil, respectively), and Nitrosophaerales was more abundant in the Norway spruce rhizosphere compared to the bulk soil samples (6.31% vs. 3.72%, respectively). This trend was not observed in the beech samples.

For fungi, both the 18S rRNA and ITS approaches revealed a very limited rhizosphere effect. Indeed, no differences were observed at the phylum and class levels, though multivariate analyses tended to separate the rhizosphere and bulk soil samples under each tree stand (Figures S4 and S5). The 18S rRNA molecular marker revealed a significant enrichment of sequences assigned to Glomerales in the bulk soil compared to the rhizosphere of Norway spruce (0.82% in NW-BS vs 0.07% in NW-R, p = 0.026). At the same phylogenetic level, the ITS marker revealed a significant enrichment of Boletales in the beech rhizosphere (47.2% in B-R vs 21.8% in B-BS, p = 0.003). At the family level, sequences assigned to Sclerodermataceae were enriched in the beech rhizosphere using both markers (18S rRNA: 25.5% in B-R vs 9.3% in B-BS, p = 0.003; ITS: 46.1% in B-R vs 20.8% in B-BS, p = 0.003). Notably, this enrichment was confirmed for the two molecular markers at the genus and OTU levels, showing that *Scleroderma citrinum* was significantly enriched in the beech rhizosphere (p = 0.003). Other species appeared to be enriched in the bulk soil, including *Mortierellales* sp. or *Amanita pantherina* below beech and *Inocybe* sp. and *Jaapia argillacea* below Norway spruce. A global analysis performed at the OTU level also confirmed strong similarities of the rhizosphere and bulk soil fungal communities using the 18S rRNA and ITS approaches (ANOSIM, p = 0.91 with 18S rRNA and p = 0.83 for ITS).

## Discussion

Understanding how tree species affect the distribution of soil microbial communities should allow for a better comprehension of forest ecosystem functioning and sustainability. Such knowledge is particularly important for nutrient-poor soils, in which different tree species will potentially employ different strategies to access the nutrients required for their growth, notably by selecting specific microbial taxa within the vicinity of their roots^39^,^40^. In this study, we hypothesized that deciduous (beech) and evergreen (Norway spruce) tree species of the same age and developing in the same soil will enrich specific taxa from the soil reservoir within the vicinity of their root system.

Although the impact of plant species on soil microbial communities is widely documented^1^,^6^,^7^,^10^,^26^,^28^,^41^, few studies have been performed on tree-associated microbial communities. However, such surveys are essential because by modifying the soil chemistry (pH, nitrogen and cations availability) and microbial biomass and activity, trees have a long-term impact on the environment compared to plants with a shorter lifetime^8^,^27^,^42^. In the last decade, high-throughput sequencing approaches have been applied to decipher the diversity and composition of bacterial and fungal communities in relation to tree species under field and microcosm conditions^10^,^27^,^35^,^43^,^44^, yet such analyses remain rare. Furthermore, the compartment effect (i.e., rhizosphere vs bulk soil) has never analyzed together for the various types of microorganisms. In our study, we utilized quantitative PCR and high-throughput sequencing to reveal quantitative and qualitative affects on archaeal, bacterial and fungal communities below stands of two trees and across soil compartments (rhizosphere vs bulk soil).

By analyzing together archaeal, bacterial and fungal communities in the same samples (bulk soil and rhizosphere) below each tree stand, we demonstrated that these communities have been impacted in by their environment in different ways. Notably, we observed that the structure and density of fungal communities were strongly determined by tree species and poorly by compartment (rhizosphere vs bulk soil). Interestingly, Buée *et al*.^44^ employed sporophore inventory to show at the same experimental site significant host preferences between coniferous and deciduous trees. Although this method offered a relatively biased picture of the true underground fungal diversity, it did permit discrimination between the two tree stands. The impact in our study was observed using both ITS- and 18S rRNA-based approaches, showing the robustness of our analyses. Similarly, the structure of the archaeal communities appeared to be related to the tree species and not to the soil compartment. Together, our results suggest that fungal and archaeal communities are more strongly related to the conditions occurring in each stand, such as edaphic parameters or litter chemistry. Interestingly, Bomberg and Timonen^45^ revealed in a boreal forest soil that the structure and richness of archaeal communities on mycorrhizal roots differ from that on non-mycorrhizal roots, suggesting a potential impact of symbiotic fungi on archaeal communities. However, Karlsson *et al*.^1^ showed that this impact varies when examining one symbiotic fungus associated with a different tree species. In contrast, bacterial communities appear not to be determined by tree species but by their ecological habitat. Indeed, in our study, the rhizosphere bacterial communities under each tree species were different from those of the surrounding soil; Uroz *et al*.^19^ also revealed differentiation among rhizosphere and bulk soil bacterial communities below an oak stand at the same experimental site. Interestingly, by comparing litter and soil bacterial and fungal communities in mixed forest, Urbanova *et al*.^36^ also suggested that the structures of fungal and bacterial communities were not determined by the same drivers. Although their experimental design did not consider rhizosphere and bulk soil compartments, these authors suggested that fungal communities are strongly related to tree species and bacterial communities to root exudates. In our study, correlation analyses revealed higher scores for soil parameters such as pH, C, N and exchangeable Fe for fungi compared to bacteria and archaea. Together, our data show that archaeal, bacterial and fungal communities are not driven by the same environmental parameters.

A specific focus on the rhizosphere compartment revealed minimal affects on microbial communities. Indeed, quantitative PCR revealed no difference between the rhizosphere and bulk soil compartments for total archaea, bacteria and fungi. However, 16S rRNA pyrosequencing and qPCR analyses performed on specific bacterial taxa known for their ability to mobilize nutrients, such as *Burkholderia* and *Collimonas*^18^,^46^, showed significant enrichment in both the beech and Norway spruce rhizospheres. These two bacterial genera have been shown to be very effective at solubilizing minerals, thus increasing plant tree health and nutrition^18^,^38^,^46^. Moreover, the enrichment in these two genera observed in our study is in agreement with the results obtained by a cultivation-dependent approach to compare the mineral weathering ability of soil and rhizosphere bacteria performed at the same experimental site, at the same sampling time and below the same tree stands^16^. Indeed, Collignon *et al*.^16^ revealed that rhizosphere bacteria in both beech and Norway spruce stands are significantly more effective at weathering minerals than are those from the surrounding bulk soil. Of note, phylogenetic affiliation highlighted a dominance of *Burkholderia* strains, especially in the Norway spruce stand. Such enrichment within the root vicinity has also been reported in previous studies at the same forest site^15^,^18^,^19^, showing the stability of this selection. In addition, Calvaruso *et al*.^15^ showed by a culture-dependent approach that the rhizosphere effect is tree species dependent. A comparison of the pyrosequencing data generated in this study for beech and Norway spruce with those obtained at the same experimental site below oak^19^ revealed a similar significant enrichment of *Burkholderia* in the oak rhizosphere but also a tree species effect differentiating oak-, beech- and Norway spruce-associated bacterial communities. Altogether, the data generated in our study suggest that the different tree species examined select specific bacterial taxa that are effective at weathering minerals, especially beech.

Unlike the well-described functional role of bacterial communities, the role of archaea in soil remains to be determined. Indeed, these communities represent an important proportion of soil prokaryotes detected in our study, giving a similar ratio (bacteria/archaea; 10% of the prokaryotes) to that previously reported in acidic forest soils^47^,^48^,^49^,^50^ and particularly in the forest soil of Breuil-Chenue^37^. Specific archaeal taxa have been suggested to be important ammonia-oxidizing organisms under acidic conditions^48^,^49^. Although taxonomic resolution remains limited for soil archaea, our analysis revealed that approximately 8.4% of the archaeal 16S rRNA generated are strongly related to *Nitrosotalea devanaterra*, a recently described obligate acidophilic ammonia oxidizer^48^. This organism’s optimal growth conditions are within a pH range of 4 to 5, which corresponds to the pH conditions occurring at the experimental site of Breuil-Chenue (pH = 3.5 to 4.5). The presence of this archaeal taxon and the lack of detection of bacterial 16S rRNA sequences affiliated with ammonia-oxidizing bacteria (*Nitrosomonas, Nitrospira*) in our dataset suggest that archaeal communities may contribute to ammonia oxidation at the Breuil-Chenue experimental site.

Finally, regarding fungal communities, it was difficult to assess the impact of the environmental parameters tested. Nevertheless, our results clearly showed the involvement of symbiotic ectomycorrhizal fungal species in host preference, with a non-stochastic distribution of fungal species from the following genera: *Amanita*, *Inocybe, Russula* and *Scleroderma*. These data fit very well with those obtained at the same experimental site using sporocarp surveys, mycorrhizal tip collection and amplicon pyrosequencing^44^,^51^. Moreover, multifactorial analyses based on 18S and ITS taxonomic assignments revealed a stronger host specificity for Archaeorhizomyces. This large fungal class, placed within subphylum Taphrinomycotina in Ascomycota^52^, is largely distributed at the global scale but is intimately associated with conifer roots^53^.

## Conclusion

The experimental site of Breuil-Chenue, which is characterized by mono-specific stands planted in one location with different tree species (including *Fagus sylvatica*, *Picea abies*) of the same age, was ideal for testing the tree host effect on soil microbial communities. We combined soil analyses, quantitative PCR and pyrosequencing to show that different tree species growing in the same soil qualitatively and quantitatively impact their soil microbiota. Our data confirmed previous data obtained by a cultivation-dependent approach, highlighting the importance of characterizing soil microbial communities by both cultivation-dependent and -independent approaches. For the first time, we characterized the forest soil archaeal biome and showed it to be an important compartment of the soil microbiome, despite the lack of attention to date. The fact that the archaeal biome is dominated by Thaumarchaea and that these archaea are involved in the recycling of nitrogen highlighted the need to consider soil archaea in future studies. This work provides new data to support the hypothesis that tree species differentially impact soil microbial communities, with archaea and fungi being more strongly determined by tree species and bacteria showing a stronger rhizosphere effect. These results fit very well with the conclusions obtained by Urbanova *et al*.^36^, who suggested that fungal communities are more affected by tree species than are communities of bacteria. Future studies are required to determine how active communities are differently impacted and how they evolve from one season to the next.

## Material and Methods

### Study site and sampling

Soil samples were collected at the Breuil-Chenue experimental forest site (Morvan, 47°18′N, 4°5′E, France). The forest is situated on a plateau at an elevation of 638 m. The native forest was clear-felled in 1976 and replaced by mono-specific plantations distributed in independent plots of 0.1 ha^54^. The soil is classified as Typic Dystrochrept (USDA, 1999) developed on “Pierre qui Vire” granite. The soil and rhizosphere are characterized by pH values ranging from 3.5 to 4^16^. On 17 September 2008, a total of n = 6 soil cores of 10 × 10 × 15 cm dimensions were collected throughout the beech (B; *Fagus sylvatica* L.) and Norway spruce (NW; *Picea abies* Karst) stands; the two tree stands are separated by approx. 100 m. Each soil core in each tree stand was spatially distant from another of approx. 10 m distance. In each tree stand, three distant soil cores were collected in the mineral horizon (10 to 15 cm deep, after removing forest floor debris). The separation of the soil samples into bulk soil (BS samples, n = 3 for each tree stand) and rhizosphere (R samples, n = 3 for each tree stand) was performed in the lab, with a total of 12 samples treated independently. After roots were carefully separated from the soil, the remaining soil was considered to be bulk soil. The soil strongly associated with the root system was collected and considered as rhizosphere soil.

### Soil chemical analyses

For chemical analyses, all soil samples were sieved at 200 μm and homogenized. After oven drying (30 °C), exchangeable cations were extracted in either 1 M KCl (Mg^2+^, Ca^2+^, Na^+^, Fe^3+^, Mn^2+^) or 1 M NH_4_Cl (K^+^), according to Espiau and Peyronel^55^, and determined by ICP-AES (JY180 ULTRACE). The 1 M KCl extract was also titrated using an automatic titrimeter (Mettler TS2DL25) to assess exchangeable H^+^ and Al^3+^ 56. Total carbon (C) and nitrogen (N) contents (both obtained after combustion at 1000 °C) were determined using a Thermo Quest Type NCS 2500 analyzer. The pH of the soil samples was measured in H_2_O at a soil to solution ratio of 1:2 (pH meter Mettler TSDL25). Exchangeable acidity (EA) was calculated by taking the sum of H^+^ and Al^3+^. The cation-exchange capacity (CEC) was calculated by taking the sum of both extracted exchangeable base cations and exchangeable acidity. Base saturation (BS) is the percentage of exchangeable base cations in the CEC (BS = (Mg^2+^, Ca^2+^, Na^+^, K^+^, Fe^3+^, Mn^2+^)/CEC).

### DNA extraction and quantitative PCR

For molecular analyses, all soil samples were sieved at 2 mm and homogenized. Total DNA was extracted from the 12 soil samples using the PowerSoil DNA isolation kit (MO-BIO Laboratories Inc., Carlsbad, CA, USA). The samples were treated according to the manufacturer’s instructions. DNA was quantified on a 1% agarose gel by comparison with a low DNA mass ladder (Invitrogen®). Total DNA was used to quantify the total archaeal, bacterial and fungal communities using archaeal and bacterial 16S and 18S rRNA gene-specific primers (10 μM each; 571F/910R^57^, 968F /1401R^58^, and Fung5f/FF390r^59^, respectively) and SybrGreen detection (iQ SybrGreen Supermix, Biorad). Four-step (45 cycles: 20Sec at 95 °C, 20Sec at the specific annealing temperature, 20Sec at 72 °C and fluorescence acquisition at 82 °C) amplification protocols were performed using previously described protocols with annealing temperatures of 56 °C for bacteria^60^, 50 °C for fungi^59^ and 60 °C for archaea. Absolute quantifications were performed using serial dilutions of standard plasmids containing archaeal or bacterial 16S rDNA or fungal 18S rDNA inserts (from 10^8^ to 10^2^ gene copies/μl). We used the *Collimonas*-specific Taqman assay^61^ for *Collimonas* quantification and Burk3 and BurkR primers^62^ for that of *Burkholderia*. The population sizes of total bacteria, *Burkholderia* or *Collimonas*, are expressed as log [number of 16S rRNA gene copies per gram dry weight soil].

### PCR and pyrosequencing

Amplicon libraries were performed as recommended for 454 pyrosequencing using a combination of two barcoded primers. For Archaea, the primers used were 571F (5′-xxxGCY TAA AGS RIC CGT AGC-3′) and 910R (5′-xxxGCT CCC CCG CCA ATT C-3′) to generate PCR fragments of ca 340 bp^57^. For Bacteria, the primers used were 787r (5′-xxxATTAGAT-ACCYTGTAGTCC-3′) and 1073f (5′-xxxACGAGCTGACGACARCCATG-3′)^57^,^58^ to generate PCR fragments of ca 250 bp. For fungi, the ITS and 18S rRNA regions were amplified, using ITS1F (5′-xxxCTTGGTCATTTAGAGGAAGTAA-3′) and ITS2 (5′-xxxGCTGCGTTCTTCATCGATGC-3′)^63^,^64^, and 817F (5′-xxxTTAGCATGGAATAATRRAATAGGA-3′) and 1196R (5′-xxxTCTGGACCTGGTGAGTTTCC-3′)^65^, where xxx is a identification barcoding key. The PCR were performed using 50 ng of genomic DNA as template. The PCR conditions used were 94 °C for 4 min, 30 cycles of 94 °C, 30S denaturation; 50 °C, 1 min annealing and 72 °C, 1 min 30S extension; followed by 72 °C, 10 min. Ten separate amplification reactions of 50 μl for each sample were pooled and purified as recommended using QIAquick PCR purification columns (Qiagen). The amplicon length and concentration was estimated and all 48 amplicon libraries were mixed to achieve a 9:1 ratio of bacterial sequences to archaeal/fungal sequences. This mix was used for pyrosequencing on the Genome Sequencer (GS) FLX 454 System (Roche) at Beckman Coulter Genomics (Danvers, MA, USA) which resulted in 432,000 reads (details are provided in Table S1) which passed the length and quality criteria.

### Bioinformatic analyses

DNA sequences were processed using the Trim.seqs command of Mothur^66^ (average quality score of 25) allowing 2 mismatches in the primer and 0 in the identification barcoding key. Sequences from forward and reverse primers were sorted according to their primer sequences using the trim.seqs and split.groups commands of Mothur. Sequences from reverse primers were then converted into their reverse complements. All the sequences were aligned at the start of the forward primer sequence. For each amplicon type, sequences were sorted according to a minimum and maximum size (bacteria, 250 to 300 bp; Archaea, 350 to 400 bp; Fungal 18S rRNA, 400 to 460 bp) using the trim.seqs command of Mothur. For Dikarya sequences, the internal transcribed spacer 2 (ITS2) region was extracted using Fungal ITS X extractor^67^ and sequences shorter than 100 bp were removed.

For each organism-type considered, all sequences were pooled and sorted by decreasing length as recommended for Usearch clustering^68^. Operational taxonomic units (OTUs) were generated from these reads using the cluster_smallmem command of Usearch v 6.0.307^68^ at a 97% similarity threshold. Phylogenetic assignment was determined for each OTU representative sequence (consensus sequence) using the Basic Local Alignment Search Tool (BLAST) algorithm v 2.2.23^69^ against the Ribosomal Data Project (RDP) database release 10.3^70^ for bacterial and archaeal sequences. For fungi, the phylogenetic assignment was determined against UNITE database release 5.0^71^ and the NCBI. All assignation were determined using a minimum e-value cut-off of 1e-5.

For each organism-type considered, matrices containing the sequence abundances of different OTUs in each soil sample were created. To make comparable samples with different number of sequences generated for each sample and each organism type (details provided in Table S1), matrices were subsampled with a same number of sequences for each soil sample using the sub.sample command of Mothur. This number of sequences was determined to conserve a minimum of three independent replicates for each treatment considered in the study. This subsampling procedure allowed considering 2,869 sequences for Bacteria, 932 for Archaea, 1153 and 445 for fungal ITS and 18S rRNA, respectively. For Archaea, one replicate reached only 261 sequences (spruce rhizosphere) and was not considered for OTU and richness index calculations.

### Statistical analysis

Quantification data from quantitative PCR were compared as Log gene copy using a Student-t test. The effect of the tree species or soil compartment on the abundance of the different taxa or genera considered was tested by ANOVA analyses. To determine the taxonomic differences between the niche (bulk soil or rhizosphere) and of the tree stand (beech or Norway spruce), analysis of variance (ANOVA) at a threshold level of *P* = 0.05 and by the Fisher test were applied on the relative distribution values after an arcsine transformation. Mantel tests (10,000 permutations, Pearson correlations) were performed to test correlations between soil parameters and phylum distribution of each organism type based on Bray-Curtis distance dissimilarity matrices. All statistical analyses were performed using the XLstat2011 software (Addinsoft, Paris, France). Analyses of similarity (ANOSIM) based on Bray-Curtis distances and principal coordinate analysis (PcoA) were performed at the OTU level using Mothur for each type of microorganism considered.

## Additional Information

Accession codes: The sequences generated in this study have been deposited on the Sequence Read Archive (SRA; sra@ftp-private.ncbi.nlm.nih.gov) service of the GenBank database under the accession numbers : biosample SUB981017 and BioProject PRJNA169429.

**How to cite this article**: Uroz, S. *et al*. Specific impacts of beech and Norway spruce on the structure and diversity of the rhizosphere and soil microbial communities. *Sci. Rep.* **6**, 27756; doi: 10.1038/srep27756 (2016).

## Supplementary Material

Supplementary Information

## Figures and Tables

**Table 1:** Soil chemical analyses performed on the rhizosphere (R) and bulk soil (BS) samples collected below the beech (B) and Norway spruce (NW) stands.

Origin	pH	C	N	C/N	K	Ca	Fe	Mg	Mn	Na	H^+^	Al^3+^	EA^a^	CEC^b^
		g/kg			Cmolc/kg									
NW-R	3.61^a^	99.71^a^	5.16^a^	19.15^a^	0.54^a^,^b^	0.48^a^	0.86^a^	0.37^a^	0.08^a^	0.24^a^	1.62^a^	12.42^a^	14.03^a^	16.52^a^
NW-BS	3.97^b^	68.47^a^	3.77^a^	18.06^a^	0.22^c^	0.59^a^	0.33^b^	0.32^a^	0.08^a^	0.56^a^	0.42^b^	12.29^a^	12.71^a^	14.74^a^
B-R	3.86^b^	83.28^a^	4.48^a^	18.06^a^	0.57^a^	0.56^a^	0.44^b^	0.33^a^	0.11^a^	0.27^a^	1.22^a^,^b^	11.32^a^	12.53^a^	14.70^a^
B-BS	3.86^b^	72.35^a^	3.84^a^	18.84^a^	0.35^b^,^c^	0.57^a^	0.38^b^	0.27^a^	0.09^a^	0.22^a^	0.96^a^,^b^	12.93^a^	13.89^a^	15.72^a^

Different letters between terraces (a or b) indicate significant differences, according to a one-factor ANOVA and a Bonferronie-Dunn test (p < 0.05).

^a^EA: Exchangeable acidity.

^b^CEC: Cationic exchange capacity.

**Table 2:** Presentation of the significant Pearson correlations among microbial taxa distribution at the phylum level and the soil parameters.

Marker	Phylum	pH	N	C	K	Al	Ca	Fe	Mg	H+
*16*S *rRNA bacteria*	Acidobacteria		−0.729*							
	Bacteroidetes				0.768**					
	Chloroflexi	0.712*	−0.718*							
	Gemmatimonadetes					−0.766**				
	Nitrospira					−0.721*				
										
*16*S *rRNA archaea*	Crenarchaeota								−0.753*	
										
*Fungal ITS*	Ascomycota	−0.847**	0.724*	0.806**				0.859***		
	Basidiomycota	0.781**						−0.741*		
	Chytridiomycota	−0.747*								0.748*
										
*Fungal 18*S *rRNA*	Ascomycota	−0.795**						0.801**		
	Cryptomycota						0.806**			
	Basidiomycota	0.807**						−0.793**		

Correlation was done considering all the samples (n = 12) and using the relative distribution of the sequences. Correlation scores are presented with asterisk according to the p value (*p < 0.05; **p < 0.005; ***p < 0.0005).
