# Supplementary material for: Specific impacts of beech and Norway spruce on the structure and diversity of the rhizosphere and soil microbial communities
Source: Sci Rep. 2016 Jun 15;6:27756. doi: 10.1038/srep27756 (PMC4908602; doi:10.1038/srep27756)
Supplement: Supplementary Information [file srep27756-s1.pdf]

Supplementary figures of the manuscript entitled:

Specific impacts of beech and Norway spruce on the structure and diversity of the rhizosphere and soil microbial communities

Uroz S.[1ab,2], Oger P.[3], Tisserand E.[1], Cébron A.[4,5], Turpault M-P.[2], Buée M.[1], De Boer W.[6], Leveau J.H.J.[7], and P. Frey-Klett[1]

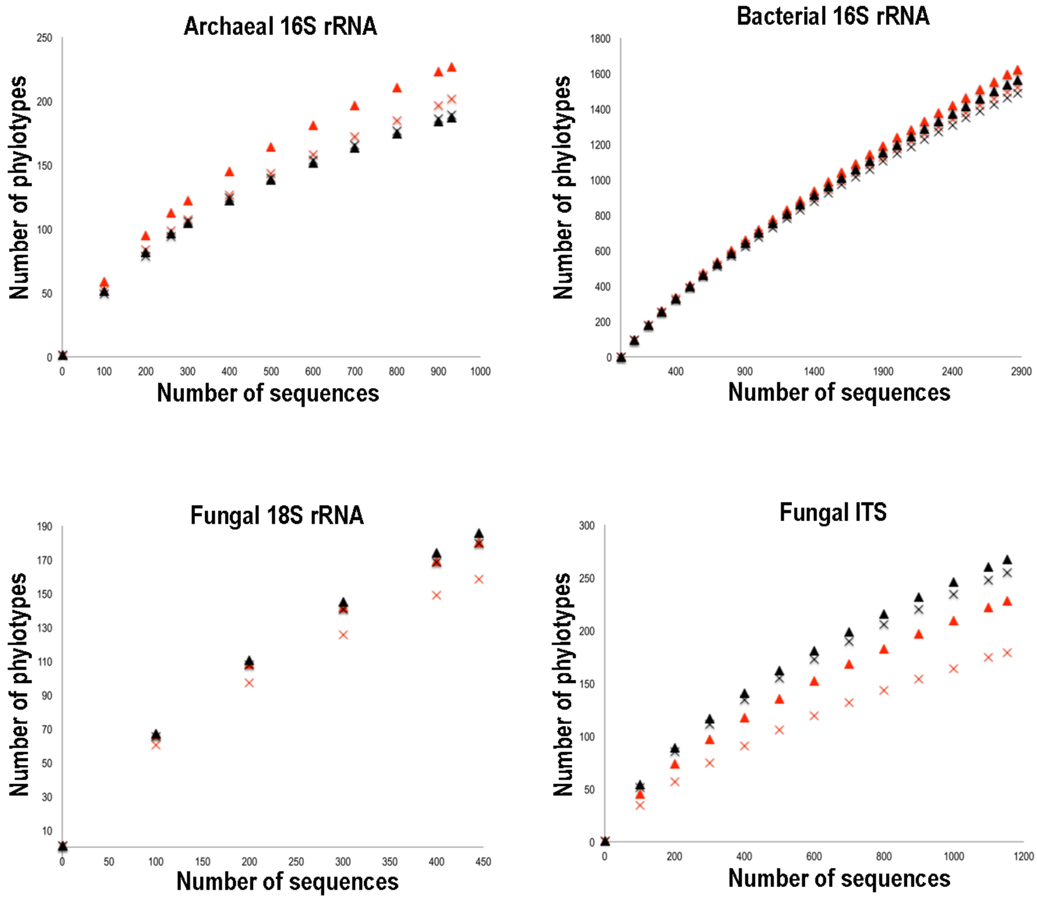

Figure S1: Rarefaction analyses. For each organism type, red symbols correspond to beech (B) samples coming from the bulk soil (red triangle) and the rhizosphere (red cross). Black symbols correspond to Norway spruce (NW) samples coming from the bulk soil (black triangle) and the rhizosphere (black cross).

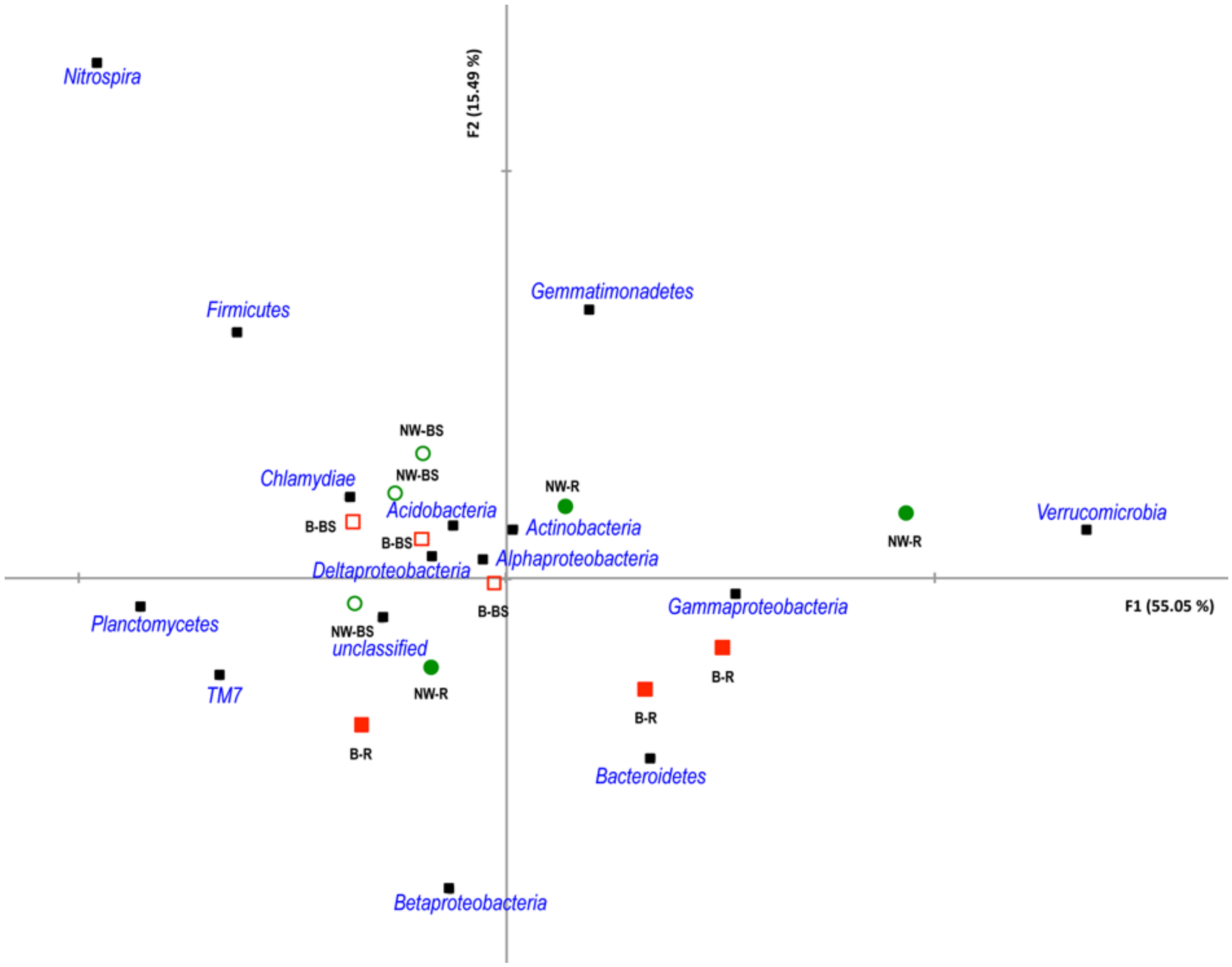

Figure S2: Multifactorial analysis of the relative proportion of the major bacterial phyla present in the rhizosphere and surrounding bulk soil under beech and Norway spruce stands. Green circle symbols correspond to Norway Spruce (NW) samples coming from the bulk soil (NW-BS ; open circle) and the rhizosphere (NW-R ; filed circle). Red square symbols correspond to Beech (B) samples coming from the bulk soil (B-BS ; open square) and the rhizosphere (B-R ; filed square). Vector position of the main phyla is presented by black squares.

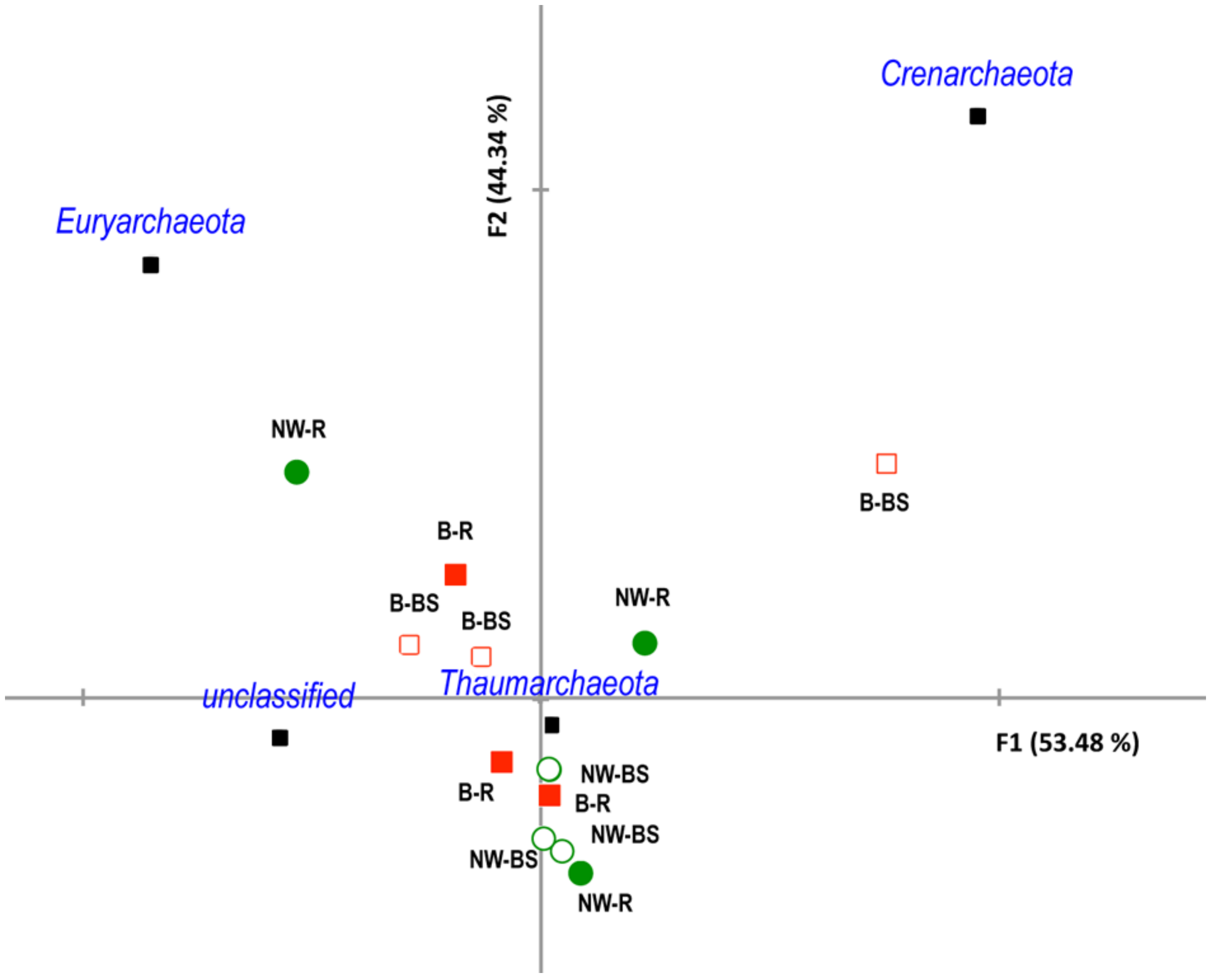

Figure S3: Multifactorial analysis of the relative proportion of the major archaeal phyla present in the rhizosphere and surrounding bulk soil under beech and Norway spruce stands. Green circle symbols correspond to Norway Spruce (NW) samples coming from the bulk soil (NW-BS ; open circle) and the rhizosphere (NW-R ; filed circle). Red square symbols correspond to Beech (B) samples coming from the bulk soil (B-BS ; open square) and the rhizosphere (B-R ; filed square). Vector position of the main phyla is presented by black squares.

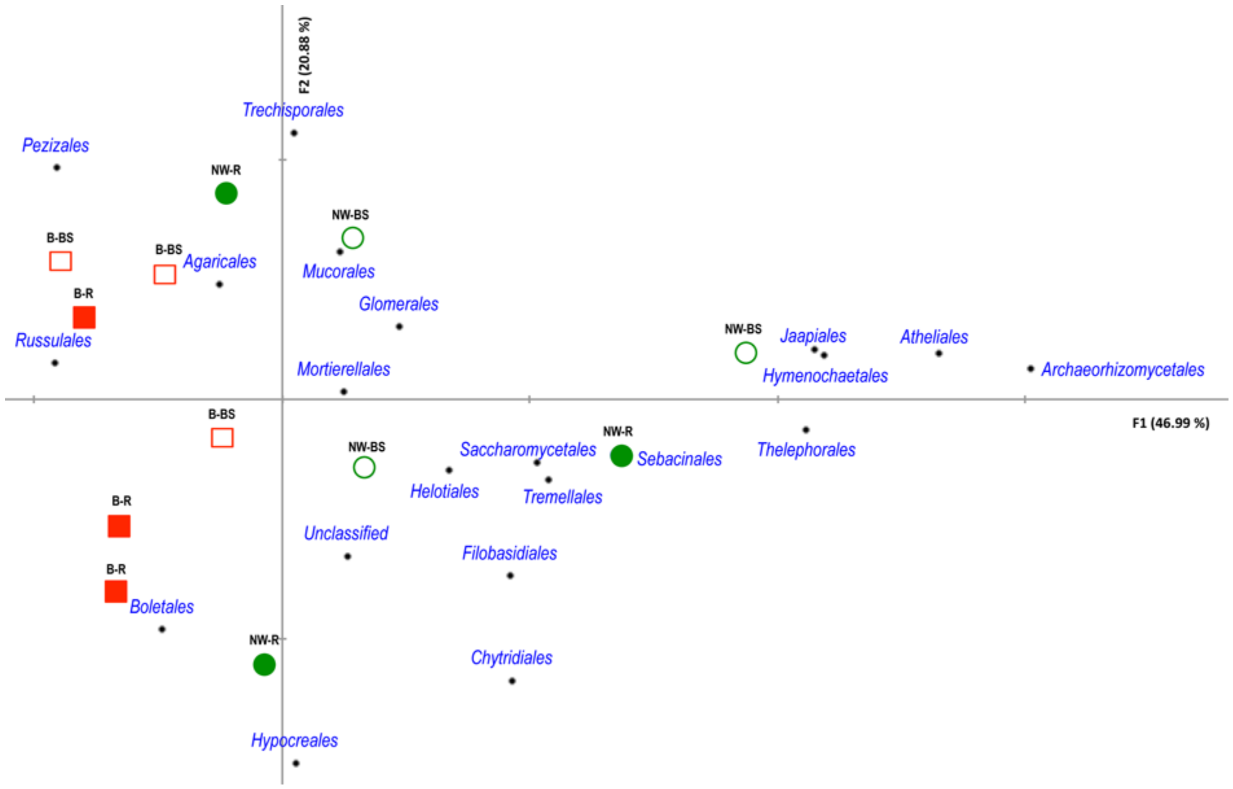

Figure S4: Multifactorial analysis of the relative proportion of the major fungal phyla (according to 18S rRNA assignation) present in the rhizosphere and surrounding bulk soil under beech and Norway spruce stands. Green circle symbols correspond to Norway Spruce (NW) samples coming from the bulk soil (NW-BS ; open circle) and the rhizosphere (NW-R ; filed circle). Red square symbols correspond to Beech (B) samples coming from the bulk soil (B-BS ; open square) and the rhizosphere (B-R ; filed square). Vector position of the main phyla is presented by black squares.

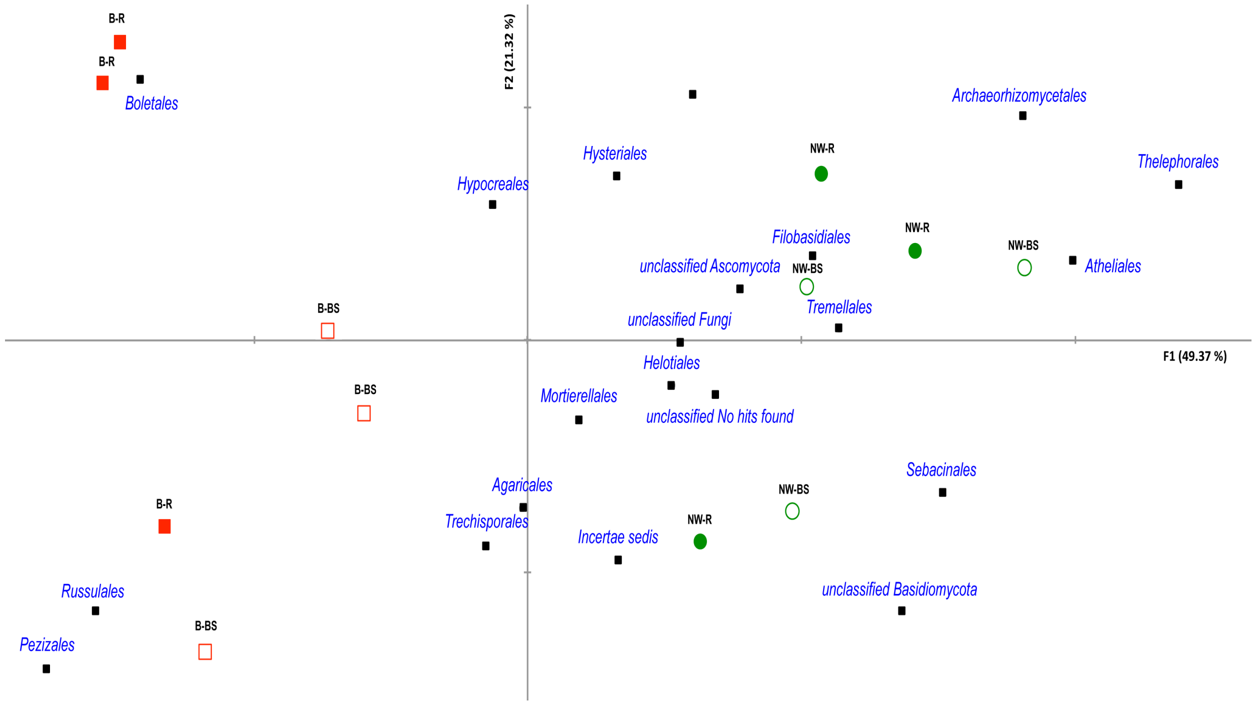

Figure S5: Multifactorial analysis of the relative proportion of the major fungal phyla (according to ITS assignation) present in the rhizosphere and surrounding bulk soil under beech and Norway spruce stands. Green circle symbols correspond to Norway Spruce (NW) samples coming from the bulk soil (NW-BS ; open circle) and the rhizosphere (NW-R ; filed circle). Red square symbols correspond to Beech (B) samples coming from the bulk soil (B-BS ; open square) and the rhizosphere (B-R ; filed square). Vector position of the main phyla is presented by black squares.

Table S1: presentation of the raw data

q25= quality of 25

**BACTERIA**	sample	raw	q25
H1R	1	4315	2912
H1BS	2	10986	7846
H2R	3	21077	14517
H2BS	4	49961	37617
H4R	5	23987	17819
H4BS	6	27595	19908
E2R	7	4442	2869
E2BS	8	32206	23537
E3R	9	28678	21542
E3BS	10	19358	13960
E4R	11	33256	24614
E4BS	12	24033	16335
	total	*279894*	*203476*

**ARCHAEA**	sample	raw	q25
H1R	1	3128	932
H1BS	2	3367	966
H2R	3	10692	2471
H2BS	4	20118	7127
H4R	5	4806	1702
H4BS	6	7297	2287
E2R	7	2433	261
E2BS	8	5117	1590
E3R	9	7727	3221
E3BS	10	9729	3155
E4R	11	3033	1364
E4BS	12	7273	1310
	total	*84720*	*26386*

**18S rRNA**	sample	raw	q25
H1R	1	1167	553
H1BS	2	2779	1369
H2R	3	5899	2889
H2BS	4	3542	1803
H4R	5	850	445
H4BS	6	4780	1561
E2R	7	1534	772

E2BS	8	1023	501
E3R	9	4303	1968
E3BS	10	1555	671
E4R	11	3347	1505
E4BS	12	1258	570
	total	*32037*	*14607*

**ITS**	sample	raw	q25	q25 and ITSx
H1R	1	1802	1772	1374
H1BS	2	1524	1494	1153
H2R	3	3082	3044	2165
H2BS	4	4234	4155	2989
H4R	5	2205	2174	1538
H4BS	6	2152	2136	1783
E2R	7	1640	1607	1365
E2BS	8	2983	2929	2270
E3R	9	5456	5397	2193
E3BS	10	2844	2800	2258
E4R	11	4360	4302	3476
E4BS	12	2524	2493	1976
	total	*34806*	*34303*	*24540*

Table S2: Details of the diversity and richness values

A/ in average

Archaea	NW-BS	NW-R	B-BS	B-R
OTU Obs	183	198	224	204
shannon	4.09	4.17	4.49	4.30
chao	293.66	306.06	363.25	337.17
**Bacteria**				
OTU Obs	1578	1478	1568	1523
shannon	6.92	6.83	6.93	6.91
chao	5064.12	4702.17	5012.04	4390.78
**Fungi (18S)**				
OTU Obs	187	180	181	156
shannon	4.68	4.63	4.69	4.40
chao	384.49	371.40	356.10	305.96
**Fungi (ITS)**				
OTU Obs	297	250	278	174
shannon	4.33	4.18	3.91	3.11
chao	598.2	545.4	474.0	417.9

B/ in detail sample per sample

**ARCHAEA**	**chao**	chao_lci	chao_hci	**shannon**	shannon_lci	shannon_hci	**OTU Richness**
E2BS	307.52	260.54	388.67	3.99	3.86	4.11	194
E3BS	310.84	259.63	400.15	4.21	4.11	4.32	184
E4BS	262.63	222.23	336.18	4.06	3.95	4.17	231
E3R	366.11	308.37	462.29	4.35	4.24	4.46	172
E4R	246.01	203.79	326.08	3.98	3.88	4.09	165
H1BS	410.30	350.70	505.70	4.71	4.62	4.81	250
H4BS	313.16	266.47	393.48	4.28	4.18	4.39	243
H2BS	366.30	311.17	457.21	4.48	4.38	4.58	218
H1R	391.22	334.22	483.84	4.67	4.58	4.77	193
H2R	331.11	272.96	431.98	4.26	4.16	4.36	205
H4R	289.18	234.46	388.47	3.98	3.87	4.08	176

**BACTERIA**	**chao**	chao_lci	chao_hci	**shannon**	shannon_lci	shannon_hci	**OTU Richness**
E2BS	5305.53	4702.08	6028.23	7.05	7.01	7.09	1607
E3BS	3076.93	2745.08	3483.15	6.59	6.54	6.64	1199
E4BS	6809.89	5994.47	7783.19	7.11	7.07	7.16	1308
E2R	2590.73	2330.94	2910.14	6.57	6.53	6.61	1462
E3R	4774.98	4199.65	5472.25	6.80	6.75	6.85	1818
E4R	6740.79	5940.64	7695.20	7.14	7.09	7.18	1772
H1BS	5394.55	4791.24	6114.83	7.06	7.02	7.10	1659
H2BS	6350.89	5587.33	7265.22	7.06	7.02	7.11	1570
H4BS	3290.68	2929.95	3731.50	6.67	6.63	6.72	1718
H1R	4243.23	3804.83	4767.44	6.96	6.92	7.00	1402
H2R	3674.56	3279.34	4153.37	6.82	6.77	6.86	1327
H4R	5254.56	4633.04	6002.77	6.94	6.90	6.99	1598

**18S rRNA**	**chao**	chao_lci	chao_hci	**shannon**	shannon_lci	shannon_hci	**OTU Richness**
E2BS	376.70	302.55	500.29	4.81	4.70	4.91	193
E3BS	370.04	292.55	500.83	4.65	4.54	4.76	166
E4BS	406.71	318.00	554.84	4.57	4.44	4.70	185
E2R	329.51	264.64	440.89	4.66	4.55	4.76	178
E3R	333.52	263.02	455.56	4.48	4.35	4.60	184
E4R	451.17	351.40	615.57	4.76	4.65	4.88	195
H1BS	400.28	324.21	524.38	4.90	4.80	5.01	199
H2BS	372.28	292.20	508.34	4.65	4.54	4.76	134
H4BS	295.74	237.36	398.17	4.52	4.41	4.63	174
H1R	220.01	179.51	295.72	4.02	3.87	4.16	164
H2R	361.70	282.83	497.13	4.58	4.47	4.70	171
H4R	336.16	267.48	453.23	4.61	4.50	4.71	170

**ITS**	**chao**	chao_lci	chao_hci	**shannon**	shannon_lci	shannon_hci	**OTU Richness**
E2BS	568.34	449.60	757.07	4.10	3.99	4.21	323
E3BS	538.73	429.39	714.77	4.30	4.20	4.40	215
E4BS	687.60	554.72	891.23	4.60	4.50	4.70	254
E2R	457.93	363.23	614.56	3.82	3.71	3.94	240
E3R	520.65	416.82	686.81	4.08	3.98	4.19	313
E4R	657.75	534.00	847.64	4.63	4.53	4.73	294
H1BS	480.60	396.82	610.90	4.01	3.90	4.12	322
H2BS	512.60	403.25	690.44	3.95	3.84	4.06	176
H4BS	428.75	344.28	566.73	3.78	3.67	3.89	227
H1R	474.78	351.41	687.97	2.93	2.80	3.07	150
H2R	356.11	271.94	504.11	2.79	2.66	2.93	286
H4R	422.67	330.23	578.74	3.59	3.48	3.70	196

Table S3: OTU based analysis

A/ Details of bacterial OTUs and associated statitics

OTUId	phylum	class	order	family	genus	H1R	E3BS	E4R	E4BS	H1BS	H2R	H2BS	H4R	H4BS	E2R	E2BS	E3R	sum	%
Cluster6847;	Acidobact	Acidobacteria_Gp2			Gp2	25	66	2	10	35	29	36	39	30	7	19	72	370	1.07
Cluster1810З	Acidobact	Acidobacteria_Gp2			Gp2	36	11	23	34	37	26	21	26	51	21	12	13	311	0.90
Cluster17992	Acidobact	Acidobacteria_Gp2			Gp2	15	13	34	32	18	33	37	25	21	6	16	12	262	0.76
Cluster14562	Acidobact	Acidobacteria_Gp2			Gp2	24	15	18	26	16	21	23	27	33	7	25	23	258	0.75
Cluster2850;	Acidobact	Acidobacteria_Gp1			Granulicella	19	29	17	21	21	9	15	17	12	30	16	7	213	0.62
Cluster4524;	Acidobact	Acidobacteria_Gp2			Gp2	10	68	2	4	15	4	2	13	14	0	10	62	204	0.59
Cluster11489	Proteobacteria					9	50	10	4	7	17	5	10	14	22	19	26	193	0.56
Cluster21203	Acidobact	Acidobacteria_Gp1			Edaphobacter	16	10	13	13	17	13	14	15	15	19	20	23	188	0.55
Cluster18940	Acidobact	Acidobacteria_Gp2			Gp2	17	18	28	25	6	17	17	18	7	3	8	4	168	0.49
Cluster12709	Acidobact	Acidobacteria_Gp1				21	22	4	1	6	13	10	26	19	1	7	23	153	0.44
Cluster4743;	Acidobact	Acidobacteria_Gp1			Edaphobacter	7	6	14	5	16	14	13	11	9	15	17	5	132	0.38
Cluster23035	Proteobac	Gamm	Xantl	Sinobac	Steroidobacter	2	2	21	4	6	19	11	7	2	29	13	6	122	0.35
Cluster7691;size=778						2	11	1	3	10	10	11	21	33	4	6	7	119	0.35
Cluster3506;size=796						12	21	5	8	5	3	14	15	13	3	6	13	118	0.34
Cluster16237	Acidobact	Acidobacteria_Gp2			Gp2	6	6	11	11	9	13	19	9	16	2	10	5	117	0.34
Cluster2589;	Acidobact	Acidobacteria_Gp2			Gp2	5	4	25	24	3	10	11	7	11	2	8	5	115	0.33
Cluster4397;size=806						2	8	8	11	5	21	19	14	11	2	8	6	115	0.33
Cluster14571	Acidobact	Acidobacteria_Gp1				10	15	1	4	7	10	11	18	2	9	13	11	111	0.32
Cluster7758;	Proteobac	Alphaproteobacteria				2	18	1	8	3	9	11	10	32	1	1	10	106	0.31
Cluster119;si	Acidobact	Acidobacteria_Gp2			Gp2	5	25	6	5	6	7	10	6	11	0	2	16	99	0.29
Cluster23427	Proteobac	Gamm	Xantl	Sinobac	Steroidobacter	0	0	0	2	15	7	7	13	8	43	0	0	95	0.28
Cluster6540;	Acidobact	Acidobacteria_Gp2			Gp2	4	10	1	11	6	6	9	14	12	0	4	16	93	0.27
Cluster179;si	Proteobac	Alphap	Rhizobiales			9	2	10	7	11	10	4	5	12	9	6	5	90	0.26
Cluster8857;	Verrucom	Opituta	Opitu	Opituta	Opitutus	10	6	4	1	0	13	1	2	7	33	6	6	89	0.26

Cluster14049	Acidobact	Acidobacteria_Gp1				9	7	11	2	7	9	8	3	7	14	4	8	89	0.26
Cluster19548	Proteobac	Gamm	Xantl	Sinobac	Steroidobacter	1	6	5	12	10	9	3	3	3	17	11	7	87	0.25
Cluster8;size	Proteobac	Alphap	Rhodospirillales			7	1	10	7	6	6	17	3	4	12	13	0	86	0.25
Cluster3306;s	Proteobac	Gammaproteobacteria				13	1	11	8	11	12	11	4	2	6	5	2	86	0.25
Cluster1045;s	Actinobac	Actinol	Acidi	Acidimi	Aciditerrimona	4	10	9	4	6	6	7	7	6	13	8	4	84	0.24
Cluster3862;s	Proteobac	Gammaproteobacteria				20	11	3	3	7	4	4	4	11	0	2	14	83	0.24
Cluster16175	Acidobact	Acidobacteria_Gp1			Gp1	1	1	11	15	9	6	2	0	5	21	12	0	83	0.24
Cluster5604;s	Acidobacteria					4	9	1	4	9	2	9	8	15	2	11	8	82	0.24
Cluster16075	Acidobact	Acidobacteria_Gp1				0	1	2	6	19	10	0	4	21	5	14	0	82	0.24
Cluster19322	Acidobact	Acidobacteria_Gp2			Gp2	9	19	5	9	8	12	12	1	3	0	0	1	79	0.23
Cluster2845;s	Actinobac	Actinol	Actinomycetales			6	4	2	1	10	4	9	10	9	6	10	7	78	0.23
Cluster7100;s	Proteobac	Alphap	Rhizo	Xanthol	Pseudolabrys	7	2	5	13	9	7	7	6	5	2	11	1	75	0.22
Cluster1707;s	Actinobac	Actinol	Actinomycetales			6	2	4	0	8	10	3	13	10	7	5	6	74	0.21
Cluster15933	Acidobact	Acidobacteria_Gp2			Gp2	6	6	5	3	15	11	6	4	8	1	2	5	72	0.21
Cluster27;size	Verrucom	Opituta	Opitu	Opituta	Opitutus	18	0	2	0	6	24	6	3	2	10	0	0	71	0.21
Cluster2830;s	Proteobac	Deltaproteobacteria				5	8	5	4	10	3	0	1	13	5	9	7	70	0.20
Cluster14115	Acidobact	Acidobacteria_Gp1				4	9	0	1	3	8	2	14	16	3	3	7	70	0.20
Cluster200;si	Acidobact	Acidobacteria_Gp3			Gp3	3	1	9	5	2	4	2	2	4	26	9	2	69	0.20
Cluster3529;s	Acidobact	Acidobacteria_Gp2			Gp2	4	14	3	3	1	9	10	0	1	1	6	17	69	0.20
Cluster5329;s	Proteobac	Gamm	Xantl	Sinobac	Steroidobacter	7	3	11	7	2	1	2	1	4	9	16	5	68	0.20
Cluster171;si	Proteobac	Betapr	Burkholderiales			7	1	1	1	1	11	1	30	9	2	0	3	67	0.19
Cluster3010;s	Proteobac	Alphap	Rhodospirillales			6	1	4	4	11	3	7	1	4	6	19	1	67	0.19
Cluster10446	Proteobac	Betaproteobacteria				3	4	4	11	1	11	7	5	6	1	8	6	67	0.19
Cluster26697	Actinobac	Actinol	Actinomycetales			3	2	0	5	9	10	11	6	6	8	4	3	67	0.19
Cluster11223	Proteobac	Alphap	Rhizobiales			5	10	8	2	7	3	1	1	12	7	3	6	65	0.19
Cluster12566	Acidobact	Acidobacteria_Gp2			Gp2	9	6	4	3	4	2	3	8	6	1	10	8	64	0.19
Cluster3358;s	Acidobact	Acidobacteria_Gp2			Gp2	3	5	3	1	3	4	4	6	19	4	6	5	63	0.18
Cluster7861;s	Acidobact	Acidobacteria_Gp2			Gp2	1	7	4	7	3	2	5	2	7	8	3	14	63	0.18
Cluster19858	Acidobact	Acidobacteria_Gp2			Gp2	10	8	7	5	19	7	5	0	0	0	2	0	63	0.18

Cluster57;siz	Acidobact	Acidobacteria_Gp1			Gp1	11	2	7	2	7	6	2	2	2	18	0	3	62	0.18
Cluster7883;	Proteobac	Gamm	Xantl	Sinobac	Steroidobacter	14	2	5	4	8	5	2	5	2	5	7	3	62	0.18
Cluster1510S	Acidobact	Acidobacteria_Gp2			Gp2	4	10	3	4	7	6	1	6	10	4	2	5	62	0.18
Cluster727;si	Proteobac	Gammaproteobacteria				11	7	3	9	6	3	3	5	6	0	6	2	61	0.18
Cluster2797;	Acidobact	Acidobacteria_Gp3				8	2	3	3	5	3	2	1	3	15	7	9	61	0.18
Cluster5715;	Acidobact	Acidobacteria_Gp2			Gp2	0	3	7	19	1	2	1	8	10	2	5	3	61	0.18
Cluster7356;	Proteobac	Alphap	Rhodospirillales			3	4	10	13	3	5	2	9	8	1	1	2	61	0.18
Cluster10871	Proteobac	Alphap	Rhizc	Bradyrhizobiaceae		5	10	5	3	2	9	5	3	4	4	1	10	61	0.18
Cluster9;size	Proteobac	Alphap	Rhodospirillales			3	6	3	2	0	10	10	8	10	0	2	6	60	0.17
Cluster4538;	Proteobac	Alphap	Rhizobiales			3	2	0	10	4	6	10	9	7	1	5	2	59	0.17
Cluster772;si	Acidobact	Acidobacteria_Gp1				6	6	2	4	8	8	3	6	7	3	1	4	58	0.17
Cluster4050;	Acidobact	Acidobacteria_Gp2			Gp2	3	4	4	10	1	6	6	4	8	1	6	5	58	0.17
Cluster1455C	Acidobact	Acidobacteria_Gp1				7	0	4	8	12	3	7	3	0	8	6	0	58	0.17
Cluster17748	Proteobac	Gamm	Xantl	Sinobac	Steroidobacter	9	9	1	12	0	1	1	2	0	4	8	11	58	0.17
Cluster1759;	Verrucom	Opitut	Opitu	Opituta	Opitutus	3	2	7	0	1	5	1	2	4	22	5	5	57	0.17
Cluster16659;size=285						1	0	5	5	11	13	0	0	15	1	6	0	57	0.17
Cluster356;si	Proteobac	Betapr	Burkl	Burkhol	Burkholderia	17	2	8	3	10	2	2	4	4	2	0	2	56	0.16
Cluster2439;	Proteobac	Gamm	Xantl	Sinobac	Steroidobacter	0	10	6	2	1	8	2	5	7	4	4	7	56	0.16
Cluster3009;	Acidobact	Acidobacteria_Gp3			Gp3	2	15	1	2	2	4	4	8	9	3	2	4	56	0.16
Cluster3226;	Proteobac	Alphap	Caulc	Caulobacteraceae		12	1	2	2	11	1	4	2	0	9	10	2	56	0.16
Cluster5271;	Acidobact	Acidobacteria_Gp2			Gp2	3	4	3	2	3	6	2	10	8	3	8	4	56	0.16
Cluster14783	Proteobac	Betapr	Burkl	Burkhol	Burkholderia	3	3	7	6	4	4	3	8	9	1	2	6	56	0.16
Cluster2230;	Verrucom	Opitut	Opitu	Opituta	Opitutus	6	3	0	1	2	7	1	0	2	30	0	3	55	0.16
Cluster6361;	Acidobact	Acidobacteria_Gp2			Gp2	2	12	0	2	7	2	2	9	11	0	4	4	55	0.16
Cluster14148;size=314						3	5	2	3	6	5	10	9	3	0	5	4	55	0.16
Cluster23;size=331						2	6	5	8	1	4	3	5	6	5	5	4	54	0.16
Cluster2553;	Acidobact	Acidobacteria_Gp2			Gp2	0	13	0	4	0	3	2	12	9	0	2	9	54	0.16
Cluster4922;	Verrucom	Opitut	Opitu	Opituta	Opitutus	1	23	0	0	0	0	1	0	0	2	1	26	54	0.16
Cluster1654;	Actinobac	Actinol	Actinomycetales			4	7	1	3	4	3	9	2	4	9	4	3	53	0.15

Cluster1900;	Proteobac	Gammaproteobacteria				16	1	2	3	14	8	4	2	2	0	0	1	53	0.15
Cluster2857;	Acidobact	Acidobacteria_Gp1			Gp1	8	3	6	5	9	4	2	1	0	8	4	3	53	0.15
Cluster7790;	Acidobact	Acidobacteria_Gp1				2	8	9	6	0	5	7	2	5	4	2	3	53	0.15
Cluster23167	Proteobac	Gammaproteobacteria				0	0	0	0	16	9	6	10	11	0	0	1	53	0.15
Cluster6175;	Acidobact	Acidobacteria_Gp2			Gp2	0	19	0	3	6	0	1	3	1	0	0	19	52	0.15
Cluster2340;	Acidobact	Acidobacteria_Gp1				3	9	1	0	3	3	5	9	8	1	2	7	51	0.15
Cluster5050;	Acidobacteria					1	8	2	0	4	5	3	8	6	0	9	5	51	0.15
Cluster12173	Verrucom	Opituta	Opitu	Opituta	Opitutus	21	0	0	1	6	16	3	2	2	0	0	0	51	0.15
Cluster19869	Acidobact	Acidobacteria_Gp2			Gp2	3	0	17	27	0	2	1	0	0	0	1	0	51	0.15
Cluster3098;	Actinobac	Actinol	Actinomycetales			3	1	0	3	3	5	8	13	6	2	6	0	50	0.15
Cluster7920;	Acidobact	Acidobacteria_Gp3				5	1	3	3	4	6	2	6	2	12	4	2	50	0.15
Cluster18579	Acidobact	Acidobacteria_Gp2			Gp2	0	0	0	1	0	0	5	5	17	20	1	1	50	0.15
Cluster21922	Acidobact	Acidobacteria_Gp1			Edaphobacter	4	1	5	5	4	2	5	4	3	9	5	3	50	0.15
Cluster1257;	Acidobact	Acidobacteria_Gp1				3	12	1	2	1	6	6	4	7	0	2	5	49	0.14
Cluster2571;	Proteobac	Alphap	Rhizo	Xanthol	Labrys	1	12	0	1	1	2	1	6	9	5	7	4	49	0.14
Cluster8030;	Proteobac	Gamm	Xantl	Sinobac	Steroidobacter	2	4	3	5	6	3	3	3	1	11	5	3	49	0.14
Cluster1062;	Acidobact	Acidobacteria_Gp2			Gp2	3	6	1	1	7	3	4	6	7	0	6	4	48	0.14
Cluster1725;	Proteobac	Gamm	Xantl	Sinobac	Steroidobacter	3	1	4	2	5	1	1	0	2	18	9	2	48	0.14
Cluster1800;size=265						6	4	7	5	4	3	1	1	1	5	6	5	48	0.14
Cluster3179;	Proteobac	Alphap	Rhizobiales			1	2	9	5	3	3	1	3	8	8	3	2	48	0.14
Cluster3604;	Proteobac	Gamm	Xantl	Sinobac	Steroidobacter	1	1	9	6	4	2	2	1	1	12	4	5	48	0.14
Cluster4497;	Acidobact	Acidobacteria_Gp10			Gp10	2	8	4	8	1	1	2	6	4	0	3	9	48	0.14
Cluster11252	Acidobact	Acidobacteria_Gp1				1	2	16	8	1	0	0	1	1	12	5	1	48	0.14
Cluster16681	Proteobac	Gamm	Xantl	Sinobac	Steroidobacter	8	7	8	6	0	1	0	0	0	1	11	6	48	0.14
Cluster2315;	Acidobact	Acidobacteria_Gp3			Gp3	1	6	0	1	3	1	2	3	14	2	1	13	47	0.14
Cluster3983;	Proteobac	Betapr	Burkl	Burkhol	Burkholderia	4	2	8	4	4	8	5	6	5	1	0	0	47	0.14
Cluster190;si	Verrucom	Subdivision3			Subdivision3_g	7	4	0	2	5	9	6	3	0	2	5	3	46	0.13
Cluster1224;	Proteobac	Alphap	Rhizobiales			3	0	3	3	10	2	1	4	5	5	9	1	46	0.13
Cluster2435;	Proteobac	Alphap	Rhizobiales			1	9	2	1	4	3	8	8	6	2	1	1	46	0.13

Cluster3793;	Proteobac	Alphap	Alphaproteob		Rhizomicrobiu	3	2	5	1	6	6	4	2	1	5	7	4	46	0.13
Cluster353;si	Verrucom	Opitut	Opitu	Opituta	Opitutus	0	3	3	1	0	4	0	1	2	13	1	17	45	0.13
Cluster10767	Acidobacteria					3	5	0	1	9	2	1	8	6	0	2	8	45	0.13
Cluster1178;	Acidobact	Acidobacteria_Gp1			Gp1	4	4	3	2	6	1	3	6	2	9	4	0	44	0.13
Cluster13751	Acidobact	Acidobacteria_Gp10			Gp10	4	8	3	4	4	1	1	6	2	0	2	9	44	0.13
Cluster14445	Proteobac	Gamm	Xantl	Sinobac	Steroidobacter	4	17	1	3	1	1	0	1	1	1	1	13	44	0.13
Cluster371;si	Gemmatir	Gemm	Gemi	Gemma	Gemmatimona	1	2	1	2	3	7	6	4	4	10	3	0	43	0.12
Cluster7671;	Proteobac	Gammaproteobacteria				6	4	2	4	1	7	4	4	4	3	3	1	43	0.12
Cluster9864;	Acidobact	Acidobacteria_Gp2			Gp2	1	6	8	7	2	0	0	2	3	3	4	7	43	0.12
Cluster15102;size=271						9	6	4	2	3	1	3	5	2	0	3	5	43	0.12
Cluster17377	Acidobacteria					0	2	5	2	0	7	0	0	15	6	5	1	43	0.12
Cluster18205	Acidobact	Acidobacteria_Gp2			Gp2	2	2	4	0	4	4	5	5	1	5	6	5	43	0.12
Cluster18793	Acidobact	Acidobacteria_Gp1			Gp1	2	7	3	0	2	4	4	4	7	3	2	5	43	0.12
Cluster485;si	Proteobac	Alphap	Rhizo	Xanthob	Labrys	11	0	4	2	1	7	0	3	4	0	3	7	42	0.12
Cluster5780;	Verrucom	Opitut	Opitu	Opituta	Opitutus	6	2	1	0	4	6	0	0	0	17	0	6	42	0.12
Cluster1608;	Proteobac	Deltaproteobacteria				1	1	2	6	0	5	5	5	6	1	7	2	41	0.12
Cluster1736;	Proteobac	Gammaproteobacteria				1	1	5	5	3	6	2	3	0	9	4	2	41	0.12
Cluster1970;	Proteobac	Alphap	Rhizobiales			3	7	6	0	1	2	4	1	4	2	5	6	41	0.12
Cluster14310	Acidobact	Acidobacteria_Gp1				1	9	4	2	2	5	2	4	4	4	0	4	41	0.12
Cluster18117	Proteobac	Betapr	Burkl	Burkhol	Burkholderia	4	4	6	7	4	1	4	0	0	3	3	5	41	0.12
Cluster2233;	Proteobacteria					3	13	1	1	1	4	5	4	3	0	0	5	40	0.12
Cluster2235;	Proteobac	Alphap	Caulo	Cauloba	Caulobacter	1	2	3	3	3	5	2	1	2	5	9	4	40	0.12
Cluster3766;	Acidobact	Acidobacteria_Gp2			Gp2	1	6	3	0	0	11	6	0	0	0	4	9	40	0.12
Cluster25806	Actinobac	Actinol	Actinomycetales			0	4	1	1	1	10	5	5	4	2	4	3	40	0.12
Cluster1253;	Acidobact	Acidobacteria_Gp2			Gp2	7	1	5	2	5	3	0	4	4	2	4	2	39	0.11
Cluster3610;	Acidobact	Acidobacteria_Gp2			Gp2	1	4	1	1	5	2	4	4	0	0	2	15	39	0.11
Cluster4408;	Acidobact	Acidobacteria_Gp1				1	1	8	10	2	0	1	7	8	1	0	0	39	0.11
Cluster12555	Acidobact	Acidobacteria_Gp2			Gp2	1	8	0	0	4	1	1	3	3	0	1	17	39	0.11
Cluster24741	Proteobac	Alphap	Caulo	Caulobacteraceae		3	1	3	5	6	2	1	2	5	8	3	0	39	0.11

Cluster512;si	Proteobac	Alphap	Rhod	Acetobacteraceae		5	5	3	6	2	1	1	0	1	5	5	4	38	0.11
Cluster1431;s	Acidobact	Acidobacteria_Gp1				7	0	4	3	2	3	0	5	0	13	1	0	38	0.11
Cluster6725;s	Acidobact	Acidobacteria_Gp3				2	6	1	4	3	1	3	3	3	5	6	1	38	0.11
Cluster9399;s	Acidobact	Acidobacteria_Gp1			Gp1	2	7	1	1	1	7	4	2	5	1	0	7	38	0.11
Cluster11510	Proteobac	Alphap	Rhizo	Xanthol	Pseudolabrys	5	6	2	0	3	1	1	2	3	7	4	4	38	0.11
Cluster13130	Bacteroid	Sphing	Sphir	Chitinophagaceae		2	2	0	4	6	3	7	2	4	1	4	3	38	0.11
Cluster1795;s	Proteobac	Gammaproteobacteria				6	6	0	0	1	9	0	1	2	1	3	8	37	0.11
Cluster2326;size=202						2	6	4	10	0	2	1	4	1	0	4	3	37	0.11
Cluster2835;s	Proteobac	Alphap	Rhod	Acetoba	Acidisphaera	4	0	6	4	2	3	3	0	2	7	6	0	37	0.11
Cluster3222;size=176						2	3	3	2	5	1	1	2	5	6	4	3	37	0.11
Cluster3542;s	Proteobac	Gammaproteobacteria				5	0	2	4	5	2	1	4	3	4	3	4	37	0.11
Cluster4371;s	Acidobact	Acidobacteria_Gp1				3	1	3	1	2	3	1	7	7	1	3	5	37	0.11
Cluster11246	Proteobac	Alphap	Rhizobiales			3	5	0	0	2	4	1	4	9	0	4	5	37	0.11
Cluster18996	Acidobact	Acidobacteria_Gp2			Gp2	1	8	0	3	4	5	4	4	3	0	2	3	37	0.11
Cluster64;size	Proteobac	Alphap	Rhizo	Beijerin	Methylocapsa	4	4	5	0	2	1	2	6	2	5	4	1	36	0.10
Cluster1655;s	Acidobact	Acidobacteria_Gp2			Gp2	9	8	1	1	4	3	2	1	1	1	3	2	36	0.10
Cluster3855;s	Acidobact	Acidobacteria_Gp1				3	2	1	3	2	6	3	1	10	1	2	2	36	0.10
Cluster4077;s	Proteobac	Alphap	Rhodospirillales			0	8	4	4	1	6	2	1	4	1	2	3	36	0.10
Cluster7282;s	Proteobac	Alphap	Rhizo	Bradyrh	Bradyrhizobiur	0	1	0	0	3	5	4	10	8	1	4	0	36	0.10
Cluster13828	Acidobact	Acidobacteria_Gp1			Gp1	4	2	5	4	2	0	1	2	2	10	3	1	36	0.10
Cluster14736	Acidobact	Acidobacteria_Gp2			Gp2	0	3	0	0	1	3	0	6	11	2	9	1	36	0.10
Cluster906;si	Proteobac	Betapr	Burkl	Burkhol	Burkholderia	2	3	6	3	4	5	1	4	5	0	0	2	35	0.10
Cluster3785;s	Acidobact	Acidobacteria_Gp2			Gp2	1	5	1	1	1	4	3	8	5	0	3	3	35	0.10
Cluster5621;s	Acidobact	Acidobacteria_Gp2			Gp2	4	3	0	1	1	5	5	3	4	1	6	2	35	0.10
Cluster16787	Proteobac	Gammaproteobacteria				7	5	2	4	0	1	0	1	1	0	2	12	35	0.10
Cluster22368	Actinobac	Actinol	Actin	Mycoba	Mycobacteriur	1	3	2	1	3	2	6	4	3	6	1	3	35	0.10
Cluster7;size	Acidobact	Acidobacteria_Gp2			Gp2	0	3	7	5	4	0	3	2	1	2	3	4	34	0.10
Cluster1343;s	Proteobac	Alphap	Rhodospirillales			1	10	0	0	2	1	3	10	4	0	1	2	34	0.10
Cluster2251;s	Acidobact	Acidobacteria_Gp2			Gp2	6	2	4	4	2	1	2	2	5	0	3	3	34	0.10

Cluster3297;size=58						23	0	0	0	5	0	0	3	2	1	0	0	34	0.10
Cluster4648;	Acidobact	Acidobacteria_Gp1			Gp1	3	5	0	0	0	4	0	7	9	0	0	6	34	0.10
Cluster4968;	Proteobac	Alphap	Rhizc	Xanthol	Pseudolabrys	1	6	1	2	4	1	2	4	4	0	6	3	34	0.10
Cluster273;si	Acidobacteria					3	4	3	3	1	4	3	2	3	2	1	4	33	0.10
Cluster717;si	Acidobact	Acidobacteria_Gp3			Gp3	0	2	3	1	2	1	2	2	1	12	7	0	33	0.10
Cluster1697;size=179						6	2	1	3	2	1	2	2	5	5	3	1	33	0.10
Cluster2076;	Proteobac	Alphap	Alphaproteol		Rhizomicrobiu	3	2	1	3	4	4	4	5	3	2	0	2	33	0.10
Cluster3341;	Proteobac	Alphap	Rhizc	Xanthol	Pseudolabrys	3	0	4	4	1	2	5	1	5	1	6	1	33	0.10
Cluster3889;	Proteobac	Alphap	Rhodospirillales			1	5	1	3	0	8	1	6	6	0	1	1	33	0.10
Cluster43;siz	Proteobac	Alphap	Rhizobiales			2	5	1	2	2	5	8	0	1	3	2	1	32	0.09
Cluster192;si	Acidobact	Acidobacteria_Gp10			Gp10	4	8	1	4	1	0	0	4	4	0	3	3	32	0.09
Cluster4374;	Acidobact	Acidobacteria_Gp1				0	2	0	5	1	5	2	6	8	0	3	0	32	0.09
Cluster5708;size=243						3	5	3	3	1	2	7	2	2	0	2	2	32	0.09
Cluster1418(	Acidobact	Acidobacteria_Gp1			Gp1	4	2	0	0	3	2	10	3	2	1	2	3	32	0.09
Cluster68;siz	Proteobac	Betaproteobacteria				2	5	0	4	3	4	5	1	3	0	2	2	31	0.09
Cluster292;si	Proteobac	Alphap	Rhod	Rhodospirillaceae		6	5	3	2	2	2	3	0	0	1	3	4	31	0.09
Cluster674;si	Actinobac	Actinol	Actinomycetales			1	2	1	2	2	6	3	4	4	3	2	1	31	0.09
Cluster723;si	Proteobac	Alphap	Rhod	Rhodospirillaceae		2	7	0	1	3	2	1	2	4	0	0	9	31	0.09
Cluster1018;	Verrucom	Spartobacteria			Spartobacteria	2	3	1	4	1	1	0	2	7	0	2	8	31	0.09
Cluster4489;	Proteobac	Alphap	Rhodospirillales			2	2	1	1	2	5	2	5	7	2	0	2	31	0.09
Cluster14716	Acidobact	Acidobacteria_Gp3				2	7	1	4	2	1	4	4	1	1	3	1	31	0.09
Cluster16702	Acidobact	Acidobacteria_Gp1			Granulicella	0	0	5	4	2	5	0	0	5	7	3	0	31	0.09
Cluster38;size=266						2	1	2	4	0	4	4	1	5	0	4	3	30	0.09
Cluster741;si	Proteobac	Alphap	Rhod	Acetobacteraceae		1	0	3	1	0	0	1	0	1	13	10	0	30	0.09
Cluster3931;	Proteobac	Alphap	Rhodospirillales			1	4	3	2	1	8	2	2	4	1	2	0	30	0.09
Cluster12534	Acidobact	Acidobacteria_Gp3			Gp3	0	9	0	1	0	4	0	2	5	0	1	8	30	0.09
Cluster17212	Verrucom	Opitut	Opitu	Opituta	Opitutus	2	0	0	0	0	25	0	0	1	2	0	0	30	0.09
Cluster18572	Proteobac	Gamm	Xantl	Sinobac	Steroidobacte	1	6	2	2	2	2	0	2	4	3	2	4	30	0.09
Cluster569;si	Acidobact	Acidobacteria_Gp1			Gp1	4	0	1	2	6	2	2	1	3	7	1	0	29	0.08

Cluster596;si	Armatimc	Armati	Arma	Armatir	Armatimonas/	4	0	3	0	0	2	2	9	6	1	0	2	29	0.08
Cluster716;si	Gemmatii	Gemm	Gemi	Gemma	Gemmatimona	0	12	0	1	2	3	2	1	2	0	2	4	29	0.08
Cluster1612;:	Proteobac	Deltap	Myxc	Polyangiaceae		2	2	4	1	3	3	1	1	1	0	8	3	29	0.08
Cluster4595;:	Proteobac	Alphap	Rhodospirillales			1	5	0	0	3	2	1	3	8	0	0	6	29	0.08
Cluster5284;:	Proteobac	Alphap	Rhizobiales			1	3	2	0	3	3	5	1	7	0	2	2	29	0.08
Cluster10788	Proteobac	Alphap	Rhizc	Bradyrh	Bradyrhizobiur	3	1	0	3	3	1	3	0	7	5	0	3	29	0.08
Cluster348;si	Acidobact	Acidobacteria_Gp3			Gp3	3	1	3	2	1	3	2	3	3	5	1	1	28	0.08
Cluster963;si	Actinobac	Actinol	Actin	Actinos	Actinospica	3	1	3	0	2	1	2	4	2	5	3	2	28	0.08
Cluster2236;:	Acidobact	Acidobacteria_Gp2			Gp2	0	3	2	2	6	3	1	2	5	0	4	0	28	0.08
Cluster2887;:	Acidobact	Acidobacteria_Gp1			Gp1	1	2	3	1	0	10	7	1	1	0	1	1	28	0.08
Cluster13361	Acidobact	Acidobacteria_Gp1			Gp1	0	3	1	5	0	0	0	1	3	8	3	4	28	0.08
Cluster2814C	Bacteroid	Sphing	Sphir	Sphingc	Mucilaginibact	3	2	2	0	0	8	5	6	2	0	0	0	28	0.08
Cluster161;si	Verrucom	Spartobacteria			Spartobacteria	3	4	0	1	7	0	3	4	2	0	0	3	27	0.08
Cluster1386;:	Chloroflex	Ktedor	Ktedonobacterales			1	3	1	2	0	6	3	5	5	0	1	0	27	0.08
Cluster2179;:	Firmicutes					2	2	3	4	2	4	4	2	0	1	3	0	27	0.08
Cluster2828;:	Acidobact	Acidobacteria_Gp2			Gp2	5	6	0	2	1	0	4	1	2	0	1	5	27	0.08
Cluster3829;:	Acidobact	Acidobacteria_Gp1			Gp1	2	0	2	2	2	8	5	2	2	1	1	0	27	0.08
Cluster5652;:	Acidobact	Acidobacteria_Gp2			Gp2	3	1	2	4	1	1	1	2	1	0	6	5	27	0.08
Cluster5731;:	Acidobact	Acidobacteria_Gp2			Gp2	0	0	4	2	3	1	1	3	2	1	10	0	27	0.08
Cluster13474	Acidobact	Acidobacteria_Gp3			Gp3	1	0	2	1	0	8	8	2	3	2	0	0	27	0.08
Cluster14203	Acidobact	Acidobacteria_Gp1			Gp1	3	9	1	0	0	0	1	0	0	0	0	13	27	0.08
Cluster14439	Acidobact	Acidobacteria_Gp1				1	4	2	5	5	0	1	1	3	3	1	1	27	0.08
Cluster14637	Proteobac	Alphap	Alphaproteok		Rhizomicrobiu	1	0	3	0	0	1	3	5	3	8	2	1	27	0.08
Cluster1506C	Acidobact	Acidobacteria_Gp3				0	5	0	0	3	3	3	2	8	0	1	2	27	0.08
Cluster18909	Proteobac	Betapr	Burkl	Burkhol	Burkholderia	9	0	3	0	1	5	1	3	3	1	1	0	27	0.08
Cluster26051	Proteobac	Alphap	Caulc	Cauloba	Phenylobacter	3	0	1	1	4	3	3	1	0	7	4	0	27	0.08
Cluster6;size	Acidobact	Acidobacteria_Gp2			Gp2	5	0	2	2	3	3	1	1	4	4	1	0	26	0.08
Cluster2057;:	Proteobac	Gamm	Xantl	Sinobac	Steroidobacter	2	2	4	4	0	2	1	0	0	8	1	2	26	0.08
Cluster5803;:	Proteobac	Deltap	Myxococcales			5	1	1	1	5	6	1	2	0	2	2	0	26	0.08

Cluster7153;:	Proteobac	Betapr	Burkl	Burkhol	Burkholderia	5	1	2	2	1	3	0	8	2	1	0	1	26	0.08
Cluster25876	Bacteroid	Sphing	Sphir	Chitinophagaceae		3	1	2	1	0	6	1	4	0	2	2	4	26	0.08
Cluster929;si	Gemmatir	Gemm	Gemı	Gemma	Gemmatimona	0	6	0	0	0	0	2	1	4	0	0	12	25	0.07
Cluster1152;:	Acidobact	Acidobacteria_Gp2			Gp2	2	7	1	3	2	1	2	3	3	0	1	0	25	0.07
Cluster2735;:	Proteobac	Alphap	Rhizobiales			1	5	2	1	2	1	2	3	2	0	1	5	25	0.07
Cluster6130;:	Verrucom	Subdivision3			Subdivision3_g	1	1	0	1	7	1	3	1	0	1	5	4	25	0.07
Cluster8297;:	Actinobac	Actinol	Actin	Mycoba	Mycobacteriur	2	2	2	2	1	3	2	1	0	1	8	1	25	0.07
Cluster23562	Proteobac	Gamm	Xantl	Sinobac	Steroidobacter	0	0	0	0	1	2	0	0	2	20	0	0	25	0.07
Cluster24294	Proteobac	Alphap	Caulc	Caulobacteraceae		5	1	2	1	2	3	4	1	0	4	1	1	25	0.07
Cluster433;si	Proteobac	Alphap	Rhodospirillales			1	4	0	1	1	1	2	5	3	1	1	4	24	0.07
Cluster1411;:	Acidobact	Acidobacteria_Gp3			Gp3	4	0	0	5	1	3	2	3	4	1	0	1	24	0.07
Cluster1489;:	Verrucom	Opitutae				3	2	0	1	1	1	0	0	0	15	0	1	24	0.07
Cluster1853;size=156						2	1	4	4	1	3	2	0	2	3	2	0	24	0.07
Cluster2385;:	Proteobac	Betapr	Burkl	Burkhol	Burkholderia	2	2	1	0	0	3	4	3	3	0	0	6	24	0.07
Cluster2941;:	Verrucom	Spartobacteria			Spartobacteria	0	1	0	2	1	2	16	0	1	0	1	0	24	0.07
Cluster3520;:	Bacteroid	Sphing	Sphir	Chitinophagaceae		4	1	0	0	3	2	0	5	1	0	1	7	24	0.07
Cluster4273;:	Actinobac	Actinol	Actin	Mycoba	Mycobacteriur	2	1	3	2	2	0	3	3	0	2	4	2	24	0.07
Cluster4570;:	Proteobac	Alphap	Rhod	Rhodospirillaceae		1	2	0	2	1	1	2	1	6	0	4	4	24	0.07
Cluster4600;size=54						9	1	0	0	8	0	0	2	4	0	0	0	24	0.07
Cluster7609;:	Proteobac	Alphap	Rhizc	Hyphomicrobiaceae		1	1	1	4	4	1	2	3	2	2	1	2	24	0.07
Cluster8525;:	Acidobact	Acidobacteria_Gp2			Gp2	0	2	5	5	1	0	0	0	2	2	5	2	24	0.07
Cluster11371	Gemmatir	Gemm	Gemı	Gemma	Gemmatimona	3	1	1	1	3	7	1	0	1	4	2	0	24	0.07
Cluster13363	Acidobact	Acidobacteria_Gp1			Gp1	0	0	0	0	1	7	4	3	1	0	3	5	24	0.07
Cluster15086;size=164						1	3	4	1	1	2	0	0	4	1	1	6	24	0.07
Cluster10;siz	Acidobact	Acidobacteria_Gp2			Gp2	1	2	0	1	1	2	5	0	0	4	5	2	23	0.07
Cluster91;siz	Acidobact	Acidobacteria_Gp1				2	1	6	2	1	2	0	0	2	3	2	2	23	0.07
Cluster136;si	Proteobac	Deltap	Myxc	Polyangiaceae		2	2	1	0	2	4	1	0	5	3	3	0	23	0.07
Cluster269;si	Acidobact	Acidobacteria_Gp1			Gp1	1	10	0	0	1	0	1	2	6	0	1	1	23	0.07
Cluster1359;:	Acidobacteria					0	2	1	1	2	2	2	4	3	4	2	0	23	0.07

Cluster1676;:	Bacteroid	Sphing	Sphir	Chitinophagaceae		4	1	1	1	2	4	1	4	1	1	0	3	23	0.07
Cluster1869;:	Nitrospira	Nitrosp	Nitro	Nitrospi	Nitrospira	0	11	2	1	0	0	2	1	1	0	1	4	23	0.07
Cluster2869;:	Actinobac	Actinol	Acidi	Acidimi	Aciditerrimona	0	2	5	0	1	3	1	1	4	4	2	0	23	0.07
Cluster4082;:	Bacteroid	Sphing	Sphir	Chitinophagaceae		2	0	2	1	1	4	0	2	3	4	4	0	23	0.07
Cluster4282;:	Proteobac	Gammaproteobacteria				5	3	6	0	1	1	0	0	3	3	0	1	23	0.07
Cluster5539;:	Proteobac	Alphaproteobacteria				0	5	1	2	1	2	0	5	4	1	0	2	23	0.07
Cluster7724;:	Verrucom	Subdivision3			Subdivision3_g	2	0	2	2	0	3	2	2	2	0	3	5	23	0.07
Cluster8005;:	Acidobact	Acidobacteria_Gp1				5	2	6	1	4	0	0	1	0	2	1	1	23	0.07
Cluster259;si	Proteobac	Betaproteobacteria				0	2	3	2	2	3	0	2	5	0	2	1	22	0.06
Cluster343;si	Bacteroid	Sphing	Sphir	Chitinop	Flavisolibacter	3	5	1	1	1	5	3	0	2	0	1	0	22	0.06
Cluster547;si	Acidobact	Acidobacteria_Gp3			Gp3	0	1	2	4	0	1	2	0	1	3	5	3	22	0.06
Cluster871;si	Verrucom	Opitut	Opitu	Opituta	Opitutus	1	0	1	0	1	5	0	3	5	3	1	2	22	0.06
Cluster996;si	Acidobact	Acidobacteria_Gp1				1	2	0	0	0	2	3	3	3	0	1	7	22	0.06
Cluster2572;:	Acidobact	Acidobacteria_Gp3			Gp3	1	1	0	2	0	4	6	4	2	0	1	1	22	0.06
Cluster4510;:	Verrucom	Subdivision3			Subdivision3_g	0	1	0	0	1	2	3	2	7	0	2	4	22	0.06
Cluster12413	Acidobact	Acidobacteria_Gp1				4	1	2	1	0	0	2	4	1	4	2	1	22	0.06
Cluster14698	Acidobact	Acidobacteria_Gp1			Gp1	1	1	1	1	3	3	3	1	1	6	1	0	22	0.06
Cluster14701	Acidobact	Acidobacteria_Gp1				1	3	3	2	3	2	1	1	0	0	2	4	22	0.06
Cluster16636	Acidobact	Acidobacteria_Gp1			Gp1	1	0	0	5	4	4	0	0	2	5	1	0	22	0.06
Cluster18200	Acidobact	Acidobacteria_Gp3			Gp3	1	3	0	2	2	5	1	0	3	4	0	1	22	0.06
Cluster22274	Acidobact	Acidobacteria_Gp1			Gp1	3	0	2	0	4	3	3	0	1	2	1	3	22	0.06
Cluster124;size=128						2	1	0	1	5	1	5	2	1	2	1	0	21	0.06
Cluster276;si	Proteobac	Alphap	Rhizc	Bradyrhizobiaceae		0	6	1	2	0	1	0	0	2	4	2	3	21	0.06
Cluster848;si	Acidobact	Acidobacteria_Gp3				3	1	6	2	2	2	1	0	3	1	0	0	21	0.06
Cluster1787;:	Verrucom	Opitut	Opitu	Opituta	Opitutus	4	0	1	0	0	4	0	0	0	12	0	0	21	0.06
Cluster2017;:	Verrucom	Opitutae				0	5	0	1	0	0	0	0	0	9	3	3	21	0.06
Cluster2376;:	Proteobac	Gamm	Xantl	Xanthor	Rhodanobacte	2	0	1	1	4	2	6	1	2	2	0	0	21	0.06
Cluster2836;:	Verrucom	Subdivision3			Subdivision3_g	0	1	3	0	2	1	4	4	5	0	0	1	21	0.06
Cluster3195;:	Proteobac	Alphap	Alphaproteol		Rhizomicrobiu	1	1	2	1	2	2	1	1	2	7	1	0	21	0.06

Cluster3237;	Proteobac	Alphap	Rhodospirillales			1	1	2	2	5	0	1	0	2	5	2	0	21	0.06
Cluster3676;	Bacteroid	Sphing	Sphir	Chitinophagaceae		1	3	2	0	3	2	1	3	1	4	0	1	21	0.06
Cluster3797;	Proteobac	Alphap	Rhizobiales			0	1	3	2	0	3	0	0	1	2	4	5	21	0.06
Cluster4610;	Proteobac	Alphap	Rhodospirillales			4	3	0	0	2	1	0	4	4	0	1	2	21	0.06
Cluster4659;	Proteobac	Gammaproteobacteria				1	5	0	0	0	1	0	1	2	0	5	6	21	0.06
Cluster6798;	Proteobac	Deltap	Myxococcales			1	2	1	0	3	3	1	2	4	1	0	3	21	0.06
Cluster10901	Proteobac	Alphaproteobacteria				3	4	0	0	0	2	3	3	0	0	1	5	21	0.06
Cluster13304	Acidobact	Acidobacteria_Gp1				0	2	4	1	5	0	1	0	0	7	1	0	21	0.06
Cluster14009	Acidobact	Acidobacteria_Gp1			Edaphobacter	5	3	1	6	0	0	0	0	0	1	3	2	21	0.06
Cluster18715	Acidobact	Acidobacteria_Gp3			Gp3	3	0	2	2	4	0	0	0	3	6	1	0	21	0.06
Cluster27003	Bacteroid	Sphing	Sphir	Chitinophagaceae		1	0	4	1	1	1	0	0	0	7	5	1	21	0.06
Cluster15;siz	Proteobac	Alphap	Rhizc	Bradyrh	Bradyrhizobiur	3	6	0	1	0	0	0	1	1	3	3	2	20	0.06
Cluster84;siz	Proteobac	Alphap	Rhizc	Hyphomicrobiaceae		4	2	1	0	3	0	0	2	3	5	0	0	20	0.06
Cluster427;si	Verrucom	Opitut	Opitu	Opituta	Opitutus	2	1	1	2	0	7	0	0	0	6	0	1	20	0.06
Cluster1794;	Bacteroid	Sphing	Sphir	Chitinophagaceae		2	1	2	1	2	0	0	0	1	7	3	1	20	0.06
Cluster2186;	Actinobac	Actinobacteria				2	0	4	2	0	1	0	2	3	3	1	2	20	0.06
Cluster2300;	Bacteroid	Sphing	Sphir	Chitinophagaceae		2	1	2	0	5	1	1	1	0	4	3	0	20	0.06
Cluster3281;	Acidobact	Acidobacteria_Gp1			Edaphobacter	2	0	3	1	0	2	1	2	0	4	3	2	20	0.06
Cluster4895;	Bacteroid	Sphing	Sphir	Sphingc	Mucilaginibact	0	2	2	0	0	2	4	2	3	1	2	2	20	0.06
Cluster5661;	Proteobac	Alphaproteobacteria				0	2	1	3	0	4	1	0	7	0	2	0	20	0.06
Cluster9859;	Proteobac	Alphap	Rhizc	Bradyrh	Bradyrhizobiur	1	1	0	7	0	3	8	0	0	0	0	0	20	0.06
Cluster13870	Acidobact	Acidobacteria_Gp1			Gp1	0	1	2	2	2	0	2	0	2	7	1	1	20	0.06
Cluster15555	Acidobact	Acidobacteria_Gp1				0	0	1	1	3	2	0	2	6	2	2	1	20	0.06
Cluster18407	Proteobac	Alphap	Rhod	Acetobacteraceae		2	0	1	5	1	6	0	1	1	0	2	1	20	0.06
Cluster5;size	Acidobact	Acidobacteria_Gp2			Gp2	0	9	0	0	1	0	0	1	2	1	0	5	19	0.06
Cluster85;siz	Proteobac	Gamm	Xanthomonadales			2	1	1	0	0	0	0	0	0	14	0	1	19	0.06
Cluster263;si	Acidobact	Acidobacteria_Gp3			Gp3	0	14	0	0	1	0	0	0	0	0	1	3	19	0.06
Cluster417;si	Proteobac	Alphap	Rhodospirillales			0	0	4	3	2	2	1	5	1	0	1	0	19	0.06
Cluster435;si	Verrucom	Opitut	Opitu	Opituta	Opitutus	1	1	0	1	0	3	0	0	0	13	0	0	19	0.06

Cluster470;si	Verrucom	Opituta	Opitu	Opituta	Opitutus	2	3	1	0	1	4	0	0	0	1	0	7	19	0.06
Cluster521;si	Acidobact	Acidobacteria_Gp1				6	3	0	1	2	1	1	3	0	1	0	1	19	0.06
Cluster632;si	Acidobact	Acidobacteria_Gp2			Gp2	0	0	3	4	0	1	0	0	0	5	5	1	19	0.06
Cluster652;si	Acidobact	Acidobacteria_Gp2			Gp2	1	3	1	2	1	0	2	1	1	5	0	2	19	0.06
Cluster1543;s	Proteobac	Alphaproteobacteria				0	0	0	3	2	2	2	2	3	2	2	1	19	0.06
Cluster1576;s	Actinobac	Actinol	Actin	Actinos	Actinospica	1	1	1	2	0	1	1	2	2	2	0	6	19	0.06
Cluster1627;s	Acidobact	Acidobacteria_Gp2			Gp2	0	2	0	0	0	0	0	4	5	0	1	7	19	0.06
Cluster1784;s	Bacteroid	Sphing	Sphir	Chitinop	Flavitalea	0	0	2	1	3	3	3	1	3	2	1	0	19	0.06
Cluster2936;s	Verrucom	Subdivision3			Subdivision3_g	2	1	2	0	4	2	2	1	2	0	2	1	19	0.06
Cluster3803;s	Proteobac	Alphap	Rhizo	Xanthol	Pseudolabrys	1	0	1	2	3	0	1	2	1	1	6	1	19	0.06
Cluster9200;s	Proteobac	Alphap	Rhodospirillales			6	5	0	0	0	0	2	2	0	0	0	4	19	0.06
Cluster14177	Acidobact	Acidobacteria_Gp1			Granulicella	1	4	2	1	0	2	1	0	0	2	1	5	19	0.06
Cluster14675	Verrucom	Opituta	Opitu	Opituta	Opitutus	1	5	1	0	3	1	0	0	0	3	0	5	19	0.06
Cluster14852	Acidobact	Acidobacteria_Gp1			Gp1	0	1	3	4	1	0	0	0	0	1	6	3	19	0.06
Cluster16867	Acidobact	Acidobacteria_Gp1			Gp1	0	0	2	4	0	0	0	0	0	6	7	0	19	0.06
Cluster17007	Verrucom	Opituta	Opitu	Opituta	Opitutus	1	0	0	0	0	6	0	0	2	9	1	0	19	0.06
Cluster17347	Verrucom	Opituta	Opitu	Opituta	Opitutus	0	0	0	1	0	3	0	0	0	15	0	0	19	0.06
Cluster22248	Bacteroid	Sphing	Sphir	Chitinophagaceae		0	4	0	0	0	6	1	3	1	0	0	4	19	0.06
Cluster22809	Proteobac	Gamm	Xantl	Sinobac	Steroidobacter	0	0	5	0	0	3	1	0	0	7	3	0	19	0.06
Cluster23477	Acidobact	Acidobacteria_Gp2			Gp2	0	0	0	8	2	1	1	2	1	1	1	2	19	0.06
Cluster25863	Proteobac	Alphap	Alphaproteob		Rhizomicrobiu	0	0	0	4	1	2	0	1	0	8	3	0	19	0.06
Cluster11;siz	Acidobact	Acidobacteria_Gp3				1	2	0	0	2	4	0	0	4	0	2	3	18	0.05
Cluster198;si	Acidobact	Acidobacteria_Gp2			Gp2	2	3	1	1	0	2	0	4	0	3	1	1	18	0.05
Cluster323;si	Proteobac	Alphap	Rhizo	Rhizobia	Bauldia	2	2	0	2	3	0	1	1	2	0	1	4	18	0.05
Cluster643;si	Proteobac	Alphap	Rhod	Acetobacteraceae		1	0	1	3	0	0	0	0	0	11	2	0	18	0.05
Cluster759;si	Acidobact	Acidobacteria_Gp1			Gp1	0	4	0	1	2	0	1	4	2	1	1	2	18	0.05
Cluster916;si	Acidobact	Acidobacteria_Gp3			Gp3	3	0	2	1	2	3	0	0	4	3	0	0	18	0.05
Cluster1171;s	Proteobac	Alphap	Alphaproteob		Rhizomicrobiu	1	1	1	1	1	2	1	0	2	4	2	2	18	0.05
Cluster1517;s	Acidobact	Acidobacteria_Gp2			Gp2	5	1	1	1	0	2	0	0	3	2	2	1	18	0.05

Cluster1588;:	Bacteroid	Sphing	Sphir	Chitinophagaceae		6	0	1	0	2	1	0	4	1	2	0	1	18	0.05
Cluster1920;:	Proteobac	Alphap	Rhodospirillales			1	6	0	0	0	1	1	1	3	0	1	4	18	0.05
Cluster2151;:	Proteobac	Betaproteobacteria				0	10	0	0	0	0	1	2	1	0	0	4	18	0.05
Cluster2227;:	Proteobac	Gamm	Xantl	Xanthomonadaceae		2	2	2	0	1	1	0	0	1	8	1	0	18	0.05
Cluster2344;:	Acidobact	Acidobacteria_Gp3				3	0	1	0	1	2	0	1	1	3	6	0	18	0.05
Cluster2806;:	Acidobact	Acidobacteria_Gp2			Gp2	1	1	2	2	0	1	2	4	0	0	5	0	18	0.05
Cluster3815;:	Acidobact	Acidobacteria_Gp2			Gp2	1	1	0	1	2	2	4	2	0	3	0	2	18	0.05
Cluster4314;:	Proteobac	Alphap	Rhizc	Bradyrh	Bradyrhizobiur	0	2	0	0	3	2	0	5	3	0	2	1	18	0.05
Cluster6334;:	Acidobact	Acidobacteria_Gp3			Gp3	1	2	2	0	0	5	4	0	3	0	0	1	18	0.05
Cluster6688;:	Acidobact	Acidobacteria_Gp3				0	2	0	0	1	2	2	1	9	0	0	1	18	0.05
Cluster8202;:	Proteobac	Alphap	Rhod	Rhodospirillaceae		2	1	5	1	5	1	0	1	2	0	0	0	18	0.05
Cluster8535;:	Verrucom	Spartobacteria			Spartobacteria	0	0	1	0	1	1	3	5	1	0	1	5	18	0.05
Cluster9406;size=110						1	3	1	1	0	2	3	1	1	0	3	2	18	0.05
Cluster13214	Acidobact	Acidobacteria_Gp1			Gp1	0	1	3	6	0	1	1	2	2	0	1	1	18	0.05
Cluster24641	Actinobac	Actinol	Actinomycetales			1	1	1	0	2	2	3	1	3	3	1	0	18	0.05
Cluster27132	Bacteroid	Sphing	Sphir	Chitinophagaceae		3	0	2	0	1	3	2	1	0	1	3	2	18	0.05
Cluster393;size=141						0	2	0	0	1	0	2	0	6	0	2	4	17	0.05
Cluster472;size=58						2	0	2	1	2	2	1	2	4	1	0	0	17	0.05
Cluster630;si	Proteobac	Gammaproteobacteria				5	0	2	2	1	0	1	0	2	1	2	1	17	0.05
Cluster655;si	Armatimonadetes				Armatimonade	1	6	0	0	0	1	0	4	2	0	1	2	17	0.05
Cluster760;si	Acidobact	Acidobacteria_Gp3			Bryobacter	0	1	1	0	2	0	0	3	2	0	1	7	17	0.05
Cluster818;si	Proteobac	Alphap	Caulc	Cauloba	Caulobacter	3	0	1	2	3	1	2	0	3	1	1	0	17	0.05
Cluster1164;:	Proteobac	Alphap	Caulc	Caulobacteraceae		5	0	1	0	3	0	2	2	1	3	0	0	17	0.05
Cluster1256;:	Verrucom	Opituta	Opitu	Opituta	Opitutus	3	1	0	0	2	3	0	0	2	5	1	0	17	0.05
Cluster1408;:	Acidobact	Acidobacteria_Gp2			Gp2	2	1	1	1	1	1	4	4	0	0	1	1	17	0.05
Cluster1685;:	Actinobac	Actinol	Solirı	Conexib	Conexibacter	0	1	1	0	1	2	2	5	1	1	2	1	17	0.05
Cluster2144;:	Acidobact	Acidobacteria_Gp1				0	1	6	0	1	1	1	1	1	1	2	2	17	0.05
Cluster2670;:	Bacteroid	Sphing	Sphir	Chitinophagaceae		0	1	0	0	3	5	2	2	0	2	1	1	17	0.05
Cluster3418;:	Acidobact	Acidobacteria_Gp1			Gp1	0	1	3	3	0	2	1	2	1	3	1	0	17	0.05

Cluster3980;size=120						0	2	1	1	1	3	0	4	2	0	2	1	17	0.05
Cluster5824;:	Proteobac	Gammaproteobacteria				0	0	1	0	2	0	1	0	0	13	0	0	17	0.05
Cluster6628;:	Bacteroid	Sphing	Sphir	Sphingo	Mucilaginibact	0	0	5	3	1	0	2	1	1	4	0	0	17	0.05
Cluster9546;:	Acidobact	Acidobacteria_Gp2			Gp2	2	0	2	2	1	2	0	2	2	0	2	2	17	0.05
Cluster11875	Acidobact	Acidobacteria_Gp1			Edaphobacter	5	1	0	3	0	0	0	1	1	0	3	3	17	0.05
Cluster11883	Verrucom	Opituta	Opitu	Opituta	Opitutus	1	0	0	0	3	2	1	1	6	2	0	1	17	0.05
Cluster13889	Acidobact	Acidobacteria_Gp1				2	1	2	0	3	0	1	2	3	0	2	1	17	0.05
Cluster15315	Acidobact	Acidobacteria_Gp10			Gp10	0	1	0	0	3	1	3	4	2	0	2	1	17	0.05
Cluster16158	Acidobact	Acidobacteria_Gp2			Gp2	0	0	1	0	0	1	0	4	4	1	2	4	17	0.05
Cluster24539	Bacteroid	Sphing	Sphir	Chitinophagaceae		3	0	1	2	1	4	2	1	0	1	0	2	17	0.05
Cluster26492	Bacteroid	Sphing	Sphir	Chitinophagaceae		2	1	1	2	2	0	1	1	1	6	0	0	17	0.05
Cluster128;si	Proteobac	Alphap	Rhodospirillales			2	2	1	0	3	0	0	1	2	0	2	3	16	0.05
Cluster473;si	Proteobac	Deltaproteobacteria				1	3	1	3	1	1	0	1	0	1	4	0	16	0.05
Cluster515;si	Verrucom	Subdivision3			Subdivision3_g	0	0	2	2	5	1	0	0	1	2	3	0	16	0.05
Cluster678;si	Verrucom	Opituta	Opitu	Opituta	Opitutus	0	3	0	0	0	5	0	0	1	6	0	1	16	0.05
Cluster1010;:	Acidobact	Acidobacteria_Gp1			Gp1	0	1	1	0	2	3	2	1	1	1	2	2	16	0.05
Cluster1553;:	Proteobac	Betapr	Burkl	Burkholderiaceae		1	0	1	1	0	2	2	3	2	1	1	2	16	0.05
Cluster1876;:	Acidobact	Acidobacteria_Gp2			Gp2	1	0	2	4	0	0	4	0	1	0	1	3	16	0.05
Cluster2064;:	Proteobac	Alphap	Caulc	Caulobа	Caulobacter	1	1	2	0	0	0	1	1	2	4	2	2	16	0.05
Cluster2293;:	Verrucom	Opituta	Opitu	Opituta	Opitutus	3	0	2	1	1	1	0	0	3	0	0	5	16	0.05
Cluster2909;size=100						1	2	0	0	0	10	3	0	0	0	0	0	16	0.05
Cluster2928;:	Proteobac	Alphap	Alphaproteob		Rhizomicrobiu	0	1	0	0	2	2	4	0	5	0	1	1	16	0.05
Cluster3107;size=120						0	0	0	3	0	2	4	0	3	0	0	4	16	0.05
Cluster3250;size=47						1	2	0	2	1	3	0	3	2	1	1	0	16	0.05
Cluster3796;:	Acidobact	Acidobacteria_Gp1				0	0	0	1	3	1	2	4	0	0	2	3	16	0.05
Cluster5984;:	Verrucom	Opituta	Opitu	Opituta	Opitutus	4	0	1	3	1	5	1	0	0	0	0	1	16	0.05
Cluster6524;size=81						2	1	1	2	0	4	1	1	3	0	0	1	16	0.05
Cluster8219;size=99						1	0	0	1	0	2	2	2	5	0	3	0	16	0.05
Cluster8675;:	Acidobact	Acidobacteria_Gp2			Gp2	2	0	0	0	1	1	0	1	0	2	6	3	16	0.05

Cluster9083;	Acidobact	Acidobacteria_Gp2			Gp2	2	0	1	1	1	3	1	4	1	2	0	0	16	0.05
Cluster9570;	Proteobac	Gamm	Xantl	Sinobacteraceae		1	4	0	1	0	0	1	0	1	0	4	4	16	0.05
Cluster13037	Acidobact	Acidobacteria_Gp3			Gp3	0	3	0	1	2	1	0	3	1	2	2	1	16	0.05
Cluster16981	Acidobact	Acidobacteria_Gp1			Granulicella	0	0	2	0	0	4	0	0	1	7	2	0	16	0.05
Cluster23514	Proteobac	Gammaproteobacteria				0	0	0	0	0	7	1	6	2	0	0	0	16	0.05
Cluster24068	Bacteroid	Sphing	Sphir	Sphingc	Mucilaginibact	1	0	1	1	2	2	2	0	1	3	3	0	16	0.05
Cluster2;size	Proteobac	Alphap	Rhizobiales			3	3	0	0	0	2	0	4	0	0	1	2	15	0.04
Cluster4;size	Acidobact	Acidobacteria_Gp3			Gp3	2	2	0	1	0	1	0	3	5	0	1	0	15	0.04
Cluster184;si	Acidobact	Acidobacteria_Gp3			Gp3	2	5	0	1	1	2	0	0	3	0	1	0	15	0.04
Cluster622;si	Acidobact	Acidobacteria_Gp1			Edaphobacter	0	1	1	0	0	1	0	0	1	10	1	0	15	0.04
Cluster623;si	Acidobact	Acidobacteria_Gp1				1	1	1	4	2	0	0	1	1	2	2	0	15	0.04
Cluster1116;	Proteobac	Alphaproteobacteria				1	2	1	1	0	0	5	0	1	0	1	3	15	0.04
Cluster1247;	Acidobact	Acidobacteria_Gp5			Gp5	0	3	0	0	0	2	2	3	1	0	2	2	15	0.04
Cluster1446;	Acidobact	Acidobacteria_Gp2			Gp2	2	0	1	3	1	1	5	1	0	0	0	1	15	0.04
Cluster1639;	Bacteroid	Sphing	Sphir	Chitinophagaceae		1	1	1	0	0	0	0	4	2	5	1	0	15	0.04
Cluster2008;	Actinobac	Actinol	Actin	Catenul	Catenulispora	0	2	0	0	0	2	1	1	1	2	1	5	15	0.04
Cluster2050;	Acidobact	Acidobacteria_Gp3			Gp3	1	0	1	1	0	3	3	0	5	1	0	0	15	0.04
Cluster2125;	TM7				TM7_genera_i	2	0	0	0	1	3	0	6	2	0	1	0	15	0.04
Cluster2129;	Verrucom	Opituta	Opitu	Opituta	Opitutus	1	0	0	0	1	12	1	0	0	0	0	0	15	0.04
Cluster2268;	Actinobac	Actinol	Actin	Mycobacteriaceae		1	2	0	1	1	2	0	2	1	2	2	1	15	0.04
Cluster3123;	Bacteroid	Sphing	Sphir	Sphingc	Mucilaginibact	2	1	0	0	0	4	0	2	0	4	1	1	15	0.04
Cluster3256;	Proteobac	Alphap	Rhodospirillales			1	1	1	2	3	2	1	1	0	0	1	2	15	0.04
Cluster3425;	Proteobac	Alphap	Rhod	Rhodospirillaceae		0	1	2	3	0	0	0	0	0	6	2	1	15	0.04
Cluster5393;size=67						2	1	0	0	0	0	0	1	7	1	0	3	15	0.04
Cluster6289;	Proteobac	Alphap	Rhod	Acetobacteraceae		4	0	2	1	3	0	0	1	2	1	1	0	15	0.04
Cluster7137;	Acidobact	Acidobacteria_Gp2			Gp2	2	3	0	2	0	2	1	2	0	0	0	3	15	0.04
Cluster7921;	Acidobact	Acidobacteria_Gp2			Gp2	0	0	2	3	0	0	2	1	1	2	4	0	15	0.04
Cluster9680;	Proteobac	Alphaproteobacteria				0	1	1	4	0	0	1	3	5	0	0	0	15	0.04
Cluster10937	Acidobact	Acidobacteria_Gp3				2	0	0	0	3	4	3	0	1	1	1	0	15	0.04

Cluster12202	Proteobac	Betaproteobacteria				1	0	2	4	0	3	2	1	0	0	0	2	15	0.04
Cluster14732	Proteobac	Betapr	Burkl	Oxalobacteraceae		0	1	5	2	0	0	0	2	0	1	3	1	15	0.04
Cluster16489;size=72						0	4	1	0	0	1	1	1	1	0	1	5	15	0.04
Cluster24312	Bacteroid	Sphing	Sphir	Chitinophagaceae		1	1	0	1	1	0	1	1	1	4	1	3	15	0.04
Cluster25436	Proteobac	Alphap	Alphaproteob		Rhizomicrobiu	2	0	3	1	0	2	1	0	1	4	0	1	15	0.04
Cluster25477	Proteobac	Alphap	Alphaproteob		Rhizomicrobiu	3	2	1	0	0	3	0	1	2	1	2	0	15	0.04
Cluster42;siz	Proteobac	Deltap	Myxococcales			1	2	1	0	0	2	7	1	0	0	0	0	14	0.04
Cluster60;siz	Acidobact	Acidobacteria_Gp2			Gp2	1	3	1	3	1	2	0	1	0	0	1	1	14	0.04
Cluster129;si	Acidobact	Acidobacteria_Gp3			Gp3	0	2	2	1	0	1	2	1	2	0	2	1	14	0.04
Cluster615;si	Acidobact	Acidobacteria_Gp2			Gp2	0	0	0	2	0	3	2	0	1	1	5	0	14	0.04
Cluster662;si	Proteobac	Alphap	Alphaproteob		Rhizomicrobiu	7	2	1	0	1	0	2	0	1	0	0	0	14	0.04
Cluster729;si	Acidobact	Acidobacteria_Gp1				3	0	1	2	0	0	1	3	1	0	3	0	14	0.04
Cluster771;si	Proteobac	Alphap	Caulo	Caulobacteraceae		2	0	2	1	2	0	0	0	1	5	0	1	14	0.04
Cluster872;si	Proteobac	Alphap	Rhod	Acetobacteraceae		0	0	0	0	3	2	0	0	0	3	4	2	14	0.04
Cluster1050;s	Proteobac	Gamm	Xanth	Xanthomonadaceae		2	0	0	0	1	3	0	5	2	0	0	1	14	0.04
Cluster1264;size=72						0	3	0	0	1	1	0	1	2	0	2	4	14	0.04
Cluster1290;s	Actinobac	Actinol	Actinomycetales			1	0	1	0	3	0	2	3	2	1	1	0	14	0.04
Cluster1394;s	Acidobact	Acidobacteria_Gp1				0	0	5	0	2	1	1	0	2	1	1	1	14	0.04
Cluster1395;s	Proteobac	Alphap	Rhizobiales			0	1	0	1	3	2	4	1	1	1	0	0	14	0.04
Cluster1690;s	Verrucom	Opituta	Opitu	Opituta	Opitutus	3	5	0	1	0	1	0	0	0	0	1	3	14	0.04
Cluster2014;s	Proteobac	Deltap	Myxc	Polyangiaceae		0	0	1	2	0	1	0	1	2	2	3	2	14	0.04
Cluster2285;s	Proteobac	Alphap	Rhod	Rhodospirillaceae		1	0	1	0	0	0	0	8	1	3	0	0	14	0.04
Cluster2332;s	Actinobac	Actinol	Soliru	Conexib	Conexibacter	1	0	2	1	2	2	1	0	0	2	2	1	14	0.04
Cluster2630;s	Actinobac	Actinol	Actinomycetales			0	0	4	0	2	0	0	0	0	5	3	0	14	0.04
Cluster3093;s	Proteobac	Alphap	Rhizo	Bradyrh	Bradyrhizobiur	2	1	1	1	1	2	2	0	1	1	0	2	14	0.04
Cluster3432;s	Proteobac	Alphap	Caulo	Cauloba	Phenylobacter	1	0	1	0	6	1	1	1	1	1	0	1	14	0.04
Cluster4367;s	Verrucom	Opituta	Opitu	Opituta	Opitutus	7	0	0	0	2	4	1	0	0	0	0	0	14	0.04
Cluster4439;s	Proteobac	Alphap	Rhodospirillales			0	0	0	0	0	0	1	9	4	0	0	0	14	0.04
Cluster4447;s	Proteobac	Alphap	Rhizobiales			2	5	0	2	0	2	0	0	0	0	0	3	14	0.04

Cluster6273;	Acidobact	Acidobacteria_Gp1			Gp1	0	0	3	0	1	0	0	2	4	0	2	2	14	0.04
Cluster8016;	Acidobact	Acidobacteria_Gp3				1	0	0	0	0	1	1	1	5	2	3	0	14	0.04
Cluster8902;	Proteobac	Alphap	Rhod	Rhodospirillaceae		1	0	0	0	0	6	4	1	1	0	0	1	14	0.04
Cluster8939;	Acidobact	Acidobacteria_Gp1			Gp1	1	3	3	0	1	0	2	0	0	1	1	2	14	0.04
Cluster9893;	Acidobact	Acidobacteria_Gp1			Gp1	1	0	1	1	0	4	3	3	1	0	0	0	14	0.04
Cluster10530	Acidobact	Acidobacteria_Gp2			Gp2	0	0	2	1	1	3	2	1	1	0	3	0	14	0.04
Cluster11528	Proteobac	Alphap	Rhizobiales			1	6	1	1	0	1	0	0	2	1	0	1	14	0.04
Cluster11911	Acidobact	Acidobacteria_Gp1				1	1	1	0	1	0	1	2	2	0	0	5	14	0.04
Cluster14863	Gemmatir	Gemm	Gemr	Gemma	Gemmatimona	3	0	0	2	5	0	2	0	0	1	1	0	14	0.04
Cluster15746	Verrucom	Opituta	Opitu	Opituta	Opitutus	0	3	1	1	1	1	0	0	1	2	0	4	14	0.04
Cluster17189	Acidobact	Acidobacteria_Gp10			Gp10	0	2	2	2	0	3	0	1	3	0	1	0	14	0.04
Cluster18237	Acidobact	Acidobacteria_Gp2			Gp2	0	0	0	4	1	0	1	4	3	0	1	0	14	0.04
Cluster22477	Acidobact	Acidobacteria_Gp6			Gp6	1	3	0	1	1	2	1	0	1	1	1	2	14	0.04
Cluster23323	Proteobac	Gammaproteobacteria				0	0	0	0	2	3	3	5	1	0	0	0	14	0.04
Cluster26908	Bacteroid	Sphing	Sphir	Chitinophagaceae		3	0	1	0	0	1	0	7	1	0	1	0	14	0.04
Cluster29065	Bacteroidetes					4	0	1	0	1	3	0	1	0	2	2	0	14	0.04
Cluster24;siz	Acidobact	Acidobacteria_Gp2			Gp2	3	4	1	0	1	0	2	1	0	0	1	0	13	0.04
Cluster108;si	Acidobact	Acidobacteria_Gp1				4	1	1	0	0	1	0	0	0	6	0	0	13	0.04
Cluster109;si	Proteobac	Alphap	Rhod	Rhodospirillaceae		0	4	0	0	0	0	2	2	3	0	1	1	13	0.04
Cluster132;si	Acidobact	Acidobacteria_Gp3				2	0	0	0	1	2	2	1	1	2	0	2	13	0.04
Cluster310;size=68						3	2	1	1	0	1	0	1	1	2	1	0	13	0.04
Cluster332;si	Proteobac	Deltap	Myxc	Polyangiaceae		0	1	1	1	3	0	1	1	0	2	2	1	13	0.04
Cluster334;si	Actinobac	Actinol	Actinomycetales			1	2	2	0	1	0	0	0	2	2	2	1	13	0.04
Cluster430;si	Acidobact	Acidobacteria_Gp2			Gp2	2	3	0	0	1	3	1	0	1	0	1	1	13	0.04
Cluster474;size=39						0	2	2	1	0	1	0	1	1	2	2	1	13	0.04
Cluster647;si	Verrucom	Opituta	Opitu	Opituta	Opitutus	3	1	0	0	0	0	1	0	0	7	0	1	13	0.04
Cluster743;si	Acidobact	Acidobacteria_Gp1			Acidobacteriur	3	0	2	0	1	1	2	1	0	2	1	0	13	0.04
Cluster926;si	Proteobac	Alphap	Rhodospirillales			2	0	0	0	0	0	2	0	0	5	4	0	13	0.04
Cluster1015;	Acidobact	Acidobacteria_Gp3			Gp3	2	1	0	2	1	2	1	0	0	2	2	0	13	0.04

Cluster1117;s	Proteobac	Gamm	Xantl	Sinobac	Steroidobacter	1	2	0	0	2	1	0	1	0	6	0	0	13	0.04
Cluster1966;s	Bacteroid	Sphing	Sphir	Sphingo	Mucilaginibact	2	2	2	1	1	0	1	2	0	0	2	0	13	0.04
Cluster2106;s	Actinobac	Actinobacteria				1	4	0	0	3	0	0	1	1	1	0	2	13	0.04
Cluster2485;s	Proteobac	Alphap	Rhod	Acetobacteraceae		0	0	1	0	7	0	0	0	0	4	0	1	13	0.04
Cluster3209;s	Actinobac	Actinol	Solirubrobacterales			1	2	1	0	0	1	1	0	0	1	5	1	13	0.04
Cluster4605;s	Proteobac	Alphap	Rhod	Acetobacteraceae		0	2	1	0	0	1	0	0	0	4	4	1	13	0.04
Cluster4671;s	Proteobac	Alphaproteobacteria				1	0	0	0	1	2	4	2	2	0	1	0	13	0.04
Cluster5013;s	Acidobact	Acidobacteria_Gp3			Gp3	0	3	0	0	0	3	1	1	1	0	2	2	13	0.04
Cluster7181;s	Acidobact	Acidobacteria_Gp1			Gp1	0	0	0	0	0	0	0	7	6	0	0	0	13	0.04
Cluster8481;s	Verrucom	Opituta	Opitu	Opituta	Opitutus	4	0	1	0	0	1	0	0	0	5	2	0	13	0.04
Cluster8883;s	Proteobac	Alphap	Rhodospirillales			1	4	0	0	0	0	2	3	0	0	0	3	13	0.04
Cluster11417	Acidobact	Acidobacteria_Gp1			Edaphobacter	1	0	1	2	2	2	1	1	0	2	1	0	13	0.04
Cluster11840;size=57						2	1	1	3	0	2	1	1	0	0	1	1	13	0.04
Cluster14832	Acidobact	Acidobacteria_Gp1			Gp1	0	2	0	4	0	0	1	2	0	0	1	3	13	0.04
Cluster17238	Proteobac	Deltaproteobacteria				0	0	2	3	2	2	0	1	0	1	1	1	13	0.04
Cluster18753	Acidobact	Acidobacteria_Gp2			Gp2	0	2	1	0	1	1	1	0	3	0	1	3	13	0.04
Cluster19216	Acidobact	Acidobacteria_Gp2			Gp2	0	8	0	0	3	0	0	1	0	0	0	1	13	0.04
Cluster20393	Proteobac	Gamm	Xantl	Xanthor	Rhodanobacte	6	1	0	0	1	1	4	0	0	0	0	0	13	0.04
Cluster24902	Proteobac	Alphap	Caulc	Caulobacteraceae		3	0	1	0	2	1	1	1	0	2	2	0	13	0.04
Cluster24940	Actinobac	Actinol	Acidi	Acidimi	Aciditerrimona	0	2	1	0	0	3	1	0	4	1	0	1	13	0.04
Cluster191;si	Proteobac	Gamm	Xantl	Sinobac	Steroidobacter	2	0	1	0	0	1	0	1	2	4	1	0	12	0.03
Cluster494;si	Acidobact	Acidobacteria_Gp1			Gp1	2	4	0	0	1	0	0	1	4	0	0	0	12	0.03
Cluster669;si	Proteobac	Alphap	Rhod	Acetoba	Acidisphaera	1	0	1	0	2	0	0	0	1	4	3	0	12	0.03
Cluster690;si	Proteobac	Gammaproteobacteria				3	2	0	0	3	0	0	0	2	1	1	0	12	0.03
Cluster817;si	Acidobact	Acidobacteria_Gp2			Gp2	1	2	0	2	1	0	0	1	1	2	1	1	12	0.03
Cluster938;si	Proteobacteria					6	0	0	0	5	0	0	0	1	0	0	0	12	0.03
Cluster998;si	Acidobact	Acidobacteria_Gp2			Gp2	2	3	1	3	1	0	0	2	0	0	0	0	12	0.03
Cluster1129;size=60						1	0	4	2	0	2	1	1	0	0	0	1	12	0.03
Cluster1217;s	Proteobac	Alphaproteobacteria				0	1	0	1	3	1	1	1	2	2	0	0	12	0.03

Cluster1478;	Actinobac	Actinobacteria				0	0	1	1	0	1	0	0	1	4	3	1	12	0.03
Cluster1721;	Proteobac	Gammaproteobacteria				0	0	1	1	0	1	2	0	3	2	0	2	12	0.03
Cluster1968;	Acidobact	Acidobacteria_Gp1				1	1	1	1	1	1	0	0	3	1	1	1	12	0.03
Cluster2178;	Acidobact	Acidobacteria_Gp1				0	2	0	0	1	2	3	1	1	1	0	1	12	0.03
Cluster2194;	Acidobact	Acidobacteria_Gp13			Gp13	1	3	0	0	2	1	0	1	0	0	1	3	12	0.03
Cluster2207;	Proteobac	Alphaproteobacteria				0	5	0	0	0	0	0	1	3	0	0	3	12	0.03
Cluster2392;	Proteobac	Alphap	Rhod	Acetobacteraceae		0	0	4	2	0	0	0	0	0	5	1	0	12	0.03
Cluster2441;	Acidobact	Acidobacteria_Gp3			Gp3	1	1	3	0	1	1	0	0	0	3	2	0	12	0.03
Cluster2816;	Acidobact	Acidobacteria_Gp3			Gp3	2	1	1	0	1	0	0	1	3	0	0	3	12	0.03
Cluster2827;	Actinobac	Actinol	Acidimicrobiales			1	3	0	1	1	1	0	1	1	0	1	2	12	0.03
Cluster2913;	Bacteroid	Sphing	Sphir	Sphingc	Mucilaginibact	0	0	0	0	0	2	3	4	3	0	0	0	12	0.03
Cluster3025;	Proteobac	Gammaproteobacteria				0	0	0	1	2	5	2	0	1	0	0	1	12	0.03
Cluster3069;	Acidobact	Acidobacteria_Gp2			Gp2	3	0	1	1	1	0	1	1	2	1	0	1	12	0.03
Cluster3814;	Proteobac	Alphap	Rhizc	Beijerinckiaceae		0	1	2	0	0	2	0	0	2	0	1	4	12	0.03
Cluster3979;	Acidobact	Acidobacteria_Gp3				0	0	0	2	3	3	0	1	0	2	1	0	12	0.03
Cluster4031;	Proteobac	Deltaproteobacteria				0	3	0	1	0	0	2	0	4	0	2	0	12	0.03
Cluster4368;	Proteobac	Betapr	Burkl	Burkhol	Burkholderia	3	0	3	4	0	0	0	0	1	0	1	0	12	0.03
Cluster5186;	Chlorobi					1	1	3	2	0	1	2	0	1	1	0	0	12	0.03
Cluster5487;	Proteobac	Alphap	Rhodospirillales			0	1	0	1	1	0	1	1	3	0	3	1	12	0.03
Cluster5549;	Verrucom	Spartobacteria			Spartobacteria	0	1	0	0	0	2	6	1	0	0	1	1	12	0.03
Cluster5617;	Proteobac	Alphap	Rhizobiales			1	0	0	0	0	0	2	1	0	4	4	0	12	0.03
Cluster6246;	Proteobac	Alphap	Rhizc	Hyphon	Rhodoplanes	1	0	6	1	0	0	0	0	0	2	2	0	12	0.03
Cluster6335;	Proteobac	Alphaproteobacteria				1	2	0	2	0	0	1	3	1	0	2	0	12	
Cluster6446;	Actinobac	Actinol	Actinomycetales			0	1	0	0	0	1	3	3	3	0	0	1	12	
Cluster7624;	Proteobac	Gammaproteobacteria				2	1	1	1	1	0	0	1	2	0	2	1	12	
Cluster8278;	Acidobact	Acidobacteria_Gp1				2	0	1	1	1	2	0	1	0	4	0	0	12	
Cluster12214	Acidobact	Acidobacteria_Gp10			Gp10	1	1	0	0	1	1	0	1	1	0	2	4	12	
Cluster13866	Acidobact	Acidobacteria_Gp1			Gp1	1	0	1	0	3	0	2	1	0	0	0	4	12	
Cluster15243	Acidobact	Acidobacteria_Gp1			Gp1	0	0	9	3	0	0	0	0	0	0	0	0	12	

Cluster18758	Acidobact	Acidobacteria_Gp2			Gp2	1	1	1	3	2	2	0	1	0	0	1	0	12
Cluster22039	Proteobac	Alphap	Rhod	Acetoba	Acidisphaera	1	0	3	0	0	2	2	0	0	2	2	0	12
Cluster23040	Proteobac	Gammaproteobacteria				0	0	2	0	0	0	1	1	2	0	0	6	12
Cluster27818	Bacteroid	Sphing	Sphir	Chitinophagaceae		0	5	1	0	0	4	0	0	1	0	0	1	12
Cluster28173	Acidobact	Acidobacteria_Gp2			Gp2	0	0	1	0	0	1	2	4	4	0	0	0	12
Cluster31;size	Chlamydia	Chlamy	Chlar	Parachlamydiaceae		0	0	0	0	0	0	0	6	4	0	1	0	11
Cluster127;si	Acidobact	Acidobacteria_Gp3			Gp3	0	0	1	2	1	0	0	0	0	6	1	0	11
Cluster137;si	Acidobact	Acidobacteria_Gp2			Gp2	1	0	1	0	0	0	2	1	1	3	1	1	11
Cluster166;si	Acidobact	Acidobacteria_Gp1				0	2	0	0	0	0	3	2	2	0	1	1	11
Cluster223;size=117						2	0	1	1	0	1	2	1	1	0	1	1	11
Cluster284;size=30						2	0	2	2	1	0	0	2	1	1	0	0	11
Cluster373;si	Firmicutes					0	4	0	0	0	0	1	0	2	0	2	2	11
Cluster601;si	Proteobac	Gammaproteobacteria				1	0	0	0	1	3	2	1	0	0	0	3	11
Cluster659;si	Proteobac	Deltap	Myxococcales			2	0	0	0	5	1	2	0	0	1	0	0	11
Cluster786;size=52						2	2	1	2	1	1	0	0	0	0	0	2	11
Cluster846;size=77						0	0	2	4	0	0	0	0	0	4	1	0	11
Cluster877;si	Proteobac	Deltap	Myxococcales			1	1	3	1	0	0	0	1	2	0	0	2	11
Cluster923;si	Proteobac	Alphap	Rhizc	Bradyrh	Bradyrhizobiur	0	0	0	2	1	2	0	1	1	1	2	1	11
Cluster967;si	Proteobac	Alphap	Rhizobiales			0	1	1	0	1	0	2	2	2	0	0	2	11
Cluster994;si	Acidobact	Acidobacteria_Gp1			Edaphobacter	5	0	0	0	1	1	1	0	0	3	0	0	11
Cluster1069;	Proteobac	Alphap	Rhod	Rhodospirillaceae		0	0	4	0	2	1	0	1	2	0	1	0	11
Cluster1487;	Acidobact	Acidobacteria_Gp2			Gp2	2	1	0	1	1	1	1	1	0	0	2	1	11
Cluster1550;	Acidobact	Acidobacteria_Gp2			Gp2	0	0	0	0	0	0	0	3	5	0	0	3	11
Cluster1902;	Proteobac	Gamm	Xantl	Xanthomonadaceae		1	0	0	0	1	4	2	2	1	0	0	0	11
Cluster1910;	Proteobac	Alphap	Rhizc	Xanthol	Labrys	0	3	0	1	1	0	1	1	0	3	1	0	11
Cluster1954;	Acidobact	Acidobacteria_Gp2			Gp2	0	2	0	1	3	0	1	2	0	0	0	2	11
Cluster1987;	Proteobac	Alphap	Rhod	Acetobacteraceae		1	0	3	0	2	0	0	0	0	3	2	0	11
Cluster2001;	Actinobac	Actinol	Actinomycetales			0	0	0	2	1	0	0	1	2	2	3	0	11
Cluster2080;	Acidobact	Acidobacteria_Gp2			Gp2	0	0	0	0	1	0	0	0	0	7	3	0	11

Cluster2188;	Acidobact	Acidobacteria_Gp2			Gp2	4	0	1	0	0	1	0	0	3	2	0	0	11
Cluster2225;	Acidobact	Acidobacteria_Gp3				0	3	0	1	0	3	1	0	1	0	0	2	11
Cluster2258;	Acidobact	Acidobacteria_Gp3			Gp3	1	2	0	0	0	1	3	0	2	0	2	0	11
Cluster2484;	Bacteroid	Sphing	Sphir	Chitinophagaceae		0	0	1	1	1	0	0	0	0	6	2	0	11
Cluster2655;	Acidobact	Acidobacteria_Gp6			Gp6	1	1	0	1	1	1	1	1	0	0	1	3	11
Cluster2715;	Proteobac	Alphap	Rhizobiales			1	5	0	0	0	3	0	0	2	0	0	0	11
Cluster2791;	Acidobact	Acidobacteria_Gp1				2	2	1	0	2	0	2	0	0	1	1	0	11
Cluster2889;	Planctom	Plancto	Planc	Planctomycetaceae		0	0	1	2	0	0	4	1	0	0	2	1	11
Cluster2903;	Actinobac	Actinol	Acidimicrobiales			0	4	0	1	0	1	0	0	2	0	1	2	11
Cluster3021;size=54						1	2	1	0	0	0	1	0	2	1	2	1	11
Cluster3129;	Acidobact	Acidobacteria_Gp2			Gp2	0	3	0	1	0	0	2	1	1	0	1	2	11
Cluster3403;	Bacteroid	Sphing	Sphir	Chitinophagaceae		0	0	2	1	0	2	2	1	1	2	0	0	11
Cluster4065;size=40						0	1	0	0	2	1	0	0	3	2	0	2	11
Cluster4224;	Proteobac	Alphap	Rhodospirillales			4	2	1	1	0	0	0	1	0	0	1	1	11
Cluster7614;	Acidobact	Acidobacteria_Gp1			Edaphobacter	0	1	0	2	0	0	0	1	3	0	4	0	11
Cluster1002	Acidobact	Acidobacteria_Gp1			Gp1	0	0	1	2	0	0	0	3	4	0	0	1	11
Cluster1024	Actinobac	Actinol	Solirubrobacterales			2	1	3	0	0	1	1	0	0	1	2	0	11
Cluster1341	Proteobac	Alphap	Rhizc	Bradyrh	Bradyrhizobiur	2	3	0	0	1	0	1	2	0	0	0	2	11
Cluster1357	Proteobac	Betapr	Burkl	Burkhol	Burkholderia	1	0	1	0	0	2	1	1	1	1	1	2	11
Cluster1405	Acidobact	Acidobacteria_Gp1			Gp1	0	2	1	0	0	1	1	2	3	0	1	0	11
Cluster1559	Proteobac	Betapr	Burkholderiales			0	1	0	0	0	2	1	4	0	2	1	0	11
Cluster1606	Acidobact	Acidobacteria_Gp1			Gp1	0	0	1	1	1	1	0	1	2	2	1	1	11
Cluster1643	Acidobact	Acidobacteria_Gp1			Acidobacteriur	0	0	4	0	2	0	1	0	0	3	1	0	11
Cluster17536;size=74						0	0	2	2	0	3	0	0	0	0	1	3	11
Cluster1813	Acidobact	Acidobacteria_Gp2			Gp2	2	0	0	0	0	0	0	0	1	4	4	0	11
Cluster20335;size=63						2	1	0	1	0	1	3	0	0	0	1	2	11
Cluster2252	Proteobac	Alphap	Rhod	Acetoba	Acidisphaera	1	0	2	0	0	3	0	0	0	3	2	0	11
Cluster2546	Proteobac	Alphap	Caulc	Cauloba	Caulobacter	4	0	1	1	0	1	1	0	0	0	3	0	11
Cluster46;siz	Gemmatir	Gemm	Gem	Gemma	Gemmatimona	0	2	2	1	0	1	0	0	0	1	3	0	10

Cluster62;siz	Proteobac	Betapr	Burkl	Burkhol	Burkholderia	4	1	0	0	1	1	0	2	0	1	0	0	10
Cluster141;si	Proteobac	Deltap	Myxo	Cystobacteraceae		1	0	2	2	0	1	1	1	1	0	0	1	10
Cluster316;si	Proteobac	Alphaproteobacteria				0	0	0	0	2	1	0	2	3	2	0	0	10
Cluster327;si	Acidobact	Acidobacteria_Gp3				1	1	1	0	1	1	0	0	0	4	1	0	10
Cluster514;si	Acidobact	Acidobacteria_Gp3			Gp3	1	0	1	3	0	0	0	1	4	0	0	0	10
Cluster552;size=47						0	1	0	0	0	1	1	1	2	0	0	4	10
Cluster633;si	Armatimonadetes				Armatimonade	0	1	2	0	0	1	2	1	1	0	1	1	10
Cluster641;si	Proteobac	Deltap	Myxo	Cystobacteraceae		2	1	1	0	1	1	1	0	2	0	0	1	10
Cluster670;si	Proteobac	Gammaproteobacteria				3	0	2	0	1	1	0	1	0	0	1	1	10
Cluster705;si	Acidobact	Acidobacteria_Gp1				3	0	1	0	1	0	0	0	1	4	0	0	10
Cluster918;si	Armatimo	Chthon	Chtho	Chthono	Chthonomona	0	1	1	1	1	0	1	0	0	3	1	1	10
Cluster1007;s	Acidobact	Acidobacteria_Gp5			Gp5	1	2	2	1	0	0	0	1	0	3	0	0	10
Cluster1208;s	Acidobact	Acidobacteria_Gp1			Gp1	0	1	0	0	3	0	1	0	1	3	1	0	10
Cluster1218;s	Proteobac	Alphap	Rhodospirillales			1	3	0	1	1	1	0	0	0	0	1	2	10
Cluster1466;s	Acidobact	Acidobacteria_Gp1			Gp1	2	0	0	0	2	3	0	1	1	0	0	1	10
Cluster1477;s	Actinobac	Actinol	Soliru	Conexib	Conexibacter	0	0	2	1	0	0	1	0	1	4	1	0	10
Cluster1547;s	Acidobact	Acidobacteria_Gp2			Gp2	1	1	2	0	0	3	1	0	0	0	0	2	10
Cluster1716;s	Bacteroid	Sphing	Sphin	Chitinophagaceae		1	1	1	0	1	2	0	0	1	1	1	1	10
Cluster1791;s	Actinobac	Actinol	Solirubrobacterales			0	1	1	0	1	0	1	1	1	1	1	2	10
Cluster1813;s	Bacteroid	Sphing	Sphin	Chitinophagaceae		0	1	0	0	1	1	1	1	0	2	3	0	10
Cluster1879;size=37						1	0	1	0	2	0	2	1	0	0	2	1	10
Cluster1896;s	Acidobact	Acidobacteria_Gp2			Gp2	0	1	1	3	1	0	1	2	1	0	0	0	10
Cluster2005;s	Acidobact	Acidobacteria_Gp3			Gp3	0	0	1	1	0	1	1	4	0	0	1	1	10
Cluster2062;s	Acidobact	Acidobacteria_Gp1				0	0	0	0	1	0	0	0	0	0	0	9	10
Cluster2090;s	Proteobac	Alphap	Rhizo	Methylocystaceae		0	0	1	2	1	0	1	1	0	3	1	0	10
Cluster2116;s	Acidobact	Acidobacteria_Gp2			Gp2	1	2	1	0	1	0	0	1	4	0	0	0	10
Cluster2172;s	Bacteroid	Sphing	Sphingobacteriales			2	0	0	0	3	1	0	0	0	2	0	2	10
Cluster2239;size=51						5	1	0	0	0	0	0	0	2	1	1	0	10
Cluster2249;s	TM7				TM7_genera_i	0	2	0	0	0	0	1	0	2	0	5	0	10

Cluster2615;	Proteobac	Alphap	Rhodospirillales			0	1	1	2	3	0	1	0	1	0	0	1	10
Cluster2689;	Acidobact	Acidobacteria_Gp1				2	0	3	0	0	1	1	0	1	1	0	1	10
Cluster2912;	Bacteroid	Sphing	Sphir	Chitinophagaceae		1	0	3	1	3	0	0	0	0	2	0	0	10
Cluster2972;	Verrucom	Opitut	Opitu	Opituta	Opitutus	0	0	0	0	0	0	0	0	1	9	0	0	10
Cluster3008;	Proteobac	Gamm	Legio	Coxiella	Aquicella	1	2	1	0	2	0	3	1	0	0	0	0	10
Cluster3282;size=61						0	2	1	0	0	2	1	3	1	0	0	0	10
Cluster3388;	Proteobac	Alphap	Rhizobiales			0	1	2	0	0	1	1	1	0	0	3	1	10
Cluster3837;	Acidobact	Acidobacteria_Gp3			Bryobacter	1	1	0	0	2	1	1	0	1	1	1	1	10
Cluster4569;size=35						3	3	0	0	1	2	0	0	0	0	1	0	10
Cluster4598;	Acidobact	Acidobacteria_Gp3			Gp3	4	0	0	0	5	1	0	0	0	0	0	0	10
Cluster4884;	Proteobac	Alphap	Rhizc	Methylocystaceae		1	1	1	0	1	1	2	1	1	1	0	0	10
Cluster5036;	Proteobac	Deltap	Myxococcales			2	1	1	0	0	1	1	0	1	1	1	1	10
Cluster5159;	Acidobact	Acidobacteria_Gp1				0	1	2	1	3	0	1	0	0	2	0	0	10
Cluster6615;	Acidobact	Acidobacteria_Gp3			Gp3	1	0	0	1	3	0	0	0	1	1	3	0	10
Cluster7107;	Acidobact	Acidobacteria_Gp1				3	0	2	0	0	1	0	1	2	1	0	0	10
Cluster7304;	Proteobac	Alphap	Rhodospirillales			0	3	0	0	0	0	1	0	3	0	2	1	10
Cluster7774;	Proteobac	Alphap	Rhizc	Bradyrh	Bradyrhizobiur	1	1	0	1	0	0	2	1	2	0	2	0	10
Cluster10675	Proteobac	Alphap	Caulc	Caulobacteraceae		1	0	4	1	0	2	1	0	0	1	0	0	10
Cluster11632	Acidobact	Acidobacteria_Gp3			Gp3	1	2	1	0	2	0	0	1	1	0	0	2	10
Cluster15735	Actinobac	Actinol	Acidimicrobiales			0	0	1	1	3	0	0	1	2	0	1	1	10
Cluster15789	Acidobact	Acidobacteria_Gp2			Gp2	0	3	0	1	1	0	1	0	0	0	2	2	10
Cluster15991	Acidobact	Acidobacteria_Gp1			Gp1	1	0	1	0	2	4	0	0	0	1	1	0	10
Cluster17034	Actinobac	Actinol	Acidi	Acidimi	Aciditerrimona	0	0	3	1	2	3	0	0	0	0	0	1	10
Cluster18192	Proteobac	Alphap	Rhod	Acetobacteraceae		0	0	0	0	1	0	0	0	0	6	3	0	10
Cluster19409	Acidobact	Acidobacteria_Gp2			Gp2	0	3	1	1	0	1	2	2	0	0	0	0	10
Cluster22029	Proteobac	Gammaproteobacteria				2	0	1	0	0	0	0	0	0	6	1	0	10
Cluster22327	Proteobac	Gamm	Xantl	Sinobac	Nevskia	2	0	0	0	1	3	0	1	0	0	0	3	10
Cluster22388	Proteobac	Gamm	Xantl	Sinobacteraceae		0	1	0	0	1	0	3	0	1	0	1	3	10
Cluster22691	Actinobac	Actinol	Acidi	Acidimi	Aciditerrimona	1	0	0	1	1	2	0	0	0	0	2	3	10

Cluster2299€	Actinobac	Actinol	Actinomycetales			2	0	1	0	1	0	2	0	2	0	1	1	10
Cluster23244	Actinobac	Actinol	Actin	Mycoba	Mycobacteriur	0	0	0	0	1	0	2	3	1	3	0	0	10
Cluster26135	Proteobac	Alphap	Caulc	Caulobа	Phenylobacter	2	0	0	0	3	1	2	0	1	0	1	0	10
Cluster28;siz	Proteobac	Betapr	Burkl	Oxalobacteraceae		1	0	0	0	0	0	2	2	0	3	1	0	9
Cluster40;siz	Proteobac	Gamm	Xantl	Xanthomonadaceae		1	0	0	0	1	3	0	0	2	2	0	0	9
Cluster221;si	Acidobact	Acidobacteria_Gp2			Gp2	4	0	1	0	1	0	0	0	0	1	2	0	9
Cluster272;size=31						0	2	0	0	0	0	1	1	0	3	0	2	9
Cluster312;si	Proteobac	Alphaproteobacteria				2	2	0	0	1	0	1	1	0	1	0	1	9
Cluster589;si	Proteobac	Alphap	Rhodospirillales			4	0	0	0	0	1	2	0	1	0	1	0	9
Cluster649;si	Proteobac	Alphap	Rhod	Acetoba	Acidocella	1	0	1	1	0	0	0	0	0	4	2	0	9
Cluster714;si	Acidobact	Acidobacteria_Gp3			Gp3	0	1	0	1	0	0	0	2	0	1	1	3	9
Cluster875;si	Acidobact	Acidobacteria_Gp1				2	0	1	0	1	0	0	1	2	0	1	1	9
Cluster934;si	Acidobact	Acidobacteria_Gp5			Gp5	0	2	1	0	1	0	2	0	2	0	0	1	9
Cluster1021;	Proteobac	Alphap	Rhodospirillales			0	2	0	0	0	1	1	3	0	0	0	2	9
Cluster1119;	Proteobac	Gamm	Pseu	Pseudoi	Pseudomonas	0	1	1	1	0	2	1	0	0	0	3	0	9
Cluster1191;	Actinobac	Actinobacteria				1	0	1	0	2	1	2	1	0	0	1	0	9
Cluster1266;	Proteobac	Alphap	Rhizobiales			0	1	0	2	1	0	1	0	1	1	1	1	9
Cluster1631;	Proteobac	Alphap	Caulc	Caulobа	Phenylobacter	3	0	0	0	1	2	0	0	0	1	2	0	9
Cluster1663;	Acidobact	Acidobacteria_Gp2			Gp2	0	0	2	3	1	0	0	2	0	0	1	0	9
Cluster1715;	Bacteroid	Sphing	Sphir	Chitinophagaceae		1	0	1	1	0	1	1	1	1	1	1	0	9
Cluster2281;	Chloroflex	Ktedor	Ktedc	Ktedonc	Ktedonobacter	0	0	0	0	6	0	0	0	1	1	1	0	9
Cluster2443;	Acidobact	Acidobacteria_Gp2			Gp2	3	0	0	2	2	0	0	1	0	0	1	0	9
Cluster2777;	Acidobact	Acidobacteria_Gp13			Gp13	1	1	0	0	1	1	1	1	1	2	0	0	9
Cluster2865;	Bacteroidetes					0	1	0	0	1	0	2	0	1	4	0	0	9
Cluster2876;	Proteobac	Betapr	Burkl	Comamonadaceae		1	0	0	0	0	3	0	3	0	0	2	0	9
Cluster2935;	Proteobac	Alphap	Rhizc	Bradyrh	Agromonas	1	0	2	0	0	0	0	1	1	2	2	0	9
Cluster3091;	Bacteroid	Sphing	Sphir	Chitinophagaceae		1	0	0	2	0	3	0	0	0	0	0	3	9
Cluster3136;	Actinobac	Actinol	Acidi	Acidimi	Aciditerrimona	1	1	1	1	2	1	0	0	1	1	0	0	9
Cluster3350;	Acidobact	Acidobacteria_Gp13			Gp13	0	2	0	1	1	1	1	1	0	0	2	0	9

Cluster3501;:	Acidobact	Acidobacteria_Gp3			Gp3	0	1	0	0	0	1	1	3	1	0	1	1	9
Cluster3726;:	Planctomy	Plancto	Planc	Plancto	Singulisphaera	0	1	2	0	3	0	0	2	0	0	1	0	9
Cluster3935;:	Proteobac	Alphap	Rhizo	Beijerinckiaceae		0	3	0	1	0	0	1	1	1	0	0	2	9
Cluster4383;size=40						1	1	0	1	1	0	1	2	1	0	1	0	9
Cluster4483;:	Acidobact	Acidobacteria_Gp1				2	0	1	0	0	0	0	1	2	0	2	1	9
Cluster4917;:	Acidobact	Acidobacteria_Gp2			Gp2	0	0	0	1	0	0	0	1	4	3	0	0	9
Cluster5404;:	Acidobact	Acidobacteria_Gp2			Gp2	0	0	1	2	0	0	2	0	1	0	1	2	9
Cluster8344;:	Bacteroid	Sphing	Sphir	Chitinophagaceae		1	0	0	0	0	0	1	1	0	4	2	0	9
Cluster8845;:	Acidobact	Acidobacteria_Gp2			Gp2	0	1	0	3	0	1	0	3	0	0	1	0	9
Cluster10345	Proteobac	Alphap	Rhizo	Xanthob	Pseudolabrys	0	0	0	1	1	0	0	1	2	0	2	2	9
Cluster11684	Acidobact	Acidobacteria_Gp3			Gp3	0	0	1	0	0	0	0	2	1	1	3	1	9
Cluster12253	Proteobac	Gamm	Xanth	Xanthor	Frateuria	0	0	2	0	3	2	0	0	0	1	1	0	9
Cluster12976	Proteobac	Gammaproteobacteria				0	2	4	0	0	1	1	0	0	0	0	1	9
Cluster14773	Proteobac	Betapr	Burkl	Oxalobacteraceae		2	0	1	0	1	0	1	1	3	0	0	0	9
Cluster14809	Acidobact	Acidobacteria_Gp2			Gp2	1	1	3	0	0	0	0	0	0	2	2	0	9
Cluster15171	Acidobact	Acidobacteria_Gp3			Gp3	1	2	0	0	0	1	0	1	4	0	0	0	9
Cluster16108	Acidobact	Acidobacteria_Gp1			Granulicella	0	0	1	0	2	1	0	0	1	4	0	0	9
Cluster16160	Acidobact	Acidobacteria_Gp1				0	0	0	2	2	0	0	0	2	1	2	0	9
Cluster16270	Acidobact	Acidobacteria_Gp2			Gp2	0	0	3	5	0	0	0	0	0	0	1	0	9
Cluster16282	Acidobact	Acidobacteria_Gp2			Gp2	1	0	0	0	0	0	0	0	3	0	3	2	9
Cluster17089	Acidobact	Acidobacteria_Gp1				0	0	0	1	0	0	0	0	2	6	0	0	9
Cluster17249	Proteobac	Alphap	Caulo	Cauloba	Phenylobacter	0	2	2	0	0	0	0	1	2	1	1	0	9
Cluster18922	Acidobact	Acidobacteria_Gp3			Gp3	0	1	0	1	1	0	0	1	1	2	1	1	9
Cluster22098	Proteobac	Gammaproteobacteria				0	0	1	0	0	1	0	4	0	1	0	2	9
Cluster22141	Proteobac	Alphap	Rhod	Acetoba	Acidisphaera	4	0	3	1	0	0	0	0	0	1	0	0	9
Cluster23397	Proteobac	Gamm	Xanth	Sinobac	Steroidobacter	1	2	1	0	0	3	0	0	1	1	0	0	9
Cluster25738	Bacteroid	Sphing	Sphir	Chitinop	Flavitalea	1	1	0	0	0	4	2	0	1	0	0	0	9
Cluster29218	Bacteroid	Sphing	Sphir	Sphingobacteriaceae		2	1	0	0	0	1	0	0	3	0	0	2	9
Cluster30;size=69						2	0	0	0	2	1	1	0	2	0	0	0	8

Cluster54;siz	Proteobac	Alphap	Rhodospirillales			0	2	0	0	2	0	0	1	3	0	0	0	8
Cluster58;siz	Acidobact	Acidobacteria_Gp2			Gp2	0	0	1	1	2	0	0	1	1	1	1	0	8
Cluster81;siz	Proteobac	Betapr	Burkl	Burkhol	Burkholderia	0	0	0	1	1	3	0	0	2	1	0	0	8
Cluster112;size=55						0	0	1	2	0	1	2	0	0	1	1	0	8
Cluster178;si	Proteobac	Deltap	Myxc	Polyangiaceae		1	0	1	1	0	1	1	1	0	1	1	0	8
Cluster302;size=62						3	1	1	0	1	1	0	0	0	1	0	0	8
Cluster387;si	Proteobac	Alphap	Rhodospirillales			2	0	1	0	0	1	0	0	1	2	1	0	8
Cluster507;size=51						0	0	0	0	0	1	0	1	5	0	0	1	8
Cluster631;si	Acidobact	Acidobacteria_Gp1				0	0	2	1	1	1	0	2	1	0	0	0	8
Cluster710;si	Acidobact	Acidobacteria_Gp2			Gp2	0	3	0	0	0	0	0	0	2	0	1	2	8
Cluster752;si	Acidobact	Acidobacteria_Gp1			Gp1	0	3	0	0	0	0	2	2	0	0	0	1	8
Cluster777;si	Proteobac	Alphap	Rhod	Acetoba	Acidisphaera	0	0	0	1	2	2	1	0	0	2	0	0	8
Cluster1161;size=35						1	0	0	0	0	4	1	0	0	1	0	1	8
Cluster1263;	Acidobacteria					2	0	0	0	1	0	0	1	1	1	2	0	8
Cluster1288;	Acidobact	Acidobacteria_Gp2			Gp2	0	0	0	0	0	1	1	1	2	1	2	0	8
Cluster1300;	Proteobac	Alphap	Rhodospirillales			0	0	0	1	1	4	0	0	1	0	0	1	8
Cluster1374;	Acidobact	Acidobacteria_Gp6			Gp6	0	2	0	0	2	0	1	1	0	1	0	1	8
Cluster1445;	Acidobact	Acidobacteria_Gp2			Gp2	1	2	0	0	1	0	1	0	0	0	1	2	8
Cluster1585;	Acidobact	Acidobacteria_Gp1			Granulicella	2	0	0	3	0	1	0	0	0	1	1	0	8
Cluster1610;	Acidobact	Acidobacteria_Gp2			Gp2	1	1	2	0	0	1	1	0	1	1	0	0	8
Cluster1687;	Acidobact	Acidobacteria_Gp3			Bryobacter	1	0	0	0	2	0	0	0	1	1	0	3	8
Cluster1698;	Bacteroid	Sphing	Sphir	Chitinophagaceae		1	2	1	0	0	1	1	0	0	0	1	1	8
Cluster1705;	Bacteroid	Sphing	Sphir	Chitinophagaceae		0	1	0	1	2	2	0	1	0	0	1	0	8
Cluster1846;	Acidobact	Acidobacteria_Gp3			Gp3	2	0	1	0	0	0	0	0	0	3	2	0	8
Cluster2108;	Acidobact	Acidobacteria_Gp2			Gp2	0	1	1	0	2	0	2	0	0	1	1	0	8
Cluster2149;	Actinobac	Actinol	Acidi	Acidimi	Aciditerrimona	1	1	2	1	1	1	0	0	0	0	1	0	8
Cluster2199;	Proteobac	Gamm	Xantl	Sinobac	Steroidobacter	0	2	2	1	0	0	0	0	0	3	0	0	8
Cluster2323;size=29						2	0	0	0	1	1	0	2	0	0	1	1	8
Cluster2346;	Acidobact	Acidobacteria_Gp1				0	1	0	1	0	0	1	1	2	1	1	0	8

Cluster2487;	Acidobact	Acidobacteria_Gp10			Gp10	1	2	1	2	1	0	0	0	1	0	0	0	8
Cluster2497;	Bacteroid	Sphing	Sphir	Chitinophagaceae		2	1	0	0	1	0	3	0	1	0	0	0	8
Cluster2668;	Proteobac	Alphap	Rhizo	Methylocystaceae		1	0	2	2	0	0	0	0	1	1	1	0	8
Cluster2775;	Actinobac	Actinol	Acidi	Acidimi	Aciditerrimona	0	0	1	0	1	0	1	0	1	3	1	0	8
Cluster3053;	Proteobac	Betapr	Burkl	Oxalobacteraceae		1	0	0	0	1	2	0	1	2	0	1	0	8
Cluster3126;	Proteobac	Betapr	Burkl	Burkhol	Burkholderia	3	0	0	1	1	1	0	0	0	2	0	0	8
Cluster3163;	Bacteroidetes					1	1	1	0	0	0	0	1	0	0	4	0	8
Cluster3166;	Proteobac	Alphap	Rhod	Acetobacteraceae		0	0	1	0	0	1	0	0	0	4	1	1	8
Cluster3367;	Acidobact	Acidobacteria_Gp6			Gp6	1	1	0	0	1	0	1	0	2	1	0	1	8
Cluster3540;	Verrucom	Subdivision3			Subdivision3_g	3	0	0	0	1	1	0	0	0	0	1	2	8
Cluster3729;	Acidobacteria					0	2	0	0	1	1	2	1	0	0	1	0	8
Cluster4446;	Proteobac	Alphap	Rhod	Rhodospirillaceae		0	0	2	2	1	1	0	1	1	0	0	0	8
Cluster4466;	Bacteroid	Sphing	Sphir	Sphingo	Mucilaginibact	1	1	1	0	0	0	0	0	1	4	0	0	8
Cluster4790;	Proteobac	Alphap	Rhodospirillales			0	1	0	0	0	2	0	2	1	0	2	0	8
Cluster5123;	Verrucom	Opitut	Opitu	Opituta	Opitutus	0	2	0	0	0	0	1	0	1	3	0	1	8
Cluster5382;	Proteobac	Alphap	Rhodospirillales			0	2	0	0	0	0	1	0	3	0	2	0	8
Cluster6402;	Proteobac	Alphap	Rhizobiales			1	1	0	2	2	1	0	0	1	0	0	0	8
Cluster6435;	Proteobac	Alphap	Rhodospirillales			1	0	0	0	0	1	2	2	1	0	0	1	8
Cluster6674;	Acidobact	Acidobacteria_Gp2			Gp2	0	0	0	0	0	0	0	1	5	0	1	1	8
Cluster7085;	Acidobact	Acidobacteria_Gp1			Gp1	0	0	4	1	0	1	1	0	0	1	0	0	8
Cluster7150;	Actinobac	Actinol	Actinomycetales			1	0	0	2	0	0	0	1	0	4	0	0	8
Cluster7224;	Acidobact	Acidobacteria_Gp1			Gp1	0	0	0	3	3	0	0	0	0	2	0	0	8
Cluster7262;	Proteobac	Deltaproteobacteria				0	1	0	1	0	1	1	0	1	0	3	0	8
Cluster7912;	Bacteroid	Sphing	Sphir	Chitinophagaceae		3	0	1	1	0	0	0	0	1	1	1	0	8
Cluster8250;size=36						1	0	0	0	2	3	2	0	0	0	0	0	8
Cluster8563;	Acidobact	Acidobacteria_Gp10			Gp10	0	1	1	0	0	0	2	0	4	0	0	0	8
Cluster9738;	Proteobac	Alphap	Caulo	Caulob	Phenylobacter	0	1	1	0	0	1	2	0	2	0	0	1	8
Cluster9951;	Proteobac	Deltap	Myxococcales			0	0	0	0	0	1	2	0	2	0	2	1	8
Cluster10084	Acidobact	Acidobacteria_Gp1				0	0	0	0	0	0	1	2	1	3	1	0	8

Cluster11410	Acidobact	Acidobacteria_Gp1			Gp1	2	0	0	0	0	1	4	1	0	0	0	0	8
Cluster12447	Acidobact	Acidobacteria_Gp3			Gp3	0	0	0	0	0	0	1	1	2	2	2	0	8
Cluster12721	Acidobacteria					0	2	0	0	0	0	2	0	4	0	0	0	8
Cluster13406	Proteobac	Alphap	Rhizobiales			0	2	1	2	1	0	0	0	0	0	0	2	8
Cluster14176	Acidobact	Acidobacteria_Gp10			Gp10	0	1	0	0	0	0	0	1	5	0	0	1	8
Cluster14515	Acidobact	Acidobacteria_Gp1				2	1	0	1	4	0	0	0	0	0	0	0	8
Cluster14608	Proteobac	Alphaproteobacteria				0	0	0	1	2	1	0	0	0	1	3	0	8
Cluster14975	Proteobac	Alphap	Rhizc	Bradyrh	Bradyrhizobiu	1	1	2	0	0	0	0	0	0	2	2	0	8
Cluster15400	Acidobact	Acidobacteria_Gp1			Edaphobacter	3	1	0	1	1	0	0	0	0	1	1	0	8
Cluster15997	Acidobact	Acidobacteria_Gp1			Gp1	0	1	0	0	0	1	0	2	1	1	2	0	8
Cluster16249	Proteobac	Alphaproteobacteria				0	0	1	0	0	0	0	0	0	5	2	0	8
Cluster16817	Acidobact	Acidobacteria_Gp1				0	0	1	0	0	0	0	0	0	3	4	0	8
Cluster17168;size=78						0	0	1	2	0	1	0	0	4	0	0	0	8
Cluster17310	Acidobact	Acidobacteria_Gp1			Gp1	1	0	1	1	0	1	0	0	0	1	3	0	8
Cluster18130	Acidobact	Acidobacteria_Gp2			Gp2	0	4	0	0	0	0	0	0	1	0	0	3	8
Cluster18586	Proteobac	Alphap	Rhizc	Methylocystaceae		0	0	1	1	2	2	1	0	1	0	0	0	8
Cluster18765	Acidobact	Acidobacteria_Gp3			Gp3	1	1	1	0	2	1	0	0	1	1	0	0	8
Cluster18863	Proteobac	Betapr	Burkl	Oxaloba	Collimonas	0	0	0	0	1	2	0	4	1	0	0	0	8
Cluster19135	Acidobact	Acidobacteria_Gp3			Gp3	0	0	1	0	0	1	2	0	0	2	2	0	8
Cluster20157	Acidobact	Acidobacteria_Gp3			Gp3	0	0	1	5	1	1	0	0	0	0	0	0	8
Cluster20224	Actinobac	Actinol	Solirı	Conexib	Conexibacter	0	1	1	0	2	0	2	1	1	0	0	0	8
Cluster22182	Acidobact	Acidobacteria_Gp1			Gp1	1	1	0	0	0	0	0	2	1	1	2	0	8
Cluster22297	Acidobact	Acidobacteria_Gp2			Gp2	1	0	3	0	0	1	1	0	1	1	0	0	8
Cluster22430	Proteobac	Gamm	Xantl	Sinobac	Steroidobacter	2	0	2	0	2	1	0	0	0	0	0	1	8
Cluster22891	Proteobac	Gamm	Xanthomonadales			0	0	0	0	1	1	0	1	0	0	5	0	8
Cluster23610	Proteobac	Alphap	Alphaproteol		Rhizomicrobiu	1	1	0	0	1	0	1	2	0	0	0	2	8
Cluster24201	Proteobac	Alphap	Alphaproteol		Rhizomicrobiu	2	0	0	0	2	2	1	1	0	0	0	0	8
Cluster24597	Chloroflex	Ktedor	Ktedonobacterales			0	0	0	1	0	0	4	1	0	0	2	0	8
Cluster27776	Bacteroid	Sphing	Sphir	Chitinophagaceae		0	1	1	0	1	4	0	0	0	0	1	0	8

Cluster26;siz	Proteobac	Alphap	Rhodospirillales			0	1	1	0	1	0	0	0	4	0	0	0	7
Cluster56;siz	Acidobact	Acidobacteria_Gp2			Gp2	1	1	0	0	2	0	2	1	0	0	0	0	7
Cluster93;siz	Verrucom	Opituta	Opitu	Opituta	Opitutus	1	1	0	0	0	3	1	1	0	0	0	0	7
Cluster156;si	Acidobact	Acidobacteria_Gp2			Gp2	0	0	3	0	2	0	0	1	1	0	0	0	7
Cluster159;size=30						1	0	1	0	0	3	1	0	0	0	0	1	7
Cluster390;si	Proteobac	Betapr	Burkholderiales			0	1	0	0	1	2	2	0	0	1	0	0	7
Cluster395;size=17						1	2	1	0	0	1	0	0	0	2	0	0	7
Cluster415;si	Acidobact	Acidobacteria_Gp3			Gp3	2	0	1	0	2	0	0	0	0	0	0	2	7
Cluster428;size=23						0	1	2	1	1	0	0	0	1	0	0	1	7
Cluster442;si	Armatimonadetes				Armatimonade	0	0	1	1	0	0	3	1	0	0	1	0	7
Cluster449;si	Proteobac	Alphap	Rhizobiales			1	0	0	0	2	1	1	1	0	1	0	0	7
Cluster518;si	Acidobact	Acidobacteria_Gp2			Gp2	4	1	0	0	0	0	0	0	0	0	0	2	7
Cluster531;si	Acidobact	Acidobacteria_Gp2			Gp2	1	0	0	4	0	1	0	0	1	0	0	0	7
Cluster607;si	Actinobac	Actinol	Solirubrobacterales			0	1	0	0	1	1	2	1	1	0	0	0	7
Cluster682;si	Gemmatir	Gemm	Gemi	Gemma	Gemmatimona	0	1	0	0	0	1	5	0	0	0	0	0	7
Cluster685;si	Acidobact	Acidobacteria_Gp3			Gp3	0	2	0	0	2	0	0	1	1	0	1	0	7
Cluster948;si	Acidobact	Acidobacteria_Gp1			Gp1	0	1	0	0	0	2	0	0	1	0	0	3	7
Cluster1077;	Acidobact	Acidobacteria_Gp1			Gp1	2	0	2	2	1	0	0	0	0	0	0	0	7
Cluster1134;	Proteobac	Alphap	Rhodospirillales			1	1	0	1	1	0	1	1	0	0	1	0	7
Cluster1138;	Acidobact	Acidobacteria_Gp3			Gp3	1	1	1	0	2	0	0	0	1	1	0	0	7
Cluster1669;	Proteobac	Alphaproteobacteria				0	0	0	1	1	2	1	0	1	0	1	0	7
Cluster1735;	Acidobact	Acidobacteria_Gp3			Gp3	0	0	1	2	0	0	1	0	0	2	1	0	7
Cluster1901;	Acidobact	Acidobacteria_Gp2			Gp2	0	0	0	1	0	0	0	1	0	4	1	0	7
Cluster2164;	Proteobac	Alphap	Rhod	Acetobacteraceae		1	0	0	0	0	0	0	0	0	3	3	0	7
Cluster2286;	Actinobac	Actinol	Actinomycetales			1	0	0	0	0	2	1	0	0	2	1	0	7
Cluster2313;	Acidobact	Acidobacteria_Gp2			Gp2	2	1	1	0	1	1	0	0	1	0	0	0	7
Cluster2316;	Proteobac	Gamm	Xantl	Sinobac	Steroidobacter	0	0	2	1	0	1	0	0	0	3	0	0	7
Cluster2470;size=57						0	0	0	0	0	2	3	2	0	0	0	0	7
Cluster2515;	Acidobact	Acidobacteria_Gp3				1	0	1	2	3	0	0	0	0	0	0	0	7

Cluster2517;	Actinobac	Actinobacteria				1	0	0	0	1	1	0	1	2	0	1	0	7
Cluster2646;	Acidobact	Acidobacteria_Gp1				0	3	0	0	0	0	0	0	0	0	1	3	7
Cluster2682;	Acidobact	Acidobacteria_Gp3			Gp3	1	1	0	0	1	1	0	0	1	0	2	0	7
Cluster2748;	Proteobac	Betapr	Burkl	Oxaloba	Oxalicibacteriu	2	0	0	0	2	1	0	1	1	0	0	0	7
Cluster2796;	Actinobac	Actinol	Acidimicrobiales			0	1	0	0	0	0	3	0	1	0	0	2	7
Cluster2846;	Proteobac	Alphap	Alphaproteol		Rhizomicrobiu	1	1	0	1	0	1	1	0	0	1	1	0	7
Cluster2915;size=11						0	0	0	0	0	1	0	0	0	1	1	4	7
Cluster2988;	Proteobac	Gamm	Xantl	Sinobac	Steroidobacter	1	0	2	2	0	0	0	0	0	0	0	2	7
Cluster3109;	Acidobact	Acidobacteria_Gp2			Gp2	2	0	0	0	2	0	0	2	0	1	0	0	7
Cluster3110;	Proteobacteria					1	0	0	1	0	1	0	0	0	3	1	0	7
Cluster3159;	Verrucom	Opituta	Opitu	Opituta	Opitutus	0	2	0	0	0	0	0	0	0	0	0	5	7
Cluster3241;	Acidobacteria					0	0	0	0	0	1	3	0	0	0	3	0	7
Cluster3353;	Verrucom	Subdivision3			Subdivision3_g	1	1	1	1	1	0	0	0	1	1	0	0	7
Cluster3413;	Acidobact	Acidobacteria_Gp2			Gp2	0	0	0	2	1	0	1	0	3	0	0	0	7
Cluster3460;	Acidobact	Acidobacteria_Gp1				0	0	1	1	0	1	1	0	0	1	2	0	7
Cluster3577;	Acidobact	Acidobacteria_Gp13			Gp13	0	2	0	0	1	0	0	0	1	0	2	1	7
Cluster3596;	Acidobact	Acidobacteria_Gp6			Gp6	0	0	0	1	2	0	2	0	0	0	1	1	7
Cluster3842;	Acidobact	Acidobacteria_Gp6			Gp6	0	0	0	1	3	1	0	1	0	0	1	0	7
Cluster4233;size=16						0	0	0	2	4	0	0	1	0	0	0	0	7
Cluster4744;	Acidobact	Acidobacteria_Gp1			Gp1	0	1	0	0	0	1	2	2	1	0	0	0	7
Cluster4980;	Proteobac	Alphap	Rhodospirillales			0	1	1	0	0	1	1	1	2	0	0	0	7
Cluster5021;	Acidobact	Acidobacteria_Gp2			Gp2	0	2	0	0	0	0	0	1	2	0	1	1	7
Cluster5064;size=18						0	4	0	0	0	0	0	0	0	0	0	3	7
Cluster5689;	Verrucom	Opitutae				1	0	0	0	3	1	1	0	0	1	0	0	7
Cluster5866;	Acidobact	Acidobacteria_Gp2			Gp2	0	0	0	0	1	0	3	2	0	0	0	1	7
Cluster6617;	Acidobact	Acidobacteria_Gp1			Edaphobacter	0	1	0	2	1	1	0	2	0	0	0	0	7
Cluster6637;	Verrucom	Subdivision3			Subdivision3_g	2	0	2	0	2	0	0	0	0	1	0	0	7
Cluster7378;	Proteobac	Alphap	Rhodospirillales			0	0	0	2	1	2	0	1	0	0	0	1	7
Cluster7785;	Proteobac	Alphap	Rhodospirillales			3	0	0	1	0	0	0	0	1	0	0	2	7

Cluster8211;	Chlamydia	Chlamy	Chlar	Parachlamydiaceae		0	0	0	0	1	0	1	0	2	0	1	2	7
Cluster8507;	Acidobact	Acidobacteria_Gp2			Gp2	0	0	0	1	0	0	2	1	0	1	1	1	7
Cluster8717;	Acidobact	Acidobacteria_Gp3			Gp3	1	0	1	1	1	1	1	0	0	1	0	0	7
Cluster8755;	Proteobac	Deltap	Myxc	Polyangiaceae		0	0	1	0	1	0	1	0	1	2	1	0	7
Cluster8801;	Acidobact	Acidobacteria_Gp2			Gp2	0	0	0	0	0	0	0	2	5	0	0	0	7
Cluster9106;	Proteobac	Alphap	Rhizc	Hyphon	Rhodoplanes	2	1	2	0	0	0	0	0	1	1	0	0	7
Cluster9153;	Proteobac	Deltap	Myxc	Polyangiaceae		2	0	0	0	0	3	0	0	0	1	1	0	7
Cluster9707;	Proteobac	Alphap	Rhizobiales			0	0	0	5	0	0	0	0	1	0	1	0	7
Cluster10820	Armatimc	Armati	Arma	Armatir	Armatimonas/	2	0	0	0	1	2	0	1	0	0	0	1	7
Cluster11070	Proteobac	Gamm	Xantl	Sinobac	Steroidobacter	1	2	0	0	0	0	0	0	1	0	0	3	7
Cluster11212	Acidobact	Acidobacteria_Gp1			Gp1	0	1	0	1	0	1	1	2	0	0	0	1	7
Cluster11599	Bacteroid	Sphing	Sphir	Chitinop	Ferruginibacte	0	2	0	0	0	1	0	1	2	0	0	1	7
Cluster13373	Verrucom	Opituta	Opitu	Opituta	Opitutus	1	1	0	0	1	1	0	0	1	1	0	1	7
Cluster13806	Proteobac	Alphap	Alphaproteob		Rhizomicrobiu	0	2	0	0	0	2	1	1	0	0	1	0	7
Cluster14458	Proteobac	Gamm	Xantl	Sinobac	Steroidobacter	0	0	1	0	0	2	1	1	0	2	0	0	7
Cluster14766	Acidobact	Acidobacteria_Gp1				2	1	0	0	0	1	0	2	1	0	0	0	7
Cluster14846	Acidobact	Acidobacteria_Gp1			Gp1	0	0	1	2	1	0	1	0	0	1	1	0	7
Cluster14909	Actinobac	Actinol	Actin	Mycoba	Mycobacterium	0	0	1	1	1	0	1	1	0	0	2	0	7
Cluster15098	Acidobact	Acidobacteria_Gp1				3	0	0	0	0	0	1	0	0	2	1	0	7
Cluster15311	Acidobact	Acidobacteria_Gp1			Gp1	1	0	0	4	0	1	0	0	1	0	0	0	7
Cluster15351	Acidobact	Acidobacteria_Gp1			Gp1	1	0	0	0	1	0	1	0	0	2	1	1	7
Cluster16682	Proteobac	Alphaproteobacteria				0	0	0	0	1	2	0	0	1	0	3	0	7
Cluster16861	Acidobact	Acidobacteria_Gp1				0	0	0	0	0	3	0	0	3	0	1	0	7
Cluster17494	Acidobact	Acidobacteria_Gp1				0	0	0	1	0	2	0	0	0	3	1	0	7
Cluster17607	Proteobac	Betapr	Burkl	Burkhol	Burkholderia	0	1	0	1	0	0	0	1	0	0	0	4	7
Cluster19153	Acidobact	Acidobacteria_Gp3			Gp3	1	0	0	0	1	3	2	0	0	0	0	0	7
Cluster19897	Proteobac	Gamm	Xantl	Sinobacteraceae		0	2	0	0	1	0	0	1	0	0	1	2	7
Cluster20495	Acidobact	Acidobacteria_Gp1				1	0	0	1	0	0	1	2	1	0	1	0	7
Cluster20586	Proteobac	Alphap	Alphaproteob		Rhizomicrobiu	0	0	0	2	0	0	1	0	1	3	0	0	7

Cluster21013	Actinobac	Actinol	Solirubrobacterales			1	0	0	0	0	0	1	1	0	1	3	0	7
Cluster21289	Chloroflex	Ktedor	Ktedonobacterales			0	2	0	0	1	0	1	0	0	0	0	3	7
Cluster21850	Actinobac	Actinol	Solirι	Conexib	Conexibacter	1	0	0	0	1	0	1	0	0	3	1	0	7
Cluster22541	Acidobact	Acidobacteria_Gp6			Gp6	1	0	0	2	0	0	2	0	0	0	1	1	7
Cluster25162	Proteobac	Alphap	Caulc	Caulobacteraceae		1	1	0	2	0	0	0	1	0	1	0	1	7
Cluster27044	Bacteroid	Sphing	Sphir	Chitinophagaceae		1	0	2	0	0	0	0	1	0	1	2	0	7
Cluster28435	Actinobac	Actinol	Actinomycetales			0	0	0	1	2	0	0	0	1	0	3	0	7
Cluster14;siz	Proteobac	Alphap	Rhodospirillales			2	2	1	0	0	0	0	0	0	0	0	1	6
Cluster16;siz	Proteobac	Alphap	Rhizobiales			1	0	0	0	1	0	1	1	0	0	0	2	6
Cluster20;siz	Acidobact	Acidobacteria_Gp2			Gp2	0	0	0	2	2	0	0	0	0	2	0	0	6
Cluster34;size=42						0	0	1	1	0	0	2	0	1	0	1	0	6
Cluster66;siz	Proteobac	Alphap	Rhizobiales			0	2	0	0	0	0	0	0	0	1	0	3	6
Cluster126;si	Verrucom	Subdivision3			Subdivision3_g	0	0	0	1	0	2	0	1	0	0	1	1	6
Cluster158;si	Acidobact	Acidobacteria_Gp1				3	0	0	0	0	2	0	0	0	1	0	0	6
Cluster180;si	Acidobact	Acidobacteria_Gp2			Gp2	2	0	1	2	0	0	0	1	0	0	0	0	6
Cluster234;si	Acidobact	Acidobacteria_Gp3			Gp3	1	0	0	0	0	2	0	0	0	1	2	0	6
Cluster247;si	Acidobact	Acidobacteria_Gp1			Gp1	0	0	0	1	2	0	0	0	1	0	0	2	6
Cluster258;si	Acidobact	Acidobacteria_Gp3			Gp3	1	0	2	0	0	1	0	0	1	1	0	0	6
Cluster306;si	Proteobac	Alphap	Rhizobiales			1	0	0	0	2	0	0	0	0	0	2	1	6
Cluster317;si	Acidobacteria					1	2	0	0	1	0	0	1	0	0	1	0	6
Cluster384;si	Verrucom	Spartobacteria			Spartobacteria	0	1	1	1	1	0	0	0	0	1	1	0	6
Cluster402;si	Acidobact	Acidobacteria_Gp1			Gp1	1	0	0	0	1	1	0	0	0	3	0	0	6
Cluster420;si	Proteobac	Betapr	Burkl	Burkhol	Burkholderia	3	1	0	0	0	1	0	0	0	0	0	1	6
Cluster451;si	Acidobact	Acidobacteria_Gp2			Gp2	2	0	0	2	1	1	0	0	0	0	0	0	6
Cluster526;si	Actinobac	Actinol	Actinomycetales			0	0	1	0	0	0	0	0	0	3	2	0	6
Cluster561;si	Acidobact	Acidobacteria_Gp1			Gp1	2	0	0	0	2	1	0	1	0	0	0	0	6
Cluster618;si	Proteobac	Gamm	Xantl	Xanthor	Rhodanobacte	1	1	0	0	1	2	1	0	0	0	0	0	6
Cluster724;si	Proteobac	Gammaproteobacteria				0	1	1	0	0	0	0	0	0	2	1	1	6
Cluster833;size=30						0	0	0	0	2	0	0	0	1	0	0	3	6

Cluster836;si	Proteobac	Betapr	Burkl	Comamonadaceae		1	2	1	0	0	0	0	0	0	0	0	2	6
Cluster876;si	Acidobact	Acidobacteria_Gp3			Gp3	0	1	0	0	1	0	1	0	2	0	0	1	6
Cluster932;si	Acidobact	Acidobacteria_Gp2			Gp2	0	3	0	0	0	2	0	1	0	0	0	0	6
Cluster939;si	Acidobact	Acidobacteria_Gp1			Gp1	1	1	1	0	0	0	0	2	0	0	1	0	6
Cluster949;si	Acidobact	Acidobacteria_Gp1			Granulicella	2	0	1	0	1	0	0	0	0	2	0	0	6
Cluster1032;:	Acidobact	Acidobacteria_Gp1				2	0	2	1	0	0	0	0	1	0	0	0	6
Cluster1113;:	Proteobac	Alphap	Rhizobiales			0	0	1	0	2	0	0	0	0	3	0	0	6
Cluster1131;size=13						0	0	2	0	0	1	0	0	0	0	1	2	6
Cluster1143;size=33						0	0	1	2	1	0	0	0	0	1	1	0	6
Cluster1160;:	Acidobact	Acidobacteria_Gp1			Gp1	1	1	0	1	0	1	1	0	0	1	0	0	6
Cluster1245;:	Acidobact	Acidobacteria_Gp1				2	0	0	0	1	0	0	0	0	2	1	0	6
Cluster1274;:	Bacteroid	Sphing	Sphir	Chitinophagaceae		2	0	0	0	0	0	1	0	0	3	0	0	6
Cluster1283;:	Acidobact	Acidobacteria_Gp1			Gp1	0	2	1	0	0	0	0	1	1	1	0	0	6
Cluster1381;:	Acidobacteria					0	1	0	0	2	0	0	2	0	1	0	0	6
Cluster1523;:	Acidobact	Acidobacteria_Gp1				1	0	0	1	1	0	2	0	0	0	1	0	6
Cluster1530;:	Chloroflex	Ktedor	Ktedo	Ktedono	Ktedonobacter	1	1	1	0	1	0	1	0	0	0	1	0	6
Cluster1531;:	Proteobac	Betapr	Burkl	Burkhol	Burkholderia	1	0	0	1	1	1	0	0	0	2	0	0	6
Cluster1552;:	Acidobact	Acidobacteria_Gp2			Gp2	0	1	0	0	0	0	0	2	1	0	1	1	6
Cluster1581;:	Acidobact	Acidobacteria_Gp1				0	0	2	1	0	0	1	0	1	0	1	0	6
Cluster1694;:	Bacteroid	Sphing	Sphir	Chitinophagaceae		1	0	0	0	1	1	0	2	1	0	0	0	6
Cluster1730;:	Acidobact	Acidobacteria_Gp3			Gp3	0	0	0	0	0	0	0	0	2	3	1	0	6
Cluster1777;:	Acidobact	Acidobacteria_Gp1			Gp1	0	0	1	0	0	0	2	0	2	0	0	1	6
Cluster1789;:	Actinobac	Actinol	Solirubrobacterales			2	1	0	1	0	0	0	1	0	0	0	1	6
Cluster1804;:	Chlamydia	Chlamy	Chlamydiales			0	0	0	0	0	0	2	2	0	1	0	1	6
Cluster1814;:	Acidobact	Acidobacteria_Gp1				0	0	0	0	2	0	1	1	0	2	0	0	6
Cluster1816;:	Proteobac	Alphap	Rhizo	Hyphon	Rhodoplanes	0	0	0	1	1	2	0	1	0	0	1	0	6
Cluster1823;:	Proteobac	Alphap	Rhizobiales			0	0	0	1	0	0	0	0	0	1	3	1	6
Cluster1824;:	Armatimonadetes				Armatimonade	1	0	1	0	1	1	0	1	0	0	0	1	6
Cluster1840;:	Proteobac	Alphap	Rhodospirillales			0	0	0	0	0	2	2	0	1	0	0	1	6

Cluster1867;size=12						0	0	1	1	0	0	1	0	0	3	0	0	6
Cluster1893;	Proteobac	Gamm	Xantl	Xanthomonadaceae		1	0	0	0	0	0	0	1	4	0	0	0	6
Cluster1988;	Proteobac	Alphap	Rhod	Acetobacteraceae		0	0	0	0	0	1	2	1	2	0	0	0	6
Cluster1993;	Proteobac	Alphap	Rhizobiales			0	1	0	0	3	0	1	0	0	1	0	0	6
Cluster2252;	Proteobac	Gammaproteobacteria				1	0	0	1	0	0	0	0	1	1	0	2	6
Cluster2277;	Acidobact	Acidobacteria_Gp1				0	1	0	0	0	1	0	0	0	0	2	2	6
Cluster2371;	Verrucom	Subdivision3			Subdivision3_g	1	0	1	0	0	0	0	0	0	2	1	1	6
Cluster2412;	Acidobacteria					1	0	0	1	1	1	1	0	1	0	0	0	6
Cluster2414;	Acidobact	Acidobacteria_Gp1				1	0	0	0	0	0	0	1	0	1	3	0	6
Cluster2508;	Acidobact	Acidobacteria_Gp2			Gp2	1	0	0	0	1	1	0	0	1	2	0	0	6
Cluster2557;	Actinobac	Actinol	Actin	Catenul	Catenulispora	0	0	0	1	1	2	0	0	0	0	0	2	6
Cluster2632;	Actinobac	Actinol	Actinomycetales			0	0	0	1	1	1	1	0	0	0	2	0	6
Cluster2642;	Proteobac	Alphaproteobacteria				1	0	1	0	1	0	0	0	0	1	1	1	6
Cluster2839;	Chlamydia	Chlamy	Chlamydiales			1	0	0	0	1	0	1	0	1	1	0	1	6
Cluster2841;	Proteobac	Alphap	Rhod	Acetobacteraceae		2	0	0	0	2	0	0	1	0	1	0	0	6
Cluster2914;	Actinobac	Actinol	Actinomycetales			0	1	1	0	0	2	0	1	0	0	1	0	6
Cluster2927;	Proteobac	Alphap	Rhodospirillales			0	0	0	1	0	1	1	0	1	0	2	0	6
Cluster3204;	Proteobac	Alphap	Alphaproteob		Rhizomicrobiu	0	0	1	1	1	0	0	0	0	2	0	1	6
Cluster3261;	Actinobac	Actinol	Acidi	Acidimi	Aciditerrimona	0	1	0	1	0	2	0	0	1	0	0	1	6
Cluster3313;	Bacteroid	Sphing	Sphir	Sphingc	Mucilaginibact	1	0	0	0	0	2	2	0	0	0	1	0	6
Cluster3741;	Proteobac	Deltap	Myxc	Polyangiaceae		0	0	0	0	1	2	0	0	0	1	1	1	6
Cluster3779;size=27						3	0	0	0	0	0	0	1	2	0	0	0	6
Cluster3946;	Acidobact	Acidobacteria_Gp7			Gp7	1	0	0	0	2	1	0	0	1	0	0	1	6
Cluster4015;	Acidobact	Acidobacteria_Gp3			Gp3	0	0	0	0	5	0	0	0	1	0	0	0	6
Cluster4042;	Acidobact	Acidobacteria_Gp1				1	0	1	1	1	0	0	0	0	1	0	1	6
Cluster4088;	Bacteroid	Sphing	Sphir	Chitinophagaceae		0	0	0	1	0	3	0	0	0	0	1	1	6
Cluster4095;	Proteobac	Alphap	Rhodospirillales			0	1	0	0	1	0	0	2	0	1	1	0	6
Cluster4116;	Acidobact	Acidobacteria_Gp2			Gp2	1	0	0	0	1	0	3	1	0	0	0	0	6
Cluster5203;	Acidobact	Acidobacteria_Gp1				0	0	0	0	0	1	0	1	3	0	1	0	6

Cluster5675;	Proteobac	Alphap	Rhizobiales			0	0	0	2	0	1	2	0	1	0	0	0	6
Cluster6207;	Verrucom	Subdivision3			Subdivision3_g	0	1	0	1	2	0	0	1	0	0	0	1	6
Cluster6370;	Acidobact	Acidobacteria_Gp10			Gp10	3	0	0	1	0	0	0	1	1	0	0	0	6
Cluster6406;	Acidobact	Acidobacteria_Gp1				0	0	1	1	0	0	2	1	1	0	0	0	6
Cluster6612;	Acidobact	Acidobacteria_Gp3			Gp3	0	1	0	0	0	0	0	1	1	0	1	2	6
Cluster6659;	Acidobact	Acidobacteria_Gp1			Edaphobacter	1	0	0	0	1	0	1	1	0	1	1	0	6
Cluster6724;	Proteobac	Deltap	Myxc	Polyangiaceae		1	1	0	0	0	1	1	2	0	0	0	0	6
Cluster6950;	Proteobac	Alphap	Rhodospirillales			0	2	0	0	1	0	0	0	0	0	0	3	6
Cluster7148;	Acidobact	Acidobacteria_Gp3			Gp3	0	0	1	0	0	0	1	0	1	2	1	0	6
Cluster8050;	Acidobact	Acidobacteria_Gp2			Gp2	0	3	0	0	0	0	1	0	0	0	0	2	6
Cluster8083;	Proteobac	Alphap	Rhodospirillales			0	0	0	0	1	0	0	0	0	0	4	1	6
Cluster8153;	Acidobact	Acidobacteria_Gp2			Gp2	1	1	0	1	1	1	0	0	1	0	0	0	6
Cluster8590;	Proteobac	Alphap	Rhizobiales			0	0	0	2	0	0	2	2	0	0	0	0	6
Cluster8595;	Proteobac	Alphap	Rhodospirillales			0	3	0	0	0	0	0	0	0	0	0	3	6
Cluster8716;	Proteobac	Alphaproteobacteria				1	1	0	0	1	0	1	1	1	0	0	0	6
Cluster9192;	Proteobac	Alphap	Alphaproteol		Rhizomicrobiu	0	1	2	1	0	0	0	0	1	1	0	0	6
Cluster9630;	Proteobac	Alphap	Rhizobiales			2	0	0	0	0	0	1	0	2	0	0	1	6
Cluster9651;	Proteobac	Alphap	Rhodospirillales			0	0	0	0	2	0	1	1	2	0	0	0	6
Cluster9745;	Proteobac	Alphap	Rhodospirillales			4	0	0	0	0	0	1	1	0	0	0	0	6
Cluster9759;	Proteobac	Alphap	Rhodospirillales			0	1	0	0	0	0	2	0	1	0	0	2	6
Cluster10407	Acidobact	Acidobacteria_Gp1				0	3	0	0	1	0	0	1	0	0	0	1	6
Cluster10438	Acidobact	Acidobacteria_Gp3			Gp3	0	1	2	1	0	0	0	0	0	1	1	0	6
Cluster10637	Acidobact	Acidobacteria_Gp1				0	2	0	2	0	0	0	1	0	0	0	1	6
Cluster11078;size=38						1	0	0	1	0	1	1	0	2	0	0	0	6
Cluster11245	Proteobac	Alphap	Rhodospirillales			0	3	0	0	0	1	0	0	0	0	1	1	6
Cluster11409	Proteobac	Betapr	Burkl	Burkhol	Burkholderia	1	0	0	0	2	1	1	0	0	1	0	0	6
Cluster11428	Proteobac	Alphap	Rhizobiales			0	0	1	0	1	1	0	0	0	2	1	0	6
Cluster11648	Proteobac	Alphap	Rhizc	Bradyrh	Bradyrhizobiur	0	0	0	0	0	1	0	0	0	2	3	0	6
Cluster11768	Proteobac	Betapr	Burkl	Burkhol	Burkholderia	1	0	2	0	2	0	0	0	0	0	0	1	6

Cluster1205	Acidobact	Acidobacteria_Gp1				0	2	1	2	0	0	0	0	0	0	1	0	6
Cluster1249	Verrucom	Opitut	Opitu	Opituta	Opitutus	0	1	0	0	0	0	1	0	1	3	0	0	6
Cluster1296	Actinobac	Actino	Soliru	Conexib	Conexibacter	0	0	1	0	0	0	0	1	1	1	0	2	6
Cluster1328	Proteobac	Alphap	Rhizobiales			0	0	1	2	0	0	0	0	0	0	1	2	6
Cluster1364	Actinobac	Actinobacteria				0	3	0	0	0	0	0	1	0	0	1	1	6
Cluster1374	Proteobac	Deltaproteobacteria				1	0	0	0	1	0	1	0	1	0	2	0	6
Cluster1386	Acidobact	Acidobacteria_Gp1			Gp1	0	0	0	0	0	2	0	1	1	0	2	0	6
Cluster1388	Acidobact	Acidobacteria_Gp1				2	0	0	0	0	0	0	0	4	0	0	0	6
Cluster1402	Acidobact	Acidobacteria_Gp1			Gp1	0	0	2	0	1	1	0	0	0	0	2	0	6
Cluster1436	Acidobact	Acidobacteria_Gp3			Gp3	0	0	1	1	0	1	0	0	0	2	1	0	6
Cluster1441	Proteobac	Betapr	Burkl	Oxalobacteraceae		2	0	2	0	0	1	0	0	0	1	0	0	6
Cluster1458	Acidobact	Acidobacteria_Gp1				0	0	1	1	0	1	0	0	0	3	0	0	6
Cluster1461	Chloroflex	Ktedonobacteria				0	0	0	0	2	0	0	3	1	0	0	0	6
Cluster1461	Acidobact	Acidobacteria_Gp1				1	0	0	0	0	2	1	1	1	0	0	0	6
Cluster1501	Acidobact	Acidobacteria_Gp3			Gp3	0	1	0	1	0	1	0	2	1	0	0	0	6
Cluster1587	Acidobact	Acidobacteria_Gp2			Gp2	0	1	0	0	0	0	1	0	1	0	0	3	6
Cluster1654	Acidobacteria					0	0	0	4	0	1	0	0	1	0	0	0	6
Cluster1655	Acidobact	Acidobacteria_Gp2			Gp2	4	0	2	0	0	0	0	0	0	0	0	0	6
Cluster1674	Acidobact	Acidobacteria_Gp1				0	0	1	3	0	0	0	0	1	1	0	0	6
Cluster1686	Proteobac	Alphap	Rhod	Acetobacteraceae		0	1	0	1	0	0	0	1	0	2	1	0	6
Cluster1695	Acidobact	Acidobacteria_Gp2			Gp2	1	0	0	0	1	0	0	0	2	1	1	0	6
Cluster1724	Acidobact	Acidobacteria_Gp1			Gp1	0	0	0	0	2	3	0	0	0	0	1	0	6
Cluster1748	Acidobact	Acidobacteria_Gp1			Gp1	0	1	0	0	2	0	0	3	0	0	0	0	6
Cluster1755	Proteobac	Alphap	Rhizobiales			0	0	1	0	0	0	0	1	0	4	0	0	6
Cluster1775	Acidobact	Acidobacteria_Gp3			Gp3	0	0	1	1	0	0	0	1	0	1	2	0	6
Cluster1780	Acidobact	Acidobacteria_Gp2			Gp2	0	0	0	0	0	0	0	1	0	0	2	3	6
Cluster1781	Acidobact	Acidobacteria_Gp2			Gp2	1	0	1	1	1	0	0	1	0	0	1	0	6
Cluster1811	Acidobact	Acidobacteria_Gp2			Gp2	0	0	0	0	4	0	0	2	0	0	0	0	6
Cluster1815	Acidobact	Acidobacteria_Gp3			Gp3	0	0	0	1	1	1	1	0	1	0	1	0	6

Cluster18537	Acidobact	Acidobacteria_Gp3			Gp3	0	1	1	0	0	1	0	0	1	0	0	2	6
Cluster18582	Proteobac	Alphap	Alphaproteob		Rhizomicrobiu	0	0	0	1	0	1	0	1	1	2	0	0	6
Cluster18611	Actinobac	Actinobacteria				3	0	0	0	2	0	1	0	0	0	0	0	6
Cluster18948	Actinobac	Actinol	Acidi	Acidimi	Aciditerrimona	0	0	0	1	1	0	0	1	1	2	0	0	6
Cluster19477	Acidobact	Acidobacteria_Gp2			Gp2	0	2	0	0	1	0	3	0	0	0	0	0	6
Cluster19520	Acidobact	Acidobacteria_Gp2			Gp2	0	4	0	1	1	0	0	0	0	0	0	0	6
Cluster20220	Acidobacteria					2	0	0	2	2	0	0	0	0	0	0	0	6
Cluster20244	Acidobact	Acidobacteria_Gp3			Gp3	0	0	0	0	0	2	1	0	0	0	0	3	6
Cluster20370	Acidobacteria					3	0	0	0	1	0	2	0	0	0	0	0	6
Cluster20571	Proteobac	Gamm	Xanthomonadales			1	1	1	3	0	0	0	0	0	0	0	0	6
Cluster20836	Acidobact	Acidobacteria_Gp3			Gp3	2	2	0	0	0	0	2	0	0	0	0	0	6
Cluster20914	Bacteroid	Sphing	Sphir	Sphingc	Mucilaginibact	1	0	0	0	1	0	2	1	0	1	0	0	6
Cluster21800	Proteobac	Alphap	Rhod	Acetobacteraceae		0	1	1	0	1	1	0	0	0	2	0	0	6
Cluster21931	Acidobact	Acidobacteria_Gp1				1	1	0	1	1	1	0	1	0	0	0	0	6
Cluster22240	Actinobac	Actinol	Actin	Catenul	Catenulispora	0	0	0	0	0	0	1	4	0	0	0	1	6
Cluster23733;size=39						1	0	0	0	0	2	0	1	2	0	0	0	6
Cluster24905	Acidobact	Acidobacteria_Gp2			Gp2	0	0	0	2	0	0	1	0	1	0	2	0	6
Cluster25269	Bacteroid	Sphing	Sphir	Chitinophagaceae		0	1	0	0	1	0	1	3	0	0	0	0	6
Cluster25973	Actinobac	Actinol	Actinomycetales			0	0	1	2	0	0	1	1	0	0	1	0	6
Cluster27174	Bacteroid	Sphing	Sphir	Chitinophagaceae		0	1	0	0	0	2	2	0	1	0	0	0	6
Cluster27979	Proteobac	Gamm	Xanthomonadales			0	1	0	2	1	0	0	1	0	1	0	0	6
Cluster28933	Bacteroid	Sphing	Sphir	Sphingc	Mucilaginibact	0	0	1	2	0	1	0	0	1	0	0	1	6
Cluster28939	Bacteroid	Sphing	Sphir	Sphingc	Mucilaginibact	0	0	0	0	2	2	0	1	1	0	0	0	6
Cluster0;size=46						1	0	2	0	0	1	0	0	1	0	0	0	5
Cluster39;siz	Acidobact	Acidobacteria_Gp2			Gp2	1	3	1	0	0	0	0	0	0	0	0	0	5
Cluster77;siz	TM7				TM7_genera_i	0	1	0	0	0	0	1	1	0	1	1	0	5
Cluster94;siz	Proteobac	Alphap	Rhizobiales			0	3	0	0	0	0	0	0	2	0	0	0	5
Cluster98;siz	Acidobact	Acidobacteria_Gp2			Gp2	0	1	1	1	0	1	0	1	0	0	0	0	5
Cluster162;si	Acidobact	Acidobacteria_Gp1				1	1	0	0	0	2	0	0	0	0	1	0	5

Cluster164;si	Acidobact	Acidobacteria_Gp1				0	0	0	1	1	0	0	1	0	2	0	0	5
Cluster207;si	Proteobac	Alphap	Rhodospirillales			0	0	1	0	1	1	0	0	1	0	1	0	5
Cluster246;si	Verrucom	Spartobacteria			Spartobacteria	0	0	0	0	0	0	0	2	2	0	1	0	5
Cluster267;si	Acidobact	Acidobacteria_Gp3				0	3	0	0	0	0	0	0	0	1	0	1	5
Cluster309;size=23						0	1	0	0	1	1	1	0	0	1	0	0	5
Cluster368;si	Chlamydia	Chlamy	Chlar	Parachlamydiaceae		1	0	0	1	0	0	0	0	0	3	0	0	5
Cluster392;si	Proteobac	Alphap	Rhodospirillales			1	0	1	0	0	0	0	1	0	1	1	0	5
Cluster400;size=16						0	4	0	0	0	0	0	0	0	0	0	1	5
Cluster452;si	Proteobac	Betapr	Burkl	Burkhol	Burkholderia	1	0	0	0	1	0	1	0	0	0	2	0	5
Cluster453;si	Acidobact	Acidobacteria_Gp5			Gp5	1	0	0	1	0	0	0	0	0	2	1	0	5
Cluster571;si	Verrucom	Opitutae				3	0	0	0	0	1	0	0	1	0	0	0	5
Cluster619;si	Acidobact	Acidobacteria_Gp1			Gp1	0	0	0	0	0	1	1	1	1	0	0	1	5
Cluster665;size=31						2	0	0	1	2	0	0	0	0	0	0	0	5
Cluster713;size=18						0	0	0	0	3	1	0	0	1	0	0	0	5
Cluster747;si	Proteobac	Alphap	Rhizc	Xanthol	Pseudolabrys	0	0	1	1	0	3	0	0	0	0	0	0	5
Cluster764;si	Chlamydia	Chlamy	Chlamydiales			0	0	0	1	0	0	1	1	0	0	2	0	5
Cluster767;size=37						1	0	1	0	0	2	1	0	0	0	0	0	5
Cluster774;si	Acidobact	Acidobacteria_Gp1			Terriglobus	0	1	0	1	2	0	0	0	0	1	0	0	5
Cluster775;si	Acidobact	Acidobacteria_Gp1			Gp1	0	0	0	0	0	1	1	1	1	1	0	0	5
Cluster895;si	Acidobact	Acidobacteria_Gp1				1	0	0	0	1	1	0	0	1	0	1	0	5
Cluster941;si	Acidobact	Acidobacteria_Gp3			Gp3	1	0	0	1	0	0	0	1	0	0	2	0	5
Cluster961;si	Proteobac	Alphap	Rhizobiales			0	0	2	1	0	0	0	0	0	0	1	1	5
Cluster1016;	Bacteroid	Sphing	Sphir	Chitinophagaceae		1	2	0	0	0	0	0	0	0	1	0	1	5
Cluster1063;	Actinobac	Actinol	Acidimicrobiales			1	0	0	0	1	1	2	0	0	0	0	0	5
Cluster1094;	Acidobacteria					0	3	0	0	0	0	1	0	1	0	0	0	5
Cluster1095;	Proteobacteria					1	0	1	2	0	0	0	0	0	0	0	1	5
Cluster1097;	Acidobact	Acidobacteria_Gp1				1	2	0	0	0	1	0	0	1	0	0	0	5
Cluster1145;	Bacteroid	Sphing	Sphir	Chitinophagaceae		1	0	2	0	1	1	0	0	0	0	0	0	5
Cluster1147;	Proteobac	Betapr	Burkholderiales			0	0	1	0	0	0	0	1	0	2	0	1	5

Cluster1156;	Acidobact	Acidobacteria_Gp2		Gp2	0	0	1	0	0	0	1	1	2	0	0	0	5
Cluster1195;	Proteobac	Alphap	Rhodospirillales		0	0	0	0	0	0	0	0	0	5	0	0	5
Cluster1287;	Acidobact	Acidobacteria_Gp1		Gp1	0	0	0	0	0	1	0	1	1	0	1	1	5
Cluster1342;	Bacteroid	Sphing	Sphir	Chitinophagaceae	2	0	0	0	1	1	1	0	0	0	0	0	5
Cluster1353;	Acidobact	Acidobacteria_Gp1		Gp1	1	0	0	0	2	0	1	0	0	0	1	0	5
Cluster1429;	Acidobact	Acidobacteria_Gp3			1	0	0	0	1	1	0	0	1	1	0	0	5
Cluster1563;	Proteobac	Betapr	Burkl	Oxalobacteraceae	0	1	0	0	0	1	0	1	0	0	1	1	5
Cluster1564;	Acidobact	Acidobacteria_Gp1		Gp1	1	0	2	0	0	1	0	0	0	0	1	0	5
Cluster1570;	Bacteroid	Sphing	Sphir	Chitinophagaceae	0	0	1	0	0	0	0	1	1	2	0	0	5
Cluster1674;size=13					0	2	0	0	0	0	0	0	0	2	0	1	5
Cluster1728;	Acidobact	Acidobacteria_Gp1		Gp1	0	0	2	0	0	1	0	0	0	1	1	0	5
Cluster1780;	Acidobact	Acidobacteria_Gp2		Gp2	1	1	0	0	0	1	1	0	1	0	0	0	5
Cluster1830;	Verrucom	Opitut	Opitu	Opituta Opitutus	0	2	0	0	0	0	0	0	0	2	0	1	5
Cluster1907;	Proteobac	Alphap	Alphaproteob	Rhizomicrobiu	0	3	0	0	0	0	1	0	0	0	0	1	5
Cluster1934;	Acidobact	Acidobacteria_Gp1		Acidobacteriur	2	0	1	1	0	0	0	0	0	0	0	1	5
Cluster1946;	Planctomy	Plancto	Planc	Plancto Planctomyces	0	0	0	0	1	0	1	1	0	0	2	0	5
Cluster2053;size=40					0	2	0	0	0	0	1	1	0	0	1	0	5
Cluster2103;	Acidobact	Acidobacteria_Gp1		Gp1	0	1	1	0	0	0	1	0	1	1	0	0	5
Cluster2128;	Actinobac	Actinol	Actinomycetales		0	0	0	0	0	1	0	2	2	0	0	0	5
Cluster2134;	Actinobac	Actinol	Actin	Pseudonocardiaceae	0	0	0	0	0	0	0	2	0	0	0	3	5
Cluster2237;	Acidobact	Acidobacteria_Gp2		Gp2	0	0	0	0	2	0	0	0	2	1	0	0	5
Cluster2257;	Acidobact	Acidobacteria_Gp2		Gp2	0	0	0	2	1	0	1	0	0	0	0	1	5
Cluster2388;	Proteobac	Alphap	Caulc	Caulobacteraceae	1	1	0	0	1	0	1	0	0	1	0	0	5
Cluster2658;	Proteobac	Alphaproteobacteria			1	0	0	0	0	1	0	3	0	0	0	0	5
Cluster2697;	Acidobact	Acidobacteria_Gp2		Gp2	1	0	0	1	2	1	0	0	0	0	0	0	5
Cluster2768;	Acidobact	Acidobacteria_Gp1		Gp1	1	0	0	1	0	0	0	1	0	1	0	1	5
Cluster2810;	Bacteroid	Sphing	Sphir	Chitinophagaceae	0	0	2	1	1	0	0	0	0	1	0	0	5
Cluster3006;	Acidobact	Acidobacteria_Gp1			1	0	2	0	1	0	0	1	0	0	0	0	5
Cluster3016;	Acidobact	Acidobacteria_Gp2		Gp2	0	0	0	1	1	0	1	0	1	0	0	1	5

Cluster3088;:	Acidobact	Acidobacteria_Gp1			Gp1	0	0	1	0	0	1	2	1	0	0	0	0	5
Cluster3128;:	Acidobact	Acidobacteria_Gp1			Edaphobacter	0	0	1	0	0	1	0	1	1	0	0	1	5
Cluster3140;:	Proteobac	Betapr	Burkl	Burkhol	Burkholderia	0	0	0	2	3	0	0	0	0	0	0	0	5
Cluster3180;:	Planctomy	Plancto	Planc	Planctomycetaceae		0	1	2	0	1	1	0	0	0	0	0	0	5
Cluster3407;:	Acidobact	Acidobacteria_Gp2			Gp2	0	0	0	1	1	0	1	1	0	0	1	0	5
Cluster3437;:	Acidobacteria					0	1	0	0	1	0	1	0	0	0	1	1	5
Cluster3472;:	Acidobact	Acidobacteria_Gp2			Gp2	1	0	0	1	0	0	1	0	0	1	0	1	5
Cluster3574;:	Acidobact	Acidobacteria_Gp1				0	1	0	1	0	0	1	0	1	1	0	0	5
Cluster3690;size=14						0	0	0	2	1	1	1	0	0	0	0	0	5
Cluster3705;:	Bacteroid	Sphing	Sphir	Chitinophagaceae		2	0	1	0	1	0	0	0	1	0	0	0	5
Cluster3746;:	Proteobac	Alphap	Rhod	Acetoba	Acidisphaera	0	0	0	2	0	1	1	0	0	0	1	0	5
Cluster4014;:	Bacteroid	Sphing	Sphir	Chitinophagaceae		1	0	1	0	1	1	0	1	0	0	0	0	5
Cluster4033;:	Acidobact	Acidobacteria_Gp2			Gp2	0	0	0	0	0	0	0	1	2	0	1	1	5
Cluster4289;:	Proteobac	Alphap	Rhod	Rhodospirillaceae		0	1	0	0	1	0	0	1	0	1	0	1	5
Cluster4321;:	Proteobac	Alphap	Rhizc	Bradyrh	Bradyrhizobiur	1	0	0	0	1	0	0	0	0	1	1	1	5
Cluster4327;:	Actinobac	Actinol	Actinomycetales			0	0	1	0	1	0	0	0	1	1	0	1	5
Cluster4404;:	Proteobac	Deltap	Myxc	Polyangiaceae		0	1	0	0	0	1	1	0	0	1	1	0	5
Cluster4785;:	Proteobac	Gamm	Legio	Coxiella	Aquicella	0	0	3	1	0	0	0	0	0	0	1	0	5
Cluster4826;size=33						0	0	2	2	0	1	0	0	0	0	0	0	5
Cluster5681;:	Bacteroid	Sphing	Sphir	Sphingc	Mucilaginibact	0	0	0	1	0	0	0	2	0	0	2	0	5
Cluster5697;size=21						1	0	0	0	4	0	0	0	0	0	0	0	5
Cluster5704;:	Acidobact	Acidobacteria_Gp1				0	0	0	0	1	0	0	1	3	0	0	0	5
Cluster5828;:	Bacteroid	Sphing	Sphir	Sphingc	Mucilaginibact	0	0	0	0	0	1	0	2	2	0	0	0	5
Cluster6373;:	Acidobact	Acidobacteria_Gp3			Gp3	1	0	1	0	1	1	0	0	0	0	1	0	5
Cluster6749;:	Verrucom	Opituta	Opitu	Opituta	Opitutus	2	0	0	0	0	2	0	0	1	0	0	0	5
Cluster6945;:	Proteobac	Alphap	Rhodospirillales			3	0	0	0	0	0	0	1	1	0	0	0	5
Cluster7029;:	Acidobact	Acidobacteria_Gp5			Gp5	1	0	0	1	0	0	1	0	0	1	1	0	5
Cluster7173;:	Proteobac	Alphap	Rhodospirillales			0	1	0	1	0	0	0	0	0	3	0	0	5
Cluster7198;:	Proteobac	Alphaproteobacteria				0	0	3	0	1	1	0	0	0	0	0	0	5

Cluster7320;:	Gemmatii	Gemm	Gemi	Gemma	Gemmatimona	1	0	0	0	3	0	0	0	0	0	1	0	5
Cluster7464;:	Verrucom	Spartobacteria			Spartobacteria	1	1	0	0	0	0	1	1	0	0	0	1	5
Cluster7662;:	Acidobact	Acidobacteria_Gp3			Gp3	2	0	0	0	1	0	0	0	0	2	0	0	5
Cluster7674;:	Firmicutes					0	2	0	0	0	0	1	0	0	0	0	2	5
Cluster7722;:	Verrucom	Opitut	Opitu	Opituta	Opitutus	1	0	0	0	0	4	0	0	0	0	0	0	5
Cluster7803;:	Proteobac	Alphap	Rhod	Acetobacteraceae		0	0	1	0	0	1	0	2	0	0	1	0	5
Cluster7895;:	Proteobac	Deltap	Myxococcales			0	1	1	0	0	1	0	1	0	0	0	1	5
Cluster8022;size=32						2	0	0	0	0	1	1	0	0	0	1	0	5
Cluster8023;:	Proteobac	Alphap	Rhodospirillales			1	0	0	0	0	1	0	0	0	1	2	0	5
Cluster8325;size=9						0	1	0	0	0	0	0	1	1	0	2	0	5
Cluster8522;:	Acidobact	Acidobacteria_Gp3			Gp3	0	0	0	0	0	0	3	0	0	0	1	1	5
Cluster8587;:	Verrucom	Subdivision3			Subdivision3_g	2	1	0	0	1	0	0	0	1	0	0	0	5
Cluster8628;:	Acidobact	Acidobacteria_Gp2			Gp2	1	0	0	0	1	0	0	0	1	1	1	0	5
Cluster8784;:	Acidobact	Acidobacteria_Gp2			Gp2	1	1	0	0	0	2	1	0	0	0	0	0	5
Cluster8944;size=31						0	0	0	1	0	0	2	2	0	0	0	0	5
Cluster9121;:	Proteobac	Alphaproteobacteria				0	2	0	0	0	0	0	1	0	0	0	2	5
Cluster9217;:	Acidobact	Acidobacteria_Gp3				0	0	0	0	0	0	1	2	2	0	0	0	5
Cluster9373;:	Proteobac	Alphap	Rhod	Rhodospirillaceae		3	0	0	0	0	0	0	2	0	0	0	0	5
Cluster9906;:	Acidobact	Acidobacteria_Gp1			Granulicella	0	0	1	1	0	1	0	1	1	0	0	0	5
Cluster10612	Proteobac	Alphap	Caulo	Cauloba	Caulobacter	0	0	2	0	0	0	0	0	0	1	2	0	5
Cluster10824	Proteobac	Betapr	Burkl	Burkhol	Burkholderia	0	1	0	1	0	0	0	0	0	1	1	1	5
Cluster11024	Acidobact	Acidobacteria_Gp3			Gp3	0	0	0	0	0	0	0	0	2	0	3	0	5
Cluster11521	Acidobact	Acidobacteria_Gp5			Gp5	0	2	0	0	0	1	1	0	0	0	0	1	5
Cluster11672	Acidobact	Acidobacteria_Gp1				0	1	2	0	0	0	1	0	0	0	1	0	5
Cluster12086	Proteobac	Gamm	Xantl	Sinobac	Steroidobacter	0	0	0	1	0	1	2	0	1	0	0	0	5
Cluster12219	Proteobac	Alphap	Alphaproteob		Rhizomicrobiu	0	0	0	0	0	1	0	0	4	0	0	0	5
Cluster12222	Acidobact	Acidobacteria_Gp1			Gp1	1	0	0	0	0	0	1	0	0	1	2	0	5
Cluster12836	Proteobac	Deltaproteobacteria				0	0	2	1	0	0	1	0	1	0	0	0	5
Cluster12915	Acidobact	Acidobacteria_Gp2			Gp2	0	0	3	1	0	0	0	0	0	0	1	0	5

Cluster1380(	Acidobact	Acidobacteria_Gp2			Gp2	0	0	0	0	2	0	1	1	0	0	0	1	5
Cluster1392(	Acidobact	Acidobacteria_Gp2			Gp2	0	0	0	0	0	0	2	1	0	0	2	0	5
Cluster1417(	Acidobact	Acidobacteria_Gp1			Gp1	0	1	0	0	0	1	2	0	1	0	0	0	5
Cluster14261	Proteobac	Betapr	Burkholderiales			2	0	1	0	0	1	0	0	1	0	0	0	5
Cluster1439(	Proteobac	Betapr	Burkl	Burkhol	Burkholderia	1	1	0	0	0	0	1	0	0	0	1	1	5
Cluster1459(	Acidobact	Acidobacteria_Gp1				1	0	0	1	3	0	0	0	0	0	0	0	5
Cluster15163;size=28						0	0	0	0	2	1	0	1	0	0	0	1	5
Cluster1525(	Acidobact	Acidobacteria_Gp1				1	0	0	0	2	0	0	1	1	0	0	0	5
Cluster1595(	Acidobact	Acidobacteria_Gp1				0	0	1	2	0	0	0	0	0	2	0	0	5
Cluster1606(	Acidobact	Acidobacteria_Gp2			Gp2	0	0	1	0	0	1	3	0	0	0	0	0	5
Cluster1624(	Proteobac	Deltaproteobacteria				0	0	0	0	0	1	0	0	2	0	2	0	5
Cluster1626(	Acidobact	Acidobacteria_Gp1				0	0	0	0	2	1	0	0	1	1	0	0	5
Cluster1746(	Acidobact	Acidobacteria_Gp2			Gp2	0	0	0	1	0	1	0	1	1	0	0	1	5
Cluster1801(	Acidobact	Acidobacteria_Gp2			Gp2	0	1	0	0	0	0	0	0	2	0	2	0	5
Cluster1816(	Actinobac	Actinol	Solirı	Conexib	Conexibacter	1	1	0	1	0	0	1	0	0	0	1	0	5
Cluster1848(	Acidobact	Acidobacteria_Gp1				2	0	0	0	0	0	1	0	1	1	0	0	5
Cluster1868(	Acidobact	Acidobacteria_Gp2			Gp2	1	1	0	1	1	0	1	0	0	0	0	0	5
Cluster1891(	Acidobact	Acidobacteria_Gp3			Gp3	1	0	0	0	1	1	1	0	0	1	0	0	5
Cluster1933(	Acidobact	Acidobacteria_Gp2			Gp2	0	0	1	0	0	1	2	0	0	0	0	1	5
Cluster20081	Actinobac	Actinol	Solirubrobacterales			0	2	1	0	0	0	0	0	0	1	1	0	5
Cluster2010(	Acidobact	Acidobacteria_Gp3			Gp3	0	1	1	2	0	0	0	0	1	0	0	0	5
Cluster2012(	Acidobact	Acidobacteria_Gp1				2	0	1	0	0	0	1	0	0	0	0	1	5
Cluster2028(	Proteobac	Alphap	Rhod	Acetobacteraceae		1	0	1	1	0	0	0	0	1	0	1	0	5
Cluster2038(	Acidobact	Acidobacteria_Gp3			Gp3	2	0	0	0	0	1	2	0	0	0	0	0	5
Cluster2059(	Acidobact	Acidobacteria_Gp2			Gp2	1	1	0	0	1	0	2	0	0	0	0	0	5
Cluster2086(	Proteobac	Gamm	Xantl	Sinobac	Steroidobacter	1	0	1	2	0	1	0	0	0	0	0	0	5
Cluster2101(	Actinobac	Actinol	Acidi	Acidimi	Aciditerrimona	2	0	0	2	1	0	0	0	0	0	0	0	5
Cluster2103(	Actinobac	Actinol	Acidimicrobiales			0	0	0	0	0	1	1	1	0	1	1	0	5
Cluster2113(	Chlamydia	Chlamy	Chlamydiales			0	1	0	0	0	0	0	0	0	0	0	4	5

Cluster21603;size=26						0	0	1	1	0	0	0	1	1	0	0	1	5
Cluster21671	Proteobac	Alphap	Rhod	Acetobacteraceae		0	0	0	0	0	0	0	1	0	1	3	0	5
Cluster21724	Acidobacteria					0	0	1	0	0	0	2	0	0	0	2	0	5
Cluster22037	Acidobact	Acidobacteria_Gp3			Gp3	2	0	1	0	0	0	0	1	0	1	0	0	5
Cluster22107;size=17						0	0	0	1	0	0	3	0	1	0	0	0	5
Cluster22165	Proteobac	Deltaproteobacteria				1	1	0	0	1	0	1	0	1	0	0	0	5
Cluster22443	Actinobac	Actinol	Actinomycetales			0	1	1	1	0	0	1	0	0	0	1	0	5
Cluster22792	Proteobac	Alphap	Caulc	Caulobacteraceae		0	0	2	1	0	0	0	0	0	0	2	0	5
Cluster22974	Proteobac	Gamm	Xanthomonadales			0	0	2	0	0	0	1	0	0	1	1	0	5
Cluster23066	Bacteroid	Sphing	Sphir	Chitinophagaceae		0	0	0	2	0	1	1	0	0	1	0	0	5
Cluster23438	Actinobac	Actinol	Actinomycetales			0	1	0	0	0	0	2	0	0	0	2	0	5
Cluster24477	Bacteroid	Sphing	Sphir	Chitinophagaceae		1	0	0	0	2	0	0	1	0	0	1	0	5
Cluster24510	Bacteroid	Sphing	Sphir	Chitinophagaceae		0	0	1	0	1	0	0	3	0	0	0	0	5
Cluster24514	Acidobact	Acidobacteria_Gp1			Gp1	0	0	0	0	0	1	1	1	1	1	0	0	5
Cluster24681	Proteobac	Alphap	Alphaproteob		Rhizomicrobiu	2	1	1	1	0	0	0	0	0	0	0	0	5
Cluster24840	Acidobact	Acidobacteria_Gp13			Gp13	0	2	0	0	0	0	1	1	0	0	0	1	5
Cluster25065	Actinobac	Actinobacteria				0	2	0	0	1	0	1	0	1	0	0	0	5
Cluster25407	Acidobact	Acidobacteria_Gp2			Gp2	1	0	0	0	0	0	1	1	1	0	1	0	5
Cluster25736	Actinobac	Actinol	Acidi	Acidimi	Aciditerrimona	1	0	1	0	0	0	0	0	0	1	2	0	5
Cluster25779	Bacteroid	Sphing	Sphir	Chitinophagaceae		0	0	0	0	1	1	0	1	0	2	0	0	5
Cluster26115	Proteobac	Alphaproteobacteria				1	0	1	0	0	1	0	0	2	0	0	0	5
Cluster26926	Actinobac	Actinol	Actinomycetales			0	0	0	0	0	0	1	2	0	1	0	1	5
Cluster26986	Bacteroid	Sphing	Sphir	Chitinophagaceae		0	0	0	1	0	0	0	0	0	2	2	0	5
Cluster27080	Acidobacteria					0	0	0	0	0	0	0	1	3	0	1	0	5
Cluster27365	Actinobac	Actinol	Acidimicrobiales			0	1	0	0	1	0	0	0	0	2	0	1	5
Cluster27637	Bacteroid	Sphing	Sphir	Chitinophagaceae		2	0	0	0	0	1	0	0	0	1	0	1	5
Cluster28121	Actinobac	Actinol	Acidi	Acidimi	Aciditerrimona	0	1	1	0	0	0	1	1	0	0	0	1	5
Cluster29289	Bacteroid	Bacteroidetes_incert			Ohtaekwangia	0	0	0	1	1	0	2	0	1	0	0	0	5
Cluster25;siz	Acidobact	Acidobacteria_Gp3			Gp3	0	1	0	0	1	0	0	0	2	0	0	0	4

Cluster72;siz	Proteobac	Alphap	Rhizc	Hyphon	Devosia	2	0	0	0	0	1	0	0	0	1	0	0	4
Cluster87;size=31						0	0	0	0	1	1	0	0	1	0	1	0	4
Cluster139;si	Acidobact	Acidobacteria_Gp2			Gp2	0	0	0	0	1	0	0	0	0	1	0	2	4
Cluster147;si	Acidobact	Acidobacteria_Gp3				0	2	0	0	0	1	0	0	0	1	0	0	4
Cluster172;si	Proteobac	Alphap	Rhizc	Xanthol	Pseudolabrys	1	0	0	0	0	2	0	0	0	1	0	0	4
Cluster236;si	Proteobac	Betapr	Burkl	Oxalobacteraceae		0	1	0	0	0	1	0	0	0	2	0	0	4
Cluster262;si	Proteobac	Alphap	Rhizc	Bradyrh	Bradyrhizobiur	1	0	0	1	1	0	1	0	0	0	0	0	4
Cluster364;si	Acidobact	Acidobacteria_Gp1				0	0	0	1	0	1	1	1	0	0	0	0	4
Cluster379;si	Proteobac	Betapr	Burkl	Burkhol	Burkholderia	0	1	0	0	1	0	0	0	0	2	0	0	4
Cluster380;si	Acidobact	Acidobacteria_Gp3			Gp3	0	0	0	0	1	0	0	0	1	2	0	0	4
Cluster389;si	Proteobac	Alphap	Rhodospirillales			1	1	0	0	0	1	0	1	0	0	0	0	4
Cluster411;si	Proteobac	Deltap	Bdell	Bdellovi	Vampirovibrio	1	2	0	0	0	0	0	0	0	0	0	1	4
Cluster463;size=18						0	0	0	0	1	2	0	0	1	0	0	0	4
Cluster476;si	Proteobac	Betapr	Burkl	Burkhol	Burkholderia	2	0	0	0	0	0	0	1	1	0	0	0	4
Cluster484;si	Proteobac	Gamm	Xantl	Sinobac	Steroidobacter	1	1	0	0	1	1	0	0	0	0	0	0	4
Cluster487;si	Proteobac	Gammaproteobacteria				1	0	0	0	1	1	0	0	0	0	0	1	4
Cluster535;si	Acidobact	Acidobacteria_Gp2			Gp2	0	0	0	0	1	0	2	0	0	0	1	0	4
Cluster562;si	Proteobac	Alphap	Rhizc	Xanthol	Pseudolabrys	2	1	0	0	0	0	0	0	0	0	0	1	4
Cluster611;si	Acidobact	Acidobacteria_Gp2			Gp2	0	0	0	1	0	0	0	1	0	0	2	0	4
Cluster616;si	Proteobac	Alphap	Rhodospirillales			0	1	1	1	0	0	0	0	0	1	0	0	4
Cluster646;si	Acidobact	Acidobacteria_Gp2			Gp2	0	1	0	1	0	1	0	0	1	0	0	0	4
Cluster706;si	Proteobac	Deltap	Bdell	Bdellovi	Vampirovibrio	1	1	0	0	2	0	0	0	0	0	0	0	4
Cluster711;si	Gemmatii	Gemm	Gemi	Gemma	Gemmatimona	0	2	0	1	1	0	0	0	0	0	0	0	4
Cluster719;si	Acidobact	Acidobacteria_Gp15			Gp15	0	1	0	0	1	1	0	0	0	0	1	0	4
Cluster758;si	Gemmatii	Gemm	Gemi	Gemma	Gemmatimona	0	1	0	0	0	0	0	0	1	2	0	0	4
Cluster785;si	Acidobact	Acidobacteria_Gp1				0	0	0	1	1	1	0	0	0	0	1	0	4
Cluster788;si	Acidobact	Acidobacteria_Gp3			Gp3	0	0	0	0	0	0	0	1	3	0	0	0	4
Cluster795;si	Acidobact	Acidobacteria_Gp2			Gp2	0	0	1	0	0	0	2	0	0	0	1	0	4
Cluster797;size=16						0	0	0	1	2	0	0	0	0	0	0	1	4

Cluster798;si	Acidobact	Acidobacteria_Gp1			Edaphobacter	0	0	0	0	2	0	0	1	0	1	0	0	4
Cluster869;si	Acidobact	Acidobacteria_Gp2			Gp2	1	0	0	1	0	0	1	0	1	0	0	0	4
Cluster873;si	Acidobact	Acidobacteria_Gp15			Gp15	0	0	0	0	0	1	0	0	1	1	1	0	4
Cluster905;si	Proteobacteria					1	0	0	0	0	0	0	2	1	0	0	0	4
Cluster914;si	Proteobacteria					0	1	1	0	0	0	0	0	1	1	0	0	4
Cluster928;si	Acidobact	Acidobacteria_Gp1			Terriglobus	0	0	0	1	0	0	0	2	0	1	0	0	4
Cluster951;si	Actinobac	Actinol	Actin	Mycoba	Mycobacteriur	0	0	1	1	0	0	0	0	0	0	2	0	4
Cluster990;si	Proteobac	Alphap	Rhod	Rhodospirillaceae		0	1	0	0	0	0	0	2	1	0	0	0	4
Cluster1040;	Proteobac	Alphap	Sphir	Sphingc	Sphingomonas	2	0	0	0	0	1	0	0	0	0	1	0	4
Cluster1122;	Actinobac	Actinol	Acidi	Acidimi	Aciditerrimona	0	1	0	1	1	1	0	0	0	0	0	0	4
Cluster1124;	Proteobac	Alphap	Rhodospirillales			0	1	0	0	0	0	0	0	1	0	0	2	4
Cluster1125;	Proteobac	Alphap	Caulc	Caulobа	Phenylobacter	0	0	0	0	0	0	0	1	1	0	2	0	4
Cluster1126;size=15						0	0	0	0	1	0	1	0	1	0	1	0	4
Cluster1132;	Bacteroid	Sphing	Sphir	Chitinophagaceae		0	0	0	0	2	0	1	1	0	0	0	0	4
Cluster1265;	Acidobact	Acidobacteria_Gp2			Gp2	0	0	0	0	0	0	1	0	0	3	0	0	4
Cluster1272;	Proteobac	Alphap	Alphaproteob		Rhizomicrobiu	2	0	0	0	0	0	0	0	1	0	1	0	4
Cluster1282;	Acidobact	Acidobacteria_Gp2			Gp2	0	0	0	1	0	0	1	0	2	0	0	0	4
Cluster1292;	Acidobact	Acidobacteria_Gp6			Gp6	1	0	2	0	0	1	0	0	0	0	0	0	4
Cluster1297;	Acidobact	Acidobacteria_Gp2			Gp2	0	0	0	0	0	1	0	0	0	2	1	0	4
Cluster1308;	Proteobac	Deltaproteobacteria				0	0	0	0	0	0	1	0	0	0	2	1	4
Cluster1332;	Acidobact	Acidobacteria_Gp1			Gp1	0	3	0	0	0	0	0	0	1	0	0	0	4
Cluster1354;	Acidobact	Acidobacteria_Gp3			Gp3	0	0	0	0	2	0	1	0	0	1	0	0	4
Cluster1356;	Acidobact	Acidobacteria_Gp3			Gp3	0	0	0	2	1	1	0	0	0	0	0	0	4
Cluster1364;size=42						1	0	0	1	0	0	0	1	0	0	1	0	4
Cluster1373;size=32						0	0	0	2	2	0	0	0	0	0	0	0	4
Cluster1401;	Armatimc	Armati	Arma	Armatir	Armatimonas/	0	0	0	0	0	1	1	0	0	1	1	0	4
Cluster1415;	Acidobact	Acidobacteria_Gp1			Gp1	1	0	1	0	0	0	0	1	0	1	0	0	4
Cluster1418;	Acidobact	Acidobacteria_Gp6			Gp6	0	1	1	0	0	0	1	0	1	0	0	0	4
Cluster1519;	Proteobac	Gamm	Xantl	Sinobac	Nevskia	0	0	0	1	0	1	0	0	0	2	0	0	4

Cluster1539;	Proteobac	Betapr	Burkl	Oxaloba	Janthinobacter	0	0	0	1	0	0	0	0	0	2	1	0	4
Cluster1556;	Acidobact	Acidobacteria_Gp3			Gp3	0	1	0	0	0	1	1	0	0	0	1	0	4
Cluster1561;	Acidobact	Acidobacteria_Gp6			Gp6	0	0	1	1	0	1	0	0	1	0	0	0	4
Cluster1599;	Bacteroid	Sphing	Sphir	Chitino	Chitinophaga	0	0	0	0	0	1	0	2	1	0	0	0	4
Cluster1632;	Actinobac	Actinol	Actin	Streptomycetaceae		0	0	0	0	0	0	0	0	1	3	0	0	4
Cluster1665;	Proteobac	Alphap	Rhod	Acetoba	Acidisphaera	3	0	0	0	0	1	0	0	0	0	0	0	4
Cluster1668;	Proteobac	Alphap	Caulc	Cauloba	Phenylobacter	0	1	1	0	0	0	0	0	0	2	0	0	4
Cluster1672;	Actinobac	Actinobacteria				0	0	0	0	0	1	0	0	1	1	1	0	4
Cluster1718;	Proteobac	Alphaproteobacteria				0	0	0	0	0	3	0	1	0	0	0	0	4
Cluster1766;	Acidobact	Acidobacteria_Gp3			Gp3	0	1	0	0	0	1	0	1	1	0	0	0	4
Cluster1774;	Acidobact	Acidobacteria_Gp13			Gp13	0	1	0	0	1	0	0	2	0	0	0	0	4
Cluster1782;	Chloroflexi					3	0	0	0	0	1	0	0	0	0	0	0	4
Cluster1788;	Acidobact	Acidobacteria_Gp2			Gp2	0	0	1	1	0	0	0	1	0	0	1	0	4
Cluster1838;size=18						0	0	2	0	0	0	1	1	0	0	0	0	4
Cluster1866;	Actinobac	Actinol	Solirubrobacterales			0	0	2	0	0	0	1	0	0	1	0	0	4
Cluster1932;	Proteobac	Betaproteobacteria				0	2	0	0	0	0	0	0	0	1	0	1	4
Cluster1933;	Acidobact	Acidobacteria_Gp1			Gp1	0	0	0	0	2	0	0	0	1	0	1	0	4
Cluster2009;	Acidobact	Acidobacteria_Gp3			Gp3	1	0	1	0	1	0	0	0	0	1	0	0	4
Cluster2079;	Proteobac	Alphap	Alphaproteol		Rhizomicrobiu	0	0	1	0	0	0	0	1	0	2	0	0	4
Cluster2094;size=23						0	0	0	0	1	1	0	1	1	0	0	0	4
Cluster2138;	Chlamydia	Chlamy	Chlamydiales			2	0	0	2	0	0	0	0	0	0	0	0	4
Cluster2180;	Acidobact	Acidobacteria_Gp1			Gp1	1	1	0	0	1	0	0	0	1	0	0	0	4
Cluster2246;	Acidobact	Acidobacteria_Gp2			Gp2	0	1	0	0	0	3	0	0	0	0	0	0	4
Cluster2262;	Acidobact	Acidobacteria_Gp10			Gp10	0	0	0	1	0	0	0	1	0	1	0	1	4
Cluster2267;size=5						1	0	1	0	0	1	0	0	0	0	1	0	4
Cluster2312;	Proteobac	Alphap	Rhizc	Xanthol	Labrys	0	1	0	0	1	0	0	0	0	1	0	1	4
Cluster2317;	Acidobact	Acidobacteria_Gp3				0	2	0	1	0	0	1	0	0	0	0	0	4
Cluster2449;	Proteobac	Alphap	Caulc	Caulobacteraceae		0	0	1	1	0	0	0	0	0	2	0	0	4
Cluster2592;	Acidobact	Acidobacteria_Gp1				0	0	0	0	1	1	1	0	1	0	0	0	4

Cluster2612;size=20						0	1	0	0	1	1	0	0	0	0	0	1	4
Cluster2693;	Proteobac	Betapr	Burkl	Oxaloba	Collimonas	0	0	0	0	0	1	0	3	0	0	0	0	4
Cluster2739;	Bacteroid	Sphing	Sphir	Chitinophagaceae		0	0	1	1	1	1	0	0	0	0	0	0	4
Cluster2954;	Actinobac	Actinob	Actinomycetales			0	0	1	0	0	0	0	0	0	1	2	0	4
Cluster2967;	Proteobac	Betaproteobacteria				1	0	0	1	1	0	1	0	0	0	0	0	4
Cluster2978;	Acidobact	Acidobacteria_Gp1				0	0	0	4	0	0	0	0	0	0	0	0	4
Cluster3040;	Proteobac	Alphap	Rhod	Rhodospirillaceae		0	1	0	0	0	1	1	0	0	0	0	1	4
Cluster3135;	Actinobac	Actinob	Actin	Nocardi	Nocardia	0	1	0	0	0	1	0	1	0	1	0	0	4
Cluster3160;	Actinobac	Actinob	Actin	Nocardioidaceae		0	0	0	0	0	3	1	0	0	0	0	0	4
Cluster3221;	Proteobac	Alphap	Rhodospirillales			0	0	1	0	0	2	0	0	1	0	0	0	4
Cluster3272;	Actinobac	Actinob	Acidimicrobiales			0	1	0	0	2	1	0	0	0	0	0	0	4
Cluster3327;size=34						0	0	0	0	0	0	0	1	1	0	1	1	4
Cluster3492;	Proteobac	Gamm	Xanth	Sinobac	Steroidobacter	0	0	0	2	0	0	1	1	0	0	0	0	4
Cluster3593;	Chlamydia	Chlamy	Chlam	Parachlamydiaceae		1	0	1	0	2	0	0	0	0	0	0	0	4
Cluster3594;	Acidobact	Acidobacteria_Gp1				0	0	1	0	0	0	0	1	0	0	1	1	4
Cluster3662;size=10						2	0	0	0	2	0	0	0	0	0	0	0	4
Cluster3699;	Actinobac	Actinob	Actinomycetales			0	1	0	1	2	0	0	0	0	0	0	0	4
Cluster3768;	Proteobac	Deltap	Myxococcales			1	0	0	0	0	3	0	0	0	0	0	0	4
Cluster3834;	Armatimo	Armati	Arma	Armatin	Armatimonas/	1	0	0	0	0	0	1	0	2	0	0	0	4
Cluster3877;size=40						0	0	0	0	0	1	0	0	2	0	1	0	4
Cluster3957;	Bacteroid	Sphing	Sphir	Chitinop	Ferruginibacte	0	3	0	0	0	0	0	0	0	0	1	0	4
Cluster4123;	Actinobac	Actinob	Actin	Pseudonocardiaceae		0	0	0	0	1	2	0	0	0	0	0	1	4
Cluster4223;	Acidobact	Acidobacteria_Gp2			Gp2	0	0	0	1	1	0	0	0	1	1	0	0	4
Cluster4334;	Proteobac	Deltap	Bdell	Bdellovi	Vampirovibrio	0	2	0	0	0	0	0	0	1	0	1	0	4
Cluster4370;	Actinobac	Actinob	Acidimicrobiales			0	0	1	0	0	1	1	0	0	0	0	1	4
Cluster4432;	Planctom	Plancto	Planc	Plancto	Singulisphaera	0	0	0	1	0	0	1	0	0	0	1	1	4
Cluster4673;size=19						0	2	0	0	0	0	0	0	0	2	0	0	4
Cluster4680;size=15						1	0	0	0	0	0	0	3	0	0	0	0	4
Cluster4725;	Verrucom	Subdivision3			Subdivision3_g	0	0	0	0	3	1	0	0	0	0	0	0	4

Cluster4803;size=22						0	0	0	1	1	0	0	0	1	0	1	0	4
Cluster4936;size=35						0	1	0	0	0	1	1	1	0	0	0	0	4
Cluster4946;:	Planctomy	Plancto	Planc	Plancto	Singulisphaera	0	2	0	0	0	1	1	0	0	0	0	0	4
Cluster5006;:	Proteobac	Alphap	Rhodospirillales			0	0	1	0	1	1	0	0	0	1	0	0	4
Cluster5202;:	Proteobac	Alphap	Rhodospirillales			0	1	0	0	2	0	0	0	0	0	0	1	4
Cluster5244;:	Proteobac	Alphap	Rhizo	Bradyrh	Bradyrhizobiur	0	3	0	0	0	0	0	0	0	0	0	1	4
Cluster5248;:	Proteobac	Alphap	Rhizobiales			0	0	0	1	0	0	1	0	0	2	0	0	4
Cluster5305;:	Proteobac	Alphaproteobacteria				0	0	0	1	0	0	0	0	0	0	3	0	4
Cluster5412;:	Verrucom	Opitut	Opitu	Opituta	Opitutus	0	0	0	0	0	1	0	0	0	2	1	0	4
Cluster5429;:	Proteobac	Alphap	Rhod	Acetobacteraceae		0	0	1	1	0	0	0	0	0	2	0	0	4
Cluster5430;:	Bacteroid	Sphing	Sphir	Chitinophagaceae		2	0	0	0	1	0	0	1	0	0	0	0	4
Cluster5470;:	Acidobact	Acidobacteria_Gp1				1	0	0	0	0	0	1	0	1	0	0	1	4
Cluster5683;:	Proteobac	Deltaproteobacteria				0	1	1	2	0	0	0	0	0	0	0	0	4
Cluster5692;:	Proteobac	Alphap	Caulo	Caulobа	Phenylobacter	0	0	1	0	0	0	0	0	0	0	0	3	4
Cluster5710;size=17						0	1	0	0	0	0	1	0	1	0	0	1	4
Cluster5808;:	Elusimicro	Elusim	Elusir	Elusimic	Elusimicrobiur	0	0	0	0	0	0	1	0	0	1	1	1	4
Cluster5812;:	Acidobact	Acidobacteria_Gp2			Gp2	0	0	2	0	0	0	1	0	0	0	1	0	4
Cluster5898;size=35						0	0	1	1	0	0	0	0	0	1	0	1	4
Cluster5945;:	Verrucom	Subdivision3			Subdivision3_g	0	0	0	0	0	1	0	0	0	1	2	0	4
Cluster6009;:	Acidobact	Acidobacteria_Gp2			Gp2	0	0	0	2	0	0	1	0	0	0	1	0	4
Cluster6141;size=18						0	0	1	0	0	0	0	0	1	0	1	1	4
Cluster6234;:	Acidobact	Acidobacteria_Gp3			Gp3	0	2	0	0	0	0	0	0	1	0	1	0	4
Cluster6260;:	Acidobact	Acidobacteria_Gp3			Gp3	0	0	0	0	0	1	1	1	0	0	1	0	4
Cluster6317;size=14						1	0	1	0	0	1	0	0	0	0	0	1	4
Cluster6440;:	Verrucom	Subdivision3			Subdivision3_g	0	1	1	0	0	0	0	0	0	0	2	0	4
Cluster6476;:	Proteobac	Gamm	Xantl	Sinobac	Steroidobacter	1	1	0	0	0	0	0	0	0	0	0	2	4
Cluster6480;:	Proteobac	Deltap	Myxococcales			0	0	0	0	0	1	0	3	0	0	0	0	4
Cluster6509;:	Verrucom	Subdivision3			Subdivision3_g	0	0	0	0	0	1	1	1	0	0	0	1	4
Cluster6591;:	Firmicutes	Bacilli	Bacill	Bacillac	Bacillus	0	0	0	0	0	0	1	1	2	0	0	0	4

Cluster6710;:	Proteobac	Alphap	Rhizobiales			1	0	0	0	1	1	0	0	0	1	0	0	4
Cluster7030;:	Acidobact	Acidobacteria_Gp3			Gp3	0	1	1	2	0	0	0	0	0	0	0	0	4
Cluster7076;:	Acidobact	Acidobacteria_Gp3			Gp3	0	2	0	0	0	1	0	0	0	1	0	0	4
Cluster7116;:	Bacteroid	Sphing	Sphingobacteriales			1	1	0	0	0	0	0	2	0	0	0	0	4
Cluster7129;:	Acidobact	Acidobacteria_Gp1			Gp1	0	0	0	1	3	0	0	0	0	0	0	0	4
Cluster7432;size=12						0	1	0	0	0	1	0	0	0	2	0	0	4
Cluster7434;:	Proteobac	Alphap	Rhod	Acetoba	Acidisphaera	2	0	1	1	0	0	0	0	0	0	0	0	4
Cluster7633;:	Acidobact	Acidobacteria_Gp3			Gp3	1	0	0	1	0	0	1	0	1	0	0	0	4
Cluster7854;:	Proteobac	Betapr	Burkholderiales			1	0	1	0	0	0	0	1	0	0	1	0	4
Cluster7860;:	Verrucomicrobia					1	0	2	0	0	0	0	0	0	0	0	1	4
Cluster7937;:	Proteobac	Alphap	Rhodospirillales			0	0	0	0	2	0	0	0	0	0	0	2	4
Cluster7970;size=7						1	0	0	0	0	1	0	0	1	0	1	0	4
Cluster8215;size=23						0	0	1	0	0	0	1	0	0	0	1	1	4
Cluster8233;:	Proteobac	Betapr	Burkl	Burkhol	Burkholderia	4	0	0	0	0	0	0	0	0	0	0	0	4
Cluster8237;:	Acidobact	Acidobacteria_Gp1			Gp1	0	0	0	0	1	0	2	1	0	0	0	0	4
Cluster8334;:	Proteobac	Gamm	Xantl	Sinobac	Steroidobacter	0	0	1	3	0	0	0	0	0	0	0	0	4
Cluster8394;:	Proteobac	Alphap	Rhodospirillales			1	0	0	0	0	0	0	0	0	0	2	1	4
Cluster8562;:	Proteobac	Alphap	Rhodospirillales			0	2	0	0	0	0	0	0	0	1	0	1	4
Cluster8812;size=44						1	0	1	0	0	0	0	1	0	0	1	0	4
Cluster9100;:	Verrucom	Spartobacteria			Spartobacteria	0	1	0	0	0	0	1	2	0	0	0	0	4
Cluster9405;:	Gemmatir	Gemm	Gemi	Gemma	Gemmatimona	0	2	0	0	0	0	0	0	0	0	0	2	4
Cluster9418;:	Acidobact	Acidobacteria_Gp1				0	0	0	1	0	0	1	1	0	0	1	0	4
Cluster9529;:	Proteobac	Betapr	Burkl	Burkhol	Burkholderia	0	1	0	1	0	1	0	0	0	0	0	1	4
Cluster9566;:	Acidobact	Acidobacteria_Gp2			Gp2	0	1	1	1	0	0	0	1	0	0	0	0	4
Cluster9614;:	Proteobac	Alphap	Rhod	Acetoba	Acidisphaera	2	0	0	0	1	1	0	0	0	0	0	0	4
Cluster9660;:	Proteobac	Alphap	Rhod	Acetobacteraceae		0	0	1	0	0	1	0	1	0	1	0	0	4
Cluster9729;:	Actinobac	Actinobacteria				1	0	0	0	2	0	0	0	1	0	0	0	4
Cluster10012	Proteobac	Alphap	Rhodospirillales			0	0	0	0	0	0	0	2	1	0	0	1	4
Cluster10075	TM7				TM7_genera_i	0	0	1	0	0	0	0	0	1	1	1	0	4

Cluster10162	Acidobact	Acidobacteria_Gp2			Gp2	0	0	0	0	0	0	0	1	1	0	1	1	4
Cluster10297	Proteobac	Deltap	Myxococcales			0	0	0	0	0	1	1	0	1	1	0	0	4
Cluster10414	Acidobact	Acidobacteria_Gp1				0	0	1	2	0	0	0	0	0	0	1	0	4
Cluster10445	Proteobac	Alphap	Rhizobiales			0	0	1	0	0	0	1	0	1	0	0	1	4
Cluster10764	Proteobac	Betapr	Burkl	Burkhol	Burkholderia	0	1	1	1	0	1	0	0	0	0	0	0	4
Cluster10800	Proteobac	Deltap	Myxc	Cystoba	Cystobacter	0	1	0	0	1	1	1	0	0	0	0	0	4
Cluster10808	Acidobact	Acidobacteria_Gp1				0	0	0	1	0	0	0	0	0	2	1	0	4
Cluster10964	Actinobac	Actinol	Actinomycetales			0	1	0	0	0	0	0	0	0	1	1	1	4
Cluster11256	Acidobact	Acidobacteria_Gp1				0	0	4	0	0	0	0	0	0	0	0	0	4
Cluster11392	Proteobac	Deltap	Myxc	Cystobacteraceae		1	1	0	0	1	0	0	0	0	1	0	0	4
Cluster11761	Gemmatir	Gemm	Gemi	Gemma	Gemmatimona	0	0	1	1	0	0	0	0	0	1	1	0	4
Cluster12094;size=16						0	0	1	0	0	0	1	2	0	0	0	0	4
Cluster12408	Proteobac	Betaproteobacteria				0	3	0	0	0	0	0	0	0	0	0	1	4
Cluster12420	Proteobac	Deltaproteobacteria				0	0	0	0	1	0	2	0	0	0	0	1	4
Cluster12511	Proteobac	Alphap	Rhizc	Bradyrhizobiaceae		0	0	3	0	0	0	0	0	0	0	0	1	4
Cluster12603	Proteobac	Alphap	Rhodospirillales			0	1	0	1	0	0	1	1	0	0	0	0	4
Cluster12625	Proteobac	Alphap	Rhod	Acetoba	Acidocella	0	2	0	0	0	0	0	1	0	0	1	0	4
Cluster12700	Acidobact	Acidobacteria_Gp1				0	0	0	1	0	0	0	1	0	0	1	1	4
Cluster13008	Acidobact	Acidobacteria_Gp2			Gp2	0	0	4	0	0	0	0	0	0	0	0	0	4
Cluster13187	Proteobac	Alphap	Rhizobiales			4	0	0	0	0	0	0	0	0	0	0	0	4
Cluster13548;size=22						0	2	1	0	0	1	0	0	0	0	0	0	4
Cluster13853	Acidobact	Acidobacteria_Gp1			Gp1	0	0	0	0	0	1	1	0	0	0	2	0	4
Cluster13983	Acidobact	Acidobacteria_Gp1			Gp1	0	0	0	1	0	1	0	1	0	0	0	1	4
Cluster14925	Acidobact	Acidobacteria_Gp1			Gp1	0	0	0	0	1	1	0	0	0	0	2	0	4
Cluster15404	Actinobac	Actinol	Acidimicrobiales			0	1	0	0	0	0	0	0	0	1	1	1	4
Cluster15499	Acidobact	Acidobacteria_Gp1				0	0	0	0	0	0	0	0	3	0	0	1	4
Cluster15661	Acidobact	Acidobacteria_Gp2			Gp2	0	0	1	0	0	1	0	0	0	0	0	2	4
Cluster16002	Acidobact	Acidobacteria_Gp3			Gp3	1	0	0	2	0	0	0	0	0	1	0	0	4
Cluster16089	Acidobact	Acidobacteria_Gp3			Gp3	0	4	0	0	0	0	0	0	0	0	0	0	4

Cluster16093	Firmicutes					0	0	2	0	0	0	0	0	0	1	1	0	4
Cluster16284	Acidobact	Acidobacteria_Gp3			Gp3	0	0	0	2	0	0	0	1	0	0	1	0	4
Cluster16381	Acidobact	Acidobacteria_Gp2			Gp2	0	0	0	0	1	0	0	1	2	0	0	0	4
Cluster16407	Acidobact	Acidobacteria_Gp1			Gp1	0	0	0	0	0	1	0	0	2	1	0	0	4
Cluster16534	Acidobact	Acidobacteria_Gp1				0	0	0	0	0	1	0	0	0	2	1	0	4
Cluster16591	Gemmatir	Gemm	Gem	Gemma	Gemmatimona	0	0	0	0	0	0	0	1	1	1	1	0	4
Cluster16805	Proteobac	Deltaproteobacteria				0	0	1	2	0	0	0	0	0	0	0	1	4
Cluster16846	Acidobact	Acidobacteria_Gp1				0	0	2	1	0	1	0	0	0	0	0	0	4
Cluster16860	Acidobact	Acidobacteria_Gp1				0	0	0	1	0	0	0	0	0	2	1	0	4
Cluster17047	Chloroflex	Ktedor	Kted	Ktedon	Ktedonobacter	0	0	0	0	1	0	0	2	0	0	1	0	4
Cluster17154	Actinobac	Actinol	Actin	Streptor	Kitasatospora	0	0	1	0	0	0	0	0	1	0	1	1	4
Cluster17165	Acidobact	Acidobacteria_Gp2			Gp2	0	0	2	0	0	0	1	0	0	0	1	0	4
Cluster17186	Acidobact	Acidobacteria_Gp1			Gp1	0	0	2	1	0	0	0	0	0	0	1	0	4
Cluster17188	Verrucom	Opitut	Opitu	Opituta	Opitutus	0	0	0	0	0	1	0	0	0	3	0	0	4
Cluster17384	Acidobact	Acidobacteria_Gp1				1	1	0	1	0	0	0	0	0	1	0	0	4
Cluster17936	Acidobact	Acidobacteria_Gp3				0	1	0	0	0	0	0	1	0	0	1	1	4
Cluster18083	Proteobac	Alphap	Rhodospirillales			1	0	0	0	0	0	0	0	0	0	3	0	4
Cluster18181	Proteobac	Betapr	Rhod	Rhodocyclaceae		3	0	0	0	1	0	0	0	0	0	0	0	4
Cluster18344	Acidobact	Acidobacteria_Gp2			Gp2	0	0	1	2	0	0	0	0	0	0	1	0	4
Cluster18486	Acidobact	Acidobacteria_Gp2			Gp2	1	2	0	0	0	0	0	0	1	0	0	0	4
Cluster18510	Bacteroid	Sphing	Sphir	Chitinophagaceae		1	0	0	0	0	2	0	0	0	1	0	0	4
Cluster18516	Acidobact	Acidobacteria_Gp2			Gp2	0	0	0	0	1	0	0	1	0	0	2	0	4
Cluster18520	Proteobac	Alphap	Rhod	Acetobacteraceae		0	0	0	0	0	0	0	0	1	2	1	0	4
Cluster18713	Acidobact	Acidobacteria_Gp2			Gp2	0	0	1	3	0	0	0	0	0	0	0	0	4
Cluster18773	Acidobact	Acidobacteria_Gp2			Gp2	2	1	0	0	1	0	0	0	0	0	0	0	4
Cluster18814	Acidobact	Acidobacteria_Gp2			Gp2	0	0	2	0	0	0	1	0	1	0	0	0	4
Cluster19021	Acidobact	Acidobacteria_Gp2			Gp2	1	0	0	0	0	0	1	0	1	0	0	1	4
Cluster19172	Proteobac	Alphap	Alphaproteob		Rhizomicrobiu	1	0	0	0	0	0	0	1	0	0	1	1	4
Cluster19233	Acidobact	Acidobacteria_Gp3			Gp3	0	1	0	1	0	0	0	1	1	0	0	0	4

Cluster1970€	Acidobact	Acidobacteria_Gp3		Gp3	0	0	0	0	0	1	2	0	0	0	0	1	4
Cluster1978€	Proteobac	Betapr	Burkl	Burkhol	Pandoraea	0	0	0	0	0	0	2	0	0	1	1	0	4
Cluster20172	Acidobact	Acidobacteria_Gp1				0	0	0	2	1	1	0	0	0	0	0	0	4
Cluster20395	Proteobac	Betapr	Burkl	Burkhol	Burkholderia	0	0	2	2	0	0	0	0	0	0	0	0	4
Cluster20425	Acidobact	Acidobacteria_Gp3			Gp3	0	0	1	1	1	0	0	0	0	1	0	0	4
Cluster20634	Proteobac	Gammaproteobacteria				0	0	1	1	1	0	0	0	0	0	0	1	4
Cluster21344;size=15						0	0	1	1	0	0	0	1	0	0	1	0	4
Cluster2146€	Proteobac	Alphap	Rhodospirillales			0	0	1	3	0	0	0	0	0	0	0	0	4
Cluster21725	Acidobact	Acidobacteria_Gp1				0	0	1	0	2	0	1	0	0	0	0	0	4
Cluster21783	Proteobac	Alphap	Rhod	Acetobacteraceae		0	0	0	0	0	0	0	0	0	2	2	0	4
Cluster21853	Acidobact	Acidobacteria_Gp2			Gp2	0	1	0	0	0	0	0	1	1	0	1	0	4
Cluster21983	Proteobac	Alphap	Rhodospirillales			0	0	0	1	0	0	3	0	0	0	0	0	4
Cluster22104	Acidobact	Acidobacteria_Gp2			Gp2	1	0	0	0	0	0	0	2	0	1	0	0	4
Cluster2218C	Bacteroid	Sphing	Sphir	Chitinophagaceae		1	0	0	2	0	0	0	0	0	0	1	0	4
Cluster22271	Actinobac	Actinol	Actinomycetales			0	0	0	0	0	0	1	0	0	1	1	1	4
Cluster22321	Chlamydia	Chlamy	Chlar	Parachlamydiaceae		0	2	0	0	1	0	0	1	0	0	0	0	4
Cluster23857	Proteobac	Alphap	Caulc	Cauloba	Phenylobacter	0	0	1	0	1	1	0	0	0	1	0	0	4
Cluster23878	Proteobac	Gammaproteobacteria				0	0	0	0	3	1	0	0	0	0	0	0	4
Cluster24368	Bacteroid	Sphing	Sphir	Chitinophagaceae		0	0	1	0	1	0	0	1	0	0	1	0	4
Cluster24594	Actinobac	Actinol	Actinomycetales			0	0	0	2	1	0	0	0	0	1	0	0	4
Cluster24675	Actinobac	Actinol	Actin	Thermo	Actinomadura	0	1	0	0	0	0	0	0	0	1	0	2	4
Cluster24778	Actinobac	Actinol	Actinomycetales			0	0	1	0	0	0	2	1	0	0	0	0	4
Cluster24807	Actinobac	Actinol	Actinomycetales			0	0	0	1	0	0	2	0	0	0	1	0	4
Cluster24870;size=10						0	0	0	1	0	0	0	2	0	0	1	0	4
Cluster25182	Proteobac	Alphap	Alphaproteob		Rhizomicrobiu	0	0	1	0	0	0	2	0	1	0	0	0	4
Cluster25742	Acidobact	Acidobacteria_Gp2			Gp2	0	1	0	0	0	1	1	1	0	0	0	0	4
Cluster25757	Proteobac	Gammaproteobacteria				0	0	0	0	1	1	0	1	0	1	0	0	4
Cluster26809	Acidobact	Acidobacteria_Gp3			Gp3	0	0	0	1	0	0	0	0	0	0	0	3	4
Cluster27201	Bacteroid	Sphing	Sphir	Chitinop	Sediminibacter	0	0	0	1	0	1	0	0	0	1	0	1	4

Cluster27931	Actinobac	Actinol	Actinomycetales			0	0	0	1	0	1	1	0	0	0	1	0	4
Cluster28996	Bacteroid	Sphing	Sphir	Sphingo	Mucilaginibact	0	0	0	0	0	0	1	0	1	1	1	0	4
Cluster86;siz	Proteobac	Alphap	Rhizo	Bradyrh	Agromonas	0	0	1	0	1	0	0	0	0	0	0	1	3
Cluster92;siz	Elusimicro	Elusim	Elusi	Elusimic	Elusimicrobium	0	0	1	1	0	1	0	0	0	0	0	0	3
Cluster160;si	Proteobac	Alphap	Rhodospirillales			0	2	0	0	0	0	0	0	1	0	0	0	3
Cluster220;si	Proteobac	Gammaproteobacteria				2	0	0	0	0	0	1	0	0	0	0	0	3
Cluster228;size=17						1	0	0	0	0	0	0	1	0	1	0	0	3
Cluster231;si	Acidobact	Acidobacteria_Gp2			Gp2	0	0	0	0	0	0	0	1	0	1	0	1	3
Cluster235;size=10						1	0	0	0	0	1	0	0	0	1	0	0	3
Cluster239;si	Acidobact	Acidobacteria_Gp2			Gp2	1	0	0	0	0	0	1	1	0	0	0	0	3
Cluster253;si	Acidobacteria					0	2	0	0	0	0	0	1	0	0	0	0	3
Cluster270;si	Proteobacteria					0	1	0	0	0	0	0	0	1	0	1	0	3
Cluster278;si	Proteobac	Gammaproteobacteria				0	0	0	2	0	0	1	0	0	0	0	0	3
Cluster297;si	Acidobact	Acidobacteria_Gp2			Gp2	0	0	1	0	0	0	1	0	0	0	0	1	3
Cluster311;si	Proteobac	Alphap	Rhizo	Phyllobacteriaceae		0	0	0	0	0	0	0	1	2	0	0	0	3
Cluster324;si	Proteobacteria					0	0	1	1	1	0	0	0	0	0	0	0	3
Cluster326;size=16						1	0	1	0	0	0	0	0	0	0	1	0	3
Cluster337;si	Acidobact	Acidobacteria_Gp1			Gp1	0	2	0	1	0	0	0	0	0	0	0	0	3
Cluster345;si	Proteobac	Alphap	Rhodospirillales			0	1	0	0	0	0	0	0	1	0	0	1	3
Cluster358;si	Proteobac	Gamm	Xanth	Sinobac	Steroidobacter	0	0	0	0	1	0	0	0	0	2	0	0	3
Cluster363;si	Acidobact	Acidobacteria_Gp2			Gp2	0	0	0	0	0	0	0	0	2	0	0	1	3
Cluster403;size=5						0	0	0	0	1	0	0	1	0	1	0	0	3
Cluster407;si	Acidobact	Acidobacteria_Gp1			Gp1	0	0	1	1	0	0	0	0	0	1	0	0	3
Cluster414;si	Proteobac	Alphap	Rhizobiales			0	1	0	0	1	1	0	0	0	0	0	0	3
Cluster422;si	Acidobact	Acidobacteria_Gp3			Gp3	0	0	0	0	1	0	0	0	0	1	1	0	3
Cluster441;si	Acidobact	Acidobacteria_Gp2			Gp2	0	1	0	0	0	0	0	0	1	1	0	0	3
Cluster478;si	Acidobact	Acidobacteria_Gp2			Gp2	1	0	0	1	0	0	0	1	0	0	0	0	3
Cluster530;size=33						0	1	0	0	0	0	0	1	0	0	0	1	3
Cluster543;si	Actinobac	Actinol	Acidi	Acidimi	Aciditerrimona	1	0	0	1	0	0	0	0	0	0	0	1	3

Cluster550;si	Acidobact	Acidobacteria_Gp1			Gp1	0	0	0	0	1	1	0	0	1	0	0	0	3
Cluster582;si	Proteobac	Betaproteobacteria				0	0	0	0	0	0	0	1	1	1	0	0	3
Cluster591;si	Proteobac	Alphap	Rhodospirillales			2	0	0	0	0	0	0	0	0	0	1	0	3
Cluster598;si	Acidobact	Acidobacteria_Gp1			Gp1	0	0	0	1	0	0	1	0	1	0	0	0	3
Cluster650;si	Acidobact	Acidobacteria_Gp1			Gp1	0	0	1	0	0	0	0	0	0	1	1	0	3
Cluster661;si	Proteobac	Alphap	Rhod	Acetobacteraceae		0	0	1	1	0	0	0	0	0	0	1	0	3
Cluster664;size=28						1	1	0	0	0	0	0	0	0	1	0	0	3
Cluster766;size=12						0	0	1	1	0	0	1	0	0	0	0	0	3
Cluster769;si	Actinobac	Actinol	Solirubrobacterales			0	0	0	0	0	0	0	0	0	1	2	0	3
Cluster792;si	Chloroflex	Ktedor	Ktedonobacterales			1	0	0	0	0	0	0	2	0	0	0	0	3
Cluster799;si	Acidobact	Acidobacteria_Gp1			Gp1	0	1	0	0	0	1	0	0	1	0	0	0	3
Cluster804;si	Nitrospira	Nitrosp	Nitro	Nitrospi	Nitrospira	0	1	0	0	0	0	0	0	0	0	0	2	3
Cluster852;si	Proteobacteria					0	1	0	0	0	2	0	0	0	0	0	0	3
Cluster860;si	Actinobac	Actinol	Actin	Actinos	Actinospica	1	1	0	0	0	1	0	0	0	0	0	0	3
Cluster864;si	Verrucom	Spartobacteria			Spartobacteria	0	0	0	0	0	0	0	1	0	0	1	1	3
Cluster887;si	Acidobact	Acidobacteria_Gp1			Gp1	0	0	0	0	0	0	0	0	0	3	0	0	3
Cluster907;si	Gemmatir	Gemm	Gem	Gemma	Gemmatimona	0	0	0	0	0	0	0	2	0	1	0	0	3
Cluster912;si	Proteobac	Alphap	Rhizc	Bradyrhizobiaceae		0	0	1	0	1	1	0	0	0	0	0	0	3
Cluster924;si	Acidobact	Acidobacteria_Gp1			Gp1	0	0	0	0	0	0	0	0	0	3	0	0	3
Cluster943;si	Actinobac	Actinol	Solirı	Conexib	Conexibacter	0	0	0	0	0	0	2	1	0	0	0	0	3
Cluster954;si	Acidobact	Acidobacteria_Gp3			Gp3	0	2	0	0	0	0	0	0	1	0	0	0	3
Cluster958;si	Acidobact	Acidobacteria_Gp1				0	0	1	1	1	0	0	0	0	0	0	0	3
Cluster995;si	Proteobac	Gammaproteobacteria				1	0	0	0	0	0	0	1	0	1	0	0	3
Cluster1002;size=15						0	0	1	2	0	0	0	0	0	0	0	0	3
Cluster1030;	Proteobac	Alphaproteobacteria				1	0	0	1	0	0	0	0	0	0	1	0	3
Cluster1071;	Proteobac	Deltap	Myxc	Cystobacteraceae		0	0	1	1	0	1	0	0	0	0	0	0	3
Cluster1074;	Verrucom	Opitutae				2	0	0	0	0	1	0	0	0	0	0	0	3
Cluster1081;	Proteobac	Betapr	Rhod	Rhodocyclaceae		0	1	0	0	0	0	2	0	0	0	0	0	3
Cluster1090;	Acidobact	Acidobacteria_Gp1			Gp1	0	0	0	0	1	1	0	0	1	0	0	0	3

Cluster1092;	Proteobac	Gammaproteobacteria				1	0	0	0	0	0	0	0	0	2	0	0	3
Cluster1120;	Acidobact	Acidobacteria_Gp6			Gp6	1	0	0	0	1	0	1	0	0	0	0	0	3
Cluster1144;	Acidobact	Acidobacteria_Gp2			Gp2	0	1	1	0	0	0	0	0	0	0	0	1	3
Cluster1165;	Acidobact	Acidobacteria_Gp2			Gp2	0	2	0	0	0	0	0	0	0	0	0	1	3
Cluster1182;	Acidobact	Acidobacteria_Gp2			Gp2	0	0	0	0	0	0	1	0	0	2	0	0	3
Cluster1198;	Armatimc	Armati	Arma	Armatir	Armatimonas/	0	0	0	0	0	0	1	0	0	0	1	1	3
Cluster1207;	Verrucom	Opitut	Opitu	Opituta	Opitutus	0	1	0	0	0	1	0	0	0	1	0	0	3
Cluster1216;	Acidobact	Acidobacteria_Gp1			Acidobacteriur	0	0	0	1	0	0	0	0	0	2	0	0	3
Cluster1252;	Acidobact	Acidobacteria_Gp2			Gp2	0	0	0	1	0	0	0	1	0	0	0	1	3
Cluster1275;	Acidobact	Acidobacteria_Gp1			Gp1	0	0	0	0	1	1	1	0	0	0	0	0	3
Cluster1279;	Actinobac	Actinol	Actinomycetales			1	0	0	0	0	0	0	0	0	1	0	1	3
Cluster1310;	Gemmatir	Gemm	Gemi	Gemma	Gemmatimona	0	0	0	0	0	0	2	1	0	0	0	0	3
Cluster1331;	TM7				TM7_genera_i	0	0	0	0	2	0	0	1	0	0	0	0	3
Cluster1333;	Actinobac	Actinol	Acidimicrobiales			0	1	0	0	0	0	0	0	1	0	0	1	3
Cluster1344;	Acidobact	Acidobacteria_Gp1			Gp1	1	0	0	0	0	1	0	1	0	0	0	0	3
Cluster1379;size=21						0	0	0	1	0	0	1	0	0	0	0	1	3
Cluster1388;	Acidobact	Acidobacteria_Gp1				0	1	1	0	0	0	0	1	0	0	0	0	3
Cluster1404;	Proteobac	Alphap	Rhodospirillales			0	0	0	1	1	0	0	0	0	0	0	1	3
Cluster1449;	Armatimonadetes				Armatimonade	1	0	0	0	0	1	0	0	0	0	0	1	3
Cluster1452;	Acidobact	Acidobacteria_Gp2			Gp2	1	0	0	1	0	0	0	0	0	0	0	1	3
Cluster1454;	Proteobac	Deltap	Myxococcales			0	0	0	0	0	1	0	1	1	0	0	0	3
Cluster1491;	Acidobact	Acidobacteria_Gp1			Gp1	0	0	0	0	0	0	0	0	0	1	0	2	3
Cluster1516;	Proteobac	Deltap	Myxc	Cystobacteraceae		0	0	0	0	0	0	1	1	0	0	0	1	3
Cluster1528;	Chlamydia	Chlamy	Chlamydiales			0	0	0	0	1	1	1	0	0	0	0	0	3
Cluster1555;	Acidobact	Acidobacteria_Gp1				0	0	1	0	1	0	0	0	0	0	0	1	3
Cluster1559;size=22						1	0	0	0	0	0	1	0	0	0	1	0	3
Cluster1601;	Proteobac	Alphaproteobacteria				0	0	0	0	0	0	1	1	0	0	0	1	3
Cluster1609;	Acidobact	Acidobacteria_Gp1			Gp1	0	2	0	0	0	0	0	0	0	0	0	1	3
Cluster1626;	Bacteroid	Sphing	Sphir	Chitinophagaceae		0	0	0	0	0	1	2	0	0	0	0	0	3

Cluster1647;	Acidobact	Acidobacteria_Gp1			Gp1	0	0	0	2	0	0	0	1	0	0	0	0	3
Cluster1695;	Proteobac	Betapr	Burkl	Burkhol	Burkholderia	0	0	0	1	0	0	1	1	0	0	0	0	3
Cluster1717;	Proteobac	Alphap	Alphaproteob		Rhizomicrobiu	0	1	0	0	0	0	1	0	0	0	0	1	3
Cluster1739;	Acidobact	Acidobacteria_Gp1			Edaphobacter	0	0	1	0	0	0	1	0	0	1	0	0	3
Cluster1743;	Proteobac	Alphap	Rhodospirillales			0	0	0	0	0	0	0	0	1	1	1	0	3
Cluster1757;size=10						2	0	0	1	0	0	0	0	0	0	0	0	3
Cluster1768;	Proteobacteria					0	0	0	0	0	1	1	0	1	0	0	0	3
Cluster1801;size=15						0	0	0	0	1	0	0	0	1	1	0	0	3
Cluster1820;	Acidobact	Acidobacteria_Gp3			Gp3	0	1	0	0	0	0	0	0	0	0	0	2	3
Cluster1860;	Acidobact	Acidobacteria_Gp2			Gp2	0	1	1	0	0	0	1	0	0	0	0	0	3
Cluster1912;	Proteobac	Gammaproteobacteria				0	0	0	0	1	0	0	0	0	0	1	1	3
Cluster1921;	Acidobact	Acidobacteria_Gp6			Gp6	0	2	0	0	0	0	0	1	0	0	0	0	3
Cluster1926;size=12						0	1	0	0	0	0	0	1	1	0	0	0	3
Cluster1956;	Acidobact	Acidobacteria_Gp2			Gp2	1	0	0	0	0	0	0	0	0	1	0	1	3
Cluster1967;	Bacteroid	Sphing	Sphir	Chitinophagaceae		2	0	1	0	0	0	0	0	0	0	0	0	3
Cluster2013;	Proteobac	Gammaproteobacteria				0	0	0	0	0	2	0	0	0	1	0	0	3
Cluster2039;	Proteobac	Gamm	Xantl	Sinobac	Steroidobacter	1	0	0	1	0	0	0	0	0	0	1	0	3
Cluster2043;	Acidobact	Acidobacteria_Gp2			Gp2	0	0	0	1	0	0	1	0	0	0	0	1	3
Cluster2044;	Acidobact	Acidobacteria_Gp1				1	0	0	0	0	0	0	0	0	1	1	0	3
Cluster2063;	Proteobac	Deltaproteobacteria				1	0	0	1	0	0	0	0	0	1	0	0	3
Cluster2073;	Proteobac	Alphap	Alphaproteob		Rhizomicrobiu	0	0	0	1	0	0	1	0	0	0	1	0	3
Cluster2085;	Proteobac	Alphap	Rhodospirillales			0	0	0	1	0	0	0	0	0	2	0	0	3
Cluster2086;	Verrucom	Opitut	Opitu	Opituta	Opitutus	0	2	0	0	0	0	0	0	1	0	0	0	3
Cluster2098;	Acidobact	Acidobacteria_Gp2			Gp2	1	1	1	0	0	0	0	0	0	0	0	0	3
Cluster2117;	Proteobac	Deltap	Myxococcales			1	0	0	0	1	0	0	0	0	0	1	0	3
Cluster2135;	Planctomycetes					0	0	0	0	0	1	0	1	0	0	1	0	3
Cluster2143;	Proteobac	Gamm	Enter	Enterobacteriaceae		0	0	0	0	0	2	1	0	0	0	0	0	3
Cluster2189;	Proteobac	Betapr	Burkl	Burkhol	Burkholderia	2	0	0	0	0	0	1	0	0	0	0	0	3
Cluster2244;size=21						0	0	0	0	0	0	0	0	1	0	0	2	3

Cluster2291;	Proteobac	Alphap	Caulo	Caulobacteraceae		0	1	0	2	0	0	0	0	0	0	0	0	3
Cluster2324;	Actinobac	Actinol	Acidi	Acidimi	Aciditerrimona	0	0	0	0	1	0	1	0	1	0	0	0	3
Cluster2329;size=14						2	0	0	0	0	0	0	0	0	0	0	1	3
Cluster2364;	Acidobact	Acidobacteria_Gp1				0	0	2	1	0	0	0	0	0	0	0	0	3
Cluster2420;	Proteobac	Deltap	Myxococcales			0	1	0	0	2	0	0	0	0	0	0	0	3
Cluster2430;	Actinobac	Actinol	Actinomycetales			0	0	0	0	0	1	0	0	1	0	1	0	3
Cluster2451;	Elusimicro	Elusim	Elusi	Elusimic	Elusimicrobiun	2	1	0	0	0	0	0	0	0	0	0	0	3
Cluster2474;	Proteobac	Betapr	Burkl	Burkhol	Burkholderia	0	1	1	0	0	0	0	0	0	0	0	1	3
Cluster2503;size=9						0	0	0	0	0	0	0	1	0	1	0	1	3
Cluster2573;	Proteobac	Gamm	Legio	Coxiella	Aquicella	0	0	0	0	2	0	1	0	0	0	0	0	3
Cluster2609;	Acidobact	Acidobacteria_Gp3				0	0	0	0	1	0	1	0	0	0	0	1	3
Cluster2676;	Acidobact	Acidobacteria_Gp1			Gp1	0	2	0	0	0	0	0	0	1	0	0	0	3
Cluster2704;	Acidobact	Acidobacteria_Gp2			Gp2	0	0	0	0	0	0	0	2	0	0	0	1	3
Cluster2717;	Chloroflex	Ktedor	Ktedonobacterales			0	2	0	0	0	0	1	0	0	0	0	0	3
Cluster2781;	Chloroflex	Ktedor	Kted	Thermo	Thermosporot	0	0	1	0	1	1	0	0	0	0	0	0	3
Cluster2798;	Acidobact	Acidobacteria_Gp2			Gp2	0	0	0	2	0	0	0	1	0	0	0	0	3
Cluster2823;size=22						1	0	0	1	0	0	0	0	0	0	0	1	3
Cluster2864;	Actinobac	Actinol	Actin	Streptomycetaceae		1	1	0	0	0	0	0	0	0	1	0	0	3
Cluster2923;	Armatimonadetes				Armatimonade	0	0	1	0	0	0	0	0	0	2	0	0	3
Cluster2925;	Planctomy	Plancto	Planc	Plancto	Singulisphaera	0	0	0	0	1	0	1	1	0	0	0	0	3
Cluster2931;	Proteobac	Alphap	Rhizo	Bradyrh	Bradyrhizobiur	0	0	0	0	0	2	0	0	0	0	1	0	3
Cluster2933;	Proteobac	Gamm	Xantl	Sinobac	Steroidobacter	1	0	0	0	0	0	1	0	0	1	0	0	3
Cluster2957;	Acidobact	Acidobacteria_Gp3			Gp3	0	0	0	0	0	1	1	0	0	1	0	0	3
Cluster3036;	Acidobact	Acidobacteria_Gp3			Gp3	2	0	0	0	0	1	0	0	0	0	0	0	3
Cluster3063;	Acidobact	Acidobacteria_Gp1			Gp1	0	0	0	1	1	0	0	0	1	0	0	0	3
Cluster3076;size=11						0	0	0	0	0	0	0	0	0	1	0	2	3
Cluster3141;size=25						0	0	0	0	0	0	0	1	0	1	0	1	3
Cluster3191;	Acidobact	Acidobacteria_Gp2			Gp2	0	0	0	0	0	0	0	0	0	1	2	0	3
Cluster3217;	Proteobac	Alphaproteobacteria				0	0	0	0	0	1	0	0	0	0	0	2	3

Cluster3325;	Acidobact	Acidobacteria_Gp3				0	0	1	1	0	0	0	0	0	0	0	1	3
Cluster3352;	Proteobac	Alphap	Rhizobiales			0	1	0	0	0	0	0	1	0	1	0	0	3
Cluster3363;	Bacteroid	Sphing	Sphir	Sphingo	Mucilaginibact	0	0	0	0	0	2	0	1	0	0	0	0	3
Cluster3378;	Acidobact	Acidobacteria_Gp2			Gp2	0	0	2	0	0	1	0	0	0	0	0	0	3
Cluster3400;	Proteobac	Alphap	Rhodospirillales			0	0	0	0	0	1	1	0	0	0	0	1	3
Cluster3404;size=15						0	0	0	0	0	1	0	1	0	0	0	1	3
Cluster3421;	Proteobac	Alphap	Alphaproteob		Rhizomicrobiu	0	0	0	0	0	1	1	0	1	0	0	0	3
Cluster3427;	Verrucom	Subdivision3			Subdivision3_g	2	0	0	0	1	0	0	0	0	0	0	0	3
Cluster3448;	Planctomy	Plancto	Planc	Plancto	Singulisphaera	0	0	1	0	0	1	0	0	0	0	0	1	3
Cluster3449;size=14						0	0	1	1	0	0	0	0	0	0	1	0	3
Cluster3459;	Proteobac	Gamm	Legio	Coxiella	Aquicella	0	2	0	0	0	0	0	0	0	0	0	1	3
Cluster3474;	Actinobac	Actinol	Acidimicrobiales			0	0	0	0	0	1	0	1	0	0	0	1	3
Cluster3525;size=19						0	0	0	0	0	0	0	1	2	0	0	0	3
Cluster3589;	Actinobac	Actinol	Acidimicrobiales			0	0	0	0	1	0	1	1	0	0	0	0	3
Cluster3638;size=20						0	0	0	0	0	2	0	0	0	0	0	1	3
Cluster3652;	Proteobac	Alphap	Rhizo	Xanthob	Pseudolabrys	0	0	0	0	0	0	0	1	1	0	0	1	3
Cluster3675;	Planctomy	Phycisp	Phyci	Phycispl	Phycisphaera	0	0	0	0	1	1	0	1	0	0	0	0	3
Cluster3906;	Acidobact	Acidobacteria_Gp1			Granulicella	0	0	0	1	1	1	0	0	0	0	0	0	3
Cluster3918;	Proteobac	Deltap	Bdell	Bdellovi	Vampirovibrio	0	0	0	0	0	1	0	0	2	0	0	0	3
Cluster3920;size=4						1	1	0	1	0	0	0	0	0	0	0	0	3
Cluster3934;	Verrucom	Opituta	Opitu	Opituta	Opitutus	0	0	0	0	0	1	0	0	0	2	0	0	3
Cluster3938;	Chlorobi	Ignavib	Ignav	Ignaviba	Ignavibacteriu	0	0	0	0	0	1	1	0	0	0	1	0	3
Cluster4022;size=11						0	1	0	1	0	0	0	0	0	0	0	1	3
Cluster4156;	Proteobac	Alphap	Rhodospirillales			0	1	0	0	0	1	1	0	0	0	0	0	3
Cluster4318;	Planctomycetes					0	0	0	0	0	0	1	1	1	0	0	0	3
Cluster4458;size=14						0	0	0	1	0	0	0	0	1	0	0	1	3
Cluster4472;	Proteobacteria					0	0	0	0	0	0	1	1	1	0	0	0	3
Cluster4484;	Acidobact	Acidobacteria_Gp1				0	0	1	0	0	0	0	2	0	0	0	0	3
Cluster4509;	Proteobac	Alphap	Rhodospirillales			0	0	0	1	0	0	1	0	0	0	1	0	3

Cluster4549;	Bacteroid	Sphing	Sphir	Chitinophagaceae		0	1	0	0	0	0	0	1	0	0	0	1	3
Cluster4713;	Verrucom	Opituta	Opitu	Opituta	Opitutus	0	1	0	0	0	0	0	0	0	0	0	2	3
Cluster4758;	Verrucom	Subdivision3			Subdivision3_g	0	0	0	2	0	0	0	0	0	1	0	0	3
Cluster4760;	Verrucom	Subdivision3			Subdivision3_g	0	0	1	0	1	0	0	0	0	1	0	0	3
Cluster4767;	Acidobact	Acidobacteria_Gp1				0	0	0	1	0	0	1	0	0	0	1	0	3
Cluster4773;	Proteobac	Alphaproteobacteria				0	0	0	0	1	1	0	0	0	1	0	0	3
Cluster4814;	Proteobac	Alphap	Rhod	Rhodos	Dongia	0	0	0	0	0	0	0	0	0	2	1	0	3
Cluster4848;	Acidobact	Acidobacteria_Gp2			Gp2	1	1	0	0	0	0	0	1	0	0	0	0	3
Cluster4872;	Verrucom	Subdivision3			Subdivision3_g	0	1	0	0	1	0	0	0	1	0	0	0	3
Cluster4952;	Acidobact	Acidobacteria_Gp1				0	0	1	0	0	0	0	0	0	2	0	0	3
Cluster5130;	Proteobac	Alphap	Rhizc	Bradyrh	Bradyrhizobiur	0	0	0	0	1	0	0	1	1	0	0	0	3
Cluster5168;	Proteobac	Alphap	Rhodospirillales			0	0	1	0	1	0	1	0	0	0	0	0	3
Cluster5204;	Verrucom	Subdivision3			Subdivision3_g	0	0	0	0	0	1	0	1	0	0	0	1	3
Cluster5331;	Actinobac	Actinol	Acidi	Acidimi	Aciditerrimona	1	1	1	0	0	0	0	0	0	0	0	0	3
Cluster5379;	Actinobac	Actinobacteria				0	0	0	0	1	0	1	0	0	0	0	1	3
Cluster5439;	Proteobac	Gammaproteobacteria				0	0	0	0	1	0	1	0	1	0	0	0	3
Cluster5459;	Proteobac	Betapr	Rhod	Rhodocyclaceae		0	0	1	0	0	0	0	0	1	0	1	0	3
Cluster5471;	Proteobac	Alphap	Rhodospirillales			1	0	0	1	0	0	0	0	1	0	0	0	3
Cluster5563;	Proteobac	Alphap	Rhizobiales			0	1	0	1	0	0	0	0	0	0	0	1	3
Cluster5589;	Planctomycetes					0	0	0	1	0	0	0	0	1	1	0	0	3
Cluster5602;	Proteobac	Alphap	Rhizc	Rhizobia	Bauldia	0	0	0	1	0	0	0	1	0	0	1	0	3
Cluster5651;	Acidobact	Acidobacteria_Gp1				0	0	1	0	0	0	1	0	0	1	0	0	3
Cluster5712;	Proteobac	Alphap	Rhizc	Bradyrh	Bradyrhizobiur	1	1	0	0	0	0	0	0	0	1	0	0	3
Cluster5747;size=11						1	0	0	0	0	1	0	1	0	0	0	0	3
Cluster5798;	Proteobac	Alphap	Rhizc	Hyphon	Rhodoplanes	0	0	1	0	0	0	2	0	0	0	0	0	3
Cluster5806;	Acidobact	Acidobacteria_Gp3			Gp3	0	0	2	0	0	0	1	0	0	0	0	0	3
Cluster5825;	Proteobac	Alphap	Rhizobiales			1	0	0	1	0	0	1	0	0	0	0	0	3
Cluster5849;size=9						0	0	0	0	0	0	0	0	0	3	0	0	3
Cluster5886;	Verrucom	Opituta	Opitu	Opituta	Opitutus	0	0	0	0	0	0	0	0	0	3	0	0	3

Cluster5902;	Actinobac	Actinol	Acidi	Acidimi	Aciditerrimona	0	2	0	0	0	0	0	0	0	0	0	1	3
Cluster5933;size=14						0	3	0	0	0	0	0	0	0	0	0	0	3
Cluster5934;size=3						1	0	0	2	0	0	0	0	0	0	0	0	3
Cluster5998;	Proteobac	Deltap	Myxc	Cystobacteraceae		0	1	0	0	0	1	0	0	0	0	1	0	3
Cluster6028;	Acidobact	Acidobacteria_Gp1				0	0	1	0	1	0	0	0	0	1	0	0	3
Cluster6290;	Proteobac	Alphap	Rhodospirillales			0	0	0	0	0	1	0	1	0	0	1	0	3
Cluster6299;	Proteobac	Betapr	Rhod	Rhodocyclaceae		0	0	1	1	0	0	0	0	1	0	0	0	3
Cluster6369;	Acidobact	Acidobacteria_Gp2			Gp2	0	0	1	2	0	0	0	0	0	0	0	0	3
Cluster6441;	Acidobact	Acidobacteria_Gp3				0	1	0	0	0	0	1	0	1	0	0	0	3
Cluster6687;	Proteobac	Deltaproteobacteria				0	2	0	0	1	0	0	0	0	0	0	0	3
Cluster6701;	Verrucom	Subdivision3			Subdivision3_g	0	0	1	0	0	1	0	0	0	0	1	0	3
Cluster6718;size=6						1	0	0	0	0	2	0	0	0	0	0	0	3
Cluster6801;	Proteobac	Alphap	Rhizobiales			0	0	0	0	0	0	0	0	1	1	0	1	3
Cluster6919;	Acidobact	Acidobacteria_Gp2			Gp2	0	0	0	1	0	0	0	1	0	0	0	1	3
Cluster6993;	Acidobact	Acidobacteria_Gp3			Gp3	0	0	0	0	1	0	0	1	0	0	0	1	3
Cluster7266;	Acidobact	Acidobacteria_Gp1				0	0	0	0	1	1	0	0	0	0	1	0	3
Cluster7313;	Proteobac	Deltaproteobacteria				1	0	0	0	0	0	1	0	0	0	1	0	3
Cluster7458;	Proteobac	Deltap	Myxococcales			0	0	0	0	0	3	0	0	0	0	0	0	3
Cluster7468;	Chlamydia	Chlamy	Chlar	Parachlamydiaceae		0	0	2	1	0	0	0	0	0	0	0	0	3
Cluster7552;	Verrucom	Subdivision3			Subdivision3_g	0	0	2	1	0	0	0	0	0	0	0	0	3
Cluster7603;	Acidobact	Acidobacteria_Gp1			Gp1	0	1	1	0	0	0	0	0	1	0	0	0	3
Cluster7638;	Proteobac	Betapr	Burkl	Burkhol	Burkholderia	0	0	3	0	0	0	0	0	0	0	0	0	3
Cluster7645;	Proteobac	Deltap	Bdell	Bdellovi	Bdellovibrio	0	1	1	0	0	0	0	0	0	1	0	0	3
Cluster7793;	Verrucom	Subdivision3			Subdivision3_g	0	0	0	0	1	0	1	0	0	1	0	0	3
Cluster7802;	Proteobac	Betapr	Burkl	Oxalobacteraceae		0	0	0	0	0	1	0	1	0	1	0	0	3
Cluster8067;	Proteobac	Alphap	Rhodospirillales			0	0	0	0	0	0	2	0	0	1	0	0	3
Cluster8136;	Acidobact	Acidobacteria_Gp1				0	0	1	0	0	0	0	0	0	2	0	0	3
Cluster8186;	Proteobac	Alphap	Rhizc	Bradyrhizobiaceae		0	0	2	0	1	0	0	0	0	0	0	0	3
Cluster8272;	Acidobact	Acidobacteria_Gp1			Gp1	0	0	0	0	1	0	2	0	0	0	0	0	3

Cluster8296;size=4						1	0	0	0	0	1	0	0	0	0	0	1	3
Cluster8328;:	Acidobact	Acidobacteria_Gp1			Acidobacteriur	0	0	0	0	0	1	1	0	0	0	1	0	3
Cluster8419;:	Proteobac	Alphap	Rhodospirillales			1	0	0	1	0	0	1	0	0	0	0	0	3
Cluster8468;:	Proteobac	Deltaproteobacteria				0	0	0	0	0	1	0	0	1	0	1	0	3
Cluster8473;:	Proteobac	Alphaproteobacteria				0	0	0	0	1	0	0	0	0	0	2	0	3
Cluster8480;:	Proteobac	Alphap	Rhodospirillales			0	1	0	1	0	0	0	0	0	0	1	0	3
Cluster8553;:	Acidobact	Acidobacteria_Gp1				1	0	0	0	2	0	0	0	0	0	0	0	3
Cluster8637;:	Proteobac	Deltap	Myxc	Cystobacteraceae		0	0	0	0	0	0	1	0	0	1	1	0	3
Cluster8650;:	Acidobacteria					0	0	2	1	0	0	0	0	0	0	0	0	3
Cluster8662;:	Proteobac	Alphap	Rhodospirillales			0	0	0	0	2	0	0	0	1	0	0	0	3
Cluster8721;:	Verrucom	Opitut:	Opitu	Opituta	Opitutus	0	0	0	0	0	1	0	0	0	2	0	0	3
Cluster8732;size=3						0	0	0	0	1	0	1	0	0	0	1	0	3
Cluster8827;:	Proteobac	Alphap	Rhodospirillales			0	0	0	0	1	0	1	0	0	0	0	1	3
Cluster8834;:	Acidobact	Acidobacteria_Gp2			Gp2	0	1	0	0	0	0	0	0	1	0	1	0	3
Cluster8922;:	Acidobact	Acidobacteria_Gp2			Gp2	0	2	0	0	0	0	0	1	0	0	0	0	3
Cluster8997;:	Acidobact	Acidobacteria_Gp2			Gp2	0	0	1	1	0	0	0	1	0	0	0	0	3
Cluster9031;:	Acidobact	Acidobacteria_Gp2			Gp2	0	0	0	0	0	1	1	0	0	0	0	1	3
Cluster9068;size=13						0	1	0	0	0	0	0	0	1	0	0	1	3
Cluster9119;:	Proteobac	Alphaproteobacteria				0	0	0	0	0	0	1	2	0	0	0	0	3
Cluster9139;:	Proteobac	Betapr	Burkholderiales			0	0	0	1	0	0	1	1	0	0	0	0	3
Cluster9164;:	Bacteroid	Sphing	Sphir	Sphingc	Mucilaginibact	0	1	0	1	0	0	0	1	0	0	0	0	3
Cluster9235;size=38						0	1	0	0	0	0	0	0	0	1	1	0	3
Cluster9328;size=5						1	0	0	0	0	0	0	1	0	0	1	0	3
Cluster9329;:	Actinobac	Actinol	Actin	Mycoba	Mycobacteriur	0	0	2	1	0	0	0	0	0	0	0	0	3
Cluster9351;:	Proteobac	Deltap	Myxococcales			0	2	0	0	0	0	0	0	0	0	1	0	3
Cluster9482;:	Acidobact	Acidobacteria_Gp3			Gp3	1	0	1	0	0	0	1	0	0	0	0	0	3
Cluster9536;:	Acidobact	Acidobacteria_Gp1				0	0	2	1	0	0	0	0	0	0	0	0	3
Cluster9627;:	Proteobac	Alphap	Rhodospirillales			0	0	0	0	0	0	3	0	0	0	0	0	3
Cluster9634;:	Acidobacteria					0	0	0	1	1	0	0	0	0	0	1	0	3

Cluster9643;:	Proteobac	Betaproteobacteria				1	1	0	0	0	0	0	0	0	0	0	1	3
Cluster9675;:	Bacteroid	Sphing	Sphir	Chitinophagaceae		1	0	0	0	0	0	0	2	0	0	0	0	3
Cluster9689;:	Acidobact	Acidobacteria_Gp1				1	0	0	0	0	0	0	0	2	0	0	0	3
Cluster9749;:	Bacteroid	Sphing	Sphir	Sphingc	Mucilaginibact	0	0	1	0	1	0	0	0	0	1	0	0	3
Cluster9784;:	Actinobac	Actinol	Actin	Propion	Propionibacter	1	0	0	0	0	0	0	1	0	0	0	1	3
Cluster9895;size=5						0	0	2	0	0	0	1	0	0	0	0	0	3
Cluster10237	Acidobact	Acidobacteria_Gp1				0	0	2	1	0	0	0	0	0	0	0	0	3
Cluster10437	Acidobact	Acidobacteria_Gp2			Gp2	0	0	0	0	0	0	1	1	0	0	1	0	3
Cluster10566	Acidobact	Acidobacteria_Gp3				0	0	0	1	1	0	0	0	0	0	0	1	3
Cluster10579	Acidobact	Acidobacteria_Gp1			Gp1	0	0	0	0	1	0	1	1	0	0	0	0	3
Cluster10601	Acidobacteria					1	0	0	0	0	2	0	0	0	0	0	0	3
Cluster10773;size=4						0	0	0	0	0	1	2	0	0	0	0	0	3
Cluster10809	Acidobact	Acidobacteria_Gp3				0	0	0	1	0	0	0	1	0	0	1	0	3
Cluster10847	Acidobact	Acidobacteria_Gp1			Gp1	0	0	0	1	0	0	1	0	0	1	0	0	3
Cluster10848	Proteobac	Deltap	Myxococcales			0	0	1	0	0	0	1	0	0	0	0	1	3
Cluster10960	Acidobact	Acidobacteria_Gp3			Gp3	0	0	0	0	1	1	0	0	1	0	0	0	3
Cluster11133	Acidobact	Acidobacteria_Gp3			Gp3	0	0	0	1	0	0	0	0	0	2	0	0	3
Cluster11268	Proteobac	Deltaproteobacteria				0	1	0	0	0	1	1	0	0	0	0	0	3
Cluster11295	Gemmatir	Gemm	Gemi	Gemma	Gemmatimona	0	0	0	0	2	0	0	0	0	1	0	0	3
Cluster11467	Proteobac	Alphap	Rhizobiales			0	1	0	0	0	0	0	0	1	0	1	0	3
Cluster11654	Proteobac	Deltap	Myxococcales			0	0	0	0	0	0	0	1	2	0	0	0	3
Cluster11670	Acidobact	Acidobacteria_Gp2			Gp2	0	0	0	0	0	0	2	1	0	0	0	0	3
Cluster11682	Acidobact	Acidobacteria_Gp10			Gp10	0	0	0	1	0	0	0	0	1	0	1	0	3
Cluster11805;size=14						0	1	0	0	0	0	2	0	0	0	0	0	3
Cluster11848;size=10						0	1	0	0	0	0	0	0	0	0	2	0	3
Cluster11905	Acidobact	Acidobacteria_Gp1			Acidobacteriur	0	0	1	0	0	0	1	1	0	0	0	0	3
Cluster11941	Acidobact	Acidobacteria_Gp1				0	0	1	0	0	0	1	0	1	0	0	0	3
Cluster11958	Acidobact	Acidobacteria_Gp1			Granulicella	0	0	0	0	0	0	0	0	0	1	1	1	3
Cluster12057	Armatimonadetes				Armatimonade	0	0	0	0	1	0	0	0	1	1	0	0	3

Cluster12149	Acidobact	Acidobacteria_Gp1				0	1	1	0	0	0	0	0	1	0	0	0	3
Cluster12182	Acidobact	Acidobacteria_Gp1			Gp1	0	0	1	0	0	1	0	0	0	0	1	0	3
Cluster12206	Acidobact	Acidobacteria_Gp3			Gp3	0	0	0	0	0	2	0	0	1	0	0	0	3
Cluster12487	Gemmatir	Gemm	Gemm	Gemma	Gemmatimona	0	0	0	0	0	1	0	0	1	0	1	0	3
Cluster12499	Proteobac	Betapr	Burkh	Burkhol	Burkholderia	0	0	0	0	0	0	1	0	0	2	0	0	3
Cluster12607	Acidobact	Acidobacteria_Gp1			Gp1	0	0	2	0	1	0	0	0	0	0	0	0	3
Cluster12640	Acidobacteria					0	0	0	2	0	0	0	0	0	0	1	0	3
Cluster12676	Acidobact	Acidobacteria_Gp1			Gp1	0	0	0	0	0	0	0	1	0	0	0	2	3
Cluster12902	Actinobac	Actinob	Acidimicrobiales			0	0	0	0	1	0	0	0	0	1	0	1	3
Cluster13040	Acidobact	Acidobacteria_Gp3			Bryobacter	0	0	1	1	0	0	1	0	0	0	0	0	3
Cluster13117	Proteobac	Gamm	Xanth	Sinobac	Steroidobacter	0	0	1	2	0	0	0	0	0	0	0	0	3
Cluster13182;size=3						0	0	2	0	0	0	0	0	0	0	1	0	3
Cluster13424	Acidobact	Acidobacteria_Gp1			Gp1	0	0	0	1	0	0	1	0	0	0	0	1	3
Cluster13686	Proteobac	Gamm	Xanth	Sinobac	Nevskia	0	0	1	0	1	0	0	0	0	0	0	1	3
Cluster13747	Actinobac	Actinob	Soliru	Conexib	Conexibacter	0	0	0	0	0	0	0	0	0	1	2	0	3
Cluster13851	Acidobact	Acidobacteria_Gp2			Gp2	0	0	1	0	1	0	0	0	0	0	1	0	3
Cluster13876	Proteobac	Deltap	Myxococcales			0	0	0	0	0	2	0	0	0	0	0	1	3
Cluster13950	Acidobacteria					0	0	1	0	0	0	1	0	0	0	1	0	3
Cluster14172;size=9						0	0	0	0	0	0	0	0	0	3	0	0	3
Cluster14198	Acidobact	Acidobacteria_Gp2			Gp2	0	0	1	1	0	0	0	0	0	0	1	0	3
Cluster14212	Acidobact	Acidobacteria_Gp1			Gp1	2	0	1	0	0	0	0	0	0	0	0	0	3
Cluster14411	Acidobact	Acidobacteria_Gp3			Gp3	0	1	0	0	0	1	1	0	0	0	0	0	3
Cluster14424	Acidobact	Acidobacteria_Gp1				1	0	0	0	2	0	0	0	0	0	0	0	3
Cluster14514	Acidobact	Acidobacteria_Gp1				0	0	1	1	0	1	0	0	0	0	0	0	3
Cluster14524	Acidobact	Acidobacteria_Gp2			Gp2	0	1	0	1	1	0	0	0	0	0	0	0	3
Cluster14554;size=15						0	0	0	1	2	0	0	0	0	0	0	0	3
Cluster14580	Acidobact	Acidobacteria_Gp1			Gp1	0	1	0	1	0	0	0	0	0	0	1	0	3
Cluster14595	Acidobact	Acidobacteria_Gp2			Gp2	0	0	0	0	0	0	0	2	1	0	0	0	3
Cluster14628	Acidobact	Acidobacteria_Gp2			Gp2	0	0	0	0	0	0	0	1	1	0	0	1	3

Cluster14654	Acidobact	Acidobacteria_Gp2			Gp2	0	0	0	1	0	0	0	0	1	0	1	0	3
Cluster14755	Acidobact	Acidobacteria_Gp1			Gp1	0	0	0	0	2	1	0	0	0	0	0	0	3
Cluster14772	Actinobac	Actinol	Actinomycetales			0	0	0	0	0	0	0	2	1	0	0	0	3
Cluster14848	Acidobact	Acidobacteria_Gp1			Gp1	0	1	0	0	0	0	0	2	0	0	0	0	3
Cluster14900	Acidobacteria					0	0	1	0	1	0	0	0	0	0	0	1	3
Cluster14911	Acidobact	Acidobacteria_Gp2			Gp2	0	0	0	0	0	0	1	2	0	0	0	0	3
Cluster14927	Proteobac	Alphap	Rhizobiales			0	0	0	0	0	0	2	0	0	0	0	1	3
Cluster15004	Chloroflex	Ktedor	Ktedo	Ktedono	Ktedonobacter	0	0	0	0	0	0	0	2	0	0	1	0	3
Cluster15030	Proteobac	Deltap	Bdell	Bdellovi	Vampirovibrio	0	0	0	0	0	0	0	0	0	3	0	0	3
Cluster15139	Acidobact	Acidobacteria_Gp2			Gp2	0	0	0	0	0	0	0	0	3	0	0	0	3
Cluster15178	Acidobact	Acidobacteria_Gp2			Gp2	0	0	1	1	0	0	0	1	0	0	0	0	3
Cluster15197;size=15						0	0	0	0	1	0	0	0	1	0	0	1	3
Cluster15208	Acidobact	Acidobacteria_Gp1			Gp1	1	0	0	0	0	1	0	0	0	0	0	1	3
Cluster15210	Acidobact	Acidobacteria_Gp1				0	0	0	1	0	0	0	0	0	0	0	2	3
Cluster15299	Acidobact	Acidobacteria_Gp1				0	2	0	0	0	0	0	1	0	0	0	0	3
Cluster15544	Firmicutes	Bacilli	Bacill	Paeniba	Paenibacillus	0	3	0	0	0	0	0	0	0	0	0	0	3
Cluster15575	Proteobac	Alphap	Rhizo	Bradyrh	Bradyrhizobium	0	1	0	0	0	1	1	0	0	0	0	0	3
Cluster15721	Acidobact	Acidobacteria_Gp1			Granulicella	0	0	0	2	1	0	0	0	0	0	0	0	3
Cluster15791;size=10						0	1	0	0	0	0	1	1	0	0	0	0	3
Cluster15861	Acidobact	Acidobacteria_Gp5			Gp5	0	0	0	0	2	0	0	0	0	1	0	0	3
Cluster15984	Acidobact	Acidobacteria_Gp1			Gp1	0	0	0	0	1	0	0	0	2	0	0	0	3
Cluster16030	Gemmatir	Gemm	Gemi	Gemma	Gemmatimona	0	0	0	1	0	0	0	0	0	1	1	0	3
Cluster16133	Proteobac	Betapr	Burkl	Oxalobacteraceae		0	0	2	0	1	0	0	0	0	0	0	0	3
Cluster16247	Acidobact	Acidobacteria_Gp1				0	0	1	2	0	0	0	0	0	0	0	0	3
Cluster16321	Acidobact	Acidobacteria_Gp1			Granulicella	0	0	2	0	0	0	0	0	0	1	0	0	3
Cluster16416	Acidobact	Acidobacteria_Gp1				0	0	1	1	0	0	0	0	0	0	1	0	3
Cluster16571	Proteobacteria					2	0	0	0	1	0	0	0	0	0	0	0	3
Cluster16671	Proteobac	Alphaproteobacteria				0	0	2	1	0	0	0	0	0	0	0	0	3
Cluster16901	Chlamydia	Chlamy	Chlar	Parachlamydiaceae		0	0	0	1	0	0	1	0	0	0	1	0	3

Cluster17000;size=7						1	0	0	0	0	0	0	1	0	0	1	0	3
Cluster17023;size=7						1	0	0	0	0	0	0	1	0	0	1	0	3
Cluster17088	Proteobac	Alphap	Rhizobiales			1	0	0	2	0	0	0	0	0	0	0	0	3
Cluster17138	Acidobact	Acidobacteria_Gp1				0	0	0	1	0	1	0	0	0	0	0	1	3
Cluster17175	Acidobact	Acidobacteria_Gp1			Gp1	0	0	1	0	0	0	0	0	0	2	0	0	3
Cluster17471	Acidobact	Acidobacteria_Gp1			Gp1	0	0	0	0	0	0	0	0	0	3	0	0	3
Cluster17492	Acidobact	Acidobacteria_Gp1				0	1	0	0	0	0	1	0	0	0	1	0	3
Cluster17516	Proteobac	Deltap	Bdell	Bdellovi	Vampirovibrio	0	1	0	0	0	0	0	0	1	0	0	1	3
Cluster17680	Actinobac	Actinol	Solirı	Conexib	Conexibacter	0	0	0	0	1	0	1	0	0	0	0	1	3
Cluster17721	Acidobact	Acidobacteria_Gp1			Gp1	0	0	1	1	0	0	0	0	0	0	1	0	3
Cluster17731;size=5						0	0	0	0	0	1	0	2	0	0	0	0	3
Cluster17817	Proteobac	Gammaproteobacteria				0	0	0	1	1	0	0	1	0	0	0	0	3
Cluster18041	Proteobac	Gamm	Xantl	Sinobac	Steroidobacter	0	0	0	0	0	0	0	0	0	2	1	0	3
Cluster18047;size=13						0	0	1	0	1	0	0	0	0	1	0	0	3
Cluster18099	Proteobac	Gamm	Xantl	Sinobac	Steroidobacter	0	0	0	0	0	0	1	0	1	0	0	1	3
Cluster18118	OP11				OP11_genera_	1	1	0	0	0	0	1	0	0	0	0	0	3
Cluster18122	Verrucom	Opituta	Opitu	Opituta	Opitutus	0	0	1	0	0	1	0	0	0	0	1	0	3
Cluster18208	Proteobac	Alphap	Rhizo	Bradyrhizobiaceae		0	0	1	1	0	0	0	0	0	0	1	0	3
Cluster18247	Acidobact	Acidobacteria_Gp2			Gp2	0	0	0	0	0	0	1	1	0	0	1	0	3
Cluster18383	Acidobact	Acidobacteria_Gp2			Gp2	0	0	1	1	0	0	0	0	0	0	1	0	3
Cluster18445	Actinobac	Actinol	Solirı	Conexib	Conexibacter	0	1	0	0	0	0	0	0	0	0	2	0	3
Cluster18482	Proteobac	Alphaproteobacteria				0	0	0	0	0	0	0	1	0	1	1	0	3
Cluster18535	Acidobact	Acidobacteria_Gp3			Gp3	0	0	0	0	2	0	0	0	1	0	0	0	3
Cluster18565	Acidobact	Acidobacteria_Gp2			Gp2	0	0	0	1	2	0	0	0	0	0	0	0	3
Cluster18583	Acidobact	Acidobacteria_Gp1				0	0	1	1	1	0	0	0	0	0	0	0	3
Cluster18628	Acidobact	Acidobacteria_Gp2			Gp2	0	0	0	0	1	0	0	2	0	0	0	0	3
Cluster18667	Proteobac	Gammaproteobacteria				1	0	0	0	0	0	1	0	0	0	0	1	3
Cluster18757	Proteobac	Alphap	Rhizobiales			0	1	0	0	0	0	0	0	0	0	1	1	3
Cluster18769	Acidobact	Acidobacteria_Gp1			Granulicella	0	0	0	0	1	1	0	0	0	0	0	1	3

Cluster18780	Acidobact	Acidobacteria_Gp1			Gp1	0	0	0	0	1	1	1	0	0	0	0	0	3
Cluster18820	Acidobacteria					0	0	0	1	0	0	1	0	1	0	0	0	3
Cluster18848	Acidobact	Acidobacteria_Gp3				0	1	0	0	0	0	0	0	0	1	0	1	3
Cluster18854	Acidobact	Acidobacteria_Gp3			Gp3	0	0	0	0	0	2	0	0	0	1	0	0	3
Cluster19002	Proteobac	Alphap	Rhod	Acetobacteraceae		0	0	0	0	0	0	0	0	0	2	1	0	3
Cluster19095	Acidobact	Acidobacteria_Gp3			Gp3	0	0	0	2	0	0	0	0	0	0	0	1	3
Cluster19128	Acidobact	Acidobacteria_Gp3			Gp3	0	0	0	0	0	0	1	0	0	2	0	0	3
Cluster19146;size=10						1	0	0	1	0	0	1	0	0	0	0	0	3
Cluster19260	Acidobact	Acidobacteria_Gp2			Gp2	0	0	0	0	0	0	1	1	0	0	0	1	3
Cluster19280	Acidobact	Acidobacteria_Gp2			Gp2	0	0	0	0	0	0	0	0	1	0	2	0	3
Cluster19384	Acidobact	Acidobacteria_Gp3			Bryobacter	0	0	0	0	0	0	0	1	1	0	1	0	3
Cluster19495	Proteobac	Alphap	Caulo	Caulobа	Phenylobacter	1	0	0	0	1	0	0	0	0	1	0	0	3
Cluster19568	Acidobacteria					0	1	0	0	0	1	1	0	0	0	0	0	3
Cluster19750	Proteobac	Alphap	Rhod	Acetobacteraceae		0	0	2	0	0	0	0	0	0	1	0	0	3
Cluster19766	Acidobact	Acidobacteria_Gp3				1	0	0	1	0	1	0	0	0	0	0	0	3
Cluster19831	Acidobact	Acidobacteria_Gp1				0	0	0	2	0	0	0	0	0	0	0	1	3
Cluster19859	Actinobac	Actino	Actinomycetales			0	0	0	0	2	1	0	0	0	0	0	0	3
Cluster19925	Acidobact	Acidobacteria_Gp3			Gp3	0	0	1	1	1	0	0	0	0	0	0	0	3
Cluster19977	Proteobac	Gamm	Legio	Legione	Legionella	1	0	0	1	0	0	1	0	0	0	0	0	3
Cluster20054	Acidobact	Acidobacteria_Gp3			Gp3	2	0	1	0	0	0	0	0	0	0	0	0	3
Cluster20118	Proteobac	Alphap	Rhod	Acetoba	Acidisphaera	0	0	1	1	1	0	0	0	0	0	0	0	3
Cluster20177	Actinobac	Actino	Acidi	Acidimi	Aciditerrimona	0	0	1	0	0	1	1	0	0	0	0	0	3
Cluster20361	Proteobac	Gammaproteobacteria				0	1	0	0	0	2	0	0	0	0	0	0	3
Cluster20368	Proteobac	Alphap	Rhod	Acetoba	Acidisphaera	1	0	1	1	0	0	0	0	0	0	0	0	3
Cluster20374	Acidobact	Acidobacteria_Gp1			Acidobacteriu	0	0	1	1	0	0	0	0	0	1	0	0	3
Cluster20382	Proteobac	Betapr	Burkl	Oxalobacteraceae		0	0	0	0	1	1	1	0	0	0	0	0	3
Cluster20440	Bacteroid	Sphing	Sphin	Chitinophagaceae		0	0	0	0	2	1	0	0	0	0	0	0	3
Cluster20445	Acidobact	Acidobacteria_Gp1				0	0	2	1	0	0	0	0	0	0	0	0	3
Cluster20493	Acidobact	Acidobacteria_Gp3			Gp3	2	0	0	0	0	0	1	0	0	0	0	0	3

Cluster20538	Proteobac	Alphap	Alphaproteob		Rhizomicrobiu	0	0	0	1	1	0	1	0	0	0	0	0	3
Cluster20580	Proteobac	Betaproteobacteria				1	0	0	1	1	0	0	0	0	0	0	0	3
Cluster20644	Acidobact	Acidobacteria_Gp6			Gp6	0	0	0	1	1	0	0	0	0	0	1	0	3
Cluster20723	Proteobac	Gamm	Xantl	Sinobac	Steroidobacter	1	0	0	0	1	1	0	0	0	0	0	0	3
Cluster20820	Proteobac	Alphaproteobacteria				0	2	0	0	1	0	0	0	0	0	0	0	3
Cluster20821	Actinobac	Actinol	Acidimicrobiales			0	0	0	0	0	0	2	0	0	0	0	1	3
Cluster20827	Acidobact	Acidobacteria_Gp3			Gp3	0	0	1	0	1	0	0	0	0	0	1	0	3
Cluster20835	Acidobact	Acidobacteria_Gp1				0	0	0	1	0	1	0	1	0	0	0	0	3
Cluster20839	Verrucom	Opituta	Opitu	Opituta	Opitutus	1	1	0	0	0	0	1	0	0	0	0	0	3
Cluster21044	Proteobac	Alphap	Caulc	Caulobacteraceae		1	0	0	1	0	0	0	0	0	1	0	0	3
Cluster21073	Actinobac	Actinobacteria				1	0	0	0	2	0	0	0	0	0	0	0	3
Cluster21087;size=13						0	0	0	0	0	0	3	0	0	0	0	0	3
Cluster21265	Acidobact	Acidobacteria_Gp2			Gp2	0	0	0	1	0	0	1	0	0	0	1	0	3
Cluster21423	Chlamydia	Chlamy	Chlar	Parachlamydiaceae		1	0	0	0	0	1	1	0	0	0	0	0	3
Cluster21629	Actinobac	Actinol	Acidimicrobiales			0	2	0	0	0	0	0	0	1	0	0	0	3
Cluster21751	Proteobac	Gammaproteobacteria				0	0	1	1	1	0	0	0	0	0	0	0	3
Cluster21762	Proteobac	Alphap	Rhizc	Xanthol	Pseudolabrys	1	0	1	1	0	0	0	0	0	0	0	0	3
Cluster21766	Verrucom	Opituta	Opitu	Opituta	Opitutus	0	0	0	1	0	0	0	0	0	2	0	0	3
Cluster21830	Actinobac	Actinol	Actinomycetales			0	0	0	0	0	0	0	0	0	2	1	0	3
Cluster21831	Acidobact	Acidobacteria_Gp1			Gp1	0	0	0	1	0	0	0	0	0	0	2	0	3
Cluster22053	Acidobact	Acidobacteria_Gp6			Gp6	0	0	0	0	0	1	0	1	0	1	0	0	3
Cluster22091	Proteobac	Alphaproteobacteria				0	0	1	2	0	0	0	0	0	0	0	0	3
Cluster22317	Acidobact	Acidobacteria_Gp1				0	1	0	0	0	0	0	1	1	0	0	0	3
Cluster22335;size=13						0	2	0	0	0	0	0	0	0	0	0	1	3
Cluster22351;size=4						0	0	1	0	0	0	1	0	0	0	0	1	3
Cluster22386	Proteobac	Gamm	Xantl	Sinobac	Steroidobacter	1	0	1	1	0	0	0	0	0	0	0	0	3
Cluster22644	Actinobac	Actinol	Soliru	Conexib	Conexibacter	1	0	1	0	0	0	1	0	0	0	0	0	3
Cluster22654	Proteobac	Gammaproteobacteria				2	0	0	0	0	0	0	0	0	0	1	0	3
Cluster22656	Proteobac	Alphap	Rhod	Acetobacteraceae		1	0	0	0	2	0	0	0	0	0	0	0	3

Cluster22725	Proteobac	Alphap	Rhod	Acetobacteraceae		0	0	0	1	2	0	0	0	0	0	0	0	3
Cluster22793	Proteobac	Alphap	Rhod	Acetobacteraceae		0	0	0	0	1	0	0	0	0	2	0	0	3
Cluster22928	Acidobact	Acidobacteria_Gp13			Gp13	1	0	0	0	0	1	0	0	1	0	0	0	3
Cluster22979	Actinobac	Actinol	Actinomycetales			0	0	2	0	0	0	0	0	0	0	1	0	3
Cluster22985	Proteobac	Alphaproteobacteria				0	0	1	0	0	2	0	0	0	0	0	0	3
Cluster23215	Proteobac	Alphap	Caulc	Caulobacteraceae		0	0	0	0	0	0	0	0	1	0	2	0	3
Cluster23308	Acidobact	Acidobacteria_Gp3			Gp3	0	0	0	0	1	2	0	0	0	0	0	0	3
Cluster23380	Bacteroid	Sphing	Sphir	Sphingc	Mucilaginibact	1	0	0	0	1	0	0	0	0	1	0	0	3
Cluster23426	Proteobac	Gamm	Pseu	Pseudoı	Pseudomonas	0	0	0	0	0	3	0	0	0	0	0	0	3
Cluster23428	Proteobac	Gamm	Xantl	Sinobac	Nevskia	0	0	0	0	0	1	2	0	0	0	0	0	3
Cluster23968	Actinobac	Actinol	Solirubrobacterales			0	0	0	0	0	0	0	1	1	1	0	0	3
Cluster24089	Actinobac	Actinol	Actin	Mycoba	Mycobacteriur	0	0	0	0	2	0	0	0	0	0	1	0	3
Cluster24097	Bacteroid	Sphing	Sphir	Sphingc	Mucilaginibact	0	0	0	0	0	0	0	3	0	0	0	0	3
Cluster24178	Proteobac	Gammaproteobacteria				0	0	0	0	1	0	0	0	0	2	0	0	3
Cluster24215	Acidobact	Acidobacteria_Gp1				0	0	0	0	2	0	0	0	0	1	0	0	3
Cluster24406	Bacteroid	Sphing	Sphir	Sphingc	Mucilaginibact	2	0	0	0	0	0	0	1	0	0	0	0	3
Cluster24573	Proteobac	Alphap	Rhizobiales			0	0	1	0	2	0	0	0	0	0	0	0	3
Cluster24599	Proteobac	Gamm	Pseu	Pseudoı	Pseudomonas	0	0	1	0	0	0	0	1	0	0	1	0	3
Cluster24678;size=5						1	0	0	0	0	0	1	1	0	0	0	0	3
Cluster24754	Proteobac	Alphap	Rhizobiales			0	0	1	1	1	0	0	0	0	0	0	0	3
Cluster24817	Proteobac	Gamm	Pseu	Pseudoı	Pseudomonas	0	0	1	0	1	0	1	0	0	0	0	0	3
Cluster25023	Actinobac	Actinobacteria				0	0	2	0	0	0	0	0	0	1	0	0	3
Cluster25050	Bacteroid	Sphing	Sphir	Chitinophagaceae		0	0	1	0	0	0	1	0	0	1	0	0	3
Cluster25053	Actinobac	Actinol	Acidi	Acidimi	Aciditerrimona	0	0	0	1	0	0	1	0	0	1	0	0	3
Cluster25118	Actinobac	Actinol	Actinomycetales			0	0	0	0	0	1	1	1	0	0	0	0	3
Cluster25229	Acidobact	Acidobacteria_Gp3			Gp3	0	0	1	0	1	0	0	1	0	0	0	0	3
Cluster25326	Proteobac	Alphaproteobacteria				1	0	1	0	1	0	0	0	0	0	0	0	3
Cluster25399	Bacteroid	Sphing	Sphir	Sphingobacteriaceae		0	0	2	0	0	0	0	0	0	0	1	0	3
Cluster25420	Proteobac	Gamm	Enter	Enterobacteriaceae		1	0	0	1	0	0	0	1	0	0	0	0	3

Cluster25471	Actinobac	Actinol	Acidimicrobiales			0	0	1	0	0	0	0	1	0	0	1	0	3
Cluster25694	Actinobac	Actinol	Actin	Catenul	Catenulispora	0	0	0	0	0	0	0	1	0	0	1	1	3
Cluster25729	Actinobac	Actinol	Actinomycetales			1	0	0	0	0	0	0	0	1	0	1	0	3
Cluster26235	Proteobac	Alphaproteobacteria				0	0	1	1	0	0	0	0	0	0	1	0	3
Cluster26334	Proteobac	Deltap	Bdell	Bdellovi	Bdellovibrio	1	0	0	1	0	0	0	0	0	1	0	0	3
Cluster26710	Bacteroid	Sphing	Sphir	Chitinop	Sediminibacter	1	0	1	0	0	0	0	0	0	0	1	0	3
Cluster26744	Bacteroid	Sphing	Sphir	Chitinop	Ferruginibacte	2	0	0	0	0	0	1	0	0	0	0	0	3
Cluster26846;size=16						0	0	1	0	0	0	0	1	0	0	1	0	3
Cluster26913	Acidobacteria					0	1	0	0	0	0	1	0	0	0	0	1	3
Cluster26945	Bacteroid	Sphing	Sphir	Chitinophagaceae		0	0	1	0	0	0	0	0	0	1	0	1	3
Cluster27243	Bacteroid	Sphing	Sphir	Chitinophagaceae		0	0	3	0	0	0	0	0	0	0	0	0	3
Cluster27396	Bacteroid	Sphing	Sphir	Chitinophagaceae		0	0	0	1	0	1	0	1	0	0	0	0	3
Cluster27402;size=5						0	0	0	0	0	0	1	0	0	0	2	0	3
Cluster27431	Actinobac	Actinol	Acidi	Acidimi	Aciditerrimona	0	0	2	0	0	0	1	0	0	0	0	0	3
Cluster27747	Bacteroid	Sphing	Sphir	Chitinophagaceae		0	0	0	1	0	1	0	1	0	0	0	0	3
Cluster27879	Bacteroid	Sphing	Sphingobacteriales			0	0	0	0	0	0	1	0	0	2	0	0	3
Cluster28109;size=10						0	0	0	0	1	0	0	1	0	0	0	1	3
Cluster28265	Bacteroid	Sphing	Sphir	Sphingo	Mucilaginibact	0	0	0	1	0	1	0	1	0	0	0	0	3
Cluster28266	Actinobac	Actinol	Actinomycetales			0	0	1	0	0	0	0	0	0	2	0	0	3
Cluster28826	Actinobac	Actinol	Acidi	Acidimi	Aciditerrimona	0	0	0	0	2	1	0	0	0	0	0	0	3
Cluster29032	Bacteroid	Sphing	Sphir	Sphingo	Mucilaginibact	0	1	0	0	0	0	0	0	1	0	0	1	3
Cluster29183	Actinobac	Actinobacteria				0	0	0	1	1	0	0	1	0	0	0	0	3
Cluster29219	Bacteroidetes					0	0	1	0	0	0	0	2	0	0	0	0	3
Cluster29259	Actinobac	Actinol	Actinomycetales			0	1	0	0	0	0	1	1	0	0	0	0	3
Cluster29290	Bacteroid	Sphing	Sphir	Sphingo	Mucilaginibact	1	0	1	0	0	0	0	0	1	0	0	0	3
Cluster29324	Bacteroid	Sphing	Sphir	Sphingo	Mucilaginibact	0	1	0	1	0	1	0	0	0	0	0	0	3
Cluster29499	Bacteroid	Sphing	Sphir	Sphingo	Mucilaginibact	0	0	0	0	0	0	0	0	0	1	1	1	3
Cluster17;siz	Acidobact	Acidobacteria_Gp10			Gp10	0	0	0	0	0	0	0	1	0	0	0	1	2
Cluster22;siz	Acidobact	Acidobacteria_Gp1				0	1	0	0	0	0	0	1	0	0	0	0	2

Cluster59;siz	Proteobac	Gamm	Xantl	Sinobacteraceae		1	0	0	0	0	0	0	0	0	0	0	1	2
Cluster71;siz	Proteobac	Deltap	Myxc	Cystobacteraceae		0	0	0	1	0	0	0	0	0	0	0	1	2
Cluster76;siz	Proteobac	Deltaproteobacteria				0	1	1	0	0	0	0	0	0	0	0	0	2
Cluster107;si	Proteobac	Alphap	Rhizobiales			0	1	0	0	0	0	0	1	0	0	0	0	2
Cluster110;si	Proteobac	Deltap	Bdell	Bdellovi	Vampirovibrio	0	1	0	0	0	0	1	0	0	0	0	0	2
Cluster117;si	Planctomy	Plancto	Planc	Plancto	Planctomyces	0	0	0	0	1	0	1	0	0	0	0	0	2
Cluster145;si	Proteobacteria					0	0	0	0	0	0	0	1	0	0	0	1	2
Cluster152;si	Acidobact	Acidobacteria_Gp1				0	0	1	1	0	0	0	0	0	0	0	0	2
Cluster175;si	Acidobact	Acidobacteria_Gp2			Gp2	0	0	0	1	0	0	0	0	0	0	1	0	2
Cluster195;size=21						0	0	0	0	1	0	0	0	1	0	0	0	2
Cluster201;si	Acidobact	Acidobacteria_Gp3			Gp3	0	0	0	0	0	1	0	1	0	0	0	0	2
Cluster213;size=3						0	0	0	1	0	0	0	1	0	0	0	0	2
Cluster230;si	Acidobact	Acidobacteria_Gp2			Gp2	0	0	0	0	1	0	1	0	0	0	0	0	2
Cluster256;si	Acidobact	Acidobacteria_Gp1				0	0	0	0	0	0	0	2	0	0	0	0	2
Cluster275;si	Acidobact	Acidobacteria_Gp2			Gp2	0	0	1	0	0	0	0	0	1	0	0	0	2
Cluster279;si	Acidobact	Acidobacteria_Gp3			Gp3	1	0	0	0	1	0	0	0	0	0	0	0	2
Cluster285;si	Proteobac	Alphap	Rhodospirillales			0	0	0	0	1	0	0	0	0	0	1	0	2
Cluster320;si	Acidobact	Acidobacteria_Gp1			Granulicella	0	0	0	0	0	0	0	0	0	1	1	0	2
Cluster331;si	Acidobact	Acidobacteria_Gp2			Gp2	0	0	0	1	0	1	0	0	0	0	0	0	2
Cluster357;si	Acidobact	Acidobacteria_Gp2			Gp2	0	0	0	0	0	0	0	1	0	0	0	1	2
Cluster360;si	Proteobac	Betaproteobacteria				0	0	0	0	0	0	0	0	0	2	0	0	2
Cluster369;si	Acidobact	Acidobacteria_Gp3			Bryobacter	0	0	1	0	0	0	0	0	0	1	0	0	2
Cluster370;si	TM7				TM7_genera_i	0	0	1	0	0	1	0	0	0	0	0	0	2
Cluster372;si	Proteobac	Gammaproteobacteria				0	0	1	0	0	0	0	0	0	1	0	0	2
Cluster378;size=14						0	1	1	0	0	0	0	0	0	0	0	0	2
Cluster382;si	Acidobact	Acidobacteria_Gp1				0	0	0	0	1	0	0	0	0	1	0	0	2
Cluster396;si	Acidobact	Acidobacteria_Gp1			Gp1	0	0	0	0	0	0	0	1	0	1	0	0	2
Cluster398;si	Acidobacteria					0	0	1	0	1	0	0	0	0	0	0	0	2
Cluster413;si	Acidobact	Acidobacteria_Gp2			Gp2	0	0	0	0	0	0	0	0	0	1	1	0	2

Cluster418;si	Acidobact	Acidobacteria_Gp3				0	0	0	0	0	1	0	0	0	1	0	0	2
Cluster450;si	Acidobact	Acidobacteria_Gp3				0	1	0	0	0	0	0	0	1	0	0	0	2
Cluster457;si	Verrucom	Opituta	Opitu	Opituta	Opitutus	1	0	0	0	0	0	0	0	0	1	0	0	2
Cluster489;si	Acidobact	Acidobacteria_Gp1				0	1	1	0	0	0	0	0	0	0	0	0	2
Cluster528;si	Acidobact	Acidobacteria_Gp1			Gp1	0	0	2	0	0	0	0	0	0	0	0	0	2
Cluster529;si	Proteobac	Betaproteobacteria				0	2	0	0	0	0	0	0	0	0	0	0	2
Cluster546;si	Acidobact	Acidobacteria_Gp1			Gp1	0	1	0	0	1	0	0	0	0	0	0	0	2
Cluster557;si	Proteobac	Deltap	Myxc	Cystoba	Anaeromyxoba	1	0	1	0	0	0	0	0	0	0	0	0	2
Cluster559;si	Acidobact	Acidobacteria_Gp3			Gp3	0	1	0	0	1	0	0	0	0	0	0	0	2
Cluster572;si	Acidobact	Acidobacteria_Gp10			Gp10	0	0	0	1	0	0	0	0	1	0	0	0	2
Cluster578;si	Acidobact	Acidobacteria_Gp2			Gp2	1	0	0	1	0	0	0	0	0	0	0	0	2
Cluster594;si	Proteobac	Alphap	Alphaproteob		Rhizomicrobiu	0	0	0	1	0	0	1	0	0	0	0	0	2
Cluster595;si	Acidobact	Acidobacteria_Gp1				0	0	0	0	0	0	0	1	1	0	0	0	2
Cluster602;si	Acidobact	Acidobacteria_Gp1				1	0	0	0	0	0	0	0	1	0	0	0	2
Cluster603;si	Acidobact	Acidobacteria_Gp1				1	0	0	1	0	0	0	0	0	0	0	0	2
Cluster604;size=2						1	0	0	0	0	1	0	0	0	0	0	0	2
Cluster626;si	Acidobact	Acidobacteria_Gp1				0	0	0	0	0	0	0	0	0	1	1	0	2
Cluster634;si	Proteobac	Deltaproteobacteria				2	0	0	0	0	0	0	0	0	0	0	0	2
Cluster654;si	Acidobact	Acidobacteria_Gp1			Gp1	0	0	0	0	0	0	0	0	0	0	2	0	2
Cluster671;size=7						0	0	0	0	1	0	0	0	0	1	0	0	2
Cluster672;si	Proteobac	Alphap	Rhizobiales			0	0	0	0	2	0	0	0	0	0	0	0	2
Cluster673;si	Acidobact	Acidobacteria_Gp1			Gp1	1	0	0	1	0	0	0	0	0	0	0	0	2
Cluster686;si	Proteobac	Gamm	Legio	Coxiella	Aquicella	0	0	1	0	0	0	0	0	1	0	0	0	2
Cluster687;si	Acidobact	Acidobacteria_Gp3				1	0	0	0	0	0	0	0	0	0	1	0	2
Cluster692;si	Acidobact	Acidobacteria_Gp1				0	0	0	0	1	0	0	0	0	0	0	1	2
Cluster715;si	Proteobac	Alphap	Rhod	Acetobacteraceae		0	0	0	1	0	0	0	0	0	0	1	0	2
Cluster732;si	Acidobact	Acidobacteria_Gp3			Gp3	0	0	0	0	0	1	0	0	1	0	0	0	2
Cluster733;si	Acidobact	Acidobacteria_Gp1			Terriglobus	0	0	2	0	0	0	0	0	0	0	0	0	2
Cluster738;size=21						0	0	0	0	0	0	2	0	0	0	0	0	2

Cluster744;si	Proteobac	Alphap	Rhod	Acetobacteraceae		0	0	0	0	0	0	0	0	0	1	1	0	2
Cluster762;si	Acidobact	Acidobacteria_Gp2			Gp2	0	2	0	0	0	0	0	0	0	0	0	0	2
Cluster780;si	Bacteroid	Sphing	Sphir	Chitinophagaceae		0	0	0	0	1	0	0	0	0	0	0	1	2
Cluster781;si	Proteobac	Alphap	Rhizobiales			0	1	0	1	0	0	0	0	0	0	0	0	2
Cluster815;size=6						0	0	0	0	1	0	0	0	0	0	1	0	2
Cluster825;si	Proteobac	Deltap	Myxc	Polyangiaceae		0	0	0	0	1	0	0	1	0	0	0	0	2
Cluster827;si	Acidobact	Acidobacteria_Gp2			Gp2	0	0	1	0	0	0	1	0	0	0	0	0	2
Cluster837;si	Acidobact	Acidobacteria_Gp1			Gp1	1	0	0	0	0	0	0	0	0	1	0	0	2
Cluster841;si	Acidobact	Acidobacteria_Gp3			Gp3	0	0	0	0	0	1	0	0	0	0	0	1	2
Cluster854;si	Proteobac	Alphap	Rhizobiales			0	0	0	0	0	0	0	0	0	1	1	0	2
Cluster861;si	Acidobact	Acidobacteria_Gp3				0	0	0	0	1	0	0	0	1	0	0	0	2
Cluster874;si	Acidobact	Acidobacteria_Gp1				1	0	0	0	0	0	0	0	0	0	1	0	2
Cluster879;si	Proteobac	Deltaproteobacteria				0	2	0	0	0	0	0	0	0	0	0	0	2
Cluster884;si	Actinobac	Actinobacteria				0	0	0	1	0	0	0	0	0	0	0	1	2
Cluster898;si	Acidobact	Acidobacteria_Gp2			Gp2	0	0	0	0	0	0	0	0	0	0	1	1	2
Cluster925;si	Actinobac	Actinol	Actin	Nocardiaceae		0	0	0	1	0	0	1	0	0	0	0	0	2
Cluster975;si	Acidobact	Acidobacteria_Gp2			Gp2	1	0	0	0	1	0	0	0	0	0	0	0	2
Cluster978;si	Proteobac	Alphap	Rhizobiales			0	0	0	0	0	0	1	0	0	0	0	1	2
Cluster979;si	Acidobact	Acidobacteria_Gp1				0	0	0	0	0	0	0	2	0	0	0	0	2
Cluster991;size=18						0	1	0	0	0	0	1	0	0	0	0	0	2
Cluster997;si	Acidobact	Acidobacteria_Gp3				1	0	0	0	1	0	0	0	0	0	0	0	2
Cluster1006;:	Acidobact	Acidobacteria_Gp2			Gp2	0	0	1	1	0	0	0	0	0	0	0	0	2
Cluster1012;:	Acidobact	Acidobacteria_Gp3			Gp3	0	0	0	0	2	0	0	0	0	0	0	0	2
Cluster1020;:	Acidobact	Acidobacteria_Gp1			Acidobacteriur	1	0	0	0	0	0	0	0	0	0	0	1	2
Cluster1027;:	Proteobac	Betaproteobacteria				0	0	1	0	0	0	0	0	0	0	1	0	2
Cluster1029;:	Acidobact	Acidobacteria_Gp2			Gp2	1	0	0	0	0	1	0	0	0	0	0	0	2
Cluster1031;:	Proteobac	Alphap	Rhizobiales			0	0	0	0	1	1	0	0	0	0	0	0	2
Cluster1034;:	Proteobac	Alphap	Rhod	Acetobacteraceae		1	0	0	0	0	1	0	0	0	0	0	0	2
Cluster1037;:	Acidobacteria					1	0	0	1	0	0	0	0	0	0	0	0	2

Cluster1041;	Acidobact	Acidobacteria_Gp1			Gp1	1	0	0	0	0	0	0	0	0	1	0	0	2
Cluster1047;	Actinobac	Actinob	Actin	Streptomycetaceae		0	0	0	0	1	0	0	0	0	0	1	0	2
Cluster1048;size=14						0	0	0	0	0	0	0	0	1	1	0	0	2
Cluster1052;	Proteobac	Deltap	Bdell	Bdellovi	Vampirovibrio	1	0	0	0	1	0	0	0	0	0	0	0	2
Cluster1066;	Proteobac	Gammaproteobacteria				0	0	1	0	0	0	0	0	0	1	0	0	2
Cluster1080;	Chloroflex	Ktedor	Ktedonobacterales			0	0	1	0	0	0	0	0	0	0	1	0	2
Cluster1084;	Proteobac	Alphap	Rhod	Acetobacteraceae		0	0	1	0	0	0	0	0	0	1	0	0	2
Cluster1086;	Acidobact	Acidobacteria_Gp1			Gp1	0	0	0	0	0	0	0	0	0	2	0	0	2
Cluster1100;	Proteobac	Betapr	Burkholderiales			1	0	0	0	0	0	0	0	1	0	0	0	2
Cluster1104;	Proteobac	Alphap	Rhodospirillales			0	1	0	0	0	0	0	0	1	0	0	0	2
Cluster1127;	Acidobact	Acidobacteria_Gp1				0	0	0	0	0	0	0	0	0	2	0	0	2
Cluster1128;	Acidobact	Acidobacteria_Gp2			Gp2	0	0	2	0	0	0	0	0	0	0	0	0	2
Cluster1133;	Acidobact	Acidobacteria_Gp2			Gp2	0	0	0	1	0	0	0	0	0	0	0	1	2
Cluster1136;	Acidobact	Acidobacteria_Gp2			Gp2	0	0	1	0	0	0	0	1	0	0	0	0	2
Cluster1139;	Acidobact	Acidobacteria_Gp3			Gp3	0	0	1	0	0	0	0	0	0	0	1	0	2
Cluster1148;	Acidobact	Acidobacteria_Gp3				0	0	0	0	0	0	1	0	1	0	0	0	2
Cluster1183;	Actinobac	Actinob	Solirubrobacterales			0	0	0	0	0	0	0	1	0	1	0	0	2
Cluster1210;	Elusimicro	Elusim	Elusi	Elusimic	Elusimicrobiu	0	1	0	0	0	0	0	0	0	0	1	0	2
Cluster1222;	Acidobact	Acidobacteria_Gp3			Gp3	0	0	0	0	0	0	0	1	0	0	1	0	2
Cluster1225;	Proteobac	Alphap	Rhodospirillales			0	0	0	0	1	0	0	0	0	0	0	1	2
Cluster1226;	Proteobac	Deltap	Myxc	Kofleria	Kofleria	1	0	0	0	0	0	0	0	0	1	0	0	2
Cluster1232;	Acidobact	Acidobacteria_Gp2			Gp2	0	2	0	0	0	0	0	0	0	0	0	0	2
Cluster1236;	Acidobact	Acidobacteria_Gp3			Bryobacter	0	1	0	0	0	0	0	0	0	1	0	0	2
Cluster1239;	Acidobact	Acidobacteria_Gp1			Gp1	0	0	0	0	0	0	0	0	1	1	0	0	2
Cluster1261;	Acidobact	Acidobacteria_Gp3				0	0	0	0	0	0	0	0	2	0	0	0	2
Cluster1267;	Acidobacteria					1	0	0	0	0	0	0	0	0	0	1	0	2
Cluster1278;size=4						0	0	0	0	0	1	1	0	0	0	0	0	2
Cluster1280;	Acidobact	Acidobacteria_Gp3			Gp3	0	0	0	1	0	0	0	0	0	0	1	0	2
Cluster1281;	Proteobac	Alphap	Rhod	Acetobacteraceae		1	0	0	1	0	0	0	0	0	0	0	0	2

Cluster1289;	Acidobact	Acidobacteria_Gp1			Edaphobacter	0	0	2	0	0	0	0	0	0	0	0	0	2
Cluster1313;	Proteobac	Deltaproteobacteria				0	0	0	1	0	0	0	0	0	1	0	0	2
Cluster1321;	Proteobac	Gamm	Xantl	Xanthor	Frateuria	0	0	0	0	2	0	0	0	0	0	0	0	2
Cluster1325;	Proteobac	Deltap	Myxc	Cystobacteraceae		0	0	0	0	0	1	1	0	0	0	0	0	2
Cluster1326;	Proteobac	Gammaproteobacteria				0	1	0	0	0	0	0	1	0	0	0	0	2
Cluster1329;size=27						0	2	0	0	0	0	0	0	0	0	0	0	2
Cluster1335;	Proteobac	Betapr	Burkl	Burkhol	Burkholderia	0	0	1	0	0	1	0	0	0	0	0	0	2
Cluster1336;	Acidobact	Acidobacteria_Gp3			Bryobacter	0	0	0	0	0	1	0	1	0	0	0	0	2
Cluster1372;	Proteobac	Alphap	Rhodospirillales			0	0	0	0	0	0	1	0	1	0	0	0	2
Cluster1377;	Proteobac	Alphaproteobacteria				0	0	0	0	0	0	0	0	0	1	0	1	2
Cluster1382;	Verrucom	Opituta	Opitu	Opituta	Opitutus	0	0	1	0	0	0	0	0	0	0	0	1	2
Cluster1393;	Acidobact	Acidobacteria_Gp2			Gp2	0	0	0	0	1	0	0	0	0	0	1	0	2
Cluster1419;	Acidobact	Acidobacteria_Gp1			Gp1	0	0	1	1	0	0	0	0	0	0	0	0	2
Cluster1423;	Proteobacteria					0	1	0	0	1	0	0	0	0	0	0	0	2
Cluster1428;	Proteobac	Deltap	Myxc	Cystoba	Stigmatella	0	0	0	0	0	0	1	0	0	0	1	0	2
Cluster1432;	Proteobac	Deltaproteobacteria				0	0	0	0	0	0	1	0	1	0	0	0	2
Cluster1444;	Acidobact	Acidobacteria_Gp6			Gp6	0	1	0	0	0	0	0	0	0	1	0	0	2
Cluster1458;size=13						1	0	0	0	0	0	0	0	0	1	0	0	2
Cluster1461;	Acidobact	Acidobacteria_Gp1			Edaphobacter	0	0	0	1	1	0	0	0	0	0	0	0	2
Cluster1470;	Acidobact	Acidobacteria_Gp2			Gp2	0	0	1	1	0	0	0	0	0	0	0	0	2
Cluster1479;	Acidobact	Acidobacteria_Gp2			Gp2	0	0	1	0	0	0	0	1	0	0	0	0	2
Cluster1486;	Acidobact	Acidobacteria_Gp1				0	0	0	0	1	0	0	1	0	0	0	0	2
Cluster1498;	Acidobact	Acidobacteria_Gp1			Gp1	1	0	0	0	0	0	1	0	0	0	0	0	2
Cluster1520;size=12						0	0	1	0	0	0	1	0	0	0	0	0	2
Cluster1532;size=26						1	0	0	0	1	0	0	0	0	0	0	0	2
Cluster1565;	Proteobacteria					0	0	1	0	0	0	0	0	0	0	1	0	2
Cluster1567;	Acidobact	Acidobacteria_Gp3			Gp3	0	1	0	0	0	0	1	0	0	0	0	0	2
Cluster1574;	Acidobact	Acidobacteria_Gp3			Gp3	0	0	1	1	0	0	0	0	0	0	0	0	2
Cluster1577;	Actinobac	Actinol	Actin	Thermo	Actinomadura	0	0	0	0	0	0	0	0	0	0	0	2	2

Cluster1578;	Acidobact	Acidobacteria_Gp1			Gp1	0	0	0	0	1	1	0	0	0	0	0	0	2
Cluster1579;size=9						0	1	0	0	0	0	0	0	0	0	1	0	2
Cluster1591;	Acidobact	Acidobacteria_Gp3				0	0	1	0	0	0	0	0	0	0	1	0	2
Cluster1597;size=11						0	0	0	0	1	0	0	1	0	0	0	0	2
Cluster1603;	Proteobac	Alphap	Rhizc	Beijerinckiaceae		0	0	0	0	0	0	0	0	0	1	1	0	2
Cluster1604;	Acidobact	Acidobacteria_Gp3			Gp3	0	0	0	0	0	0	1	0	0	0	1	0	2
Cluster1616;	Proteobac	Alphap	Rhizobiales			1	0	0	0	0	1	0	0	0	0	0	0	2
Cluster1637;	Acidobact	Acidobacteria_Gp2			Gp2	0	0	0	0	2	0	0	0	0	0	0	0	2
Cluster1641;	Bacteroid	Sphing	Sphir	Chitinophagaceae		1	0	0	0	0	0	0	0	0	0	1	0	2
Cluster1643;size=7						1	0	1	0	0	0	0	0	0	0	0	0	2
Cluster1645;	Proteobac	Alphap	Rhod	Acetobacteraceae		0	0	0	0	0	1	0	0	0	1	0	0	2
Cluster1658;	Acidobact	Acidobacteria_Gp1			Granulicella	0	0	1	0	0	0	0	0	0	0	1	0	2
Cluster1661;	Verrucom	Subdivision3			Subdivision3_g	0	0	0	1	0	0	1	0	0	0	0	0	2
Cluster1667;	Actinobac	Actinol	Actinomycetales			0	0	0	0	1	1	0	0	0	0	0	0	2
Cluster1678;	Verrucom	Opitut	Opitu	Opituta	Opitutus	0	1	0	0	0	0	0	0	0	0	0	1	2
Cluster1703;	Acidobact	Acidobacteria_Gp3			Gp3	0	0	0	0	0	0	1	0	0	0	0	1	2
Cluster1710;	Proteobac	Gammaproteobacteria				1	0	0	0	0	0	0	0	0	0	0	1	2
Cluster1711;	Proteobac	Alphap	Rhizc	Rhizobia	Bauldia	0	0	0	0	1	0	0	1	0	0	0	0	2
Cluster1731;	Acidobact	Acidobacteria_Gp1				0	0	0	0	0	0	0	1	0	0	0	1	2
Cluster1733;	Acidobact	Acidobacteria_Gp3				0	0	0	0	1	0	0	0	0	0	0	1	2
Cluster1762;	Proteobac	Gammaproteobacteria				0	0	0	0	0	1	0	0	0	1	0	0	2
Cluster1764;	Proteobac	Gamm	Xantl	Sinobac	Steroidobacter	0	0	1	0	0	0	0	0	0	0	1	0	2
Cluster1778;	Proteobac	Gammaproteobacteria				0	0	0	0	2	0	0	0	0	0	0	0	2
Cluster1806;	Proteobac	Alphap	Rhod	Acetobacteraceae		0	0	2	0	0	0	0	0	0	0	0	0	2
Cluster1817;size=4						0	0	1	1	0	0	0	0	0	0	0	0	2
Cluster1827;	Proteobac	Deltap	Bdell	Bdellovi	Bdellovibrio	0	1	0	1	0	0	0	0	0	0	0	0	2
Cluster1835;	Proteobac	Alphaproteobacteria				0	0	0	0	0	0	0	0	0	1	1	0	2
Cluster1836;	Proteobac	Alphap	Caulc	Caulobacteraceae		2	0	0	0	0	0	0	0	0	0	0	0	2
Cluster1849;	Proteobac	Gamm	Xantl	Sinobac	Steroidobacter	0	1	0	0	0	0	0	0	0	1	0	0	2

Cluster1855;	Acidobact	Acidobacteria_Gp2			Gp2	0	0	0	0	0	0	0	1	0	0	0	1	2
Cluster1872;	Acidobact	Acidobacteria_Gp2			Gp2	0	0	0	1	0	0	0	1	0	0	0	0	2
Cluster1888;	Proteobac	Deltap	Myxococcales			0	0	0	0	0	0	1	0	1	0	0	0	2
Cluster1889;	Acidobact	Acidobacteria_Gp1				0	0	1	0	1	0	0	0	0	0	0	0	2
Cluster1890;size=2						0	0	0	0	1	0	0	0	0	0	1	0	2
Cluster1917;	Acidobact	Acidobacteria_Gp1			Gp1	0	0	0	0	0	0	0	1	1	0	0	0	2
Cluster1919;	Proteobac	Deltap	Myxococcales			0	0	1	1	0	0	0	0	0	0	0	0	2
Cluster1928;	Chlamydia	Chlamy	Chlamydiales			0	0	0	0	1	0	1	0	0	0	0	0	2
Cluster1951;	Acidobacteria					1	0	0	0	0	0	0	1	0	0	0	0	2
Cluster1972;size=6						1	0	0	0	0	0	1	0	0	0	0	0	2
Cluster1982;	Acidobact	Acidobacteria_Gp1			Gp1	1	0	0	0	0	0	0	1	0	0	0	0	2
Cluster1996;	Acidobact	Acidobacteria_Gp2			Gp2	0	0	1	0	0	0	0	0	0	0	1	0	2
Cluster2006;	Acidobact	Acidobacteria_Gp2			Gp2	0	0	0	0	1	0	0	1	0	0	0	0	2
Cluster2023;	Proteobac	Alphaproteobacteria				1	0	0	0	0	0	0	0	0	0	0	1	2
Cluster2047;size=9						1	0	0	1	0	0	0	0	0	0	0	0	2
Cluster2067;	Proteobac	Alphaproteobacteria				0	0	0	0	1	0	0	0	1	0	0	0	2
Cluster2078;	Chlamydia	Chlamy	Chlar	Parachla	Parachlamydia	0	0	0	0	0	1	1	0	0	0	0	0	2
Cluster2097;	Proteobac	Deltaproteobacteria				0	1	1	0	0	0	0	0	0	0	0	0	2
Cluster2099;	Acidobact	Acidobacteria_Gp3				0	0	0	0	0	1	1	0	0	0	0	0	2
Cluster2123;	Acidobact	Acidobacteria_Gp3			Gp3	0	0	0	0	1	0	1	0	0	0	0	0	2
Cluster2131;	Acidobact	Acidobacteria_Gp2			Gp2	1	0	0	0	1	0	0	0	0	0	0	0	2
Cluster2133;	Acidobact	Acidobacteria_Gp2			Gp2	0	0	1	0	1	0	0	0	0	0	0	0	2
Cluster2220;	Proteobac	Betaproteobacteria				1	0	0	0	0	0	1	0	0	0	0	0	2
Cluster2221;	Acidobact	Acidobacteria_Gp1				0	0	0	0	1	0	0	1	0	0	0	0	2
Cluster2224;	Acidobact	Acidobacteria_Gp3			Gp3	0	0	0	1	0	1	0	0	0	0	0	0	2
Cluster2234;	Proteobac	Alphap	Alphaproteob		Rhizomicrobiu	1	0	0	0	0	0	0	0	0	1	0	0	2
Cluster2241;	Proteobac	Gammaproteobacteria				0	2	0	0	0	0	0	0	0	0	0	0	2
Cluster2282;	Proteobac	Deltap	Bdell	Bdellovi	Bdellovibrio	0	1	0	0	0	0	0	0	0	1	0	0	2
Cluster2287;	Acidobact	Acidobacteria_Gp3			Gp3	0	1	0	1	0	0	0	0	0	0	0	0	2

Cluster2303;	Acidobacteria					1	0	0	0	0	0	0	1	0	0	0	0	2
Cluster2310;	Actinobac	Actinol	Actinomycetales			0	1	0	0	0	1	0	0	0	0	0	0	2
Cluster2318;	Acidobact	Acidobacteria_Gp1			Gp1	0	0	0	0	0	0	0	0	0	2	0	0	2
Cluster2343;	Acidobacteria					0	0	0	0	0	0	0	0	0	1	1	0	2
Cluster2354;size=5						0	1	0	0	0	0	1	0	0	0	0	0	2
Cluster2358;	Bacteroid	Sphing	Sphir	Sphingc	Mucilaginibact	1	0	0	0	0	0	0	0	1	0	0	0	2
Cluster2406;	Acidobact	Acidobacteria_Gp1			Gp1	0	0	0	0	1	1	0	0	0	0	0	0	2
Cluster2407;	Acidobact	Acidobacteria_Gp1			Gp1	2	0	0	0	0	0	0	0	0	0	0	0	2
Cluster2417;	Planctomy	Plancto	Planc	Plancto	Zavarzinella	0	1	0	0	0	0	1	0	0	0	0	0	2
Cluster2423;	Acidobact	Acidobacteria_Gp1				0	0	1	0	0	0	0	0	1	0	0	0	2
Cluster2434;	Proteobac	Alphap	Rhodospirillales			0	0	1	0	0	0	0	0	0	0	1	0	2
Cluster2447;	Proteobac	Alphap	Rhod	Acetobacteraceae		0	1	0	0	0	0	0	0	0	0	0	1	2
Cluster2466;	Acidobact	Acidobacteria_Gp2			Gp2	2	0	0	0	0	0	0	0	0	0	0	0	2
Cluster2469;	Proteobac	Gamm	Xantl	Sinobac	Steroidobacter	0	0	0	0	0	1	0	0	0	0	0	1	2
Cluster2472;	Proteobac	Betapr	Rhod	Rhodocyclaceae		0	0	1	0	0	1	0	0	0	0	0	0	2
Cluster2498;	Acidobact	Acidobacteria_Gp1				1	0	0	0	0	0	1	0	0	0	0	0	2
Cluster2533;size=8						0	0	0	1	0	0	0	0	1	0	0	0	2
Cluster2537;	Bacteroid	Sphing	Sphir	Chitinop	Ferruginibacte	0	0	1	0	0	0	0	0	0	1	0	0	2
Cluster2543;	Bacteroid	Sphing	Sphir	Chitinophagaceae		0	0	0	0	0	0	1	0	0	1	0	0	2
Cluster2594;size=11						0	0	0	0	0	0	0	0	1	0	0	1	2
Cluster2611;	Acidobact	Acidobacteria_Gp1			Edaphobacter	0	0	0	0	0	0	0	0	0	2	0	0	2
Cluster2623;size=9						1	0	0	0	0	0	0	1	0	0	0	0	2
Cluster2627;	Acidobacteria					0	2	0	0	0	0	0	0	0	0	0	0	2
Cluster2651;	Acidobact	Acidobacteria_Gp3			Gp3	0	0	0	1	0	0	0	0	0	0	1	0	2
Cluster2673;	Proteobac	Deltap	Myxc	Kofleria	Kofleria	0	0	0	0	0	0	0	1	1	0	0	0	2
Cluster2678;	Acidobact	Acidobacteria_Gp2			Gp2	0	0	1	1	0	0	0	0	0	0	0	0	2
Cluster2707;	Planctomy	Plancto	Planc	Plancto	Singulisphaera	1	0	0	0	0	0	0	0	1	0	0	0	2
Cluster2724;	Acidobact	Acidobacteria_Gp2			Gp2	0	0	0	1	0	0	0	0	0	0	1	0	2
Cluster2743;size=17						0	0	0	0	0	1	1	0	0	0	0	0	2

Cluster2751;	Acidobact	Acidobacteria_Gp1			Edaphobacter	1	0	0	0	0	0	0	0	0	0	1	0	2
Cluster2764;	Acidobact	Acidobacteria_Gp1			Gp1	0	0	0	0	0	0	1	1	0	0	0	0	2
Cluster2766;size=17						0	1	0	0	0	0	0	0	0	0	1	0	2
Cluster2783;	Proteobac	Betapr	Burkl	Burkhol	Burkholderia	0	0	1	0	0	0	1	0	0	0	0	0	2
Cluster2794;	Acidobact	Acidobacteria_Gp1			Gp1	0	0	0	2	0	0	0	0	0	0	0	0	2
Cluster2801;	Verrucomicrobia					1	0	0	0	0	1	0	0	0	0	0	0	2
Cluster2837;	Proteobac	Betapr	Burkl	Burkholderiales_incert		0	0	0	0	0	2	0	0	0	0	0	0	2
Cluster2842;	Acidobact	Acidobacteria_Gp3			Gp3	0	0	0	0	0	0	0	0	0	0	0	2	2
Cluster2862;	Proteobac	Alphap	Rhizobiales			0	0	1	0	1	0	0	0	0	0	0	0	2
Cluster2868;	Proteobac	Gamm	Xantl	Sinobac	Steroidobacter	0	1	1	0	0	0	0	0	0	0	0	0	2
Cluster2870;	Acidobact	Acidobacteria_Gp1			Gp1	0	2	0	0	0	0	0	0	0	0	0	0	2
Cluster2883;	Proteobac	Alphap	Alphaproteob		Rhizomicrobiu	1	0	0	0	0	0	0	1	0	0	0	0	2
Cluster2890;	Chloroflex	Ktedor	Ktedonobacterales			0	0	0	0	0	0	1	0	0	0	1	0	2
Cluster2891;	Verrucom	Subdivision3			Subdivision3_g	0	0	1	0	0	0	0	0	0	1	0	0	2
Cluster2916;size=8						1	0	1	0	0	0	0	0	0	0	0	0	2
Cluster2917;	Verrucom	Subdivision3			Subdivision3_g	0	0	0	0	0	1	0	0	0	0	1	0	2
Cluster2929;	Acidobact	Acidobacteria_Gp3				0	0	0	0	1	0	1	0	0	0	0	0	2
Cluster2932;	Actinobac	Actinol	Actinomycetales			0	0	0	0	0	1	1	0	0	0	0	0	2
Cluster2999;	Proteobac	Alphap	Rhod	Acetobacteraceae		0	0	1	0	0	0	0	0	0	0	1	0	2
Cluster3044;	Bacteroid	Sphing	Sphir	Sphingc	Mucilaginibact	0	0	1	1	0	0	0	0	0	0	0	0	2
Cluster3105;	Proteobac	Gamm	Legio	Coxiella	Coxiella	0	0	0	0	0	2	0	0	0	0	0	0	2
Cluster3121;	Chlamydia	Chlamy	Chlamydiales			0	0	0	0	0	0	2	0	0	0	0	0	2
Cluster3134;	Proteobac	Alphap	Rhizc	Xanthol	Pseudolabrys	0	0	0	0	1	0	1	0	0	0	0	0	2
Cluster3145;	Gemmatir	Gemm	Gemi	Gemma	Gemmatimona	0	1	0	0	0	0	0	0	0	0	0	1	2
Cluster3153;	Proteobac	Gammaproteobacteria				0	0	0	1	0	1	0	0	0	0	0	0	2
Cluster3156;	Actinobac	Actinol	Actin	Thermomonosporacea		0	0	1	0	1	0	0	0	0	0	0	0	2
Cluster3181;	Proteobac	Alphap	Caulc	Caulobа	Phenylobacter	0	0	0	1	0	0	0	0	1	0	0	0	2
Cluster3183;	Acidobacteria					0	1	0	0	0	0	0	1	0	0	0	0	2
Cluster3184;	Acidobact	Acidobacteria_Gp2			Gp2	0	0	0	1	0	0	0	0	0	0	0	1	2

Cluster3188;	Proteobac	Alphap	Rhodospirillales			0	1	0	0	0	0	0	0	0	0	0	1	2
Cluster3215;	Acidobact	Acidobacteria_Gp2			Gp2	0	0	0	0	0	0	1	1	0	0	0	0	2
Cluster3220;	Acidobact	Acidobacteria_Gp2			Gp2	0	0	0	0	0	0	0	0	0	0	0	2	2
Cluster3244;	Acidobact	Acidobacteria_Gp2			Gp2	0	0	0	0	1	0	1	0	0	0	0	0	2
Cluster3260;	Acidobact	Acidobacteria_Gp2			Gp2	0	0	1	0	1	0	0	0	0	0	0	0	2
Cluster3271;size=8						0	0	2	0	0	0	0	0	0	0	0	0	2
Cluster3292;	Proteobac	Alphap	Rhizobiales			2	0	0	0	0	0	0	0	0	0	0	0	2
Cluster3312;	Verrucom	Subdivision3			Subdivision3_	0	0	0	0	1	0	0	0	0	0	0	1	2
Cluster3314;	Planctom	Plancto	Planc	Plancto	Singulisphaera	0	0	0	0	0	0	0	2	0	0	0	0	2
Cluster3330;	Planctom	Plancto	Planc	Plancto	Singulisphaera	0	0	0	0	0	0	0	1	1	0	0	0	2
Cluster3344;	Proteobac	Gamm	Xantl	Sinobac	Steroidobacter	0	0	0	0	0	0	0	0	0	1	1	0	2
Cluster3348;	Planctom	Plancto	Planc	Plancto	Singulisphaera	0	0	0	0	0	0	0	0	0	1	1	0	2
Cluster3375;	Verrucom	Opitutae				1	0	0	0	1	0	0	0	0	0	0	0	2
Cluster3382;	Bacteroid	Sphing	Sphir	Chitinophagaceae		0	1	0	0	1	0	0	0	0	0	0	0	2
Cluster3394;	Proteobac	Alphap	Caulc	Cauloba	Caulobacter	0	0	1	0	0	0	0	0	0	1	0	0	2
Cluster3401;size=2						0	0	0	0	0	1	0	0	0	0	1	0	2
Cluster3409;	Acidobact	Acidobacteria_Gp3				0	0	0	0	0	0	0	0	1	0	0	1	2
Cluster3414;	Verrucom	Spartobacteria			Spartobacteria	0	0	0	0	0	0	1	0	1	0	0	0	2
Cluster3442;	Planctomycetes					0	1	1	0	0	0	0	0	0	0	0	0	2
Cluster3444;	Proteobac	Betapr	Burkl	Burkhol	Burkholderia	0	1	0	0	0	0	0	1	0	0	0	0	2
Cluster3461;size=14						0	0	0	0	0	1	0	1	0	0	0	0	2
Cluster3511;	Acidobacteria					0	0	0	0	0	0	0	0	1	0	0	1	2
Cluster3531;	Acidobact	Acidobacteria_Gp2			Gp2	2	0	0	0	0	0	0	0	0	0	0	0	2
Cluster3590;	Actinobac	Actinol	Acidimicrobiales			0	0	0	0	1	0	0	0	0	1	0	0	2
Cluster3592;	Proteobac	Deltap	Bdell	Bdellovi	Vampirovibrio	0	0	1	1	0	0	0	0	0	0	0	0	2
Cluster3620;	Proteobac	Alphap	Rhizc	Beijerin	Chelatococcus	0	0	0	1	0	0	0	0	1	0	0	0	2
Cluster3655;size=5						0	0	0	1	0	0	1	0	0	0	0	0	2
Cluster3661;	Proteobac	Gammaproteobacteria				0	0	0	0	2	0	0	0	0	0	0	0	2
Cluster3666;	Proteobac	Alphap	Caulc	Caulobacteraceae		0	0	1	0	0	0	0	0	0	0	1	0	2

Cluster3672;	Proteobac	Alphap	Rhod	Rhodospirillaceae		0	0	0	0	1	0	0	1	0	0	0	0	2
Cluster3706;	Proteobac	Alphap	Caulc	Caulobacteraceae		0	1	0	1	0	0	0	0	0	0	0	0	2
Cluster3711;	Planctomy	Plancto	Planc	Planctomycetaceae		0	0	0	2	0	0	0	0	0	0	0	0	2
Cluster3748;size=8						0	0	1	0	0	0	0	0	0	1	0	0	2
Cluster3759;	Proteobac	Gamm	Legio	Coxiella	Aquicella	0	0	0	0	0	2	0	0	0	0	0	0	2
Cluster3767;	Proteobac	Alphap	Rhizc	Beijerinckiaceae		0	1	0	0	0	0	0	0	0	0	0	1	2
Cluster3817;	Proteobac	Gamm	Enter	Enterobacteriaceae		1	0	0	0	0	0	1	0	0	0	0	0	2
Cluster3819;	Acidobact	Acidobacteria_Gp1				0	0	0	1	0	0	1	0	0	0	0	0	2
Cluster3853;	Acidobact	Acidobacteria_Gp2			Gp2	0	0	0	0	0	0	0	1	0	0	0	1	2
Cluster3861;	Proteobac	Alphap	Rhodospirillales			0	1	0	0	0	0	0	0	1	0	0	0	2
Cluster3868;	Actinobac	Actinol	Acidi	Acidimi	Aciditerrimona	0	0	0	0	0	1	1	0	0	0	0	0	2
Cluster3966;	Planctomy	Plancto	Planc	Plancto	Singulisphaera	0	0	0	1	0	0	1	0	0	0	0	0	2
Cluster3976;	Bacteroid	Sphing	Sphir	Chitinophagaceae		0	0	1	0	1	0	0	0	0	0	0	0	2
Cluster4041;	Bacteroid	Bacteroidetes_incert			Ohtaekwangia	0	1	0	1	0	0	0	0	0	0	0	0	2
Cluster4075;size=2						0	1	0	0	0	0	0	0	1	0	0	0	2
Cluster4098;size=7						0	0	0	0	0	1	0	0	0	0	1	0	2
Cluster4099;	Acidobact	Acidobacteria_Gp1			Gp1	0	0	0	0	0	0	0	0	0	0	2	0	2
Cluster4169;	Proteobac	Alphap	Rhodospirillales			0	0	0	0	0	0	1	0	0	0	0	1	2
Cluster4180;	Proteobac	Alphap	Rhodospirillales			0	0	0	0	0	1	0	0	0	0	1	0	2
Cluster4182;	Verrucom	Opitut	Opitu	Opituta	Opitutus	0	0	0	0	0	1	0	0	0	0	1	0	2
Cluster4195;	Verrucom	Opitut	Opitu	Opituta	Opitutus	0	0	0	0	0	1	0	0	0	1	0	0	2
Cluster4199;	Verrucom	Opitut	Opitu	Opituta	Opitutus	0	0	0	0	0	2	0	0	0	0	0	0	2
Cluster4213;size=8						0	1	0	0	0	0	0	0	0	0	1	0	2
Cluster4280;size=9						0	0	0	0	1	0	1	0	0	0	0	0	2
Cluster4305;size=8						0	0	1	0	0	0	0	0	1	0	0	0	2
Cluster4308;	Proteobac	Alphap	Rhizobiales			0	0	0	1	0	1	0	0	0	0	0	0	2
Cluster4330;	Proteobac	Gammaproteobacteria				0	0	0	0	1	1	0	0	0	0	0	0	2
Cluster4359;	Verrucom	Subdivision3			Subdivision3_g	0	0	0	0	0	0	0	0	0	1	1	0	2
Cluster4426;	Proteobac	Alphap	Rhodospirillales			0	0	0	2	0	0	0	0	0	0	0	0	2

Cluster4437;	Proteobac	Alphap	Rhod	Acetobacteraceae		0	0	0	0	0	0	0	0	0	1	1	0	2
Cluster4455;	Proteobac	Alphap	Rhizc	Bradyrhizobiaceae		0	0	0	0	2	0	0	0	0	0	0	0	2
Cluster4460;	Proteobac	Alphap	Rhod	Rhodospirillaceae		0	0	1	0	0	0	0	0	0	0	0	1	2
Cluster4464;	Bacteroid	Sphing	Sphir	Sphingc	Mucilaginibact	1	0	0	0	0	0	0	0	0	1	0	0	2
Cluster4537;	Proteobac	Gamm	Xantl	Xanthor	Pseudoxanthor	0	0	0	0	0	0	0	2	0	0	0	0	2
Cluster4555;	Bacteroid	Sphing	Sphir	Sphingobacteriaceae		0	0	0	1	0	0	0	0	1	0	0	0	2
Cluster4561;size=7						0	0	0	0	0	0	0	1	0	0	1	0	2
Cluster4607;	Acidobacteria					0	1	0	0	0	0	0	0	0	0	1	0	2
Cluster4624;	Actinobac	Actinol	Actinomycetales			2	0	0	0	0	0	0	0	0	0	0	0	2
Cluster4656;	Verrucomicrobia					0	0	0	0	0	0	0	1	0	1	0	0	2
Cluster4685;	Acidobact	Acidobacteria_Gp2			Gp2	0	0	0	0	0	0	0	1	0	0	0	1	2
Cluster4694;	Proteobac	Alphap	Rhizc	Bradyrh	Bradyrhizobiur	0	1	0	0	0	0	1	0	0	0	0	0	2
Cluster4702;size=11						0	0	0	0	0	2	0	0	0	0	0	0	2
Cluster4717;	Proteobacteria					0	0	1	0	0	0	0	0	0	1	0	0	2
Cluster4823;	Proteobac	Alphaproteobacteria				0	0	0	0	0	0	0	1	0	0	0	1	2
Cluster4847;	Acidobact	Acidobacteria_Gp1			Gp1	0	0	0	0	0	0	0	2	0	0	0	0	2
Cluster4854;	Planctomycetes					0	0	1	0	0	0	0	0	0	0	1	0	2
Cluster4861;	Proteobac	Alphap	Rhizobiales			0	0	0	0	0	0	0	1	1	0	0	0	2
Cluster4885;	Planctomycetes					0	0	0	0	1	0	0	1	0	0	0	0	2
Cluster4935;size=8						0	0	1	0	0	1	0	0	0	0	0	0	2
Cluster5043;	Proteobac	Alphaproteobacteria				0	0	0	0	0	0	0	2	0	0	0	0	2
Cluster5060;	Proteobac	Deltaproteobacteria				0	0	0	1	0	0	0	0	0	1	0	0	2
Cluster5093;	Verrucom	Subdivision3			Subdivision3_g	0	0	0	0	0	1	0	1	0	0	0	0	2
Cluster5101;	Proteobac	Alphap	Rhizobiales			0	0	0	0	0	0	0	0	0	0	1	1	2
Cluster5110;	Proteobac	Alphap	Sphir	Sphingc	Sphingomonas	0	0	0	0	0	0	0	0	0	2	0	0	2
Cluster5120;	Proteobac	Alphap	Rhizobiales			0	0	0	1	0	0	1	0	0	0	0	0	2
Cluster5162;	Proteobac	Betaproteobacteria				1	0	0	0	0	0	0	0	1	0	0	0	2
Cluster5166;	Verrucom	Spartobacteria			Spartobacteria	0	0	0	0	0	0	0	0	0	2	0	0	2
Cluster5171;	Acidobact	Acidobacteria_Gp2			Gp2	0	0	0	1	0	0	0	0	0	0	0	1	2

Cluster5175;	Planctomycetes					0	0	0	0	0	0	1	0	0	0	1	0	2
Cluster5185;	Proteobac	Betapr	Burkl	Burkhol	Burkholderia	0	0	1	1	0	0	0	0	0	0	0	0	2
Cluster5195;	Acidobact	Acidobacteria_Gp1			Acidobacteriur	0	0	0	1	1	0	0	0	0	0	0	0	2
Cluster5197;	Planctomy	Plancto	Planc	Plancto	Singulisphaera	0	0	0	0	0	0	1	0	0	0	1	0	2
Cluster5245;	Proteobac	Alphap	Rhodospirillales			0	0	0	0	1	0	0	0	1	0	0	0	2
Cluster5314;	Proteobac	Deltap	Myxc	Cystobacteraceae		0	0	0	0	1	1	0	0	0	0	0	0	2
Cluster5317;	TM7				TM7_genera_i	0	0	0	0	0	0	2	0	0	0	0	0	2
Cluster5322;	Acidobact	Acidobacteria_Gp2			Gp2	0	0	0	0	0	0	2	0	0	0	0	0	2
Cluster5327;	Proteobac	Alphap	Rhodospirillales			0	0	0	0	0	0	0	1	1	0	0	0	2
Cluster5366;	Proteobac	Alphap	Rhizobiales			0	2	0	0	0	0	0	0	0	0	0	0	2
Cluster5389;	Verrucom	Opituta	Opitu	Opituta	Opitutus	1	0	1	0	0	0	0	0	0	0	0	0	2
Cluster5395;	Proteobac	Betaproteobacteria				0	0	1	0	0	0	1	0	0	0	0	0	2
Cluster5401;	Acidobact	Acidobacteria_Gp2			Gp2	0	0	0	0	0	0	0	1	0	0	0	1	2
Cluster5441;	Proteobac	Alphap	Rhodospirillales			0	1	0	0	0	0	0	1	0	0	0	0	2
Cluster5445;	Acidobact	Acidobacteria_Gp3				0	0	0	0	0	0	1	0	0	0	1	0	2
Cluster5496;	Proteobac	Betapr	Rhod	Rhodocyclaceae		0	1	0	0	0	0	0	0	0	0	0	1	2
Cluster5519;size=11						1	0	0	0	0	0	0	0	0	1	0	0	2
Cluster5527;	Acidobact	Acidobacteria_Gp2			Gp2	0	0	0	0	0	0	0	0	0	0	2	0	2
Cluster5533;	Acidobact	Acidobacteria_Gp3				0	0	0	0	0	0	2	0	0	0	0	0	2
Cluster5588;size=6						0	0	0	0	0	1	1	0	0	0	0	0	2
Cluster5601;	Proteobac	Alphap	Rhizc	Bradyrhizobiaceae		0	0	0	0	0	0	0	1	0	0	1	0	2
Cluster5632;	Proteobac	Deltaproteobacteria				0	0	0	1	0	0	0	0	1	0	0	0	2
Cluster5684;	Proteobac	Alphap	Rhodospirillales			0	0	0	0	0	0	0	2	0	0	0	0	2
Cluster5687;	Acidobact	Acidobacteria_Gp5			Gp5	0	1	0	0	0	0	1	0	0	0	0	0	2
Cluster5694;	Verrucom	Opituta	Opitu	Opituta	Opitutus	0	0	0	0	1	0	0	1	0	0	0	0	2
Cluster5713;	Acidobact	Acidobacteria_Gp2			Gp2	0	0	0	0	0	0	0	0	0	0	1	1	2
Cluster5838;	Proteobac	Betaproteobacteria				0	2	0	0	0	0	0	0	0	0	0	0	2
Cluster5841;	Verrucom	Subdivision3			Subdivision3_g	0	0	0	1	0	0	0	0	0	0	0	1	2
Cluster5843;	Bacteroid	Sphing	Sphir	Chitinophagaceae		0	0	1	0	1	0	0	0	0	0	0	0	2

Cluster5865;:	Proteobacteria					0	0	1	1	0	0	0	0	0	0	0	0	2
Cluster5869;:	Acidobact	Acidobacteria_Gp2			Gp2	1	0	1	0	0	0	0	0	0	0	0	0	2
Cluster5877;:	Acidobact	Acidobacteria_Gp2			Gp2	0	0	1	0	0	0	1	0	0	0	0	0	2
Cluster5897;size=9						0	0	0	0	1	0	1	0	0	0	0	0	2
Cluster5916;:	Proteobac	Alphap	Rhizobiales			0	0	0	0	1	1	0	0	0	0	0	0	2
Cluster5936;:	Proteobac	Betapr	Rhod	Rhodocyclaceae		0	0	1	0	0	0	0	0	1	0	0	0	2
Cluster5962;:	Proteobac	Betapr	Rhod	Rhodocyclaceae		1	0	0	0	0	0	0	1	0	0	0	0	2
Cluster5964;:	Acidobact	Acidobacteria_Gp2			Gp2	0	0	0	0	0	0	0	1	1	0	0	0	2
Cluster5980;:	Proteobac	Gamm	Xantl	Xanthor	Frateuria	1	0	0	0	0	0	1	0	0	0	0	0	2
Cluster6048;:	Proteobac	Alphap	Rhizobiales			0	0	1	0	0	0	0	0	0	0	0	1	2
Cluster6061;size=10						0	1	0	0	0	0	0	1	0	0	0	0	2
Cluster6064;:	Armatimc	Armati	Arma	Armatir	Armatimonas/.	0	0	1	0	0	0	0	0	0	0	0	1	2
Cluster6109;:	Acidobact	Acidobacteria_Gp1				0	0	0	0	0	0	2	0	0	0	0	0	2
Cluster6121;:	Proteobac	Alphaproteobacteria				2	0	0	0	0	0	0	0	0	0	0	0	2
Cluster6147;:	Planctomy	Planctc	Planc	Plancto	Planctomyces	0	0	0	0	1	1	0	0	0	0	0	0	2
Cluster6156;:	Proteobac	Alphap	Rhizobiales			0	0	0	0	1	0	0	0	0	0	1	0	2
Cluster6187;:	Proteobac	Alphap	Rhodospirillales			0	1	0	0	0	0	0	0	1	0	0	0	2
Cluster6189;:	Proteobac	Alphap	Rhizc	Hyphomicrobiaceae		1	0	0	0	0	0	1	0	0	0	0	0	2
Cluster6217;:	Proteobac	Alphap	Rhod	Rhodos	Dongia	0	0	0	0	0	0	1	1	0	0	0	0	2
Cluster6228;:	Proteobac	Deltap	Myxc	Cystobacteraceae		0	0	0	0	0	0	0	2	0	0	0	0	2
Cluster6235;:	Proteobac	Alphap	Rhodospirillales			0	0	0	2	0	0	0	0	0	0	0	0	2
Cluster6275;:	Proteobac	Alphap	Rhod	Rhodospirillaceae		0	1	0	0	1	0	0	0	0	0	0	0	2
Cluster6287;:	Acidobact	Acidobacteria_Gp2			Gp2	1	0	0	1	0	0	0	0	0	0	0	0	2
Cluster6291;:	Acidobact	Acidobacteria_Gp2			Gp2	0	0	0	0	0	1	1	0	0	0	0	0	2
Cluster6340;:	Proteobac	Alphap	Rhod	Rhodos	Dongia	0	0	0	0	0	2	0	0	0	0	0	0	2
Cluster6343;:	Proteobac	Alphap	Rhizobiales			0	0	0	0	1	0	0	0	0	0	0	1	2
Cluster6350;:	Proteobac	Alphap	Rhod	Rhodos	Dongia	1	0	0	0	0	0	0	1	0	0	0	0	2
Cluster6359;:	Acidobact	Acidobacteria_Gp1				0	0	0	0	0	0	0	0	1	1	0	0	2
Cluster6425;:	Proteobac	Alphap	Rhizc	Xanthol	Pseudolabrys	0	1	0	0	0	0	0	0	0	0	0	1	2

Cluster6436;	Acidobact	Acidobacteria_Gp6			Gp6	0	0	0	0	0	0	0	1	0	0	1	0	2
Cluster6513;	Proteobac	Deltaproteobacteria				0	1	0	0	0	0	0	0	1	0	0	0	2
Cluster6528;size=10						0	0	0	0	0	0	0	1	0	0	1	0	2
Cluster6547;	Proteobac	Alphap	Rhod	Rhodospirillaceae		0	0	0	1	1	0	0	0	0	0	0	0	2
Cluster6563;size=5						1	1	0	0	0	0	0	0	0	0	0	0	2
Cluster6609;	Acidobact	Acidobacteria_Gp1			Terriglobus	0	0	1	1	0	0	0	0	0	0	0	0	2
Cluster6636;	Acidobact	Acidobacteria_Gp2			Gp2	1	0	0	0	1	0	0	0	0	0	0	0	2
Cluster6667;	Elusimicro	Elusim	Elusi	Elusimic	Elusimicrobiun	0	0	0	0	0	0	0	0	0	1	1	0	2
Cluster6670;size=6						0	0	0	0	0	0	0	2	0	0	0	0	2
Cluster6717;	Chloroflex	Ktedor	Ktedonobacterales			0	0	0	0	0	0	0	2	0	0	0	0	2
Cluster6776;	Acidobact	Acidobacteria_Gp1			Gp1	0	0	0	0	0	0	0	1	0	0	0	1	2
Cluster6778;	Verrucom	Subdivision3			Subdivision3_g	0	1	0	0	0	0	1	0	0	0	0	0	2
Cluster6780;	Proteobac	Alphap	Rhodospirillales			0	0	0	1	1	0	0	0	0	0	0	0	2
Cluster6785;	Actinobac	Actinol	Actinomycetales			0	0	0	0	0	1	0	0	0	0	1	0	2
Cluster6786;	Proteobac	Deltap	Bdell	Bdellovi	Bdellovibrio	0	0	0	0	0	1	1	0	0	0	0	0	2
Cluster6805;	Acidobact	Acidobacteria_Gp3			Bryobacter	0	0	1	0	1	0	0	0	0	0	0	0	2
Cluster6837;	Proteobac	Gamm	Xantl	Sinobac	Steroidobacter	1	0	1	0	0	0	0	0	0	0	0	0	2
Cluster6842;	Proteobac	Alphap	Rhodospirillales			0	0	0	0	0	0	0	0	0	0	2	0	2
Cluster6858;	Proteobac	Alphaproteobacteria				0	0	0	0	2	0	0	0	0	0	0	0	2
Cluster6908;	Proteobac	Deltap	Bdell	Bdellovi	Bdellovibrio	1	0	0	0	0	1	0	0	0	0	0	0	2
Cluster6922;	Proteobac	Alphap	Rhizobiales			0	0	0	0	1	1	0	0	0	0	0	0	2
Cluster6923;	Acidobact	Acidobacteria_Gp1				0	0	0	1	0	0	1	0	0	0	0	0	2
Cluster6959;	Acidobact	Acidobacteria_Gp1			Gp1	0	0	0	0	0	0	0	0	0	1	1	0	2
Cluster6979;	Acidobact	Acidobacteria_Gp3			Gp3	0	0	0	0	0	0	1	0	1	0	0	0	2
Cluster6998;	Proteobac	Alphap	Rhodospirillales			1	0	0	0	0	0	0	0	0	0	0	1	2
Cluster7003;	Acidobact	Acidobacteria_Gp1				0	0	1	0	0	0	0	0	0	0	1	0	2
Cluster7048;	Proteobac	Alphap	Rhod	Rhodospirillaceae		0	0	0	0	0	0	0	0	0	1	1	0	2
Cluster7059;	Acidobact	Acidobacteria_Gp3			Gp3	0	1	0	0	0	0	1	0	0	0	0	0	2
Cluster7065;size=12						0	0	0	0	0	1	1	0	0	0	0	0	2

Cluster7120;	Proteobac	Alphap	Rhodospirillales			0	1	0	0	1	0	0	0	0	0	0	0	2
Cluster7179;	Proteobac	Alphaproteobacteria				0	0	1	0	0	1	0	0	0	0	0	0	2
Cluster7192;	Proteobac	Gamm	Xantl	Sinobac	Steroidobacter	0	0	1	0	1	0	0	0	0	0	0	0	2
Cluster7232;	Armatimc	Armati	Arma	Armatir	Armatimonas/	0	1	0	0	0	0	0	0	0	1	0	0	2
Cluster7276;size=4						0	0	1	0	0	0	0	0	0	0	0	1	2
Cluster7284;	Acidobact	Acidobacteria_Gp3			Gp3	1	0	1	0	0	0	0	0	0	0	0	0	2
Cluster7285;	Verrucom	Opituta	Opitu	Opituta	Opitutus	0	0	0	1	0	0	0	0	0	1	0	0	2
Cluster7373;	Acidobact	Acidobacteria_Gp2			Gp2	0	0	1	0	0	1	0	0	0	0	0	0	2
Cluster7388;	Proteobac	Betapr	Burkl	Oxalobacteraceae		0	0	1	1	0	0	0	0	0	0	0	0	2
Cluster7398;	Proteobac	Alphap	Rhizo	Xanthob	Pseudolabrys	0	0	0	1	0	1	0	0	0	0	0	0	2
Cluster7428;	Acidobact	Acidobacteria_Gp2			Gp2	0	0	0	0	0	0	2	0	0	0	0	0	2
Cluster7484;	Verrucom	Subdivision3			Subdivision3_g	1	0	0	0	0	0	0	0	0	0	0	1	2
Cluster7492;size=9						0	1	0	1	0	0	0	0	0	0	0	0	2
Cluster7594;size=5						0	1	0	0	0	0	0	0	0	1	0	0	2
Cluster7623;	Acidobact	Acidobacteria_Gp1				0	0	0	0	0	1	0	0	0	0	1	0	2
Cluster7654;	Acidobact	Acidobacteria_Gp1			Gp1	0	0	0	0	1	0	1	0	0	0	0	0	2
Cluster7702;	Acidobact	Acidobacteria_Gp1			Gp1	0	2	0	0	0	0	0	0	0	0	0	0	2
Cluster7714;	Proteobac	Alphap	Rhizobiales			0	0	0	0	0	0	1	0	1	0	0	0	2
Cluster7726;	Proteobac	Alphap	Rhodospirillales			0	0	1	0	1	0	0	0	0	0	0	0	2
Cluster7736;	Bacteroid	Sphing	Sphir	Chitinophagaceae		0	0	0	0	0	0	1	0	0	0	1	0	2
Cluster7760;	Proteobac	Alphap	Rhodospirillales			0	0	0	0	0	0	1	1	0	0	0	0	2
Cluster7787;	Chloroflex	Ktedor	Ktedonobacterales			0	0	0	0	0	0	0	0	1	0	1	0	2
Cluster7807;	Acidobact	Acidobacteria_Gp1			Gp1	0	0	0	0	0	0	2	0	0	0	0	0	2
Cluster7832;	Proteobac	Alphap	Sphir	Sphingc	Sphingomonas	0	0	0	0	0	1	0	1	0	0	0	0	2
Cluster7904;	Proteobacteria					0	0	0	1	0	0	0	0	0	0	1	0	2
Cluster7948;	Proteobac	Alphap	Rhodospirillales			1	0	0	0	1	0	0	0	0	0	0	0	2
Cluster7955;	Verrucom	Subdivision3			Subdivision3_g	0	0	1	0	1	0	0	0	0	0	0	0	2
Cluster7958;	Proteobac	Alphap	Rhod	Acetobacteraceae		0	0	1	1	0	0	0	0	0	0	0	0	2
Cluster7989;size=23						0	1	0	0	1	0	0	0	0	0	0	0	2

Cluster8006;	Elusimicro	Elusim	Elusi	Elusimic	Elusimicrobiun	1	0	0	0	0	1	0	0	0	0	0	0	2
Cluster8009;	Proteobac	Alphap	Rhod	Rhodospirillaceae		0	1	0	1	0	0	0	0	0	0	0	0	2
Cluster8028;	Proteobac	Alphap	Rhodospirillales			0	0	0	0	0	1	0	0	0	0	1	0	2
Cluster8081;	Proteobac	Alphap	Rhod	Rhodospirillaceae		0	0	0	1	1	0	0	0	0	0	0	0	2
Cluster8084;	Acidobact	Acidobacteria_Gp1			Gp1	0	0	0	0	0	0	1	0	0	0	1	0	2
Cluster8085;	Proteobac	Alphap	Rhodospirillales			0	0	0	1	0	0	0	0	0	1	0	0	2
Cluster8087;	Proteobac	Alphap	Rhodospirillales			0	0	0	0	0	0	0	0	2	0	0	0	2
Cluster8095;	Proteobac	Betapr	Rhod	Rhodocyclaceae		0	0	0	2	0	0	0	0	0	0	0	0	2
Cluster8125;	Verrucom	Subdivision3			Subdivision3_g	0	0	0	0	0	0	0	0	0	2	0	0	2
Cluster8126;	Proteobac	Alphap	Rhodospirillales			0	0	1	0	0	1	0	0	0	0	0	0	2
Cluster8170;	Acidobact	Acidobacteria_Gp2			Gp2	0	0	0	0	0	0	0	0	2	0	0	0	2
Cluster8171;	Verrucom	Subdivision3			Subdivision3_g	0	0	0	0	0	0	0	0	0	2	0	0	2
Cluster8176;	Proteobac	Alphaproteobacteria				0	0	0	0	0	0	0	0	0	2	0	0	2
Cluster8188;	Proteobac	Gammaproteobacteria				2	0	0	0	0	0	0	0	0	0	0	0	2
Cluster8227;	Acidobact	Acidobacteria_Gp1				0	0	0	2	0	0	0	0	0	0	0	0	2
Cluster8234;	Proteobac	Alphap	Rhizobiales			0	0	0	0	1	0	0	1	0	0	0	0	2
Cluster8266;	Proteobac	Alphap	Rhizc	Bradyrh	Bradyrhizobiur	0	0	0	0	1	0	0	1	0	0	0	0	2
Cluster8291;size=20						0	0	0	0	1	0	0	0	0	0	1	0	2
Cluster8295;	Proteobac	Alphap	Rhod	Rhodospirillaceae		0	0	1	1	0	0	0	0	0	0	0	0	2
Cluster8312;	Proteobac	Alphap	Rhizc	Bradyrh	Bradyrhizobiur	0	0	0	0	0	0	0	0	0	0	2	0	2
Cluster8329;	Acidobact	Acidobacteria_Gp2			Gp2	0	1	0	0	0	1	0	0	0	0	0	0	2
Cluster8339;	Firmicutes	Bacilli	Bacill	Paeniba	Paenibacillus	0	0	0	0	0	0	0	0	0	0	1	1	2
Cluster8346;	Acidobact	Acidobacteria_Gp2			Gp2	0	0	1	0	0	0	1	0	0	0	0	0	2
Cluster8403;	Proteobac	Alphap	Rhod	Rhodospirillaceae		0	0	0	0	0	0	0	0	0	0	0	2	2
Cluster8405;	Proteobac	Alphaproteobacteria				0	0	1	0	0	0	0	0	0	0	1	0	2
Cluster8406;size=3						0	0	0	0	1	0	0	0	0	0	1	0	2
Cluster8459;	Proteobac	Alphap	Rhod	Rhodos	Dongia	0	0	0	1	0	1	0	0	0	0	0	0	2
Cluster8465;	Acidobacteria					0	0	0	1	0	0	0	0	0	0	0	1	2
Cluster8511;	Acidobact	Acidobacteria_Gp1			Gp1	0	0	0	2	0	0	0	0	0	0	0	0	2

Cluster8544;	Acidobact	Acidobacteria_Gp2			Gp2	0	0	0	0	0	0	0	0	0	1	1	0	2
Cluster8546;size=7						0	0	0	0	0	0	0	0	0	0	0	2	2
Cluster8556;size=12						0	0	0	1	0	0	0	0	0	0	1	0	2
Cluster8588;	Proteobac	Alphap	Rhodospirillales			0	0	1	0	1	0	0	0	0	0	0	0	2
Cluster8593;	Acidobact	Acidobacteria_Gp3			Gp3	0	0	0	1	0	0	1	0	0	0	0	0	2
Cluster8610;size=13						1	0	0	0	0	0	0	0	0	0	1	0	2
Cluster8683;	Proteobac	Alphap	Rhodospirillales			0	0	1	0	0	0	0	0	0	1	0	0	2
Cluster8702;	Proteobac	Betapr	Burkl	Oxalobacteraceae		0	0	0	0	1	0	0	0	0	0	1	0	2
Cluster8707;	Acidobact	Acidobacteria_Gp3			Gp3	1	0	0	0	0	0	0	0	0	0	1	0	2
Cluster8710;	Acidobact	Acidobacteria_Gp1			Gp1	0	0	0	0	0	1	0	0	0	1	0	0	2
Cluster8731;	Acidobact	Acidobacteria_Gp1			Gp1	0	0	0	0	1	0	0	0	0	1	0	0	2
Cluster8739;	Proteobac	Deltap	Myxococcales			0	0	2	0	0	0	0	0	0	0	0	0	2
Cluster8767;	Proteobac	Alphap	Rhod	Rhodospirillaceae		0	0	0	0	1	0	0	0	0	0	1	0	2
Cluster8773;	Proteobac	Alphap	Rhodospirillales			1	1	0	0	0	0	0	0	0	0	0	0	2
Cluster8778;size=17						0	0	0	0	0	1	0	0	0	0	1	0	2
Cluster8781;	Proteobac	Alphap	Rhodospirillales			0	0	0	0	0	0	1	0	0	0	1	0	2
Cluster8793;	Verrucomicrobia					0	0	1	0	0	0	0	0	0	1	0	0	2
Cluster8794;	Proteobac	Deltap	Bdell	Bdellovi	Bdellovibrio	0	0	0	0	1	0	0	0	0	1	0	0	2
Cluster8804;	Acidobact	Acidobacteria_Gp10			Gp10	0	0	0	1	0	0	0	0	0	0	0	1	2
Cluster8863;	Acidobact	Acidobacteria_Gp3			Gp3	0	0	0	2	0	0	0	0	0	0	0	0	2
Cluster8900;	Acidobact	Acidobacteria_Gp1			Gp1	0	0	1	0	0	0	0	0	0	1	0	0	2
Cluster8908;size=2						0	0	0	0	0	0	1	0	1	0	0	0	2
Cluster8982;	Proteobac	Alphap	Rhodospirillales			0	0	1	0	0	0	0	1	0	0	0	0	2
Cluster8983;	Proteobac	Alphap	Rhodospirillales			1	0	0	0	0	0	0	1	0	0	0	0	2
Cluster9017;size=16						0	0	0	2	0	0	0	0	0	0	0	0	2
Cluster9019;	Gemmati	Gemm	Gem	Gemma	Gemmatimona	0	0	0	0	1	0	1	0	0	0	0	0	2
Cluster9036;	Proteobac	Gammaproteobacteria				0	0	0	1	1	0	0	0	0	0	0	0	2
Cluster9041;	Acidobact	Acidobacteria_Gp1			Gp1	0	0	0	1	0	0	0	0	0	0	0	1	2
Cluster9082;	Verrucom	Spartobacteria			Spartobacteria	0	0	0	0	0	0	2	0	0	0	0	0	2

Cluster9123;:	Proteobac	Alphaproteobacteria				0	0	0	0	0	0	1	0	1	0	0	0	2
Cluster9167;:	Proteobac	Alphap	Rhodospirillales			0	1	0	0	0	0	0	0	0	0	0	1	2
Cluster9221;:	Proteobac	Betapr	Burkl	Burkhol	Burkholderia	0	2	0	0	0	0	0	0	0	0	0	0	2
Cluster9252;:	Proteobac	Alphaproteobacteria				0	0	0	2	0	0	0	0	0	0	0	0	2
Cluster9260;:	Proteobac	Alphap	Rhodospirillales			0	1	0	0	0	0	0	0	0	0	0	1	2
Cluster9269;:	Proteobac	Betapr	Burkl	Burkhol	Burkholderia	0	0	0	1	0	0	0	0	0	0	0	1	2
Cluster9273;size=4						0	0	1	1	0	0	0	0	0	0	0	0	2
Cluster9283;:	Acidobact	Acidobacteria_Gp2			Gp2	0	1	0	0	0	0	0	0	0	0	0	1	2
Cluster9290;:	Proteobac	Alphap	Rhodospirillales			0	1	0	0	0	0	1	0	0	0	0	0	2
Cluster9294;:	Acidobacteria					0	0	1	0	1	0	0	0	0	0	0	0	2
Cluster9347;:	Acidobact	Acidobacteria_Gp2			Gp2	0	1	0	1	0	0	0	0	0	0	0	0	2
Cluster9439;:	Verrucom	Spartobacteria			Spartobacteria	0	1	0	0	0	0	0	0	0	0	0	1	2
Cluster9459;:	Proteobac	Alphap	Rhodospirillales			0	0	0	0	0	1	0	0	0	0	0	1	2
Cluster9491;:	Acidobact	Acidobacteria_Gp1			Acidobacteriur	0	0	0	0	1	0	0	0	0	0	1	0	2
Cluster9533;:	Acidobact	Acidobacteria_Gp3				0	0	0	1	0	0	0	0	0	0	0	1	2
Cluster9674;:	Acidobact	Acidobacteria_Gp2			Gp2	0	0	1	0	0	0	0	1	0	0	0	0	2
Cluster9681;:	Proteobac	Alphap	Rhizc	Bradyrh	Agromonas	1	0	0	1	0	0	0	0	0	0	0	0	2
Cluster9687;:	Proteobac	Deltap	Bdell	Bdellovi	Bdellovibrio	1	0	0	0	0	0	0	0	1	0	0	0	2
Cluster9698;:	Proteobac	Alphaproteobacteria				0	0	0	0	0	0	1	0	1	0	0	0	2
Cluster9862;:	Proteobac	Betaproteobacteria				0	1	0	0	0	1	0	0	0	0	0	0	2
Cluster9875;:	Proteobac	Alphap	Rhod	Rhodospirillaceae		0	0	0	0	0	1	0	0	0	0	0	1	2
Cluster9880;:	Proteobac	Alphap	Rhodospirillales			0	0	0	0	0	0	0	2	0	0	0	0	2
Cluster9908;:	Proteobac	Alphap	Rhizobiales			0	0	0	0	0	0	1	0	0	0	1	0	2
Cluster9940;:	Acidobact	Acidobacteria_Gp1			Gp1	0	0	0	0	0	0	0	2	0	0	0	0	2
Cluster9942;:	Proteobac	Betapr	Burkl	Burkhol	Burkholderia	0	1	0	0	0	0	0	1	0	0	0	0	2
Cluster9947;:	Proteobac	Gammaproteobacteria				0	1	0	0	0	0	0	0	1	0	0	0	2
Cluster10004	Proteobac	Alphap	Rhizc	Bradyrh	Agromonas	0	0	0	0	0	0	1	1	0	0	0	0	2
Cluster10033	Proteobac	Alphap	Rhodospirillales			1	0	0	1	0	0	0	0	0	0	0	0	2
Cluster10092	Proteobac	Deltap	Myxococcales			0	0	0	0	1	0	0	0	0	0	0	1	2

Cluster1014S	Actinobac	Actinol	Actinomycetales			0	0	0	1	1	0	0	0	0	0	0	0	2
Cluster1015S	Verrucom	Opituta	Opitu	Opituta	Opitutus	0	0	0	2	0	0	0	0	0	0	0	0	2
Cluster10184	Actinobac	Actinol	Solirubrobacterales			0	1	0	0	0	0	0	0	0	0	1	0	2
Cluster1019З	Proteobac	Alphap	Caulc	Caulob	Phenylobacter	0	0	0	1	0	0	1	0	0	0	0	0	2
Cluster10194	Acidobact	Acidobacteria_Gp1			Gp1	2	0	0	0	0	0	0	0	0	0	0	0	2
Cluster1019S	Proteobac	Alphaproteobacteria				0	0	0	1	0	0	1	0	0	0	0	0	2
Cluster1022C	Proteobac	Alphap	Rhizobiales			0	0	1	0	0	0	0	0	0	0	0	1	2
Cluster1024E	Proteobac	Alphap	Rhizc	Bradyrh	Bradyrhizobiur	0	1	0	0	0	0	0	1	0	0	0	0	2
Cluster1028E	Proteobac	Alphap	Rhodospirillales			1	0	0	0	0	0	0	0	0	0	0	1	2
Cluster1032	Acidobact	Acidobacteria_Gp3				0	0	0	0	0	0	1	1	0	0	0	0	2
Cluster10353;size=3						0	0	0	0	0	0	1	0	0	0	1	0	2
Cluster1037E	Proteobac	Deltaproteobacteria				0	0	1	0	0	0	1	0	0	0	0	0	2
Cluster1037E	Acidobact	Acidobacteria_Gp1			Acidobacteriur	0	0	0	0	0	0	1	0	0	0	1	0	2
Cluster1038	Proteobac	Deltaproteobacteria				1	0	0	0	0	0	0	1	0	0	0	0	2
Cluster1046S	Proteobac	Alphap	Rhizobiales			0	0	0	0	0	0	1	0	0	0	0	1	2
Cluster1048C	Acidobact	Acidobacteria_Gp3				0	0	0	1	0	0	0	1	0	0	0	0	2
Cluster1050S	Acidobact	Acidobacteria_Gp1				1	0	0	0	0	0	0	0	1	0	0	0	2
Cluster10593;size=4						0	0	1	0	1	0	0	0	0	0	0	0	2
Cluster1059E	Proteobac	Alphap	Rhizc	Beijerinckiaceae		0	0	0	0	0	0	1	1	0	0	0	0	2
Cluster1062	Proteobac	Alphap	Rhizobiales			0	0	0	0	0	0	0	0	0	0	0	2	2
Cluster1064E	Proteobac	Alphap	Rhizobiales			0	0	0	0	1	0	0	0	0	0	1	0	2
Cluster1065	Acidobacteria					1	0	0	0	0	0	0	1	0	0	0	0	2
Cluster10653;size=3						0	0	0	1	0	0	0	0	0	0	1	0	2
Cluster10695;size=9						0	0	1	0	0	0	0	1	0	0	0	0	2
Cluster10703;size=11						0	0	0	0	0	0	1	1	0	0	0	0	2
Cluster1073	Acidobact	Acidobacteria_Gp1				0	0	1	0	0	0	0	0	0	0	1	0	2
Cluster1074S	Bacteroidetes					0	0	0	2	0	0	0	0	0	0	0	0	2
Cluster1075E	Acidobacteria					1	0	1	0	0	0	0	0	0	0	0	0	2
Cluster1079З	Armatimonadetes				Armatimonade	0	0	1	1	0	0	0	0	0	0	0	0	2

Cluster10936	Proteobac	Alphap	Rhizobiales			0	0	0	1	0	0	1	0	0	0	0	0	2
Cluster11018	Acidobact	Acidobacteria_Gp2			Gp2	0	0	0	0	1	0	1	0	0	0	0	0	2
Cluster11030;size=11						0	1	0	0	0	1	0	0	0	0	0	0	2
Cluster11032	Proteobac	Alphap	Caulo	Cauloba	Phenylobacter	0	0	0	0	1	0	1	0	0	0	0	0	2
Cluster11088	Proteobac	Deltap	Myxo	Cystobacteraceae		0	0	0	1	0	0	0	0	1	0	0	0	2
Cluster11113	Acidobact	Acidobacteria_Gp2			Gp2	0	0	0	0	1	0	0	0	0	0	0	1	2
Cluster11114	Proteobacteria					1	0	0	0	0	0	0	0	0	0	0	1	2
Cluster11127	Proteobacteria					1	0	0	0	0	0	1	0	0	0	0	0	2
Cluster11242	Acidobact	Acidobacteria_Gp1			Acidobacteriu	0	0	0	0	0	0	1	0	0	1	0	0	2
Cluster11272	Proteobac	Alphap	Rhizo	Phylloba	Aminobacter	0	0	0	0	0	1	0	1	0	0	0	0	2
Cluster11288	Proteobac	Deltap	Myxococcales			0	0	0	0	0	1	0	1	0	0	0	0	2
Cluster11318	Proteobac	Betapr	Burkholderiales			0	0	0	0	2	0	0	0	0	0	0	0	2
Cluster11334	Acidobact	Acidobacteria_Gp1			Gp1	0	0	1	0	0	0	0	1	0	0	0	0	2
Cluster11356	Acidobact	Acidobacteria_Gp1				0	0	1	0	0	0	0	0	0	1	0	0	2
Cluster11364	Actinobac	Actinob	Actinomycetales			0	0	0	0	0	0	1	1	0	0	0	0	2
Cluster11406	Proteobacteria					0	0	0	0	0	0	0	0	1	1	0	0	2
Cluster11420	Verrucom	Subdivision3			Subdivision3_g	0	0	0	0	0	1	0	1	0	0	0	0	2
Cluster11465	Proteobac	Alphap	Ricke	Ricketts	Rickettsia	1	0	0	0	1	0	0	0	0	0	0	0	2
Cluster11483	Proteobac	Deltap	Myxo	Polyangiaceae		0	0	0	0	1	0	0	0	0	0	0	1	2
Cluster11493	Proteobac	Alphaproteobacteria				0	0	0	0	0	1	0	0	0	0	1	0	2
Cluster11496	Gemmati	Gemm	Gem	Gemma	Gemmatimona	0	0	0	0	0	0	0	1	0	0	1	0	2
Cluster11548;size=7						0	0	0	0	0	0	1	0	0	0	0	1	2
Cluster11570;size=6						0	0	0	0	0	1	1	0	0	0	0	0	2
Cluster11591	Acidobact	Acidobacteria_Gp1				0	0	1	0	0	0	0	1	0	0	0	0	2
Cluster11602	Bacteroid	Sphing	Sphir	Chitinophagaceae		1	0	1	0	0	0	0	0	0	0	0	0	2
Cluster11609	Proteobac	Deltap	Myxo	Cystoba	Anaeromyxoba	0	0	0	0	0	0	0	0	1	1	0	0	2
Cluster11634	Acidobact	Acidobacteria_Gp2			Gp2	0	0	0	0	0	0	1	0	1	0	0	0	2
Cluster11656	Actinobac	Actinob	Actinomycetales			0	1	0	0	0	0	1	0	0	0	0	0	2
Cluster11658;size=4						0	0	0	0	0	0	0	2	0	0	0	0	2

Cluster11722	Proteobac	Gamm	Xantl	Xanthomonadaceae		0	0	0	0	0	0	0	0	2	0	0	0	2
Cluster11771	Proteobac	Alphap	Rhod	Rhodos	Telmatospirillu	0	0	0	0	0	0	0	0	0	2	0	0	2
Cluster11782	Proteobac	Betaproteobacteria				0	0	0	1	0	1	0	0	0	0	0	0	2
Cluster11789	Proteobac	Alphaproteobacteria				0	0	0	0	2	0	0	0	0	0	0	0	2
Cluster11808	Proteobac	Alphaproteobacteria				0	0	0	0	0	0	0	0	0	2	0	0	2
Cluster11825	Acidobact	Acidobacteria_Gp1				0	0	0	0	0	0	0	0	0	2	0	0	2
Cluster11889	Proteobac	Alphap	Rhod	Rhodospirillaceae		0	0	0	0	1	0	1	0	0	0	0	0	2
Cluster11890	Proteobac	Alphap	Rhizobiales			0	1	0	1	0	0	0	0	0	0	0	0	2
Cluster11896	Acidobact	Acidobacteria_Gp3				1	0	0	0	0	0	0	0	0	0	0	1	2
Cluster11908;size=17						0	0	0	0	0	0	0	1	1	0	0	0	2
Cluster11909	Acidobact	Acidobacteria_Gp1			Gp1	0	0	0	0	1	0	0	0	1	0	0	0	2
Cluster11931	Proteobac	Gamm	Xantl	Sinobac	Steroidobacter	0	1	0	0	0	0	0	0	0	1	0	0	2
Cluster11949	Proteobac	Gammaproteobacteria				0	0	0	0	0	0	0	0	0	1	1	0	2
Cluster11971	Proteobac	Alphaproteobacteria				0	0	1	0	0	0	0	0	0	0	0	1	2
Cluster11989	Acidobact	Acidobacteria_Gp2			Gp2	0	0	0	0	1	0	0	1	0	0	0	0	2
Cluster12008	Acidobact	Acidobacteria_Gp3			Bryobacter	0	0	1	0	1	0	0	0	0	0	0	0	2
Cluster12029	Acidobact	Acidobacteria_Gp2			Gp2	0	0	1	1	0	0	0	0	0	0	0	0	2
Cluster12082	Acidobact	Acidobacteria_Gp2			Gp2	0	0	0	0	0	0	0	0	1	0	1	0	2
Cluster12104	Proteobac	Deltaproteobacteria				0	0	0	1	0	0	1	0	0	0	0	0	2
Cluster12117	Verrucom	Opituta	Opitu	Opitutaceae		2	0	0	0	0	0	0	0	0	0	0	0	2
Cluster12127	Proteobac	Alphap	Rhizo	Bradyrh	Bradyrhizobium	0	0	1	0	0	0	0	0	1	0	0	0	2
Cluster12185	Acidobact	Acidobacteria_Gp1			Gp1	1	0	0	0	0	0	1	0	0	0	0	0	2
Cluster12259	Proteobac	Alphap	Rhod	Acetobacteraceae		0	0	1	0	0	0	0	0	1	0	0	0	2
Cluster12281	Proteobac	Alphap	Rhizo	Bradyrh	Agromonas	1	0	1	0	0	0	0	0	0	0	0	0	2
Cluster12286	Bacteroid	Sphing	Sphir	Chitinophagaceae		0	0	0	0	1	1	0	0	0	0	0	0	2
Cluster12300	Acidobact	Acidobacteria_Gp2			Gp2	0	0	0	0	0	0	0	1	0	0	0	1	2
Cluster12310	Acidobact	Acidobacteria_Gp1				0	0	2	0	0	0	0	0	0	0	0	0	2
Cluster12365	Verrucom	Opituta	Opitu	Opituta	Opitutus	0	0	1	0	0	0	0	0	0	0	0	1	2
Cluster12366	Chloroflex	Ktedor	Ktedo	Ktedono	Ktedonobacter	0	2	0	0	0	0	0	0	0	0	0	0	2

Cluster1243	Acidobact	Acidobacteria_Gp2			Gp2	0	0	0	0	0	0	2	0	0	0	0	0	2
Cluster1245	Acidobact	Acidobacteria_Gp1			Gp1	0	0	1	0	0	0	0	0	1	0	0	0	2
Cluster1254	Proteobac	Betaproteobacteria				0	0	0	0	0	0	0	0	0	0	1	1	2
Cluster1255	Proteobac	Alphap	Rhizobiales			0	1	0	0	0	0	0	0	0	0	1	0	2
Cluster1257	Acidobact	Acidobacteria_Gp1				0	0	2	0	0	0	0	0	0	0	0	0	2
Cluster1258	Proteobac	Gamm	Xantl	Sinobac	Steroidobacter	1	0	0	0	0	0	0	0	0	0	1	0	2
Cluster12594;size=21						0	0	1	1	0	0	0	0	0	0	0	0	2
Cluster1259	Actinobac	Actinobacteria				1	0	0	0	0	0	0	0	0	0	1	0	2
Cluster1276	Proteobac	Deltap	Bdell	Bdellovi	Vampirovibrio	1	1	0	0	0	0	0	0	0	0	0	0	2
Cluster12791	OD1				OD1_genera_i	1	0	0	0	0	0	0	0	0	0	1	0	2
Cluster1280	Acidobact	Acidobacteria_Gp1			Edaphobacter	1	0	1	0	0	0	0	0	0	0	0	0	2
Cluster1282	Proteobac	Alphap	Caulc	Caulobа	Caulobacter	0	0	0	0	0	0	0	0	0	2	0	0	2
Cluster1286	Acidobact	Acidobacteria_Gp2			Gp2	0	0	1	1	0	0	0	0	0	0	0	0	2
Cluster12888;size=2						0	0	1	0	0	0	1	0	0	0	0	0	2
Cluster12945;size=10						0	0	0	0	0	0	2	0	0	0	0	0	2
Cluster1297	Chlamydia	Chlamy	Chlar	Parachlamydiaceae		1	0	0	0	0	0	0	0	0	0	1	0	2
Cluster1298	Acidobact	Acidobacteria_Gp2			Gp2	0	0	0	0	0	0	1	0	0	0	0	1	2
Cluster1300	Proteobac	Deltap	Myxc	Polyangiaceae		2	0	0	0	0	0	0	0	0	0	0	0	2
Cluster1301	Proteobac	Gamm	Xantl	Sinobac	Steroidobacter	1	0	1	0	0	0	0	0	0	0	0	0	2
Cluster1301	Proteobac	Alphap	Rhizobiales			1	0	1	0	0	0	0	0	0	0	0	0	2
Cluster1306	Acidobact	Acidobacteria_Gp2			Gp2	0	0	0	0	0	1	0	0	0	0	1	0	2
Cluster1311	Acidobact	Acidobacteria_Gp1			Granulicella	0	1	1	0	0	0	0	0	0	0	0	0	2
Cluster13181	Proteobacteria					2	0	0	0	0	0	0	0	0	0	0	0	2
Cluster1319	Acidobact	Acidobacteria_Gp1				0	0	0	1	0	0	0	0	1	0	0	0	2
Cluster13191	Acidobact	Acidobacteria_Gp2			Gp2	0	0	0	1	0	0	0	0	0	0	1	0	2
Cluster13196;size=12						0	2	0	0	0	0	0	0	0	0	0	0	2
Cluster1320	Acidobact	Acidobacteria_Gp1			Gp1	0	0	1	0	0	0	0	0	0	1	0	0	2
Cluster1329	Proteobac	Alphap	Caulc	Caulobа	Phenylobacter	0	0	2	0	0	0	0	0	0	0	0	0	2
Cluster1330	Acidobact	Acidobacteria_Gp1			Edaphobacter	0	0	2	0	0	0	0	0	0	0	0	0	2

Cluster13322;size=13						1	0	0	0	0	0	0	0	0	0	1	0	2
Cluster13371	Verrucom	Opitutae				0	0	0	0	0	0	1	0	0	0	0	1	2
Cluster13374	Acidobact	Acidobacteria_Gp2			Gp2	0	0	0	0	1	0	0	0	0	0	0	1	2
Cluster13378	Acidobact	Acidobacteria_Gp15			Gp15	1	0	0	0	1	0	0	0	0	0	0	0	2
Cluster13428	Actinobac	Actinol	Acidi	Acidimi	Aciditerrimona	0	0	0	0	1	0	0	0	0	0	1	0	2
Cluster13506;size=3						0	1	0	0	0	0	0	0	0	0	0	1	2
Cluster13507	Acidobact	Acidobacteria_Gp1			Granulicella	0	0	0	1	0	0	0	0	0	1	0	0	2
Cluster13566	Acidobact	Acidobacteria_Gp2			Gp2	0	0	0	1	1	0	0	0	0	0	0	0	2
Cluster13579	Proteobac	Deltap	Bdell	Bdellovi	Bdellovibrio	0	1	0	0	1	0	0	0	0	0	0	0	2
Cluster13609	Acidobacteria					0	0	0	0	0	0	0	1	1	0	0	0	2
Cluster13613	Bacteroid	Sphing	Sphir	Chitinophagaceae		1	0	0	0	1	0	0	0	0	0	0	0	2
Cluster13654	Proteobac	Deltap	Myxococcales			2	0	0	0	0	0	0	0	0	0	0	0	2
Cluster13658	Acidobact	Acidobacteria_Gp3			Gp3	0	0	0	0	0	0	0	0	0	0	1	1	2
Cluster13708	Proteobac	Alphap	Rhod	Acetobacteraceae		0	0	0	1	0	0	0	0	0	1	0	0	2
Cluster13714;size=3						0	0	0	0	1	0	1	0	0	0	0	0	2
Cluster13720	Acidobact	Acidobacteria_Gp3			Gp3	0	1	1	0	0	0	0	0	0	0	0	0	2
Cluster13805;size=13						0	0	0	0	0	0	0	0	0	0	1	1	2
Cluster13824	Acidobact	Acidobacteria_Gp1			Gp1	0	0	1	0	0	0	1	0	0	0	0	0	2
Cluster13845	Proteobac	Deltaproteobacteria				0	0	0	0	0	0	0	0	0	0	0	2	2
Cluster13871	Acidobact	Acidobacteria_Gp1			Gp1	0	0	1	0	0	1	0	0	0	0	0	0	2
Cluster13910	Acidobact	Acidobacteria_Gp3			Gp3	0	0	0	1	0	0	0	0	0	0	0	1	2
Cluster13914	Proteobac	Alphaproteobacteria				0	0	0	0	0	1	0	0	0	0	0	1	2
Cluster13921	Proteobac	Deltap	Myxococcales			0	0	0	0	0	0	1	0	0	0	1	0	2
Cluster13996;size=8						0	0	1	0	0	0	1	0	0	0	0	0	2
Cluster14054	Proteobac	Deltap	Myxococcales			1	0	0	0	0	0	0	0	1	0	0	0	2
Cluster14070	Firmicutes	Bacilli	Bacill	Paeniba	Paenibacillus	0	0	0	0	0	1	0	0	1	0	0	0	2
Cluster14107	Acidobact	Acidobacteria_Gp1			Granulicella	1	0	0	1	0	0	0	0	0	0	0	0	2
Cluster14163	Acidobact	Acidobacteria_Gp1				0	0	0	0	0	1	0	0	1	0	0	0	2
Cluster14208	Acidobact	Acidobacteria_Gp2			Gp2	0	0	1	0	0	0	0	0	1	0	0	0	2

Cluster14233;size=10						0	0	0	1	1	0	0	0	0	0	0	0	2
Cluster14238	Proteobac	Alphap	Rhizobiales			0	0	0	0	0	0	0	1	1	0	0	0	2
Cluster14274	Acidobact	Acidobacteria_Gp3			Gp3	0	0	0	1	0	0	0	0	1	0	0	0	2
Cluster14299;size=11						0	1	0	0	0	0	0	1	0	0	0	0	2
Cluster14305	Acidobact	Acidobacteria_Gp1			Gp1	0	0	0	2	0	0	0	0	0	0	0	0	2
Cluster14380	Proteobac	Betapr	Burkholderiales			0	0	0	0	0	1	0	1	0	0	0	0	2
Cluster14407;size=9						0	0	0	2	0	0	0	0	0	0	0	0	2
Cluster14409	Acidobact	Acidobacteria_Gp1			Gp1	0	0	0	0	0	0	0	0	0	1	0	1	2
Cluster14435	Proteobac	Alphap	Rhodospirillales			0	0	0	1	0	0	1	0	0	0	0	0	2
Cluster14452	Acidobact	Acidobacteria_Gp1			Gp1	1	0	0	0	0	0	0	0	0	0	1	0	2
Cluster14545	Acidobact	Acidobacteria_Gp1				0	0	2	0	0	0	0	0	0	0	0	0	2
Cluster14566	Acidobact	Acidobacteria_Gp2			Gp2	0	0	0	0	0	0	0	1	1	0	0	0	2
Cluster14619	Acidobact	Acidobacteria_Gp1				0	0	0	0	0	0	0	0	2	0	0	0	2
Cluster14635	TM7				TM7_genera_i	0	0	0	0	0	1	1	0	0	0	0	0	2
Cluster14681	Proteobac	Deltap	Myxococcales			0	0	1	0	0	0	0	0	0	0	1	0	2
Cluster14684	Acidobact	Acidobacteria_Gp3			Gp3	0	1	0	0	0	0	1	0	0	0	0	0	2
Cluster14695	Acidobact	Acidobacteria_Gp1			Gp1	0	0	0	0	0	0	0	0	1	0	0	1	2
Cluster14702	Acidobact	Acidobacteria_Gp2			Gp2	0	0	1	0	0	0	1	0	0	0	0	0	2
Cluster14723	Acidobact	Acidobacteria_Gp1			Gp1	0	0	0	0	0	0	2	0	0	0	0	0	2
Cluster14734	Acidobact	Acidobacteria_Gp3			Bryobacter	0	0	1	1	0	0	0	0	0	0	0	0	2
Cluster14735	Proteobac	Alphap	Rhizobiales			2	0	0	0	0	0	0	0	0	0	0	0	2
Cluster14776	Verrucom	Spartobacteria			Spartobacteria	0	0	0	1	0	0	1	0	0	0	0	0	2
Cluster14791	Acidobact	Acidobacteria_Gp2			Gp2	0	0	0	1	0	0	0	1	0	0	0	0	2
Cluster14792	Acidobact	Acidobacteria_Gp1			Gp1	0	0	1	1	0	0	0	0	0	0	0	0	2
Cluster14805	Actinobac	Actinol	Actin	Mycoba	Mycobacteriur	0	0	0	0	0	0	0	1	0	1	0	0	2
Cluster14816	Actinobac	Actinol	Actinomycetales			0	0	0	0	0	1	1	0	0	0	0	0	2
Cluster14822	Proteobacteria					1	0	0	0	0	0	0	0	0	0	0	1	2
Cluster14823	Proteobac	Betapr	Burkl	Burkhol	Pandoraea	1	0	0	0	0	0	0	0	1	0	0	0	2
Cluster14835	Verrucom	Opituta	Opitu	Opituta	Opitutus	0	0	0	0	0	0	0	0	0	0	0	2	2

Cluster14866	Proteobac	Gammaproteobacteria				0	0	1	0	0	0	1	0	0	0	0	0	2
Cluster14922	Acidobact	Acidobacteria_Gp2			Gp2	1	0	0	0	0	0	0	0	0	0	1	0	2
Cluster14998	Chlamydia	Chlamy	Chlar	Parachla	Parachlamydia	0	1	0	0	0	0	0	0	0	1	0	0	2
Cluster15020	Proteobac	Alphap	Alphaproteob		Rhizomicrobiu	0	0	0	0	0	0	0	0	1	0	0	1	2
Cluster15048	Acidobact	Acidobacteria_Gp1			Edaphobacter	0	1	0	0	1	0	0	0	0	0	0	0	2
Cluster15122	Proteobac	Betaproteobacteria				0	0	0	0	0	0	0	0	1	0	0	1	2
Cluster15144	Chlamydia	Chlamy	Chlar	Parachlamydiaceae		0	1	0	1	0	0	0	0	0	0	0	0	2
Cluster15147	Acidobact	Acidobacteria_Gp2			Gp2	0	0	0	0	0	0	0	0	0	0	1	1	2
Cluster15167	Proteobac	Deltap	Myxococcales			0	1	0	0	0	0	0	1	0	0	0	0	2
Cluster15177	Acidobact	Acidobacteria_Gp2			Gp2	0	0	0	0	0	0	0	1	0	0	1	0	2
Cluster15251	Acidobact	Acidobacteria_Gp1			Gp1	2	0	0	0	0	0	0	0	0	0	0	0	2
Cluster15305	Proteobacteria					2	0	0	0	0	0	0	0	0	0	0	0	2
Cluster15319	Proteobac	Deltap	Myxococcales			0	0	1	0	0	0	0	1	0	0	0	0	2
Cluster15342	Acidobact	Acidobacteria_Gp1				2	0	0	0	0	0	0	0	0	0	0	0	2
Cluster15359	Proteobac	Gamm	Xanth	Sinobacteraceae		2	0	0	0	0	0	0	0	0	0	0	0	2
Cluster15363	Acidobact	Acidobacteria_Gp1				0	0	0	0	0	0	0	0	0	1	0	1	2
Cluster15364	Acidobact	Acidobacteria_Gp1			Gp1	0	0	0	0	0	0	1	1	0	0	0	0	2
Cluster15373	Acidobact	Acidobacteria_Gp2			Gp2	0	0	0	0	0	0	0	0	1	0	0	1	2
Cluster15384	Acidobact	Acidobacteria_Gp1				0	1	0	0	0	0	0	0	1	0	0	0	2
Cluster15411	Actinobac	Actinob	Actinomycetales			2	0	0	0	0	0	0	0	0	0	0	0	2
Cluster15446	TM7				TM7_genera_i	0	0	0	1	0	0	0	0	0	0	0	1	2
Cluster15448	Acidobact	Acidobacteria_Gp6			Gp6	0	0	0	0	0	0	1	0	0	0	1	0	2
Cluster15455	Acidobact	Acidobacteria_Gp1				0	0	2	0	0	0	0	0	0	0	0	0	2
Cluster15461	Proteobac	Alphap	Rhizobiales			0	0	0	0	1	0	0	0	0	0	0	1	2
Cluster15491	Actinobac	Actinob	Solirubrobacterales			0	0	0	1	0	0	0	0	0	0	1	0	2
Cluster15494	Acidobact	Acidobacteria_Gp1				0	0	0	0	1	0	1	0	0	0	0	0	2
Cluster15506	Proteobac	Deltaproteobacteria				1	1	0	0	0	0	0	0	0	0	0	0	2
Cluster15560	Acidobact	Acidobacteria_Gp3			Gp3	0	0	0	0	0	0	0	0	0	2	0	0	2
Cluster15567	Acidobact	Acidobacteria_Gp1			Gp1	0	0	0	0	1	0	0	1	0	0	0	0	2

Cluster15581	Actinobac	Actinol	Solirι	Solirubr	Solirubrobacte	0	1	0	0	0	0	0	1	0	0	0	0	2
Cluster15600	Chlamydia	Chlamy	Chlar	Parachlamydiaceae		0	0	0	0	0	0	2	0	0	0	0	0	2
Cluster15614	Acidobact	Acidobacteria_Gp1			Gp1	0	0	0	0	0	0	1	0	1	0	0	0	2
Cluster15635	Acidobact	Acidobacteria_Gp1			Gp1	0	0	0	0	2	0	0	0	0	0	0	0	2
Cluster15687	Proteobac	Alphaproteobacteria				0	0	0	0	0	0	1	1	0	0	0	0	2
Cluster15690	Acidobact	Acidobacteria_Gp1				0	0	0	1	0	0	0	0	0	1	0	0	2
Cluster15740	Proteobac	Alphap	Rhod	Acetobacteraceae		0	0	0	1	0	0	0	1	0	0	0	0	2
Cluster15751	Proteobac	Gamm	Legio	Coxiella	Aquicella	0	0	1	0	0	0	1	0	0	0	0	0	2
Cluster15772	Acidobact	Acidobacteria_Gp2			Gp2	0	0	0	0	0	0	0	1	0	0	1	0	2
Cluster15774	Acidobact	Acidobacteria_Gp10			Gp10	0	1	0	1	0	0	0	0	0	0	0	0	2
Cluster15802;size=4						0	0	1	0	0	0	0	1	0	0	0	0	2
Cluster15833	Actinobac	Actinol	Actinomycetales			0	0	0	0	0	0	0	0	0	0	1	1	2
Cluster15846	Armatimc	Chthor	Chth	Chthon	Chthonomona	0	0	1	1	0	0	0	0	0	0	0	0	2
Cluster15847	Acidobact	Acidobacteria_Gp1				0	0	1	0	0	0	0	0	0	0	0	1	2
Cluster15848	Acidobact	Acidobacteria_Gp3			Gp3	0	0	0	1	0	0	0	0	0	0	0	1	2
Cluster15880	Acidobact	Acidobacteria_Gp3				0	1	1	0	0	0	0	0	0	0	0	0	2
Cluster15885	Acidobact	Acidobacteria_Gp2			Gp2	0	1	0	0	0	0	0	0	1	0	0	0	2
Cluster15888;size=7						0	0	0	1	0	0	0	1	0	0	0	0	2
Cluster15957;size=2						0	0	0	0	0	0	1	1	0	0	0	0	2
Cluster15962	Acidobact	Acidobacteria_Gp2			Gp2	0	0	0	0	0	0	1	0	0	0	1	0	2
Cluster15985	Proteobac	Alphaproteobacteria				0	1	0	0	0	1	0	0	0	0	0	0	2
Cluster16050	Proteobac	Alphap	Rhizc	Bradyrhizobiaceae		0	0	1	0	0	0	0	0	1	0	0	0	2
Cluster16121	Acidobact	Acidobacteria_Gp3			Bryobacter	0	0	0	0	0	1	0	0	1	0	0	0	2
Cluster16136	Proteobac	Alphap	Caulc	Cauloba	Phenylobacter	0	0	0	0	0	0	1	0	0	0	1	0	2
Cluster16145	Actinobac	Actinol	Solirι	Conexib	Conexibacter	0	0	0	1	1	0	0	0	0	0	0	0	2
Cluster16146	Proteobac	Alphap	Rhodospirillales			0	0	0	0	0	0	0	0	0	0	0	2	2
Cluster16147	Bacteroid	Sphing	Sphir	Chitinophagaceae		0	0	1	1	0	0	0	0	0	0	0	0	2
Cluster16149	Acidobact	Acidobacteria_Gp1				0	0	0	1	1	0	0	0	0	0	0	0	2
Cluster16184	Acidobact	Acidobacteria_Gp1				0	0	1	0	0	0	0	0	0	0	1	0	2

Cluster16207	Proteobac	Alphaproteobacteria				0	0	0	0	0	0	0	0	0	1	1	0	2
Cluster16212	Acidobact	Acidobacteria_Gp1				0	0	1	0	0	1	0	0	0	0	0	0	2
Cluster16216	Proteobac	Gamm	Legio	Coxiella	Coxiella	0	1	0	0	0	0	0	0	1	0	0	0	2
Cluster16274	Acidobact	Acidobacteria_Gp1			Gp1	0	0	1	1	0	0	0	0	0	0	0	0	2
Cluster16291	Acidobact	Acidobacteria_Gp1				0	0	0	0	0	1	0	0	0	1	0	0	2
Cluster16308	Acidobact	Acidobacteria_Gp2			Gp2	1	0	0	0	0	0	0	0	0	0	1	0	2
Cluster16313	Proteobac	Gammaproteobacteria				0	0	0	0	2	0	0	0	0	0	0	0	2
Cluster16322	Proteobac	Alphap	Alphaproteob		Rhizomicrobiu	0	0	1	0	0	0	0	1	0	0	0	0	2
Cluster16457	Acidobacteria					0	0	0	0	0	0	0	0	1	0	1	0	2
Cluster16470	Proteobac	Gammaproteobacteria				0	0	0	0	2	0	0	0	0	0	0	0	2
Cluster16507	Acidobact	Acidobacteria_Gp3				0	0	0	0	0	2	0	0	0	0	0	0	2
Cluster16655	Proteobac	Betapr	Burkl	Burkhol	Burkholderia	0	0	1	0	1	0	0	0	0	0	0	0	2
Cluster16725	Verrucom	Opitut	Opitu	Opituta	Opitutus	0	0	0	0	0	0	0	0	0	1	0	1	2
Cluster16735	Proteobac	Alphap	Rhod	Acetobacteraceae		0	0	0	0	0	0	0	0	0	0	2	0	2
Cluster16764	Acidobact	Acidobacteria_Gp2			Gp2	0	0	0	1	0	0	0	0	1	0	0	0	2
Cluster16823	Acidobact	Acidobacteria_Gp1				0	0	1	0	0	0	0	0	0	0	0	1	2
Cluster16829	Acidobact	Acidobacteria_Gp1			Gp1	0	0	0	0	0	0	0	0	0	2	0	0	2
Cluster16851	Acidobact	Acidobacteria_Gp3			Gp3	0	0	0	0	1	0	0	0	1	0	0	0	2
Cluster16960;size=10						0	0	0	1	0	0	1	0	0	0	0	0	2
Cluster16966	Proteobac	Alphap	Rhizobiales			0	0	0	0	0	0	1	1	0	0	0	0	2
Cluster17012	TM7				TM7_genera_i	0	0	0	0	1	0	0	0	1	0	0	0	2
Cluster17172	Acidobact	Acidobacteria_Gp5			Gp5	0	0	0	1	0	0	0	0	1	0	0	0	2
Cluster17217	Acidobact	Acidobacteria_Gp2			Gp2	0	0	0	0	1	0	0	0	1	0	0	0	2
Cluster17225	Proteobac	Deltaproteobacteria				0	0	0	0	0	2	0	0	0	0	0	0	2
Cluster17235	Actinobac	Actinol	Actin	Actinos	Actinospica	0	0	0	1	0	0	0	0	0	0	1	0	2
Cluster17288	Proteobacteria					0	0	0	0	0	0	2	0	0	0	0	0	2
Cluster17315	Acidobact	Acidobacteria_Gp1			Gp1	0	0	0	1	0	0	0	1	0	0	0	0	2
Cluster17336	Bacteroid	Sphing	Sphir	Chitinophagaceae		0	0	0	0	2	0	0	0	0	0	0	0	2
Cluster17338	Verrucom	Subdivision3			Subdivision3_g	0	0	0	1	1	0	0	0	0	0	0	0	2

Cluster17401	Acidobact	Acidobacteria_Gp2			Gp2	0	0	0	0	1	0	0	0	0	0	1	0	2
Cluster17449	Proteobacteria					0	1	0	0	0	0	0	0	0	1	0	0	2
Cluster17452	Actinobac	Actinol	Solirubrobacterales			0	0	0	0	0	0	0	0	0	0	1	1	2
Cluster17460	Proteobac	Betapr	Burkl	Burkhol	Burkholderia	0	0	1	1	0	0	0	0	0	0	0	0	2
Cluster17469	Acidobact	Acidobacteria_Gp1			Gp1	0	0	1	0	1	0	0	0	0	0	0	0	2
Cluster17649	Proteobac	Alphaproteobacteria				0	0	1	0	0	0	0	0	0	0	1	0	2
Cluster17671	Acidobact	Acidobacteria_Gp3			Gp3	0	0	0	0	0	0	0	0	1	1	0	0	2
Cluster17736	Proteobac	Alphap	Rhizc	Bradyrhizobiaceae		1	0	0	0	0	0	0	0	0	0	0	1	2
Cluster17739	Acidobact	Acidobacteria_Gp3			Gp3	1	0	0	0	0	0	0	0	1	0	0	0	2
Cluster17743	Acidobact	Acidobacteria_Gp3				0	0	0	0	0	0	0	1	1	0	0	0	2
Cluster17759	Acidobacteria					0	0	2	0	0	0	0	0	0	0	0	0	2
Cluster17804	TM7				TM7_genera_i	0	0	0	0	0	0	0	0	1	0	1	0	2
Cluster17816	Acidobact	Acidobacteria_Gp2			Gp2	0	0	0	1	0	0	0	1	0	0	0	0	2
Cluster17825;size=4						1	0	0	0	0	0	0	0	0	0	1	0	2
Cluster17826	Verrucom	Opituta	Opitu	Opituta	Opitutus	1	0	0	0	1	0	0	0	0	0	0	0	2
Cluster17834	Acidobact	Acidobacteria_Gp2			Gp2	0	0	0	0	0	0	0	0	0	0	2	0	2
Cluster17837	Acidobact	Acidobacteria_Gp2			Gp2	0	0	0	0	0	0	1	1	0	0	0	0	2
Cluster17849	Proteobac	Gamm	Xantl	Sinobac	Steroidobacter	0	0	1	0	1	0	0	0	0	0	0	0	2
Cluster17856	Proteobac	Betaproteobacteria				0	0	0	0	0	0	0	2	0	0	0	0	2
Cluster17871	Proteobac	Alphap	Caulc	Caulobacteraceae		0	0	1	0	0	0	0	1	0	0	0	0	2
Cluster17878;size=12						0	0	0	1	0	0	0	1	0	0	0	0	2
Cluster17885	Verrucom	Opituta	Opitu	Opituta	Opitutus	2	0	0	0	0	0	0	0	0	0	0	0	2
Cluster17889	Actinobac	Actinol	Actinomycetales			0	0	0	1	0	0	0	0	0	0	1	0	2
Cluster17943	Acidobact	Acidobacteria_Gp3			Gp3	0	0	0	0	0	0	0	1	0	1	0	0	2
Cluster17949	Acidobact	Acidobacteria_Gp3				0	0	0	0	0	0	0	0	0	0	2	0	2
Cluster17982	Verrucom	Opituta	Opitu	Opituta	Opitutus	0	0	0	0	0	0	0	0	0	1	0	1	2
Cluster18012	Verrucom	Opituta	Opitu	Opituta	Opitutus	0	0	0	0	0	1	0	0	1	0	0	0	2
Cluster18015	Actinobac	Actinol	Solirι	Conexib	Conexibacter	0	0	0	0	0	0	0	0	0	2	0	0	2
Cluster18095	Acidobact	Acidobacteria_Gp2			Gp2	0	0	0	1	0	0	0	0	0	0	1	0	2

Cluster18105	Proteobac	Betapr	Burkl	Burkholderiaceae		0	1	0	0	0	0	0	1	0	0	0	0	2
Cluster18154	Acidobact	Acidobacteria_Gp1				0	0	2	0	0	0	0	0	0	0	0	0	2
Cluster18177	Acidobact	Acidobacteria_Gp2			Gp2	0	0	1	1	0	0	0	0	0	0	0	0	2
Cluster18178	Acidobact	Acidobacteria_Gp2			Gp2	0	0	0	0	0	0	0	0	1	0	1	0	2
Cluster18207	Acidobact	Acidobacteria_Gp2			Gp2	0	0	0	0	1	0	0	0	0	0	1	0	2
Cluster18215	Acidobact	Acidobacteria_Gp3			Gp3	0	0	1	0	0	0	0	1	0	0	0	0	2
Cluster18254	Acidobact	Acidobacteria_Gp2			Gp2	0	0	0	0	0	0	1	1	0	0	0	0	2
Cluster18256	Acidobact	Acidobacteria_Gp2			Gp2	0	0	1	0	0	0	1	0	0	0	0	0	2
Cluster18273	Proteobac	Alphap	Rhod	Acetobacteraceae		0	0	0	0	0	0	0	0	0	0	0	2	2
Cluster18283	Acidobact	Acidobacteria_Gp2			Gp2	0	0	0	0	0	0	0	1	1	0	0	0	2
Cluster18285	Acidobact	Acidobacteria_Gp2			Gp2	0	0	0	2	0	0	0	0	0	0	0	0	2
Cluster18286	Acidobact	Acidobacteria_Gp2			Gp2	0	0	0	0	0	0	0	1	1	0	0	0	2
Cluster18289;size=2						0	1	0	0	0	0	0	0	0	0	1	0	2
Cluster18290	Acidobact	Acidobacteria_Gp3			Gp3	0	0	1	0	1	0	0	0	0	0	0	0	2
Cluster18299	Proteobac	Alphap	Rhizobiales			0	0	0	0	0	0	0	1	1	0	0	0	2
Cluster18308	Acidobact	Acidobacteria_Gp1				0	0	1	1	0	0	0	0	0	0	0	0	2
Cluster18309	Acidobact	Acidobacteria_Gp3			Gp3	0	1	0	1	0	0	0	0	0	0	0	0	2
Cluster18396	Acidobact	Acidobacteria_Gp2			Gp2	0	0	0	1	0	0	0	0	1	0	0	0	2
Cluster18405	Acidobact	Acidobacteria_Gp2			Gp2	1	0	0	0	0	0	0	0	0	0	1	0	2
Cluster18422	Acidobact	Acidobacteria_Gp2			Gp2	0	0	1	0	0	0	0	0	0	1	0	0	2
Cluster18428	Acidobact	Acidobacteria_Gp3			Gp3	0	0	1	0	0	0	0	1	0	0	0	0	2
Cluster18439	TM7				TM7_genera_i	0	0	0	0	0	0	0	1	1	0	0	0	2
Cluster18484	Acidobact	Acidobacteria_Gp2			Gp2	0	0	1	1	0	0	0	0	0	0	0	0	2
Cluster18485	Proteobac	Betapr	Burkl	Burkhol	Burkholderia	1	0	0	0	0	0	0	0	0	0	0	1	2
Cluster18496	Acidobact	Acidobacteria_Gp1				0	0	0	0	1	0	1	0	0	0	0	0	2
Cluster18515	Acidobact	Acidobacteria_Gp2			Gp2	0	0	0	1	0	0	0	0	0	0	0	1	2
Cluster18527	Acidobact	Acidobacteria_Gp1				0	0	0	0	0	0	0	0	0	1	1	0	2
Cluster18549	Proteobac	Alphap	Alphaproteol		Rhizomicrobiu	0	0	0	1	0	1	0	0	0	0	0	0	2
Cluster18570	Acidobact	Acidobacteria_Gp2			Gp2	1	0	0	0	0	0	0	1	0	0	0	0	2

Cluster18576	Actinobac	Actinol	Actinomycetales			0	0	0	0	0	0	1	0	0	0	1	0	2
Cluster18617	Acidobact	Acidobacteria_Gp3			Gp3	0	0	0	1	0	0	0	0	0	0	0	1	2
Cluster18632	Acidobact	Acidobacteria_Gp2			Gp2	0	0	1	1	0	0	0	0	0	0	0	0	2
Cluster18665	Proteobacteria					0	0	0	1	0	0	0	0	1	0	0	0	2
Cluster18675	Acidobact	Acidobacteria_Gp1				0	0	0	1	0	0	0	0	0	1	0	0	2
Cluster18695	Proteobac	Alphap	Rhizobiales			0	0	0	1	0	0	0	0	0	0	1	0	2
Cluster18717	Acidobact	Acidobacteria_Gp3				0	2	0	0	0	0	0	0	0	0	0	0	2
Cluster18719	Acidobact	Acidobacteria_Gp2			Gp2	0	0	0	0	1	0	0	1	0	0	0	0	2
Cluster18738	Acidobact	Acidobacteria_Gp2			Gp2	0	0	0	1	0	0	0	0	0	0	0	1	2
Cluster18739;size=3						0	0	0	1	0	0	0	0	0	0	0	1	2
Cluster18775	Proteobac	Gammaproteobacteria				1	0	0	0	0	0	0	0	0	0	0	1	2
Cluster18782	Proteobac	Gammaproteobacteria				0	0	0	0	1	0	0	0	1	0	0	0	2
Cluster18840	Chlamydi	Chlamy	Chlar	Parachlamydiaceae		0	1	0	0	0	0	0	0	0	0	0	1	2
Cluster18890	Proteobac	Gamm	Xantl	Xanthomonadaceae		0	1	1	0	0	0	0	0	0	0	0	0	2
Cluster18912	Acidobact	Acidobacteria_Gp1				0	0	0	0	0	0	0	0	0	2	0	0	2
Cluster18924	Acidobact	Acidobacteria_Gp2			Gp2	0	0	0	1	0	0	0	0	0	1	0	0	2
Cluster18934;size=2						0	0	0	0	0	0	0	0	0	2	0	0	2
Cluster18935	Proteobac	Alphap	Rhodospirillales			0	0	0	0	0	0	0	1	0	0	0	1	2
Cluster18963	Proteobac	Betapr	Burkl	Comamonadaceae		0	0	0	0	0	0	0	2	0	0	0	0	2
Cluster18971;size=5						0	0	0	0	0	0	1	0	0	1	0	0	2
Cluster19008	Acidobact	Acidobacteria_Gp2			Gp2	1	0	0	0	0	0	0	0	0	0	1	0	2
Cluster19030	Proteobac	Betapr	Burkl	Burkhol	Burkholderia	1	0	0	0	0	0	1	0	0	0	0	0	2
Cluster19037	Acidobact	Acidobacteria_Gp2			Gp2	0	0	0	0	0	0	1	0	1	0	0	0	2
Cluster19050	Proteobac	Betaproteobacteria				0	0	0	0	0	1	0	1	0	0	0	0	2
Cluster19065	Proteobac	Alphap	Rhizobiales			0	1	0	0	0	0	0	0	0	0	1	0	2
Cluster19109	Acidobacteria					0	0	1	0	0	0	0	1	0	0	0	0	2
Cluster19114	Actinobac	Actinol	Solirı	Conexib	Conexibacter	0	0	1	0	0	0	0	0	0	1	0	0	2
Cluster19159	Planctomycetes					0	0	0	0	0	0	1	0	0	0	1	0	2
Cluster19164	Acidobact	Acidobacteria_Gp2			Gp2	0	0	0	0	0	1	0	0	0	1	0	0	2

Cluster19185	Acidobact	Acidobacteria_Gp2			Gp2	1	0	0	0	0	0	0	1	0	0	0	0	2
Cluster19188	Proteobac	Alphaproteobacteria				1	0	0	0	0	0	0	1	0	0	0	0	2
Cluster19201	Acidobact	Acidobacteria_Gp3				0	0	1	0	0	0	0	0	0	0	0	1	2
Cluster19230	Acidobact	Acidobacteria_Gp2			Gp2	0	0	0	0	0	1	1	0	0	0	0	0	2
Cluster19235	Chloroflex	Ktedor	Ktedonobacterales			0	0	0	0	0	0	0	1	0	0	0	1	2
Cluster19244	Verrucom	Opitut	Opitu	Opituta	Opitutus	1	0	1	0	0	0	0	0	0	0	0	0	2
Cluster19254	Acidobact	Acidobacteria_Gp2			Gp2	0	0	0	0	0	0	0	2	0	0	0	0	2
Cluster19262	Proteobac	Alphap	Alphaproteob		Rhizomicrobiu	0	0	0	0	0	0	0	1	1	0	0	0	2
Cluster19264	Proteobac	Betaproteobacteria				1	0	0	0	1	0	0	0	0	0	0	0	2
Cluster19269	Acidobact	Acidobacteria_Gp2			Gp2	0	0	0	2	0	0	0	0	0	0	0	0	2
Cluster19306	Proteobac	Gammaproteobacteria				0	0	2	0	0	0	0	0	0	0	0	0	2
Cluster19307	Acidobact	Acidobacteria_Gp2			Gp2	2	0	0	0	0	0	0	0	0	0	0	0	2
Cluster19347	Proteobac	Alphaproteobacteria				0	0	0	0	0	0	0	0	0	0	2	0	2
Cluster19427;size=5						0	0	0	0	0	0	2	0	0	0	0	0	2
Cluster19432	Actinobac	Actinol	Solirubrobacterales			0	0	0	0	0	0	0	0	0	1	1	0	2
Cluster19447	Chloroflex	Ktedor	Ktedonobacterales			0	1	0	0	0	0	1	0	0	0	0	0	2
Cluster19448	Proteobacteria					0	0	0	0	0	0	2	0	0	0	0	0	2
Cluster19460	Proteobac	Gamm	Xantl	Sinobac	Steroidobacter	0	0	0	1	0	1	0	0	0	0	0	0	2
Cluster19467	Proteobac	Alphap	Rhizobiales			0	0	1	0	0	1	0	0	0	0	0	0	2
Cluster19478	Acidobact	Acidobacteria_Gp3			Bryobacter	0	0	0	0	2	0	0	0	0	0	0	0	2
Cluster19512	Proteobac	Alphap	Rhizc	Beijerinckiaceae		0	0	1	1	0	0	0	0	0	0	0	0	2
Cluster19516	Acidobact	Acidobacteria_Gp2			Gp2	1	0	0	0	0	0	0	0	1	0	0	0	2
Cluster19522	Proteobac	Alphaproteobacteria				1	0	0	0	1	0	0	0	0	0	0	0	2
Cluster19543	Acidobact	Acidobacteria_Gp1				0	0	1	0	1	0	0	0	0	0	0	0	2
Cluster19558	Chloroflex	Ktedor	Ktedonobacterales			0	0	0	0	0	0	1	1	0	0	0	0	2
Cluster19567	Acidobact	Acidobacteria_Gp2			Gp2	0	0	1	1	0	0	0	0	0	0	0	0	2
Cluster19595	Proteobac	Gammaproteobacteria				0	0	0	0	1	1	0	0	0	0	0	0	2
Cluster19675	Acidobact	Acidobacteria_Gp2			Gp2	1	0	1	0	0	0	0	0	0	0	0	0	2
Cluster19691	Acidobact	Acidobacteria_Gp1				0	0	0	0	1	0	1	0	0	0	0	0	2

Cluster19692	Acidobact	Acidobacteria_Gp2			Gp2	0	0	2	0	0	0	0	0	0	0	0	0	2
Cluster19694	Proteobac	Alphaproteobacteria				0	0	0	0	1	1	0	0	0	0	0	0	2
Cluster19700	Acidobact	Acidobacteria_Gp2			Gp2	0	0	1	0	0	0	1	0	0	0	0	0	2
Cluster19736	Acidobact	Acidobacteria_Gp3			Bryobacter	0	0	0	0	0	0	2	0	0	0	0	0	2
Cluster19765	Proteobac	Betaproteobacteria				0	0	0	0	1	0	1	0	0	0	0	0	2
Cluster19795	Acidobacteria					0	0	1	1	0	0	0	0	0	0	0	0	2
Cluster19798	Acidobact	Acidobacteria_Gp1			Granulicella	0	0	1	1	0	0	0	0	0	0	0	0	2
Cluster19832	Acidobact	Acidobacteria_Gp2			Gp2	1	0	0	0	0	0	0	0	0	0	1	0	2
Cluster19851	Proteobac	Gammaproteobacteria				0	0	0	0	2	0	0	0	0	0	0	0	2
Cluster19864	Acidobact	Acidobacteria_Gp3			Gp3	0	0	0	1	1	0	0	0	0	0	0	0	2
Cluster19905;size=3						0	0	0	0	0	0	2	0	0	0	0	0	2
Cluster19916	Acidobact	Acidobacteria_Gp2			Gp2	0	0	0	0	2	0	0	0	0	0	0	0	2
Cluster19976	Acidobact	Acidobacteria_Gp3				0	0	1	0	1	0	0	0	0	0	0	0	2
Cluster19994	Acidobact	Acidobacteria_Gp2			Gp2	0	0	0	0	1	0	1	0	0	0	0	0	2
Cluster19999;size=3						2	0	0	0	0	0	0	0	0	0	0	0	2
Cluster20001	Proteobac	Alphap	Rhizo	Bradyrh	Bradyrhizobiur	0	0	0	1	1	0	0	0	0	0	0	0	2
Cluster20072	Acidobact	Acidobacteria_Gp2			Gp2	0	0	0	0	0	1	1	0	0	0	0	0	2
Cluster20080	Acidobact	Acidobacteria_Gp2			Gp2	0	0	1	1	0	0	0	0	0	0	0	0	2
Cluster20082	Acidobact	Acidobacteria_Gp3				1	0	0	1	0	0	0	0	0	0	0	0	2
Cluster20130	Acidobact	Acidobacteria_Gp3			Gp3	0	0	1	0	0	0	1	0	0	0	0	0	2
Cluster20252	Acidobact	Acidobacteria_Gp2			Gp2	0	0	0	2	0	0	0	0	0	0	0	0	2
Cluster20299	Verrucom	Opituta	Opitu	Opituta	Opitutus	0	0	0	0	0	2	0	0	0	0	0	0	2
Cluster20318	Proteobac	Gammaproteobacteria				0	1	0	0	0	1	0	0	0	0	0	0	2
Cluster20357	Proteobac	Gamm	Legio	Coxiella	Aquicella	0	0	0	2	0	0	0	0	0	0	0	0	2
Cluster20360	Proteobac	Betapr	Burkl	Burkhol	Burkholderia	0	0	1	1	0	0	0	0	0	0	0	0	2
Cluster20366	Acidobact	Acidobacteria_Gp3			Gp3	0	0	0	0	1	0	1	0	0	0	0	0	2
Cluster20397	Proteobac	Alphap	Rhod	Acetobacteraceae		0	0	0	0	0	1	0	0	0	1	0	0	2
Cluster20457	Acidobact	Acidobacteria_Gp2			Gp2	1	0	1	0	0	0	0	0	0	0	0	0	2
Cluster20485	Proteobacteria					0	1	0	0	1	0	0	0	0	0	0	0	2

Cluster20492	Acidobact	Acidobacteria_Gp1			Gp1	0	0	0	1	0	0	0	1	0	0	0	0	2
Cluster20511	Actinobac	Actinol	Solirubrobacterales			0	0	0	0	0	1	1	0	0	0	0	0	2
Cluster20665	Acidobact	Acidobacteria_Gp2			Gp2	2	0	0	0	0	0	0	0	0	0	0	0	2
Cluster20676	Acidobact	Acidobacteria_Gp1				0	0	0	0	0	0	1	0	0	1	0	0	2
Cluster20711	Proteobac	Gammaproteobacteria				0	0	0	1	0	0	0	0	0	0	1	0	2
Cluster20730	Bacteroid	Sphing	Sphir	Chitinophagaceae		2	0	0	0	0	0	0	0	0	0	0	0	2
Cluster20755	Proteobac	Gamm	Xantl	Xanthor	Rudaea	0	0	0	1	0	0	1	0	0	0	0	0	2
Cluster20756	Acidobact	Acidobacteria_Gp2			Gp2	1	0	0	0	0	0	1	0	0	0	0	0	2
Cluster20802	Proteobac	Gamm	Xanthomonadales			0	0	0	0	1	0	0	0	0	0	1	0	2
Cluster20877	Acidobact	Acidobacteria_Gp2			Gp2	0	0	0	1	0	0	1	0	0	0	0	0	2
Cluster20936	Proteobac	Gammaproteobacteria				0	1	0	0	1	0	0	0	0	0	0	0	2
Cluster20967;size=2						1	0	0	0	0	0	1	0	0	0	0	0	2
Cluster20979	Proteobac	Deltap	Myxococcales			2	0	0	0	0	0	0	0	0	0	0	0	2
Cluster20998	Acidobact	Acidobacteria_Gp2			Gp2	0	0	0	2	0	0	0	0	0	0	0	0	2
Cluster21027	Verrucom	Opitut	Opitu	Opituta	Opitutus	2	0	0	0	0	0	0	0	0	0	0	0	2
Cluster21030	Acidobact	Acidobacteria_Gp2			Gp2	1	0	0	0	0	0	1	0	0	0	0	0	2
Cluster21042	Acidobact	Acidobacteria_Gp2			Gp2	2	0	0	0	0	0	0	0	0	0	0	0	2
Cluster21217	Proteobac	Alphap	Rhizc	Bradyrhizobiaceae		0	0	0	0	0	0	2	0	0	0	0	0	2
Cluster21356	Proteobac	Alphap	Caulc	Cauloba	Phenylobacter	0	0	0	0	0	0	0	1	0	1	0	0	2
Cluster21430	Acidobact	Acidobacteria_Gp2			Gp2	0	0	0	0	0	0	1	0	0	0	0	1	2
Cluster21531;size=2						0	0	1	1	0	0	0	0	0	0	0	0	2
Cluster21667	Acidobact	Acidobacteria_Gp3			Gp3	1	0	0	0	0	0	0	0	0	0	1	0	2
Cluster21684;size=2						0	0	0	0	0	0	0	0	0	0	2	0	2
Cluster21738	Proteobacteria					0	0	0	0	0	0	0	0	0	0	0	2	2
Cluster21758	Acidobact	Acidobacteria_Gp2			Gp2	0	0	1	0	0	0	0	1	0	0	0	0	2
Cluster21761	Acidobact	Acidobacteria_Gp3			Gp3	1	0	0	0	0	1	0	0	0	0	0	0	2
Cluster21778;size=5						0	0	0	0	0	0	0	0	1	0	1	0	2
Cluster21779;size=2						0	0	0	1	0	0	0	0	0	0	0	1	2
Cluster21789	Acidobact	Acidobacteria_Gp2			Gp2	1	0	0	0	0	0	1	0	0	0	0	0	2

Cluster21811	Proteobac	Gamm	Xantl	Sinobac	Steroidobacter	1	0	0	1	0	0	0	0	0	0	0	0	2
Cluster21848	Acidobact	Acidobacteria_Gp1				0	1	0	0	0	0	1	0	0	0	0	0	2
Cluster21876	Actinobac	Actinol	Acidi	Acidimi	Aciditerrimona	0	0	1	0	0	0	1	0	0	0	0	0	2
Cluster21882	Proteobac	Alphaproteobacteria				0	0	0	1	0	0	1	0	0	0	0	0	2
Cluster21934	Actinobac	Actinol	Acidi	Acidimi	Aciditerrimona	0	0	0	2	0	0	0	0	0	0	0	0	2
Cluster21954	Proteobacteria					0	0	1	1	0	0	0	0	0	0	0	0	2
Cluster21956	Proteobac	Gammaproteobacteria				0	0	1	1	0	0	0	0	0	0	0	0	2
Cluster21990	Acidobact	Acidobacteria_Gp1				0	1	0	1	0	0	0	0	0	0	0	0	2
Cluster22060	Acidobact	Acidobacteria_Gp1			Granulicella	0	0	0	0	1	0	0	0	0	1	0	0	2
Cluster22069	Acidobact	Acidobacteria_Gp1				1	0	0	0	0	0	0	0	1	0	0	0	2
Cluster22097	Proteobacteria					0	0	0	0	0	0	0	1	0	0	0	1	2
Cluster22106	Acidobact	Acidobacteria_Gp2			Gp2	0	0	0	1	0	1	0	0	0	0	0	0	2
Cluster22126	Proteobac	Gammaproteobacteria				0	0	1	0	0	0	0	0	1	0	0	0	2
Cluster22132	Acidobacteria					0	0	1	0	0	0	0	0	0	0	0	1	2
Cluster22160	Acidobact	Acidobacteria_Gp1			Gp1	0	0	0	1	0	0	0	0	0	1	0	0	2
Cluster22215	Proteobac	Alphap	Caulc	Caulobacteraceae		0	0	1	0	0	0	0	0	0	1	0	0	2
Cluster22224	Actinobac	Actinol	Solirubrobacterales			0	0	0	0	0	1	0	0	1	0	0	0	2
Cluster22265	Acidobact	Acidobacteria_Gp3			Gp3	0	0	1	1	0	0	0	0	0	0	0	0	2
Cluster22268	Acidobact	Acidobacteria_Gp2			Gp2	0	0	0	0	0	0	0	0	0	0	0	2	2
Cluster22313;size=5						0	2	0	0	0	0	0	0	0	0	0	0	2
Cluster22337	Acidobacteria					0	0	0	0	0	0	0	1	0	0	0	1	2
Cluster22385	Proteobac	Alphaproteobacteria				0	0	1	0	0	1	0	0	0	0	0	0	2
Cluster22389	Proteobac	Alphap	Alphaproteol		Rhizomicrobiu	0	0	0	0	0	0	0	0	0	0	1	1	2
Cluster22397	Acidobact	Acidobacteria_Gp1				0	0	2	0	0	0	0	0	0	0	0	0	2
Cluster22406	Acidobact	Acidobacteria_Gp1				0	0	0	0	0	0	0	0	0	0	1	1	2
Cluster22419	Acidobact	Acidobacteria_Gp3			Gp3	0	2	0	0	0	0	0	0	0	0	0	0	2
Cluster22448	Proteobac	Alphaproteobacteria				0	0	1	0	0	0	1	0	0	0	0	0	2
Cluster22463	Acidobact	Acidobacteria_Gp1				0	0	0	0	1	0	0	0	0	1	0	0	2
Cluster22474	Acidobact	Acidobacteria_Gp1			Gp1	0	0	0	0	1	0	1	0	0	0	0	0	2

Cluster22488	Proteobac	Alphap	Rhod	Acetoba	Acidisphaera	0	0	2	0	0	0	0	0	0	0	0	0	2
Cluster22496	Acidobact	Acidobacteria_Gp1			Gp1	0	0	0	0	1	1	0	0	0	0	0	0	2
Cluster22506	Proteobac	Alphaproteobacteria				0	1	1	0	0	0	0	0	0	0	0	0	2
Cluster22533	Acidobact	Acidobacteria_Gp1				0	1	0	0	0	0	0	0	0	0	1	0	2
Cluster22537	Actinobac	Actinol	Acidimicrobiales			0	0	0	1	0	0	0	0	0	0	0	1	2
Cluster22545	Acidobact	Acidobacteria_Gp1				0	0	0	0	0	0	0	0	0	0	0	2	2
Cluster22551	Acidobact	Acidobacteria_Gp1			Gp1	0	2	0	0	0	0	0	0	0	0	0	0	2
Cluster22568	Actinobac	Actinol	Actin	Actinos	Actinospica	0	0	0	0	0	0	0	0	0	1	0	1	2
Cluster22606	Proteobac	Gammaproteobacteria				1	0	0	0	0	0	0	0	0	1	0	0	2
Cluster22608	Acidobact	Acidobacteria_Gp1			Acidobacteriur	1	0	0	0	0	0	0	0	0	0	1	0	2
Cluster22611	Acidobact	Acidobacteria_Gp1				1	0	0	0	0	0	0	0	0	0	1	0	2
Cluster22612	Acidobact	Acidobacteria_Gp1				1	0	0	0	1	0	0	0	0	0	0	0	2
Cluster22620	Actinobac	Actinol	Actin	Actinos	Actinospica	1	0	0	0	0	0	0	1	0	0	0	0	2
Cluster22638	Acidobact	Acidobacteria_Gp2			Gp2	1	0	0	0	0	0	0	0	0	1	0	0	2
Cluster22642	Proteobac	Gammaproteobacteria				1	0	0	0	0	0	1	0	0	0	0	0	2
Cluster22645	TM7				TM7_genera_i	2	0	0	0	0	0	0	0	0	0	0	0	2
Cluster22663	Proteobac	Alphap	Rhodospirillales			0	0	0	0	0	0	0	0	0	0	0	2	2
Cluster22670	Proteobac	Gammaproteobacteria				0	1	0	1	0	0	0	0	0	0	0	0	2
Cluster22685	Actinobac	Actinol	Acidimicrobiales			0	0	1	0	0	0	0	0	0	0	0	1	2
Cluster22745;size=2						0	2	0	0	0	0	0	0	0	0	0	0	2
Cluster22791	Proteobac	Gamm	Xantl	Sinobac	Steroidobacter	1	0	1	0	0	0	0	0	0	0	0	0	2
Cluster22797	Acidobacteria					0	0	1	0	0	0	0	0	0	1	0	0	2
Cluster22822	Acidobact	Acidobacteria_Gp1			Gp1	0	0	0	0	0	0	0	1	0	0	1	0	2
Cluster22890	Acidobacteria					0	0	0	0	0	0	0	2	0	0	0	0	2
Cluster22908	Acidobact	Acidobacteria_Gp2			Gp2	0	0	0	1	0	0	0	0	0	0	1	0	2
Cluster22980	Proteobac	Alphaproteobacteria				0	0	2	0	0	0	0	0	0	0	0	0	2
Cluster23002	Acidobact	Acidobacteria_Gp3			Bryobacter	0	0	1	0	0	0	1	0	0	0	0	0	2
Cluster23121	Chloroflex	Ktedonobacteria				0	0	0	0	0	0	0	0	0	0	0	2	2
Cluster23203	Acidobact	Acidobacteria_Gp1			Edaphobacter	0	0	1	1	0	0	0	0	0	0	0	0	2

Cluster23245	Proteobacteria					0	0	0	0	0	0	1	1	0	0	0	0	2
Cluster23284	Proteobac	Alphap	Rhod	Acetobacteraceae		0	0	0	0	0	0	1	0	0	0	1	0	2
Cluster23296	Acidobact	Acidobacteria_Gp1				0	0	0	0	1	0	1	0	0	0	0	0	2
Cluster23310	Acidobact	Acidobacteria_Gp3			Gp3	0	0	1	0	1	0	0	0	0	0	0	0	2
Cluster23312	Proteobac	Gammaproteobacteria				0	0	0	0	1	0	1	0	0	0	0	0	2
Cluster23379	Acidobact	Acidobacteria_Gp3				0	0	0	0	0	0	0	2	0	0	0	0	2
Cluster23391	Proteobac	Gammaproteobacteria				0	0	0	0	0	0	2	0	0	0	0	0	2
Cluster23408	Acidobacteria					0	0	0	0	0	0	2	0	0	0	0	0	2
Cluster23440	Proteobac	Gammaproteobacteria				0	0	0	0	1	1	0	0	0	0	0	0	2
Cluster23445;size=6						0	0	0	0	0	1	0	1	0	0	0	0	2
Cluster23465	Proteobac	Alphap	Rhizo	Bradyrhizobiaceae		0	0	0	0	0	0	2	0	0	0	0	0	2
Cluster23505	Proteobac	Gamm	Enter	Enterobacteriaceae		0	0	0	0	1	0	0	1	0	0	0	0	2
Cluster23564	Proteobac	Alphap	Rhod	Acetoba	Acidisphaera	0	0	0	0	0	1	1	0	0	0	0	0	2
Cluster23592	Actinobac	Actinol	Actin	Pseudonocardiaceae		0	1	0	0	1	0	0	0	0	0	0	0	2
Cluster23599	Acidobact	Acidobacteria_Gp1			Gp1	0	0	0	0	1	0	0	0	1	0	0	0	2
Cluster23644	Acidobact	Acidobacteria_Gp3			Gp3	0	0	0	0	0	1	0	0	0	1	0	0	2
Cluster23668	Proteobac	Gammaproteobacteria				0	0	0	0	1	1	0	0	0	0	0	0	2
Cluster23677	Proteobac	Betapr	Burkholderiales			0	0	0	0	0	0	0	1	1	0	0	0	2
Cluster23717;size=2						0	0	0	0	0	1	0	1	0	0	0	0	2
Cluster23765	Actinobac	Actinobacteria				0	0	0	1	0	1	0	0	0	0	0	0	2
Cluster23795	Proteobac	Gamm	Xantl	Sinobac	Steroidobacter	0	0	0	0	1	0	1	0	0	0	0	0	2
Cluster23824	Acidobact	Acidobacteria_Gp3			Gp3	0	0	0	0	0	0	2	0	0	0	0	0	2
Cluster23865;size=3						0	0	0	0	1	0	0	1	0	0	0	0	2
Cluster23870	Actinobac	Actinol	Acidi	Acidimi	Aciditerrimona	0	0	0	0	0	0	0	0	1	0	0	1	2
Cluster23912	Actinobac	Actinol	Acidi	Acidimi	Aciditerrimona	0	1	0	0	0	0	0	0	0	1	0	0	2
Cluster23989	Actinobac	Actinobacteria				0	0	1	0	0	0	0	0	0	0	1	0	2
Cluster24074	Proteobac	Betapr	Burkl	Burkhol	Burkholderia	0	0	0	0	0	0	0	0	0	2	0	0	2
Cluster24105	Proteobac	Gammaproteobacteria				0	0	0	0	0	0	0	0	0	2	0	0	2
Cluster24117	Proteobac	Alphaproteobacteria				0	0	0	0	2	0	0	0	0	0	0	0	2

Cluster24189	Actinobac	Actinob	Actinomycetales			0	0	0	0	0	0	0	1	0	0	1	0	2
Cluster24198	Actinobac	Actinob	Soliru	Conexib	Conexibacter	0	0	0	0	0	0	0	0	0	2	0	0	2
Cluster24269	Proteobac	Gammaproteobacteria				0	0	0	0	0	0	1	1	0	0	0	0	2
Cluster24293	Actinobac	Actinob	Actinomycetales			0	0	0	0	1	0	0	1	0	0	0	0	2
Cluster24372	Acidobact	Acidobacteria_Gp2			Gp2	0	0	0	0	1	0	0	1	0	0	0	0	2
Cluster24425	Actinobac	Actinobacteria				0	0	0	0	1	0	0	1	0	0	0	0	2
Cluster24449	Acidobact	Acidobacteria_Gp2			Gp2	0	0	0	0	0	0	1	1	0	0	0	0	2
Cluster24463	Proteobac	Gamm	Meth	Methylo	Methylobacter	0	0	0	0	0	1	0	0	0	0	1	0	2
Cluster24478	Actinobac	Actinob	Actinomycetales			0	0	1	0	0	0	1	0	0	0	0	0	2
Cluster24565	Proteobac	Alphap	Alphaproteob		Rhizomicrobiu	0	0	0	0	1	0	0	1	0	0	0	0	2
Cluster24572	Acidobact	Acidobacteria_Gp1			Gp1	0	0	0	0	0	0	0	1	0	0	0	1	2
Cluster24598	Actinobac	Actinob	Actin	Microbacteriaceae		0	1	0	0	0	0	0	0	0	0	0	1	2
Cluster24605	Bacteroid	Sphing	Sphin	Chitinophagaceae		0	0	1	0	0	0	0	1	0	0	0	0	2
Cluster24664	Proteobac	Gammaproteobacteria				0	1	0	0	0	0	0	0	0	0	0	1	2
Cluster24707	Proteobac	Alphaproteobacteria				0	0	0	0	0	0	0	0	0	1	1	0	2
Cluster24763	Actinobac	Actinob	Acidimicrobiales			0	1	0	0	0	0	0	0	0	0	1	0	2
Cluster24780	Acidobact	Acidobacteria_Gp1			Edaphobacter	0	0	0	2	0	0	0	0	0	0	0	0	2
Cluster24854	Proteobac	Gamm	Legio	Coxiella	Aquicella	0	1	0	0	0	0	1	0	0	0	0	0	2
Cluster24872	Acidobact	Acidobacteria_Gp1			Edaphobacter	0	0	0	0	0	0	0	0	1	0	1	0	2
Cluster24882	Acidobact	Acidobacteria_Gp2			Gp2	0	0	0	1	0	0	1	0	0	0	0	0	2
Cluster24962	Proteobac	Alphap	Rhizobiales			0	0	0	2	0	0	0	0	0	0	0	0	2
Cluster24987	Actinobac	Actinob	Actinomycetales			0	0	0	0	0	1	0	1	0	0	0	0	2
Cluster25008	Proteobacteria					0	0	0	1	0	0	0	0	0	0	0	1	2
Cluster25015	Bacteroid	Sphing	Sphin	Chitinophagaceae		0	1	0	0	0	0	0	0	0	1	0	0	2
Cluster25019;size=7						1	0	0	0	0	0	0	1	0	0	0	0	2
Cluster25054	Acidobact	Acidobacteria_Gp2			Gp2	1	0	0	1	0	0	0	0	0	0	0	0	2
Cluster25085;size=4						0	0	1	0	1	0	0	0	0	0	0	0	2
Cluster25092	Bacteroid	Sphing	Sphin	Chitinop	Sediminibacter	0	0	0	0	1	1	0	0	0	0	0	0	2
Cluster25094;size=8						0	0	0	1	1	0	0	0	0	0	0	0	2

Cluster25139	Proteobac	Gamm	Legio	Coxiella	Aquicella	0	0	0	0	0	0	0	0	1	0	0	1	2
Cluster25142	Acidobact	Acidobacteria_Gp2			Gp2	0	0	0	1	1	0	0	0	0	0	0	0	2
Cluster25191;size=4						0	0	1	0	0	0	0	0	0	0	1	0	2
Cluster25196	Proteobac	Gamm	Legio	Coxiella	Aquicella	0	0	0	0	0	2	0	0	0	0	0	0	2
Cluster25250	Proteobac	Alphap	Caulo	Caulobacteraceae		0	0	0	1	0	0	0	1	0	0	0	0	2
Cluster25256	Actinobac	Actinob	Actinomycetales			0	0	0	0	0	0	0	1	0	1	0	0	2
Cluster25314	Proteobac	Alphap	Rhod	Acetoba	Acidisphaera	0	0	2	0	0	0	0	0	0	0	0	0	2
Cluster25336	Proteobac	Alphap	Caulo	Caulobacteraceae		0	0	0	0	0	1	0	0	0	1	0	0	2
Cluster25405	Bacteroidetes					0	0	0	0	0	0	2	0	0	0	0	0	2
Cluster25438	Actinobac	Actinob	Actinomycetales			1	0	0	0	0	0	0	0	0	1	0	0	2
Cluster25569	Acidobact	Acidobacteria_Gp1			Gp1	0	0	0	0	0	0	0	0	2	0	0	0	2
Cluster25654	Acidobact	Acidobacteria_Gp3			Gp3	0	0	0	1	1	0	0	0	0	0	0	0	2
Cluster25672	Proteobac	Gamm	Legio	Coxiella	Aquicella	0	0	0	0	1	0	0	0	1	0	0	0	2
Cluster25747	Bacteroid	Sphing	Sphin	Sphingo	Mucilaginibact	0	1	0	0	0	0	0	0	0	1	0	0	2
Cluster25762;size=3						0	0	0	0	2	0	0	0	0	0	0	0	2
Cluster25776	Proteobac	Alphap	Rhod	Acetoba	Acidisphaera	0	0	0	0	0	0	0	0	0	0	2	0	2
Cluster25795	Acidobact	Acidobacteria_Gp3			Bryobacter	0	0	0	0	0	2	0	0	0	0	0	0	2
Cluster25911	Proteobac	Gammaproteobacteria				0	0	1	0	0	1	0	0	0	0	0	0	2
Cluster26025;size=3						0	0	1	0	1	0	0	0	0	0	0	0	2
Cluster26067	Actinobac	Actinob	Actinomycetales			1	0	0	0	0	0	0	0	0	0	1	0	2
Cluster26076	Bacteroid	Sphing	Sphingobacteriales			1	0	0	0	0	0	0	1	0	0	0	0	2
Cluster26147	Proteobac	Alphap	Caulo	Cauloba	Caulobacter	0	0	0	0	0	0	0	0	0	0	2	0	2
Cluster26236	Acidobact	Acidobacteria_Gp13			Gp13	0	1	0	0	0	0	0	0	0	0	0	1	2
Cluster26252	Proteobac	Gammaproteobacteria				0	0	0	0	0	0	0	0	0	2	0	0	2
Cluster26268	Proteobac	Gammaproteobacteria				0	0	0	0	1	1	0	0	0	0	0	0	2
Cluster26348	Acidobact	Acidobacteria_Gp13			Gp13	0	0	0	0	0	0	0	0	1	0	0	1	2
Cluster26352	Actinobac	Actinob	Actinomycetales			0	0	1	0	0	0	0	0	0	1	0	0	2
Cluster26367	Proteobac	Alphaproteobacteria				0	0	1	0	0	0	0	0	1	0	0	0	2
Cluster26368	Proteobac	Betapr	Burkh	Burkhol	Xylophilus	2	0	0	0	0	0	0	0	0	0	0	0	2

Cluster26464	Proteobac	Alphap	Rhizc	Xanthol	Labrys	2	0	0	0	0	0	0	0	0	0	0	0	2
Cluster26593	Proteobac	Alphap	Caulc	Caulobа	Caulobacter	0	0	0	0	2	0	0	0	0	0	0	0	2
Cluster26599	Bacteroid	Sphing	Sphir	Chitinophagaceae		0	0	1	0	1	0	0	0	0	0	0	0	2
Cluster26643	Acidobact	Acidobacteria_Gp2			Gp2	0	0	0	0	0	0	0	0	1	0	1	0	2
Cluster26669	Bacteroid	Sphing	Sphir	Chitinophagaceae		0	0	0	2	0	0	0	0	0	0	0	0	2
Cluster26672;size=3						0	0	0	0	0	0	1	0	0	0	1	0	2
Cluster26699	Actinobac	Actinol	Actinomycetales			1	0	0	0	0	0	0	0	0	0	0	1	2
Cluster26719	Bacteroid	Sphing	Sphir	Chitinophagaceae		0	0	0	0	0	0	0	0	0	1	1	0	2
Cluster26735	Acidobact	Acidobacteria_Gp3			Bryobacter	0	0	0	1	0	0	1	0	0	0	0	0	2
Cluster26767	Acidobact	Acidobacteria_Gp3			Gp3	0	1	0	0	0	0	0	0	0	0	1	0	2
Cluster26770;size=7						1	0	0	0	0	0	0	1	0	0	0	0	2
Cluster26802	Proteobac	Alphaproteobacteria				0	1	0	0	0	0	0	0	0	0	0	1	2
Cluster26820	Proteobac	Alphap	Caulc	Caulobа	Phenylobacter	1	1	0	0	0	0	0	0	0	0	0	0	2
Cluster26870	Bacteroid	Sphing	Sphir	Chitinophagaceae		0	0	0	1	1	0	0	0	0	0	0	0	2
Cluster26896	Actinobac	Actinobacteria				0	0	1	0	0	0	0	0	0	0	1	0	2
Cluster26932	Acidobact	Acidobacteria_Gp10			Gp10	0	0	0	1	0	0	1	0	0	0	0	0	2
Cluster26953	Actinobac	Actinol	Acidimicrobiales			0	0	0	0	0	0	2	0	0	0	0	0	2
Cluster26954;size=5						0	0	2	0	0	0	0	0	0	0	0	0	2
Cluster26958	Bacteroid	Sphing	Sphir	Chitinoן	Sediminibacter	0	0	1	0	0	0	0	1	0	0	0	0	2
Cluster27075	Acidobact	Acidobacteria_Gp13			Gp13	0	0	0	0	0	0	0	1	1	0	0	0	2
Cluster27085;size=3						1	0	0	1	0	0	0	0	0	0	0	0	2
Cluster27111	Proteobac	Alphap	Rhizobiales			0	0	0	0	0	0	0	0	0	2	0	0	2
Cluster27128	Bacteroid	Sphing	Sphir	Chitinophagaceae		0	0	1	0	1	0	0	0	0	0	0	0	2
Cluster27206	Actinobac	Actinol	Acidimicrobiales			1	0	0	1	0	0	0	0	0	0	0	0	2
Cluster27264	Bacteroid	Sphing	Sphir	Chitinophagaceae		0	0	0	0	0	1	1	0	0	0	0	0	2
Cluster27384	Actinobac	Actinol	Actinomycetales			0	0	0	0	0	0	0	0	1	0	1	0	2
Cluster27546	Acidobact	Acidobacteria_Gp2			Gp2	0	0	0	0	0	0	2	0	0	0	0	0	2
Cluster27597	Acidobact	Acidobacteria_Gp7			Gp7	0	0	2	0	0	0	0	0	0	0	0	0	2
Cluster27784	Bacteroid	Sphing	Sphir	Chitinophagaceae		1	0	0	0	0	1	0	0	0	0	0	0	2

Cluster27820	Actinobac	Actinol	Actinomycetales			0	0	0	0	0	0	0	0	0	1	1	0	2
Cluster27952;size=7						0	0	0	0	0	2	0	0	0	0	0	0	2
Cluster28045	Actinobac	Actinol	Acidimicrobiales			0	0	1	0	0	1	0	0	0	0	0	0	2
Cluster28087	Acidobact	Acidobacteria_Gp3				0	0	2	0	0	0	0	0	0	0	0	0	2
Cluster28172	Bacteroid	Sphing	Sphir	Sphingo	Mucilaginibact	0	0	2	0	0	0	0	0	0	0	0	0	2
Cluster28216	Acidobacteria					0	0	0	0	1	0	0	0	1	0	0	0	2
Cluster28230	Bacteroid	Sphing	Sphir	Sphingo	Mucilaginibact	0	0	0	1	0	0	0	0	1	0	0	0	2
Cluster28251	Proteobac	Alphap	Caulo	Caulobacteraceae		0	0	0	1	1	0	0	0	0	0	0	0	2
Cluster28318	Bacteroid	Sphing	Sphir	Sphingo	Parapedobacte	0	0	0	0	1	0	0	1	0	0	0	0	2
Cluster28504	Actinobac	Actinol	Actinomycetales			0	0	0	0	0	0	0	0	0	0	2	0	2
Cluster28808;size=7						0	0	0	0	0	0	1	1	0	0	0	0	2
Cluster28837	Actinobac	Actinol	Actinomycetales			0	0	1	0	0	0	0	0	0	0	0	1	2
Cluster28894	Acidobact	Acidobacteria_Gp1				1	0	0	0	1	0	0	0	0	0	0	0	2
Cluster28931	Bacteroid	Sphing	Sphingobacteriales			0	1	0	1	0	0	0	0	0	0	0	0	2
Cluster29077	Actinobac	Actinol	Acidimicrobiales			0	1	0	0	0	0	0	1	0	0	0	0	2
Cluster29090	Bacteroid	Sphing	Sphir	Sphingo	Mucilaginibact	0	0	1	0	0	0	0	1	0	0	0	0	2
Cluster29134	Bacteroidetes					0	1	0	0	0	0	1	0	0	0	0	0	2
Cluster29255	Bacteroidetes					0	0	0	0	0	2	0	0	0	0	0	0	2
Cluster29294	Actinobac	Actinobacteria				0	0	0	1	0	0	0	0	1	0	0	0	2
Cluster29320	Bacteroid	Sphing	Sphir	Chitinophagaceae		2	0	0	0	0	0	0	0	0	0	0	0	2
Cluster29424	Chlorobi	Ignavib	Ignav	Ignaviba	Ignavibacteriu	0	0	0	0	0	0	1	0	1	0	0	0	2
Cluster29502	Proteobac	Gamm	Xanth	Sinobac	Steroidobacter	0	0	0	0	1	0	1	0	0	0	0	0	2
Cluster29588	Acidobact	Acidobacteria_Gp2			Gp2	0	0	0	0	1	0	0	0	1	0	0	0	2
Cluster29664;size=5						0	0	0	0	0	0	0	0	0	0	0	2	2
Cluster29667	Proteobac	Deltap	Myxococcales			1	0	0	0	0	0	0	0	0	0	1	0	2
Cluster29703	Actinobac	Actinol	Acidi	Acidimi	Aciditerrimona	0	0	0	0	0	0	0	0	0	2	0	0	2
Cluster30132	Bacteroidetes					0	0	0	0	0	2	0	0	0	0	0	0	2
Cluster3;size=7						0	0	1	0	0	0	0	0	0	0	0	0	1
Cluster12;siz	Actinobac	Actinol	Actinomycetales			0	0	0	0	0	0	0	0	0	0	0	1	1

Cluster18;siz	Acidobact	Acidobacteria_Gp1				0	0	0	0	0	0	0	0	1	0	0	0	1
Cluster41;siz	Proteobac	Alphap	Rhodospirillales			1	0	0	0	0	0	0	0	0	0	0	0	1
Cluster45;siz	Proteobac	Alphap	Rhizobiales			0	0	0	1	0	0	0	0	0	0	0	0	1
Cluster49;siz	Acidobact	Acidobacteria_Gp2			Gp2	0	0	0	0	0	0	1	0	0	0	0	0	1
Cluster63;size=14						0	1	0	0	0	0	0	0	0	0	0	0	1
Cluster70;siz	Acidobact	Acidobacteria_Gp2			Gp2	0	0	1	0	0	0	0	0	0	0	0	0	1
Cluster73;siz	Armatimc	Armati	Arma	Armatir	Armatimonas/	0	0	1	0	0	0	0	0	0	0	0	0	1
Cluster74;siz	Acidobact	Acidobacteria_Gp1				0	0	0	0	0	1	0	0	0	0	0	0	1
Cluster80;siz	Proteobac	Deltap	Myxococcales			0	1	0	0	0	0	0	0	0	0	0	0	1
Cluster89;siz	Acidobact	Acidobacteria_Gp3			Gp3	0	0	0	0	0	1	0	0	0	0	0	0	1
Cluster101;size=16						0	0	0	0	0	0	0	0	0	0	0	1	1
Cluster104;si	Proteobac	Betapr	Burkl	Burkhol	Burkholderia	1	0	0	0	0	0	0	0	0	0	0	0	1
Cluster106;si	Proteobac	Alphap	Rhizc	Bradyrhizobiaceae		0	0	0	0	0	0	0	1	0	0	0	0	1
Cluster111;si	Acidobact	Acidobacteria_Gp1			Gp1	0	0	0	0	0	0	1	0	0	0	0	0	1
Cluster113;si	Verrucom	Spartobacteria			Spartobacteria	0	0	0	0	0	0	0	0	0	0	1	0	1
Cluster114;si	Acidobact	Acidobacteria_Gp1				0	0	0	0	0	1	0	0	0	0	0	0	1
Cluster125;si	Acidobact	Acidobacteria_Gp3			Gp3	0	1	0	0	0	0	0	0	0	0	0	0	1
Cluster149;si	Chloroflex	Ktedor	Ktedonobacterales			0	0	0	0	0	0	0	1	0	0	0	0	1
Cluster150;si	Acidobact	Acidobacteria_Gp1			Granulicella	0	0	1	0	0	0	0	0	0	0	0	0	1
Cluster151;si	TM7				TM7_genera_i	0	1	0	0	0	0	0	0	0	0	0	0	1
Cluster167;si	Acidobact	Acidobacteria_Gp2			Gp2	0	0	0	0	1	0	0	0	0	0	0	0	1
Cluster169;si	Proteobac	Deltap	Myxococcales			0	0	0	0	0	0	0	0	1	0	0	0	1
Cluster182;si	Proteobac	Alphap	Rhodospirillales			0	0	0	0	0	0	0	0	1	0	0	0	1
Cluster185;si	Acidobact	Acidobacteria_Gp2			Gp2	0	0	1	0	0	0	0	0	0	0	0	0	1
Cluster186;si	Proteobac	Alphap	Rhizc	Beijerinckiaceae		0	0	0	0	0	1	0	0	0	0	0	0	1
Cluster188;si	Acidobact	Acidobacteria_Gp2			Gp2	0	0	0	0	0	0	1	0	0	0	0	0	1
Cluster189;si	Acidobact	Acidobacteria_Gp1				0	0	0	0	0	0	0	0	0	1	0	0	1
Cluster194;si	Acidobact	Acidobacteria_Gp6			Gp6	0	0	0	1	0	0	0	0	0	0	0	0	1
Cluster204;si	Chloroflex	Ktedor	Ktedonobacterales			0	0	0	0	0	0	0	0	1	0	0	0	1

Cluster206;si	Proteobac	Gammaproteobacteria				0	0	0	0	0	1	0	0	0	0	0	0	1
Cluster209;si	Acidobact	Acidobacteria_Gp1				0	0	0	1	0	0	0	0	0	0	0	0	1
Cluster210;si	Acidobact	Acidobacteria_Gp2			Gp2	1	0	0	0	0	0	0	0	0	0	0	0	1
Cluster212;si	Acidobact	Acidobacteria_Gp1				1	0	0	0	0	0	0	0	0	0	0	0	1
Cluster217;si	Acidobact	Acidobacteria_Gp6			Gp6	1	0	0	0	0	0	0	0	0	0	0	0	1
Cluster237;si	Proteobac	Gamm	Xanth	Sinobac	Steroidobacter	1	0	0	0	0	0	0	0	0	0	0	0	1
Cluster238;si	Proteobac	Alphap	Rhod	Rhodos	Dongia	1	0	0	0	0	0	0	0	0	0	0	0	1
Cluster240;si	Proteobac	Deltap	Myxo	Polyangiaceae		0	1	0	0	0	0	0	0	0	0	0	0	1
Cluster243;si	Acidobact	Acidobacteria_Gp1			Gp1	0	0	0	0	0	0	1	0	0	0	0	0	1
Cluster244;si	Acidobact	Acidobacteria_Gp2			Gp2	0	0	0	1	0	0	0	0	0	0	0	0	1
Cluster252;si	Acidobact	Acidobacteria_Gp1			Gp1	0	0	0	0	0	0	0	0	0	0	1	0	1
Cluster254;si	Proteobac	Alphap	Caulo	Cauloba	Caulobacter	0	0	1	0	0	0	0	0	0	0	0	0	1
Cluster265;si	Acidobact	Acidobacteria_Gp2			Gp2	1	0	0	0	0	0	0	0	0	0	0	0	1
Cluster271;si	Proteobac	Alphap	Rhizobiales			0	0	0	0	0	0	0	1	0	0	0	0	1
Cluster277;si	Proteobac	Alphap	Rhizobiales			0	0	0	0	1	0	0	0	0	0	0	0	1
Cluster287;si	Acidobact	Acidobacteria_Gp1				0	0	0	0	0	0	1	0	0	0	0	0	1
Cluster289;si	Proteobac	Alphap	Rhod	Acetobacteraceae		0	0	1	0	0	0	0	0	0	0	0	0	1
Cluster290;si	Proteobac	Betapr	Burkh	Oxaloba	Collimonas	0	0	0	0	0	0	0	1	0	0	0	0	1
Cluster293;size=3						0	0	0	0	0	0	0	0	1	0	0	0	1
Cluster296;si	Actinobac	Actinob	Actin	Pseudonocardiaceae		0	0	0	0	0	0	0	0	0	0	1	0	1
Cluster300;si	Acidobact	Acidobacteria_Gp1				0	0	0	0	0	0	0	0	1	0	0	0	1
Cluster301;si	Acidobact	Acidobacteria_Gp1			Gp1	0	0	0	0	0	0	0	0	0	0	1	0	1
Cluster303;size=5						0	0	0	0	0	0	1	0	0	0	0	0	1
Cluster305;si	Acidobact	Acidobacteria_Gp1				0	0	1	0	0	0	0	0	0	0	0	0	1
Cluster308;si	TM7				TM7_genera_i	0	0	0	0	0	0	0	0	0	1	0	0	1
Cluster318;si	Proteobac	Gamm	Xanth	Sinobac	Steroidobacter	0	0	1	0	0	0	0	0	0	0	0	0	1
Cluster319;si	Chloroflex	Ktedon	Ktedonobacterales			0	0	0	0	0	0	0	0	0	1	0	0	1
Cluster322;si	Acidobact	Acidobacteria_Gp3			Gp3	0	0	0	0	0	0	0	0	0	1	0	0	1
Cluster330;si	Acidobact	Acidobacteria_Gp1			Gp1	0	1	0	0	0	0	0	0	0	0	0	0	1

Cluster336;si	Bacteroid	Sphing	Sphir	Chitinophagaceae		0	0	0	0	1	0	0	0	0	0	0	0	1
Cluster344;size=2						0	0	0	0	1	0	0	0	0	0	0	0	1
Cluster349;size=5						1	0	0	0	0	0	0	0	0	0	0	0	1
Cluster350;si	Proteobac	Gammaproteobacteria				0	0	0	0	1	0	0	0	0	0	0	0	1
Cluster352;si	Proteobac	Deltap	Myxococcales			0	0	0	0	0	0	0	0	0	0	1	0	1
Cluster354;si	Proteobac	Betapr	Burkholderiales			0	0	0	0	0	0	0	1	0	0	0	0	1
Cluster359;si	Proteobac	Alphap	Rhizobiales			0	0	0	0	0	0	0	0	0	0	0	1	1
Cluster361;size=13						0	1	0	0	0	0	0	0	0	0	0	0	1
Cluster365;si	Acidobact	Acidobacteria_Gp1			Gp1	0	0	0	0	0	0	0	0	1	0	0	0	1
Cluster374;si	Proteobac	Deltap	Bdell	Bdellovi	Bdellovibrio	0	0	0	0	0	0	0	0	0	0	1	0	1
Cluster375;si	Acidobact	Acidobacteria_Gp1				0	0	0	0	0	0	0	0	0	0	1	0	1
Cluster376;size=2						0	0	0	0	0	0	0	0	0	1	0	0	1
Cluster381;si	Verrucom	Opituta	Opitu	Opituta	Opitutus	0	0	0	0	0	0	0	0	0	1	0	0	1
Cluster385;si	Proteobac	Alphap	Rhodospirillales			0	0	0	0	1	0	0	0	0	0	0	0	1
Cluster386;si	Acidobact	Acidobacteria_Gp2			Gp2	0	0	0	1	0	0	0	0	0	0	0	0	1
Cluster399;si	Acidobact	Acidobacteria_Gp1				0	0	0	0	0	0	0	0	0	0	0	1	1
Cluster404;si	TM7				TM7_genera_i	0	0	0	0	0	0	0	0	0	1	0	0	1
Cluster408;si	Acidobact	Acidobacteria_Gp1				0	0	0	0	0	0	0	0	0	0	1	0	1
Cluster421;si	Proteobac	Betapr	Burkholderiales			0	0	1	0	0	0	0	0	0	0	0	0	1
Cluster423;si	Acidobact	Acidobacteria_Gp3			Gp3	0	0	0	0	1	0	0	0	0	0	0	0	1
Cluster424;si	Verrucom	Subdivision3			Subdivision3_g	0	0	0	0	1	0	0	0	0	0	0	0	1
Cluster425;si	Verrucom	Subdivision3			Subdivision3_g	0	0	0	0	0	0	1	0	0	0	0	0	1
Cluster426;si	Acidobact	Acidobacteria_Gp2			Gp2	0	0	0	0	1	0	0	0	0	0	0	0	1
Cluster431;si	TM7				TM7_genera_i	0	0	0	0	0	0	0	0	1	0	0	0	1
Cluster434;si	Actinobac	Actinol	Actin	Mycoba	Mycobacteriur	0	0	1	0	0	0	0	0	0	0	0	0	1
Cluster443;si	Proteobac	Alphaproteobacteria				0	1	0	0	0	0	0	0	0	0	0	0	1
Cluster447;si	Proteobacteria					0	0	0	0	1	0	0	0	0	0	0	0	1
Cluster465;size=1						0	0	0	0	0	0	0	0	0	0	1	0	1
Cluster467;si	Armatimc	Chthor	Chth	Chthon	Chthonomona	0	1	0	0	0	0	0	0	0	0	0	0	1

Cluster469;si	Acidobact	Acidobacteria_Gp1			Acidobacteriur	0	0	0	1	0	0	0	0	0	0	0	0	1
Cluster481;si	Acidobact	Acidobacteria_Gp1			Acidobacteriur	0	0	0	0	0	0	0	1	0	0	0	0	1
Cluster493;si	Acidobact	Acidobacteria_Gp3			Gp3	0	0	0	0	1	0	0	0	0	0	0	0	1
Cluster495;si	Acidobact	Acidobacteria_Gp3			Gp3	0	0	0	0	0	0	0	0	0	1	0	0	1
Cluster498;si	Proteobac	Gammaproteobacteria				0	0	0	0	0	1	0	0	0	0	0	0	1
Cluster502;si	Acidobact	Acidobacteria_Gp1				0	0	1	0	0	0	0	0	0	0	0	0	1
Cluster510;si	Acidobact	Acidobacteria_Gp10			Gp10	0	0	0	0	0	0	0	0	1	0	0	0	1
Cluster519;si	Planctom	Plancto	Planc	Planctomycetaceae		1	0	0	0	0	0	0	0	0	0	0	0	1
Cluster522;si	Acidobact	Acidobacteria_Gp2			Gp2	0	0	0	0	0	0	1	0	0	0	0	0	1
Cluster523;si	Acidobact	Acidobacteria_Gp1				0	0	0	0	0	0	0	0	0	0	1	0	1
Cluster532;si	Acidobact	Acidobacteria_Gp6			Gp6	0	0	0	0	0	1	0	0	0	0	0	0	1
Cluster533;si	Firmicutes					0	0	0	0	0	0	0	0	1	0	0	0	1
Cluster537;si	Chloroflexi					0	0	0	1	0	0	0	0	0	0	0	0	1
Cluster540;si	Acidobact	Acidobacteria_Gp1				0	0	0	0	0	1	0	0	0	0	0	0	1
Cluster553;si	Proteobac	Betapr	Burkholderiales			1	0	0	0	0	0	0	0	0	0	0	0	1
Cluster554;si	Firmicutes	Bacilli	Bacillales			1	0	0	0	0	0	0	0	0	0	0	0	1
Cluster556;si	Acidobact	Acidobacteria_Gp2			Gp2	1	0	0	0	0	0	0	0	0	0	0	0	1
Cluster560;si	Acidobact	Acidobacteria_Gp2			Gp2	1	0	0	0	0	0	0	0	0	0	0	0	1
Cluster565;si	Acidobact	Acidobacteria_Gp10			Gp10	1	0	0	0	0	0	0	0	0	0	0	0	1
Cluster566;si	Acidobact	Acidobacteria_Gp2			Gp2	0	0	0	0	0	0	0	0	0	0	1	0	1
Cluster568;si	Proteobac	Alphap	Rhizo	Methylo	Methylosinus	0	0	0	0	0	0	0	0	0	1	0	0	1
Cluster573;si	Acidobact	Acidobacteria_Gp1			Gp1	0	0	0	0	0	0	0	0	0	0	0	1	1
Cluster574;si	Proteobac	Alphap	Rhizo	Xanthob	Pseudolabrys	0	0	0	0	1	0	0	0	0	0	0	0	1
Cluster575;si	Acidobact	Acidobacteria_Gp3			Gp3	0	0	1	0	0	0	0	0	0	0	0	0	1
Cluster576;si	Proteobac	Alphap	Rhodospirillales			1	0	0	0	0	0	0	0	0	0	0	0	1
Cluster577;si	Acidobact	Acidobacteria_Gp2			Gp2	1	0	0	0	0	0	0	0	0	0	0	0	1
Cluster580;si	Proteobac	Alphap	Rhodospirillales			1	0	0	0	0	0	0	0	0	0	0	0	1
Cluster583;si	Verrucom	Subdivision3			Subdivision3_g	1	0	0	0	0	0	0	0	0	0	0	0	1
Cluster585;size=14						0	0	0	1	0	0	0	0	0	0	0	0	1

Cluster586;si	Verrucom	Opitut	Opitu	Opituta	Opitutus	1	0	0	0	0	0	0	0	0	0	0	0	1
Cluster588;si	Acidobact	Acidobacteria_Gp2			Gp2	0	0	0	0	0	0	0	0	0	1	0	0	1
Cluster590;si	Acidobact	Acidobacteria_Gp2			Gp2	0	0	0	0	0	0	0	0	0	0	0	1	1
Cluster592;si	Acidobact	Acidobacteria_Gp3				1	0	0	0	0	0	0	0	0	0	0	0	1
Cluster597;si	Proteobac	Gammaproteobacteria				0	0	0	0	1	0	0	0	0	0	0	0	1
Cluster600;si	Bacteroid	Sphing	Sphir	Chitino	Ferruginibacte	0	1	0	0	0	0	0	0	0	0	0	0	1
Cluster605;si	Firmicute	Clostridia				0	0	0	0	0	0	0	1	0	0	0	0	1
Cluster610;si	Proteobac	Gammaproteobacteria				1	0	0	0	0	0	0	0	0	0	0	0	1
Cluster612;si	Proteobac	Alphap	Rhizobiales			0	0	0	0	1	0	0	0	0	0	0	0	1
Cluster614;si	Proteobac	Alphap	Rhodospirillales			0	0	0	0	0	0	0	0	0	0	0	1	1
Cluster635;si	Verrucom	Opitut	Opitu	Opituta	Opitutus	1	0	0	0	0	0	0	0	0	0	0	0	1
Cluster639;si	Acidobact	Acidobacteria_Gp1			Gp1	0	0	0	0	0	1	0	0	0	0	0	0	1
Cluster642;si	Acidobact	Acidobacteria_Gp2			Gp2	0	0	0	0	0	0	0	0	0	0	0	1	1
Cluster644;size=1						0	0	0	0	0	0	0	0	0	0	1	0	1
Cluster645;si	Proteobac	Alphap	Rhizobiales			0	0	0	0	0	0	0	0	0	0	1	0	1
Cluster648;si	Proteobac	Betaproteobacteria				0	0	0	0	0	0	0	0	0	0	0	1	1
Cluster660;si	Acidobact	Acidobacteria_Gp2			Gp2	1	0	0	0	0	0	0	0	0	0	0	0	1
Cluster666;si	Proteobac	Alphaproteobacteria				0	0	0	0	0	0	0	1	0	0	0	0	1
Cluster667;si	Acidobact	Acidobacteria_Gp1			Gp1	1	0	0	0	0	0	0	0	0	0	0	0	1
Cluster668;si	Acidobact	Acidobacteria_Gp1				0	0	0	0	0	0	0	0	0	1	0	0	1
Cluster675;si	Proteobac	Gammaproteobacteria				0	0	0	0	0	0	1	0	0	0	0	0	1
Cluster677;si	Bacteroid	Sphing	Sphir	Sphingobacteriaceae		0	0	0	0	0	0	0	0	0	1	0	0	1
Cluster679;si	Verrucom	Opitut	Opitu	Opituta	Opitutus	0	0	0	0	0	0	0	1	0	0	0	0	1
Cluster680;si	Proteobac	Alphap	Rhizobiales			1	0	0	0	0	0	0	0	0	0	0	0	1
Cluster684;si	Acidobact	Acidobacteria_Gp1			Acidobacteriu	1	0	0	0	0	0	0	0	0	0	0	0	1
Cluster688;si	Gemmati	Gemm	Gem	Gemma	Gemmatimona	0	0	0	0	0	0	0	0	0	0	1	0	1
Cluster691;si	Acidobact	Acidobacteria_Gp2			Gp2	0	0	0	0	1	0	0	0	0	0	0	0	1
Cluster693;si	Proteobac	Gamm	Xant	Sinobac	Steroidobacte	1	0	0	0	0	0	0	0	0	0	0	0	1
Cluster697;si	Proteobac	Alphaproteobacteria				0	0	0	0	0	1	0	0	0	0	0	0	1

Cluster701;size=1						1	0	0	0	0	0	0	0	0	0	0	0	1
Cluster703;si	Proteobac	Deltap	Myxococcales			0	0	0	0	0	0	0	0	0	0	0	1	1
Cluster707;si	Proteobac	Alphap	Rhod	Acetobacteraceae		0	0	0	1	0	0	0	0	0	0	0	0	1
Cluster708;size=6						0	0	0	1	0	0	0	0	0	0	0	0	1
Cluster709;si	Actinobac	Actinol	Actin	Nocardiaceae		0	0	0	0	0	0	0	0	0	0	0	1	1
Cluster734;si	Proteobac	Gamm	Xantl	Sinobac	Steroidobacter	0	0	1	0	0	0	0	0	0	0	0	0	1
Cluster739;si	Acidobact	Acidobacteria_Gp1			Gp1	0	0	0	1	0	0	0	0	0	0	0	0	1
Cluster745;si	Acidobacteria					0	0	1	0	0	0	0	0	0	0	0	0	1
Cluster748;si	Acidobact	Acidobacteria_Gp1			Gp1	0	0	0	0	0	0	1	0	0	0	0	0	1
Cluster756;si	Acidobact	Acidobacteria_Gp2			Gp2	0	0	0	0	0	0	1	0	0	0	0	0	1
Cluster768;si	Bacteroid	Sphing	Sphir	Chitinophagaceae		0	0	0	0	0	0	0	1	0	0	0	0	1
Cluster770;si	Acidobact	Acidobacteria_Gp2			Gp2	0	0	0	1	0	0	0	0	0	0	0	0	1
Cluster778;si	Acidobact	Acidobacteria_Gp2			Gp2	0	0	0	0	0	0	0	0	1	0	0	0	1
Cluster783;si	Acidobact	Acidobacteria_Gp2			Gp2	1	0	0	0	0	0	0	0	0	0	0	0	1
Cluster784;si	Acidobact	Acidobacteria_Gp1			Gp1	0	0	0	0	0	0	0	0	0	1	0	0	1
Cluster787;si	Acidobact	Acidobacteria_Gp1			Granulicella	0	0	1	0	0	0	0	0	0	0	0	0	1
Cluster789;size=8						0	0	0	0	0	0	0	0	0	0	1	0	1
Cluster793;si	Acidobact	Acidobacteria_Gp1			Gp1	0	0	0	0	1	0	0	0	0	0	0	0	1
Cluster800;size=2						0	0	1	0	0	0	0	0	0	0	0	0	1
Cluster803;si	Acidobact	Acidobacteria_Gp1			Gp1	0	0	0	0	0	1	0	0	0	0	0	0	1
Cluster808;si	Acidobact	Acidobacteria_Gp3			Gp3	0	0	0	0	0	1	0	0	0	0	0	0	1
Cluster811;size=2						0	0	1	0	0	0	0	0	0	0	0	0	1
Cluster814;si	Proteobac	Alphap	Rhod	Acetobacteraceae		0	0	0	0	0	0	0	0	0	1	0	0	1
Cluster820;si	Proteobac	Alphap	Caulc	Caulobacteraceae		0	0	0	1	0	0	0	0	0	0	0	0	1
Cluster821;si	Proteobacteria					1	0	0	0	0	0	0	0	0	0	0	0	1
Cluster822;si	Proteobac	Alphap	Rhodospirillales			0	0	0	0	0	0	0	1	0	0	0	0	1
Cluster823;si	Acidobact	Acidobacteria_Gp7			Gp7	0	0	0	0	1	0	0	0	0	0	0	0	1
Cluster830;si	Proteobac	Alphap	Rhod	Acetobacteraceae		0	0	1	0	0	0	0	0	0	0	0	0	1
Cluster831;si	Proteobac	Deltap	Myxc	Polyangiaceae		0	0	0	0	0	1	0	0	0	0	0	0	1

Cluster832;size=1						0	0	0	0	0	0	0	1	0	0	0	0	1
Cluster838;si	Acidobact	Acidobacteria_Gp2			Gp2	0	0	0	0	0	0	0	0	0	1	0	0	1
Cluster839;size=1						0	0	0	1	0	0	0	0	0	0	0	0	1
Cluster843;si	Chlamydia	Chlamy	Chlamydiales			0	0	0	1	0	0	0	0	0	0	0	0	1
Cluster855;si	Actinobac	Actinobacteria				0	0	1	0	0	0	0	0	0	0	0	0	1
Cluster858;si	Proteobac	Alphap	Rhizobiales			0	0	0	0	0	0	0	0	0	1	0	0	1
Cluster859;si	Acidobact	Acidobacteria_Gp1				0	0	0	0	0	1	0	0	0	0	0	0	1
Cluster863;si	Actinobac	Actinol	Acidi	Acidimi	Aciditerrimona	0	0	0	0	0	0	1	0	0	0	0	0	1
Cluster865;si	Proteobac	Gamm	Enter	Enterobacteriaceae		0	0	0	0	0	0	0	0	0	1	0	0	1
Cluster867;si	Proteobac	Gammaproteobacteria				0	0	0	0	0	1	0	0	0	0	0	0	1
Cluster885;si	Proteobac	Deltap	Syntrophobacterales			0	1	0	0	0	0	0	0	0	0	0	0	1
Cluster890;si	Proteobac	Betapr	Burkholderiales			0	0	0	1	0	0	0	0	0	0	0	0	1
Cluster891;si	Acidobact	Acidobacteria_Gp1				0	0	0	0	0	0	0	0	1	0	0	0	1
Cluster892;si	Proteobac	Deltap	Myxococcales			0	0	0	0	1	0	0	0	0	0	0	0	1
Cluster899;si	Acidobact	Acidobacteria_Gp3			Gp3	0	0	0	0	1	0	0	0	0	0	0	0	1
Cluster903;si	Acidobact	Acidobacteria_Gp1			Gp1	0	0	0	0	0	0	1	0	0	0	0	0	1
Cluster909;si	Proteobac	Deltap	Myxococcales			0	0	0	0	0	0	1	0	0	0	0	0	1
Cluster910;si	Proteobac	Alphap	Alphaproteob		Rhizomicrobiu	0	0	0	0	0	0	0	1	0	0	0	0	1
Cluster911;si	Proteobac	Deltap	Myxc	Cystobacteraceae		1	0	0	0	0	0	0	0	0	0	0	0	1
Cluster919;si	Acidobact	Acidobacteria_Gp2			Gp2	1	0	0	0	0	0	0	0	0	0	0	0	1
Cluster920;si	Acidobact	Acidobacteria_Gp1			Gp1	0	0	0	0	1	0	0	0	0	0	0	0	1
Cluster927;si	Verrucom	Subdivision3			Subdivision3_g	0	0	0	0	0	0	0	0	1	0	0	0	1
Cluster933;si	Bacteroid	Sphing	Sphir	Chitinophagaceae		0	0	0	0	0	0	0	1	0	0	0	0	1
Cluster935;size=1						1	0	0	0	0	0	0	0	0	0	0	0	1
Cluster942;si	Acidobact	Acidobacteria_Gp1			Acidobacteriur	0	0	0	1	0	0	0	0	0	0	0	0	1
Cluster944;si	Proteobac	Betapr	Burkl	Oxalobacteraceae		0	0	0	0	1	0	0	0	0	0	0	0	1
Cluster946;si	Bacteroid	Sphing	Sphir	Chitinophagaceae		0	0	0	0	0	0	0	0	0	1	0	0	1
Cluster947;si	Verrucom	Subdivision3			Subdivision3_g	0	0	1	0	0	0	0	0	0	0	0	0	1
Cluster956;si	Acidobact	Acidobacteria_Gp1			Gp1	0	0	1	0	0	0	0	0	0	0	0	0	1

Cluster957;si	Proteobac	Gamm	Xanth	Sinobac	Steroidobacter	0	0	0	0	0	0	0	0	0	1	0	0	1
Cluster968;si	Proteobac	Betapr	Burkh	Burkhol	Burkholderia	0	0	0	0	0	0	0	0	0	0	1	0	1
Cluster972;si	Proteobac	Betapr	Burkh	Burkhol	Burkholderia	0	0	0	0	0	0	0	1	0	0	0	0	1
Cluster973;si	Proteobac	Deltaproteobacteria				0	0	1	0	0	0	0	0	0	0	0	0	1
Cluster977;si	Proteobac	Gammaproteobacteria				0	0	0	0	0	0	0	0	0	1	0	0	1
Cluster986;si	Bacteroid	Sphing	Sphir	Chitinophagaceae		0	0	0	0	0	1	0	0	0	0	0	0	1
Cluster987;si	Acidobact	Acidobacteria_Gp1			Granulicella	0	0	1	0	0	0	0	0	0	0	0	0	1
Cluster993;si	Proteobac	Alphap	Rhizobiales			0	0	0	0	0	0	1	0	0	0	0	0	1
Cluster1008;	Proteobac	Alphaproteobacteria				0	0	0	0	0	0	0	0	0	1	0	0	1
Cluster1014;	Proteobac	Deltaproteobacteria				0	1	0	0	0	0	0	0	0	0	0	0	1
Cluster1023;	Proteobac	Alphap	Rhodospirillales			0	0	0	1	0	0	0	0	0	0	0	0	1
Cluster1024;	Proteobac	Alphap	Rhod	Acetoba	Acidocella	0	0	0	0	0	0	0	0	0	0	0	1	1
Cluster1026;size=4						0	0	1	0	0	0	0	0	0	0	0	0	1
Cluster1043;	Acidobact	Acidobacteria_Gp1				0	0	0	0	0	0	1	0	0	0	0	0	1
Cluster1044;	Proteobac	Alphap	Rhizo	Bradyrh	Agromonas	0	0	0	0	0	0	0	0	0	1	0	0	1
Cluster1049;	Proteobac	Gammaproteobacteria				0	0	0	0	0	0	0	1	0	0	0	0	1
Cluster1054;	Proteobac	Alphap	Rhizobiales			1	0	0	0	0	0	0	0	0	0	0	0	1
Cluster1055;	Bacteroid	Sphing	Sphir	Sphingo	Mucilaginibact	1	0	0	0	0	0	0	0	0	0	0	0	1
Cluster1056;	Chlamydia	Chlamy	Chlamydiales			0	0	1	0	0	0	0	0	0	0	0	0	1
Cluster1060;	Acidobact	Acidobacteria_Gp2			Gp2	0	0	0	0	0	0	1	0	0	0	0	0	1
Cluster1061;	Proteobac	Alphap	Rhodospirillales			1	0	0	0	0	0	0	0	0	0	0	0	1
Cluster1064;	Verrucom	Opitutae				0	0	0	0	0	0	0	0	0	1	0	0	1
Cluster1067;	Proteobac	Gammaproteobacteria				1	0	0	0	0	0	0	0	0	0	0	0	1
Cluster1088;	Bacteroid	Flavob	Flavo	Flavobacteriaceae		0	0	0	0	0	0	0	0	0	1	0	0	1
Cluster1089;size=2						0	0	0	1	0	0	0	0	0	0	0	0	1
Cluster1099;	Acidobact	Acidobacteria_Gp1			Gp1	0	0	0	0	0	0	0	1	0	0	0	0	1
Cluster1101;	Acidobact	Acidobacteria_Gp3				0	0	0	0	0	0	0	0	0	0	0	1	1
Cluster1106;	Proteobac	Deltap	Myxococcales			1	0	0	0	0	0	0	0	0	0	0	0	1
Cluster1111;	Acidobact	Acidobacteria_Gp2			Gp2	0	0	0	0	0	0	0	1	0	0	0	0	1

Cluster1112;	Proteobac	Gammaproteobacteria				0	0	0	0	0	0	0	0	1	0	0	0	1
Cluster1114;	Proteobac	Deltap	Myxococcales			0	0	0	0	0	0	0	0	0	1	0	0	1
Cluster1118;	Armatimonadetes				Armatimonade	0	0	0	0	1	0	0	0	0	0	0	0	1
Cluster1123;	Acidobact	Acidobacteria_Gp1				0	0	0	0	0	0	0	0	0	0	1	0	1
Cluster1141;	Armatimc	Armati	Arma	Armatir	Armatimonas/	1	0	0	0	0	0	0	0	0	0	0	0	1
Cluster1153;	Proteobac	Alphaproteobacteria				0	0	1	0	0	0	0	0	0	0	0	0	1
Cluster1154;	Proteobacteria					1	0	0	0	0	0	0	0	0	0	0	0	1
Cluster1162;	Acidobact	Acidobacteria_Gp2			Gp2	0	0	0	0	0	0	0	0	0	0	1	0	1
Cluster1163;	Proteobac	Gammaproteobacteria				1	0	0	0	0	0	0	0	0	0	0	0	1
Cluster1166;size=8						0	0	0	0	0	0	0	0	0	0	0	1	1
Cluster1167;	Proteobac	Alphap	Sphir	Sphingc	Novosphingob	0	0	0	0	0	1	0	0	0	0	0	0	1
Cluster1168;	Proteobac	Deltaproteobacteria				0	1	0	0	0	0	0	0	0	0	0	0	1
Cluster1169;size=3						1	0	0	0	0	0	0	0	0	0	0	0	1
Cluster1176;	Proteobac	Betaproteobacteria				0	0	0	1	0	0	0	0	0	0	0	0	1
Cluster1177;	Acidobact	Acidobacteria_Gp3			Gp3	0	0	0	0	0	1	0	0	0	0	0	0	1
Cluster1184;	Acidobact	Acidobacteria_Gp2			Gp2	1	0	0	0	0	0	0	0	0	0	0	0	1
Cluster1185;	Acidobact	Acidobacteria_Gp3				0	0	0	0	0	0	0	1	0	0	0	0	1
Cluster1186;	Proteobac	Gammaproteobacteria				0	0	1	0	0	0	0	0	0	0	0	0	1
Cluster1187;	Acidobact	Acidobacteria_Gp1				0	0	0	0	0	0	0	1	0	0	0	0	1
Cluster1192;	Acidobact	Acidobacteria_Gp2			Gp2	0	0	0	0	1	0	0	0	0	0	0	0	1
Cluster1196;	Acidobact	Acidobacteria_Gp1			Gp1	0	0	0	1	0	0	0	0	0	0	0	0	1
Cluster1200;	Chloroflex	Ktedor	Ktedonobacterales			0	0	0	0	0	0	0	0	0	0	1	0	1
Cluster1202;	Bacteroid	Sphing	Sphir	Chitinophagaceae		0	0	0	0	0	0	1	0	0	0	0	0	1
Cluster1203;	Acidobact	Acidobacteria_Gp6			Gp6	0	0	0	0	1	0	0	0	0	0	0	0	1
Cluster1204;	Acidobact	Acidobacteria_Gp1			Gp1	0	0	0	0	0	0	0	0	0	1	0	0	1
Cluster1205;size=1						0	0	0	1	0	0	0	0	0	0	0	0	1
Cluster1211;	Proteobac	Alphap	Rhizc	Beijerinckiaceae		0	0	1	0	0	0	0	0	0	0	0	0	1
Cluster1213;	Acidobact	Acidobacteria_Gp3				1	0	0	0	0	0	0	0	0	0	0	0	1
Cluster1215;	Proteobac	Gammaproteobacteria				0	0	0	0	0	0	0	1	0	0	0	0	1

Cluster1220;	Acidobact	Acidobacteria_Gp2			Gp2	0	0	0	0	0	0	1	0	0	0	0	0	1
Cluster1221;	Verrucom	Subdivision3			Subdivision3_g	1	0	0	0	0	0	0	0	0	0	0	0	1
Cluster1228;	Proteobac	Deltaproteobacteria				0	0	0	0	0	0	0	0	1	0	0	0	1
Cluster1231;	Proteobac	Gammaproteobacteria				0	0	0	1	0	0	0	0	0	0	0	0	1
Cluster1233;	Acidobact	Acidobacteria_Gp3			Gp3	0	0	0	0	1	0	0	0	0	0	0	0	1
Cluster1243;	Chlorobi	Ignavib	Ignav	Ignaviba	Ignavibacteriu	0	0	0	0	0	0	0	0	0	1	0	0	1
Cluster1250;	Verrucom	Opitut	Opitu	Opituta	Opitutus	0	0	0	0	0	1	0	0	0	0	0	0	1
Cluster1251;	Proteobac	Gamm	Legio	Legione	Legionella	1	0	0	0	0	0	0	0	0	0	0	0	1
Cluster1255;	Actinobac	Actinol	Actinomycetales			0	0	0	0	0	0	0	0	0	1	0	0	1
Cluster1260;	Actinobac	Actinol	Actin	Nocardiaceae		0	0	0	0	0	0	0	0	0	0	1	0	1
Cluster1262;size=7						0	0	0	0	0	1	0	0	0	0	0	0	1
Cluster1276;size=2						0	0	0	0	1	0	0	0	0	0	0	0	1
Cluster1284;	Proteobac	Alphap	Rhod	Acetobacteraceae		1	0	0	0	0	0	0	0	0	0	0	0	1
Cluster1291;	Proteobac	Alphap	Rhizo	Bradyrhizobiaceae		0	0	0	0	0	0	0	0	0	1	0	0	1
Cluster1306;	Acidobact	Acidobacteria_Gp1				0	0	0	0	0	0	0	0	0	1	0	0	1
Cluster1309;	Acidobact	Acidobacteria_Gp2			Gp2	0	0	0	1	0	0	0	0	0	0	0	0	1
Cluster1312;	Acidobact	Acidobacteria_Gp2			Gp2	0	0	0	0	0	0	0	0	1	0	0	0	1
Cluster1318;	Proteobac	Alphap	Rhodospirillales			0	0	0	0	0	0	1	0	0	0	0	0	1
Cluster1322;	Acidobact	Acidobacteria_Gp2			Gp2	1	0	0	0	0	0	0	0	0	0	0	0	1
Cluster1323;	Gemmatir	Gemm	Gemı	Gemma	Gemmatimona	0	0	0	0	0	0	1	0	0	0	0	0	1
Cluster1339;	Acidobact	Acidobacteria_Gp3			Bryobacter	0	0	0	0	0	0	0	0	0	0	0	1	1
Cluster1341;	Proteobac	Alphap	Caulc	Caulobacteraceae		0	0	0	0	0	0	0	1	0	0	0	0	1
Cluster1346;	Proteobac	Betapr	Burkholderiales			1	0	0	0	0	0	0	0	0	0	0	0	1
Cluster1347;	Proteobac	Alphaproteobacteria				0	0	0	0	0	0	0	0	0	1	0	0	1
Cluster1348;	Acidobact	Acidobacteria_Gp3			Gp3	1	0	0	0	0	0	0	0	0	0	0	0	1
Cluster1349;	Proteobac	Deltaproteobacteria				0	0	1	0	0	0	0	0	0	0	0	0	1
Cluster1360;	Proteobac	Alphaproteobacteria				0	0	0	0	0	0	0	1	0	0	0	0	1
Cluster1362;	Actinobac	Actinol	Actinomycetales			0	0	1	0	0	0	0	0	0	0	0	0	1
Cluster1367;	Proteobac	Alphap	Rhizobiales			0	0	0	0	0	0	1	0	0	0	0	0	1

Cluster1368;	Chlamydia	Chlamy	Chlamydiales			1	0	0	0	0	0	0	0	0	0	0	0	1
Cluster1370;	Acidobact	Acidobacteria_Gp2			Gp2	0	0	0	0	0	1	0	0	0	0	0	0	1
Cluster1375;	Acidobact	Acidobacteria_Gp3			Gp3	0	0	0	0	0	0	1	0	0	0	0	0	1
Cluster1376;size=3						0	0	0	0	0	0	0	0	0	0	1	0	1
Cluster1378;size=12						0	0	0	1	0	0	0	0	0	0	0	0	1
Cluster1380;	Acidobact	Acidobacteria_Gp2			Gp2	0	0	0	0	0	0	0	0	1	0	0	0	1
Cluster1383;	Acidobact	Acidobacteria_Gp1			Gp1	0	0	0	0	0	1	0	0	0	0	0	0	1
Cluster1389;	Proteobac	Betapr	Burkholderiales			1	0	0	0	0	0	0	0	0	0	0	0	1
Cluster1390;	Acidobact	Acidobacteria_Gp2			Gp2	0	0	0	0	0	0	0	0	0	0	1	0	1
Cluster1397;	Acidobact	Acidobacteria_Gp2			Gp2	0	0	0	0	0	0	0	1	0	0	0	0	1
Cluster1399;size=13						0	0	0	0	0	0	0	1	0	0	0	0	1
Cluster1400;	Proteobac	Alphap	Rhizobiales			0	0	0	0	0	1	0	0	0	0	0	0	1
Cluster1402;	Acidobact	Acidobacteria_Gp1				0	0	0	0	0	0	0	0	0	0	1	0	1
Cluster1407;	Acidobact	Acidobacteria_Gp1			Gp1	0	0	0	1	0	0	0	0	0	0	0	0	1
Cluster1410;	Proteobac	Gamm	Xantl	Xanthomonadaceae		1	0	0	0	0	0	0	0	0	0	0	0	1
Cluster1412;	Proteobac	Alphaproteobacteria				1	0	0	0	0	0	0	0	0	0	0	0	1
Cluster1413;size=1						0	0	0	0	0	0	0	0	0	1	0	0	1
Cluster1414;	Acidobact	Acidobacteria_Gp2			Gp2	0	0	0	0	0	0	0	0	0	0	0	1	1
Cluster1416;	Acidobact	Acidobacteria_Gp1				0	0	0	0	0	0	0	0	0	0	1	0	1
Cluster1417;	Proteobac	Alphap	Rhod	Acetobacteraceae		0	0	0	0	0	0	0	0	0	1	0	0	1
Cluster1424;	Proteobac	Deltaproteobacteria				0	0	0	0	0	0	1	0	0	0	0	0	1
Cluster1427;	Armatimo	Armati	Arma	Armatir	Armatimonas/	0	0	0	0	0	0	0	0	1	0	0	0	1
Cluster1430;	Acidobact	Acidobacteria_Gp2			Gp2	0	0	0	1	0	0	0	0	0	0	0	0	1
Cluster1433;	Proteobac	Gamm	Legio	Coxiella	Aquicella	0	0	0	0	0	0	0	0	0	1	0	0	1
Cluster1434;	Proteobac	Gamm	Pseu	Pseudo	Pseudomonas	0	0	0	0	0	0	0	0	0	0	0	1	1
Cluster1436;	Proteobac	Alphap	Rhizobiales			0	0	0	0	0	0	1	0	0	0	0	0	1
Cluster1441;	Proteobac	Alphap	Rhizobiales			0	0	0	0	0	0	0	0	0	0	1	0	1
Cluster1448;	Acidobact	Acidobacteria_Gp2			Gp2	1	0	0	0	0	0	0	0	0	0	0	0	1
Cluster1450;	Proteobac	Alphap	Rhizo	Rhizobiaceae		0	0	0	0	0	1	0	0	0	0	0	0	1

Cluster1457;	Proteobac	Gammaproteobacteria				0	0	0	0	0	1	0	0	0	0	0	0	1
Cluster1459;	Chlamydia	Chlamy	Chlar	Parachla	Parachlamydia	0	0	0	0	0	0	0	1	0	0	0	0	1
Cluster1462;	Acidobact	Acidobacteria_Gp2			Gp2	0	0	1	0	0	0	0	0	0	0	0	0	1
Cluster1468;	Acidobact	Acidobacteria_Gp2			Gp2	0	0	0	1	0	0	0	0	0	0	0	0	1
Cluster1471;	Proteobac	Alphap	Rhizc	Bradyrhizobiaceae		0	0	0	1	0	0	0	0	0	0	0	0	1
Cluster1473;	TM7				TM7_genera_i	0	0	0	0	0	0	0	0	1	0	0	0	1
Cluster1474;	Armatimc	Chthor	Chthc	Chthonc	Chthonomona	1	0	0	0	0	0	0	0	0	0	0	0	1
Cluster1492;size=1						0	0	1	0	0	0	0	0	0	0	0	0	1
Cluster1496;	Proteobac	Alphaproteobacteria				0	0	1	0	0	0	0	0	0	0	0	0	1
Cluster1497;	Chloroflex	Ktedor	Ktedonobacterales			0	0	0	0	0	0	1	0	0	0	0	0	1
Cluster1499;	Actinobac	Actinol	Soliru	Conexib	Conexibacter	0	0	0	0	0	0	0	0	0	1	0	0	1
Cluster1500;	Proteobac	Gamm	Xantl	Xanthor	Frateuria	0	0	0	0	0	0	0	0	0	1	0	0	1
Cluster1501;	Acidobact	Acidobacteria_Gp3				0	0	0	1	0	0	0	0	0	0	0	0	1
Cluster1503;size=4						0	1	0	0	0	0	0	0	0	0	0	0	1
Cluster1506;	Proteobac	Deltap	Myxc	Cystobacteraceae		0	0	0	0	0	0	1	0	0	0	0	0	1
Cluster1511;	Cyanobac	Chloroplast		Chlorop	Streptophyta	0	0	0	0	0	0	0	0	0	0	0	1	1
Cluster1515;	Proteobac	Alphap	Rhod	Acetobacteraceae		0	0	0	0	0	0	0	0	0	1	0	0	1
Cluster1525;	Acidobact	Acidobacteria_Gp2			Gp2	0	0	0	0	1	0	0	0	0	0	0	0	1
Cluster1527;size=2						0	0	1	0	0	0	0	0	0	0	0	0	1
Cluster1533;	Acidobact	Acidobacteria_Gp3				0	0	0	0	0	0	0	0	0	0	1	0	1
Cluster1535;	Acidobact	Acidobacteria_Gp2			Gp2	0	0	1	0	0	0	0	0	0	0	0	0	1
Cluster1538;	Planctomy	Plancto	Planc	Planctomycetaceae		0	0	0	0	1	0	0	0	0	0	0	0	1
Cluster1540;	Acidobact	Acidobacteria_Gp2			Gp2	0	0	1	0	0	0	0	0	0	0	0	0	1
Cluster1541;	Proteobac	Deltap	Myxococcales			0	0	0	0	0	0	0	0	0	0	1	0	1
Cluster1542;	Proteobacteria					0	0	1	0	0	0	0	0	0	0	0	0	1
Cluster1545;	Proteobac	Alphap	Rhizobiales			0	0	0	1	0	0	0	0	0	0	0	0	1
Cluster1548;	Acidobact	Acidobacteria_Gp2			Gp2	0	0	0	1	0	0	0	0	0	0	0	0	1
Cluster1549;size=3						0	0	0	0	0	0	0	0	0	0	0	1	1
Cluster1557;	Acidobact	Acidobacteria_Gp1			Gp1	0	0	0	1	0	0	0	0	0	0	0	0	1

Cluster1558;:	Acidobact	Acidobacteria_Gp1				1	0	0	0	0	0	0	0	0	0	0	0	1
Cluster1566;:	Proteobac	Alphap	Alphaproteob		Rhizomicrobiu	0	0	0	0	0	1	0	0	0	0	0	0	1
Cluster1568;:	Acidobact	Acidobacteria_Gp2			Gp2	0	0	0	0	0	0	0	0	1	0	0	0	1
Cluster1569;:	Acidobacteria					0	0	1	0	0	0	0	0	0	0	0	0	1
Cluster1571;:	Acidobact	Acidobacteria_Gp3			Gp3	0	1	0	0	0	0	0	0	0	0	0	0	1
Cluster1572;size=1						0	0	0	0	0	0	0	0	0	0	0	1	1
Cluster1573;size=1						0	0	0	0	0	1	0	0	0	0	0	0	1
Cluster1583;:	Proteobac	Betapr	Burkl	Burkholderiaceae		0	0	0	0	0	0	0	0	0	0	0	1	1
Cluster1587;:	Acidobact	Acidobacteria_Gp2			Gp2	0	0	0	0	0	1	0	0	0	0	0	0	1
Cluster1589;:	Proteobac	Deltaproteobacteria				0	0	0	0	0	0	0	1	0	0	0	0	1
Cluster1593;:	Acidobact	Acidobacteria_Gp2			Gp2	0	0	1	0	0	0	0	0	0	0	0	0	1
Cluster1602;size=5						0	0	0	0	1	0	0	0	0	0	0	0	1
Cluster1605;:	Bacteroid	Sphing	Sphir	Chitinop	Sediminibacte	0	0	0	0	0	0	0	1	0	0	0	0	1
Cluster1617;:	Actinobac	Actinol	Solirι	Conexib	Conexibacter	0	0	0	0	0	0	0	1	0	0	0	0	1
Cluster1623;:	Acidobact	Acidobacteria_Gp1			Gp1	0	0	0	0	0	0	0	0	0	0	0	1	1
Cluster1625;:	Proteobac	Gamm	Xantl	Sinobac	Steroidobacter	0	0	0	0	0	1	0	0	0	0	0	0	1
Cluster1642;:	Acidobact	Acidobacteria_Gp3			Gp3	0	0	0	0	1	0	0	0	0	0	0	0	1
Cluster1644;:	Bacteroid	Sphing	Sphir	Chitinophagaceae		0	0	0	0	1	0	0	0	0	0	0	0	1
Cluster1650;:	Acidobact	Acidobacteria_Gp1			Gp1	0	0	0	0	0	1	0	0	0	0	0	0	1
Cluster1651;:	Bacteroid	Sphing	Sphir	Chitinophagaceae		0	0	0	0	1	0	0	0	0	0	0	0	1
Cluster1653;:	Acidobact	Acidobacteria_Gp6			Gp6	0	1	0	0	0	0	0	0	0	0	0	0	1
Cluster1656;:	Proteobac	Alphap	Rhizobiales			0	0	0	0	0	0	0	0	0	0	0	1	1
Cluster1659;:	Bacteroid	Sphing	Sphir	Chitinop	Ferruginibacte	0	1	0	0	0	0	0	0	0	0	0	0	1
Cluster1670;size=1						0	0	0	0	0	0	0	0	0	0	0	1	1
Cluster1671;:	Proteobac	Alphaproteobacteria				0	0	0	0	0	0	1	0	0	0	0	0	1
Cluster1673;:	Acidobact	Acidobacteria_Gp1			Gp1	0	0	0	0	1	0	0	0	0	0	0	0	1
Cluster1682;:	Acidobact	Acidobacteria_Gp2			Gp2	0	0	0	0	0	0	1	0	0	0	0	0	1
Cluster1683;:	Proteobac	Alphap	Rhizc	Rhodob	Tepidamorphu	0	0	0	0	0	0	1	0	0	0	0	0	1
Cluster1684;:	Actinobac	Actinol	Solirubrobacterales			0	0	0	0	1	0	0	0	0	0	0	0	1

Cluster1688;	Acidobact	Acidobacteria_Gp2			Gp2	0	0	0	0	0	0	1	0	0	0	0	0	1
Cluster1689;	Proteobac	Gammaproteobacteria				0	1	0	0	0	0	0	0	0	0	0	0	1
Cluster1691;	Acidobacteria					0	0	0	0	0	1	0	0	0	0	0	0	1
Cluster1696;	Acidobact	Acidobacteria_Gp1				0	0	1	0	0	0	0	0	0	0	0	0	1
Cluster1699;	Acidobact	Acidobacteria_Gp2			Gp2	0	0	0	0	0	0	0	0	0	0	1	0	1
Cluster1700;	Bacteroid	Sphing	Sphir	Sphingc	Mucilaginibact	0	0	0	0	0	0	0	0	0	1	0	0	1
Cluster1708;	Acidobact	Acidobacteria_Gp13			Gp13	1	0	0	0	0	0	0	0	0	0	0	0	1
Cluster1712;	Proteobac	Gammaproteobacteria				0	0	0	1	0	0	0	0	0	0	0	0	1
Cluster1713;	Acidobact	Acidobacteria_Gp1				0	0	0	0	0	0	0	0	0	1	0	0	1
Cluster1719;	Actinobac	Actinobacteria				0	0	0	0	0	0	0	0	0	1	0	0	1
Cluster1720;	Actinobac	Actinol	Actinomycetales			0	0	0	0	0	1	0	0	0	0	0	0	1
Cluster1722;size=1						0	0	1	0	0	0	0	0	0	0	0	0	1
Cluster1729;	Proteobac	Gamm	Legio	Coxiella	Aquicella	0	0	0	0	0	0	0	0	0	0	0	1	1
Cluster1742;	Proteobac	Betaproteobacteria				0	0	0	0	0	0	0	0	0	0	0	1	1
Cluster1746;	Acidobacteria					0	0	0	0	0	0	0	0	0	0	1	0	1
Cluster1747;	Proteobac	Alphap	Caulc	Caulobacteraceae		0	0	0	0	1	0	0	0	0	0	0	0	1
Cluster1748;	Proteobac	Alphap	Rhizc	Hyphon	Rhodoplanes	0	0	0	0	0	0	0	1	0	0	0	0	1
Cluster1749;	Proteobac	Alphap	Caulc	Caulobа	Phenylobacter	0	0	0	1	0	0	0	0	0	0	0	0	1
Cluster1750;	Proteobac	Betapr	Burkl	Burkholderiaceae		0	0	1	0	0	0	0	0	0	0	0	0	1
Cluster1755;	OD1				OD1_genera_i	0	1	0	0	0	0	0	0	0	0	0	0	1
Cluster1758;	Actinobac	Actinol	Actinomycetales			0	0	0	0	0	0	0	0	0	1	0	0	1
Cluster1760;	Acidobact	Acidobacteria_Gp1			Gp1	0	0	1	0	0	0	0	0	0	0	0	0	1
Cluster1765;	Acidobact	Acidobacteria_Gp3				0	0	0	0	0	0	0	0	0	1	0	0	1
Cluster1769;	Actinobac	Actinol	Acidimicrobiales			0	1	0	0	0	0	0	0	0	0	0	0	1
Cluster1770;	Proteobac	Deltaproteobacteria				0	0	1	0	0	0	0	0	0	0	0	0	1
Cluster1772;	Proteobac	Gammaproteobacteria				0	0	0	1	0	0	0	0	0	0	0	0	1
Cluster1775;size=3						0	0	0	0	0	0	0	0	0	0	0	1	1
Cluster1776;size=4						0	0	0	0	0	0	0	0	0	0	1	0	1
Cluster1779;	Proteobac	Alphap	Rhod	Acetoba	Acidisphaera	0	0	0	0	0	0	0	0	0	0	1	0	1

Cluster1781;size=5						0	0	0	0	0	0	0	0	0	1	0	0	1
Cluster1785;size=3						0	0	1	0	0	0	0	0	0	0	0	0	1
Cluster1786;	Acidobact	Acidobacteria_Gp3			Gp3	0	0	0	0	0	0	0	0	0	1	0	0	1
Cluster1792;	Acidobact	Acidobacteria_Gp2			Gp2	0	0	0	0	0	0	1	0	0	0	0	0	1
Cluster1797;	Acidobact	Acidobacteria_Gp1			Gp1	0	0	0	0	0	0	1	0	0	0	0	0	1
Cluster1798;	Proteobac	Alphap	Rhodospirillales			0	0	0	0	0	0	0	0	0	0	1	0	1
Cluster1808;	Proteobac	Gammaproteobacteria				0	0	0	1	0	0	0	0	0	0	0	0	1
Cluster1809;	Chlamydia	Chlamy	Chlar	Parachlamydiaceae		0	1	0	0	0	0	0	0	0	0	0	0	1
Cluster1812;	Proteobac	Gamm	Legio	Coxiella	Coxiella	0	0	0	0	0	0	1	0	0	0	0	0	1
Cluster1822;	Proteobac	Betaproteobacteria				0	0	0	0	0	0	0	0	1	0	0	0	1
Cluster1825;	Proteobac	Alphap	Rhizobiales			1	0	0	0	0	0	0	0	0	0	0	0	1
Cluster1831;	Acidobacteria					0	0	0	0	0	0	0	1	0	0	0	0	1
Cluster1837;	Proteobac	Alphap	Rhizc	Xanthol	Labrys	0	1	0	0	0	0	0	0	0	0	0	0	1
Cluster1839;size=1						1	0	0	0	0	0	0	0	0	0	0	0	1
Cluster1843;	Firmicutes	Bacilli	Bacill	Bacillac	Bacillus	0	0	0	0	0	0	0	0	0	1	0	0	1
Cluster1845;size=4						0	0	0	0	0	0	0	0	1	0	0	0	1
Cluster1859;	Acidobact	Acidobacteria_Gp1				0	0	1	0	0	0	0	0	0	0	0	0	1
Cluster1861;	Acidobact	Acidobacteria_Gp2			Gp2	0	0	0	0	0	0	0	0	0	0	1	0	1
Cluster1863;size=13						0	0	0	0	0	0	0	0	0	0	0	1	1
Cluster1875;	Chlamydia	Chlamy	Chlar	Parachlamydiaceae		0	1	0	0	0	0	0	0	0	0	0	0	1
Cluster1877;	Acidobact	Acidobacteria_Gp1				0	0	0	0	1	0	0	0	0	0	0	0	1
Cluster1878;	Proteobac	Deltaproteobacteria				0	0	0	0	1	0	0	0	0	0	0	0	1
Cluster1882;size=2						0	0	0	1	0	0	0	0	0	0	0	0	1
Cluster1885;size=5						0	0	0	0	0	0	0	0	0	0	1	0	1
Cluster1887;	Acidobact	Acidobacteria_Gp10			Gp10	0	1	0	0	0	0	0	0	0	0	0	0	1
Cluster1892;	Proteobac	Alphap	Alphaproteol		Rhizomicrobiu	0	0	0	0	0	0	1	0	0	0	0	0	1
Cluster1894;	Planctomy	Plancto	Planc	Planctomycetaceae		0	0	0	0	0	0	0	0	1	0	0	0	1
Cluster1897;	Acidobact	Acidobacteria_Gp2			Gp2	0	0	0	0	0	0	0	0	0	0	1	0	1
Cluster1899;	Acidobact	Acidobacteria_Gp1				0	0	0	0	0	0	0	0	0	1	0	0	1

Cluster1903;	Planctomycetes					1	0	0	0	0	0	0	0	0	0	0	0	1
Cluster1905;	Proteobacteria					0	0	0	0	0	0	0	0	1	0	0	0	1
Cluster1915;	Acidobact	Acidobacteria_Gp3			Gp3	0	0	0	0	0	0	0	0	0	0	1	0	1
Cluster1916;	Proteobac	Alphap	Rhizobiales			0	0	1	0	0	0	0	0	0	0	0	0	1
Cluster1918;	Proteobac	Alphap	Rhizo	Xanthol	Pseudolabrys	0	0	0	0	1	0	0	0	0	0	0	0	1
Cluster1930;	Actinobac	Actinol	Actin	Actinos	Actinospica	0	0	0	0	0	0	0	0	0	0	0	1	1
Cluster1936;	Acidobact	Acidobacteria_Gp3			Gp3	0	0	0	0	0	1	0	0	0	0	0	0	1
Cluster1938;	Bacteroid	Sphing	Sphir	Chitinophagaceae		0	0	0	0	0	0	0	0	0	0	0	1	1
Cluster1947;	Verrucom	Opituta	Punic	Puniceic	Coraliomargari	1	0	0	0	0	0	0	0	0	0	0	0	1
Cluster1969;	Proteobac	Alphap	Rhizo	Bradyrh	Agromonas	0	0	0	1	0	0	0	0	0	0	0	0	1
Cluster1971;	Proteobac	Betaproteobacteria				0	0	0	0	0	0	0	0	0	1	0	0	1
Cluster1973;	Acidobact	Acidobacteria_Gp3			Gp3	0	1	0	0	0	0	0	0	0	0	0	0	1
Cluster1976;size=9						0	0	0	0	1	0	0	0	0	0	0	0	1
Cluster1979;	Bacteroidetes					1	0	0	0	0	0	0	0	0	0	0	0	1
Cluster1981;	Acidobacteria					1	0	0	0	0	0	0	0	0	0	0	0	1
Cluster1983;size=1						0	1	0	0	0	0	0	0	0	0	0	0	1
Cluster1990;	Proteobac	Alphaproteobacteria				0	1	0	0	0	0	0	0	0	0	0	0	1
Cluster1992;	Proteobac	Alphap	Caulo	Caulobacteraceae		1	0	0	0	0	0	0	0	0	0	0	0	1
Cluster1998;	Acidobact	Acidobacteria_Gp1			Gp1	0	0	0	0	0	0	0	0	0	0	0	1	1
Cluster2002;	Proteobac	Alphap	Rhodospirillales			0	0	0	0	0	1	0	0	0	0	0	0	1
Cluster2003;	Acidobact	Acidobacteria_Gp5			Gp5	0	0	0	0	0	0	0	0	0	0	0	1	1
Cluster2004;size=3						1	0	0	0	0	0	0	0	0	0	0	0	1
Cluster2011;size=2						0	0	0	0	0	0	0	0	1	0	0	0	1
Cluster2015;	Proteobac	Alphap	Rhod	Acetoba	Acidisphaera	0	0	0	1	0	0	0	0	0	0	0	0	1
Cluster2022;size=10						0	0	0	0	0	0	0	0	1	0	0	0	1
Cluster2024;	Proteobac	Betapr	Burkl	Burkhol	Burkholderia	0	0	0	0	1	0	0	0	0	0	0	0	1
Cluster2025;	Acidobact	Acidobacteria_Gp1				0	0	1	0	0	0	0	0	0	0	0	0	1
Cluster2029;	Proteobac	Alphap	Caulo	Caulob	Asticcacaulis	0	0	0	0	0	0	0	0	0	1	0	0	1
Cluster2034;size=1						0	0	0	0	1	0	0	0	0	0	0	0	1

Cluster2048;size=1						0	0	0	0	0	0	0	0	1	0	0	0	1
Cluster2052;:	Proteobac	Gammaproteobacteria				0	0	0	0	0	0	0	0	0	0	1	0	1
Cluster2054;:	Acidobact	Acidobacteria_Gp3			Bryobacter	1	0	0	0	0	0	0	0	0	0	0	0	1
Cluster2055;:	Acidobact	Acidobacteria_Gp1			Gp1	0	0	0	0	0	0	0	0	0	1	0	0	1
Cluster2059;size=4						0	0	0	0	0	0	0	0	0	0	1	0	1
Cluster2065;:	Proteobacteria					1	0	0	0	0	0	0	0	0	0	0	0	1
Cluster2074;:	Acidobact	Acidobacteria_Gp2			Gp2	0	1	0	0	0	0	0	0	0	0	0	0	1
Cluster2075;:	Proteobac	Alphap	Caulo	Caulobacteraceae		0	0	0	0	0	0	0	0	0	0	1	0	1
Cluster2081;:	Proteobac	Betapr	Burkl	Burkholderiaceae		0	0	0	0	0	0	0	0	0	0	0	1	1
Cluster2083;size=3						0	0	0	0	0	0	0	0	0	0	1	0	1
Cluster2092;:	Proteobac	Gamm	Xantl	Sinobacteraceae		0	1	0	0	0	0	0	0	0	0	0	0	1
Cluster2095;:	Firmicute:	Clostri	Clost	Peptost	Sporacetigeniu	0	0	0	0	0	0	1	0	0	0	0	0	1
Cluster2100;:	Bacteroid	Sphing	Sphir	Sphingo	Mucilaginibact	0	0	0	0	1	0	0	0	0	0	0	0	1
Cluster2104;:	Acidobact	Acidobacteria_Gp1			Gp1	0	0	0	0	0	0	0	0	0	0	1	0	1
Cluster2112;:	Verrucom	Opitut:	Opitu	Opituta	Opitutus	0	0	0	0	0	0	0	0	0	1	0	0	1
Cluster2113;:	Acidobact	Acidobacteria_Gp1			Gp1	1	0	0	0	0	0	0	0	0	0	0	0	1
Cluster2119;:	Planctomycetes					1	0	0	0	0	0	0	0	0	0	0	0	1
Cluster2122;:	Acidobact	Acidobacteria_Gp2			Gp2	0	0	0	0	0	0	0	0	0	0	1	0	1
Cluster2126;:	Acidobact	Acidobacteria_Gp1				0	0	1	0	0	0	0	0	0	0	0	0	1
Cluster2127;:	Actinobac	Actinol	Soliru	Conexib	Conexibacter	0	0	0	0	0	0	0	0	0	0	1	0	1
Cluster2132;:	Proteobac	Deltaproteobacteria				0	0	0	0	0	1	0	0	0	0	0	0	1
Cluster2137;:	Acidobact	Acidobacteria_Gp3			Gp3	0	0	1	0	0	0	0	0	0	0	0	0	1
Cluster2139;:	Verrucom	Subdivision3			Subdivision3_g	1	0	0	0	0	0	0	0	0	0	0	0	1
Cluster2141;:	Proteobac	Alphaproteobacteria				0	0	0	0	0	0	0	0	1	0	0	0	1
Cluster2153;:	Acidobact	Acidobacteria_Gp1				0	0	0	0	0	0	0	0	0	0	0	1	1
Cluster2160;:	Bacteroid	Sphing	Sphir	Chitinophagaceae		1	0	0	0	0	0	0	0	0	0	0	0	1
Cluster2162;:	Proteobacteria					0	0	0	0	0	0	0	0	0	1	0	0	1
Cluster2169;:	Proteobac	Alphap	Rhizobiales			0	0	0	1	0	0	0	0	0	0	0	0	1
Cluster2175;:	Proteobac	Deltaproteobacteria				0	1	0	0	0	0	0	0	0	0	0	0	1

Cluster2193;	Acidobact	Acidobacteria_Gp2			Gp2	0	0	0	0	0	0	0	0	0	0	1	0	1
Cluster2196;	Acidobact	Acidobacteria_Gp2			Gp2	0	0	1	0	0	0	0	0	0	0	0	0	1
Cluster2204;	Acidobact	Acidobacteria_Gp1				0	0	0	0	1	0	0	0	0	0	0	0	1
Cluster2205;	Planctomy	Plancto	Planc	Plancto	Singulisphaera	0	0	0	0	0	0	0	0	0	0	0	1	1
Cluster2216;size=8						0	0	0	1	0	0	0	0	0	0	0	0	1
Cluster2219;	Planctomy	Plancto	Planc	Planctomycetaceae		0	0	0	1	0	0	0	0	0	0	0	0	1
Cluster2223;	Proteobac	Alphap	Rhod	Acetobacteraceae		0	0	1	0	0	0	0	0	0	0	0	0	1
Cluster2226;size=6						0	0	0	0	0	0	0	0	0	0	0	1	1
Cluster2231;size=10						1	0	0	0	0	0	0	0	0	0	0	0	1
Cluster2238;	Acidobact	Acidobacteria_Gp1				0	0	0	0	1	0	0	0	0	0	0	0	1
Cluster2240;	Gemmatir	Gemm	Gemı	Gemma	Gemmatimona	0	0	0	0	0	0	0	0	0	0	0	1	1
Cluster2242;	Proteobac	Alphaproteobacteria				0	0	0	0	0	0	0	0	0	1	0	0	1
Cluster2256;	Chloroflex	Ktedor	Ktedo	Ktedono	Ktedonobacter	0	1	0	0	0	0	0	0	0	0	0	0	1
Cluster2261;	Acidobact	Acidobacteria_Gp1				0	1	0	0	0	0	0	0	0	0	0	0	1
Cluster2263;	Proteobac	Alphap	Rhizobiales			0	0	0	0	0	0	1	0	0	0	0	0	1
Cluster2273;	Proteobac	Alphap	Rhodospirillales			0	0	0	0	0	1	0	0	0	0	0	0	1
Cluster2292;	Proteobac	Alphaproteobacteria				0	0	0	1	0	0	0	0	0	0	0	0	1
Cluster2296;	Actinobac	Actinol	Acidi	Acidimi	Aciditerrimona	0	0	0	1	0	0	0	0	0	0	0	0	1
Cluster2298;	Proteobac	Gamm	Legio	Coxiella	Aquicella	0	1	0	0	0	0	0	0	0	0	0	0	1
Cluster2301;	Acidobact	Acidobacteria_Gp2			Gp2	0	0	0	1	0	0	0	0	0	0	0	0	1
Cluster2302;	Proteobac	Deltaproteobacteria				0	0	0	1	0	0	0	0	0	0	0	0	1
Cluster2307;	Proteobac	Alphap	Caulc	Cauloba	Brevundimona	1	0	0	0	0	0	0	0	0	0	0	0	1
Cluster2309;	Proteobac	Alphap	Rhodospirillales			0	0	0	0	0	1	0	0	0	0	0	0	1
Cluster2311;	Proteobac	Alphap	Rhizobiales			0	0	0	0	0	0	0	0	0	0	1	0	1
Cluster2314;	Acidobact	Acidobacteria_Gp3			Gp3	0	0	0	0	0	0	0	0	0	1	0	0	1
Cluster2321;	Proteobacteria					0	0	0	0	0	1	0	0	0	0	0	0	1
Cluster2328;	Acidobact	Acidobacteria_Gp2			Gp2	0	0	0	0	1	0	0	0	0	0	0	0	1
Cluster2335;	Proteobac	Deltap	Myxc	Polyangiaceae		0	0	0	0	0	0	0	0	0	1	0	0	1
Cluster2349;	Acidobact	Acidobacteria_Gp3				0	0	0	0	0	0	0	0	0	0	1	0	1

Cluster2350;:	Elusimicro	Elusim	Elusi	Elusimic	Elusimicrobiun	0	0	1	0	0	0	0	0	0	0	0	0	1
Cluster2353;size=1						0	0	0	0	0	0	0	0	0	0	0	1	1
Cluster2355;:	Proteobac	Alphap	Rhizobiales			0	0	0	0	0	0	1	0	0	0	0	0	1
Cluster2362;size=1						0	0	0	0	0	0	0	0	0	0	0	1	1
Cluster2365;:	Verrucom	Opituta	Opitu	Opituta	Opitutus	0	0	0	0	0	0	0	1	0	0	0	0	1
Cluster2367;:	Acidobact	Acidobacteria_Gp3			Gp3	1	0	0	0	0	0	0	0	0	0	0	0	1
Cluster2368;:	Gemmatir	Gemm	Gemı	Gemma	Gemmatimona	0	0	0	0	0	0	1	0	0	0	0	0	1
Cluster2369;:	Planctomy	Plancto	Planc	Plancto	Planctomyces	0	0	0	0	0	0	1	0	0	0	0	0	1
Cluster2372;:	Proteobac	Alphap	Rhod	Rhodos	Dongia	0	0	0	0	0	1	0	0	0	0	0	0	1
Cluster2379;:	Proteobac	Gamm	Xanth	Sinobac	Steroidobacter	1	0	0	0	0	0	0	0	0	0	0	0	1
Cluster2380;:	Bacteroid	Sphing	Sphir	Sphingo	Mucilaginibact	0	1	0	0	0	0	0	0	0	0	0	0	1
Cluster2382;:	Acidobact	Acidobacteria_Gp1				0	0	0	1	0	0	0	0	0	0	0	0	1
Cluster2391;:	Acidobact	Acidobacteria_Gp1			Gp1	0	0	0	0	0	0	0	0	0	1	0	0	1
Cluster2393;:	Acidobact	Acidobacteria_Gp3			Gp3	0	0	0	0	0	0	0	0	0	0	1	0	1
Cluster2394;size=1						0	0	0	0	0	0	0	0	0	0	1	0	1
Cluster2395;:	Chloroflex	Ktedor	Ktedonobacterales			0	0	0	0	1	0	0	0	0	0	0	0	1
Cluster2396;:	Actinobac	Actinol	Actinomycetales			0	0	0	0	1	0	0	0	0	0	0	0	1
Cluster2398;size=4						0	0	0	0	0	0	0	0	0	0	0	1	1
Cluster2399;:	Proteobac	Alphap	Rhizo	Hyphomicrobiaceae		0	0	0	0	0	1	0	0	0	0	0	0	1
Cluster2402;:	Proteobacteria					0	0	0	0	0	0	1	0	0	0	0	0	1
Cluster2403;:	Acidobact	Acidobacteria_Gp1				0	0	0	0	1	0	0	0	0	0	0	0	1
Cluster2408;:	Acidobact	Acidobacteria_Gp1				0	0	0	0	0	0	0	0	0	0	1	0	1
Cluster2413;:	Proteobac	Deltaproteobacteria				1	0	0	0	0	0	0	0	0	0	0	0	1
Cluster2428;:	Acidobact	Acidobacteria_Gp1			Granulicella	0	0	0	0	0	0	0	0	0	1	0	0	1
Cluster2433;:	Proteobac	Gammaproteobacteria				0	1	0	0	0	0	0	0	0	0	0	0	1
Cluster2436;:	Bacteroidetes					0	0	0	0	1	0	0	0	0	0	0	0	1
Cluster2437;:	Proteobac	Gammaproteobacteria				0	0	0	0	1	0	0	0	0	0	0	0	1
Cluster2440;:	Proteobac	Gamm	Legio	Coxiella	Aquicella	0	0	0	0	1	0	0	0	0	0	0	0	1
Cluster2444;size=1						0	0	0	0	1	0	0	0	0	0	0	0	1

Cluster2445;	Proteobac	Alphap	Rhizobiales			0	0	0	0	0	0	0	0	0	1	0	0	1
Cluster2453;	Proteobac	Alphap	Rhodospirillales			0	0	0	0	0	0	0	1	0	0	0	0	1
Cluster2457;	Proteobac	Alphap	Caulc	Cauloba	Caulobacter	0	0	0	0	0	0	0	0	0	1	0	0	1
Cluster2459;	Proteobac	Alphap	Rhizobiales			1	0	0	0	0	0	0	0	0	0	0	0	1
Cluster2462;	Proteobac	Deltap	Myxococcales			1	0	0	0	0	0	0	0	0	0	0	0	1
Cluster2467;	Proteobac	Alphap	Alphaproteok		Rhizomicrobiu	0	0	0	0	0	0	0	0	0	0	1	0	1
Cluster2475;	Acidobact	Acidobacteria_Gp1				0	0	1	0	0	0	0	0	0	0	0	0	1
Cluster2476;	Acidobact	Acidobacteria_Gp3			Gp3	0	0	0	0	0	0	0	0	0	0	1	0	1
Cluster2478;	Actinobac	Actinobacteria				0	0	1	0	0	0	0	0	0	0	0	0	1
Cluster2488;	Bacteroid	Sphing	Sphir	Chitinophagaceae		0	0	1	0	0	0	0	0	0	0	0	0	1
Cluster2494;	Armatimc	Chthor	Chth	Chthonc	Chthonomona	0	0	1	0	0	0	0	0	0	0	0	0	1
Cluster2506;size=1						0	0	0	0	1	0	0	0	0	0	0	0	1
Cluster2511;	Proteobac	Alphaproteobacteria				0	0	0	0	0	0	0	0	0	1	0	0	1
Cluster2513;	Verrucom	Spartobacteria			Spartobacteria	0	0	0	0	0	0	1	0	0	0	0	0	1
Cluster2516;	Proteobac	Betapr	Burkl	Comamonadaceae		0	0	1	0	0	0	0	0	0	0	0	0	1
Cluster2518;	Proteobac	Betapr	Burkl	Burkhol	Burkholderia	0	0	0	0	0	1	0	0	0	0	0	0	1
Cluster2525;	Acidobact	Acidobacteria_Gp2			Gp2	0	0	0	0	0	0	0	0	0	0	0	1	1
Cluster2526;	Proteobac	Alphap	Rhod	Acetobacteraceae		0	0	0	0	0	0	0	0	0	0	1	0	1
Cluster2529;	Actinobac	Actinol	Soliru	Conexib	Conexibacter	1	0	0	0	0	0	0	0	0	0	0	0	1
Cluster2538;	Chlamydia	Chlamy	Chlar	Parachla	Parachlamydia	1	0	0	0	0	0	0	0	0	0	0	0	1
Cluster2540;	Proteobac	Alphap	Rhodospirillales			0	0	0	0	1	0	0	0	0	0	0	0	1
Cluster2544;	Proteobac	Deltap	Bdell	Bdellovi	Bdellovibrio	0	0	1	0	0	0	0	0	0	0	0	0	1
Cluster2550;	Acidobact	Acidobacteria_Gp2			Gp2	0	0	0	0	0	0	0	1	0	0	0	0	1
Cluster2559;	Chloroflexi					0	0	0	0	0	0	0	0	1	0	0	0	1
Cluster2566;	Acidobact	Acidobacteria_Gp1				1	0	0	0	0	0	0	0	0	0	0	0	1
Cluster2567;	Verrucom	Spartobacteria			Spartobacteria	0	0	0	0	0	0	1	0	0	0	0	0	1
Cluster2569;	Acidobact	Acidobacteria_Gp2			Gp2	0	0	0	1	0	0	0	0	0	0	0	0	1
Cluster2570;	Proteobac	Gammaproteobacteria				0	0	0	0	0	0	0	0	1	0	0	0	1
Cluster2580;size=3						0	1	0	0	0	0	0	0	0	0	0	0	1

Cluster2586;	Acidobact	Acidobacteria_Gp1				0	1	0	0	0	0	0	0	0	0	0	0	1
Cluster2593;size=1						0	0	0	0	0	0	0	0	0	0	0	1	1
Cluster2598;	Proteobac	Gammaproteobacteria				0	1	0	0	0	0	0	0	0	0	0	0	1
Cluster2600;	Acidobact	Acidobacteria_Gp1			Gp1	1	0	0	0	0	0	0	0	0	0	0	0	1
Cluster2606;size=12						0	0	1	0	0	0	0	0	0	0	0	0	1
Cluster2608;	Acidobact	Acidobacteria_Gp3				0	0	0	0	0	0	0	0	0	1	0	0	1
Cluster2626;	Acidobact	Acidobacteria_Gp2			Gp2	0	0	0	1	0	0	0	0	0	0	0	0	1
Cluster2629;	Actinobac	Actinol	Actin	Thermomonosporacea		0	0	0	0	0	0	0	0	0	0	1	0	1
Cluster2634;	Chlamydia	Chlamy	Chlamydiales			0	0	0	0	0	0	0	0	0	1	0	0	1
Cluster2636;	Verrucom	Subdivision3			Subdivision3_g	1	0	0	0	0	0	0	0	0	0	0	0	1
Cluster2641;	Proteobac	Betaproteobacteria				0	0	1	0	0	0	0	0	0	0	0	0	1
Cluster2644;size=4						0	0	0	1	0	0	0	0	0	0	0	0	1
Cluster2653;	Proteobac	Alphap	Caulc	Caulobacteraceae		0	0	0	1	0	0	0	0	0	0	0	0	1
Cluster2657;	Actinobac	Actinol	Actin	Mycoba	Mycobacteriur	1	0	0	0	0	0	0	0	0	0	0	0	1
Cluster2661;size=3						0	0	0	0	0	1	0	0	0	0	0	0	1
Cluster2662;	Proteobac	Deltaproteobacteria				0	0	0	0	0	0	0	0	0	1	0	0	1
Cluster2663;	Bacteroid	Sphing	Sphir	Sphingc	Mucilaginibact	0	0	0	0	0	0	0	1	0	0	0	0	1
Cluster2667;	Proteobac	Alphap	Rhodospirillales			0	0	0	0	0	0	1	0	0	0	0	0	1
Cluster2671;size=2						0	0	0	0	1	0	0	0	0	0	0	0	1
Cluster2672;	Bacteroid	Sphing	Sphir	Chitinophagaceae		0	1	0	0	0	0	0	0	0	0	0	0	1
Cluster2679;	Acidobact	Acidobacteria_Gp2			Gp2	0	0	0	1	0	0	0	0	0	0	0	0	1
Cluster2683;	Acidobact	Acidobacteria_Gp2			Gp2	0	0	0	0	0	0	0	0	1	0	0	0	1
Cluster2686;	Acidobact	Acidobacteria_Gp2			Gp2	0	0	1	0	0	0	0	0	0	0	0	0	1
Cluster2690;	Acidobact	Acidobacteria_Gp2			Gp2	1	0	0	0	0	0	0	0	0	0	0	0	1
Cluster2701;	Bacteroid	Sphing	Sphir	Chitinophagaceae		0	0	0	0	1	0	0	0	0	0	0	0	1
Cluster2708;size=1						0	1	0	0	0	0	0	0	0	0	0	0	1
Cluster2722;	Proteobacteria					0	0	0	0	0	0	0	1	0	0	0	0	1
Cluster2725;	Acidobact	Acidobacteria_Gp2			Gp2	0	0	0	0	0	0	1	0	0	0	0	0	1
Cluster2727;	Acidobact	Acidobacteria_Gp1			Acidobacteriur	0	0	0	0	0	0	0	1	0	0	0	0	1

Cluster2729;	Chlamydia	Chlamy	Chlamydiales			0	0	0	0	1	0	0	0	0	0	0	0	1
Cluster2732;	Proteobac	Alphap	Rhodospirillales			0	0	0	0	1	0	0	0	0	0	0	0	1
Cluster2734;	Proteobac	Alphap	Rhodospirillales			0	0	0	0	0	0	0	0	0	0	0	1	1
Cluster2737;	Acidobact	Acidobacteria_Gp2			Gp2	0	0	0	1	0	0	0	0	0	0	0	0	1
Cluster2741;size=5						0	0	0	0	1	0	0	0	0	0	0	0	1
Cluster2747;size=1						1	0	0	0	0	0	0	0	0	0	0	0	1
Cluster2758;	Acidobact	Acidobacteria_Gp2			Gp2	0	0	0	0	0	0	1	0	0	0	0	0	1
Cluster2763;	Proteobac	Alphap	Rhizobiales			0	0	0	0	1	0	0	0	0	0	0	0	1
Cluster2767;	Acidobact	Acidobacteria_Gp1			Gp1	0	0	0	0	0	0	0	0	0	1	0	0	1
Cluster2771;	Acidobact	Acidobacteria_Gp13			Gp13	0	0	0	0	0	0	0	0	1	0	0	0	1
Cluster2776;	Acidobact	Acidobacteria_Gp1				0	0	0	0	0	0	0	0	0	1	0	0	1
Cluster2784;	Proteobac	Alphap	Caulc	Caulobacteraceae		0	0	0	0	0	0	0	0	0	0	1	0	1
Cluster2785;	Actinobac	Actinol	Actinomycetales			0	0	0	0	1	0	0	0	0	0	0	0	1
Cluster2789;	Proteobac	Alphap	Rhodospirillales			0	0	0	0	0	0	0	0	1	0	0	0	1
Cluster2790;	Actinobac	Actinol	Actinomycetales			0	0	0	0	0	0	1	0	0	0	0	0	1
Cluster2807;	Proteobac	Alphaproteobacteria				0	0	0	0	0	0	0	0	0	1	0	0	1
Cluster2819;	Proteobac	Gammaproteobacteria				0	0	0	0	0	1	0	0	0	0	0	0	1
Cluster2820;	Acidobact	Acidobacteria_Gp1				0	0	0	0	0	0	0	0	0	1	0	0	1
Cluster2824;	Acidobact	Acidobacteria_Gp3			Gp3	0	0	0	1	0	0	0	0	0	0	0	0	1
Cluster2829;	Proteobac	Alphap	Alphaproteok		Rhizomicrobiu	0	0	0	0	1	0	0	0	0	0	0	0	1
Cluster2840;	Acidobact	Acidobacteria_Gp2			Gp2	0	0	0	0	1	0	0	0	0	0	0	0	1
Cluster2844;	Proteobac	Deltap	Myxococcales			0	0	0	0	0	0	0	1	0	0	0	0	1
Cluster2847;	Proteobac	Betapr	Burkholderiales			0	0	0	0	0	0	0	1	0	0	0	0	1
Cluster2851;	Acidobact	Acidobacteria_Gp2			Gp2	0	0	0	0	0	1	0	0	0	0	0	0	1
Cluster2854;size=3						1	0	0	0	0	0	0	0	0	0	0	0	1
Cluster2859;	Proteobacteria					0	0	0	0	1	0	0	0	0	0	0	0	1
Cluster2866;size=6						0	0	0	0	0	0	0	0	0	0	0	1	1
Cluster2879;	Acidobact	Acidobacteria_Gp2			Gp2	0	0	0	0	0	0	0	0	0	0	0	1	1
Cluster2893;	Bacteroid	Sphing	Sphir	Sphingc	Mucilaginibact	0	0	0	0	1	0	0	0	0	0	0	0	1

Cluster2894;	Proteobac	Alphaproteobacteria				0	0	0	0	0	0	0	0	1	0	0	0	1
Cluster2896;	Proteobac	Alphaproteobacteria				0	0	0	1	0	0	0	0	0	0	0	0	1
Cluster2898;	Bacteroid	Sphing	Sphir	Sphingobacteriaceae		1	0	0	0	0	0	0	0	0	0	0	0	1
Cluster2904;	Acidobact	Acidobacteria_Gp1			Acidobacteriur	0	0	0	1	0	0	0	0	0	0	0	0	1
Cluster2919;	Proteobac	Deltap	Myxc	Cystobacteraceae		0	0	0	0	0	0	0	0	1	0	0	0	1
Cluster2921;	Acidobact	Acidobacteria_Gp1			Edaphobacter	0	0	0	1	0	0	0	0	0	0	0	0	1
Cluster2924;	Proteobac	Alphap	Rhizc	Hyphon	Rhodomicrobiu	0	0	0	0	1	0	0	0	0	0	0	0	1
Cluster2934;size=1						0	0	0	0	0	0	0	1	0	0	0	0	1
Cluster2937;size=1						0	1	0	0	0	0	0	0	0	0	0	0	1
Cluster2940;	Gemmatir	Gemm	Gemı	Gemma	Gemmatimona	0	0	0	0	0	0	0	0	0	0	0	1	1
Cluster2951;size=1						0	1	0	0	0	0	0	0	0	0	0	0	1
Cluster2953;	Proteobacteria					0	0	0	0	0	0	0	0	0	0	0	1	1
Cluster2955;	Acidobact	Acidobacteria_Gp2			Gp2	0	0	0	0	0	0	1	0	0	0	0	0	1
Cluster2959;	Proteobac	Deltaproteobacteria				0	1	0	0	0	0	0	0	0	0	0	0	1
Cluster2961;	Acidobact	Acidobacteria_Gp3				0	0	1	0	0	0	0	0	0	0	0	0	1
Cluster2962;size=1						0	0	0	0	0	0	0	1	0	0	0	0	1
Cluster2963;size=2						0	0	0	0	0	0	0	0	1	0	0	0	1
Cluster2977;	Proteobac	Alphap	Rhizc	Bradyrh	Agromonas	0	0	0	0	0	0	0	0	0	1	0	0	1
Cluster2982;	Proteobac	Gammaproteobacteria				0	0	0	0	1	0	0	0	0	0	0	0	1
Cluster2983;size=5						0	0	0	0	0	0	0	0	1	0	0	0	1
Cluster2993;	Acidobact	Acidobacteria_Gp2			Gp2	0	0	1	0	0	0	0	0	0	0	0	0	1
Cluster2995;	Acidobact	Acidobacteria_Gp2			Gp2	0	0	0	0	0	0	0	0	1	0	0	0	1
Cluster3000;size=1						0	0	0	0	0	0	0	0	0	1	0	0	1
Cluster3005;	Acidobact	Acidobacteria_Gp1			Gp1	0	0	0	0	0	0	0	0	0	0	1	0	1
Cluster3015;	Acidobact	Acidobacteria_Gp1			Gp1	0	0	0	0	0	0	0	0	0	0	1	0	1
Cluster3018;size=4						0	0	0	0	0	1	0	0	0	0	0	0	1
Cluster3028;	Proteobac	Gammaproteobacteria				0	0	0	0	1	0	0	0	0	0	0	0	1
Cluster3031;	Acidobact	Acidobacteria_Gp2			Gp2	0	0	0	1	0	0	0	0	0	0	0	0	1
Cluster3033;	Proteobac	Gamm	Xantl	Sinobac	Steroidobacter	1	0	0	0	0	0	0	0	0	0	0	0	1

Cluster3034;	Acidobact	Acidobacteria_Gp1				0	0	1	0	0	0	0	0	0	0	0	0	1
Cluster3038;	Proteobac	Deltaproteobacteria				1	0	0	0	0	0	0	0	0	0	0	0	1
Cluster3041;	Acidobacteria					0	0	0	0	0	0	0	0	0	0	0	1	1
Cluster3042;	Proteobac	Alphap	Rhod	Acetobacteraceae		1	0	0	0	0	0	0	0	0	0	0	0	1
Cluster3047;	Proteobac	Alphaproteobacteria				1	0	0	0	0	0	0	0	0	0	0	0	1
Cluster3049;	Acidobact	Acidobacteria_Gp3			Gp3	0	0	0	0	0	0	0	0	0	0	0	1	1
Cluster3050;	Bacteroid	Sphing	Sphir	Chitinophagaceae		1	0	0	0	0	0	0	0	0	0	0	0	1
Cluster3052;size=1						1	0	0	0	0	0	0	0	0	0	0	0	1
Cluster3055;size=2						0	0	0	0	0	0	0	1	0	0	0	0	1
Cluster3057;	Proteobac	Alphap	Caulc	Caulob	Phenylobacter	0	0	0	0	0	0	0	0	0	1	0	0	1
Cluster3060;size=2						0	0	0	0	0	1	0	0	0	0	0	0	1
Cluster3061;	Actinobac	Actinol	Actin	Micrococ	Rothia	0	1	0	0	0	0	0	0	0	0	0	0	1
Cluster3062;	Acidobact	Acidobacteria_Gp13			Gp13	0	0	0	0	1	0	0	0	0	0	0	0	1
Cluster3067;	Proteobac	Betapr	Burkl	Burkhol	Burkholderia	0	0	1	0	0	0	0	0	0	0	0	0	1
Cluster3071;	Acidobact	Acidobacteria_Gp1				0	0	1	0	0	0	0	0	0	0	0	0	1
Cluster3080;size=1						1	0	0	0	0	0	0	0	0	0	0	0	1
Cluster3083;	Proteobac	Gamm	Xantl	Sinobac	Steroidobacter	0	0	0	0	0	0	0	0	1	0	0	0	1
Cluster3086;	Proteobacteria					0	0	1	0	0	0	0	0	0	0	0	0	1
Cluster3095;	Acidobact	Acidobacteria_Gp2			Gp2	0	0	0	0	0	0	0	0	0	0	0	1	1
Cluster3100;	Bacteroid	Sphing	Sphir	Chitinop	Flavitalea	0	0	1	0	0	0	0	0	0	0	0	0	1
Cluster3101;	Proteobac	Betapr	Burkl	Burkholderiaceae		0	0	1	0	0	0	0	0	0	0	0	0	1
Cluster3104;	Acidobact	Acidobacteria_Gp1				0	0	0	0	1	0	0	0	0	0	0	0	1
Cluster3111;	Proteobac	Alphap	Rhodospirillales			0	0	0	0	0	0	0	0	0	0	1	0	1
Cluster3118;	Acidobact	Acidobacteria_Gp2			Gp2	0	1	0	0	0	0	0	0	0	0	0	0	1
Cluster3127;	Acidobact	Acidobacteria_Gp2			Gp2	0	0	1	0	0	0	0	0	0	0	0	0	1
Cluster3131;size=1						1	0	0	0	0	0	0	0	0	0	0	0	1
Cluster3146;	Proteobac	Gamm	Xantl	Sinobac	Steroidobacter	0	0	0	1	0	0	0	0	0	0	0	0	1
Cluster3147;	Acidobact	Acidobacteria_Gp7			Gp7	0	0	0	0	0	0	0	0	0	0	1	0	1
Cluster3151;	Planctomycetes					0	1	0	0	0	0	0	0	0	0	0	0	1

Cluster3155;	Acidobact	Acidobacteria_Gp1			Gp1	0	0	1	0	0	0	0	0	0	0	0	0	1
Cluster3157;	Acidobact	Acidobacteria_Gp1			Gp1	1	0	0	0	0	0	0	0	0	0	0	0	1
Cluster3158;	Acidobacteria					0	0	0	0	0	1	0	0	0	0	0	0	1
Cluster3167;	Proteobac	Alphap	Rhodospirillales			0	0	0	0	0	0	0	0	0	1	0	0	1
Cluster3168;	Firmicute	Bacilli	Bacill	Pasteur	Pasteuria	0	0	0	0	0	0	1	0	0	0	0	0	1
Cluster3169;size=2						0	0	0	1	0	0	0	0	0	0	0	0	1
Cluster3170;	Proteobac	Alphap	Rhizo	Rhizobia	Rhizobium	0	0	0	0	0	0	0	0	0	1	0	0	1
Cluster3192;size=1						0	0	0	0	0	1	0	0	0	0	0	0	1
Cluster3196;size=6						0	0	1	0	0	0	0	0	0	0	0	0	1
Cluster3197;	Bacteroid	Sphing	Sphir	Sphingo	Nubsella	0	0	0	0	0	1	0	0	0	0	0	0	1
Cluster3200;	Acidobact	Acidobacteria_Gp3			Gp3	0	0	1	0	0	0	0	0	0	0	0	0	1
Cluster3207;size=1						0	0	0	0	0	0	1	0	0	0	0	0	1
Cluster3211;	Proteobac	Alphap	Rhizo	Bradyrh	Bradyrhizobiu	1	0	0	0	0	0	0	0	0	0	0	0	1
Cluster3213;size=2						0	0	0	0	1	0	0	0	0	0	0	0	1
Cluster3216;	Acidobact	Acidobacteria_Gp1				0	0	1	0	0	0	0	0	0	0	0	0	1
Cluster3218;	Proteobac	Alphaproteobacteria				0	0	0	1	0	0	0	0	0	0	0	0	1
Cluster3233;	Proteobac	Alphap	Rhodospirillales			0	0	0	0	0	0	0	0	0	0	1	0	1
Cluster3236;	Proteobac	Alphap	Rhizobiales			0	0	0	1	0	0	0	0	0	0	0	0	1
Cluster3240;	Proteobac	Betapr	Burkholderiales			0	0	0	0	0	0	0	0	0	0	1	0	1
Cluster3242;	Proteobac	Alphap	Rhizo	Bradyrh	Bradyrhizobiu	0	0	0	0	0	0	0	1	0	0	0	0	1
Cluster3247;	Proteobac	Alphap	Rhodospirillales			0	0	0	0	0	0	0	0	0	0	0	1	1
Cluster3252;	Proteobac	Betapr	Burkl	Burkhol	Burkholderia	0	0	0	1	0	0	0	0	0	0	0	0	1
Cluster3254;	Actinobac	Actino	Acidi	Acidimi	Aciditerrimona	1	0	0	0	0	0	0	0	0	0	0	0	1
Cluster3265;	Proteobac	Deltap	Myxo	Cystobacteraceae		0	0	0	0	0	0	0	0	0	1	0	0	1
Cluster3266;	Acidobacteria					0	0	0	0	0	0	0	0	0	1	0	0	1
Cluster3276;	Acidobact	Acidobacteria_Gp1				0	1	0	0	0	0	0	0	0	0	0	0	1
Cluster3284;	Proteobac	Alphap	Rhod	Rhodospirillaceae		1	0	0	0	0	0	0	0	0	0	0	0	1
Cluster3285;	Acidobact	Acidobacteria_Gp1			Gp1	0	0	0	1	0	0	0	0	0	0	0	0	1
Cluster3287;size=3						0	0	0	0	0	0	0	0	0	0	1	0	1

Cluster3288;:	Acidobact	Acidobacteria_Gp2			Gp2	0	0	0	0	0	0	0	0	0	0	1	0	1
Cluster3289;:	Proteobac	Alphap	Rhod	Rhodospirillaceae		0	0	0	0	0	0	0	0	0	0	0	1	1
Cluster3295;:	Verrucom	Opitutae				0	0	0	0	0	0	0	0	0	0	0	1	1
Cluster3300;:	Proteobac	Alphap	Rhizc	Xanthol	Pseudolabrys	1	0	0	0	0	0	0	0	0	0	0	0	1
Cluster3301;:	Proteobac	Alphap	Rhodospirillales			0	1	0	0	0	0	0	0	0	0	0	0	1
Cluster3302;:	Bacteroid	Sphing	Sphir	Chitinop	Flavitalea	0	0	0	0	0	0	0	1	0	0	0	0	1
Cluster3303;:	Proteobac	Alphap	Rhodospirillales			0	0	0	0	0	0	0	1	0	0	0	0	1
Cluster3309;:	Firmicutes	Bacilli	Bacill	Alicyclo	Alicyclobacillu	0	1	0	0	0	0	0	0	0	0	0	0	1
Cluster3319;:	Acidobact	Acidobacteria_Gp1				0	0	0	1	0	0	0	0	0	0	0	0	1
Cluster3320;:	Acidobact	Acidobacteria_Gp1				0	0	0	0	0	0	0	0	1	0	0	0	1
Cluster3321;size=3						0	0	0	0	0	0	0	1	0	0	0	0	1
Cluster3322;:	Armatimonadetes				Armatimonade	0	0	0	0	0	0	0	0	0	0	0	1	1
Cluster3326;:	Acidobact	Acidobacteria_Gp1				0	1	0	0	0	0	0	0	0	0	0	0	1
Cluster3334;:	Verrucom	Opituta	Opitu	Opituta	Opitutus	0	0	0	0	0	1	0	0	0	0	0	0	1
Cluster3335;:	Verrucom	Opituta	Opitu	Opituta	Opitutus	0	0	0	0	1	0	0	0	0	0	0	0	1
Cluster3346;:	Acidobact	Acidobacteria_Gp1			Gp1	0	0	0	1	0	0	0	0	0	0	0	0	1
Cluster3347;:	Acidobact	Acidobacteria_Gp3			Gp3	1	0	0	0	0	0	0	0	0	0	0	0	1
Cluster3355;:	Bacteroid	Sphing	Sphir	Sphingo	Mucilaginibact	0	0	1	0	0	0	0	0	0	0	0	0	1
Cluster3356;size=1						0	1	0	0	0	0	0	0	0	0	0	0	1
Cluster3361;:	Proteobac	Betapr	Burkl	Burkhol	Burkholderia	0	0	0	1	0	0	0	0	0	0	0	0	1
Cluster3364;size=3						0	0	0	1	0	0	0	0	0	0	0	0	1
Cluster3365;:	Acidobact	Acidobacteria_Gp2			Gp2	0	0	0	0	1	0	0	0	0	0	0	0	1
Cluster3373;:	Proteobac	Alphap	Rhod	Rhodos	Dongia	0	0	0	0	1	0	0	0	0	0	0	0	1
Cluster3383;:	Actinobac	Actinol	Actinomycetales			0	0	0	0	0	0	0	1	0	0	0	0	1
Cluster3384;size=8						0	0	0	1	0	0	0	0	0	0	0	0	1
Cluster3386;:	Acidobact	Acidobacteria_Gp13			Gp13	0	0	0	0	0	0	0	0	0	0	0	1	1
Cluster3387;size=1						0	0	0	0	0	0	0	0	1	0	0	0	1
Cluster3392;:	Proteobac	Alphap	Alphaproteob		Rhizomicrobiu	1	0	0	0	0	0	0	0	0	0	0	0	1
Cluster3411;:	Planctomy	Plancto	Planc	Plancto	Singulisphaera	0	0	0	0	0	0	0	1	0	0	0	0	1

Cluster3422;	Proteobac	Alphap	Rhizobiales			0	0	0	0	0	0	1	0	0	0	0	0	1
Cluster3441;	Elusimicro	Elusim	Elusi	Elusimic	Elusimicrobiun	0	0	0	0	0	0	0	0	0	0	0	1	1
Cluster3445;	Verrucom	Spartobacteria			Spartobacteria	0	0	0	0	0	1	0	0	0	0	0	0	1
Cluster3454;	Acidobact	Acidobacteria_Gp1			Gp1	0	0	0	0	0	0	0	1	0	0	0	0	1
Cluster3467;size=14						0	0	0	0	0	0	0	0	1	0	0	0	1
Cluster3469;	Verrucom	Opitut	Opitu	Opituta	Opitutus	0	0	0	0	0	0	0	0	0	1	0	0	1
Cluster3477;	Acidobact	Acidobacteria_Gp2			Gp2	0	0	0	0	0	0	0	0	0	0	0	1	1
Cluster3481;	TM7				TM7_genera_i	0	0	0	0	0	0	0	1	0	0	0	0	1
Cluster3485;	Bacteroid	Sphing	Sphir	Chitinophagaceae		0	0	0	0	0	0	0	0	0	0	0	1	1
Cluster3488;size=9						0	1	0	0	0	0	0	0	0	0	0	0	1
Cluster3489;size=1						0	0	1	0	0	0	0	0	0	0	0	0	1
Cluster3497;	Acidobact	Acidobacteria_Gp2			Gp2	0	0	0	0	0	0	0	0	0	0	0	1	1
Cluster3500;size=1						0	0	0	0	1	0	0	0	0	0	0	0	1
Cluster3503;	Acidobact	Acidobacteria_Gp1			Gp1	0	0	1	0	0	0	0	0	0	0	0	0	1
Cluster3504;size=11						0	0	0	0	0	0	0	0	0	0	0	1	1
Cluster3516;	Acidobact	Acidobacteria_Gp6			Gp6	0	0	0	0	0	0	1	0	0	0	0	0	1
Cluster3517;size=9						0	0	0	0	0	0	1	0	0	0	0	0	1
Cluster3518;	Acidobact	Acidobacteria_Gp1			Gp1	0	1	0	0	0	0	0	0	0	0	0	0	1
Cluster3526;	Acidobact	Acidobacteria_Gp2			Gp2	0	0	0	0	0	0	0	1	0	0	0	0	1
Cluster3527;	Acidobact	Acidobacteria_Gp1			Granulicella	0	0	0	0	0	0	0	0	0	0	1	0	1
Cluster3528;	Proteobac	Gammaproteobacteria				0	0	0	0	0	1	0	0	0	0	0	0	1
Cluster3530;size=8						0	0	0	0	0	0	0	0	1	0	0	0	1
Cluster3533;	Acidobact	Acidobacteria_Gp1				0	0	0	0	0	0	0	0	0	0	1	0	1
Cluster3546;	Acidobact	Acidobacteria_Gp1				0	0	0	0	0	0	0	0	0	1	0	0	1
Cluster3547;	Proteobac	Gamm	Xantl	Xanthor	Rhodanobacte	0	0	0	0	1	0	0	0	0	0	0	0	1
Cluster3556;size=4						0	0	0	0	0	0	0	0	0	0	1	0	1
Cluster3559;size=3						0	0	0	0	0	0	0	0	1	0	0	0	1
Cluster3560;	Acidobact	Acidobacteria_Gp2			Gp2	0	0	0	0	0	0	0	1	0	0	0	0	1
Cluster3564;	Acidobact	Acidobacteria_Gp1			Granulicella	0	0	0	0	0	0	0	0	0	0	0	1	1

Cluster3566;:	Acidobact	Acidobacteria_Gp2			Gp2	0	0	0	1	0	0	0	0	0	0	0	0	1
Cluster3572;:	Proteobac	Betapr	Burkl	Burkhol	Burkholderia	1	0	0	0	0	0	0	0	0	0	0	0	1
Cluster3584;:	Chloroflex	Ktedor	Kted	Ktedon	Ktedonobacter	0	1	0	0	0	0	0	0	0	0	0	0	1
Cluster3587;:	Bacteroid	Sphing	Sphir	Sphingo	Mucilaginibact	0	0	1	0	0	0	0	0	0	0	0	0	1
Cluster3602;:	Proteobac	Alphaproteobacteria				0	0	0	0	0	0	0	1	0	0	0	0	1
Cluster3606;:	Proteobac	Alphap	Rhizo	Bradyrh	Bradyrhizobiur	0	1	0	0	0	0	0	0	0	0	0	0	1
Cluster3611;:	Acidobact	Acidobacteria_Gp1			Edaphobacter	0	0	1	0	0	0	0	0	0	0	0	0	1
Cluster3627;:	Proteobac	Gamm	Legio	Coxiella	Aquicella	0	0	0	0	1	0	0	0	0	0	0	0	1
Cluster3633;:	Planctomycetes					0	0	0	0	0	0	0	1	0	0	0	0	1
Cluster3636;:	Acidobact	Acidobacteria_Gp1				0	0	0	0	0	0	0	1	0	0	0	0	1
Cluster3651;:	Acidobact	Acidobacteria_Gp2			Gp2	0	0	0	1	0	0	0	0	0	0	0	0	1
Cluster3653;:	Proteobac	Alphap	Rhod	Acetoba	Stella	0	0	0	0	0	0	0	0	0	1	0	0	1
Cluster3657;:	Actinobac	Actinol	Solirı	Conexib	Conexibacter	0	0	0	0	0	0	0	0	0	1	0	0	1
Cluster3658;:	Proteobac	Alphap	Rhodospirillales			0	0	0	0	0	0	0	0	0	0	1	0	1
Cluster3660;:	Acidobact	Acidobacteria_Gp2			Gp2	0	0	0	0	1	0	0	0	0	0	0	0	1
Cluster3667;:	Verrucom	Opitutae				0	0	0	0	0	0	0	0	0	1	0	0	1
Cluster3669;:	Acidobact	Acidobacteria_Gp2			Gp2	0	0	0	0	1	0	0	0	0	0	0	0	1
Cluster3677;size=3						0	0	0	0	0	0	0	0	0	0	0	1	1
Cluster3678;size=1						0	0	0	0	0	0	0	0	0	0	0	1	1
Cluster3688;:	Proteobac	Deltap	Myxococcales			0	1	0	0	0	0	0	0	0	0	0	0	1
Cluster3692;:	Proteobac	Alphap	Rhizobiales			0	0	0	0	0	0	1	0	0	0	0	0	1
Cluster3696;size=1						0	0	0	0	0	0	0	0	0	1	0	0	1
Cluster3697;size=1						0	0	0	0	0	0	1	0	0	0	0	0	1
Cluster3698;:	Verrucom	Opituta	Opitu	Opituta	Opitutus	0	0	1	0	0	0	0	0	0	0	0	0	1
Cluster3700;:	Proteobac	Alphap	Rhodospirillales			0	0	0	0	0	0	0	0	1	0	0	0	1
Cluster3702;:	Proteobac	Gammaproteobacteria				0	0	0	0	0	0	0	0	0	0	1	0	1
Cluster3707;:	Proteobac	Alphaproteobacteria				0	0	0	1	0	0	0	0	0	0	0	0	1
Cluster3710;:	Acidobact	Acidobacteria_Gp1			Acidobacteriur	0	0	0	0	0	0	1	0	0	0	0	0	1
Cluster3714;:	Acidobact	Acidobacteria_Gp1			Gp1	0	0	0	0	0	0	1	0	0	0	0	0	1

Cluster3732;	Acidobact	Acidobacteria_Gp2			Gp2	0	0	0	0	0	0	1	0	0	0	0	0	1
Cluster3733;size=3						0	0	0	1	0	0	0	0	0	0	0	0	1
Cluster3739;	Proteobac	Alphap	Rhodospirillales			0	0	0	0	0	0	0	1	0	0	0	0	1
Cluster3740;size=2						0	1	0	0	0	0	0	0	0	0	0	0	1
Cluster3750;	Proteobac	Alphap	Rhodospirillales			0	0	0	0	0	0	0	0	0	0	1	0	1
Cluster3752;	Acidobact	Acidobacteria_Gp2			Gp2	0	0	0	0	0	0	0	0	0	0	0	1	1
Cluster3764;size=2						0	1	0	0	0	0	0	0	0	0	0	0	1
Cluster3770;	Proteobac	Alphap	Rhod	Rhodospirillaceae		0	0	0	0	0	0	0	0	0	0	1	0	1
Cluster3780;	Proteobac	Alphap	Rhodospirillales			0	0	0	0	0	0	0	0	0	0	1	0	1
Cluster3783;	Proteobac	Alphap	Rhizobiales			0	0	0	0	0	0	0	0	0	0	0	1	1
Cluster3799;	Armatimc	Armati	Arma	Armatir	Armatimonas/	0	0	0	0	0	1	0	0	0	0	0	0	1
Cluster3800;	Acidobact	Acidobacteria_Gp2			Gp2	0	0	0	0	0	0	0	0	0	0	1	0	1
Cluster3802;	Proteobac	Alphap	Rhizc	Bradyrh	Bradyrhizobiur	0	0	0	0	0	0	0	0	0	0	1	0	1
Cluster3806;size=9						0	0	0	1	0	0	0	0	0	0	0	0	1
Cluster3807;	Acidobact	Acidobacteria_Gp3				1	0	0	0	0	0	0	0	0	0	0	0	1
Cluster3821;	Proteobac	Alphap	Rhizc	Bradyrh	Bradyrhizobiur	0	0	0	0	0	0	1	0	0	0	0	0	1
Cluster3822;	Acidobact	Acidobacteria_Gp1				0	0	0	0	0	0	0	0	0	0	0	1	1
Cluster3827;	Acidobact	Acidobacteria_Gp1				0	0	0	0	0	0	1	0	0	0	0	0	1
Cluster3830;	Acidobact	Acidobacteria_Gp2			Gp2	0	0	0	0	1	0	0	0	0	0	0	0	1
Cluster3835;	Proteobac	Alphap	Rhodospirillales			0	0	0	0	0	0	0	0	0	0	1	0	1
Cluster3838;size=1						0	0	0	0	0	0	0	0	0	0	0	1	1
Cluster3859;	Proteobac	Alphap	Rhodospirillales			0	1	0	0	0	0	0	0	0	0	0	0	1
Cluster3866;	Proteobac	Alphap	Rhodospirillales			0	0	0	0	0	0	1	0	0	0	0	0	1
Cluster3870;	Planctom	Plancto	Planc	Planctomycetaceae		0	0	0	0	0	1	0	0	0	0	0	0	1
Cluster3878;	Proteobac	Alphap	Rhizc	Xanthol	Pseudolabrys	0	0	0	0	0	0	0	0	1	0	0	0	1
Cluster3882;size=3						0	0	0	1	0	0	0	0	0	0	0	0	1
Cluster3884;size=1						1	0	0	0	0	0	0	0	0	0	0	0	1
Cluster3886;	Actinobac	Actinol	Solirı	Conexib	Conexibacter	0	0	0	0	0	0	0	0	0	0	1	0	1
Cluster3887;size=3						0	0	0	0	0	0	0	0	0	0	1	0	1

Cluster3888;	Acidobact	Acidobacteria_Gp2			Gp2	0	0	0	1	0	0	0	0	0	0	0	0	1
Cluster3892;	Actinobac	Actinol	Solirubrobacterales			0	0	0	0	0	0	0	0	0	0	1	0	1
Cluster3896;	Proteobac	Alphap	Rhodospirillales			0	0	0	0	0	0	0	0	1	0	0	0	1
Cluster3899;size=6						0	0	1	0	0	0	0	0	0	0	0	0	1
Cluster3914;	Acidobact	Acidobacteria_Gp2			Gp2	1	0	0	0	0	0	0	0	0	0	0	0	1
Cluster3915;	Planctomy	Plancto	Planc	Plancto	Singulisphaera	1	0	0	0	0	0	0	0	0	0	0	0	1
Cluster3916;size=1						0	0	0	0	0	0	1	0	0	0	0	0	1
Cluster3917;	Proteobac	Gammaproteobacteria				0	0	0	0	0	0	1	0	0	0	0	0	1
Cluster3921;	Bacteroid	Sphing	Sphir	Sphingo	Mucilaginibact	0	0	0	0	0	0	0	0	0	0	1	0	1
Cluster3923;size=3						1	0	0	0	0	0	0	0	0	0	0	0	1
Cluster3936;size=3						0	0	0	0	0	0	0	0	1	0	0	0	1
Cluster3939;	Proteobac	Alphap	Rhod	Rhodospirillaceae		1	0	0	0	0	0	0	0	0	0	0	0	1
Cluster3941;	Verrucom	Spartobacteria			Spartobacteria	0	0	0	0	0	0	0	0	1	0	0	0	1
Cluster3942;size=1						0	0	0	1	0	0	0	0	0	0	0	0	1
Cluster3943;	Bacteroid	Sphing	Sphingobacteriales			0	0	0	0	0	0	0	0	0	0	0	1	1
Cluster3948;	Planctomy	Plancto	Planc	Planctomycetaceae		0	0	0	1	0	0	0	0	0	0	0	0	1
Cluster3949;size=1						0	0	0	0	0	0	0	0	1	0	0	0	1
Cluster3953;	Acidobact	Acidobacteria_Gp2			Gp2	0	0	0	1	0	0	0	0	0	0	0	0	1
Cluster3959;	Actinobac	Actinol	Acidimicrobiales			0	0	1	0	0	0	0	0	0	0	0	0	1
Cluster3964;	Acidobact	Acidobacteria_Gp1			Gp1	0	0	0	0	0	0	0	0	0	1	0	0	1
Cluster3974;size=4						0	0	0	0	0	1	0	0	0	0	0	0	1
Cluster3975;	Acidobact	Acidobacteria_Gp2			Gp2	0	0	0	1	0	0	0	0	0	0	0	0	1
Cluster3984;size=1						1	0	0	0	0	0	0	0	0	0	0	0	1
Cluster3987;	Actinobac	Actinol	Actin	Actinos	Actinospica	0	0	0	0	0	0	0	0	0	0	0	1	1
Cluster3992;	Proteobac	Gamm	Xantl	Xanthor	Dyella	0	0	1	0	0	0	0	0	0	0	0	0	1
Cluster3997;	Proteobac	Betaproteobacteria				0	0	0	0	0	0	0	0	0	0	1	0	1
Cluster3999;	Proteobac	Alphaproteobacteria				1	0	0	0	0	0	0	0	0	0	0	0	1
Cluster4001;	Acidobact	Acidobacteria_Gp4			Gp4	0	0	0	0	0	0	0	0	0	1	0	0	1
Cluster4003;	Acidobact	Acidobacteria_Gp1				0	0	0	0	1	0	0	0	0	0	0	0	1

Cluster4006;	Proteobac	Gamm	Xantl	Sinobac	Steroidobacter	0	0	0	0	0	1	0	0	0	0	0	0	1
Cluster4011;	Acidobact	Acidobacteria_Gp1				0	0	0	0	0	0	0	0	0	0	0	1	1
Cluster4013;	Acidobact	Acidobacteria_Gp1			Gp1	0	1	0	0	0	0	0	0	0	0	0	0	1
Cluster4021;	Proteobac	Alphap	Rhizobiales			0	1	0	0	0	0	0	0	0	0	0	0	1
Cluster4028;	Proteobac	Alphap	Rhodospirillales			0	0	0	0	0	1	0	0	0	0	0	0	1
Cluster4036;	Proteobacteria					0	0	0	0	1	0	0	0	0	0	0	0	1
Cluster4048;	Armatimonadetes				Armatimonade	0	0	0	0	0	0	1	0	0	0	0	0	1
Cluster4052;size=8						0	0	0	0	0	1	0	0	0	0	0	0	1
Cluster4054;size=1						0	0	0	1	0	0	0	0	0	0	0	0	1
Cluster4071;	Acidobact	Acidobacteria_Gp2			Gp2	0	0	0	0	0	0	1	0	0	0	0	0	1
Cluster4074;size=2						0	0	0	0	0	0	1	0	0	0	0	0	1
Cluster4080;	Bacteroid	Sphing	Sphir	Sphingo	Mucilaginibact	0	0	0	0	0	0	0	0	0	1	0	0	1
Cluster4087;	Bacteroid	Sphing	Sphir	Chitino	Flavitalea	0	0	0	0	1	0	0	0	0	0	0	0	1
Cluster4092;	Proteobac	Alphap	Caulo	Caulob	Phenylobacter	0	0	1	0	0	0	0	0	0	0	0	0	1
Cluster4094;	Acidobact	Acidobacteria_Gp1			Acidobacteriu	0	0	0	0	0	0	1	0	0	0	0	0	1
Cluster4100;	Acidobact	Acidobacteria_Gp3			Gp3	0	0	0	0	1	0	0	0	0	0	0	0	1
Cluster4101;	Acidobact	Acidobacteria_Gp1			Edaphobacter	0	0	0	0	1	0	0	0	0	0	0	0	1
Cluster4104;	Proteobac	Gamm	Xantl	Sinobac	Steroidobacter	0	0	0	0	0	0	1	0	0	0	0	0	1
Cluster4106;	Chlamydia	Chlamy	Chlamydiales			1	0	0	0	0	0	0	0	0	0	0	0	1
Cluster4107;	Proteobac	Alphaproteobacteria				0	0	0	0	0	0	0	0	0	1	0	0	1
Cluster4111;	Acidobact	Acidobacteria_Gp2			Gp2	0	0	0	0	0	0	1	0	0	0	0	0	1
Cluster4114;	Acidobact	Acidobacteria_Gp1			Terriglobus	0	0	0	0	0	1	0	0	0	0	0	0	1
Cluster4122;	Acidobact	Acidobacteria_Gp3				0	0	0	0	0	0	0	0	1	0	0	0	1
Cluster4124;	Planctomycetes					0	0	0	0	1	0	0	0	0	0	0	0	1
Cluster4125;	Proteobacteria					0	0	0	0	0	0	0	0	0	1	0	0	1
Cluster4126;	Proteobac	Alphaproteobacteria				0	0	0	1	0	0	0	0	0	0	0	0	1
Cluster4128;	Acidobact	Acidobacteria_Gp1			Gp1	0	0	0	0	1	0	0	0	0	0	0	0	1
Cluster4129;	Verrucom	Opitutae				1	0	0	0	0	0	0	0	0	0	0	0	1
Cluster4131;	Proteobac	Gammaproteobacteria				0	0	0	0	0	0	0	0	1	0	0	0	1

Cluster4137;	Acidobact	Acidobacteria_Gp2			Gp2	0	1	0	0	0	0	0	0	0	0	0	0	1
Cluster4138;	Acidobact	Acidobacteria_Gp1				0	0	1	0	0	0	0	0	0	0	0	0	1
Cluster4143;size=4						0	1	0	0	0	0	0	0	0	0	0	0	1
Cluster4144;size=1						0	0	0	0	0	0	0	0	0	0	1	0	1
Cluster4149;size=5						0	0	0	0	0	0	0	0	0	0	1	0	1
Cluster4151;	Verrucom	Spartobacteria			Spartobacteria	0	0	0	0	0	0	0	0	1	0	0	0	1
Cluster4157;	Acidobact	Acidobacteria_Gp2			Gp2	0	0	0	0	0	0	1	0	0	0	0	0	1
Cluster4158;	Planctomy	Planctc	Planc	Plancto	Singulisphaera	0	0	1	0	0	0	0	0	0	0	0	0	1
Cluster4161;	Acidobact	Acidobacteria_Gp10			Gp10	0	0	0	1	0	0	0	0	0	0	0	0	1
Cluster4162;	Verrucom	Opitut	Opitu	Opituta	Opitutus	0	0	0	0	0	0	0	0	0	0	0	1	1
Cluster4163;size=1						0	1	0	0	0	0	0	0	0	0	0	0	1
Cluster4166;	Acidobact	Acidobacteria_Gp2			Gp2	0	0	0	0	0	0	0	0	1	0	0	0	1
Cluster4171;	Proteobac	Alphaproteobacteria				0	0	0	0	0	0	0	0	1	0	0	0	1
Cluster4173;size=2						0	0	0	0	0	0	1	0	0	0	0	0	1
Cluster4175;	Verrucom	Spartobacteria			Spartobacteria	0	0	0	0	0	0	1	0	0	0	0	0	1
Cluster4181;	Planctomy	Planctc	Planc	Planctomycetaceae		0	0	0	0	0	0	1	0	0	0	0	0	1
Cluster4183;	Proteobac	Alphap	Rhod	Rhodos	Dongia	0	0	0	0	0	0	0	0	0	1	0	0	1
Cluster4187;size=5						0	0	0	0	1	0	0	0	0	0	0	0	1
Cluster4193;	Acidobact	Acidobacteria_Gp1			Acidobacteriur	0	0	1	0	0	0	0	0	0	0	0	0	1
Cluster4196;	Proteobac	Deltap	Myxc	Polyangiaceae		0	0	0	0	0	0	1	0	0	0	0	0	1
Cluster4200;	Verrucom	Subdivision3			Subdivision3_g	0	0	0	0	0	0	0	0	0	0	1	0	1
Cluster4206;	Proteobacteria					0	1	0	0	0	0	0	0	0	0	0	0	1
Cluster4207;	Proteobac	Alphap	Rhizobiales			0	0	0	1	0	0	0	0	0	0	0	0	1
Cluster4211;	Actinobac	Actinol	Actin	Actinos	Actinospica	0	0	0	0	0	0	1	0	0	0	0	0	1
Cluster4212;	Planctomycetes					0	0	0	0	0	0	0	1	0	0	0	0	1
Cluster4216;	Proteobac	Alphap	Rhodospirillales			0	0	0	1	0	0	0	0	0	0	0	0	1
Cluster4229;	Planctomycetes					0	0	0	0	0	0	1	0	0	0	0	0	1
Cluster4234;size=1						0	0	0	0	1	0	0	0	0	0	0	0	1
Cluster4235;	Acidobact	Acidobacteria_Gp1				0	0	1	0	0	0	0	0	0	0	0	0	1

Cluster4236;size=1						0	0	0	0	0	0	1	0	0	0	0	0	1
Cluster4248;:	Proteobac	Betaproteobacteria				0	0	1	0	0	0	0	0	0	0	0	0	1
Cluster4250;:	Armatimc	Armati	Arma	Armatir	Armatimonas/.	0	0	1	0	0	0	0	0	0	0	0	0	1
Cluster4251;:	Acidobact	Acidobacteria_Gp2			Gp2	0	0	0	0	0	0	0	0	1	0	0	0	1
Cluster4254;:	Proteobac	Alphap	Rhod	Acetobacteraceae		0	0	0	0	0	0	0	0	0	1	0	0	1
Cluster4255;:	Proteobac	Alphap	Alphaproteob		Rhizomicrobiu	0	0	0	0	0	0	0	0	1	0	0	0	1
Cluster4259;:	Acidobact	Acidobacteria_Gp1				0	0	0	0	0	0	0	0	0	0	1	0	1
Cluster4265;:	Proteobac	Alphap	Rhodospirillales			0	0	0	0	1	0	0	0	0	0	0	0	1
Cluster4269;:	Acidobact	Acidobacteria_Gp1			Gp1	0	0	0	0	0	0	1	0	0	0	0	0	1
Cluster4276;:	Acidobact	Acidobacteria_Gp3				0	0	1	0	0	0	0	0	0	0	0	0	1
Cluster4281;:	Proteobac	Alphap	Rhodospirillales			0	0	0	0	0	0	0	0	1	0	0	0	1
Cluster4284;:	Proteobac	Betaproteobacteria				0	0	0	0	0	0	0	0	0	0	0	1	1
Cluster4285;size=6						0	0	0	0	1	0	0	0	0	0	0	0	1
Cluster4287;:	Proteobac	Alphap	Rhodospirillales			0	0	0	0	0	0	0	0	1	0	0	0	1
Cluster4290;:	Proteobac	Alphap	Rhizobiales			0	0	1	0	0	0	0	0	0	0	0	0	1
Cluster4295;:	Acidobact	Acidobacteria_Gp1			Granulicella	0	0	0	0	0	0	0	0	0	0	1	0	1
Cluster4297;:	Bacteroid	Sphing	Sphir	Chitinophagaceae		0	0	0	0	0	1	0	0	0	0	0	0	1
Cluster4300;:	Acidobact	Acidobacteria_Gp1			Gp1	0	0	0	0	0	0	1	0	0	0	0	0	1
Cluster4303;:	Acidobact	Acidobacteria_Gp10			Gp10	0	0	0	0	0	0	0	1	0	0	0	0	1
Cluster4310;:	Verrucom	Subdivision3			Subdivision3_g	0	0	1	0	0	0	0	0	0	0	0	0	1
Cluster4311;:	Actinobac	Actinol	Acidi	Acidimi	Aciditerrimona	0	0	0	0	0	0	0	0	0	0	0	1	1
Cluster4312;:	Acidobact	Acidobacteria_Gp1				0	0	0	0	0	1	0	0	0	0	0	0	1
Cluster4315;:	Proteobac	Deltaproteobacteria				0	0	0	0	0	0	0	0	0	0	0	1	1
Cluster4324;:	Planctomycetes					0	0	1	0	0	0	0	0	0	0	0	0	1
Cluster4325;:	Acidobact	Acidobacteria_Gp2			Gp2	0	0	0	0	0	1	0	0	0	0	0	0	1
Cluster4332;:	Acidobact	Acidobacteria_Gp2			Gp2	0	1	0	0	0	0	0	0	0	0	0	0	1
Cluster4333;:	Verrucom	Subdivision3			Subdivision3_g	0	0	0	0	0	0	0	0	0	0	0	1	1
Cluster4335;:	Acidobact	Acidobacteria_Gp3				0	0	0	0	0	1	0	0	0	0	0	0	1
Cluster4348;size=4						0	0	0	0	0	0	0	0	1	0	0	0	1

Cluster4355;	Planctomycetes					0	0	0	1	0	0	0	0	0	0	0	0	1
Cluster4360;	Acidobact	Acidobacteria_Gp1				0	0	0	0	0	0	0	0	0	1	0	0	1
Cluster4380;size=2						0	0	0	0	0	0	1	0	0	0	0	0	1
Cluster4381;	Proteobac	Deltaproteobacteria				0	1	0	0	0	0	0	0	0	0	0	0	1
Cluster4382;	Proteobac	Gamm	Xantl	Sinobac	Steroidobacter	0	0	1	0	0	0	0	0	0	0	0	0	1
Cluster4385;	Proteobac	Alphap	Rhodospirillales			0	0	0	0	0	0	0	0	1	0	0	0	1
Cluster4398;size=4						0	1	0	0	0	0	0	0	0	0	0	0	1
Cluster4400;	Proteobac	Gammaproteobacteria				0	0	0	0	0	0	0	0	1	0	0	0	1
Cluster4415;	Planctomy	Plancto	Planc	Planctomycetaceae		0	0	0	0	0	0	0	0	0	0	1	0	1
Cluster4433;	Acidobacteria					0	0	1	0	0	0	0	0	0	0	0	0	1
Cluster4444;	Verrucom	Spartobacteria			Spartobacteria	0	0	0	0	0	0	1	0	0	0	0	0	1
Cluster4448;	Proteobac	Alphap	Rhodospirillales			0	0	0	0	0	0	0	0	1	0	0	0	1
Cluster4457;	Proteobac	Alphap	Rhodospirillales			0	0	1	0	0	0	0	0	0	0	0	0	1
Cluster4461;	Proteobac	Alphaproteobacteria				0	0	0	0	0	1	0	0	0	0	0	0	1
Cluster4470;	Proteobac	Gamm	Legio	Coxiella	Aquicella	0	0	0	0	0	0	1	0	0	0	0	0	1
Cluster4485;	Proteobac	Gammaproteobacteria				1	0	0	0	0	0	0	0	0	0	0	0	1
Cluster4490;	Verrucom	Subdivision3			Subdivision3_g	0	0	0	0	0	0	0	0	0	0	1	0	1
Cluster4492;	Proteobac	Betapr	Burkl	Burkhol	Burkholderia	1	0	0	0	0	0	0	0	0	0	0	0	1
Cluster4493;size=1						0	0	0	1	0	0	0	0	0	0	0	0	1
Cluster4512;	Acidobact	Acidobacteria_Gp1			Edaphobacter	0	0	0	0	0	0	0	0	0	1	0	0	1
Cluster4518;	Proteobac	Alphap	Rhodospirillales			0	1	0	0	0	0	0	0	0	0	0	0	1
Cluster4530;	Armatimo	Armati	Arma	Armatin	Armatimonas/	0	0	0	0	0	0	1	0	0	0	0	0	1
Cluster4533;size=11						0	0	0	0	1	0	0	0	0	0	0	0	1
Cluster4543;	Planctomycetes					0	0	0	0	0	0	0	0	0	0	1	0	1
Cluster4552;	Acidobact	Acidobacteria_Gp3				0	0	0	0	0	0	0	0	0	1	0	0	1
Cluster4557;	Acidobact	Acidobacteria_Gp2			Gp2	0	0	0	0	0	0	0	0	0	1	0	0	1
Cluster4559;	Proteobac	Deltap	Myxococcales			0	0	0	1	0	0	0	0	0	0	0	0	1
Cluster4563;size=1						0	0	0	0	0	0	0	0	0	0	0	1	1
Cluster4573;	Acidobacteria					0	0	0	0	0	1	0	0	0	0	0	0	1

Cluster4576;size=6						0	0	0	1	0	0	0	0	0	0	0	0	1
Cluster4579;size=1						0	0	0	0	0	0	0	0	0	1	0	0	1
Cluster4584;size=1						0	0	0	0	1	0	0	0	0	0	0	0	1
Cluster4585;	Verrucom	Subdivision3			Subdivision3_g	0	0	0	0	0	0	0	0	1	0	0	0	1
Cluster4586;	Proteobac	Betapr	Burkl	Burkholderiaceae		0	0	0	0	0	0	0	0	0	0	1	0	1
Cluster4596;	Acidobact	Acidobacteria_Gp2			Gp2	0	0	0	0	1	0	0	0	0	0	0	0	1
Cluster4611;	Verrucom	Opitut	Opitu	Opituta	Opitutus	0	0	0	0	0	0	0	0	0	0	1	0	1
Cluster4618;	Verrucom	Opitut	Opitu	Opituta	Opitutus	0	0	0	0	0	0	0	0	0	0	0	1	1
Cluster4622;	Proteobac	Alphaproteobacteria				0	0	0	0	0	0	0	0	0	0	0	1	1
Cluster4633;	Proteobac	Alphap	Rhizobiales			0	0	0	0	1	0	0	0	0	0	0	0	1
Cluster4636;size=1						0	0	0	0	0	0	0	0	0	0	1	0	1
Cluster4645;	Proteobac	Alphap	Rhizo	Xanthol	Labrys	1	0	0	0	0	0	0	0	0	0	0	0	1
Cluster4652;	Proteobac	Alphap	Rhizo	Xanthol	Pseudolabrys	0	0	0	1	0	0	0	0	0	0	0	0	1
Cluster4662;	Elusimicro	Elusim	Elusi	Elusimic	Elusimicrobiun	0	0	0	0	0	0	0	0	0	0	1	0	1
Cluster4665;	Acidobact	Acidobacteria_Gp3			Gp3	0	0	0	0	0	0	0	1	0	0	0	0	1
Cluster4667;	Proteobac	Alphap	Rhizo	Bradyrhizobiaceae		0	0	0	1	0	0	0	0	0	0	0	0	1
Cluster4669;size=1						0	0	0	0	0	0	0	0	0	0	1	0	1
Cluster4674;size=3						0	0	0	0	0	0	0	0	0	0	1	0	1
Cluster4677;	Proteobac	Deltaproteobacteria				0	0	0	1	0	0	0	0	0	0	0	0	1
Cluster4682;size=2						0	0	0	0	0	0	1	0	0	0	0	0	1
Cluster4684;	Proteobac	Deltap	Myxococcales			1	0	0	0	0	0	0	0	0	0	0	0	1
Cluster4687;size=6						0	0	0	0	0	1	0	0	0	0	0	0	1
Cluster4690;	Acidobact	Acidobacteria_Gp3			Gp3	0	0	0	0	0	0	0	0	0	0	1	0	1
Cluster4691;size=1						0	0	0	0	0	0	0	0	0	0	0	1	1
Cluster4700;	Proteobac	Betapr	Burkholderiales			0	0	0	0	0	0	0	1	0	0	0	0	1
Cluster4709;	Proteobac	Deltap	Myxo	Polyangiaceae		0	0	0	0	0	0	0	1	0	0	0	0	1
Cluster4714;	Acidobact	Acidobacteria_Gp1			Gp1	0	1	0	0	0	0	0	0	0	0	0	0	1
Cluster4715;	Acidobact	Acidobacteria_Gp2			Gp2	0	0	0	0	0	1	0	0	0	0	0	0	1
Cluster4722;	Acidobact	Acidobacteria_Gp10			Gp10	0	0	0	0	0	0	0	0	1	0	0	0	1

Cluster4729;size=1						0	0	0	1	0	0	0	0	0	0	0	0	1
Cluster4733;	Acidobact	Acidobacteria_Gp1				0	0	0	1	0	0	0	0	0	0	0	0	1
Cluster4742;	Proteobac	Alphap	Rhodospirillales			0	0	0	0	0	0	0	0	0	0	1	0	1
Cluster4746;	Acidobact	Acidobacteria_Gp3			Gp3	0	0	0	0	0	0	0	0	0	0	0	1	1
Cluster4748;	Firmicute:	Bacilli	Bacill	Paeniba	Paenibacillus	0	0	0	0	0	0	0	0	0	0	0	1	1
Cluster4750;	Proteobac	Deltaproteobacteria				0	0	0	0	0	0	0	1	0	0	0	0	1
Cluster4751;	Armatimonadetes				Armatimonade	0	1	0	0	0	0	0	0	0	0	0	0	1
Cluster4752;	Acidobact	Acidobacteria_Gp2			Gp2	0	0	0	0	1	0	0	0	0	0	0	0	1
Cluster4753;	Proteobacteria					0	0	0	0	0	0	1	0	0	0	0	0	1
Cluster4754;size=1						1	0	0	0	0	0	0	0	0	0	0	0	1
Cluster4762;	Proteobac	Alphap	Rhod	Acetobacteraceae		0	0	0	0	0	0	0	0	0	0	1	0	1
Cluster4766;	Proteobac	Alphap	Rhizc	Xanthob	Pseudolabrys	0	0	0	1	0	0	0	0	0	0	0	0	1
Cluster4775;	Proteobac	Alphap	Rhizc	Xanthob	Labrys	0	0	0	0	1	0	0	0	0	0	0	0	1
Cluster4777;	Proteobac	Alphap	Rhodospirillales			0	0	0	0	0	0	0	1	0	0	0	0	1
Cluster4782;	Actinobac	Actinol	Actinomycetales			0	0	0	0	0	1	0	0	0	0	0	0	1
Cluster4786;	Planctomy	Plancto	Planc	Planctomycetaceae		0	0	0	0	1	0	0	0	0	0	0	0	1
Cluster4791;	Planctomy	Plancto	Planc	Plancto	Singulisphaera	0	0	0	0	0	0	0	0	0	0	0	1	1
Cluster4792;	Proteobac	Alphap	Rhizobiales			0	0	0	1	0	0	0	0	0	0	0	0	1
Cluster4793;	Proteobac	Alphap	Rhodospirillales			0	0	0	0	0	0	0	0	0	0	0	1	1
Cluster4798;	Elusimicro	Elusim	Elusi	Elusimic	Elusimicrobiun	1	0	0	0	0	0	0	0	0	0	0	0	1
Cluster4804;	Acidobact	Acidobacteria_Gp1				0	0	1	0	0	0	0	0	0	0	0	0	1
Cluster4811;	Acidobact	Acidobacteria_Gp2			Gp2	0	0	0	0	1	0	0	0	0	0	0	0	1
Cluster4812;	Acidobact	Acidobacteria_Gp1			Gp1	1	0	0	0	0	0	0	0	0	0	0	0	1
Cluster4817;	Proteobac	Alphap	Rhodospirillales			0	0	0	0	0	0	0	0	1	0	0	0	1
Cluster4834;size=5						0	0	0	0	0	0	0	1	0	0	0	0	1
Cluster4836;	Verrucomicrobia					0	0	0	0	0	0	0	0	0	1	0	0	1
Cluster4846;size=12						0	0	0	0	0	0	1	0	0	0	0	0	1
Cluster4851;	Actinobac	Actinol	Solirubrobacterales			0	0	0	0	0	0	0	0	0	0	0	1	1
Cluster4855;	Proteobac	Betaproteobacteria				0	0	0	1	0	0	0	0	0	0	0	0	1

Cluster4862;:	Planctomy	Plancto	Planc	Planctomycetaceae		0	0	0	0	0	0	1	0	0	0	0	0	1
Cluster4874;:	Acidobact	Acidobacteria_Gp1				0	0	0	0	0	0	0	0	1	0	0	0	1
Cluster4875;size=1						0	0	0	1	0	0	0	0	0	0	0	0	1
Cluster4879;:	Proteobac	Alphap	Rhizc	Bradyrh	Agromonas	0	0	0	0	0	0	0	1	0	0	0	0	1
Cluster4880;:	Planctomycetes					0	1	0	0	0	0	0	0	0	0	0	0	1
Cluster4887;:	Acidobact	Acidobacteria_Gp2			Gp2	0	0	0	1	0	0	0	0	0	0	0	0	1
Cluster4889;:	Verrucom	Spartobacteria				0	0	0	1	0	0	0	0	0	0	0	0	1
Cluster4907;:	Acidobact	Acidobacteria_Gp3			Gp3	0	0	1	0	0	0	0	0	0	0	0	0	1
Cluster4910;:	Proteobac	Deltaproteobacteria				0	0	0	0	0	0	1	0	0	0	0	0	1
Cluster4913;:	Proteobac	Alphap	Rhizc	Xanthol	Pseudolabrys	0	0	0	0	0	0	0	0	0	1	0	0	1
Cluster4926;:	Verrucom	Subdivision3			Subdivision3_g	0	0	0	0	0	0	0	0	0	0	1	0	1
Cluster4930;:	Proteobac	Alphap	Rhizobiales			0	0	0	1	0	0	0	0	0	0	0	0	1
Cluster4943;size=1						0	1	0	0	0	0	0	0	0	0	0	0	1
Cluster4944;:	Acidobact	Acidobacteria_Gp2			Gp2	0	0	0	0	0	0	0	0	0	0	0	1	1
Cluster4957;:	Acidobact	Acidobacteria_Gp2			Gp2	0	1	0	0	0	0	0	0	0	0	0	0	1
Cluster4962;size=7						0	0	0	0	1	0	0	0	0	0	0	0	1
Cluster4963;:	Proteobac	Gammaproteobacteria				0	0	0	0	0	0	0	0	0	0	1	0	1
Cluster4964;:	Acidobact	Acidobacteria_Gp2			Gp2	0	0	0	0	0	0	0	0	1	0	0	0	1
Cluster4966;:	Verrucom	Subdivision3			Subdivision3_g	0	0	0	0	0	1	0	0	0	0	0	0	1
Cluster4967;:	Proteobac	Alphap	Caulc	Cauloba	Caulobacter	0	0	0	0	0	1	0	0	0	0	0	0	1
Cluster4977;:	Proteobac	Alphap	Rhodospirillales			0	0	0	0	0	0	1	0	0	0	0	0	1
Cluster4978;:	Planctomycetes					0	0	0	0	0	0	0	0	0	1	0	0	1
Cluster4983;:	Proteobac	Alphap	Rhizobiales			0	0	0	0	0	0	0	0	0	0	0	1	1
Cluster4986;:	Proteobac	Gamm	Legio	Coxiella	Aquicella	0	0	0	1	0	0	0	0	0	0	0	0	1
Cluster4989;:	Actinobac	Actinol	Solirubrobacterales			0	0	1	0	0	0	0	0	0	0	0	0	1
Cluster4994;:	Acidobact	Acidobacteria_Gp2			Gp2	0	1	0	0	0	0	0	0	0	0	0	0	1
Cluster4995;:	Acidobact	Acidobacteria_Gp1			Gp1	0	0	0	0	0	0	0	0	0	0	1	0	1
Cluster5002;:	Proteobac	Alphap	Rhizobiales			0	0	1	0	0	0	0	0	0	0	0	0	1
Cluster5005;:	Actinobac	Actinol	Actinomycetales			0	0	0	0	0	0	0	0	1	0	0	0	1

Cluster5007;	Proteobac	Alphap	Rhodospirillales			0	0	0	0	0	0	0	0	0	0	1	0	1
Cluster5008;	Acidobact	Acidobacteria_Gp1				0	0	0	0	1	0	0	0	0	0	0	0	1
Cluster5009;	Acidobact	Acidobacteria_Gp1			Acidobacteriur	0	0	0	0	1	0	0	0	0	0	0	0	1
Cluster5012;size=6						0	1	0	0	0	0	0	0	0	0	0	0	1
Cluster5023;	Acidobact	Acidobacteria_Gp1				0	0	0	1	0	0	0	0	0	0	0	0	1
Cluster5025;	Proteobac	Alphap	Rhizobiales			0	0	0	0	0	1	0	0	0	0	0	0	1
Cluster5027;	Acidobact	Acidobacteria_Gp2			Gp2	0	0	0	0	0	0	0	1	0	0	0	0	1
Cluster5028;	Proteobac	Alphap	Rhod	Rhodospirillaceae		0	0	0	0	0	1	0	0	0	0	0	0	1
Cluster5029;	Proteobac	Alphap	Rhodospirillales			0	0	0	0	1	0	0	0	0	0	0	0	1
Cluster5031;	Chlamydia	Chlamy	Chlamydiales			0	0	0	0	0	0	0	0	1	0	0	0	1
Cluster5034;size=2						1	0	0	0	0	0	0	0	0	0	0	0	1
Cluster5044;	Proteobac	Betapr	Burkl	Burkhol	Burkholderia	0	0	0	0	0	0	0	1	0	0	0	0	1
Cluster5047;	Proteobac	Alphap	Rhodospirillales			1	0	0	0	0	0	0	0	0	0	0	0	1
Cluster5051;	Acidobact	Acidobacteria_Gp1				0	0	0	0	1	0	0	0	0	0	0	0	1
Cluster5054;size=4						0	0	0	0	0	0	0	0	0	0	1	0	1
Cluster5055;	Actinobac	Actinol	Actinomycetales			0	0	0	1	0	0	0	0	0	0	0	0	1
Cluster5063;size=2						0	0	0	0	0	0	0	1	0	0	0	0	1
Cluster5065;	Armatimonadetes				Armatimonade	0	0	1	0	0	0	0	0	0	0	0	0	1
Cluster5071;size=8						0	0	0	1	0	0	0	0	0	0	0	0	1
Cluster5073;	Proteobac	Alphap	Rhodospirillales			0	1	0	0	0	0	0	0	0	0	0	0	1
Cluster5076;	Acidobact	Acidobacteria_Gp1				1	0	0	0	0	0	0	0	0	0	0	0	1
Cluster5077;	Verrucom	Subdivision3			Subdivision3_g	0	1	0	0	0	0	0	0	0	0	0	0	1
Cluster5079;	Verrucom	Opitut	Opitu	Opituta	Opitutus	0	0	0	0	0	1	0	0	0	0	0	0	1
Cluster5083;size=1						0	0	0	0	0	1	0	0	0	0	0	0	1
Cluster5091;	Proteobac	Alphap	Caulc	Caulobacteraceae		0	0	0	1	0	0	0	0	0	0	0	0	1
Cluster5096;	Acidobact	Acidobacteria_Gp1			Gp1	0	0	0	0	0	0	0	0	0	0	1	0	1
Cluster5103;	TM7				TM7_genera_i	0	0	0	0	0	0	0	1	0	0	0	0	1
Cluster5108;	Acidobact	Acidobacteria_Gp2			Gp2	0	0	0	0	0	1	0	0	0	0	0	0	1
Cluster5111;	Acidobact	Acidobacteria_Gp2			Gp2	1	0	0	0	0	0	0	0	0	0	0	0	1

Cluster5112;	Proteobac	Deltap	Myxococcales			0	0	0	0	0	0	0	0	0	0	1	0	1
Cluster5113;	Planctomycetes					0	0	0	1	0	0	0	0	0	0	0	0	1
Cluster5115;	Proteobac	Alphap	Rhizo	Bradyrhizobiaceae		0	0	0	0	0	0	1	0	0	0	0	0	1
Cluster5127;	Planctomycetes					0	0	0	0	0	0	0	1	0	0	0	0	1
Cluster5135;	Proteobac	Alphap	Rhizo	Bradyrh	Bradyrhizobiur	0	0	0	0	0	0	0	0	0	1	0	0	1
Cluster5138;	Proteobac	Alphap	Rhod	Rhodospirillaceae		0	0	0	1	0	0	0	0	0	0	0	0	1
Cluster5143;size=2						0	0	0	0	0	0	0	0	1	0	0	0	1
Cluster5151;	Planctomy	Plancto	Planc	Plancto	Singulisphaera	0	0	0	0	0	0	0	1	0	0	0	0	1
Cluster5154;	Planctomy	Plancto	Planc	Plancto	Singulisphaera	0	0	0	0	0	0	1	0	0	0	0	0	1
Cluster5167;	Acidobact	Acidobacteria_Gp1			Gp1	0	0	0	0	0	0	0	0	0	0	0	1	1
Cluster5169;	Acidobact	Acidobacteria_Gp2			Gp2	0	0	0	0	0	0	1	0	0	0	0	0	1
Cluster5174;	Actinobac	Actinol	Actin	Pseudonocardiaceae		0	0	0	1	0	0	0	0	0	0	0	0	1
Cluster5181;	Proteobac	Betapr	Burkholderiales			0	0	0	0	0	0	0	0	0	0	0	1	1
Cluster5187;	Proteobacteria					0	0	1	0	0	0	0	0	0	0	0	0	1
Cluster5189;	Proteobac	Alphap	Rhodospirillales			0	0	0	0	0	0	0	0	0	0	1	0	1
Cluster5191;	Acidobact	Acidobacteria_Gp1				0	0	0	1	0	0	0	0	0	0	0	0	1
Cluster5213;	Proteobacteria					0	0	0	0	0	0	0	1	0	0	0	0	1
Cluster5215;	Acidobact	Acidobacteria_Gp1			Terriglobus	0	0	0	0	0	0	0	0	0	0	1	0	1
Cluster5216;	Acidobact	Acidobacteria_Gp2			Gp2	0	0	0	0	0	0	1	0	0	0	0	0	1
Cluster5240;	Proteobac	Alphaproteobacteria				0	0	0	0	0	0	0	0	0	0	0	1	1
Cluster5243;size=1						0	0	1	0	0	0	0	0	0	0	0	0	1
Cluster5247;	Acidobact	Acidobacteria_Gp1			Gp1	0	0	0	0	0	0	0	0	0	0	0	1	1
Cluster5249;	Acidobact	Acidobacteria_Gp2			Gp2	0	0	0	0	0	0	0	0	0	0	0	1	1
Cluster5251;	Acidobact	Acidobacteria_Gp2			Gp2	1	0	0	0	0	0	0	0	0	0	0	0	1
Cluster5252;	Acidobact	Acidobacteria_Gp1			Terriglobus	0	0	1	0	0	0	0	0	0	0	0	0	1
Cluster5254;size=4						0	0	0	0	0	0	0	0	0	1	0	0	1
Cluster5260;size=7						0	0	0	0	1	0	0	0	0	0	0	0	1
Cluster5261;	Acidobacteria					0	0	0	0	0	0	0	0	0	0	0	1	1
Cluster5266;size=2						1	0	0	0	0	0	0	0	0	0	0	0	1

Cluster5278;size=1						0	0	0	0	0	0	0	0	1	0	0	0	1
Cluster5280;size=1						0	0	0	0	1	0	0	0	0	0	0	0	1
Cluster5297;	Acidobact	Acidobacteria_Gp1				0	0	0	0	0	0	0	0	0	0	1	0	1
Cluster5299;	Proteobac	Deltap	Myxc	Cystobacteraceae		0	0	0	0	1	0	0	0	0	0	0	0	1
Cluster5302;size=15						0	0	0	0	0	1	0	0	0	0	0	0	1
Cluster5308;	Acidobact	Acidobacteria_Gp2			Gp2	0	0	0	0	0	1	0	0	0	0	0	0	1
Cluster5316;	Actinobac	Actinol	Actin	Mycoba	Mycobacteriur	0	0	0	0	0	1	0	0	0	0	0	0	1
Cluster5321;	Actinobac	Actinol	Actinomycetales			0	0	1	0	0	0	0	0	0	0	0	0	1
Cluster5326;size=2						0	0	0	0	0	1	0	0	0	0	0	0	1
Cluster5328;	Acidobact	Acidobacteria_Gp3			Gp3	0	0	0	0	0	0	1	0	0	0	0	0	1
Cluster5338;	Planctomy	Plancto	Planc	Planctor	Singulisphaera	0	0	0	0	0	0	0	0	0	0	1	0	1
Cluster5347;	Proteobacteria					0	0	0	0	0	0	0	0	0	0	1	0	1
Cluster5352;	Acidobact	Acidobacteria_Gp1			Gp1	1	0	0	0	0	0	0	0	0	0	0	0	1
Cluster5355;	Proteobac	Gamm	Xanthomonadales			0	0	0	0	0	0	0	0	0	0	0	1	1
Cluster5364;	Proteobac	Deltaproteobacteria				0	1	0	0	0	0	0	0	0	0	0	0	1
Cluster5368;	Proteobac	Alphaproteobacteria				0	0	1	0	0	0	0	0	0	0	0	0	1
Cluster5369;	Proteobac	Alphap	Rhodospirillales			0	0	1	0	0	0	0	0	0	0	0	0	1
Cluster5372;size=2						0	0	0	0	0	1	0	0	0	0	0	0	1
Cluster5373;	Acidobact	Acidobacteria_Gp3			Gp3	0	0	0	1	0	0	0	0	0	0	0	0	1
Cluster5377;	Acidobact	Acidobacteria_Gp2			Gp2	0	0	0	1	0	0	0	0	0	0	0	0	1
Cluster5378;size=5						0	0	1	0	0	0	0	0	0	0	0	0	1
Cluster5381;	Acidobact	Acidobacteria_Gp2			Gp2	0	0	0	0	0	0	0	0	0	0	1	0	1
Cluster5384;	Verrucom	Subdivision3			Subdivision3_g	0	0	0	0	0	0	0	0	0	1	0	0	1
Cluster5388;	Acidobact	Acidobacteria_Gp1				0	0	0	1	0	0	0	0	0	0	0	0	1
Cluster5392;	Acidobact	Acidobacteria_Gp10			Gp10	0	0	0	0	0	0	0	0	1	0	0	0	1
Cluster5398;	Proteobac	Alphap	Rhizc	Hyphon	Rhodoplanes	0	0	0	0	0	0	1	0	0	0	0	0	1
Cluster5400;size=4						0	0	0	0	0	0	0	1	0	0	0	0	1
Cluster5411;	Proteobac	Alphap	Rhizc	Bradyrh	Bradyrhizobiur	0	1	0	0	0	0	0	0	0	0	0	0	1
Cluster5413;	Proteobac	Alphap	Rhodospirillales			0	0	0	0	0	0	0	0	0	0	1	0	1

Cluster5414;size=3						0	0	0	0	0	0	0	0	0	0	0	1	1
Cluster5416;:	Actinobac	Actinol	Actinomycetales			0	0	0	0	0	0	0	0	0	0	1	0	1
Cluster5420;:	Proteobac	Alphaproteobacteria				0	0	0	1	0	0	0	0	0	0	0	0	1
Cluster5432;size=1						0	0	0	0	0	1	0	0	0	0	0	0	1
Cluster5434;:	Acidobact	Acidobacteria_Gp2			Gp2	0	0	0	0	0	0	0	0	0	0	1	0	1
Cluster5435;size=7						0	0	0	0	0	1	0	0	0	0	0	0	1
Cluster5438;:	Planctomy	Plancto	Planc	Planctomycetaceae		0	0	0	0	0	0	1	0	0	0	0	0	1
Cluster5444;:	Acidobacteria					0	0	0	0	0	0	0	1	0	0	0	0	1
Cluster5447;size=4						1	0	0	0	0	0	0	0	0	0	0	0	1
Cluster5452;:	Acidobact	Acidobacteria_Gp6			Gp6	0	0	0	0	0	0	0	0	0	0	0	1	1
Cluster5453;:	Proteobac	Alphaproteobacteria				0	0	0	1	0	0	0	0	0	0	0	0	1
Cluster5462;:	Proteobac	Deltaproteobacteria				0	0	0	0	0	1	0	0	0	0	0	0	1
Cluster5476;:	Proteobac	Gamm	Xantl	Sinobac	Steroidobacter	0	0	0	0	0	0	0	0	0	1	0	0	1
Cluster5484;:	Proteobacteria					0	0	0	0	0	0	1	0	0	0	0	0	1
Cluster5488;size=2						0	0	0	0	0	0	0	0	1	0	0	0	1
Cluster5490;size=3						0	1	0	0	0	0	0	0	0	0	0	0	1
Cluster5492;:	Acidobact	Acidobacteria_Gp1				0	0	1	0	0	0	0	0	0	0	0	0	1
Cluster5495;:	Verrucom	Subdivision3			Subdivision3_g	0	0	0	0	0	0	1	0	0	0	0	0	1
Cluster5500;:	Proteobac	Alphap	Rhodospirillales			0	0	0	0	0	0	0	0	0	0	1	0	1
Cluster5502;:	Proteobac	Deltap	Myxococcales			0	0	0	0	0	0	0	0	0	0	0	1	1
Cluster5504;:	Acidobact	Acidobacteria_Gp2			Gp2	0	0	0	0	0	0	0	0	0	0	0	1	1
Cluster5512;:	Proteobacteria					0	0	0	0	0	0	1	0	0	0	0	0	1
Cluster5528;:	Actinobac	Actinol	Actinomycetales			0	0	0	0	0	0	1	0	0	0	0	0	1
Cluster5531;:	Proteobac	Gammaproteobacteria				1	0	0	0	0	0	0	0	0	0	0	0	1
Cluster5542;:	Proteobac	Gamm	Xantl	Sinobac	Steroidobacter	1	0	0	0	0	0	0	0	0	0	0	0	1
Cluster5544;:	Proteobac	Deltap	Myxococcales			0	0	0	0	0	0	1	0	0	0	0	0	1
Cluster5547;:	Acidobact	Acidobacteria_Gp3				0	0	0	0	0	0	0	0	1	0	0	0	1
Cluster5551;size=4						0	0	0	0	0	1	0	0	0	0	0	0	1
Cluster5571;:	Proteobac	Deltaproteobacteria				0	0	0	0	0	0	0	1	0	0	0	0	1

Cluster5580;	Acidobact	Acidobacteria_Gp1			Gp1	0	0	0	0	0	0	0	0	1	0	0	0	1
Cluster5583;	Verrucom	Subdivision3			Subdivision3_g	0	0	0	1	0	0	0	0	0	0	0	0	1
Cluster5584;	Armatimonadetes				Armatimonade	0	0	0	1	0	0	0	0	0	0	0	0	1
Cluster5590;	Acidobact	Acidobacteria_Gp2			Gp2	1	0	0	0	0	0	0	0	0	0	0	0	1
Cluster5591;	Proteobacteria					0	0	0	0	0	0	0	0	0	0	0	1	1
Cluster5595;	Proteobac	Alphaproteobacteria				0	0	0	0	1	0	0	0	0	0	0	0	1
Cluster5598;	Proteobac	Alphap	Rhizobiales			1	0	0	0	0	0	0	0	0	0	0	0	1
Cluster5608;	Acidobact	Acidobacteria_Gp2			Gp2	0	0	0	1	0	0	0	0	0	0	0	0	1
Cluster5627;	Firmicutes	Bacilli	Bacill	Staphyl	Staphylococcu	0	0	0	0	0	0	0	1	0	0	0	0	1
Cluster5629;	Acidobacteria					0	0	0	1	0	0	0	0	0	0	0	0	1
Cluster5635;	Proteobac	Alphap	Rhizo	Beijerinckiaceae		0	0	0	0	0	0	0	0	0	0	1	0	1
Cluster5640;	Verrucom	Opituta	Opitu	Opituta	Opitutus	1	0	0	0	0	0	0	0	0	0	0	0	1
Cluster5645;	Proteobacteria					1	0	0	0	0	0	0	0	0	0	0	0	1
Cluster5646;	Acidobact	Acidobacteria_Gp2			Gp2	0	0	0	1	0	0	0	0	0	0	0	0	1
Cluster5649;	Acidobact	Acidobacteria_Gp1			Gp1	0	0	0	0	0	0	0	0	1	0	0	0	1
Cluster5653;	Proteobac	Alphap	Caulo	Cauloba	Phenylobacter	0	0	0	0	0	0	0	0	0	0	1	0	1
Cluster5654;	Proteobac	Alphaproteobacteria				0	0	0	0	0	0	1	0	0	0	0	0	1
Cluster5655;size=6						0	0	0	0	0	1	0	0	0	0	0	0	1
Cluster5659;	Proteobac	Gamm	Xanth	Sinobac	Steroidobacter	0	0	0	0	0	0	1	0	0	0	0	0	1
Cluster5665;	Actinobac	Actinol	Actinomycetales			0	0	0	1	0	0	0	0	0	0	0	0	1
Cluster5668;	Acidobact	Acidobacteria_Gp3			Gp3	0	0	0	0	0	0	0	1	0	0	0	0	1
Cluster5678;	Proteobac	Alphap	Rhodospirillales			0	0	0	0	0	0	0	0	0	0	0	1	1
Cluster5685;	Proteobac	Alphap	Rhizo	Bradyrh	Bradyrhizobiur	0	0	0	0	0	0	1	0	0	0	0	0	1
Cluster5688;size=1						0	0	0	0	0	0	0	0	0	0	1	0	1
Cluster5691;	Planctomycetes					0	0	0	0	0	0	0	0	0	0	0	1	1
Cluster5693;	Proteobac	Alphap	Rhodospirillales			1	0	0	0	0	0	0	0	0	0	0	0	1
Cluster5696;	Acidobact	Acidobacteria_Gp3			Gp3	0	0	0	0	0	0	0	0	0	0	1	0	1
Cluster5699;	Planctomy	Plancto	Planc	Plancto	Singulisphaera	0	0	0	0	1	0	0	0	0	0	0	0	1
Cluster5707;	Acidobact	Acidobacteria_Gp1			Terriglobus	0	0	0	0	0	0	0	1	0	0	0	0	1

Cluster5717;	Acidobact	Acidobacteria_Gp2			Gp2	0	0	0	0	0	0	0	0	0	1	0	0	1
Cluster5721;	Verrucom	Opituta	Opitu	Opituta	Opitutus	0	1	0	0	0	0	0	0	0	0	0	0	1
Cluster5722;	Acidobact	Acidobacteria_Gp2			Gp2	0	0	0	1	0	0	0	0	0	0	0	0	1
Cluster5724;size=2						1	0	0	0	0	0	0	0	0	0	0	0	1
Cluster5725;	Proteobac	Alphap	Rhizc	Bradyrh	Agromonas	0	0	0	0	0	0	0	1	0	0	0	0	1
Cluster5726;	Planctomy	Plancto	Planc	Planctoi	Planctomyces	0	0	0	0	0	1	0	0	0	0	0	0	1
Cluster5728;size=3						0	0	0	0	0	0	1	0	0	0	0	0	1
Cluster5734;	Actinobac	Actinobacteria				0	0	0	0	0	1	0	0	0	0	0	0	1
Cluster5737;	Acidobact	Acidobacteria_Gp1				0	0	0	0	1	0	0	0	0	0	0	0	1
Cluster5739;	Proteobac	Betapr	Burkl	Oxalobacteraceae		1	0	0	0	0	0	0	0	0	0	0	0	1
Cluster5740;	Acidobact	Acidobacteria_Gp1			Gp1	0	0	0	0	0	0	0	0	0	0	1	0	1
Cluster5741;size=2						1	0	0	0	0	0	0	0	0	0	0	0	1
Cluster5744;size=1						0	0	0	0	0	0	1	0	0	0	0	0	1
Cluster5750;	Verrucom	Subdivision3			Subdivision3_g	0	0	0	0	1	0	0	0	0	0	0	0	1
Cluster5756;	Proteobac	Alphap	Rhod	Acetobacteraceae		0	0	0	0	0	0	0	0	0	1	0	0	1
Cluster5759;	Acidobact	Acidobacteria_Gp2			Gp2	0	0	1	0	0	0	0	0	0	0	0	0	1
Cluster5763;	Actinobac	Actinol	Actinomycetales			0	0	0	0	0	0	0	0	1	0	0	0	1
Cluster5768;	TM7				TM7_genera_i	0	0	0	0	0	0	0	0	0	0	1	0	1
Cluster5773;size=1						0	0	0	0	0	0	0	0	0	0	0	1	1
Cluster5774;	Acidobact	Acidobacteria_Gp3				0	0	0	0	0	1	0	0	0	0	0	0	1
Cluster5778;	Acidobact	Acidobacteria_Gp1			Terriglobus	0	0	0	0	1	0	0	0	0	0	0	0	1
Cluster5779;	Acidobact	Acidobacteria_Gp2			Gp2	0	0	0	0	0	0	1	0	0	0	0	0	1
Cluster5790;size=1						0	0	0	0	0	0	1	0	0	0	0	0	1
Cluster5793;	Proteobac	Alphap	Rhod	Rhodospirillaceae		0	0	0	0	0	0	0	0	0	0	1	0	1
Cluster5813;	Acidobact	Acidobacteria_Gp2			Gp2	1	0	0	0	0	0	0	0	0	0	0	0	1
Cluster5816;	Acidobact	Acidobacteria_Gp2			Gp2	0	0	0	0	0	0	0	1	0	0	0	0	1
Cluster5837;	Acidobact	Acidobacteria_Gp1			Terriglobus	0	0	0	0	0	0	0	1	0	0	0	0	1
Cluster5839;	Acidobact	Acidobacteria_Gp1				0	0	0	0	0	0	0	0	0	1	0	0	1
Cluster5840;	Actinobac	Actinol	Acidi	Acidimi	Aciditerrimona	0	0	0	0	0	0	1	0	0	0	0	0	1

Cluster5844;	Verrucom	Opituta	Opitu	Opituta	Opitutus	1	0	0	0	0	0	0	0	0	0	0	0	1
Cluster5847;	Acidobact	Acidobacteria_Gp1				0	0	0	0	0	0	0	0	1	0	0	0	1
Cluster5855;	Proteobac	Betapr	Burkl	Burkhol	Burkholderia	0	0	0	1	0	0	0	0	0	0	0	0	1
Cluster5863;	Proteobac	Alphap	Rhodospirillales			0	0	0	0	0	0	0	0	0	0	1	0	1
Cluster5864;	Actinobac	Actinol	Actinomycetales			0	0	0	0	0	0	0	0	0	0	1	0	1
Cluster5868;	Acidobact	Acidobacteria_Gp1				0	0	0	0	0	0	0	0	1	0	0	0	1
Cluster5876;	Proteobac	Alphap	Rhod	Rhodospirillaceae		0	0	0	0	1	0	0	0	0	0	0	0	1
Cluster5880;	Planctomy	Plancto	Planc	Planctomycetaceae		0	0	0	0	1	0	0	0	0	0	0	0	1
Cluster5882;	Acidobact	Acidobacteria_Gp2			Gp2	0	0	0	0	1	0	0	0	0	0	0	0	1
Cluster5883;	Proteobac	Deltap	Bdell	Bdellovi	Vampirovibrio	0	0	0	0	0	0	0	0	0	1	0	0	1
Cluster5889;	Proteobac	Gammaproteobacteria				0	0	0	0	1	0	0	0	0	0	0	0	1
Cluster5895;	Proteobac	Alphap	Rhizo	Bradyrh	Bradyrhizobiur	0	0	0	0	0	0	0	0	1	0	0	0	1
Cluster5896;	Proteobac	Alphap	Rhizo	Beijerinckiaceae		0	0	0	1	0	0	0	0	0	0	0	0	1
Cluster5899;	Acidobact	Acidobacteria_Gp13			Gp13	0	1	0	0	0	0	0	0	0	0	0	0	1
Cluster5914;	Proteobac	Alphaproteobacteria				0	0	0	0	1	0	0	0	0	0	0	0	1
Cluster5921;	Planctomy	Plancto	Planc	Plancto	Singulisphaera	0	0	0	0	1	0	0	0	0	0	0	0	1
Cluster5924;	Proteobac	Alphap	Rhod	Acetobacteraceae		0	0	0	0	0	1	0	0	0	0	0	0	1
Cluster5927;	Verrucom	Subdivision3			Subdivision3_g	0	0	0	0	0	1	0	0	0	0	0	0	1
Cluster5932;	Acidobact	Acidobacteria_Gp1				0	0	0	0	0	0	0	0	1	0	0	0	1
Cluster5941;size=1						0	0	1	0	0	0	0	0	0	0	0	0	1
Cluster5942;	Proteobac	Alphap	Rhod	Acetoba	Roseomonas	0	0	0	0	0	0	0	0	0	0	1	0	1
Cluster5954;	Verrucom	Spartobacteria			Spartobacteria	0	0	0	1	0	0	0	0	0	0	0	0	1
Cluster5963;	Acidobact	Acidobacteria_Gp1				0	0	0	1	0	0	0	0	0	0	0	0	1
Cluster5970;	Proteobacteria					0	0	0	0	1	0	0	0	0	0	0	0	1
Cluster5974;size=1						1	0	0	0	0	0	0	0	0	0	0	0	1
Cluster5978;size=6						0	0	0	0	0	1	0	0	0	0	0	0	1
Cluster5983;	Verrucom	Opituta	Opitu	Opituta	Opitutus	1	0	0	0	0	0	0	0	0	0	0	0	1
Cluster5987;	Verrucom	Subdivision3			Subdivision3_g	0	0	0	0	0	0	0	0	0	0	0	1	1
Cluster5992;	Proteobac	Alphap	Rhizo	Methylocystaceae		0	0	0	1	0	0	0	0	0	0	0	0	1

Cluster5994;:	Proteobac	Alphaproteobacteria				0	0	0	0	0	1	0	0	0	0	0	0	1
Cluster5995;:	Actinobac	Actinol	Actin	Pseudonocardiaceae		0	0	0	0	0	0	1	0	0	0	0	0	1
Cluster6000;:	Firmicute:	Negati	Seler	Veillonellaceae		0	0	0	0	0	0	0	0	0	0	0	1	1
Cluster6003;:	Verrucom	Subdivision3			Subdivision3_g	0	0	0	0	0	0	0	1	0	0	0	0	1
Cluster6010;:	Verrucom	Subdivision3			Subdivision3_g	0	0	0	0	0	0	1	0	0	0	0	0	1
Cluster6027;:	Proteobac	Alphaproteobacteria				0	0	1	0	0	0	0	0	0	0	0	0	1
Cluster6035;:	Proteobac	Alphaproteobacteria				0	0	0	0	0	0	0	0	0	0	0	1	1
Cluster6041;:	Proteobac	Gamm	Xantl	Sinobac	Steroidobacter	0	0	0	0	0	0	0	0	0	0	1	0	1
Cluster6052;:	Proteobacteria					0	0	1	0	0	0	0	0	0	0	0	0	1
Cluster6056;:	Actinobac	Actinol	Actinomycetales			0	0	0	0	0	0	0	0	1	0	0	0	1
Cluster6057;:	Proteobac	Alphap	Rhizobiales			0	0	0	0	0	0	1	0	0	0	0	0	1
Cluster6065;:	Verrucom	Opitut	Opitu	Opituta	Opitutus	0	0	0	0	0	0	0	0	0	0	0	1	1
Cluster6071;:	Armatimo	Chthor	Chth	Chthon	Chthonomona	0	0	0	1	0	0	0	0	0	0	0	0	1
Cluster6072;size=4						0	1	0	0	0	0	0	0	0	0	0	0	1
Cluster6076;:	Verrucom	Subdivision3			Subdivision3_g	0	0	0	0	0	0	1	0	0	0	0	0	1
Cluster6077;:	Acidobact	Acidobacteria_Gp1			Gp1	0	0	0	0	0	0	0	0	0	0	1	0	1
Cluster6082;size=3						1	0	0	0	0	0	0	0	0	0	0	0	1
Cluster6089;:	Chloroflex	Ktedor	Kted	Thermo	Thermosporot	0	0	0	0	0	1	0	0	0	0	0	0	1
Cluster6090;:	Proteobac	Betaproteobacteria				0	0	0	1	0	0	0	0	0	0	0	0	1
Cluster6091;:	Actinobac	Actinol	Solirubrobacterales			1	0	0	0	0	0	0	0	0	0	0	0	1
Cluster6096;:	Acidobact	Acidobacteria_Gp3				0	0	1	0	0	0	0	0	0	0	0	0	1
Cluster6097;:	Acidobact	Acidobacteria_Gp1			Acidobacterium	1	0	0	0	0	0	0	0	0	0	0	0	1
Cluster6098;size=4						0	0	0	0	0	0	1	0	0	0	0	0	1
Cluster6108;:	Proteobac	Betapr	Burkholderiales			0	0	0	0	0	1	0	0	0	0	0	0	1
Cluster6110;size=4						0	0	0	0	0	0	0	0	0	0	1	0	1
Cluster6111;:	Verrucom	Subdivision3			Subdivision3_g	0	0	1	0	0	0	0	0	0	0	0	0	1
Cluster6113;:	Proteobac	Alphap	Rhizobiales			0	0	0	1	0	0	0	0	0	0	0	0	1
Cluster6117;:	Proteobac	Alphap	Rhodospirillales			0	0	0	0	1	0	0	0	0	0	0	0	1
Cluster6118;:	Proteobac	Betapr	Burkl	Burkhol	Burkholderia	0	0	1	0	0	0	0	0	0	0	0	0	1

Cluster6122;	Acidobact	Acidobacteria_Gp2			Gp2	0	0	0	0	0	1	0	0	0	0	0	0	1
Cluster6123;size=1						0	0	0	1	0	0	0	0	0	0	0	0	1
Cluster6124;size=1						0	0	0	0	0	1	0	0	0	0	0	0	1
Cluster6128;	Acidobact	Acidobacteria_Gp1			Terriglobus	0	0	0	0	1	0	0	0	0	0	0	0	1
Cluster6131;	Actinobac	Actinol	Actinomycetales			1	0	0	0	0	0	0	0	0	0	0	0	1
Cluster6132;	Acidobact	Acidobacteria_Gp2			Gp2	0	0	0	0	0	0	1	0	0	0	0	0	1
Cluster6140;size=1						0	0	0	1	0	0	0	0	0	0	0	0	1
Cluster6144;	Proteobac	Alphap	Rhizc	Xanthol	Pseudolabrys	0	0	0	0	0	0	0	0	0	0	1	0	1
Cluster6157;	Elusimicro	Elusim	Elusi	Elusimic	Elusimicrobium	1	0	0	0	0	0	0	0	0	0	0	0	1
Cluster6166;	Proteobac	Alphap	Rhizobiales			0	0	0	0	0	0	1	0	0	0	0	0	1
Cluster6176;size=1						0	0	0	0	0	0	0	0	0	0	1	0	1
Cluster6184;size=1						0	0	0	0	0	0	0	1	0	0	0	0	1
Cluster6191;	Proteobac	Alphaproteobacteria				1	0	0	0	0	0	0	0	0	0	0	0	1
Cluster6199;size=6						0	0	0	0	0	0	0	0	0	0	1	0	1
Cluster6201;	Acidobact	Acidobacteria_Gp2			Gp2	0	0	0	0	0	1	0	0	0	0	0	0	1
Cluster6204;	Proteobac	Alphap	Rhodospirillales			0	0	0	0	0	1	0	0	0	0	0	0	1
Cluster6206;	Proteobacteria					0	0	0	0	0	0	0	1	0	0	0	0	1
Cluster6211;	Acidobact	Acidobacteria_Gp1			Edaphobacter	0	0	0	0	0	0	0	0	1	0	0	0	1
Cluster6212;	Proteobac	Alphap	Rhizc	Xanthol	Pseudolabrys	0	0	0	1	0	0	0	0	0	0	0	0	1
Cluster6225;	Proteobac	Alphap	Rhodospirillales			0	0	0	0	0	0	0	0	0	0	1	0	1
Cluster6229;	Acidobact	Acidobacteria_Gp1				1	0	0	0	0	0	0	0	0	0	0	0	1
Cluster6239;	Proteobac	Alphap	Rhizc	Bradyrhizobiaceae		0	0	0	1	0	0	0	0	0	0	0	0	1
Cluster6243;size=2						0	0	0	0	0	0	0	1	0	0	0	0	1
Cluster6252;	Proteobac	Alphap	Rhodospirillales			0	0	1	0	0	0	0	0	0	0	0	0	1
Cluster6255;	Acidobact	Acidobacteria_Gp3				0	1	0	0	0	0	0	0	0	0	0	0	1
Cluster6274;size=2						0	0	0	0	0	0	0	1	0	0	0	0	1
Cluster6280;	Acidobact	Acidobacteria_Gp1				0	0	0	0	0	0	0	0	0	0	0	1	1
Cluster6281;	Verrucom	Subdivision3			Subdivision3_g	0	0	0	0	0	0	0	0	0	0	0	1	1
Cluster6282;size=14						0	0	0	0	0	1	0	0	0	0	0	0	1

Cluster6285;:	Proteobac	Alphap	Rhod	Acetobacteraceae		0	0	0	0	0	0	0	0	0	0	1	0	1
Cluster6286;size=4						0	0	0	0	0	0	0	0	0	0	1	0	1
Cluster6293;:	Proteobac	Alphap	Rhodospirillales			0	0	0	0	0	0	0	0	0	0	1	0	1
Cluster6297;:	Actinobac	Actinobacteria				0	0	0	1	0	0	0	0	0	0	0	0	1
Cluster6309;:	Proteobac	Alphaproteobacteria				0	0	0	0	0	0	1	0	0	0	0	0	1
Cluster6315;:	Proteobac	Alphap	Rhizobiales			0	0	0	0	0	0	1	0	0	0	0	0	1
Cluster6321;:	Acidobact	Acidobacteria_Gp1			Terriglobus	0	0	0	1	0	0	0	0	0	0	0	0	1
Cluster6341;:	Actinobac	Actinol	Acidimicrobiales			0	0	0	0	0	0	1	0	0	0	0	0	1
Cluster6345;:	Proteobac	Alphap	Rhodospirillales			0	0	0	0	0	0	0	0	1	0	0	0	1
Cluster6351;size=1						0	0	0	1	0	0	0	0	0	0	0	0	1
Cluster6357;size=2						0	1	0	0	0	0	0	0	0	0	0	0	1
Cluster6364;:	Verrucom	Subdivision3			Subdivision3_g	1	0	0	0	0	0	0	0	0	0	0	0	1
Cluster6372;:	Proteobac	Deltap	Myxc	Polyangiaceae		1	0	0	0	0	0	0	0	0	0	0	0	1
Cluster6381;:	Proteobac	Alphap	Rhodospirillales			0	0	0	0	0	0	0	0	0	0	0	1	1
Cluster6384;:	Acidobact	Acidobacteria_Gp2			Gp2	0	0	0	1	0	0	0	0	0	0	0	0	1
Cluster6385;size=1						0	0	0	0	0	0	1	0	0	0	0	0	1
Cluster6387;:	Proteobac	Alphaproteobacteria				0	0	0	1	0	0	0	0	0	0	0	0	1
Cluster6391;size=1						0	0	0	1	0	0	0	0	0	0	0	0	1
Cluster6392;:	Proteobac	Deltap	Myxococcales			0	0	0	1	0	0	0	0	0	0	0	0	1
Cluster6393;:	Verrucom	Spartobacteria			Spartobacteria	0	0	1	0	0	0	0	0	0	0	0	0	1
Cluster6400;:	Verrucom	Spartobacteria			Spartobacteria	0	0	0	0	0	0	0	1	0	0	0	0	1
Cluster6407;:	Actinobac	Actinol	Actinomycetales			0	0	0	0	0	0	0	0	0	0	0	1	1
Cluster6416;size=2						0	0	0	0	0	0	0	0	0	1	0	0	1
Cluster6421;:	Acidobact	Acidobacteria_Gp1			Gp1	0	0	0	0	0	1	0	0	0	0	0	0	1
Cluster6431;size=1						0	0	1	0	0	0	0	0	0	0	0	0	1
Cluster6432;size=4						0	0	0	0	0	0	0	0	0	0	1	0	1
Cluster6438;:	Verrucom	Opituta	Opitu	Opituta	Opitutus	0	0	0	0	0	1	0	0	0	0	0	0	1
Cluster6442;:	Proteobac	Alphap	Rhizobiales			0	0	0	0	0	0	0	0	0	0	1	0	1
Cluster6448;:	Acidobact	Acidobacteria_Gp1			Acidobacteriur	0	0	0	0	1	0	0	0	0	0	0	0	1

Cluster6458;	Actinobac	Actinol	Actin	Pseudonocardiaceae		0	0	0	0	0	0	0	0	0	0	1	0	1
Cluster6462;size=1						0	0	0	1	0	0	0	0	0	0	0	0	1
Cluster6470;size=2						0	0	1	0	0	0	0	0	0	0	0	0	1
Cluster6473;size=1						0	0	0	0	0	0	1	0	0	0	0	0	1
Cluster6474;	Verrucom	Subdivision3			Subdivision3_g	0	0	0	1	0	0	0	0	0	0	0	0	1
Cluster6482;	Acidobact	Acidobacteria_Gp2			Gp2	0	0	1	0	0	0	0	0	0	0	0	0	1
Cluster6487;	Acidobact	Acidobacteria_Gp2			Gp2	0	0	0	0	0	0	0	0	0	0	0	1	1
Cluster6492;	Verrucom	Subdivision3			Subdivision3_g	0	0	0	0	0	0	0	0	0	0	0	1	1
Cluster6494;	Verrucom	Opitut	Opitu	Opituta	Opitutus	0	1	0	0	0	0	0	0	0	0	0	0	1
Cluster6495;	Verrucom	Spartobacteria			Spartobacteria	0	0	0	0	0	0	1	0	0	0	0	0	1
Cluster6504;	Acidobact	Acidobacteria_Gp1			Gp1	0	0	0	0	0	0	0	0	0	0	0	1	1
Cluster6506;	Proteobac	Deltap	Myxococcales			0	0	0	1	0	0	0	0	0	0	0	0	1
Cluster6507;	Acidobact	Acidobacteria_Gp1				0	0	1	0	0	0	0	0	0	0	0	0	1
Cluster6512;	Proteobac	Alphap	Rhizo	Bradyrh	Bradyrhizobiur	0	0	0	0	0	0	0	0	0	0	0	1	1
Cluster6518;size=1						0	0	0	0	0	0	0	1	0	0	0	0	1
Cluster6529;	Proteobac	Deltap	Myxococcales			0	0	0	0	0	0	1	0	0	0	0	0	1
Cluster6537;	Armatimonadetes				Armatimonade	0	0	0	0	0	0	1	0	0	0	0	0	1
Cluster6543;	Proteobac	Deltap	Bdell	Bdellovi	Vampirovibrio	0	0	0	0	0	0	0	0	0	0	0	1	1
Cluster6544;	Gemmatir	Gemm	Gemi	Gemma	Gemmatimona	0	0	0	1	0	0	0	0	0	0	0	0	1
Cluster6551;	Acidobact	Acidobacteria_Gp2			Gp2	0	0	0	0	0	0	1	0	0	0	0	0	1
Cluster6555;	Verrucom	Opitut	Opitu	Opituta	Opitutus	1	0	0	0	0	0	0	0	0	0	0	0	1
Cluster6565;size=2						0	1	0	0	0	0	0	0	0	0	0	0	1
Cluster6570;	Proteobac	Deltap	Myxococcales			1	0	0	0	0	0	0	0	0	0	0	0	1
Cluster6576;	Verrucom	Subdivision3			Subdivision3_g	0	0	0	0	0	0	1	0	0	0	0	0	1
Cluster6579;	Acidobact	Acidobacteria_Gp1			Gp1	0	0	0	0	0	0	0	0	0	0	1	0	1
Cluster6586;	Proteobac	Alphaproteobacteria				0	0	0	0	0	0	0	0	1	0	0	0	1
Cluster6599;	Verrucom	Subdivision3			Subdivision3_g	1	0	0	0	0	0	0	0	0	0	0	0	1
Cluster6600;	Firmicutes	Negati	Seler	Veillonellaceae		0	0	0	1	0	0	0	0	0	0	0	0	1
Cluster6623;size=4						0	0	0	0	0	1	0	0	0	0	0	0	1

Cluster6624;	Acidobact	Acidobacteria_Gp3			Gp3	0	0	0	0	0	0	0	0	0	0	0	1	1
Cluster6629;	Acidobact	Acidobacteria_Gp1			Gp1	0	0	0	0	0	0	0	0	1	0	0	0	1
Cluster6643;size=3						0	1	0	0	0	0	0	0	0	0	0	0	1
Cluster6645;	Proteobac	Alphap	Rhodospirillales			0	0	0	0	0	0	0	0	0	0	1	0	1
Cluster6651;	Acidobact	Acidobacteria_Gp1				0	0	0	0	0	0	0	0	0	0	0	1	1
Cluster6652;	Actinobac	Actinob	Actin	Actinos	Actinospica	0	0	0	0	0	0	0	0	0	0	0	1	1
Cluster6661;size=1						0	0	0	1	0	0	0	0	0	0	0	0	1
Cluster6663;	Elusimicro	Elusim	Elusi	Elusimic	Elusimicrobiun	0	1	0	0	0	0	0	0	0	0	0	0	1
Cluster6664;	Proteobac	Betapr	Rhod	Rhodocyclaceae		0	0	0	0	0	0	1	0	0	0	0	0	1
Cluster6665;	Verrucom	Spartobacteria			Spartobacteria	0	0	0	1	0	0	0	0	0	0	0	0	1
Cluster6676;	Proteobac	Alphap	Rhodospirillales			0	0	0	1	0	0	0	0	0	0	0	0	1
Cluster6681;	Verrucom	Verruc	Verru	Verruco	Verrucomicrob	0	0	0	0	0	1	0	0	0	0	0	0	1
Cluster6682;	Acidobact	Acidobacteria_Gp2			Gp2	0	0	0	0	0	0	0	0	0	0	0	1	1
Cluster6692;	Acidobact	Acidobacteria_Gp2			Gp2	0	0	0	1	0	0	0	0	0	0	0	0	1
Cluster6693;	Acidobact	Acidobacteria_Gp1			Gp1	0	0	0	0	0	0	0	0	1	0	0	0	1
Cluster6694;	Verrucom	Opitutae				0	0	0	0	0	1	0	0	0	0	0	0	1
Cluster6698;	Planctomy	Plancto	Planc	Plancto	Singulisphaera	0	0	0	0	0	0	1	0	0	0	0	0	1
Cluster6703;	Acidobact	Acidobacteria_Gp3			Gp3	0	0	0	1	0	0	0	0	0	0	0	0	1
Cluster6707;	Proteobac	Alphap	Rhodospirillales			0	0	0	0	1	0	0	0	0	0	0	0	1
Cluster6719;	Verrucom	Spartobacteria			Spartobacteria	0	0	0	0	1	0	0	0	0	0	0	0	1
Cluster6723;	Proteobac	Alphap	Rhodospirillales			0	0	0	0	0	1	0	0	0	0	0	0	1
Cluster6727;	Proteobac	Alphap	Rhizo	Bradyrh	Agromonas	0	0	0	1	0	0	0	0	0	0	0	0	1
Cluster6730;	Verrucom	Opitut	Opitu	Opituta	Opitutus	0	0	0	0	1	0	0	0	0	0	0	0	1
Cluster6743;	Verrucom	Subdivision3			Subdivision3_g	1	0	0	0	0	0	0	0	0	0	0	0	1
Cluster6747;	Proteobac	Betapr	Burkl	Burkhol	Burkholderia	0	0	0	0	0	1	0	0	0	0	0	0	1
Cluster6751;size=1						0	0	0	0	1	0	0	0	0	0	0	0	1
Cluster6759;	Proteobac	Alphap	Rhod	Rhodos	Dongia	0	0	0	0	0	0	0	0	0	0	1	0	1
Cluster6764;size=3						0	0	0	0	0	0	0	0	0	0	1	0	1
Cluster6767;size=4						0	0	1	0	0	0	0	0	0	0	0	0	1

Cluster6768;	Proteobacteria					0	0	0	0	0	0	0	0	0	0	1	0	1
Cluster6769;	Proteobac	Alphap	Rhizc	Bradyrhizobiaceae		0	0	0	0	0	0	0	0	0	0	0	1	1
Cluster6771;	Actinobac	Actinol	Solirı	Conexib	Conexibacter	0	0	0	0	0	0	0	0	0	0	1	0	1
Cluster6772;	Proteobac	Alphap	Rhod	Acetoba	Acidisphaera	0	0	0	0	0	0	0	1	0	0	0	0	1
Cluster6775;	Acidobact	Acidobacteria_Gp1			Gp1	0	0	0	0	1	0	0	0	0	0	0	0	1
Cluster6787;	Proteobac	Alphap	Rhodospirillales			0	0	1	0	0	0	0	0	0	0	0	0	1
Cluster6796;	Actinobac	Actinol	Acidi	Acidimi	Aciditerrimona	0	0	0	0	0	0	0	0	0	0	1	0	1
Cluster6797;	Proteobac	Alphap	Rhizc	Xanthol	Labrys	0	0	0	0	0	0	0	1	0	0	0	0	1
Cluster6800;	Acidobact	Acidobacteria_Gp3			Gp3	0	0	0	0	0	0	1	0	0	0	0	0	1
Cluster6809;	Proteobac	Alphap	Rhod	Acetobacteraceae		0	0	0	0	1	0	0	0	0	0	0	0	1
Cluster6810;	Proteobac	Alphap	Alphaproteol		Rhizomicrobiu	0	0	1	0	0	0	0	0	0	0	0	0	1
Cluster6813;	Proteobac	Alphap	Rhizc	Bradyrhizobiaceae		0	0	0	0	0	0	0	0	0	0	1	0	1
Cluster6818;	Acidobact	Acidobacteria_Gp2			Gp2	0	0	0	0	0	0	0	1	0	0	0	0	1
Cluster6823;	Proteobac	Alphaproteobacteria				0	1	0	0	0	0	0	0	0	0	0	0	1
Cluster6824;	Chloroflex	Ktedor	Ktedonobacterales			0	0	0	0	0	0	0	1	0	0	0	0	1
Cluster6825;	Proteobac	Alphap	Rhodospirillales			0	0	0	0	0	0	0	1	0	0	0	0	1
Cluster6834;	Acidobact	Acidobacteria_Gp10			Gp10	1	0	0	0	0	0	0	0	0	0	0	0	1
Cluster6835;	Proteobac	Alphap	Rhodospirillales			0	0	0	0	0	0	0	0	0	0	1	0	1
Cluster6839;	Proteobacteria					0	0	0	0	1	0	0	0	0	0	0	0	1
Cluster6843;	Acidobact	Acidobacteria_Gp10			Gp10	0	0	0	0	1	0	0	0	0	0	0	0	1
Cluster6844;size=1						0	0	0	0	1	0	0	0	0	0	0	0	1
Cluster6856;	Actinobac	Actinol	Acidi	Acidimi	Aciditerrimona	0	0	0	0	0	0	0	0	0	0	1	0	1
Cluster6863;size=1						0	0	0	0	0	0	1	0	0	0	0	0	1
Cluster6864;	Firmicute	Bacilli	Bacill	Bacillac	Falsibacillus	0	0	0	0	0	0	1	0	0	0	0	0	1
Cluster6870;	Acidobact	Acidobacteria_Gp2			Gp2	0	0	0	0	0	0	1	0	0	0	0	0	1
Cluster6879;	Actinobac	Actinol	Actinomycetales			0	0	0	0	0	0	1	0	0	0	0	0	1
Cluster6889;size=1						0	0	0	0	0	0	1	0	0	0	0	0	1
Cluster6891;	Proteobac	Betapr	Rhod	Rhodocyclaceae		0	0	1	0	0	0	0	0	0	0	0	0	1
Cluster6892;size=3						0	0	0	0	0	0	0	0	0	0	1	0	1

Cluster6893;	Acidobact	Acidobacteria_Gp1			Gp1	0	0	0	0	0	0	0	1	0	0	0	0	1
Cluster6901;	Proteobac	Deltaproteobacteria				0	0	0	0	0	0	0	0	0	0	1	0	1
Cluster6902;	Proteobac	Alphap	Rhodospirillales			0	0	0	0	1	0	0	0	0	0	0	0	1
Cluster6903;	Proteobac	Alphaproteobacteria				0	0	0	0	0	0	0	0	1	0	0	0	1
Cluster6913;	Verrucom	Opituta	Opitu	Opituta	Opitutus	1	0	0	0	0	0	0	0	0	0	0	0	1
Cluster6917;	Actinobac	Actinol	Acidi	Acidimi	Aciditerrimona	0	0	0	0	0	1	0	0	0	0	0	0	1
Cluster6921;	Proteobac	Betapr	Rhod	Rhodocyclaceae		0	0	0	0	1	0	0	0	0	0	0	0	1
Cluster6926;	Proteobac	Alphap	Rhodospirillales			0	0	0	0	0	0	0	0	0	0	1	0	1
Cluster6927;	Acidobact	Acidobacteria_Gp6			Gp6	0	0	0	0	0	0	0	1	0	0	0	0	1
Cluster6928;size=2						0	1	0	0	0	0	0	0	0	0	0	0	1
Cluster6933;size=3						0	0	0	1	0	0	0	0	0	0	0	0	1
Cluster6934;	Proteobacteria					0	0	0	0	1	0	0	0	0	0	0	0	1
Cluster6942;	Acidobact	Acidobacteria_Gp2			Gp2	0	0	0	0	1	0	0	0	0	0	0	0	1
Cluster6947;size=1						0	0	0	0	0	0	0	0	0	0	0	1	1
Cluster6948;	Proteobac	Alphap	Rhizobiales			0	0	0	0	0	0	0	0	0	0	0	1	1
Cluster6949;	Acidobact	Acidobacteria_Gp10			Gp10	0	0	0	0	0	0	1	0	0	0	0	0	1
Cluster6952;	Proteobac	Betapr	Burkl	Burkhol	Burkholderia	0	0	0	0	1	0	0	0	0	0	0	0	1
Cluster6956;	Proteobac	Alphap	Rhizobiales			0	1	0	0	0	0	0	0	0	0	0	0	1
Cluster6964;	Proteobac	Deltaproteobacteria				0	0	0	0	0	0	0	0	1	0	0	0	1
Cluster6970;	Proteobacteria					0	0	0	1	0	0	0	0	0	0	0	0	1
Cluster6975;	Acidobact	Acidobacteria_Gp2			Gp2	0	0	0	0	0	0	0	0	1	0	0	0	1
Cluster6981;	Proteobacteria					0	1	0	0	0	0	0	0	0	0	0	0	1
Cluster6982;	Proteobac	Alphap	Rhizobiales			0	0	0	0	0	0	0	0	0	0	0	1	1
Cluster6986;	Verrucom	Opituta	Opitu	Opituta	Opitutus	0	0	0	0	0	0	0	0	0	0	0	1	1
Cluster6989;	Acidobact	Acidobacteria_Gp2			Gp2	0	0	0	0	0	0	0	0	0	0	0	1	1
Cluster6990;size=4						0	0	0	0	1	0	0	0	0	0	0	0	1
Cluster7002;	Acidobact	Acidobacteria_Gp1			Granulicella	0	0	0	0	0	0	0	0	0	1	0	0	1
Cluster7009;	Proteobac	Alphap	Rhodospirillales			0	0	0	0	0	0	0	0	0	0	1	0	1
Cluster7012;	Proteobac	Alphap	Rhizobiales			0	0	0	0	0	0	0	0	0	0	0	1	1

Cluster7014;	Proteobacteria					0	0	0	1	0	0	0	0	0	0	0	0	1
Cluster7015;	Acidobact	Acidobacteria_Gp3				0	0	0	0	0	0	0	0	0	0	0	1	1
Cluster7016;	Proteobac	Deltap	Bdell	Bdellovi	Vampirovibrio	0	0	0	0	0	0	0	0	0	1	0	0	1
Cluster7018;	Proteobac	Alphap	Caulc	Cauloba	Phenylobacter	0	0	0	0	0	0	0	0	0	0	1	0	1
Cluster7028;	Gemmatii	Gemm	Gemi	Gemma	Gemmatimona	0	0	0	0	1	0	0	0	0	0	0	0	1
Cluster7032;	Acidobact	Acidobacteria_Gp1			Gp1	0	0	0	0	0	0	0	0	0	0	0	1	1
Cluster7034;	Acidobact	Acidobacteria_Gp3			Bryobacter	0	0	0	0	1	0	0	0	0	0	0	0	1
Cluster7036;	Acidobact	Acidobacteria_Gp1				0	0	0	0	0	0	0	1	0	0	0	0	1
Cluster7039;	Proteobac	Alphap	Rhizc	Xanthol	Labrys	0	1	0	0	0	0	0	0	0	0	0	0	1
Cluster7040;	Actinobac	Actinol	Actinomycetales			0	0	0	0	0	0	0	0	0	1	0	0	1
Cluster7043;	Actinobac	Actinobacteria				0	0	0	0	1	0	0	0	0	0	0	0	1
Cluster7046;	Proteobac	Betapr	Burkl	Burkholderiaceae		0	0	0	0	0	0	0	0	0	1	0	0	1
Cluster7050;	Actinobac	Actinol	Actinomycetales			0	1	0	0	0	0	0	0	0	0	0	0	1
Cluster7069;	Armatimc	Armati	Arma	Armatir	Armatimonas/	0	0	0	0	1	0	0	0	0	0	0	0	1
Cluster7083;	Proteobac	Alphap	Rhizc	Beijerinckiaceae		0	0	0	0	0	0	0	0	0	0	0	1	1
Cluster7084;	Chloroflex	Ktedonobacteria				0	0	0	0	0	0	1	0	0	0	0	0	1
Cluster7088;	Proteobac	Gamm	Xantl	Xanthor	Pseudoxanthor	0	0	0	0	0	0	0	1	0	0	0	0	1
Cluster7089;	Proteobac	Alphaproteobacteria				0	0	0	0	0	0	0	1	0	0	0	0	1
Cluster7105;	Proteobac	Alphap	Rhizc	Bradyrh	Bradyrhizobiur	0	0	1	0	0	0	0	0	0	0	0	0	1
Cluster7110;	Acidobact	Acidobacteria_Gp1				0	0	0	0	0	0	0	0	0	0	0	1	1
Cluster7111;	Proteobac	Alphaproteobacteria				0	0	0	0	0	0	0	0	0	0	1	0	1
Cluster7123;	Chlamydia	Chlamy	Chlar	Parachlamydiaceae		0	0	0	0	0	0	1	0	0	0	0	0	1
Cluster7124;	Verrucomicrobia					0	0	0	0	1	0	0	0	0	0	0	0	1
Cluster7134;	Chlamydia	Chlamy	Chlamydiales			0	0	0	0	0	0	1	0	0	0	0	0	1
Cluster7138;	Acidobact	Acidobacteria_Gp3			Gp3	0	0	0	0	0	0	0	1	0	0	0	0	1
Cluster7139;	Proteobac	Alphap	Rhizc	Bradyrh	Agromonas	0	0	1	0	0	0	0	0	0	0	0	0	1
Cluster7140;	Actinobac	Actinol	Solirι	Solirubr	Solirubrobacte	0	0	0	0	0	0	0	0	0	0	1	0	1
Cluster7143;	Acidobact	Acidobacteria_Gp1				0	0	0	1	0	0	0	0	0	0	0	0	1
Cluster7144;	Armatimc	Chthor	Chth	Chthon	Chthonomona	0	0	0	0	0	0	0	0	0	0	1	0	1

Cluster7147;	Acidobact	Acidobacteria_Gp1				0	0	0	0	0	0	0	0	0	0	0	1	1
Cluster7149;size=16						0	1	0	0	0	0	0	0	0	0	0	0	1
Cluster7151;	Proteobac	Alphap	Rhodospirillales			0	0	0	0	1	0	0	0	0	0	0	0	1
Cluster7155;	Verrucom	Subdivision3			Subdivision3_g	0	0	0	0	1	0	0	0	0	0	0	0	1
Cluster7157;	Acidobact	Acidobacteria_Gp3			Gp3	0	0	1	0	0	0	0	0	0	0	0	0	1
Cluster7158;	Proteobac	Deltaproteobacteria				0	0	0	0	0	0	0	0	1	0	0	0	1
Cluster7160;	Proteobac	Alphaproteobacteria				0	0	0	1	0	0	0	0	0	0	0	0	1
Cluster7162;	Proteobac	Alphap	Rhodospirillales			0	0	0	0	1	0	0	0	0	0	0	0	1
Cluster7164;	Armatimo	Chthor	Chthc	Chthono	Chthonomona	0	0	0	0	0	0	1	0	0	0	0	0	1
Cluster7169;size=1						0	0	1	0	0	0	0	0	0	0	0	0	1
Cluster7176;	Proteobac	Gamm	Legio	Coxiella	Aquicella	0	0	0	1	0	0	0	0	0	0	0	0	1
Cluster7185;	Proteobacteria					0	0	0	0	0	0	0	0	1	0	0	0	1
Cluster7186;	Proteobac	Alphap	Rhodospirillales			0	0	0	0	0	0	0	1	0	0	0	0	1
Cluster7197;	Proteobac	Alphap	Rhodospirillales			0	0	0	0	0	0	0	0	0	1	0	0	1
Cluster7202;	Proteobacteria					0	0	0	0	0	0	0	1	0	0	0	0	1
Cluster7216;	Proteobac	Alphaproteobacteria				0	0	0	0	1	0	0	0	0	0	0	0	1
Cluster7218;	Proteobac	Betapr	Burkl	Burkhol	Burkholderia	0	0	0	0	0	0	0	0	0	0	1	0	1
Cluster7244;	Proteobacteria					0	0	0	0	0	0	0	0	0	0	0	1	1
Cluster7249;	Acidobact	Acidobacteria_Gp2			Gp2	0	0	0	0	1	0	0	0	0	0	0	0	1
Cluster7252;	Verrucom	Opitutae				0	0	0	0	0	0	1	0	0	0	0	0	1
Cluster7254;	Proteobac	Gammaproteobacteria				0	0	0	0	0	0	0	1	0	0	0	0	1
Cluster7255;size=2						0	0	0	0	0	0	1	0	0	0	0	0	1
Cluster7265;	Proteobac	Gamm	Xantl	Sinobac	Steroidobacter	0	0	0	0	0	0	0	0	0	1	0	0	1
Cluster7274;	Acidobacteria					0	0	0	0	1	0	0	0	0	0	0	0	1
Cluster7280;	Proteobac	Alphap	Rhizobiales			0	0	1	0	0	0	0	0	0	0	0	0	1
Cluster7290;	Proteobac	Alphap	Rhizc	Bradyrh	Bradyrhizobiur	0	0	0	0	0	0	0	1	0	0	0	0	1
Cluster7298;	Acidobact	Acidobacteria_Gp1			Acidobacteriur	0	0	0	0	0	0	0	1	0	0	0	0	1
Cluster7319;size=1						0	0	0	1	0	0	0	0	0	0	0	0	1
Cluster7327;size=6						0	0	0	0	0	1	0	0	0	0	0	0	1

Cluster7333;:	Verrucom	Opituta	Opitu	Opituta	Opitutus	0	0	0	0	0	1	0	0	0	0	0	0	1
Cluster7343;:	Verrucom	Spartobacteria			Spartobacteria	0	0	0	0	0	0	0	0	0	0	0	1	1
Cluster7357;:	Proteobac	Alphap	Rhizo	Bradyrh	Agromonas	0	0	0	0	0	0	0	1	0	0	0	0	1
Cluster7370;:	Acidobact	Acidobacteria_Gp1				0	0	0	0	0	0	1	0	0	0	0	0	1
Cluster7383;:	Verrucom	Spartobacteria			Spartobacteria	0	0	0	0	0	0	1	0	0	0	0	0	1
Cluster7384;:	Acidobact	Acidobacteria_Gp3			Gp3	0	0	0	0	0	0	0	0	0	0	1	0	1
Cluster7387;:	Proteobac	Alphap	Rhodospirillales			0	1	0	0	0	0	0	0	0	0	0	0	1
Cluster7392;:	Verrucom	Opituta	Opitu	Opituta	Opitutus	0	1	0	0	0	0	0	0	0	0	0	0	1
Cluster7400;:	Proteobac	Alphap	Rhodospirillales			0	1	0	0	0	0	0	0	0	0	0	0	1
Cluster7401;:	Acidobact	Acidobacteria_Gp2			Gp2	1	0	0	0	0	0	0	0	0	0	0	0	1
Cluster7402;:	Acidobact	Acidobacteria_Gp1			Acidobacteriur	0	0	1	0	0	0	0	0	0	0	0	0	1
Cluster7406;:	Proteobac	Alphap	Rhizobiales			0	0	0	1	0	0	0	0	0	0	0	0	1
Cluster7409;:	Armatimonadetes				Armatimonade	0	0	0	0	0	0	1	0	0	0	0	0	1
Cluster7418;:	Proteobac	Deltap	Myxococcales			1	0	0	0	0	0	0	0	0	0	0	0	1
Cluster7421;:	Acidobact	Acidobacteria_Gp2			Gp2	0	0	0	0	0	0	1	0	0	0	0	0	1
Cluster7424;:	Firmicutes					0	0	0	0	0	0	1	0	0	0	0	0	1
Cluster7427;:	Acidobact	Acidobacteria_Gp2			Gp2	0	0	0	1	0	0	0	0	0	0	0	0	1
Cluster7429;:	Acidobact	Acidobacteria_Gp2			Gp2	0	0	0	1	0	0	0	0	0	0	0	0	1
Cluster7433;:	Acidobact	Acidobacteria_Gp2			Gp2	0	0	0	0	0	1	0	0	0	0	0	0	1
Cluster7439;:	Acidobact	Acidobacteria_Gp1			Acidobacteriur	0	0	1	0	0	0	0	0	0	0	0	0	1
Cluster7440;:	Proteobacteria					0	0	0	0	0	0	0	0	0	0	0	1	1
Cluster7446;:	Actinobac	Actinobacteria				0	0	1	0	0	0	0	0	0	0	0	0	1
Cluster7447;:	Proteobac	Betapr	Burkl	Burkholderiales_incert		0	0	0	0	0	0	0	1	0	0	0	0	1
Cluster7462;:	Proteobac	Alphaproteobacteria				0	0	0	0	0	0	0	1	0	0	0	0	1
Cluster7463;:	Acidobact	Acidobacteria_Gp2			Gp2	0	0	0	0	0	0	0	0	0	0	1	0	1
Cluster7469;:	Proteobac	Alphaproteobacteria				0	0	0	0	0	0	0	0	1	0	0	0	1
Cluster7475;:	Proteobac	Betapr	Rhod	Rhodocyclaceae		0	0	1	0	0	0	0	0	0	0	0	0	1
Cluster7476;:	Acidobact	Acidobacteria_Gp2			Gp2	0	0	1	0	0	0	0	0	0	0	0	0	1
Cluster7479;:	Proteobac	Alphap	Rhizo	Beijerinckiaceae		0	0	0	0	1	0	0	0	0	0	0	0	1

Cluster7482;	Proteobac	Alphap	Rhodospirillales			0	0	0	0	0	0	0	1	0	0	0	0	1
Cluster7486;	Proteobac	Alphap	Rhodospirillales			0	0	0	0	0	0	0	0	1	0	0	0	1
Cluster7501;	Bacteroid	Sphing	Sphir	Chitinop	Sediminibacter	0	0	0	0	0	0	1	0	0	0	0	0	1
Cluster7508;	Proteobac	Alphap	Rhodospirillales			0	0	0	0	0	1	0	0	0	0	0	0	1
Cluster7512;	Proteobac	Alphap	Rhod	Rhodospirillaceae		0	0	0	1	0	0	0	0	0	0	0	0	1
Cluster7516;	Acidobact	Acidobacteria_Gp2			Gp2	0	0	0	0	0	0	1	0	0	0	0	0	1
Cluster7518;	Acidobact	Acidobacteria_Gp1			Gp1	0	0	0	0	0	1	0	0	0	0	0	0	1
Cluster7538;	Verrucom	Subdivision3			Subdivision3_g	0	0	0	0	0	0	0	1	0	0	0	0	1
Cluster7544;	Proteobac	Alphap	Rhizobiales			0	0	0	0	0	0	1	0	0	0	0	0	1
Cluster7547;	Actinobac	Actinob	Actin	Catenul	Catenulispora	0	0	0	0	0	0	0	1	0	0	0	0	1
Cluster7548;	Actinobac	Actinob	Acidimicrobiales			0	0	0	0	0	0	0	0	0	0	1	0	1
Cluster7559;	Proteobac	Alphap	Rhizobiales			0	0	0	0	0	0	0	0	1	0	0	0	1
Cluster7561;	Actinobac	Actinob	Acidi	Acidimi	Aciditerrimona	0	0	0	0	0	0	0	0	1	0	0	0	1
Cluster7571;	Proteobac	Alphap	Caulo	Caulobacteraceae		0	0	0	0	0	0	0	0	0	0	0	1	1
Cluster7573;size=3						0	0	0	0	0	0	0	0	0	0	1	0	1
Cluster7576;	Actinobac	Actinob	Actinomycetales			0	0	0	0	0	0	0	0	0	0	0	1	1
Cluster7578;	Proteobac	Alphap	Rhizobiales			1	0	0	0	0	0	0	0	0	0	0	0	1
Cluster7579;	Acidobact	Acidobacteria_Gp1				0	0	0	0	0	0	0	0	0	0	1	0	1
Cluster7581;	Planctomycetes					0	0	0	0	0	0	1	0	0	0	0	0	1
Cluster7586;	Acidobact	Acidobacteria_Gp1				0	0	0	0	0	0	0	0	0	0	1	0	1
Cluster7601;size=1						1	0	0	0	0	0	0	0	0	0	0	0	1
Cluster7604;	Proteobac	Alphap	Rhodospirillales			0	1	0	0	0	0	0	0	0	0	0	0	1
Cluster7607;	Acidobact	Acidobacteria_Gp7			Gp7	0	0	0	0	0	0	1	0	0	0	0	0	1
Cluster7611;	Proteobac	Alphap	Rhodospirillales			0	0	1	0	0	0	0	0	0	0	0	0	1
Cluster7617;	Proteobac	Alphaproteobacteria				0	0	0	0	0	0	0	0	0	0	1	0	1
Cluster7621;	Actinobac	Actinob	Actin	Sporich	Sporichthya	0	0	0	0	0	0	1	0	0	0	0	0	1
Cluster7639;	Proteobac	Betapr	Burkl	Burkhol	Burkholderia	0	0	0	0	0	0	0	1	0	0	0	0	1
Cluster7642;	Chlamydia	Chlamy	Chlam	Parachla	Parachlamydia	0	0	0	0	0	0	1	0	0	0	0	0	1
Cluster7647;	Acidobact	Acidobacteria_Gp10			Gp10	0	0	0	0	0	1	0	0	0	0	0	0	1

Cluster7648;	Proteobac	Gamm	Xantl	Xanthor	Frateuria	0	0	0	0	0	0	1	0	0	0	0	0	1
Cluster7649;	Proteobac	Alphaproteobacteria				0	0	0	0	1	0	0	0	0	0	0	0	1
Cluster7651;	Proteobac	Alphap	Rhizc	Bradyrh	Agromonas	0	0	0	0	0	0	1	0	0	0	0	0	1
Cluster7655;	Acidobact	Acidobacteria_Gp1				0	0	0	0	0	0	0	0	0	0	1	0	1
Cluster7680;	Proteobac	Alphap	Rhizc	Bradyrhizobiaceae		0	0	0	0	0	0	1	0	0	0	0	0	1
Cluster7682;	Acidobact	Acidobacteria_Gp3			Gp3	0	0	0	1	0	0	0	0	0	0	0	0	1
Cluster7683;	Proteobac	Alphap	Rhizobiales			0	0	0	0	0	0	0	1	0	0	0	0	1
Cluster7686;	Verrucom	Subdivision3			Subdivision3_g	0	0	1	0	0	0	0	0	0	0	0	0	1
Cluster7693;size=1						0	0	0	1	0	0	0	0	0	0	0	0	1
Cluster7700;	Proteobac	Gammaproteobacteria				0	0	0	0	0	0	0	0	0	0	1	0	1
Cluster7706;size=1						0	0	0	0	0	0	0	1	0	0	0	0	1
Cluster7708;	Acidobact	Acidobacteria_Gp2			Gp2	0	0	0	0	0	0	0	1	0	0	0	0	1
Cluster7712;	Proteobac	Alphap	Rhizobiales			0	0	0	0	0	0	0	1	0	0	0	0	1
Cluster7717;	Armatimonadetes				Armatimonade	0	0	1	0	0	0	0	0	0	0	0	0	1
Cluster7729;size=1						0	0	0	0	0	1	0	0	0	0	0	0	1
Cluster7730;	Proteobac	Alphap	Rhodospirillales			0	0	1	0	0	0	0	0	0	0	0	0	1
Cluster7731;	Acidobact	Acidobacteria_Gp1			Acidobacteriur	0	0	0	0	0	0	0	0	0	0	0	1	1
Cluster7737;	Proteobac	Alphap	Rhizc	Xanthol	Pseudolabrys	1	0	0	0	0	0	0	0	0	0	0	0	1
Cluster7738;	Proteobac	Alphap	Rhizobiales			0	0	0	0	0	0	1	0	0	0	0	0	1
Cluster7739;	Proteobac	Alphap	Rhizobiales			0	0	0	0	0	1	0	0	0	0	0	0	1
Cluster7740;	Actinobac	Actinobacteria				0	0	1	0	0	0	0	0	0	0	0	0	1
Cluster7744;	Acidobact	Acidobacteria_Gp2			Gp2	0	1	0	0	0	0	0	0	0	0	0	0	1
Cluster7746;	Proteobac	Alphap	Rhodospirillales			0	1	0	0	0	0	0	0	0	0	0	0	1
Cluster7749;	Proteobac	Alphaproteobacteria				0	0	0	1	0	0	0	0	0	0	0	0	1
Cluster7751;	Proteobac	Alphap	Rhodospirillales			0	0	0	0	0	0	0	1	0	0	0	0	1
Cluster7761;	Acidobact	Acidobacteria_Gp1			Gp1	0	0	0	0	0	0	1	0	0	0	0	0	1
Cluster7764;size=1						0	0	0	0	0	0	0	0	0	0	0	1	1
Cluster7775;	Proteobac	Alphaproteobacteria				0	0	1	0	0	0	0	0	0	0	0	0	1
Cluster7777;	Proteobac	Alphap	Rhodospirillales			0	0	0	0	0	0	1	0	0	0	0	0	1

Cluster7782;s	Proteobac	Alphap	Rhodospirillales			0	0	0	0	0	0	0	1	0	0	0	0	1
Cluster7786;s	Acidobact	Acidobacteria_Gp10			Gp10	0	0	1	0	0	0	0	0	0	0	0	0	1
Cluster7812;size=5						0	0	0	0	0	0	0	0	0	0	1	0	1
Cluster7818;s	Proteobac	Alphap	Rhodospirillales			0	0	0	0	0	0	0	0	0	0	1	0	1
Cluster7819;s	Proteobac	Alphap	Rhizo	Rhizobia	Rhizobium	0	0	0	0	0	0	0	0	1	0	0	0	1
Cluster7824;s	Proteobac	Gammaproteobacteria				0	0	0	0	0	0	0	1	0	0	0	0	1
Cluster7825;s	Acidobact	Acidobacteria_Gp1			Gp1	0	0	0	1	0	0	0	0	0	0	0	0	1
Cluster7827;s	Acidobact	Acidobacteria_Gp2			Gp2	0	0	0	0	0	0	0	0	1	0	0	0	1
Cluster7828;s	Proteobac	Alphap	Rhodospirillales			0	0	0	0	0	0	1	0	0	0	0	0	1
Cluster7831;s	Proteobac	Gamm	Xanth	Sinobac	Steroidobacter	0	0	0	0	0	0	0	0	0	1	0	0	1
Cluster7833;size=5						0	0	0	0	0	0	0	0	1	0	0	0	1
Cluster7843;s	Proteobac	Alphap	Rhizo	Bradyrh	Bradyrhizobium	0	0	1	0	0	0	0	0	0	0	0	0	1
Cluster7852;s	Proteobac	Alphap	Rhodospirillales			0	0	0	1	0	0	0	0	0	0	0	0	1
Cluster7862;s	Proteobac	Alphap	Rhodospirillales			0	0	0	0	0	0	0	0	0	0	1	0	1
Cluster7869;s	Proteobac	Alphap	Rhodospirillales			0	0	0	0	0	1	0	0	0	0	0	0	1
Cluster7875;s	Verrucom	Opituta	Opitu	Opituta	Opitutus	1	0	0	0	0	0	0	0	0	0	0	0	1
Cluster7879;size=5						0	0	0	0	0	0	0	0	0	0	0	1	1
Cluster7882;s	Acidobact	Acidobacteria_Gp1			Gp1	0	0	0	0	0	0	0	0	1	0	0	0	1
Cluster7886;s	Proteobac	Deltap	Myxococcales			0	0	0	0	0	1	0	0	0	0	0	0	1
Cluster7887;s	Proteobac	Alphap	Rhodospirillales			0	0	0	0	0	0	0	0	1	0	0	0	1
Cluster7890;s	Acidobact	Acidobacteria_Gp1			Gp1	0	0	0	0	0	0	0	0	1	0	0	0	1
Cluster7910;size=4						0	0	0	0	0	1	0	0	0	0	0	0	1
Cluster7911;s	Proteobac	Deltap	Bdell	Bdellovi	Vampirovibrio	0	0	0	0	0	0	0	0	1	0	0	0	1
Cluster7926;s	Chloroflex	Ktedor	Ktedonobacterales			0	0	0	0	0	0	0	1	0	0	0	0	1
Cluster7931;s	Acidobact	Acidobacteria_Gp2			Gp2	0	0	0	0	0	1	0	0	0	0	0	0	1
Cluster7936;s	Proteobac	Deltap	Bdell	Bdellovi	Vampirovibrio	0	0	1	0	0	0	0	0	0	0	0	0	1
Cluster7939;s	Acidobact	Acidobacteria_Gp6			Gp6	0	0	0	0	0	0	0	0	0	0	0	1	1
Cluster7944;s	Acidobact	Acidobacteria_Gp3			Gp3	0	0	0	0	0	0	1	0	0	0	0	0	1
Cluster7945;s	Proteobac	Alphap	Rhizobiales			0	0	1	0	0	0	0	0	0	0	0	0	1

Cluster7947;	Armatimo	Chthor	Chthc	Chthon	Chthonomona	0	0	0	0	0	1	0	0	0	0	0	0	1
Cluster7949;	Proteobac	Deltaproteobacteria				0	0	0	0	0	0	0	0	1	0	0	0	1
Cluster7952;	Acidobact	Acidobacteria_Gp1			Gp1	0	0	1	0	0	0	0	0	0	0	0	0	1
Cluster7956;size=2						0	0	0	1	0	0	0	0	0	0	0	0	1
Cluster7960;	Proteobac	Deltap	Myxococcales			0	0	0	0	0	1	0	0	0	0	0	0	1
Cluster7961;	Proteobac	Betapr	Burkl	Oxaloba	Janthinobacter	0	0	0	0	0	1	0	0	0	0	0	0	1
Cluster7962;	Proteobac	Alphap	Rhizc	Bradyrhizobiaceae		0	0	0	0	0	1	0	0	0	0	0	0	1
Cluster7979;	Proteobac	Alphap	Rhodospirillales			0	0	0	0	0	0	0	0	0	0	1	0	1
Cluster7982;	Proteobac	Alphap	Rhod	Acetobacteraceae		0	0	0	0	0	0	0	0	0	0	1	0	1
Cluster7983;	Acidobact	Acidobacteria_Gp3			Gp3	0	0	0	0	0	0	0	0	0	0	1	0	1
Cluster7986;	Proteobac	Gammaproteobacteria				0	0	0	0	0	0	0	0	0	0	1	0	1
Cluster7987;	Acidobact	Acidobacteria_Gp2			Gp2	0	0	0	1	0	0	0	0	0	0	0	0	1
Cluster7988;	Proteobac	Alphap	Rhizc	Bradyrhizobiaceae		0	0	0	0	0	0	0	0	1	0	0	0	1
Cluster7990;	Proteobac	Deltap	Myxc	Cystobacteraceae		0	0	0	0	0	0	0	0	0	0	1	0	1
Cluster7991;	Verrucom	Subdivision3			Subdivision3_g	0	0	0	1	0	0	0	0	0	0	0	0	1
Cluster7992;	Verrucom	Opitut	Opitu	Opituta	Opitutus	0	0	0	0	0	0	0	0	0	0	1	0	1
Cluster7994;	Proteobac	Betapr	Burkholderiales			0	0	0	1	0	0	0	0	0	0	0	0	1
Cluster7995;	Proteobac	Deltap	Myxococcales			0	0	1	0	0	0	0	0	0	0	0	0	1
Cluster8018;	Acidobact	Acidobacteria_Gp3				0	0	0	1	0	0	0	0	0	0	0	0	1
Cluster8026;	Proteobac	Deltap	Bdell	Bdellovi	Vampirovibrio	0	0	0	0	0	0	0	0	0	0	1	0	1
Cluster8031;	Acidobact	Acidobacteria_Gp3			Gp3	0	1	0	0	0	0	0	0	0	0	0	0	1
Cluster8038;	Actinobac	Actinol	Actin	Actinos	Actinospica	0	0	0	0	0	0	0	0	0	0	0	1	1
Cluster8040;	Acidobact	Acidobacteria_Gp1				0	0	0	1	0	0	0	0	0	0	0	0	1
Cluster8045;	Proteobac	Betapr	Burkl	Burkhol	Burkholderia	0	0	0	1	0	0	0	0	0	0	0	0	1
Cluster8049;	Planctomycetes					0	0	0	0	1	0	0	0	0	0	0	0	1
Cluster8051;size=8						0	0	0	0	0	0	1	0	0	0	0	0	1
Cluster8058;	Actinobac	Actinol	Actin	Actinos	Actinospica	0	0	0	1	0	0	0	0	0	0	0	0	1
Cluster8060;	Verrucom	Spartobacteria			Spartobacteria	0	0	1	0	0	0	0	0	0	0	0	0	1
Cluster8062;	Planctomy	Plancto	Planc	Plancto	Planctomyces	0	0	0	0	0	0	0	0	0	0	1	0	1

Cluster8070;	Acidobact	Acidobacteria_Gp1				0	0	0	1	0	0	0	0	0	0	0	0	1
Cluster8103;	Armatimc	Armati	Arma	Armatir	Armatimonas/	0	0	0	0	0	0	1	0	0	0	0	0	1
Cluster8105;size=2						0	0	0	0	0	0	0	0	0	0	1	0	1
Cluster8110;	Acidobact	Acidobacteria_Gp2			Gp2	0	0	0	1	0	0	0	0	0	0	0	0	1
Cluster8111;	Proteobac	Alphap	Rhizc	Bradyrh	Bradyrhizobiur	0	0	0	0	0	0	1	0	0	0	0	0	1
Cluster8114;size=1						0	0	0	0	0	0	0	0	0	0	1	0	1
Cluster8116;	Proteobac	Alphap	Rhodospirillales			0	0	0	1	0	0	0	0	0	0	0	0	1
Cluster8118;	Acidobact	Acidobacteria_Gp2			Gp2	0	0	0	1	0	0	0	0	0	0	0	0	1
Cluster8128;	Acidobact	Acidobacteria_Gp3				0	0	0	0	0	0	0	0	0	0	1	0	1
Cluster8138;	Proteobac	Alphap	Rhizc	Bradyrhizobiaceae		0	0	1	0	0	0	0	0	0	0	0	0	1
Cluster8139;size=1						0	0	0	0	1	0	0	0	0	0	0	0	1
Cluster8140;	Acidobact	Acidobacteria_Gp3			Gp3	0	0	0	0	0	0	0	0	0	1	0	0	1
Cluster8141;	Acidobact	Acidobacteria_Gp5			Gp5	0	0	0	0	0	0	0	0	0	0	1	0	1
Cluster8142;	Verrucom	Opitut	Opitu	Opituta	Opitutus	0	0	0	0	0	0	0	0	0	1	0	0	1
Cluster8158;	Acidobact	Acidobacteria_Gp2			Gp2	0	0	0	0	0	0	0	0	1	0	0	0	1
Cluster8159;	Bacteroid	Sphing	Sphir	Sphingc	Mucilaginibact	0	0	0	0	1	0	0	0	0	0	0	0	1
Cluster8163;	Proteobac	Alphap	Rhodospirillales			0	0	0	0	1	0	0	0	0	0	0	0	1
Cluster8164;	Proteobacteria					0	0	0	0	1	0	0	0	0	0	0	0	1
Cluster8166;	Proteobac	Alphaproteobacteria				1	0	0	0	0	0	0	0	0	0	0	0	1
Cluster8169;	Acidobact	Acidobacteria_Gp1				0	0	0	0	1	0	0	0	0	0	0	0	1
Cluster8172;size=1						0	0	1	0	0	0	0	0	0	0	0	0	1
Cluster8173;	Proteobac	Alphap	Rhizc	Bradyrhizobiaceae		0	0	0	1	0	0	0	0	0	0	0	0	1
Cluster8175;	Verrucom	Spartobacteria			Spartobacteria	0	0	0	0	1	0	0	0	0	0	0	0	1
Cluster8181;size=4						0	0	0	0	0	0	0	0	1	0	0	0	1
Cluster8190;	Proteobac	Betapr	Burkl	Burkhol	Burkholderia	0	0	0	0	1	0	0	0	0	0	0	0	1
Cluster8193;	Acidobact	Acidobacteria_Gp3			Gp3	0	0	0	0	0	0	0	0	0	0	1	0	1
Cluster8196;	Acidobact	Acidobacteria_Gp1			Granulicella	0	0	0	0	1	0	0	0	0	0	0	0	1
Cluster8197;	Acidobacteria					0	0	0	0	1	0	0	0	0	0	0	0	1
Cluster8199;	Acidobact	Acidobacteria_Gp2			Gp2	0	0	0	0	0	1	0	0	0	0	0	0	1

Cluster8201;s	Proteobac	Deltap	Myxococcales			0	0	0	0	1	0	0	0	0	0	0	0	1
Cluster8214;s	Proteobac	Alphap	Alphaproteob		Rhizomicrobiu	0	0	0	0	0	1	0	0	0	0	0	0	1
Cluster8221;s	Acidobact	Acidobacteria_Gp1				0	0	0	1	0	0	0	0	0	0	0	0	1
Cluster8223;size=2						0	0	0	0	0	0	0	0	0	0	1	0	1
Cluster8229;size=1						0	0	0	0	1	0	0	0	0	0	0	0	1
Cluster8232;s	Proteobacteria					0	0	0	0	0	0	0	0	0	0	1	0	1
Cluster8242;s	Gemmatir	Gemm	Gemi	Gemma	Gemmatimona	0	0	0	0	0	0	0	0	0	0	1	0	1
Cluster8243;s	Proteobac	Alphap	Rhodospirillales			0	0	0	0	1	0	0	0	0	0	0	0	1
Cluster8246;s	Proteobac	Betapr	Burkl	Burkhol	Burkholderia	0	0	0	0	0	0	0	1	0	0	0	0	1
Cluster8255;size=8						0	0	0	1	0	0	0	0	0	0	0	0	1
Cluster8256;s	Acidobact	Acidobacteria_Gp1			Edaphobacter	0	0	0	1	0	0	0	0	0	0	0	0	1
Cluster8263;s	Actinobac	Actinol	Actinomycetales			0	0	0	1	0	0	0	0	0	0	0	0	1
Cluster8273;s	Actinobac	Actinol	Acidimicrobiales			0	0	0	1	0	0	0	0	0	0	0	0	1
Cluster8275;s	Proteobac	Deltap	Myxococcales			0	0	0	0	0	0	0	0	0	0	1	0	1
Cluster8280;size=4						0	0	0	0	0	0	0	0	0	0	0	1	1
Cluster8283;size=1						0	0	0	1	0	0	0	0	0	0	0	0	1
Cluster8285;size=3						0	0	0	0	0	0	0	0	0	0	1	0	1
Cluster8292;s	Proteobac	Alphap	Rhod	Acetobacteraceae		0	0	0	1	0	0	0	0	0	0	0	0	1
Cluster8304;s	Actinobac	Actinol	Acidimicrobiales			0	0	0	0	0	0	0	0	0	0	0	1	1
Cluster8320;s	Acidobact	Acidobacteria_Gp1			Gp1	0	0	1	0	0	0	0	0	0	0	0	0	1
Cluster8323;s	Proteobac	Alphap	Rhodospirillales			0	0	0	1	0	0	0	0	0	0	0	0	1
Cluster8327;s	Proteobac	Alphap	Rhodospirillales			0	0	0	1	0	0	0	0	0	0	0	0	1
Cluster8342;size=3						0	0	0	0	0	0	0	0	0	0	1	0	1
Cluster8343;s	Armatimonadetes				Armatimonade	0	0	0	0	1	0	0	0	0	0	0	0	1
Cluster8351;s	Proteobac	Alphap	Rhodospirillales			0	0	0	1	0	0	0	0	0	0	0	0	1
Cluster8352;s	Actinobac	Actinol	Actin	Nocardi	Marmoricola	0	0	0	1	0	0	0	0	0	0	0	0	1
Cluster8355;s	Proteobac	Alphaproteobacteria				0	0	0	0	0	0	0	0	0	0	1	0	1
Cluster8357;size=1						0	0	0	1	0	0	0	0	0	0	0	0	1
Cluster8360;s	Acidobact	Acidobacteria_Gp2			Gp2	0	0	0	1	0	0	0	0	0	0	0	0	1

Cluster8361;:	Proteobac	Deltap	Myxc	Cystobacteraceae		0	0	0	1	0	0	0	0	0	0	0	0	1
Cluster8363;:	Proteobac	Gamm	Gammaproteobacteria_ince			0	0	0	1	0	0	0	0	0	0	0	0	1
Cluster8365;:	Verrucom	Subdivision3			Subdivision3_g	0	0	0	1	0	0	0	0	0	0	0	0	1
Cluster8367;:	Acidobacteria					0	0	0	1	0	0	0	0	0	0	0	0	1
Cluster8371;size=5						0	0	1	0	0	0	0	0	0	0	0	0	1
Cluster8372;:	Proteobac	Gammaproteobacteria				0	0	0	0	0	0	0	0	0	0	1	0	1
Cluster8379;:	Proteobac	Alphaproteobacteria				0	0	1	0	0	0	0	0	0	0	0	0	1
Cluster8382;:	Verrucom	Spartobacteria			Spartobacteria	0	0	0	1	0	0	0	0	0	0	0	0	1
Cluster8390;:	Proteobac	Gamm	Legio	Coxiella	Aquicella	0	0	0	0	0	0	0	0	0	0	1	0	1
Cluster8396;:	Proteobac	Alphap	Rhodospirillales			0	0	0	1	0	0	0	0	0	0	0	0	1
Cluster8399;:	Nitrospira	Nitrosp	Nitro	Nitrospi	Nitrospira	0	0	0	0	0	0	0	0	0	0	1	0	1
Cluster8409;:	Proteobac	Alphap	Rhodospirillales			0	0	0	0	0	0	0	0	0	0	1	0	1
Cluster8412;:	Proteobac	Alphap	Rhod	Acetobacteraceae		0	0	0	1	0	0	0	0	0	0	0	0	1
Cluster8413;:	Acidobact	Acidobacteria_Gp6			Gp6	0	0	0	0	0	0	0	1	0	0	0	0	1
Cluster8418;:	Proteobac	Deltaproteobacteria				0	1	0	0	0	0	0	0	0	0	0	0	1
Cluster8421;:	OD1				OD1_genera_i	0	0	0	0	0	1	0	0	0	0	0	0	1
Cluster8422;:	Proteobac	Alphap	Rhodospirillales			0	0	0	1	0	0	0	0	0	0	0	0	1
Cluster8423;:	Proteobac	Alphap	Sphir	Sphingomonadaceae		0	0	0	0	0	0	0	1	0	0	0	0	1
Cluster8424;:	Verrucom	Subdivision3			Subdivision3_g	0	0	0	1	0	0	0	0	0	0	0	0	1
Cluster8425;:	Proteobac	Deltap	Myxococcales			0	0	0	0	0	0	0	0	0	0	1	0	1
Cluster8426;:	Proteobac	Alphap	Rhizobiales			0	0	0	0	0	0	1	0	0	0	0	0	1
Cluster8429;:	Proteobac	Alphaproteobacteria				0	0	0	0	0	0	0	0	0	0	1	0	1
Cluster8430;size=1						0	0	0	0	0	0	0	0	0	0	1	0	1
Cluster8435;:	Proteobac	Gamm	Xantl	Sinobac	Steroidobacter	0	0	0	1	0	0	0	0	0	0	0	0	1
Cluster8439;:	Proteobac	Alphaproteobacteria				0	0	0	1	0	0	0	0	0	0	0	0	1
Cluster8441;:	Proteobac	Alphap	Rhizobiales			0	0	0	0	0	1	0	0	0	0	0	0	1
Cluster8442;:	Actinobac	Actinol	Actinomycetales			0	0	0	1	0	0	0	0	0	0	0	0	1
Cluster8443;size=1						0	0	0	0	0	0	0	0	0	0	1	0	1
Cluster8449;:	Proteobac	Alphap	Rhizobiales			0	0	0	0	0	0	0	0	0	0	0	1	1

Cluster8458;	Acidobact	Acidobacteria_Gp2			Gp2	0	0	0	1	0	0	0	0	0	0	0	0	1
Cluster8467;	Acidobact	Acidobacteria_Gp2			Gp2	0	0	0	0	0	0	1	0	0	0	0	0	1
Cluster8474;	Acidobact	Acidobacteria_Gp2			Gp2	0	0	0	0	0	0	0	0	0	0	1	0	1
Cluster8482;	Acidobact	Acidobacteria_Gp2			Gp2	0	0	0	1	0	0	0	0	0	0	0	0	1
Cluster8485;size=3						0	0	0	1	0	0	0	0	0	0	0	0	1
Cluster8489;	Actinobac	Actinol	Acidi	Acidimi	Aciditerrimona	1	0	0	0	0	0	0	0	0	0	0	0	1
Cluster8496;	Proteobac	Alphap	Rhod	Acetoba	Acidocella	0	0	0	0	0	0	0	0	0	0	1	0	1
Cluster8503;	Acidobact	Acidobacteria_Gp2			Gp2	0	0	0	1	0	0	0	0	0	0	0	0	1
Cluster8505;	Proteobac	Alphap	Rhodospirillales			0	0	0	0	0	1	0	0	0	0	0	0	1
Cluster8506;	Verrucom	Opituta	Opitu	Opituta	Opitutus	0	0	0	0	0	0	0	0	0	0	1	0	1
Cluster8509;	Acidobact	Acidobacteria_Gp2			Gp2	0	0	0	1	0	0	0	0	0	0	0	0	1
Cluster8519;	Proteobac	Alphap	Rhodospirillales			0	0	0	1	0	0	0	0	0	0	0	0	1
Cluster8529;	Proteobac	Betapr	Burkl	Burkhol	Burkholderia	0	0	0	1	0	0	0	0	0	0	0	0	1
Cluster8530;	Proteobac	Alphap	Rhizo	Hyphon	Rhodoplanes	0	0	0	1	0	0	0	0	0	0	0	0	1
Cluster8531;size=1						0	0	0	0	0	0	0	0	0	0	1	0	1
Cluster8537;	Planctomy	Plancto	Planc	Planctor	Singulisphaera	0	0	0	1	0	0	0	0	0	0	0	0	1
Cluster8538;	Acidobact	Acidobacteria_Gp3			Gp3	0	0	0	1	0	0	0	0	0	0	0	0	1
Cluster8539;	Proteobac	Deltap	Myxococcales			0	0	0	1	0	0	0	0	0	0	0	0	1
Cluster8545;	Proteobac	Gamm	Xantl	Sinobac	Steroidobacter	0	0	0	0	0	0	0	1	0	0	0	0	1
Cluster8550;	Acidobact	Acidobacteria_Gp1			Gp1	0	0	0	0	1	0	0	0	0	0	0	0	1
Cluster8551;	Proteobac	Alphap	Rhizo	Bradyrh	Agromonas	0	0	0	1	0	0	0	0	0	0	0	0	1
Cluster8564;	Acidobact	Acidobacteria_Gp2			Gp2	0	0	1	0	0	0	0	0	0	0	0	0	1
Cluster8570;	Proteobac	Betapr	Burkl	Burkhol	Burkholderia	1	0	0	0	0	0	0	0	0	0	0	0	1
Cluster8571;	Proteobacteria					0	0	0	0	0	1	0	0	0	0	0	0	1
Cluster8575;	Acidobact	Acidobacteria_Gp1				0	0	0	0	0	0	0	0	0	0	1	0	1
Cluster8585;	Proteobac	Alphap	Rhodospirillales			0	0	1	0	0	0	0	0	0	0	0	0	1
Cluster8602;	Elusimicro	Elusim	Elusi	Elusimic	Elusimicrobiun	0	0	0	0	0	0	0	0	0	0	1	0	1
Cluster8605;size=2						0	1	0	0	0	0	0	0	0	0	0	0	1
Cluster8608;	Proteobac	Gamm	Xantl	Sinobac	Steroidobacter	0	0	0	0	0	1	0	0	0	0	0	0	1

Cluster8609;:	Proteobac	Alphap	Rhizobiales			0	0	0	1	0	0	0	0	0	0	0	0	1
Cluster8620;size=1						0	0	0	0	0	1	0	0	0	0	0	0	1
Cluster8622;:	Verrucom	Opitut	Opitu	Opituta	Opitutus	0	0	0	0	0	0	0	0	0	1	0	0	1
Cluster8624;:	Bacteroid	Sphing	Sphir	Chitinop	Flavitalea	0	0	0	0	0	0	0	0	0	0	1	0	1
Cluster8627;:	Proteobac	Alphap	Rhizo	Bradyrhizobiaceae		0	0	0	0	0	0	0	0	0	0	1	0	1
Cluster8638;:	Proteobac	Alphap	Rhodospirillales			0	0	0	0	0	0	0	0	0	0	1	0	1
Cluster8639;:	Acidobact	Acidobacteria_Gp1				0	0	0	0	0	1	0	0	0	0	0	0	1
Cluster8642;:	Proteobac	Alphaproteobacteria				0	0	0	0	0	0	0	1	0	0	0	0	1
Cluster8643;:	Proteobac	Betapr	Burkl	Oxalobacteraceae		0	0	0	0	0	1	0	0	0	0	0	0	1
Cluster8652;:	Chloroflex	Ktedor	Ktedonobacterales			0	0	0	0	0	0	0	1	0	0	0	0	1
Cluster8656;:	Proteobac	Alphap	Rhodospirillales			0	0	0	0	0	0	0	0	0	0	0	1	1
Cluster8664;:	Proteobac	Deltap	Myxococcales			0	0	1	0	0	0	0	0	0	0	0	0	1
Cluster8671;:	Proteobac	Gammaproteobacteria				0	0	1	0	0	0	0	0	0	0	0	0	1
Cluster8674;:	Proteobac	Alphap	Rhod	Rhodospirillaceae		0	0	0	0	1	0	0	0	0	0	0	0	1
Cluster8692;:	Proteobac	Alphap	Rhodospirillales			0	0	0	0	0	1	0	0	0	0	0	0	1
Cluster8694;:	Acidobact	Acidobacteria_Gp1				0	0	0	0	0	1	0	0	0	0	0	0	1
Cluster8704;:	Actinobac	Actinol	Actinomycetales			0	0	0	0	0	0	0	0	0	0	0	1	1
Cluster8723;size=2						0	0	0	0	0	1	0	0	0	0	0	0	1
Cluster8724;:	Proteobac	Alphap	Rhizo	Bradyrhizobiaceae		0	0	0	0	0	0	0	0	0	1	0	0	1
Cluster8725;:	Acidobact	Acidobacteria_Gp3			Bryobacter	0	0	0	0	1	0	0	0	0	0	0	0	1
Cluster8728;:	Verrucom	Subdivision3			Subdivision3_g	0	0	0	0	1	0	0	0	0	0	0	0	1
Cluster8730;:	Acidobact	Acidobacteria_Gp2			Gp2	0	0	0	0	0	0	0	0	0	0	1	0	1
Cluster8735;:	Acidobact	Acidobacteria_Gp1				0	0	0	0	0	0	0	0	0	1	0	0	1
Cluster8740;:	Proteobac	Gamm	Xantl	Sinobac	Steroidobacter	0	0	0	0	0	1	0	0	0	0	0	0	1
Cluster8741;:	Proteobac	Alphap	Rhodospirillales			0	0	0	0	0	0	0	0	0	1	0	0	1
Cluster8743;size=1						0	0	0	0	1	0	0	0	0	0	0	0	1
Cluster8746;:	Acidobact	Acidobacteria_Gp2			Gp2	0	0	0	0	1	0	0	0	0	0	0	0	1
Cluster8752;:	Proteobac	Alphap	Rhizo	Bradyrh	Agromonas	0	0	0	0	0	0	0	0	0	0	1	0	1
Cluster8758;:	Actinobac	Actinol	Actinomycetales			0	0	0	0	1	0	0	0	0	0	0	0	1

Cluster8762;	Verrucom	Spartobacteria			Spartobacteria	0	0	1	0	0	0	0	0	0	0	0	0	1
Cluster8769;	Actinobac	Actinol	Actinomycetales			0	0	0	0	0	0	0	0	0	0	1	0	1
Cluster8779;	Acidobact	Acidobacteria_Gp3			Gp3	0	0	0	0	0	0	0	0	0	0	1	0	1
Cluster8789;	Proteobac	Alphap	Rhizc	Xanthol	Pseudolabrys	0	0	0	0	1	0	0	0	0	0	0	0	1
Cluster8791;	Proteobac	Alphaproteobacteria				0	0	0	0	1	0	0	0	0	0	0	0	1
Cluster8800;	Proteobac	Alphaproteobacteria				0	0	0	0	0	0	0	0	0	1	0	0	1
Cluster8802;	Proteobac	Alphap	Rhodospirillales			0	0	0	0	0	0	0	0	0	0	1	0	1
Cluster8805;	Proteobac	Alphap	Rhod	Rhodospirillaceae		0	0	0	0	1	0	0	0	0	0	0	0	1
Cluster8806;	Acidobact	Acidobacteria_Gp3			Gp3	0	0	0	0	0	0	0	0	0	1	0	0	1
Cluster8807;	Proteobac	Alphap	Rhizobiales			0	0	0	0	0	0	0	0	0	1	0	0	1
Cluster8814;	Proteobac	Gamm	Xanthomonadales			0	0	0	0	0	0	0	0	0	1	0	0	1
Cluster8823;	Acidobact	Acidobacteria_Gp3			Gp3	0	0	0	0	1	0	0	0	0	0	0	0	1
Cluster8826;	Acidobact	Acidobacteria_Gp3				0	0	0	0	1	0	0	0	0	0	0	0	1
Cluster8830;	Chlamydia	Chlamy	Chlar	Parachla	Neochlamydia	0	0	0	0	1	0	0	0	0	0	0	0	1
Cluster8832;size=1						0	0	0	0	0	0	0	0	0	0	1	0	1
Cluster8835;	Verrucom	Subdivision3			Subdivision3_g	1	0	0	0	0	0	0	0	0	0	0	0	1
Cluster8839;	Proteobac	Alphaproteobacteria				1	0	0	0	0	0	0	0	0	0	0	0	1
Cluster8846;	Verrucom	Opitut	Opitu	Opituta	Opitutus	0	0	0	0	0	1	0	0	0	0	0	0	1
Cluster8866;	Acidobact	Acidobacteria_Gp3			Gp3	0	0	1	0	0	0	0	0	0	0	0	0	1
Cluster8872;	Acidobact	Acidobacteria_Gp1				0	0	0	0	0	0	0	0	1	0	0	0	1
Cluster8878;	Proteobac	Alphaproteobacteria				0	0	0	0	0	0	0	1	0	0	0	0	1
Cluster8879;	Acidobacteria					0	0	0	0	0	0	0	1	0	0	0	0	1
Cluster8885;	Acidobact	Acidobacteria_Gp1			Gp1	0	0	0	0	0	1	0	0	0	0	0	0	1
Cluster8891;	Proteobac	Deltaproteobacteria				0	0	0	0	1	0	0	0	0	0	0	0	1
Cluster8892;	Acidobact	Acidobacteria_Gp1				0	0	0	0	0	0	0	0	0	1	0	0	1
Cluster8895;	Acidobact	Acidobacteria_Gp1				1	0	0	0	0	0	0	0	0	0	0	0	1
Cluster8909;	Acidobact	Acidobacteria_Gp1			Granulicella	0	0	1	0	0	0	0	0	0	0	0	0	1
Cluster8914;	Proteobac	Gamm	Xantl	Xanthomonadaceae		0	0	0	0	0	0	0	0	0	0	1	0	1
Cluster8918;	Acidobact	Acidobacteria_Gp3				0	0	0	0	0	0	0	0	0	0	1	0	1

Cluster8926;	Proteobac	Alphap	Rhizo	Methylocystaceae		0	0	1	0	0	0	0	0	0	0	0	0	1
Cluster8927;	Verrucom	Subdivision3			Subdivision3_g	1	0	0	0	0	0	0	0	0	0	0	0	1
Cluster8933;size=1						0	0	0	0	0	0	0	0	0	0	0	1	1
Cluster8936;	Acidobact	Acidobacteria_Gp1			Gp1	0	0	0	0	0	0	1	0	0	0	0	0	1
Cluster8948;	Acidobact	Acidobacteria_Gp1				0	0	0	0	0	0	0	0	0	0	0	1	1
Cluster8965;	Acidobacteria					0	0	0	0	0	0	0	0	0	0	0	1	1
Cluster8976;	Acidobacteria					0	0	0	0	1	0	0	0	0	0	0	0	1
Cluster8977;	Proteobac	Alphap	Rhodospirillales			0	0	1	0	0	0	0	0	0	0	0	0	1
Cluster8992;size=1						0	0	0	0	0	0	0	1	0	0	0	0	1
Cluster9001;	Proteobac	Deltaproteobacteria				0	0	0	0	0	0	1	0	0	0	0	0	1
Cluster9005;	Verrucom	Spartobacteria			Spartobacteria	0	0	0	0	0	0	1	0	0	0	0	0	1
Cluster9006;	Planctomy	Plancto	Planc	Plancto	Singulisphaera	0	0	0	0	0	0	0	1	0	0	0	0	1
Cluster9012;	Proteobac	Alphap	Rhizo	Bradyrhizobiaceae		0	0	0	0	0	0	0	0	0	0	0	1	1
Cluster9020;	Acidobact	Acidobacteria_Gp5			Gp5	0	0	0	0	0	0	1	0	0	0	0	0	1
Cluster9026;	Acidobacteria					1	0	0	0	0	0	0	0	0	0	0	0	1
Cluster9028;size=1						0	0	0	0	0	0	0	1	0	0	0	0	1
Cluster9040;	Proteobac	Alphap	Rhodospirillales			0	0	0	0	0	0	0	0	1	0	0	0	1
Cluster9042;	Proteobac	Betaproteobacteria				0	0	0	0	0	0	0	1	0	0	0	0	1
Cluster9043;	Verrucom	Spartobacteria			Spartobacteria	0	0	0	0	0	0	0	0	0	0	0	1	1
Cluster9047;	Acidobact	Acidobacteria_Gp1			Gp1	0	0	1	0	0	0	0	0	0	0	0	0	1
Cluster9056;	Actinobac	Actinol	Soliru	Conexib	Conexibacter	0	0	0	0	0	0	0	0	0	0	0	1	1
Cluster9057;size=5						0	0	0	0	0	0	1	0	0	0	0	0	1
Cluster9058;	Proteobac	Alphap	Rhod	Rhodospirillaceae		0	0	0	0	0	0	0	0	0	0	0	1	1
Cluster9060;	Proteobac	Alphap	Rhodospirillales			0	0	0	0	0	0	0	1	0	0	0	0	1
Cluster9061;	Acidobact	Acidobacteria_Gp1				0	1	0	0	0	0	0	0	0	0	0	0	1
Cluster9067;	Verrucom	Spartobacteria			Spartobacteria	0	0	0	0	0	0	1	0	0	0	0	0	1
Cluster9069;	Acidobact	Acidobacteria_Gp3				0	0	0	0	0	0	0	1	0	0	0	0	1
Cluster9072;	Nitrospira	Nitrosp	Nitro	Nitrospi	Nitrospira	0	0	0	0	0	0	0	0	1	0	0	0	1
Cluster9073;size=3						0	0	0	0	0	0	1	0	0	0	0	0	1

Cluster9076;	Proteobac	Alphaproteobacteria				0	0	0	0	0	0	0	0	0	0	0	1	1
Cluster9078;	Proteobacteria					0	0	0	0	0	0	0	1	0	0	0	0	1
Cluster9081;	Verrucom	Spartobacteria			Spartobacteria	0	0	1	0	0	0	0	0	0	0	0	0	1
Cluster9087;	Acidobact	Acidobacteria_Gp1			Gp1	0	0	0	0	0	0	0	0	0	0	0	1	1
Cluster9088;	Proteobac	Alphap	Rhizobiales			0	0	0	1	0	0	0	0	0	0	0	0	1
Cluster9089;size=2						0	0	0	0	0	0	0	0	1	0	0	0	1
Cluster9091;	Proteobac	Gammaproteobacteria				0	0	0	0	0	0	1	0	0	0	0	0	1
Cluster9095;	Proteobac	Alphap	Rhizc	Bradyrhizobiaceae		0	0	0	0	0	0	1	0	0	0	0	0	1
Cluster9113;	Proteobac	Alphap	Rhod	Rhodos	Magnetospirill	0	0	0	0	0	0	0	1	0	0	0	0	1
Cluster9114;	Acidobact	Acidobacteria_Gp3			Gp3	0	0	1	0	0	0	0	0	0	0	0	0	1
Cluster9127;	Proteobac	Alphaproteobacteria				0	0	0	0	0	0	0	0	0	0	0	1	1
Cluster9132;	Proteobac	Alphap	Rhizc	Xanthol	Pseudolabrys	0	1	0	0	0	0	0	0	0	0	0	0	1
Cluster9138;	Proteobac	Alphap	Rhod	Acetoba	Stella	0	0	0	0	0	0	0	1	0	0	0	0	1
Cluster9140;	Proteobac	Alphap	Rhizc	Beijerin	Methylocella	0	0	0	0	0	1	0	0	0	0	0	0	1
Cluster9141;	Proteobacteria					0	0	0	0	0	0	1	0	0	0	0	0	1
Cluster9144;size=2						0	0	1	0	0	0	0	0	0	0	0	0	1
Cluster9152;size=5						1	0	0	0	0	0	0	0	0	0	0	0	1
Cluster9155;	Proteobac	Gamm	Xantl	Sinobac	Steroidobacte	0	0	0	0	0	0	0	0	0	0	0	1	1
Cluster9157;size=2						0	0	0	0	0	0	0	0	0	0	0	1	1
Cluster9161;	Proteobac	Alphap	Rhodospirillales			0	0	0	0	0	0	0	0	0	0	0	1	1
Cluster9165;	Proteobac	Alphap	Rhizc	Beijerinckiaceae		0	0	0	0	0	0	0	0	0	0	0	1	1
Cluster9168;	Proteobac	Alphap	Rhizc	Bradyrhizobiaceae		0	1	0	0	0	0	0	0	0	0	0	0	1
Cluster9170;	Verrucom	Subdivision3			Subdivision3_	0	0	0	0	0	0	1	0	0	0	0	0	1
Cluster9177;	Acidobact	Acidobacteria_Gp2			Gp2	0	0	0	0	0	0	0	1	0	0	0	0	1
Cluster9181;	Proteobac	Alphap	Rhizc	Bradyrh	Agromonas	0	0	0	0	0	0	1	0	0	0	0	0	1
Cluster9182;	Acidobact	Acidobacteria_Gp2			Gp2	0	0	0	0	0	1	0	0	0	0	0	0	1
Cluster9183;	Acidobact	Acidobacteria_Gp3			Gp3	0	0	1	0	0	0	0	0	0	0	0	0	1
Cluster9184;size=2						0	0	0	0	0	0	0	0	1	0	0	0	1
Cluster9198;	Proteobac	Alphap	Rhizc	Bradyrhizobiaceae		1	0	0	0	0	0	0	0	0	0	0	0	1

Cluster9202;:	Acidobact	Acidobacteria_Gp3			Gp3	0	0	0	0	0	0	1	0	0	0	0	0	1
Cluster9210;:	Verrucom	Subdivision3			Subdivision3_g	1	0	0	0	0	0	0	0	0	0	0	0	1
Cluster9216;size=3						0	0	0	1	0	0	0	0	0	0	0	0	1
Cluster9222;size=5						0	0	0	0	0	0	0	0	0	0	0	1	1
Cluster9237;:	Acidobact	Acidobacteria_Gp1				0	0	0	0	0	0	0	0	0	0	0	1	1
Cluster9238;:	Proteobac	Betapr	Burkholderiales			0	0	0	0	0	0	0	1	0	0	0	0	1
Cluster9245;size=1						0	1	0	0	0	0	0	0	0	0	0	0	1
Cluster9248;size=2						0	0	0	0	0	0	1	0	0	0	0	0	1
Cluster9262;size=1						0	0	0	0	0	0	1	0	0	0	0	0	1
Cluster9272;size=2						0	0	0	0	0	1	0	0	0	0	0	0	1
Cluster9285;:	Proteobac	Alphap	Rhod	Rhodospirillaceae		0	1	0	0	0	0	0	0	0	0	0	0	1
Cluster9288;:	Proteobacteria					0	1	0	0	0	0	0	0	0	0	0	0	1
Cluster9291;:	Acidobact	Acidobacteria_Gp2			Gp2	0	0	0	0	0	0	1	0	0	0	0	0	1
Cluster9292;:	Proteobac	Alphaproteobacteria				0	0	0	0	0	0	0	0	0	0	0	1	1
Cluster9295;:	Proteobac	Alphap	Rhizobiales			0	1	0	0	0	0	0	0	0	0	0	0	1
Cluster9298;:	Proteobac	Alphap	Rhodospirillales			0	0	0	0	0	0	0	0	0	1	0	0	1
Cluster9301;:	Acidobact	Acidobacteria_Gp1			Gp1	0	0	0	1	0	0	0	0	0	0	0	0	1
Cluster9306;:	Proteobac	Alphap	Rhod	Rhodospirillaceae		0	1	0	0	0	0	0	0	0	0	0	0	1
Cluster9308;size=1						0	0	0	0	0	0	0	1	0	0	0	0	1
Cluster9316;:	Bacteroid	Sphing	Sphir	Chitinophagaceae		0	0	0	0	0	0	1	0	0	0	0	0	1
Cluster9317;:	Proteobac	Alphap	Rhizobiales			0	0	0	0	0	0	1	0	0	0	0	0	1
Cluster9324;:	Verrucom	Opitut	Opitu	Opituta	Opitutus	0	1	0	0	0	0	0	0	0	0	0	0	1
Cluster9326;:	Acidobact	Acidobacteria_Gp3			Bryobacter	0	0	0	0	0	0	0	1	0	0	0	0	1
Cluster9332;:	Proteobac	Gammaproteobacteria				0	0	0	0	0	0	0	0	0	0	0	1	1
Cluster9358;:	Proteobac	Alphaproteobacteria				0	1	0	0	0	0	0	0	0	0	0	0	1
Cluster9369;:	Acidobact	Acidobacteria_Gp3				0	0	0	0	0	0	0	0	0	0	1	0	1
Cluster9371;:	Acidobact	Acidobacteria_Gp2			Gp2	0	0	0	0	0	0	0	0	0	0	1	0	1
Cluster9374;:	Proteobac	Alphap	Rhodospirillales			0	0	0	0	0	0	0	1	0	0	0	0	1
Cluster9376;:	Verrucom	Verruc	Verru	Verrucomicrobiaceae		0	0	0	1	0	0	0	0	0	0	0	0	1

Cluster9381;	Proteobac	Alphap	Rhizobiales			0	0	0	0	0	0	0	0	0	0	1	0	1
Cluster9382;	Acidobact	Acidobacteria_Gp2			Gp2	1	0	0	0	0	0	0	0	0	0	0	0	1
Cluster9408;	Acidobact	Acidobacteria_Gp2			Gp2	0	0	0	0	0	0	0	0	0	0	1	0	1
Cluster9411;	Proteobacteria					0	0	0	0	0	0	0	1	0	0	0	0	1
Cluster9413;	Actinobac	Actinob	Actinomycetales			0	0	0	0	0	0	1	0	0	0	0	0	1
Cluster9414;size=9						0	0	0	0	0	0	0	0	0	0	0	1	1
Cluster9415;	Actinobac	Actinob	Actinomycetales			0	0	0	0	0	0	0	0	0	0	0	1	1
Cluster9417;	Proteobac	Alphap	Caulo	Cauloba	Phenylobacter	1	0	0	0	0	0	0	0	0	0	0	0	1
Cluster9420;	Acidobact	Acidobacteria_Gp1			Gp1	0	0	0	0	0	0	0	0	0	1	0	0	1
Cluster9427;	Acidobact	Acidobacteria_Gp1				0	0	0	0	0	0	0	0	1	0	0	0	1
Cluster9435;	Proteobac	Alphap	Rhod	Rhodos	Dongia	0	0	0	0	0	1	0	0	0	0	0	0	1
Cluster9443;	Actinobac	Actinob	Acidi	Acidimi	Aciditerrimona	0	0	0	0	0	0	0	1	0	0	0	0	1
Cluster9446;	Proteobac	Alphap	Rhizobiales			0	0	0	0	0	0	0	0	0	0	0	1	1
Cluster9447;	Acidobact	Acidobacteria_Gp2			Gp2	0	0	1	0	0	0	0	0	0	0	0	0	1
Cluster9448;	Proteobac	Alphap	Rhizo	Bradyrhizobiaceae		0	0	0	0	0	0	0	1	0	0	0	0	1
Cluster9453;size=1						0	0	0	0	0	0	0	0	0	0	0	1	1
Cluster9455;	Actinobac	Actinob	Actinomycetales			0	0	0	0	0	0	1	0	0	0	0	0	1
Cluster9460;	Acidobact	Acidobacteria_Gp3				0	0	0	0	0	0	0	1	0	0	0	0	1
Cluster9465;	Acidobact	Acidobacteria_Gp2			Gp2	0	0	0	0	0	0	0	1	0	0	0	0	1
Cluster9475;size=1						0	0	0	0	0	0	0	0	0	0	0	1	1
Cluster9478;	Gemmati	Gemm	Gem	Gemma	Gemmatimona	0	0	0	0	0	0	0	1	0	0	0	0	1
Cluster9480;	Proteobac	Gamm	Xanth	Sinobac	Steroidobacter	0	0	0	1	0	0	0	0	0	0	0	0	1
Cluster9500;	Verrucom	Subdivision3			Subdivision3_g	1	0	0	0	0	0	0	0	0	0	0	0	1
Cluster9501;	Verrucom	Opituta	Opitu	Opitutaceae		0	0	0	0	0	0	0	1	0	0	0	0	1
Cluster9502;	Acidobact	Acidobacteria_Gp1				0	0	0	0	0	0	1	0	0	0	0	0	1
Cluster9504;size=1						0	0	0	0	0	0	1	0	0	0	0	0	1
Cluster9510;	Acidobact	Acidobacteria_Gp2			Gp2	0	0	0	0	0	0	0	1	0	0	0	0	1
Cluster9513;	Acidobact	Acidobacteria_Gp3			Gp3	0	0	0	0	0	0	0	0	0	0	0	1	1
Cluster9520;	Proteobac	Alphap	Caulo	Cauloba	Phenylobacter	0	0	1	0	0	0	0	0	0	0	0	0	1

Cluster9527;	Proteobac	Alphap	Rhizc	Beijerinckiaceae		0	0	0	0	0	0	0	0	0	0	1	0	1
Cluster9530;	Acidobact	Acidobacteria_Gp1			Gp1	0	0	1	0	0	0	0	0	0	0	0	0	1
Cluster9535;size=1						0	0	0	0	0	0	0	0	0	0	0	1	1
Cluster9537;	Acidobact	Acidobacteria_Gp1				0	0	0	0	0	0	0	1	0	0	0	0	1
Cluster9553;	Acidobact	Acidobacteria_Gp1				0	0	0	0	0	0	0	1	0	0	0	0	1
Cluster9554;	Acidobact	Acidobacteria_Gp1			Acidobacteriur	0	0	0	0	0	0	0	1	0	0	0	0	1
Cluster9559;	Proteobac	Alphaproteobacteria				1	0	0	0	0	0	0	0	0	0	0	0	1
Cluster9568;	Verrucom	Subdivision3			Subdivision3_g	0	0	0	0	0	0	0	1	0	0	0	0	1
Cluster9577;	Acidobact	Acidobacteria_Gp1				0	0	1	0	0	0	0	0	0	0	0	0	1
Cluster9583;	Actinobac	Actinol	Actin	Mycoba	Mycobacteriur	0	0	0	0	0	0	1	0	0	0	0	0	1
Cluster9587;	Proteobac	Alphap	Rhizc	Bradyrhizobiaceae		1	0	0	0	0	0	0	0	0	0	0	0	1
Cluster9591;size=4						0	0	0	0	0	0	1	0	0	0	0	0	1
Cluster9595;size=1						0	0	0	0	0	0	1	0	0	0	0	0	1
Cluster9596;	Actinobac	Actinol	Solirı	Solirubr	Solirubrobacte	0	0	0	0	0	0	1	0	0	0	0	0	1
Cluster9597;	Proteobac	Betapr	Burkl	Burkhol	Ralstonia	0	0	1	0	0	0	0	0	0	0	0	0	1
Cluster9599;	Armatimonadetes				Armatimonade	1	0	0	0	0	0	0	0	0	0	0	0	1
Cluster9601;	Verrucom	Subdivision3			Subdivision3_g	0	0	0	0	0	0	1	0	0	0	0	0	1
Cluster9602;	Verrucom	Opituta	Opitu	Opituta	Opitutus	0	0	0	0	0	0	0	0	1	0	0	0	1
Cluster9608;	Acidobact	Acidobacteria_Gp2			Gp2	0	0	0	0	0	0	0	0	1	0	0	0	1
Cluster9616;	Proteobac	Alphap	Rhodospirillales			0	0	0	0	0	0	0	0	1	0	0	0	1
Cluster9621;size=1						0	0	1	0	0	0	0	0	0	0	0	0	1
Cluster9622;	Proteobac	Alphap	Rhizc	Bradyrh	Bradyrhizobiur	0	0	0	0	0	0	0	0	0	0	0	1	1
Cluster9626;	Proteobac	Betapr	Burkl	Burkholderiales_incert		0	0	0	0	0	0	0	1	0	0	0	0	1
Cluster9628;	Proteobac	Alphap	Alphaproteol		Rhizomicrobiu	0	0	1	0	0	0	0	0	0	0	0	0	1
Cluster9639;	Proteobac	Gammaproteobacteria				1	0	0	0	0	0	0	0	0	0	0	0	1
Cluster9645;size=1						1	0	0	0	0	0	0	0	0	0	0	0	1
Cluster9648;	Proteobac	Alphaproteobacteria				1	0	0	0	0	0	0	0	0	0	0	0	1
Cluster9653;	Proteobac	Gamm	Xantl	Xanthor	Frateuria	0	0	0	0	0	0	0	0	0	0	1	0	1
Cluster9659;	Proteobac	Alphap	Rhod	Acetobacteraceae		0	0	0	0	0	0	0	0	0	0	0	1	1

Cluster9663;	Proteobac	Deltap	Bdell	Bdellovi	Vampirovibrio	0	0	0	1	0	0	0	0	0	0	0	0	1
Cluster9665;	Acidobact	Acidobacteria_Gp2			Gp2	1	0	0	0	0	0	0	0	0	0	0	0	1
Cluster9666;size=1						0	0	0	0	0	0	1	0	0	0	0	0	1
Cluster9669;	Proteobac	Gamm	Xanth	Sinobac	Steroidobacter	1	0	0	0	0	0	0	0	0	0	0	0	1
Cluster9679;	Verrucom	Subdivision3			Subdivision3_g	0	0	0	0	0	0	1	0	0	0	0	0	1
Cluster9683;	Acidobact	Acidobacteria_Gp1				0	0	0	0	0	0	0	1	0	0	0	0	1
Cluster9688;	Actinobac	Actinob	Soliru	Conexib	Conexibacter	1	0	0	0	0	0	0	0	0	0	0	0	1
Cluster9696;	Proteobac	Alphap	Rhod	Rhodos	Dongia	1	0	0	0	0	0	0	0	0	0	0	0	1
Cluster9699;	Acidobact	Acidobacteria_Gp3			Gp3	1	0	0	0	0	0	0	0	0	0	0	0	1
Cluster9700;	Proteobac	Betaproteobacteria				0	0	0	0	0	0	1	0	0	0	0	0	1
Cluster9704;	Proteobac	Betapr	Burkholderiales			0	0	0	0	0	0	1	0	0	0	0	0	1
Cluster9717;	Proteobac	Alphap	Rhodospirillales			0	0	0	0	0	0	1	0	0	0	0	0	1
Cluster9718;	Proteobac	Alphap	Rhizobiales			0	0	0	1	0	0	0	0	0	0	0	0	1
Cluster9721;	Proteobac	Gammaproteobacteria				1	0	0	0	0	0	0	0	0	0	0	0	1
Cluster9728;	Acidobact	Acidobacteria_Gp2			Gp2	0	0	0	0	0	0	0	1	0	0	0	0	1
Cluster9730;	Acidobact	Acidobacteria_Gp2			Gp2	1	0	0	0	0	0	0	0	0	0	0	0	1
Cluster9737;size=1						1	0	0	0	0	0	0	0	0	0	0	0	1
Cluster9739;	Proteobac	Alphap	Rhodospirillales			0	0	0	0	0	0	0	1	0	0	0	0	1
Cluster9755;size=6						0	0	0	1	0	0	0	0	0	0	0	0	1
Cluster9761;size=3						0	0	0	0	0	0	0	0	0	0	0	1	1
Cluster9770;	Proteobac	Alphap	Rhod	Acetoba	Acidocella	0	0	1	0	0	0	0	0	0	0	0	0	1
Cluster9774;	Proteobac	Alphap	Rhizobiales			1	0	0	0	0	0	0	0	0	0	0	0	1
Cluster9778;	Acidobact	Acidobacteria_Gp1			Gp1	0	0	0	0	0	0	0	1	0	0	0	0	1
Cluster9792;	Acidobact	Acidobacteria_Gp1			Gp1	1	0	0	0	0	0	0	0	0	0	0	0	1
Cluster9796;	Verrucom	Subdivision3			Subdivision3_g	1	0	0	0	0	0	0	0	0	0	0	0	1
Cluster9802;	Proteobac	Betapr	Burkl	Burkhol	Burkholderia	1	0	0	0	0	0	0	0	0	0	0	0	1
Cluster9814;	Proteobac	Alphap	Alphaproteob		Rhizomicrobiu	0	0	0	0	0	0	0	0	0	1	0	0	1
Cluster9816;size=1						0	0	0	0	0	0	1	0	0	0	0	0	1
Cluster9820;	Verrucom	Opituta	Opitu	Opituta	Opitutus	1	0	0	0	0	0	0	0	0	0	0	0	1

Cluster9823;	Acidobact	Acidobacteria_Gp1			Gp1	1	0	0	0	0	0	0	0	0	0	0	0	1
Cluster9824;	Verrucom	Opitut	Opitu	Opituta	Opitutus	0	0	0	0	0	0	1	0	0	0	0	0	1
Cluster9829;	Verrucom	Spartobacteria			Spartobacteria	0	0	0	0	0	0	0	0	1	0	0	0	1
Cluster9831;	Acidobact	Acidobacteria_Gp2			Gp2	0	0	0	0	0	0	0	1	0	0	0	0	1
Cluster9834;	Proteobac	Alphap	Rhizobiales			0	0	0	0	1	0	0	0	0	0	0	0	1
Cluster9836;size=2						0	0	0	0	0	0	1	0	0	0	0	0	1
Cluster9839;	Proteobac	Alphap	Rhodospirillales			0	0	0	0	0	0	1	0	0	0	0	0	1
Cluster9842;	Proteobac	Gammaproteobacteria				0	0	0	0	0	0	0	1	0	0	0	0	1
Cluster9868;	Proteobac	Gamm	Xantl	Sinobac	Steroidobacter	0	1	0	0	0	0	0	0	0	0	0	0	1
Cluster9879;	Chloroflex	Ktedonobacteria				0	0	0	0	0	0	0	0	0	0	0	1	1
Cluster9885;size=2						0	0	0	0	0	0	0	0	0	0	0	1	1
Cluster9894;size=2						0	0	1	0	0	0	0	0	0	0	0	0	1
Cluster9919;	Proteobac	Alphaproteobacteria				0	0	0	0	0	0	1	0	0	0	0	0	1
Cluster9920;	Proteobac	Alphap	Rhodospirillales			1	0	0	0	0	0	0	0	0	0	0	0	1
Cluster9924;size=1						0	0	0	0	0	0	0	1	0	0	0	0	1
Cluster9934;	Cyanobac	Chloroplast		Chlorop	Streptophyta	0	0	0	0	0	0	0	0	0	0	0	1	1
Cluster9937;	Acidobacteria					0	0	0	0	0	0	0	0	0	0	0	1	1
Cluster9967;	Bacteroid	Sphing	Sphir	Chitinophagaceae		1	0	0	0	0	0	0	0	0	0	0	0	1
Cluster9969;	Acidobact	Acidobacteria_Gp2			Gp2	0	0	0	0	0	0	0	0	0	0	0	1	1
Cluster9973;	Proteobac	Alphap	Rhizobiales			0	0	0	0	0	0	0	0	0	0	0	1	1
Cluster9976;	Acidobact	Acidobacteria_Gp2			Gp2	0	0	1	0	0	0	0	0	0	0	0	0	1
Cluster9978;	Proteobac	Alphap	Rhod	Rhodospirillaceae		0	0	0	0	0	0	0	0	1	0	0	0	1
Cluster9985;	Proteobac	Alphap	Rhod	Rhodospirillaceae		0	0	1	0	0	0	0	0	0	0	0	0	1
Cluster9987;	Actinobac	Actinol	Actin	Mycoba	Mycobacteriur	0	0	0	0	0	0	1	0	0	0	0	0	1
Cluster9993;	Proteobacteria					0	0	1	0	0	0	0	0	0	0	0	0	1
Cluster9997;	Proteobac	Alphap	Rhodospirillales			0	0	0	0	0	0	0	0	1	0	0	0	1
Cluster9998;	Acidobact	Acidobacteria_Gp1				0	0	0	0	1	0	0	0	0	0	0	0	1
Cluster10017	Chlamydia	Chlamy	Chlar	Parachla	Parachlamydia	0	0	0	0	0	0	1	0	0	0	0	0	1
Cluster10026;size=7						0	0	0	0	0	0	0	0	0	0	0	1	1

Cluster10029;size=4						1	0	0	0	0	0	0	0	0	0	0	0	1
Cluster10031	Acidobact	Acidobacteria_Gp2			Gp2	0	0	0	0	0	0	1	0	0	0	0	0	1
Cluster10034;size=10						0	0	0	0	0	0	1	0	0	0	0	0	1
Cluster10039	Proteobac	Betaproteobacteria				1	0	0	0	0	0	0	0	0	0	0	0	1
Cluster10045;size=1						1	0	0	0	0	0	0	0	0	0	0	0	1
Cluster10047	Verrucom	Subdivision3			Subdivision3_g	0	0	0	0	0	0	0	0	0	0	0	1	1
Cluster10048	Armatimc	Armati	Arma	Armatir	Armatimonas/.	0	0	0	0	0	0	0	0	0	1	0	0	1
Cluster10052	Proteobacteria					0	0	0	0	1	0	0	0	0	0	0	0	1
Cluster10055	Acidobact	Acidobacteria_Gp1				0	0	0	0	0	0	0	0	1	0	0	0	1
Cluster10064	Acidobact	Acidobacteria_Gp1				0	0	0	0	0	0	0	1	0	0	0	0	1
Cluster10068	Verrucom	Spartobacteria			Spartobacteria	0	0	1	0	0	0	0	0	0	0	0	0	1
Cluster10070	Proteobac	Alphap	Rhizo	Bradyrhizobiaceae		0	1	0	0	0	0	0	0	0	0	0	0	1
Cluster10081	Proteobac	Alphap	Rhizobiales			0	0	0	0	0	1	0	0	0	0	0	0	1
Cluster10086	Proteobac	Alphap	Rhizo	Beijerin	Methylocapsa	1	0	0	0	0	0	0	0	0	0	0	0	1
Cluster10090	Acidobacteria					1	0	0	0	0	0	0	0	0	0	0	0	1
Cluster10094	Acidobact	Acidobacteria_Gp1			Gp1	0	0	0	1	0	0	0	0	0	0	0	0	1
Cluster10097	Proteobac	Alphaproteobacteria				0	0	0	0	0	0	1	0	0	0	0	0	1
Cluster10100	Firmicute:	Bacilli	Bacill	Planococcaceae		0	1	0	0	0	0	0	0	0	0	0	0	1
Cluster10114	Proteobac	Betapr	Burkholderiales			0	0	0	0	0	0	0	1	0	0	0	0	1
Cluster10118	Acidobact	Acidobacteria_Gp2			Gp2	0	0	0	0	0	0	1	0	0	0	0	0	1
Cluster10126	Acidobact	Acidobacteria_Gp1				0	0	0	0	0	0	0	0	0	0	1	0	1
Cluster10130	Proteobac	Alphap	Rhizobiales			0	0	0	0	0	0	1	0	0	0	0	0	1
Cluster10132;size=1						0	0	0	0	0	0	1	0	0	0	0	0	1
Cluster10134	Proteobac	Alphap	Rhizobiales			0	0	0	0	0	0	0	0	0	0	1	0	1
Cluster10140	Proteobac	Deltap	Bdell	Bdellovi	Vampirovibrio	0	0	0	0	1	0	0	0	0	0	0	0	1
Cluster10143	Acidobact	Acidobacteria_Gp1			Acidobacteriur	0	0	0	1	0	0	0	0	0	0	0	0	1
Cluster10145	Acidobact	Acidobacteria_Gp2			Gp2	0	0	0	0	0	0	0	0	0	0	1	0	1
Cluster10150	Actinobac	Actinol	Actinomycetales			0	0	0	0	0	0	1	0	0	0	0	0	1
Cluster10156	OP11				OP11_genera_	0	0	0	0	1	0	0	0	0	0	0	0	1

Cluster10159	Proteobac	Alphap	Rhizo	Methylo	Methylosinus	0	1	0	0	0	0	0	0	0	0	0	0	1
Cluster10160	Proteobac	Deltaproteobacteria				0	0	0	0	0	0	1	0	0	0	0	0	1
Cluster10161	Firmicutes	Clostri	Clost	Peptococcaceae1		0	0	1	0	0	0	0	0	0	0	0	0	1
Cluster10163	Acidobact	Acidobacteria_Gp1			Gp1	0	0	0	0	0	0	0	1	0	0	0	0	1
Cluster10166	Actinobac	Actinob	Soliru	Solirubr	Solirubrobacte	0	0	0	0	1	0	0	0	0	0	0	0	1
Cluster10167;size=7						0	0	0	0	1	0	0	0	0	0	0	0	1
Cluster10180	Proteobac	Alphap	Rhizobiales			0	0	0	0	0	0	1	0	0	0	0	0	1
Cluster10181	Acidobact	Acidobacteria_Gp2			Gp2	0	0	0	0	1	0	0	0	0	0	0	0	1
Cluster10182	Actinobac	Actinob	Actinomycetales			0	0	0	0	1	0	0	0	0	0	0	0	1
Cluster10186	Acidobact	Acidobacteria_Gp10			Gp10	0	0	0	1	0	0	0	0	0	0	0	0	1
Cluster10196	Acidobact	Acidobacteria_Gp2			Gp2	0	0	0	0	1	0	0	0	0	0	0	0	1
Cluster10198	Acidobacteria					0	0	0	0	0	0	0	1	0	0	0	0	1
Cluster10201;size=2						0	0	0	0	0	0	1	0	0	0	0	0	1
Cluster10211	Firmicutes	Bacilli	Bacill	Bacillac	Bacillus	0	0	0	0	0	0	0	0	1	0	0	0	1
Cluster10218	Acidobact	Acidobacteria_Gp1			Gp1	0	0	0	0	0	0	1	0	0	0	0	0	1
Cluster10221	Proteobac	Alphap	Rhizo	Hyphomicrobiaceae		0	0	0	0	0	0	1	0	0	0	0	0	1
Cluster10235;size=2						0	0	0	0	0	0	1	0	0	0	0	0	1
Cluster10236	Proteobac	Alphap	Rhizobiales			0	0	0	0	0	0	1	0	0	0	0	0	1
Cluster10238	Proteobacteria					0	0	0	0	0	0	0	1	0	0	0	0	1
Cluster10242	Actinobac	Actinob	Actin	Microbacteriaceae		0	0	0	0	0	0	0	1	0	0	0	0	1
Cluster10254	Acidobacteria					0	0	0	0	0	0	1	0	0	0	0	0	1
Cluster10260	Acidobact	Acidobacteria_Gp1				0	0	0	0	0	0	1	0	0	0	0	0	1
Cluster10268	Acidobacteria					0	0	0	0	0	0	1	0	0	0	0	0	1
Cluster10269	Acidobact	Acidobacteria_Gp2			Gp2	0	0	0	0	0	0	0	0	0	0	1	0	1
Cluster10273	TM7				TM7_genera_i	0	0	0	0	0	0	0	0	0	0	1	0	1
Cluster10276	Acidobact	Acidobacteria_Gp2			Gp2	0	0	0	0	0	0	0	1	0	0	0	0	1
Cluster10289	Verrucom	Subdivision3			Subdivision3_g	0	0	0	0	1	0	0	0	0	0	0	0	1
Cluster10290	Acidobact	Acidobacteria_Gp2			Gp2	0	0	0	0	1	0	0	0	0	0	0	0	1
Cluster10292	Proteobac	Alphaproteobacteria				0	0	0	0	0	0	1	0	0	0	0	0	1

Cluster1029З	Acidobact	Acidobacteria_Gp1		Gp1	0	0	0	0	1	0	0	0	0	0	0	0	1
Cluster10295;size=1					0	0	0	0	1	0	0	0	0	0	0	0	1
Cluster1029€	Acidobact	Acidobacteria_Gp3		Gp3	0	0	0	0	0	0	0	0	0	0	1	0	1
Cluster1030(	Proteobac	Alphap	Rhizobiales		0	0	0	1	0	0	0	0	0	0	0	0	1
Cluster1030Z	Proteobac	Gammaproteobacteria			0	0	0	0	0	0	1	0	0	0	0	0	1
Cluster10304;size=3					0	0	0	0	0	0	0	1	0	0	0	0	1
Cluster1030E	Acidobact	Acidobacteria_Gp2		Gp2	0	0	0	0	0	0	0	0	0	0	1	0	1
Cluster1030E	Proteobac	Deltaproteobacteria			0	0	0	1	0	0	0	0	0	0	0	0	1
Cluster1031З	Acidobact	Acidobacteria_Gp1			0	0	0	1	0	0	0	0	0	0	0	0	1
Cluster1031S	Planctomycetes				0	0	0	0	1	0	0	0	0	0	0	0	1
Cluster1032З	Proteobac	Betapr	Burkl	Burkhol Ralstonia	0	0	0	0	1	0	0	0	0	0	0	0	1
Cluster1032€	Proteobac	Alphap	Rhizc	Methylocystaceae	0	0	0	0	1	0	0	0	0	0	0	0	1
Cluster1033€	Proteobac	Betapr	Burkl	Burkhol Burkholderia	0	0	0	0	0	0	0	0	0	0	0	1	1
Cluster10338;size=5					0	0	0	0	0	0	1	0	0	0	0	0	1
Cluster10351	Proteobac	Deltap	Myxc	Cystoba Anaeromyxoba	0	0	0	0	1	0	0	0	0	0	0	0	1
Cluster1035Z	Proteobac	Alphap	Rhizobiales		0	0	0	0	0	0	0	0	0	0	1	0	1
Cluster1035E	Acidobact	Acidobacteria_Gp1			0	0	1	0	0	0	0	0	0	0	0	0	1
Cluster10361;size=2					0	0	0	0	0	0	0	0	0	0	0	1	1
Cluster1036S	Proteobac	Betapr	Burkl	Oxalobacteraceae	0	0	0	0	1	0	0	0	0	0	0	0	1
Cluster10371	Proteobac	Gamm	Xantl	Xanthomonadaceae	0	0	0	0	1	0	0	0	0	0	0	0	1
Cluster10386;size=1					0	0	0	0	0	0	0	1	0	0	0	0	1
Cluster10387	Acidobact	Acidobacteria_Gp1		Acidobacteriur	0	0	0	0	0	0	1	0	0	0	0	0	1
Cluster10391	Proteobac	Alphap	Rhizc	Hyphon Rhodoplanes	0	0	0	0	0	0	1	0	0	0	0	0	1
Cluster1039E	Proteobac	Alphap	Rhizc	Bradyrh Agromonas	0	0	0	0	0	0	1	0	0	0	0	0	1
Cluster1039S	Acidobact	Acidobacteria_Gp2		Gp2	0	0	0	1	0	0	0	0	0	0	0	0	1
Cluster1040(	Proteobacteria				0	0	0	0	0	0	0	1	0	0	0	0	1
Cluster1040З	Acidobact	Acidobacteria_Gp1		Gp1	0	0	0	0	0	0	0	0	0	0	0	1	1
Cluster1040E	Proteobac	Betapr	Burkholderiales		0	0	0	0	0	0	0	1	0	0	0	0	1
Cluster1041(	Proteobac	Deltap	Myxococcales		0	0	0	0	0	0	0	0	0	0	0	1	1

Cluster10417	Proteobac	Gammaproteobacteria				0	0	0	0	1	0	0	0	0	0	0	0	1
Cluster10419	Acidobact	Acidobacteria_Gp3			Gp3	0	0	0	1	0	0	0	0	0	0	0	0	1
Cluster10425	Acidobact	Acidobacteria_Gp1				0	0	1	0	0	0	0	0	0	0	0	0	1
Cluster10430	Acidobact	Acidobacteria_Gp2			Gp2	0	0	0	0	1	0	0	0	0	0	0	0	1
Cluster10433	Firmicutes	Bacilli	Bacill	Bacillac	Bacillus	0	0	0	0	0	0	0	0	0	0	1	0	1
Cluster10441	Acidobact	Acidobacteria_Gp2			Gp2	0	0	0	1	0	0	0	0	0	0	0	0	1
Cluster10444	Actinobac	Actinob	Actinomycetales			0	0	0	0	0	0	0	0	0	0	1	0	1
Cluster10447	Acidobact	Acidobacteria_Gp5			Gp5	0	0	0	0	1	0	0	0	0	0	0	0	1
Cluster10451	Firmicutes	Bacilli	Bacill	Planococcaceae		0	0	0	1	0	0	0	0	0	0	0	0	1
Cluster10460	Proteobacteria					0	0	0	0	0	0	0	1	0	0	0	0	1
Cluster10474	Acidobact	Acidobacteria_Gp1			Gp1	0	0	0	1	0	0	0	0	0	0	0	0	1
Cluster10475	Proteobac	Alphaproteobacteria				0	0	0	0	0	0	0	0	0	0	1	0	1
Cluster10482	Acidobact	Acidobacteria_Gp2			Gp2	0	0	0	1	0	0	0	0	0	0	0	0	1
Cluster10488	Proteobac	Betapr	Burkl	Burkhol	Burkholderia	0	0	0	0	0	0	1	0	0	0	0	0	1
Cluster10489	Acidobact	Acidobacteria_Gp1			Gp1	0	0	0	0	0	0	0	0	0	0	1	0	1
Cluster10495	Proteobac	Gammaproteobacteria				0	0	0	1	0	0	0	0	0	0	0	0	1
Cluster10502	Acidobact	Acidobacteria_Gp10			Gp10	0	0	0	0	0	0	0	1	0	0	0	0	1
Cluster10503	Planctomy	Plancto	Planc	Plancto	Planctomyces	0	0	0	0	0	0	1	0	0	0	0	0	1
Cluster10511	Acidobact	Acidobacteria_Gp1			Edaphobacter	0	0	0	0	0	0	0	0	0	1	0	0	1
Cluster10514	Proteobac	Alphaproteobacteria				0	0	0	0	0	0	1	0	0	0	0	0	1
Cluster10537	Proteobacteria					0	0	0	0	0	0	1	0	0	0	0	0	1
Cluster10544	Acidobact	Acidobacteria_Gp2			Gp2	0	0	0	0	0	0	0	0	0	0	1	0	1
Cluster10554	Proteobac	Alphap	Rhizobiales			0	0	0	0	0	0	1	0	0	0	0	0	1
Cluster10557	Acidobact	Acidobacteria_Gp2			Gp2	0	0	0	1	0	0	0	0	0	0	0	0	1
Cluster10559;size=4						0	0	0	1	0	0	0	0	0	0	0	0	1
Cluster10560	Acidobacteria					0	0	0	0	0	1	0	0	0	0	0	0	1
Cluster10565	Acidobact	Acidobacteria_Gp2			Gp2	0	0	0	1	0	0	0	0	0	0	0	0	1
Cluster10582	Acidobact	Acidobacteria_Gp1			Gp1	0	0	0	1	0	0	0	0	0	0	0	0	1
Cluster10589	Proteobac	Alphap	Rhizo	Hyphomicrobiaceae		0	0	0	0	0	0	0	0	1	0	0	0	1

Cluster10597	Acidobact	Acidobacteria_Gp1			Gp1	0	0	1	0	0	0	0	0	0	0	0	0	1
Cluster10602	Verrucom	Spartobacteria			Spartobacteria	0	0	0	0	0	0	1	0	0	0	0	0	1
Cluster10605	Firmicutes	Bacilli	Bacill	Staphyl	Staphylococcu	0	0	0	0	0	0	1	0	0	0	0	0	1
Cluster10606	Planctomycetes					0	0	0	0	0	0	1	0	0	0	0	0	1
Cluster10608;size=2						0	0	0	0	0	0	0	1	0	0	0	0	1
Cluster10610	Proteobac	Alphap	Rhizc	Beijerin	Methylocella	0	0	0	0	0	0	0	0	1	0	0	0	1
Cluster10613;size=1						0	0	0	0	0	0	0	1	0	0	0	0	1
Cluster10622;size=3						0	0	0	1	0	0	0	0	0	0	0	0	1
Cluster10623	Proteobac	Deltap	Myxc	Kofleria	Kofleria	0	0	0	1	0	0	0	0	0	0	0	0	1
Cluster10627	Proteobac	Deltap	Myxc	Polyangiaceae		0	0	0	0	0	0	1	0	0	0	0	0	1
Cluster10654	Proteobac	Alphap	Rhizc	Beijerinckiaceae		0	0	0	0	0	0	0	1	0	0	0	0	1
Cluster10656	Actinobac	Actinol	Actinomycetales			0	1	0	0	0	0	0	0	0	0	0	0	1
Cluster10663	Proteobac	Betapr	Burkl	Comamonadaceae		0	0	0	0	0	0	0	1	0	0	0	0	1
Cluster10668	Acidobact	Acidobacteria_Gp2			Gp2	0	0	0	0	1	0	0	0	0	0	0	0	1
Cluster10673	Acidobact	Acidobacteria_Gp2			Gp2	0	0	0	1	0	0	0	0	0	0	0	0	1
Cluster10685	Proteobac	Alphap	Rhizobiales			1	0	0	0	0	0	0	0	0	0	0	0	1
Cluster10686	Proteobac	Betaproteobacteria				0	0	0	0	0	0	0	1	0	0	0	0	1
Cluster10687;size=2						1	0	0	0	0	0	0	0	0	0	0	0	1
Cluster10700	Acidobact	Acidobacteria_Gp1			Gp1	0	0	1	0	0	0	0	0	0	0	0	0	1
Cluster10704	Acidobact	Acidobacteria_Gp2			Gp2	0	0	0	1	0	0	0	0	0	0	0	0	1
Cluster10708	Acidobact	Acidobacteria_Gp1			Gp1	0	0	0	0	0	0	0	0	0	0	1	0	1
Cluster10720	Verrucom	Spartobacteria			Spartobacteria	0	0	0	0	0	0	0	1	0	0	0	0	1
Cluster10728;size=4						0	0	0	0	0	0	0	0	0	0	0	1	1
Cluster10730	Acidobact	Acidobacteria_Gp2			Gp2	0	1	0	0	0	0	0	0	0	0	0	0	1
Cluster10732	Acidobact	Acidobacteria_Gp1			Gp1	0	0	0	0	0	0	0	1	0	0	0	0	1
Cluster10734	Proteobac	Alphap	Rhizc	Xanthol	Pseudolabrys	0	0	0	1	0	0	0	0	0	0	0	0	1
Cluster10735	Acidobact	Acidobacteria_Gp1				0	0	0	0	1	0	0	0	0	0	0	0	1
Cluster10737	Proteobac	Alphap	Rhizc	Hyphon	Rhodoplanes	0	0	0	1	0	0	0	0	0	0	0	0	1
Cluster10741	Bacteroid	Sphing	Sphir	Chitinop	Sediminibacter	0	0	0	1	0	0	0	0	0	0	0	0	1

Cluster10742	Proteobac	Alphap	Rhodospirillales			0	0	0	0	0	0	0	1	0	0	0	0	1
Cluster10744	Proteobac	Alphaproteobacteria				0	0	0	1	0	0	0	0	0	0	0	0	1
Cluster10757	Acidobact	Acidobacteria_Gp1			Gp1	0	0	0	1	0	0	0	0	0	0	0	0	1
Cluster10766;size=7						0	0	0	0	0	0	0	1	0	0	0	0	1
Cluster10769;size=1						0	0	0	0	0	0	0	0	0	0	1	0	1
Cluster10770	Proteobacteria					0	0	0	0	0	0	0	1	0	0	0	0	1
Cluster10772	Proteobac	Alphap	Rhizc	Bradyrhizobiaceae		0	0	0	0	0	0	0	1	0	0	0	0	1
Cluster10779	Proteobac	Alphap	Rhizobiales			0	0	0	0	0	0	0	0	0	1	0	0	1
Cluster10784	Actinobac	Actinol	Acidi	Acidimi	Aciditerrimona	0	0	0	1	0	0	0	0	0	0	0	0	1
Cluster10787	Proteobac	Deltap	Myxococcales			0	0	0	0	1	0	0	0	0	0	0	0	1
Cluster10790	Proteobac	Betapr	Burkholderiales			0	0	0	0	0	0	0	1	0	0	0	0	1
Cluster10794	Acidobact	Acidobacteria_Gp3			Gp3	0	0	0	1	0	0	0	0	0	0	0	0	1
Cluster10796	Acidobact	Acidobacteria_Gp1			Gp1	1	0	0	0	0	0	0	0	0	0	0	0	1
Cluster10804	Acidobacteria					0	0	0	0	0	0	0	0	0	0	1	0	1
Cluster10805	Proteobac	Alphaproteobacteria				0	0	0	1	0	0	0	0	0	0	0	0	1
Cluster10812	Acidobact	Acidobacteria_Gp1				0	0	0	0	0	0	1	0	0	0	0	0	1
Cluster10813	Acidobact	Acidobacteria_Gp1				0	0	0	0	0	1	0	0	0	0	0	0	1
Cluster10819	Acidobact	Acidobacteria_Gp1			Gp1	0	0	0	1	0	0	0	0	0	0	0	0	1
Cluster10828	Acidobact	Acidobacteria_Gp3				0	0	0	0	0	0	0	0	0	0	0	1	1
Cluster10829	Acidobact	Acidobacteria_Gp1			Gp1	0	0	0	0	0	0	0	1	0	0	0	0	1
Cluster10832	Armatimonadetes				Armatimonade	0	0	0	1	0	0	0	0	0	0	0	0	1
Cluster10834	Proteobacteria					0	0	0	1	0	0	0	0	0	0	0	0	1
Cluster10846	Actinobac	Actinol	Actinomycetales			0	0	1	0	0	0	0	0	0	0	0	0	1
Cluster10852	Verrucom	Opituta	Opitu	Opituta	Opitutus	0	0	0	0	0	0	0	1	0	0	0	0	1
Cluster10856	Proteobac	Deltap	Bdell	Bdellovi	Vampirovibrio	1	0	0	0	0	0	0	0	0	0	0	0	1
Cluster10858	Proteobac	Gamm	Xantl	Xanthor	Aquimonas	1	0	0	0	0	0	0	0	0	0	0	0	1
Cluster10861	Proteobac	Betaproteobacteria				0	0	0	1	0	0	0	0	0	0	0	0	1
Cluster10863	Acidobact	Acidobacteria_Gp2			Gp2	0	0	0	1	0	0	0	0	0	0	0	0	1
Cluster10865	Acidobact	Acidobacteria_Gp1			Gp1	0	0	0	1	0	0	0	0	0	0	0	0	1

Cluster1087(	TM7				TM7_genera_i	0	0	0	0	0	0	0	1	0	0	0	0	1
Cluster1087€	Armatimc	Armati	Arma	Armatir	Armatimonas/.	0	0	0	1	0	0	0	0	0	0	0	0	1
Cluster10881	Proteobac	Deltap	Bdell	Bdellovi	Vampirovibrio	0	0	0	0	0	0	0	0	0	0	1	0	1
Cluster10882	Proteobac	Alphap	Rhizobiales			0	0	0	0	0	0	0	1	0	0	0	0	1
Cluster1089€	Proteobac	Deltap	Myxococcales			0	0	0	1	0	0	0	0	0	0	0	0	1
Cluster1090З	Proteobac	Deltap	Myxc	Polyangiaceae		0	0	0	0	0	0	1	0	0	0	0	0	1
Cluster10907	Proteobac	Betapr	Burkl	Burkhol	Xylophilus	0	0	0	0	0	0	0	1	0	0	0	0	1
Cluster1091(	Proteobac	Deltaproteobacteria				0	0	0	0	0	0	1	0	0	0	0	0	1
Cluster1092(	Acidobact	Acidobacteria_Gp6			Gp6	0	0	0	1	0	0	0	0	0	0	0	0	1
Cluster1094(	Chlamydia	Chlamy	Chlar	Parachla	Parachlamydia	0	0	1	0	0	0	0	0	0	0	0	0	1
Cluster10941	Acidobact	Acidobacteria_Gp3				0	0	1	0	0	0	0	0	0	0	0	0	1
Cluster10947	Acidobact	Acidobacteria_Gp1			Gp1	0	0	0	0	0	0	0	1	0	0	0	0	1
Cluster1094９	Acidobact	Acidobacteria_Gp1				1	0	0	0	0	0	0	0	0	0	0	0	1
Cluster10952	Acidobact	Acidobacteria_Gp3			Gp3	0	0	0	1	0	0	0	0	0	0	0	0	1
Cluster10954	Acidobact	Acidobacteria_Gp2			Gp2	0	0	0	0	0	0	1	0	0	0	0	0	1
Cluster1095９	Proteobac	Alphap	Rhizc	Bradyrh	Agromonas	0	0	0	1	0	0	0	0	0	0	0	0	1
Cluster1097Ѕ	Proteobac	Alphap	Rhizc	Xanthol	Pseudolabrys	0	0	0	0	0	0	0	0	0	0	1	0	1
Cluster1098(	Proteobac	Deltap	Myxococcales			0	0	0	1	0	0	0	0	0	0	0	0	1
Cluster10984	Proteobac	Deltap	Myxococcales			0	0	0	0	0	0	0	0	0	0	1	0	1
Cluster1100(	Acidobact	Acidobacteria_Gp2			Gp2	0	0	0	0	0	0	0	0	0	0	1	0	1
Cluster1100€	Proteobac	Alphap	Rhizc	Xanthol	Labrys	1	0	0	0	0	0	0	0	0	0	0	0	1
Cluster11007	Acidobact	Acidobacteria_Gp2			Gp2	0	0	0	1	0	0	0	0	0	0	0	0	1
Cluster11014	Actinobac	Actinol	Actinomycetales			0	0	0	0	0	0	0	0	0	0	1	0	1
Cluster1101€	Acidobact	Acidobacteria_Gp1				0	1	0	0	0	0	0	0	0	0	0	0	1
Cluster11019;size=7						1	0	0	0	0	0	0	0	0	0	0	0	1
Cluster1102€	Acidobact	Acidobacteria_Gp1			Acidobacteriur	0	0	0	0	0	0	1	0	0	0	0	0	1
Cluster11027	Acidobact	Acidobacteria_Gp1			Gp1	0	0	0	1	0	0	0	0	0	0	0	0	1
Cluster11031	Acidobact	Acidobacteria_Gp1				0	0	0	1	0	0	0	0	0	0	0	0	1
Cluster1103€	Acidobact	Acidobacteria_Gp1			Gp1	0	0	0	1	0	0	0	0	0	0	0	0	1

Cluster11039	Proteobac	Alphap	Rhizc	Xanthol	Pseudolabrys	0	0	0	0	0	0	0	0	0	0	0	1	1
Cluster11050	Verrucom	Subdivision3			Subdivision3_	0	0	0	0	0	0	1	0	0	0	0	0	1
Cluster11055	Gemmati	Gemm	Gem	Gemma	Gemmatimona	0	0	0	0	0	0	1	0	0	0	0	0	1
Cluster11066	Proteobac	Betaproteobacteria				0	0	0	0	0	0	1	0	0	0	0	0	1
Cluster11093	Proteobac	Gamm	Xanthomonadales			1	0	0	0	0	0	0	0	0	0	0	0	1
Cluster11095	Acidobact	Acidobacteria_Gp1				0	0	0	1	0	0	0	0	0	0	0	0	1
Cluster11098	Proteobac	Alphap	Sphir	Sphingomonadaceae		0	0	0	0	0	0	0	1	0	0	0	0	1
Cluster11101	Proteobac	Betapr	Burkholderiales			0	0	0	0	0	0	0	1	0	0	0	0	1
Cluster11108	Proteobacteria					0	0	0	0	0	0	0	0	0	0	1	0	1
Cluster11109	Proteobac	Gammaproteobacteria				0	0	0	0	0	0	1	0	0	0	0	0	1
Cluster11111	Acidobact	Acidobacteria_Gp3			Gp3	0	0	0	1	0	0	0	0	0	0	0	0	1
Cluster11112	Acidobact	Acidobacteria_Gp2			Gp2	0	0	0	1	0	0	0	0	0	0	0	0	1
Cluster11119	Proteobac	Alphap	Rhod	Rhodospirillaceae		0	0	1	0	0	0	0	0	0	0	0	0	1
Cluster11122	Acidobact	Acidobacteria_Gp1				0	0	1	0	0	0	0	0	0	0	0	0	1
Cluster11125	Acidobact	Acidobacteria_Gp1			Gp1	0	0	0	1	0	0	0	0	0	0	0	0	1
Cluster11126	Proteobac	Alphap	Rhizc	Bradyrhizobiaceae		0	0	0	0	0	0	0	1	0	0	0	0	1
Cluster11128	Proteobac	Alphap	Rhizobiales			0	0	0	1	0	0	0	0	0	0	0	0	1
Cluster11131	Proteobac	Alphap	Rhodospirillales			0	0	0	0	0	0	0	1	0	0	0	0	1
Cluster11135	Proteobacteria					0	0	1	0	0	0	0	0	0	0	0	0	1
Cluster11140	Acidobact	Acidobacteria_Gp2			Gp2	0	0	1	0	0	0	0	0	0	0	0	0	1
Cluster11141	Proteobacteria					0	0	0	1	0	0	0	0	0	0	0	0	1
Cluster11143	Proteobac	Alphap	Caulc	Caulobac	Phenylobacter	0	0	0	0	0	0	0	1	0	0	0	0	1
Cluster11146;size=1						0	0	0	0	0	0	1	0	0	0	0	0	1
Cluster11149;size=1						0	0	0	0	0	0	1	0	0	0	0	0	1
Cluster11155	Proteobac	Alphaproteobacteria				0	0	0	1	0	0	0	0	0	0	0	0	1
Cluster11157;size=2						0	0	0	1	0	0	0	0	0	0	0	0	1
Cluster11162	Acidobact	Acidobacteria_Gp2			Gp2	0	0	0	0	0	0	0	0	1	0	0	0	1
Cluster11163;size=1						0	0	0	1	0	0	0	0	0	0	0	0	1
Cluster11169;size=5						1	0	0	0	0	0	0	0	0	0	0	0	1

Cluster11171	Armatimonadetes				Armatimonade	0	0	0	0	0	0	1	0	0	0	0	0	1
Cluster11176	Proteobac	Gamm	Xantl	Xanthor	Frateuria	0	0	0	0	0	0	1	0	0	0	0	0	1
Cluster11179	Acidobact	Acidobacteria_Gp1			Acidobacteriur	0	0	0	0	0	1	0	0	0	0	0	0	1
Cluster11182	Proteobac	Deltap	Myxc	Polyangiaceae		0	0	0	0	0	0	0	0	1	0	0	0	1
Cluster11194	Acidobacteria					0	0	1	0	0	0	0	0	0	0	0	0	1
Cluster11196	Proteobac	Deltap	Myxococcales			0	0	0	0	0	0	1	0	0	0	0	0	1
Cluster11206;size=5						0	0	0	0	0	0	0	0	0	0	1	0	1
Cluster11207;size=3						0	0	0	0	0	0	1	0	0	0	0	0	1
Cluster11211	Verrucom	Opituta	Opitu	Opituta	Opitutus	1	0	0	0	0	0	0	0	0	0	0	0	1
Cluster11229	Acidobact	Acidobacteria_Gp1				0	0	0	0	0	0	1	0	0	0	0	0	1
Cluster11239	Proteobacteria					0	0	0	0	0	0	0	0	1	0	0	0	1
Cluster11253	Proteobac	Alphap	Rhizobiales			0	0	0	0	0	0	0	0	0	0	0	1	1
Cluster11254	Proteobac	Alphap	Rhizc	Bradyrhizobiaceae		0	0	0	0	0	0	0	0	1	0	0	0	1
Cluster11255	Actinobac	Actinol	Actinomycetales			0	0	0	0	0	0	0	0	1	0	0	0	1
Cluster11267	Proteobac	Gamm	Thiot	Thiotric	Caedibacter	0	0	0	0	0	0	0	0	1	0	0	0	1
Cluster11276;size=3						0	0	0	0	0	1	0	0	0	0	0	0	1
Cluster11277	Acidobact	Acidobacteria_Gp1			Gp1	0	0	0	0	0	0	0	0	1	0	0	0	1
Cluster11280	Acidobact	Acidobacteria_Gp1				0	0	0	0	0	0	1	0	0	0	0	0	1
Cluster11293;size=1						0	0	0	0	0	1	0	0	0	0	0	0	1
Cluster11294	Actinobac	Actinol	Solirı	Solirubr	Solirubrobacte	0	0	0	0	0	0	1	0	0	0	0	0	1
Cluster11297	Proteobac	Deltap	Myxococcales			0	0	0	0	0	0	1	0	0	0	0	0	1
Cluster11303	Proteobac	Alphap	Rhizobiales			0	0	0	0	0	1	0	0	0	0	0	0	1
Cluster11306;size=6						0	0	0	0	0	0	1	0	0	0	0	0	1
Cluster11309	Proteobac	Alphap	Rhodospirillales			0	0	0	0	0	0	0	1	0	0	0	0	1
Cluster11313	Proteobac	Gamm	Legio	Coxiella	Aquicella	0	0	0	0	0	0	0	0	1	0	0	0	1
Cluster11314	Proteobac	Alphaproteobacteria				0	0	0	0	0	0	1	0	0	0	0	0	1
Cluster11321;size=4						0	0	0	0	0	0	0	0	0	0	1	0	1
Cluster11322	Proteobac	Alphap	Rhizc	Beijerinckiaceae		0	0	0	0	0	1	0	0	0	0	0	0	1
Cluster11336	Acidobact	Acidobacteria_Gp1				0	0	0	0	0	0	0	0	0	0	0	1	1

Cluster11351	Proteobacteria					0	1	0	0	0	0	0	0	0	0	0	0	1
Cluster11358	Actinobac	Actinol	Solirı	Solirubr	Solirubrobacte	0	0	0	0	1	0	0	0	0	0	0	0	1
Cluster11359	Acidobact	Acidobacteria_Gp3			Gp3	0	0	0	0	0	1	0	0	0	0	0	0	1
Cluster11374;size=2						0	0	0	0	0	1	0	0	0	0	0	0	1
Cluster11381	Acidobact	Acidobacteria_Gp5			Gp5	0	0	0	0	0	0	0	0	1	0	0	0	1
Cluster11387	Acidobact	Acidobacteria_Gp2			Gp2	0	0	0	0	0	1	0	0	0	0	0	0	1
Cluster11388	Acidobact	Acidobacteria_Gp1			Gp1	0	0	0	0	0	0	0	0	0	0	0	1	1
Cluster11394	Acidobact	Acidobacteria_Gp1				1	0	0	0	0	0	0	0	0	0	0	0	1
Cluster11396	Proteobac	Alphaproteobacteria				1	0	0	0	0	0	0	0	0	0	0	0	1
Cluster11397	Acidobact	Acidobacteria_Gp13			Gp13	1	0	0	0	0	0	0	0	0	0	0	0	1
Cluster11398	Bacteroidetes					0	0	0	0	0	1	0	0	0	0	0	0	1
Cluster11401	Acidobact	Acidobacteria_Gp2			Gp2	0	0	0	0	0	0	0	0	0	0	0	1	1
Cluster11403	Proteobacteria					0	0	0	0	0	1	0	0	0	0	0	0	1
Cluster11412	Proteobac	Betapr	Burkholderiales			0	0	0	0	0	0	1	0	0	0	0	0	1
Cluster11419	Proteobac	Alphap	Rhizobiales			0	0	0	0	0	0	1	0	0	0	0	0	1
Cluster11423	Acidobact	Acidobacteria_Gp1			Acidobacteriur	0	0	1	0	0	0	0	0	0	0	0	0	1
Cluster11424	Proteobac	Gammaproteobacteria				0	0	1	0	0	0	0	0	0	0	0	0	1
Cluster11425	Bacteroid	Sphing	Sphir	Sphingc	Mucilaginibact	0	0	0	0	0	0	1	0	0	0	0	0	1
Cluster11426	Acidobact	Acidobacteria_Gp1			Gp1	0	0	0	0	0	0	1	0	0	0	0	0	1
Cluster11452;size=2						0	0	0	0	0	0	0	0	0	0	0	1	1
Cluster11461;size=6						0	0	0	0	0	0	1	0	0	0	0	0	1
Cluster11470	Proteobac	Alphap	Caulc	Caulobа	Phenylobacter	0	0	0	0	0	0	1	0	0	0	0	0	1
Cluster11475	Proteobac	Betapr	Neiss	Neisseriaceae		0	0	0	0	0	0	1	0	0	0	0	0	1
Cluster11476	Proteobac	Betaproteobacteria				0	1	0	0	0	0	0	0	0	0	0	0	1
Cluster11477	Actinobac	Actinol	Actinomycetales			0	0	0	0	0	0	0	1	0	0	0	0	1
Cluster11495	Acidobact	Acidobacteria_Gp2			Gp2	0	0	0	0	0	1	0	0	0	0	0	0	1
Cluster11498	Proteobac	Alphaproteobacteria				0	0	0	0	0	0	0	0	0	0	1	0	1
Cluster11499;size=1						0	0	0	0	0	1	0	0	0	0	0	0	1
Cluster11509	Acidobact	Acidobacteria_Gp1			Gp1	0	0	0	0	0	0	0	0	1	0	0	0	1

Cluster11512;size=1						0	0	0	0	0	0	1	0	0	0	0	0	1
Cluster11515	Chlamydia	Chlamy	Chlamydiales			0	0	0	0	0	0	0	0	1	0	0	0	1
Cluster11519	Proteobac	Alphaproteobacteria				0	0	0	0	0	0	1	0	0	0	0	0	1
Cluster11523	Actinobac	Actinol	Actinomycetales			0	0	0	0	0	0	1	0	0	0	0	0	1
Cluster11535	Proteobac	Alphap	Rhodospirillales			0	0	0	0	0	0	0	0	0	0	1	0	1
Cluster11545	Acidobact	Acidobacteria_Gp1			Gp1	0	0	0	0	0	0	1	0	0	0	0	0	1
Cluster11550	Acidobact	Acidobacteria_Gp1				0	0	1	0	0	0	0	0	0	0	0	0	1
Cluster11552	Proteobac	Alphap	Rhizobiales			0	0	0	0	0	0	0	0	1	0	0	0	1
Cluster11558	Proteobac	Gammaproteobacteria				0	0	0	0	0	0	1	0	0	0	0	0	1
Cluster11568	Acidobact	Acidobacteria_Gp5			Gp5	0	0	0	0	0	0	1	0	0	0	0	0	1
Cluster11577	Proteobacteria					0	0	0	0	0	0	1	0	0	0	0	0	1
Cluster11578	Verrucom	Spartobacteria			Spartobacteria	0	0	0	0	0	0	1	0	0	0	0	0	1
Cluster11579;size=10						0	0	0	0	0	0	0	0	1	0	0	0	1
Cluster11581	Planctom	Plancto	Planc	Plancto	Planctomyces	0	0	0	0	1	0	0	0	0	0	0	0	1
Cluster11587	Acidobact	Acidobacteria_Gp13			Gp13	0	0	0	0	1	0	0	0	0	0	0	0	1
Cluster11590	Verrucom	Opitutae				1	0	0	0	0	0	0	0	0	0	0	0	1
Cluster11600	Acidobact	Acidobacteria_Gp10			Gp10	0	0	0	0	0	0	0	1	0	0	0	0	1
Cluster11604	Acidobact	Acidobacteria_Gp1			Edaphobacter	0	0	0	0	0	0	0	0	1	0	0	0	1
Cluster11621	Acidobact	Acidobacteria_Gp2			Gp2	0	0	0	1	0	0	0	0	0	0	0	0	1
Cluster11631	Proteobac	Alphap	Rhodospirillales			0	0	0	0	0	1	0	0	0	0	0	0	1
Cluster11641	Acidobact	Acidobacteria_Gp1			Gp1	1	0	0	0	0	0	0	0	0	0	0	0	1
Cluster11651;size=2						0	0	0	0	0	0	0	0	1	0	0	0	1
Cluster11657	Acidobact	Acidobacteria_Gp7			Gp7	0	0	0	0	0	0	0	0	0	0	0	1	1
Cluster11659	Proteobac	Alphaproteobacteria				0	0	0	0	0	1	0	0	0	0	0	0	1
Cluster11664	Proteobacteria					0	0	0	0	0	0	1	0	0	0	0	0	1
Cluster11665	Proteobac	Alphap	Rhizobiales			0	0	1	0	0	0	0	0	0	0	0	0	1
Cluster11671	OP11				OP11_genera_	0	0	0	0	0	0	0	0	1	0	0	0	1
Cluster11673	Armatimonadetes				Armatimonade	0	0	0	0	0	0	1	0	0	0	0	0	1
Cluster11675	Chloroflex	Ktedor	Ktedonobacterales			0	0	0	0	0	0	0	0	1	0	0	0	1

Cluster11677	Acidobact	Acidobacteria_Gp1				0	0	0	0	0	0	0	1	0	0	0	0	1
Cluster11701	Acidobact	Acidobacteria_Gp1			Edaphobacter	0	0	0	0	0	0	0	0	1	0	0	0	1
Cluster11703	Verrucom	Opitut	Opitu	Opituta	Opitutus	0	0	0	0	0	1	0	0	0	0	0	0	1
Cluster11704	Acidobact	Acidobacteria_Gp2			Gp2	0	0	0	1	0	0	0	0	0	0	0	0	1
Cluster11714	Planctomy	Plancto	Planc	Planctomycetaceae		0	0	0	0	0	0	1	0	0	0	0	0	1
Cluster11721	Proteobac	Betapr	Burkl	Burkholderiaceae		1	0	0	0	0	0	0	0	0	0	0	0	1
Cluster11728	Acidobact	Acidobacteria_Gp2			Gp2	0	0	0	0	0	0	0	0	1	0	0	0	1
Cluster11729	Proteobacteria					0	0	0	0	0	0	0	0	1	0	0	0	1
Cluster11732	Proteobac	Gammaproteobacteria				0	0	0	0	0	0	1	0	0	0	0	0	1
Cluster11738	Acidobacteria					0	0	0	0	1	0	0	0	0	0	0	0	1
Cluster11740	Acidobact	Acidobacteria_Gp6			Gp6	0	0	0	0	0	0	1	0	0	0	0	0	1
Cluster11746	Proteobac	Betaproteobacteria				0	0	0	0	0	1	0	0	0	0	0	0	1
Cluster11749	Verrucom	Spartobacteria			Spartobacteria	0	0	0	0	0	0	1	0	0	0	0	0	1
Cluster11750	Proteobacteria					0	0	0	0	1	0	0	0	0	0	0	0	1
Cluster11755	Proteobac	Alphap	Rhodospirillales			0	0	0	0	0	0	0	1	0	0	0	0	1
Cluster11758;size=1						0	0	0	0	0	0	1	0	0	0	0	0	1
Cluster11759	Acidobact	Acidobacteria_Gp2			Gp2	0	0	0	0	0	0	0	0	0	1	0	0	1
Cluster11763;size=1						0	0	0	0	0	0	0	0	0	1	0	0	1
Cluster11767	Acidobact	Acidobacteria_Gp3				0	0	0	0	0	0	0	1	0	0	0	0	1
Cluster11778	Proteobac	Alphap	Rhodospirillales			0	0	0	0	0	0	1	0	0	0	0	0	1
Cluster11780	Proteobac	Alphap	Caulo	Cauloba	Caulobacter	0	0	0	0	0	0	0	0	0	1	0	0	1
Cluster11781	Acidobact	Acidobacteria_Gp2			Gp2	0	0	0	0	1	0	0	0	0	0	0	0	1
Cluster11787	Proteobac	Alphap	Rhodospirillales			0	0	0	0	0	0	0	0	0	1	0	0	1
Cluster11788	Proteobac	Alphap	Alphaproteob		Rhizomicrobiu	0	0	1	0	0	0	0	0	0	0	0	0	1
Cluster11802	Acidobact	Acidobacteria_Gp1				1	0	0	0	0	0	0	0	0	0	0	0	1
Cluster11804	Acidobact	Acidobacteria_Gp3				0	0	0	0	1	0	0	0	0	0	0	0	1
Cluster11811	Proteobac	Betaproteobacteria				0	0	0	0	0	0	0	0	0	1	0	0	1
Cluster11816	Acidobacteria					0	0	0	0	0	0	0	1	0	0	0	0	1
Cluster11817	Acidobact	Acidobacteria_Gp1			Gp1	0	0	0	0	1	0	0	0	0	0	0	0	1

Cluster11821	Acidobact	Acidobacteria_Gp3			Gp3	0	0	0	0	0	0	0	0	0	1	0	0	1
Cluster11824;size=1						0	0	0	0	1	0	0	0	0	0	0	0	1
Cluster11826	Acidobact	Acidobacteria_Gp3			Gp3	0	0	0	0	1	0	0	0	0	0	0	0	1
Cluster11827	Proteobac	Alphap	Rhizobiales			0	0	0	0	1	0	0	0	0	0	0	0	1
Cluster11831	Acidobact	Acidobacteria_Gp6			Gp6	0	0	0	0	0	0	0	0	0	1	0	0	1
Cluster11832	Proteobac	Deltap	Bdell	Bdellovi	Bdellovibrio	0	0	0	0	0	0	0	0	0	1	0	0	1
Cluster11850	Acidobact	Acidobacteria_Gp1				0	0	1	0	0	0	0	0	0	0	0	0	1
Cluster11859	Proteobacteria					0	0	0	0	1	0	0	0	0	0	0	0	1
Cluster11860	Armatimc	Armati	Arma	Armatir	Armatimonas/	0	0	0	0	0	0	0	0	0	0	0	1	1
Cluster11863	Actinobac	Actinol	Solirubrobacterales			0	0	0	0	0	0	0	0	0	0	1	0	1
Cluster11865	Proteobac	Alphap	Rhodospirillales			1	0	0	0	0	0	0	0	0	0	0	0	1
Cluster11869;size=3						0	0	1	0	0	0	0	0	0	0	0	0	1
Cluster11872	Acidobact	Acidobacteria_Gp2			Gp2	0	0	0	0	0	0	1	0	0	0	0	0	1
Cluster11882	Proteobac	Alphap	Rhizobiales			0	0	0	0	1	0	0	0	0	0	0	0	1
Cluster11894	Acidobact	Acidobacteria_Gp1			Gp1	0	0	0	0	0	0	0	0	0	0	0	1	1
Cluster11897	Acidobact	Acidobacteria_Gp2			Gp2	0	0	0	0	0	0	1	0	0	0	0	0	1
Cluster11904	Proteobac	Alphap	Rhod	Rhodos	Dongia	0	0	0	0	1	0	0	0	0	0	0	0	1
Cluster11913	Actinobac	Actinobacteria				0	1	0	0	0	0	0	0	0	0	0	0	1
Cluster11918	Proteobac	Alphap	Caulc	Culoba	Phenylobacter	0	0	1	0	0	0	0	0	0	0	0	0	1
Cluster11919;size=3						0	0	0	0	1	0	0	0	0	0	0	0	1
Cluster11920	Acidobacteria					0	0	0	0	1	0	0	0	0	0	0	0	1
Cluster11927	Proteobac	Alphap	Rhod	Acetobacteraceae		0	0	0	0	0	0	0	0	0	1	0	0	1
Cluster11933	Acidobact	Acidobacteria_Gp3				0	0	0	0	0	0	1	0	0	0	0	0	1
Cluster11934	Proteobac	Betapr	Burkholderiales			0	0	1	0	0	0	0	0	0	0	0	0	1
Cluster11939;size=3						0	0	0	0	0	0	0	1	0	0	0	0	1
Cluster11940	Proteobac	Betapr	Burkl	Burkhol	Burkholderia	0	0	0	0	1	0	0	0	0	0	0	0	1
Cluster11942	Acidobact	Acidobacteria_Gp3				0	0	0	0	0	0	0	0	0	1	0	0	1
Cluster11944	Proteobac	Alphaproteobacteria				0	0	0	0	0	0	0	1	0	0	0	0	1
Cluster11947;size=1						0	0	0	0	0	0	0	0	0	1	0	0	1

Cluster11953;size=1						0	0	0	0	1	0	0	0	0	0	0	0	1
Cluster11956	Proteobac	Alphap	Rhizobiales			0	0	0	0	0	0	1	0	0	0	0	0	1
Cluster11974	Proteobac	Alphap	Rhizobiales			0	0	0	0	1	0	0	0	0	0	0	0	1
Cluster11975;size=1						0	0	0	0	1	0	0	0	0	0	0	0	1
Cluster11977;size=3						0	0	0	0	1	0	0	0	0	0	0	0	1
Cluster11981	Proteobac	Betapr	Burkl	Oxalobacteraceae		0	0	0	0	1	0	0	0	0	0	0	0	1
Cluster11991	Acidobact	Acidobacteria_Gp10			Gp10	0	0	0	0	0	0	0	1	0	0	0	0	1
Cluster11998	Acidobact	Acidobacteria_Gp1			Gp1	0	0	0	0	1	0	0	0	0	0	0	0	1
Cluster12000	Armatimc	Armati	Arma	Armatir	Armatimonas/	0	0	0	0	1	0	0	0	0	0	0	0	1
Cluster12002	Proteobac	Deltaproteobacteria				0	0	0	0	0	0	0	0	0	1	0	0	1
Cluster12003	Acidobact	Acidobacteria_Gp3				0	0	0	0	0	0	0	1	0	0	0	0	1
Cluster12012	Proteobac	Betapr	Burkl	Burkhol	Burkholderia	0	0	0	0	0	0	0	1	0	0	0	0	1
Cluster12017	Verrucom	Verruc	Verru	Verrucomicrobiaceae		0	0	0	0	0	0	0	1	0	0	0	0	1
Cluster12025	Acidobact	Acidobacteria_Gp1			Gp1	0	0	0	0	0	0	1	0	0	0	0	0	1
Cluster12033	Proteobac	Betapr	Burkholderiales			0	0	0	0	0	0	1	0	0	0	0	0	1
Cluster12045	Acidobact	Acidobacteria_Gp6			Gp6	0	1	0	0	0	0	0	0	0	0	0	0	1
Cluster12049	Gemmatir	Gemm	Gem	Gemma	Gemmatimona	0	0	0	0	1	0	0	0	0	0	0	0	1
Cluster12050	Proteobac	Alphap	Rhizc	Bradyrhizobiaceae		0	0	0	0	1	0	0	0	0	0	0	0	1
Cluster12055	Proteobac	Alphaproteobacteria				0	0	0	0	0	0	0	0	0	1	0	0	1
Cluster12061	Acidobact	Acidobacteria_Gp2			Gp2	0	0	0	0	0	0	1	0	0	0	0	0	1
Cluster12062	Bacteroid	Sphing	Sphir	Chitinophagaceae		0	0	0	0	1	0	0	0	0	0	0	0	1
Cluster12063	Proteobacteria					0	0	0	0	0	0	0	0	0	1	0	0	1
Cluster12067	Acidobact	Acidobacteria_Gp2			Gp2	0	0	0	0	0	0	0	1	0	0	0	0	1
Cluster12069	Proteobac	Deltaproteobacteria				0	0	0	0	0	1	0	0	0	0	0	0	1
Cluster12071	Actinobac	Actinol	Actinomycetales			0	0	0	0	0	1	0	0	0	0	0	0	1
Cluster12079	Proteobac	Deltap	Myxc	Polyangiaceae		0	0	1	0	0	0	0	0	0	0	0	0	1
Cluster12097;size=2						0	0	1	0	0	0	0	0	0	0	0	0	1
Cluster12102	Verrucom	Subdivision3			Subdivision3_g	0	0	0	0	0	0	1	0	0	0	0	0	1
Cluster12114;size=5						0	0	0	1	0	0	0	0	0	0	0	0	1

Cluster12124	Gemmatii	Gemm	Gemi	Gemma	Gemmatimona	0	0	0	0	0	0	1	0	0	0	0	0	1
Cluster12129	Proteobac	Alphaproteobacteria				0	0	0	0	0	1	0	0	0	0	0	0	1
Cluster12130	Proteobac	Alphap	Rhodospirillales			0	0	0	0	0	1	0	0	0	0	0	0	1
Cluster12145	Acidobact	Acidobacteria_Gp1			Acidobacterium	1	0	0	0	0	0	0	0	0	0	0	0	1
Cluster12148	Proteobac	Gammaproteobacteria				0	0	0	0	0	0	0	0	1	0	0	0	1
Cluster12157	Acidobact	Acidobacteria_Gp1			Gp1	0	0	0	0	0	0	0	0	1	0	0	0	1
Cluster12161	TM7				TM7_genera_i	0	0	0	0	0	0	0	0	1	0	0	0	1
Cluster12172	Acidobact	Acidobacteria_Gp1			Edaphobacter	0	0	0	0	0	1	0	0	0	0	0	0	1
Cluster12188	Acidobact	Acidobacteria_Gp1			Gp1	0	0	0	0	0	0	0	1	0	0	0	0	1
Cluster12191;size=2						0	0	0	0	0	0	0	1	0	0	0	0	1
Cluster12194	Proteobac	Betapr	Burkholderiales			0	0	0	0	0	0	0	1	0	0	0	0	1
Cluster12201	Acidobact	Acidobacteria_Gp3			Gp3	0	0	0	0	1	0	0	0	0	0	0	0	1
Cluster12208	Proteobac	Deltap	Myxococcales			0	0	0	0	0	1	0	0	0	0	0	0	1
Cluster12232;size=1						0	0	0	0	0	0	1	0	0	0	0	0	1
Cluster12242	Proteobac	Alphaproteobacteria				0	0	0	0	0	0	1	0	0	0	0	0	1
Cluster12247	Acidobact	Acidobacteria_Gp1				0	0	0	0	0	0	0	0	0	0	0	1	1
Cluster12248	Verrucom	Opituta	Opitu	Opituta	Opitutus	0	0	0	0	0	1	0	0	0	0	0	0	1
Cluster12249	Acidobact	Acidobacteria_Gp1			Gp1	0	0	0	0	0	1	0	0	0	0	0	0	1
Cluster12257;size=1						0	0	0	0	0	0	0	1	0	0	0	0	1
Cluster12264	Proteobac	Alphap	Rhod	Rhodospirillaceae		0	1	0	0	0	0	0	0	0	0	0	0	1
Cluster12268	Acidobact	Acidobacteria_Gp3				0	0	0	0	0	0	0	1	0	0	0	0	1
Cluster12277	Bacteroid	Sphing	Sphir	Cytophagaceae		0	0	0	0	0	0	0	1	0	0	0	0	1
Cluster12299;size=1						0	0	0	0	0	0	1	0	0	0	0	0	1
Cluster12301	Proteobac	Gammaproteobacteria				0	0	0	0	0	0	0	0	1	0	0	0	1
Cluster12305	Proteobac	Alphap	Rhizobiales			0	0	0	0	0	0	0	0	1	0	0	0	1
Cluster12308	Gemmatii	Gemm	Gemi	Gemma	Gemmatimona	0	0	0	0	0	0	0	0	1	0	0	0	1
Cluster12311;size=8						0	0	0	0	0	0	1	0	0	0	0	0	1
Cluster12313	Actinobac	Actinol	Actinomycetales			0	0	0	0	0	1	0	0	0	0	0	0	1
Cluster12322	Acidobact	Acidobacteria_Gp7			Gp7	0	1	0	0	0	0	0	0	0	0	0	0	1

Cluster12328	Proteobac	Deltap	Myxc	Polyangiaceae		0	0	0	0	0	0	0	1	0	0	0	0	1
Cluster12331;size=2						0	0	0	0	0	0	0	0	0	0	0	1	1
Cluster12342	Proteobac	Alphap	Rhizc	Methylc	Microvirga	0	0	0	0	0	0	0	1	0	0	0	0	1
Cluster12344	Proteobac	Gamm	Legio	Coxiella	Aquicella	0	0	0	0	0	0	1	0	0	0	0	0	1
Cluster12350	Proteobac	Alphap	Rhodospirillales			0	0	1	0	0	0	0	0	0	0	0	0	1
Cluster12351	Acidobact	Acidobacteria_Gp1			Granulicella	0	0	0	0	1	0	0	0	0	0	0	0	1
Cluster12356	Proteobac	Alphap	Caulc	Caulobа	Phenylobacter	1	0	0	0	0	0	0	0	0	0	0	0	1
Cluster12361	Acidobact	Acidobacteria_Gp1				0	0	1	0	0	0	0	0	0	0	0	0	1
Cluster12377	Proteobac	Alphap	Rhizobiales			1	0	0	0	0	0	0	0	0	0	0	0	1
Cluster12381	Proteobac	Deltaproteobacteria				0	0	1	0	0	0	0	0	0	0	0	0	1
Cluster12384	Proteobac	Alphap	Rhod	Acetobacteraceae		0	0	0	0	0	0	0	0	0	0	0	1	1
Cluster12391	Proteobac	Alphap	Rhod	Acetoba	Acidocella	0	0	1	0	0	0	0	0	0	0	0	0	1
Cluster12398	Proteobac	Alphap	Caulc	Caulobа	Caulobacter	0	1	0	0	0	0	0	0	0	0	0	0	1
Cluster12401	Proteobacteria					0	0	1	0	0	0	0	0	0	0	0	0	1
Cluster12403	Proteobac	Alphap	Rhizobiales			0	1	0	0	0	0	0	0	0	0	0	0	1
Cluster12432	Acidobact	Acidobacteria_Gp3			Bryobacter	0	0	1	0	0	0	0	0	0	0	0	0	1
Cluster12437	Proteobac	Alphap	Rhod	Rhodospirillaceae		0	0	1	0	0	0	0	0	0	0	0	0	1
Cluster12442	Acidobact	Acidobacteria_Gp1			Gp1	0	0	1	0	0	0	0	0	0	0	0	0	1
Cluster12446	Proteobac	Alphap	Rhizc	Bradyrh	Agromonas	0	0	0	0	0	0	0	0	0	0	0	1	1
Cluster12453	Proteobac	Betaproteobacteria				0	1	0	0	0	0	0	0	0	0	0	0	1
Cluster12454	Acidobact	Acidobacteria_Gp3			Gp3	0	0	0	0	0	0	0	0	0	0	1	0	1
Cluster12464;size=6						0	0	0	0	0	0	0	0	0	0	0	1	1
Cluster12471	Proteobac	Alphap	Rhod	Rhodospirillaceae		0	1	0	0	0	0	0	0	0	0	0	0	1
Cluster12476	Acidobact	Acidobacteria_Gp2			Gp2	0	0	0	0	0	0	0	0	0	0	1	0	1
Cluster12478	Proteobac	Alphaproteobacteria				0	0	1	0	0	0	0	0	0	0	0	0	1
Cluster12479	Proteobac	Alphap	Rhizc	Xanthol	Pseudolabrys	0	0	0	0	0	0	0	0	0	0	1	0	1
Cluster12482	Acidobact	Acidobacteria_Gp2			Gp2	0	0	0	0	0	0	0	0	0	0	1	0	1
Cluster12496	Acidobact	Acidobacteria_Gp10			Gp10	0	0	0	0	0	0	0	1	0	0	0	0	1
Cluster12501	Acidobact	Acidobacteria_Gp1				0	0	1	0	0	0	0	0	0	0	0	0	1

Cluster12510	Acidobacteria					0	0	0	0	0	0	0	0	1	0	0	0	1
Cluster12513	Acidobact	Acidobacteria_Gp5			Gp5	0	0	0	0	0	0	0	0	0	0	1	0	1
Cluster12514	Actinobac	Actinol	Actinomycetales			0	0	0	0	0	0	0	0	0	0	0	1	1
Cluster12519	Acidobact	Acidobacteria_Gp1			Gp1	0	0	1	0	0	0	0	0	0	0	0	0	1
Cluster12523	Proteobac	Alphap	Rhodospirillales			0	1	0	0	0	0	0	0	0	0	0	0	1
Cluster12535	Proteobac	Betapr	Burkl	Burkhol	Burkholderia	0	0	1	0	0	0	0	0	0	0	0	0	1
Cluster12536	Proteobac	Alphaproteobacteria				0	0	1	0	0	0	0	0	0	0	0	0	1
Cluster12543	Proteobac	Betaproteobacteria				0	0	0	0	0	0	0	0	0	0	0	1	1
Cluster12547	Proteobac	Gammaproteobacteria				0	0	0	0	0	0	0	0	0	0	0	1	1
Cluster12550	Actinobac	Actinol	Solirı	Solirubr	Solirubrobacte	0	0	1	0	0	0	0	0	0	0	0	0	1
Cluster12551	Actinobac	Actinol	Solirı	Conexib	Conexibacter	0	0	1	0	0	0	0	0	0	0	0	0	1
Cluster12562	Proteobac	Deltap	Myxococcales			0	0	0	0	0	0	0	0	0	0	1	0	1
Cluster12567;size=1						0	0	0	0	0	0	0	0	0	0	1	0	1
Cluster12575;size=1						0	0	1	0	0	0	0	0	0	0	0	0	1
Cluster12576	Acidobact	Acidobacteria_Gp2			Gp2	0	1	0	0	0	0	0	0	0	0	0	0	1
Cluster12582	Proteobac	Alphap	Rhizc	Bradyrhizobiaceae		0	0	0	0	0	0	0	0	0	0	0	1	1
Cluster12587	Proteobac	Alphap	Rhodospirillales			0	0	0	1	0	0	0	0	0	0	0	0	1
Cluster12595	Acidobacteria					0	0	0	0	0	0	0	0	0	0	0	1	1
Cluster12597;size=1						0	0	0	1	0	0	0	0	0	0	0	0	1
Cluster12600;size=3						0	0	0	1	0	0	0	0	0	0	0	0	1
Cluster12619	Actinobac	Actinol	Solirı	Solirubr	Solirubrobacte	0	0	0	1	0	0	0	0	0	0	0	0	1
Cluster12629	Acidobact	Acidobacteria_Gp3			Gp3	0	0	0	0	0	0	0	0	0	0	1	0	1
Cluster12631	Acidobact	Acidobacteria_Gp1				0	0	0	0	0	0	0	0	0	0	0	1	1
Cluster12632;size=1						0	0	0	0	0	0	0	0	0	0	0	1	1
Cluster12634	Acidobact	Acidobacteria_Gp1			Gp1	0	0	0	1	0	0	0	0	0	0	0	0	1
Cluster12638	Proteobac	Alphaproteobacteria				0	0	0	0	0	0	0	0	0	0	0	1	1
Cluster12639	Acidobact	Acidobacteria_Gp1				0	0	0	1	0	0	0	0	0	0	0	0	1
Cluster12641;size=1						0	0	0	0	0	0	0	0	0	0	0	1	1
Cluster12643	Acidobact	Acidobacteria_Gp1			Gp1	0	0	0	0	0	0	0	0	0	0	0	1	1

Cluster12644	Acidobact	Acidobacteria_Gp2			Gp2	0	0	0	0	0	0	1	0	0	0	0	0	1
Cluster12647	Acidobact	Acidobacteria_Gp1			Gp1	0	0	0	0	0	0	0	0	0	0	0	1	1
Cluster12652	Proteobac	Alphaproteobacteria				0	0	1	0	0	0	0	0	0	0	0	0	1
Cluster12654;size=2						0	0	0	0	0	0	1	0	0	0	0	0	1
Cluster12656	Proteobac	Gammaproteobacteria				1	0	0	0	0	0	0	0	0	0	0	0	1
Cluster12673	Verrucom	Subdivision3			Subdivision3_g	0	0	0	0	0	0	0	0	0	0	1	0	1
Cluster12678	Proteobac	Alphap	Rhodospirillales			0	0	0	0	0	0	0	0	1	0	0	0	1
Cluster12684	Armatimonadetes				Armatimonade	0	0	1	0	0	0	0	0	0	0	0	0	1
Cluster12685	Actinobac	Actinol	Actinomycetales			0	0	0	0	1	0	0	0	0	0	0	0	1
Cluster12694	Proteobac	Alphap	Rhizo	Xanthol	Pseudolabrys	0	0	0	0	0	0	0	0	0	0	0	1	1
Cluster12695;size=1						0	0	1	0	0	0	0	0	0	0	0	0	1
Cluster12702;size=1						0	1	0	0	0	0	0	0	0	0	0	0	1
Cluster12704	Bacteroid	Sphing	Sphir	Chitinophagaceae		0	0	0	0	0	0	0	0	0	0	0	1	1
Cluster12724;size=1						0	0	0	0	0	0	0	0	0	0	1	0	1
Cluster12730	Bacteroid	Sphing	Sphir	Chitinop	Sediminibacter	0	0	0	0	0	0	0	0	0	0	0	1	1
Cluster12733	Acidobact	Acidobacteria_Gp2			Gp2	0	0	0	0	0	0	0	0	0	0	0	1	1
Cluster12739	Proteobacteria					0	0	0	0	0	0	0	0	0	0	0	1	1
Cluster12741	Proteobacteria					0	1	0	0	0	0	0	0	0	0	0	0	1
Cluster12744;size=3						0	0	0	0	0	0	0	0	0	0	1	0	1
Cluster12754	Actinobac	Actinol	Actin	Microbacteriaceae		0	0	1	0	0	0	0	0	0	0	0	0	1
Cluster12756	Acidobact	Acidobacteria_Gp3			Gp3	0	0	1	0	0	0	0	0	0	0	0	0	1
Cluster12758	Proteobac	Alphap	Sphir	Sphingc	Sphingomonas	0	0	0	0	0	0	0	0	0	0	0	1	1
Cluster12767	Proteobac	Alphap	Rhodospirillales			0	0	0	0	0	0	1	0	0	0	0	0	1
Cluster12768	Proteobac	Betapr	Burkl	Oxalobacteraceae		0	0	0	0	0	0	0	0	0	0	0	1	1
Cluster12772	Acidobact	Acidobacteria_Gp3				0	0	1	0	0	0	0	0	0	0	0	0	1
Cluster12775	Proteobac	Betapr	Burkl	Burkhol	Burkholderia	0	0	0	0	0	0	0	0	0	0	0	1	1
Cluster12780	Acidobact	Acidobacteria_Gp1			Gp1	0	0	1	0	0	0	0	0	0	0	0	0	1
Cluster12784	Acidobact	Acidobacteria_Gp1			Gp1	0	0	1	0	0	0	0	0	0	0	0	0	1
Cluster12787	Acidobacteria					0	0	0	0	0	0	0	0	0	0	0	1	1

Cluster12802	Acidobact	Acidobacteria_Gp1			Granulicella	0	0	1	0	0	0	0	0	0	0	0	0	1
Cluster12804	Acidobact	Acidobacteria_Gp1			Gp1	0	1	0	0	0	0	0	0	0	0	0	0	1
Cluster12810	Acidobact	Acidobacteria_Gp1			Acidobacteriu	1	0	0	0	0	0	0	0	0	0	0	0	1
Cluster12820	Proteobacteria					0	0	0	0	0	0	0	0	0	0	1	0	1
Cluster12822	Acidobact	Acidobacteria_Gp1			Edaphobacter	1	0	0	0	0	0	0	0	0	0	0	0	1
Cluster12824	Acidobact	Acidobacteria_Gp1			Gp1	0	0	0	0	0	0	0	0	0	0	1	0	1
Cluster12825;size=1						0	0	1	0	0	0	0	0	0	0	0	0	1
Cluster12831	Acidobact	Acidobacteria_Gp2			Gp2	0	0	0	1	0	0	0	0	0	0	0	0	1
Cluster12835;size=2						0	0	0	1	0	0	0	0	0	0	0	0	1
Cluster12848	Proteobac	Deltap	Myxococcales			0	0	1	0	0	0	0	0	0	0	0	0	1
Cluster12849;size=1						0	0	0	0	0	0	0	0	0	0	0	1	1
Cluster12857	Proteobac	Betapr	Burkl	Burkhol	Burkholderia	0	0	0	0	0	0	0	0	0	0	1	0	1
Cluster12860	Proteobac	Betapr	Burkl	Oxalobacteraceae		0	0	0	0	0	0	0	0	0	0	1	0	1
Cluster12864	Proteobac	Alphap	Rhizo	Xanthob	Labrys	0	0	0	0	0	0	1	0	0	0	0	0	1
Cluster12873	Acidobact	Acidobacteria_Gp1			Gp1	0	1	0	0	0	0	0	0	0	0	0	0	1
Cluster12897	Acidobact	Acidobacteria_Gp2			Gp2	0	0	0	0	0	0	0	1	0	0	0	0	1
Cluster12901	Acidobact	Acidobacteria_Gp1				0	0	0	0	0	0	0	0	0	0	0	1	1
Cluster12903	Acidobact	Acidobacteria_Gp1				0	0	1	0	0	0	0	0	0	0	0	0	1
Cluster12904	Actinobac	Actinol	Solirubrobacterales			0	0	0	0	0	0	0	0	0	1	0	0	1
Cluster12913	Acidobact	Acidobacteria_Gp1			Gp1	0	0	0	0	0	0	0	0	0	0	0	1	1
Cluster12916	Acidobact	Acidobacteria_Gp3			Gp3	0	0	1	0	0	0	0	0	0	0	0	0	1
Cluster12922	Proteobacteria					0	0	1	0	0	0	0	0	0	0	0	0	1
Cluster12923	Acidobact	Acidobacteria_Gp1				0	0	1	0	0	0	0	0	0	0	0	0	1
Cluster12925	Acidobact	Acidobacteria_Gp2			Gp2	0	0	0	0	0	0	0	0	0	0	0	1	1
Cluster12926	Acidobact	Acidobacteria_Gp1			Gp1	0	0	0	0	0	0	1	0	0	0	0	0	1
Cluster12928	Acidobact	Acidobacteria_Gp3			Bryobacter	0	1	0	0	0	0	0	0	0	0	0	0	1
Cluster12932	Verrucom	Subdivision3			Subdivision3_g	0	0	0	0	0	0	0	0	0	0	1	0	1
Cluster12933	Proteobac	Alphap	Rhizo	Rhizobia	Bauldia	0	0	0	0	0	0	0	0	0	0	0	1	1
Cluster12935	Proteobac	Alphap	Rhizobiales			0	0	0	0	0	0	0	0	0	0	1	0	1

Cluster12937	Acidobact	Acidobacteria_Gp3		Gp3	0	0	0	0	0	0	0	0	0	0	0	1	1
Cluster12941	Proteobac	Gamm	Xantl Sinobac	Steroidobacter	0	0	0	0	0	0	0	0	0	0	1	0	1
Cluster12942	Acidobact	Acidobacteria_Gp1		Gp1	1	0	0	0	0	0	0	0	0	0	0	0	1
Cluster12943;size=1					0	0	1	0	0	0	0	0	0	0	0	0	1
Cluster12947	Acidobact	Acidobacteria_Gp2		Gp2	0	0	1	0	0	0	0	0	0	0	0	0	1
Cluster12949	Proteobac	Alphap	Rhizobiales		0	0	0	0	0	0	0	0	0	0	1	0	1
Cluster12951	Acidobact	Acidobacteria_Gp2		Gp2	1	0	0	0	0	0	0	0	0	0	0	0	1
Cluster12953	Acidobact	Acidobacteria_Gp3			1	0	0	0	0	0	0	0	0	0	0	0	1
Cluster12962	Acidobact	Acidobacteria_Gp2		Gp2	0	0	0	0	0	0	0	0	0	0	0	1	1
Cluster12963	Acidobact	Acidobacteria_Gp1		Gp1	0	0	1	0	0	0	0	0	0	0	0	0	1
Cluster12964	Acidobact	Acidobacteria_Gp1		Gp1	0	0	0	0	0	0	0	0	0	0	0	1	1
Cluster12975	Acidobact	Acidobacteria_Gp2		Gp2	0	1	0	0	0	0	0	0	0	0	0	0	1
Cluster12980;size=5					0	0	0	0	0	0	0	0	0	0	1	0	1
Cluster13015;size=1					0	0	0	0	0	0	0	0	0	0	0	1	1
Cluster13020	Acidobact	Acidobacteria_Gp3		Gp3	0	0	0	0	0	0	0	0	0	0	1	0	1
Cluster13028	Proteobac	Betaproteobacteria			0	0	0	0	0	0	0	0	0	0	0	1	1
Cluster13034;size=4					0	0	0	0	0	0	0	0	0	0	1	0	1
Cluster13035	Proteobacteria				0	0	0	0	0	0	0	0	0	0	1	0	1
Cluster13038	Proteobac	Deltap	Myxc Cystobacteraceae		0	0	1	0	0	0	0	0	0	0	0	0	1
Cluster13044	Acidobact	Acidobacteria_Gp1		Gp1	0	0	1	0	0	0	0	0	0	0	0	0	1
Cluster13046	Proteobac	Alphap	Rhizc Hyphon	Rhodoplanes	0	1	0	0	0	0	0	0	0	0	0	0	1
Cluster13049	Acidobact	Acidobacteria_Gp1			0	0	1	0	0	0	0	0	0	0	0	0	1
Cluster13053;size=3					0	0	0	1	0	0	0	0	0	0	0	0	1
Cluster13065	Acidobact	Acidobacteria_Gp1			0	0	1	0	0	0	0	0	0	0	0	0	1
Cluster13067	Acidobact	Acidobacteria_Gp2		Gp2	0	0	1	0	0	0	0	0	0	0	0	0	1
Cluster13077	Proteobac	Alphap	Rhod Rhodospirillaceae		0	0	1	0	0	0	0	0	0	0	0	0	1
Cluster13078	Proteobac	Alphap	Rhod Acetobacteraceae		0	0	0	1	0	0	0	0	0	0	0	0	1
Cluster13088	Acidobact	Acidobacteria_Gp2		Gp2	0	1	0	0	0	0	0	0	0	0	0	0	1
Cluster13094;size=3					0	0	1	0	0	0	0	0	0	0	0	0	1

Cluster13099	Verrucom	Opituta	Opitu	Opituta	Opitutus	1	0	0	0	0	0	0	0	0	0	0	0	1
Cluster13113	Proteobac	Alphap	Rhizo	Bradyrh	Agromonas	0	0	1	0	0	0	0	0	0	0	0	0	1
Cluster13115;size=1						0	0	1	0	0	0	0	0	0	0	0	0	1
Cluster13125	Proteobac	Deltap	Myxo	Polyangiaceae		0	0	1	0	0	0	0	0	0	0	0	0	1
Cluster13133	Proteobac	Gamm	Xantl	Sinobacteraceae		0	0	1	0	0	0	0	0	0	0	0	0	1
Cluster13134	Proteobac	Alphaproteobacteria				0	0	1	0	0	0	0	0	0	0	0	0	1
Cluster13139	Verrucomicrobia					0	0	0	0	0	0	0	1	0	0	0	0	1
Cluster13140;size=1						0	0	1	0	0	0	0	0	0	0	0	0	1
Cluster13151;size=5						0	0	0	0	0	0	0	0	0	0	1	0	1
Cluster13155	Acidobact	Acidobacteria_Gp2			Gp2	0	0	0	0	0	0	0	1	0	0	0	0	1
Cluster13159	Proteobac	Alphap	Rhod	Acetobacteraceae		0	0	1	0	0	0	0	0	0	0	0	0	1
Cluster13163	Proteobac	Alphap	Rhodospirillales			0	1	0	0	0	0	0	0	0	0	0	0	1
Cluster13164	Acidobact	Acidobacteria_Gp1			Gp1	1	0	0	0	0	0	0	0	0	0	0	0	1
Cluster13165	Acidobact	Acidobacteria_Gp2			Gp2	0	0	0	0	0	0	0	0	0	0	0	1	1
Cluster13166	Proteobac	Betaproteobacteria				1	0	0	0	0	0	0	0	0	0	0	0	1
Cluster13167	OD1				OD1_genera_i	0	1	0	0	0	0	0	0	0	0	0	0	1
Cluster13169	Armatimo	Armati	Arma	Armatir	Armatimonas/	0	0	1	0	0	0	0	0	0	0	0	0	1
Cluster13176	Proteobac	Gamm	Xantl	Xanthor	Frateuria	0	0	0	1	0	0	0	0	0	0	0	0	1
Cluster13180	Acidobact	Acidobacteria_Gp2			Gp2	0	0	0	0	0	0	0	1	0	0	0	0	1
Cluster13188	Proteobac	Alphap	Caulo	Cauloba	Phenylobacter	1	0	0	0	0	0	0	0	0	0	0	0	1
Cluster13192	Verrucom	Opituta	Opitu	Opituta	Opitutus	0	0	0	0	0	0	0	0	0	0	1	0	1
Cluster13193	Verrucom	Subdivision3			Subdivision3_g	0	0	1	0	0	0	0	0	0	0	0	0	1
Cluster13198	Acidobact	Acidobacteria_Gp6			Gp6	1	0	0	0	0	0	0	0	0	0	0	0	1
Cluster13199	Proteobac	Deltaproteobacteria				0	0	0	1	0	0	0	0	0	0	0	0	1
Cluster13200	Proteobac	Betapr	Burkholderiales			1	0	0	0	0	0	0	0	0	0	0	0	1
Cluster13202	Acidobact	Acidobacteria_Gp3				0	0	0	0	0	0	0	0	0	0	0	1	1
Cluster13207	Actinobac	Actinol	Acidi	Acidimi	Aciditerrimona	0	0	1	0	0	0	0	0	0	0	0	0	1
Cluster13219	Proteobac	Alphap	Rhizobiales			1	0	0	0	0	0	0	0	0	0	0	0	1
Cluster13220	Proteobac	Betapr	Burkl	Burkhol	Burkholderia	1	0	0	0	0	0	0	0	0	0	0	0	1

Cluster13223;size=1						0	0	0	0	0	0	0	0	0	0	0	1	1
Cluster13225	Armatimc	Armati	Arma	Armatir	Armatimonas/	0	0	1	0	0	0	0	0	0	0	0	0	1
Cluster13233	Bacteroid	Sphing	Sphir	Chitinophagaceae		0	0	0	0	0	0	0	0	0	1	0	0	1
Cluster13234	Proteobac	Alphap	Rhizobiales			0	1	0	0	0	0	0	0	0	0	0	0	1
Cluster13236	Acidobact	Acidobacteria_Gp1			Gp1	0	0	0	1	0	0	0	0	0	0	0	0	1
Cluster13238	Proteobac	Betapr	Burkl	Burkhol	Burkholderia	0	0	0	0	0	0	0	1	0	0	0	0	1
Cluster13244	Planctomy	Plancto	Planc	Planctomycetaceae		0	0	0	0	0	0	0	0	0	0	1	0	1
Cluster13246	Acidobact	Acidobacteria_Gp1				0	0	0	0	0	0	0	0	0	0	1	0	1
Cluster13247	Planctomy	Plancto	Planc	Planctor	Singulisphaera	0	0	0	0	0	0	0	0	0	0	0	1	1
Cluster13251	Proteobacteria					1	0	0	0	0	0	0	0	0	0	0	0	1
Cluster13262	Acidobact	Acidobacteria_Gp1			Gp1	0	0	0	0	0	0	0	1	0	0	0	0	1
Cluster13269	Proteobacteria					0	0	0	0	0	0	0	0	0	0	1	0	1
Cluster13274	Acidobact	Acidobacteria_Gp3			Gp3	0	0	0	0	0	0	0	1	0	0	0	0	1
Cluster13289	Proteobac	Betapr	Burkl	Oxalobacteraceae		0	0	1	0	0	0	0	0	0	0	0	0	1
Cluster13295	Acidobact	Acidobacteria_Gp2			Gp2	0	0	1	0	0	0	0	0	0	0	0	0	1
Cluster13296	Proteobac	Alphap	Rhizobiales			0	0	0	0	0	0	0	0	0	0	0	1	1
Cluster13302	Proteobac	Alphap	Rhod	Rhodos	Magnetospirill	0	0	0	0	0	0	0	0	0	0	1	0	1
Cluster13309	Acidobact	Acidobacteria_Gp2			Gp2	0	0	0	1	0	0	0	0	0	0	0	0	1
Cluster13320;size=1						0	1	0	0	0	0	0	0	0	0	0	0	1
Cluster13324	Proteobac	Deltaproteobacteria				0	1	0	0	0	0	0	0	0	0	0	0	1
Cluster13330	Acidobact	Acidobacteria_Gp1			Gp1	1	0	0	0	0	0	0	0	0	0	0	0	1
Cluster13331;size=1						0	0	0	1	0	0	0	0	0	0	0	0	1
Cluster13334	Proteobac	Deltap	Myxococcales			0	0	1	0	0	0	0	0	0	0	0	0	1
Cluster13335;size=2						0	0	1	0	0	0	0	0	0	0	0	0	1
Cluster13342	Proteobac	Alphap	Rhizobiales			0	0	1	0	0	0	0	0	0	0	0	0	1
Cluster13344	Acidobact	Acidobacteria_Gp2			Gp2	1	0	0	0	0	0	0	0	0	0	0	0	1
Cluster13345	Proteobac	Deltaproteobacteria				1	0	0	0	0	0	0	0	0	0	0	0	1
Cluster13349	Proteobac	Alphap	Rhizc	Methylc	Microvirga	0	0	1	0	0	0	0	0	0	0	0	0	1
Cluster13351;size=1						0	0	0	0	0	0	0	0	0	0	0	1	1

Cluster13352	Acidobacteria					0	1	0	0	0	0	0	0	0	0	0	0	1
Cluster13356	Acidobact	Acidobacteria_Gp2			Gp2	0	0	0	0	0	0	0	0	0	0	0	1	1
Cluster13358	Proteobac	Alphap	Rhizobiales			0	0	0	0	0	0	0	0	0	0	0	1	1
Cluster13360	Proteobacteria					0	1	0	0	0	0	0	0	0	0	0	0	1
Cluster13362	Proteobac	Betapr	Burkholderiales			0	0	1	0	0	0	0	0	0	0	0	0	1
Cluster13365	Verrucom	Subdivision3			Subdivision3_g	1	0	0	0	0	0	0	0	0	0	0	0	1
Cluster13375	Proteobac	Gammaproteobacteria				0	0	0	0	0	0	0	0	0	0	1	0	1
Cluster13376;size=1						0	0	0	0	0	0	0	0	0	0	0	1	1
Cluster13381	Actinobac	Actinob	Actinomycetales			1	0	0	0	0	0	0	0	0	0	0	0	1
Cluster13382	Proteobac	Alphaproteobacteria				0	0	1	0	0	0	0	0	0	0	0	0	1
Cluster13383	Proteobac	Alphap	Rhod	Acetoba	Acidisphaera	0	0	0	1	0	0	0	0	0	0	0	0	1
Cluster13387	Proteobac	Gammaproteobacteria				0	0	0	0	0	0	0	0	0	0	0	1	1
Cluster13390	Actinobac	Actinob	Actin	Streptom	Streptomyces	0	0	0	0	0	0	0	0	0	0	0	1	1
Cluster13394	Acidobact	Acidobacteria_Gp1				1	0	0	0	0	0	0	0	0	0	0	0	1
Cluster13401;size=2						0	0	1	0	0	0	0	0	0	0	0	0	1
Cluster13405	Verrucomicrobia					0	0	1	0	0	0	0	0	0	0	0	0	1
Cluster13407	Actinobac	Actinob	Actin	Nocardiaceae		1	0	0	0	0	0	0	0	0	0	0	0	1
Cluster13409	Proteobac	Alphap	Rhizo	Methylo	Methylosinus	0	0	1	0	0	0	0	0	0	0	0	0	1
Cluster13416	Proteobac	Betapr	Burkh	Burkhol	Burkholderia	0	0	0	0	0	0	0	0	0	0	1	0	1
Cluster13422	Proteobac	Betapr	Burkh	Burkhol	Burkholderia	1	0	0	0	0	0	0	0	0	0	0	0	1
Cluster13423	Proteobac	Gammaproteobacteria				0	0	1	0	0	0	0	0	0	0	0	0	1
Cluster13426	Firmicutes	Bacilli	Bacillales			1	0	0	0	0	0	0	0	0	0	0	0	1
Cluster13430;size=1						0	0	0	0	0	0	0	0	0	0	0	1	1
Cluster13439	Acidobact	Acidobacteria_Gp5			Gp5	0	0	0	0	0	0	0	0	0	0	0	1	1
Cluster13440	Acidobact	Acidobacteria_Gp3			Gp3	0	0	1	0	0	0	0	0	0	0	0	0	1
Cluster13441	Acidobact	Acidobacteria_Gp1				0	0	1	0	0	0	0	0	0	0	0	0	1
Cluster13445	Acidobact	Acidobacteria_Gp1			Gp1	1	0	0	0	0	0	0	0	0	0	0	0	1
Cluster13447	Proteobac	Alphap	Alphaproteob		Rhizomicrobiu	0	0	1	0	0	0	0	0	0	0	0	0	1
Cluster13449	Acidobact	Acidobacteria_Gp3			Gp3	0	0	1	0	0	0	0	0	0	0	0	0	1

Cluster13458	Acidobact	Acidobacteria_Gp10			Gp10	0	0	1	0	0	0	0	0	0	0	0	0	1
Cluster13463	Verrucom	Subdivision3			Subdivision3_g	0	0	0	0	0	0	0	0	0	1	0	0	1
Cluster13471	Acidobact	Acidobacteria_Gp3			Gp3	0	0	1	0	0	0	0	0	0	0	0	0	1
Cluster13477	Proteobac	Deltaproteobacteria				1	0	0	0	0	0	0	0	0	0	0	0	1
Cluster13478	Verrucom	Opituta	Opitu	Opituta	Opitutus	1	0	0	0	0	0	0	0	0	0	0	0	1
Cluster13480	Acidobact	Acidobacteria_Gp1			Acidobacterium	0	0	1	0	0	0	0	0	0	0	0	0	1
Cluster13485	Proteobac	Alphap	Rhizobiales			1	0	0	0	0	0	0	0	0	0	0	0	1
Cluster13486;size=1						1	0	0	0	0	0	0	0	0	0	0	0	1
Cluster13492	Verrucom	Spartobacteria			Spartobacteria	0	0	0	1	0	0	0	0	0	0	0	0	1
Cluster13493	Acidobact	Acidobacteria_Gp1			Acidobacterium	1	0	0	0	0	0	0	0	0	0	0	0	1
Cluster13495	Acidobact	Acidobacteria_Gp1			Gp1	0	0	0	0	0	0	0	0	0	0	0	1	1
Cluster13501	Acidobact	Acidobacteria_Gp1			Edaphobacter	0	1	0	0	0	0	0	0	0	0	0	0	1
Cluster13509	Firmicutes					0	0	1	0	0	0	0	0	0	0	0	0	1
Cluster13516	Proteobac	Alphap	Caulo	Caulobacteraceae		0	0	0	0	0	0	1	0	0	0	0	0	1
Cluster13520	Actinobac	Actinol	Solirı	Conexib	Conexibacter	0	0	0	1	0	0	0	0	0	0	0	0	1
Cluster13522	Acidobacteria					0	0	0	0	0	0	0	0	0	0	0	1	1
Cluster13524	Acidobact	Acidobacteria_Gp2			Gp2	0	0	1	0	0	0	0	0	0	0	0	0	1
Cluster13528	Proteobac	Betapr	Rhod	Rhodocyclaceae		1	0	0	0	0	0	0	0	0	0	0	0	1
Cluster13529	Acidobact	Acidobacteria_Gp3			Gp3	0	0	0	0	0	0	0	0	0	0	0	1	1
Cluster13534;size=1						0	0	0	0	0	0	0	0	0	0	0	1	1
Cluster13536	Acidobact	Acidobacteria_Gp2			Gp2	0	0	0	0	0	0	0	0	0	0	0	1	1
Cluster13538;size=1						0	0	1	0	0	0	0	0	0	0	0	0	1
Cluster13546;size=1						0	0	0	0	0	0	0	0	0	0	0	1	1
Cluster13551	Acidobact	Acidobacteria_Gp3			Gp3	0	0	0	0	0	1	0	0	0	0	0	0	1
Cluster13553	Proteobac	Alphap	Rhod	Rhodospirillaceae		0	0	0	0	0	0	0	0	0	0	1	0	1
Cluster13555	Acidobact	Acidobacteria_Gp1				1	0	0	0	0	0	0	0	0	0	0	0	1
Cluster13562	Acidobact	Acidobacteria_Gp1				0	0	0	1	0	0	0	0	0	0	0	0	1
Cluster13563;size=1						0	0	1	0	0	0	0	0	0	0	0	0	1
Cluster13568	Proteobac	Alphap	Caulo	Caulobacteraceae		0	1	0	0	0	0	0	0	0	0	0	0	1

Cluster13570	Proteobac	Alphap	Rhizobiales			1	0	0	0	0	0	0	0	0	0	0	0	1
Cluster13583	Proteobac	Alphap	Rhizo	Bradyrhizobiaceae		0	0	0	0	0	0	0	0	0	0	1	0	1
Cluster13588	Proteobac	Betaproteobacteria				0	0	0	1	0	0	0	0	0	0	0	0	1
Cluster13591	Acidobact	Acidobacteria_Gp1			Gp1	0	0	0	0	0	0	0	0	0	0	1	0	1
Cluster13592	Verrucom	Opitut	Opitu	Opitutaceae		1	0	0	0	0	0	0	0	0	0	0	0	1
Cluster13594	Acidobact	Acidobacteria_Gp1			Gp1	0	0	0	0	0	0	0	0	0	0	0	1	1
Cluster13595	Proteobac	Gammaproteobacteria				0	0	0	0	0	0	0	0	0	0	0	1	1
Cluster13598	Gemmatir	Gemm	Gem	Gemma	Gemmatimona	0	0	0	0	0	0	0	0	0	0	0	1	1
Cluster13601	Acidobact	Acidobacteria_Gp1				0	0	1	0	0	0	0	0	0	0	0	0	1
Cluster13611	Proteobac	Deltap	Myxc	Polyang	Chondromyces	0	1	0	0	0	0	0	0	0	0	0	0	1
Cluster13616;size=4						0	0	1	0	0	0	0	0	0	0	0	0	1
Cluster13618	Proteobac	Alphap	Rhodospirillales			1	0	0	0	0	0	0	0	0	0	0	0	1
Cluster13623;size=1						0	0	0	0	0	0	0	0	0	0	1	0	1
Cluster13624	Proteobac	Deltap	Myxc	Kofleria	Kofleria	0	0	0	0	0	0	0	0	0	0	0	1	1
Cluster13625	Proteobac	Alphap	Rhizo	Methylocystaceae		1	0	0	0	0	0	0	0	0	0	0	0	1
Cluster13626	Acidobact	Acidobacteria_Gp3				0	0	1	0	0	0	0	0	0	0	0	0	1
Cluster13627	Acidobact	Acidobacteria_Gp6			Gp6	0	0	0	0	0	0	0	0	0	0	1	0	1
Cluster13628	Proteobac	Betapr	Rhod	Rhodocyclaceae		0	0	0	0	0	0	0	0	0	0	0	1	1
Cluster13631	Acidobact	Acidobacteria_Gp1				0	0	1	0	0	0	0	0	0	0	0	0	1
Cluster13632	Proteobac	Gammaproteobacteria				0	0	0	0	0	0	0	0	0	1	0	0	1
Cluster13633	Elusimicro	Elusim	Elusi	Elusimic	Elusimicrobium	1	0	0	0	0	0	0	0	0	0	0	0	1
Cluster13638	Actinobac	Actinol	Actin	Nocardi	Nocardia	0	0	1	0	0	0	0	0	0	0	0	0	1
Cluster13662	Verrucom	Spartobacteria			Spartobacteria	0	0	0	0	0	0	0	0	0	0	0	1	1
Cluster13663	Proteobac	Alphap	Sphir	Sphingc	Novosphingob	0	0	0	0	1	0	0	0	0	0	0	0	1
Cluster13666	Proteobac	Alphap	Rhod	Acetobacteraceae		0	0	0	1	0	0	0	0	0	0	0	0	1
Cluster13675	Actinobac	Actinol	Actin	Mycoba	Mycobacterium	0	0	0	0	0	0	0	0	0	0	1	0	1
Cluster13676	Actinobac	Actinol	Solirubrobacterales			1	0	0	0	0	0	0	0	0	0	0	0	1
Cluster13680	Armatimonadetes				Armatimonade	1	0	0	0	0	0	0	0	0	0	0	0	1
Cluster13683;size=1						0	0	1	0	0	0	0	0	0	0	0	0	1

Cluster13688	Proteobac	Deltap	Myxococcales			0	0	1	0	0	0	0	0	0	0	0	0	1
Cluster13692	Proteobac	Alphap	Rhodospirillales			1	0	0	0	0	0	0	0	0	0	0	0	1
Cluster13696	Proteobac	Alphap	Rhodospirillales			0	0	0	0	0	0	0	0	0	0	0	1	1
Cluster13700	Proteobac	Deltap	Desu	Geobac	Geobacter	0	0	0	0	0	0	0	0	0	0	1	0	1
Cluster13712	Acidobact	Acidobacteria_Gp3			Gp3	0	0	0	0	0	0	0	0	0	0	1	0	1
Cluster13715	Verrucom	Subdivision3			Subdivision3_g	0	0	1	0	0	0	0	0	0	0	0	0	1
Cluster13719;size=3						0	0	1	0	0	0	0	0	0	0	0	0	1
Cluster13721	Proteobacteria					0	0	1	0	0	0	0	0	0	0	0	0	1
Cluster13725	Acidobact	Acidobacteria_Gp1				0	0	0	1	0	0	0	0	0	0	0	0	1
Cluster13726	Acidobact	Acidobacteria_Gp2			Gp2	0	0	0	1	0	0	0	0	0	0	0	0	1
Cluster13728	Proteobac	Alphaproteobacteria				0	0	0	1	0	0	0	0	0	0	0	0	1
Cluster13739	Verrucom	Subdivision3			Subdivision3_g	0	0	1	0	0	0	0	0	0	0	0	0	1
Cluster13745	Verrucom	Opituta	Opitu	Opituta	Opitutus	0	0	0	0	0	0	0	0	0	0	0	1	1
Cluster13748	Proteobac	Deltap	Myxc	Cystobacteraceae		0	0	1	0	0	0	0	0	0	0	0	0	1
Cluster13753	Acidobact	Acidobacteria_Gp3			Gp3	0	0	0	0	0	0	0	0	1	0	0	0	1
Cluster13760	Proteobac	Alphap	Rhizo	Bradyrh	Bradyrhizobiu	0	0	1	0	0	0	0	0	0	0	0	0	1
Cluster13761	Actinobac	Actino	Actinomycetales			0	0	0	1	0	0	0	0	0	0	0	0	1
Cluster13763	Verrucom	Opituta	Opitu	Opituta	Opitutus	1	0	0	0	0	0	0	0	0	0	0	0	1
Cluster13764	Proteobac	Alphap	Rhodospirillales			1	0	0	0	0	0	0	0	0	0	0	0	1
Cluster13767	Acidobact	Acidobacteria_Gp1			Gp1	0	0	0	1	0	0	0	0	0	0	0	0	1
Cluster13772	Verrucom	Subdivision3			Subdivision3_g	0	0	0	0	0	0	0	0	0	0	0	1	1
Cluster13780;size=1						0	1	0	0	0	0	0	0	0	0	0	0	1
Cluster13782	Proteobac	Deltaproteobacteria				0	0	0	0	0	0	0	0	0	0	0	1	1
Cluster13785	TM7				TM7_genera_i	0	0	0	0	0	0	1	0	0	0	0	0	1
Cluster13788;size=2						0	0	1	0	0	0	0	0	0	0	0	0	1
Cluster13793	Chlamydia	Chlamy	Chlamydiales			0	0	0	0	0	0	1	0	0	0	0	0	1
Cluster13797	Actinobac	Actino	Actin	Mycoba	Mycobacteriu	0	0	0	0	0	0	0	0	0	0	0	1	1
Cluster13798	Acidobact	Acidobacteria_Gp1				0	0	0	0	0	0	1	0	0	0	0	0	1
Cluster13804	Proteobac	Alphaproteobacteria				0	0	0	1	0	0	0	0	0	0	0	0	1

Cluster1381(	Verrucom	Subdivision3			Subdivision3_g	0	0	0	0	0	0	1	0	0	0	0	0	1
Cluster13834	Proteobac	Gammaproteobacteria				0	1	0	0	0	0	0	0	0	0	0	0	1
Cluster13837	Acidobact	Acidobacteria_Gp1			Gp1	0	0	0	0	0	1	0	0	0	0	0	0	1
Cluster13844	Acidobact	Acidobacteria_Gp2			Gp2	0	0	0	0	0	0	0	0	0	0	0	1	1
Cluster1385(	Acidobact	Acidobacteria_Gp1			Gp1	0	0	1	0	0	0	0	0	0	0	0	0	1
Cluster1386(	Proteobac	Gamm	Xantl	Sinobac	Steroidobacter	0	0	1	0	0	0	0	0	0	0	0	0	1
Cluster13861	Verrucom	Subdivision3			Subdivision3_g	0	0	0	0	1	0	0	0	0	0	0	0	1
Cluster13873;size=13						0	0	0	1	0	0	0	0	0	0	0	0	1
Cluster1387!	Proteobacteria					0	0	0	0	0	0	0	1	0	0	0	0	1
Cluster1388(	Acidobacteria					0	0	0	0	0	0	0	0	0	0	0	1	1
Cluster13884	Proteobac	Deltap	Myxococcales			0	0	0	0	0	0	0	1	0	0	0	0	1
Cluster13887	Acidobact	Acidobacteria_Gp1				0	0	0	0	0	0	0	1	0	0	0	0	1
Cluster1389!	Proteobac	Gammaproteobacteria				0	0	0	1	0	0	0	0	0	0	0	0	1
Cluster13897	Proteobac	Deltap	Myxococcales			0	0	0	0	0	0	0	1	0	0	0	0	1
Cluster1389{	Bacteroid	Sphing	Sphir	Chitinophagaceae		0	1	0	0	0	0	0	0	0	0	0	0	1
Cluster13902	Acidobact	Acidobacteria_Gp3				0	1	0	0	0	0	0	0	0	0	0	0	1
Cluster13904	Acidobact	Acidobacteria_Gp3			Gp3	0	0	1	0	0	0	0	0	0	0	0	0	1
Cluster13911	Proteobac	Alphap	Rhizc	Bradyrh	Agromonas	0	0	1	0	0	0	0	0	0	0	0	0	1
Cluster1391(	Proteobacteria					0	0	0	0	0	0	0	0	1	0	0	0	1
Cluster1392!	Acidobact	Acidobacteria_Gp10			Gp10	0	0	1	0	0	0	0	0	0	0	0	0	1
Cluster13932	Acidobact	Acidobacteria_Gp5			Gp5	0	0	1	0	0	0	0	0	0	0	0	0	1
Cluster1393(	Acidobact	Acidobacteria_Gp3			Bryobacter	0	0	0	0	0	0	1	0	0	0	0	0	1
Cluster13941	Proteobac	Alphap	Rhizc	Xanthol	Pseudolabrys	0	0	0	0	0	0	0	0	1	0	0	0	1
Cluster1394{	Proteobac	Alphap	Rhodospirillales			0	0	0	0	0	0	0	1	0	0	0	0	1
Cluster13957	Proteobac	Alphaproteobacteria				0	0	0	0	0	0	0	0	1	0	0	0	1
Cluster13967	Proteobac	Gamm	Xantl	Sinobac	Steroidobacter	0	0	1	0	0	0	0	0	0	0	0	0	1
Cluster1398(	Acidobact	Acidobacteria_Gp1			Gp1	0	0	0	0	0	0	0	1	0	0	0	0	1
Cluster13997	Acidobact	Acidobacteria_Gp2			Gp2	0	0	1	0	0	0	0	0	0	0	0	0	1
Cluster1399{	Proteobac	Gamm	Xantl	Sinobacteraceae		0	0	0	0	0	0	0	0	0	0	0	1	1

Cluster14007	Acidobact	Acidobacteria_Gp1				0	0	0	0	0	0	0	0	1	0	0	0	1
Cluster14011;size=5						0	0	1	0	0	0	0	0	0	0	0	0	1
Cluster14013	Chloroflex	Ktedor	Ktedo	Ktedono	Ktedonobacter	0	0	0	0	0	0	0	0	0	0	0	1	1
Cluster14016	Proteobac	Alphap	Caulo	Caulobacteraceae		0	0	0	0	0	1	0	0	0	0	0	0	1
Cluster14017	Gemmatir	Gemm	Gem	Gemma	Gemmatimona	0	0	1	0	0	0	0	0	0	0	0	0	1
Cluster14019	Acidobact	Acidobacteria_Gp3			Gp3	0	0	1	0	0	0	0	0	0	0	0	0	1
Cluster14022	Proteobac	Betapr	Burkl	Burkholderiaceae		0	0	1	0	0	0	0	0	0	0	0	0	1
Cluster14023	Acidobact	Acidobacteria_Gp1				0	0	0	0	0	0	0	1	0	0	0	0	1
Cluster14031	TM7				TM7_genera_i	0	0	1	0	0	0	0	0	0	0	0	0	1
Cluster14038	Acidobact	Acidobacteria_Gp3			Gp3	0	0	0	0	0	0	0	0	0	0	0	1	1
Cluster14039	Bacteroid	Sphing	Sphir	Chitinophagaceae		0	0	1	0	0	0	0	0	0	0	0	0	1
Cluster14042	Chlamydia	Chlamy	Chlar	Parachla	Parachlamydia	0	0	0	0	0	0	0	0	0	0	0	1	1
Cluster14043	Proteobac	Gammaproteobacteria				0	0	0	0	0	0	0	1	0	0	0	0	1
Cluster14046	Proteobac	Gamm	Xantl	Sinobac	Steroidobacter	0	0	0	0	0	0	1	0	0	0	0	0	1
Cluster14048	Nitrospira	Nitrosp	Nitro	Nitrospi	Nitrospira	0	0	0	0	0	0	0	0	0	0	0	1	1
Cluster14050	Acidobact	Acidobacteria_Gp1				1	0	0	0	0	0	0	0	0	0	0	0	1
Cluster14051	Acidobact	Acidobacteria_Gp1			Gp1	0	0	0	0	0	1	0	0	0	0	0	0	1
Cluster14055;size=3						0	1	0	0	0	0	0	0	0	0	0	0	1
Cluster14057	Proteobac	Betaproteobacteria				0	0	1	0	0	0	0	0	0	0	0	0	1
Cluster14063	Proteobac	Alphaproteobacteria				0	0	0	1	0	0	0	0	0	0	0	0	1
Cluster14064	Proteobac	Deltap	Myxococcales			0	0	0	0	0	0	1	0	0	0	0	0	1
Cluster14074	Acidobact	Acidobacteria_Gp3			Gp3	0	0	0	0	0	0	1	0	0	0	0	0	1
Cluster14075	Proteobac	Betapr	Burkholderiales			0	0	1	0	0	0	0	0	0	0	0	0	1
Cluster14076	Acidobact	Acidobacteria_Gp1			Gp1	0	0	0	0	0	0	1	0	0	0	0	0	1
Cluster14079	Acidobact	Acidobacteria_Gp3			Gp3	0	0	0	0	0	0	0	0	0	0	0	1	1
Cluster14080	Actinobac	Actinol	Actinomycetales			0	0	0	0	0	0	0	0	1	0	0	0	1
Cluster14086;size=3						0	0	1	0	0	0	0	0	0	0	0	0	1
Cluster14088	Proteobac	Alphap	Rhizobiales			0	0	0	0	0	0	0	0	0	0	0	1	1
Cluster14089	Acidobact	Acidobacteria_Gp1				0	0	0	0	0	1	0	0	0	0	0	0	1

Cluster1409	Bacteroid	Sphing Sphir Chitinophagaceae			0	0	0	0	0	0	0	1	0	0	0	0	1
Cluster14097;size=1					0	0	0	0	0	0	0	0	1	0	0	0	1
Cluster1410	Proteobac	Deltaproteobacteria			0	0	0	0	0	0	0	1	0	0	0	0	1
Cluster1411	Acidobact	Acidobacteria_Gp3	Gp3		0	0	0	0	0	0	0	0	0	0	0	1	1
Cluster1412	Planctom	Plancto Planc Planctomycetaceae			0	0	0	0	0	0	1	0	0	0	0	0	1
Cluster1412	Proteobac	Alphap Rhodospirillales			0	0	0	0	0	0	1	0	0	0	0	0	1
Cluster14125;size=11					0	1	0	0	0	0	0	0	0	0	0	0	1
Cluster14131	Acidobact	Acidobacteria_Gp1			0	0	0	1	0	0	0	0	0	0	0	0	1
Cluster1413	Acidobact	Acidobacteria_Gp1			0	0	0	0	0	0	0	1	0	0	0	0	1
Cluster1413	Acidobact	Acidobacteria_Gp3			0	0	0	0	0	0	1	0	0	0	0	0	1
Cluster1414	Acidobact	Acidobacteria_Gp1			0	0	1	0	0	0	0	0	0	0	0	0	1
Cluster14141	Proteobac	Deltaproteobacteria			0	0	0	0	0	0	0	1	0	0	0	0	1
Cluster14142;size=1					0	0	0	0	0	0	0	0	0	0	0	1	1
Cluster1414	Acidobact	Acidobacteria_Gp1	Gp1		1	0	0	0	0	0	0	0	0	0	0	0	1
Cluster1415	Verrucom	Subdivision3	Subdivision3_g		0	0	1	0	0	0	0	0	0	0	0	0	1
Cluster1415	Acidobact	Acidobacteria_Gp2	Gp2		0	0	0	0	0	0	0	1	0	0	0	0	1
Cluster1416	Acidobact	Acidobacteria_Gp3			0	0	0	1	0	0	0	0	0	0	0	0	1
Cluster1417	Acidobact	Acidobacteria_Gp2	Gp2		0	0	0	0	0	0	1	0	0	0	0	0	1
Cluster14181	Acidobact	Acidobacteria_Gp1			1	0	0	0	0	0	0	0	0	0	0	0	1
Cluster1418	Proteobac	Alphap Rhizc Xanthol	Pseudolabrys		0	0	1	0	0	0	0	0	0	0	0	0	1
Cluster1418	Acidobact	Acidobacteria_Gp1	Gp1		0	0	0	0	0	0	0	0	1	0	0	0	1
Cluster14190;size=4					0	0	1	0	0	0	0	0	0	0	0	0	1
Cluster1420	Proteobac	Gammaproteobacteria			0	0	1	0	0	0	0	0	0	0	0	0	1
Cluster1422	Acidobact	Acidobacteria_Gp1	Gp1		0	0	0	0	0	0	0	0	0	1	0	0	1
Cluster1423	Acidobact	Acidobacteria_Gp2	Gp2		0	0	0	0	0	0	0	0	0	0	0	1	1
Cluster1423	Acidobact	Acidobacteria_Gp1	Gp1		0	0	0	0	0	0	0	1	0	0	0	0	1
Cluster14241;size=8					0	0	0	0	0	0	0	0	0	0	0	1	1
Cluster1424	Acidobact	Acidobacteria_Gp3			0	0	0	0	0	0	0	0	0	0	0	1	1
Cluster1425	Acidobact	Acidobacteria_Gp1			0	0	0	0	0	0	0	1	0	0	0	0	1

Cluster14258	Actinobac	Actinol	Acidi	Acidimi	Aciditerrimona	0	0	0	0	0	0	0	0	0	0	0	1	1
Cluster14262	Proteobac	Gamm	Xantl	Xanthor	Frateuria	0	0	0	0	0	0	1	0	0	0	0	0	1
Cluster14265	Proteobac	Gamm	Legio	Coxiella	Aquicella	0	0	1	0	0	0	0	0	0	0	0	0	1
Cluster14286	Gemmatir	Gemm	Gemı	Gemma	Gemmatimona	0	0	0	0	0	0	0	0	1	0	0	0	1
Cluster14312	Actinobac	Actinol	Solirubrobacterales			0	1	0	0	0	0	0	0	0	0	0	0	1
Cluster14320	Acidobact	Acidobacteria_Gp2			Gp2	0	0	0	0	0	0	0	0	0	0	1	0	1
Cluster14322;size=1						0	0	1	0	0	0	0	0	0	0	0	0	1
Cluster14339	Acidobact	Acidobacteria_Gp1			Gp1	0	0	0	0	0	0	0	0	0	0	0	1	1
Cluster14343;size=3						0	0	0	0	0	0	1	0	0	0	0	0	1
Cluster14346	Proteobac	Alphaproteobacteria				0	0	0	0	0	0	0	0	0	0	0	1	1
Cluster14348	Verrucom	Spartobacteria			Spartobacteria	0	0	0	0	0	0	0	0	1	0	0	0	1
Cluster14349	Verrucom	Subdivision3			Subdivision3_g	0	0	0	0	0	0	1	0	0	0	0	0	1
Cluster14352	Bacteroid	Sphing	Sphir	Chitinophagaceae		0	0	0	1	0	0	0	0	0	0	0	0	1
Cluster14362	Actinobac	Actinol	Actinomycetales			0	0	0	0	0	0	0	0	0	0	0	1	1
Cluster14364	Acidobacteria					0	1	0	0	0	0	0	0	0	0	0	0	1
Cluster14373	Acidobact	Acidobacteria_Gp3			Bryobacter	0	0	0	0	0	1	0	0	0	0	0	0	1
Cluster14378	Acidobact	Acidobacteria_Gp1			Gp1	0	0	0	0	0	0	1	0	0	0	0	0	1
Cluster14382	Proteobac	Alphap	Rhodospirillales			0	0	0	0	0	0	0	0	0	0	1	0	1
Cluster14383	Proteobac	Alphap	Rhod	Acetobacteraceae		0	0	0	0	0	0	0	0	1	0	0	0	1
Cluster14386	Proteobac	Deltap	Myxc	Polyangiaceae		0	0	0	0	0	0	0	0	0	0	0	1	1
Cluster14389;size=3						0	0	0	0	0	0	0	0	0	0	1	0	1
Cluster14394	Proteobac	Alphap	Rhizobiales			0	0	0	0	0	1	0	0	0	0	0	0	1
Cluster14395	Acidobact	Acidobacteria_Gp1				0	0	1	0	0	0	0	0	0	0	0	0	1
Cluster14398	Acidobact	Acidobacteria_Gp3			Gp3	0	0	1	0	0	0	0	0	0	0	0	0	1
Cluster14399;size=2						0	0	1	0	0	0	0	0	0	0	0	0	1
Cluster14405	Acidobact	Acidobacteria_Gp3				0	0	1	0	0	0	0	0	0	0	0	0	1
Cluster14415	Chloroflex	Anaerc	Anae	Anaerolineaceae		0	1	0	0	0	0	0	0	0	0	0	0	1
Cluster14418	Acidobact	Acidobacteria_Gp1				0	0	0	0	0	1	0	0	0	0	0	0	1
Cluster14420	Proteobac	Deltaproteobacteria				0	0	0	0	0	0	0	0	0	0	0	1	1

Cluster14421	Acidobact	Acidobacteria_Gp10			Gp10	0	0	0	0	0	0	0	0	0	0	0	1	1
Cluster14425	Actinobac	Actinol	Soliru	Conexib	Conexibacter	0	0	0	0	0	1	0	0	0	0	0	0	1
Cluster14426;size=5						1	0	0	0	0	0	0	0	0	0	0	0	1
Cluster14427;size=1						0	0	1	0	0	0	0	0	0	0	0	0	1
Cluster14428	Acidobact	Acidobacteria_Gp1			Gp1	0	0	0	0	0	0	0	1	0	0	0	0	1
Cluster14448;size=1						0	0	0	0	0	0	0	0	1	0	0	0	1
Cluster14457;size=1						0	0	0	0	0	0	0	0	0	0	0	1	1
Cluster14461	Verrucom	Opituta	Opitu	Opituta	Opitutus	1	0	0	0	0	0	0	0	0	0	0	0	1
Cluster14462	Chlamydia	Chlamy	Chlamydiales			0	0	0	0	0	0	1	0	0	0	0	0	1
Cluster14465	Acidobact	Acidobacteria_Gp1				0	0	0	0	0	0	0	0	0	0	0	1	1
Cluster14467;size=5						0	0	0	0	1	0	0	0	0	0	0	0	1
Cluster14468	Acidobact	Acidobacteria_Gp1			Granulicella	0	0	0	0	0	0	0	0	1	0	0	0	1
Cluster14469	Proteobac	Gammaproteobacteria				0	0	0	0	0	1	0	0	0	0	0	0	1
Cluster14482	Acidobact	Acidobacteria_Gp3			Bryobacter	0	0	1	0	0	0	0	0	0	0	0	0	1
Cluster14486	Acidobact	Acidobacteria_Gp1				0	0	0	0	0	0	0	0	1	0	0	0	1
Cluster14487;size=1						0	0	0	0	0	0	0	0	1	0	0	0	1
Cluster14489	Acidobact	Acidobacteria_Gp1			Granulicella	0	0	0	0	0	0	1	0	0	0	0	0	1
Cluster14495;size=10						0	0	0	0	0	0	0	1	0	0	0	0	1
Cluster14500	Proteobac	Gammaproteobacteria				0	0	0	0	0	0	1	0	0	0	0	0	1
Cluster14504	Acidobacteria					0	0	1	0	0	0	0	0	0	0	0	0	1
Cluster14517	Proteobac	Alphap	Rhod	Acetoba	Acidisphaera	0	0	0	0	0	0	0	0	0	0	0	1	1
Cluster14520	Acidobact	Acidobacteria_Gp2			Gp2	1	0	0	0	0	0	0	0	0	0	0	0	1
Cluster14531	Proteobac	Alphaproteobacteria				0	0	0	0	0	0	1	0	0	0	0	0	1
Cluster14533	Acidobact	Acidobacteria_Gp1				0	0	1	0	0	0	0	0	0	0	0	0	1
Cluster14548;size=1						0	0	0	0	0	0	1	0	0	0	0	0	1
Cluster14552;size=2						0	0	0	0	0	0	0	0	1	0	0	0	1
Cluster14563	Acidobact	Acidobacteria_Gp1				0	0	0	0	0	0	1	0	0	0	0	0	1
Cluster14581	Acidobact	Acidobacteria_Gp3			Gp3	0	0	0	0	1	0	0	0	0	0	0	0	1
Cluster14582	Armatimo	Armati	Arma	Armatin	Armatimonas/	0	0	1	0	0	0	0	0	0	0	0	0	1

Cluster14583;size=2						0	0	0	0	0	0	1	0	0	0	0	0	1
Cluster14600	Proteobac	Betapr	Burkl	Oxalobacteraceae		1	0	0	0	0	0	0	0	0	0	0	0	1
Cluster14605	Acidobacteria					0	0	0	0	0	0	0	0	0	0	0	1	1
Cluster14613	Acidobact	Acidobacteria_Gp1				0	0	0	0	0	0	1	0	0	0	0	0	1
Cluster14614	Proteobac	Alphaproteobacteria				0	0	0	0	0	0	0	0	1	0	0	0	1
Cluster14615	Bacteroid	Sphing	Sphir	Chitinop	Sediminibacter	0	0	0	0	0	0	1	0	0	0	0	0	1
Cluster14620	Proteobac	Alphap	Alphaproteob		Rhizomicrobiu	0	0	0	0	1	0	0	0	0	0	0	0	1
Cluster14623	Acidobact	Acidobacteria_Gp2			Gp2	0	0	1	0	0	0	0	0	0	0	0	0	1
Cluster14631	Acidobact	Acidobacteria_Gp1				0	0	0	0	0	0	1	0	0	0	0	0	1
Cluster14632	Acidobact	Acidobacteria_Gp1				0	0	1	0	0	0	0	0	0	0	0	0	1
Cluster14651	Proteobac	Deltaproteobacteria				0	0	0	0	0	0	1	0	0	0	0	0	1
Cluster14660	Acidobact	Acidobacteria_Gp2			Gp2	0	0	0	0	0	0	1	0	0	0	0	0	1
Cluster14662	Chloroflex	Ktedon	Kted	Ktedonc	Ktedonobacter	0	0	0	0	1	0	0	0	0	0	0	0	1
Cluster14670	Acidobact	Acidobacteria_Gp1			Acidobacteriu	0	0	0	0	0	0	0	0	0	0	0	1	1
Cluster14672	Proteobac	Alphap	Rhod	Acetobacteraceae		0	0	0	0	0	0	1	0	0	0	0	0	1
Cluster14676	Verrucom	Spartobacteria			Spartobacteria	0	0	0	0	0	0	1	0	0	0	0	0	1
Cluster14677	Actinobac	Actinol	Soliru	Conexib	Conexibacter	0	0	0	0	0	0	0	0	0	1	0	0	1
Cluster14687	Proteobac	Alphap	Rhizo	Beijerinckiaceae		0	0	0	0	0	0	1	0	0	0	0	0	1
Cluster14689;size=3						0	0	0	0	1	0	0	0	0	0	0	0	1
Cluster14692	Acidobact	Acidobacteria_Gp1				1	0	0	0	0	0	0	0	0	0	0	0	1
Cluster14693	Acidobact	Acidobacteria_Gp1				0	0	0	0	1	0	0	0	0	0	0	0	1
Cluster14694	Proteobacteria					0	0	0	0	0	0	0	0	0	0	0	1	1
Cluster14707;size=5						0	0	1	0	0	0	0	0	0	0	0	0	1
Cluster14714;size=3						0	0	0	0	0	0	0	1	0	0	0	0	1
Cluster14719	Acidobact	Acidobacteria_Gp1				0	0	0	0	0	0	0	0	0	0	0	1	1
Cluster14721	Acidobact	Acidobacteria_Gp1			Gp1	0	0	1	0	0	0	0	0	0	0	0	0	1
Cluster14725	Proteobac	Alphap	Rhod	Acetoba	Acidocella	0	0	0	1	0	0	0	0	0	0	0	0	1
Cluster14727	Proteobac	Alphap	Rhizobiales			0	0	1	0	0	0	0	0	0	0	0	0	1
Cluster14737	Proteobac	Alphap	Rhizo	Xanthol	Labrys	0	0	0	0	0	0	0	0	1	0	0	0	1

Cluster14750	Proteobac	Deltaproteobacteria				0	0	0	0	0	0	1	0	0	0	0	0	1
Cluster14751	Proteobac	Gamm	Legio	Coxiella	Aquicella	0	0	0	0	0	0	1	0	0	0	0	0	1
Cluster14753	Proteobac	Gammaproteobacteria				0	0	0	0	0	0	1	0	0	0	0	0	1
Cluster14765	Proteobac	Alphap	Rhodospirillales			0	0	0	0	0	0	0	0	1	0	0	0	1
Cluster14771	Acidobact	Acidobacteria_Gp3			Gp3	0	0	0	0	0	0	0	0	0	0	0	1	1
Cluster14775	Acidobact	Acidobacteria_Gp1				0	0	0	0	0	0	0	0	0	0	0	1	1
Cluster14784;size=1						0	0	0	0	0	0	1	0	0	0	0	0	1
Cluster14785	Proteobac	Alphap	Rhizc	Bradyrh	Bradyrhizobiur	0	0	0	1	0	0	0	0	0	0	0	0	1
Cluster14788	Acidobacteria					0	0	0	0	0	0	0	0	0	0	0	1	1
Cluster14795	Proteobac	Betapr	Burkl	Burkholderiaceae		0	0	0	0	0	0	0	0	0	0	1	0	1
Cluster14810	Proteobac	Alphap	Rhizobiales			0	0	0	0	0	0	0	0	0	0	0	1	1
Cluster14812	Acidobact	Acidobacteria_Gp1			Gp1	0	0	0	1	0	0	0	0	0	0	0	0	1
Cluster14813	Acidobact	Acidobacteria_Gp1			Gp1	0	1	0	0	0	0	0	0	0	0	0	0	1
Cluster14819	Proteobac	Alphap	Rhizc	Bradyrhizobiaceae		0	0	0	0	0	0	0	0	1	0	0	0	1
Cluster14831	Proteobac	Gamm	Enter	Enterob	Rahnella	0	0	0	0	0	0	0	0	0	0	0	1	1
Cluster14836	Acidobact	Acidobacteria_Gp1			Gp1	0	0	0	0	0	0	0	0	0	0	0	1	1
Cluster14839	Acidobact	Acidobacteria_Gp1				0	0	0	0	0	0	0	0	1	0	0	0	1
Cluster14843;size=1						0	0	1	0	0	0	0	0	0	0	0	0	1
Cluster14853	Acidobact	Acidobacteria_Gp1			Gp1	0	0	1	0	0	0	0	0	0	0	0	0	1
Cluster14869	Proteobac	Deltap	Myxococcales			0	0	1	0	0	0	0	0	0	0	0	0	1
Cluster14878	Bacteroid	Sphing	Sphir	Chitinophagaceae		0	0	0	0	0	0	0	1	0	0	0	0	1
Cluster14888;size=3						0	0	0	0	0	0	1	0	0	0	0	0	1
Cluster14893	Acidobact	Acidobacteria_Gp1				0	0	0	0	0	0	0	0	0	0	0	1	1
Cluster14896	Proteobacteria					0	0	1	0	0	0	0	0	0	0	0	0	1
Cluster14899	Proteobacteria					0	0	0	0	1	0	0	0	0	0	0	0	1
Cluster14901	Actinobac	Actinol	Acidi	Acidimi	Aciditerrimona	0	0	0	0	0	0	0	0	1	0	0	0	1
Cluster14903	Acidobact	Acidobacteria_Gp3				0	0	1	0	0	0	0	0	0	0	0	0	1
Cluster14905;size=1						0	0	0	0	0	0	0	0	0	0	0	1	1
Cluster14917	Actinobac	Actinol	Actinomycetales			1	0	0	0	0	0	0	0	0	0	0	0	1

Cluster14926;size=2						0	0	0	0	0	0	1	0	0	0	0	0	1
Cluster14933	Proteobac	Deltap	Bdell	Bdellovi	Bdellovibrio	0	0	0	0	0	0	0	1	0	0	0	0	1
Cluster14935	Acidobact	Acidobacteria_Gp7			Gp7	0	0	0	0	0	0	0	0	0	0	0	1	1
Cluster14939	Acidobact	Acidobacteria_Gp1				0	1	0	0	0	0	0	0	0	0	0	0	1
Cluster14943	Acidobact	Acidobacteria_Gp2			Gp2	0	0	0	0	0	0	1	0	0	0	0	0	1
Cluster14946;size=4						0	0	0	0	0	0	0	1	0	0	0	0	1
Cluster14948	Proteobac	Deltaproteobacteria				0	0	0	0	0	0	0	1	0	0	0	0	1
Cluster14953	Actinobac	Actinobacteria				0	0	0	0	0	0	0	0	1	0	0	0	1
Cluster14971	Proteobac	Betapr	Burkl	Oxalobacteraceae		0	0	0	0	0	0	1	0	0	0	0	0	1
Cluster14978;size=3						0	0	0	0	0	0	0	1	0	0	0	0	1
Cluster14989	Acidobact	Acidobacteria_Gp1			Gp1	0	0	0	0	0	0	0	0	1	0	0	0	1
Cluster15000	Acidobact	Acidobacteria_Gp3			Gp3	0	0	1	0	0	0	0	0	0	0	0	0	1
Cluster15001;size=1						0	0	0	0	0	0	1	0	0	0	0	0	1
Cluster15008	Acidobacteria					0	0	0	0	1	0	0	0	0	0	0	0	1
Cluster15019;size=1						0	0	0	0	0	0	0	1	0	0	0	0	1
Cluster15023	Acidobact	Acidobacteria_Gp3			Gp3	0	0	0	0	0	0	0	1	0	0	0	0	1
Cluster15039	Gemmatir	Gemm	Gemı	Gemma	Gemmatimona	0	0	0	0	1	0	0	0	0	0	0	0	1
Cluster15040	Proteobac	Alphap	Rhod	Rhodospirillaceae		0	0	0	0	0	0	0	1	0	0	0	0	1
Cluster15041	Actinobac	Actinol	Acidi	Acidimi	Aciditerrimona	0	0	0	0	0	0	0	1	0	0	0	0	1
Cluster15047	Acidobact	Acidobacteria_Gp1				0	0	0	0	1	0	0	0	0	0	0	0	1
Cluster15051	Acidobact	Acidobacteria_Gp3			Gp3	0	0	0	0	0	0	0	0	1	0	0	0	1
Cluster15052	Planctomy	Plancto	Planc	Plancto	Zavarzinella	0	0	0	0	0	0	0	1	0	0	0	0	1
Cluster15053	Proteobac	Betaproteobacteria				0	0	0	0	1	0	0	0	0	0	0	0	1
Cluster15062;size=1						0	0	0	0	0	0	0	0	0	0	0	1	1
Cluster15063	Proteobac	Gamm	Xantl	Sinobac	Steroidobacter	0	0	0	0	0	0	0	0	0	0	1	0	1
Cluster15068;size=2						0	0	1	0	0	0	0	0	0	0	0	0	1
Cluster15070	Acidobact	Acidobacteria_Gp2			Gp2	0	0	0	0	0	0	0	0	0	0	1	0	1
Cluster15076	Acidobact	Acidobacteria_Gp2			Gp2	0	1	0	0	0	0	0	0	0	0	0	0	1
Cluster15077;size=1						0	0	0	0	0	0	1	0	0	0	0	0	1

Cluster15078	Proteobac	Gamm	Legio	Coxiella	Coxiella	0	0	0	0	0	0	1	0	0	0	0	0	1
Cluster15080	Acidobact	Acidobacteria_Gp2			Gp2	0	0	0	0	0	0	1	0	0	0	0	0	1
Cluster15083	Acidobact	Acidobacteria_Gp10			Gp10	0	0	0	1	0	0	0	0	0	0	0	0	1
Cluster15096	Proteobac	Alphaproteobacteria				0	0	0	0	1	0	0	0	0	0	0	0	1
Cluster15097	Nitrospira	Nitrosp	Nitro	Nitrospi	Nitrospira	0	1	0	0	0	0	0	0	0	0	0	0	1
Cluster15108	Acidobact	Acidobacteria_Gp2			Gp2	0	1	0	0	0	0	0	0	0	0	0	0	1
Cluster15140	Acidobact	Acidobacteria_Gp3			Gp3	0	0	0	0	0	0	1	0	0	0	0	0	1
Cluster15142;size=1						0	0	0	0	0	0	0	0	0	0	0	1	1
Cluster15143	Acidobact	Acidobacteria_Gp2			Gp2	0	0	0	1	0	0	0	0	0	0	0	0	1
Cluster15149;size=7						0	0	0	1	0	0	0	0	0	0	0	0	1
Cluster15161	Actinobac	Actinol	Acidimicrobiales			0	1	0	0	0	0	0	0	0	0	0	0	1
Cluster15170	Acidobact	Acidobacteria_Gp1				0	0	1	0	0	0	0	0	0	0	0	0	1
Cluster15173	Proteobac	Deltaproteobacteria				0	0	0	0	0	0	1	0	0	0	0	0	1
Cluster15182	Proteobac	Alphap	Rhizo	Bradyrhizobiaceae		0	0	0	0	0	0	1	0	0	0	0	0	1
Cluster15185	Verrucom	Opituta	Opitu	Opituta	Opitutus	0	0	1	0	0	0	0	0	0	0	0	0	1
Cluster15187	Acidobact	Acidobacteria_Gp2			Gp2	0	0	0	0	0	0	0	1	0	0	0	0	1
Cluster15188	Proteobac	Gamm	Xantl	Sinobac	Steroidobacter	0	0	0	0	0	0	1	0	0	0	0	0	1
Cluster15193	Actinobac	Actinol	Actinomycetales			0	0	0	0	0	0	1	0	0	0	0	0	1
Cluster15195	Proteobac	Alphap	Rhod	Acetoba	Acidisphaera	0	0	1	0	0	0	0	0	0	0	0	0	1
Cluster15199;size=2						1	0	0	0	0	0	0	0	0	0	0	0	1
Cluster15201	Verrucom	Opituta	Opitu	Opituta	Opitutus	0	1	0	0	0	0	0	0	0	0	0	0	1
Cluster15204;size=2						0	0	0	0	0	0	1	0	0	0	0	0	1
Cluster15207	Bacteroid	Sphing	Sphir	Chitinophagaceae		0	0	0	0	0	0	0	0	1	0	0	0	1
Cluster15215	Proteobac	Gamm	Xantl	Sinobac	Steroidobacter	0	0	0	0	0	0	0	0	0	1	0	0	1
Cluster15216	Proteobac	Alphap	Sphir	Sphingomonadaceae		0	0	0	0	0	0	0	1	0	0	0	0	1
Cluster15219	Proteobac	Gamm	Xantl	Sinobac	Nevskia	0	0	0	0	0	0	0	0	0	0	1	0	1
Cluster15230	Proteobac	Deltap	Bdell	Bdellovi	Bdellovibrio	0	1	0	0	0	0	0	0	0	0	0	0	1
Cluster15237	Chloroflex	Ktedor	Ktedonobacterales			0	0	0	0	0	0	1	0	0	0	0	0	1
Cluster15239	Armatimo	Chthor	Chth	Chthon	Chthonomona	0	0	0	0	0	0	0	1	0	0	0	0	1

Cluster15240	Acidobact	Acidobacteria_Gp2			Gp2	0	0	1	0	0	0	0	0	0	0	0	0	1
Cluster15246;size=1						1	0	0	0	0	0	0	0	0	0	0	0	1
Cluster15248	Bacteroid	Sphing	Sphir	Chitinop	Flavihumibacte	1	0	0	0	0	0	0	0	0	0	0	0	1
Cluster15250	Acidobact	Acidobacteria_Gp1				1	0	0	0	0	0	0	0	0	0	0	0	1
Cluster15252	Proteobac	Alphap	Rhizobiales			1	0	0	0	0	0	0	0	0	0	0	0	1
Cluster15256;size=2						1	0	0	0	0	0	0	0	0	0	0	0	1
Cluster15261	Acidobact	Acidobacteria_Gp10			Gp10	1	0	0	0	0	0	0	0	0	0	0	0	1
Cluster15263	Acidobact	Acidobacteria_Gp1				1	0	0	0	0	0	0	0	0	0	0	0	1
Cluster15265	Proteobac	Alphap	Rhod	Acetobacteraceae		1	0	0	0	0	0	0	0	0	0	0	0	1
Cluster15267	Proteobac	Alphap	Rhizobiales			1	0	0	0	0	0	0	0	0	0	0	0	1
Cluster15268	Proteobac	Alphap	Rhodospirillales			0	0	0	0	0	0	0	0	0	0	0	1	1
Cluster15270	Acidobact	Acidobacteria_Gp2			Gp2	1	0	0	0	0	0	0	0	0	0	0	0	1
Cluster15275	Proteobac	Alphaproteobacteria				0	0	0	0	0	0	0	0	0	1	0	0	1
Cluster15278	Proteobacteria					0	0	0	0	0	0	1	0	0	0	0	0	1
Cluster15280	Acidobact	Acidobacteria_Gp1			Gp1	0	0	0	0	0	0	1	0	0	0	0	0	1
Cluster15283	Acidobact	Acidobacteria_Gp2			Gp2	1	0	0	0	0	0	0	0	0	0	0	0	1
Cluster15285;size=4						0	0	0	0	0	0	0	0	0	0	1	0	1
Cluster15286	Armatimonadetes				Armatimonade	1	0	0	0	0	0	0	0	0	0	0	0	1
Cluster15290	Proteobac	Gammaproteobacteria				1	0	0	0	0	0	0	0	0	0	0	0	1
Cluster15296;size=1						1	0	0	0	0	0	0	0	0	0	0	0	1
Cluster15298	Verrucom	Opitut	Opitu	Opituta	Opitutus	0	0	0	0	0	0	0	0	0	0	0	1	1
Cluster15300	Proteobac	Alphap	Rhodospirillales			0	0	0	0	0	0	0	0	0	0	0	1	1
Cluster15301	Proteobac	Betaproteobacteria				0	0	0	0	0	0	1	0	0	0	0	0	1
Cluster15302	Bacteroid	Sphing	Sphir	Chitinophagaceae		0	0	0	0	0	0	0	1	0	0	0	0	1
Cluster15306	Proteobac	Gammaproteobacteria				1	0	0	0	0	0	0	0	0	0	0	0	1
Cluster15309	Acidobact	Acidobacteria_Gp1				1	0	0	0	0	0	0	0	0	0	0	0	1
Cluster15314	Acidobacteria					0	0	0	0	0	0	1	0	0	0	0	0	1
Cluster15320	Proteobac	Betapr	Burkl	Burkhol	Burkholderia	1	0	0	0	0	0	0	0	0	0	0	0	1
Cluster15323	Proteobac	Alphaproteobacteria				0	0	0	0	0	0	0	1	0	0	0	0	1

Cluster15325	Acidobact	Acidobacteria_Gp1				0	0	0	0	0	0	0	0	0	0	0	1	1
Cluster15330	Acidobact	Acidobacteria_Gp10			Gp10	0	0	0	0	0	0	0	0	0	0	1	0	1
Cluster15331	Acidobact	Acidobacteria_Gp1			Gp1	0	0	1	0	0	0	0	0	0	0	0	0	1
Cluster15335	Acidobact	Acidobacteria_Gp3			Gp3	0	0	0	0	0	0	0	0	0	0	1	0	1
Cluster15337	Verrucom	Opituta	Opitu	Opituta	Opitutus	1	0	0	0	0	0	0	0	0	0	0	0	1
Cluster15339	Chlamydia	Chlamy	Chlar	Parachla	Neochlamydia	0	0	0	0	0	0	0	1	0	0	0	0	1
Cluster15340	Acidobact	Acidobacteria_Gp1				0	0	0	0	0	0	0	0	0	0	0	1	1
Cluster15345	Proteobac	Alphap	Rhizobiales			1	0	0	0	0	0	0	0	0	0	0	0	1
Cluster15348	Armatimo	Armati	Arma	Armatin	Armatimonas/	1	0	0	0	0	0	0	0	0	0	0	0	1
Cluster15350;size=1						1	0	0	0	0	0	0	0	0	0	0	0	1
Cluster15353	Chloroflex	Ktedonobacteria				0	0	0	0	0	0	0	0	0	0	0	1	1
Cluster15354	Elusimicro	Elusim	Elusi	Elusimic	Elusimicrobiun	1	0	0	0	0	0	0	0	0	0	0	0	1
Cluster15358;size=1						1	0	0	0	0	0	0	0	0	0	0	0	1
Cluster15366	Acidobact	Acidobacteria_Gp1				1	0	0	0	0	0	0	0	0	0	0	0	1
Cluster15368	Proteobac	Betapr	Burkl	Burkhol	Burkholderia	0	0	0	0	0	0	0	1	0	0	0	0	1
Cluster15370	Verrucom	Opituta	Opitu	Opituta	Opitutus	1	0	0	0	0	0	0	0	0	0	0	0	1
Cluster15377	Proteobac	Alphap	Rhizo	Xanthol	Pseudolabrys	1	0	0	0	0	0	0	0	0	0	0	0	1
Cluster15378;size=1						1	0	0	0	0	0	0	0	0	0	0	0	1
Cluster15383	Verrucom	Opituta	Opitu	Opituta	Opitutus	0	0	0	0	0	0	0	0	0	0	0	1	1
Cluster15386	Verrucom	Opituta	Opitu	Opituta	Opitutus	1	0	0	0	0	0	0	0	0	0	0	0	1
Cluster15389	Acidobact	Acidobacteria_Gp1			Gp1	1	0	0	0	0	0	0	0	0	0	0	0	1
Cluster15393	TM7				TM7_genera_i	1	0	0	0	0	0	0	0	0	0	0	0	1
Cluster15397;size=2						0	0	0	0	0	0	1	0	0	0	0	0	1
Cluster15401	Proteobac	Gamm	Xantl	Xanthor	Rhodanobacte	0	0	0	0	0	0	1	0	0	0	0	0	1
Cluster15407	Acidobact	Acidobacteria_Gp1			Gp1	1	0	0	0	0	0	0	0	0	0	0	0	1
Cluster15413	Proteobac	Alphap	Rhodospirillales			0	0	0	1	0	0	0	0	0	0	0	0	1
Cluster15414	Acidobact	Acidobacteria_Gp2			Gp2	1	0	0	0	0	0	0	0	0	0	0	0	1
Cluster15419	Acidobact	Acidobacteria_Gp1				0	0	0	0	0	0	1	0	0	0	0	0	1
Cluster15422	Acidobact	Acidobacteria_Gp1			Gp1	0	0	0	0	0	0	0	0	0	0	0	1	1

Cluster15425	Armatimonadetes				Armatimonade	0	0	0	0	0	0	1	0	0	0	0	0	1
Cluster15429	Acidobact	Acidobacteria_Gp5			Gp5	0	0	0	0	0	0	0	1	0	0	0	0	1
Cluster15434	Proteobac	Alphaproteobacteria				0	0	0	0	0	0	0	1	0	0	0	0	1
Cluster15436	Acidobact	Acidobacteria_Gp2			Gp2	0	0	1	0	0	0	0	0	0	0	0	0	1
Cluster15453	Proteobac	Gamm	Xantl	Xanthomonadaceae		0	0	0	0	1	0	0	0	0	0	0	0	1
Cluster15467	Acidobact	Acidobacteria_Gp2			Gp2	0	0	0	0	0	0	1	0	0	0	0	0	1
Cluster15474	Proteobac	Deltap	Myxococcales			0	0	0	0	0	0	0	0	0	0	0	1	1
Cluster15477	Acidobact	Acidobacteria_Gp1				0	0	0	0	0	0	1	0	0	0	0	0	1
Cluster15478	Actinobac	Actinol	Actinomycetales			0	0	1	0	0	0	0	0	0	0	0	0	1
Cluster15482	Proteobac	Alphaproteobacteria				0	0	0	0	0	0	1	0	0	0	0	0	1
Cluster15484	Acidobact	Acidobacteria_Gp1			Gp1	0	0	0	0	0	0	1	0	0	0	0	0	1
Cluster15485;size=1						0	0	0	0	0	0	0	0	0	0	0	1	1
Cluster15497	Proteobac	Deltaproteobacteria				0	0	0	1	0	0	0	0	0	0	0	0	1
Cluster15500	Chlamydia	Chlamy	Chlar	Parachlamydiaceae		1	0	0	0	0	0	0	0	0	0	0	0	1
Cluster15504	Acidobact	Acidobacteria_Gp1				0	0	0	0	0	0	0	1	0	0	0	0	1
Cluster15513;size=1						0	0	0	0	0	0	0	1	0	0	0	0	1
Cluster15514	Proteobacteria					0	1	0	0	0	0	0	0	0	0	0	0	1
Cluster15516	Proteobac	Gamm	Pseu	Pseudoi	Rugamonas	1	0	0	0	0	0	0	0	0	0	0	0	1
Cluster15518;size=3						0	0	0	0	0	0	0	1	0	0	0	0	1
Cluster15519;size=2						0	0	1	0	0	0	0	0	0	0	0	0	1
Cluster15520	Proteobac	Gamm	Xantl	Sinobac	Steroidobacter	1	0	0	0	0	0	0	0	0	0	0	0	1
Cluster15521	Acidobact	Acidobacteria_Gp1				1	0	0	0	0	0	0	0	0	0	0	0	1
Cluster15523	Proteobac	Gammaproteobacteria				1	0	0	0	0	0	0	0	0	0	0	0	1
Cluster15525;size=1						0	0	0	0	0	0	0	1	0	0	0	0	1
Cluster15527;size=1						1	0	0	0	0	0	0	0	0	0	0	0	1
Cluster15532	Acidobact	Acidobacteria_Gp2			Gp2	0	0	0	1	0	0	0	0	0	0	0	0	1
Cluster15533	Chlamydia	Chlamy	Chlamydiales			0	0	0	0	0	0	1	0	0	0	0	0	1
Cluster15541	Acidobact	Acidobacteria_Gp3			Bryobacter	0	0	0	0	0	0	0	1	0	0	0	0	1
Cluster15547	Acidobact	Acidobacteria_Gp1			Gp1	0	0	0	0	0	1	0	0	0	0	0	0	1

Cluster15552	Acidobact	Acidobacteria_Gp3				0	0	0	0	0	0	0	1	0	0	0	0	1
Cluster15557	Proteobac	Deltaproteobacteria				0	0	0	0	0	0	0	0	0	0	0	1	1
Cluster15562	Acidobact	Acidobacteria_Gp2			Gp2	1	0	0	0	0	0	0	0	0	0	0	0	1
Cluster15563	Proteobac	Alphap	Sphir	Sphingomonadaceae		1	0	0	0	0	0	0	0	0	0	0	0	1
Cluster15564;size=1						1	0	0	0	0	0	0	0	0	0	0	0	1
Cluster15568	Acidobact	Acidobacteria_Gp1				0	0	0	0	0	0	0	0	1	0	0	0	1
Cluster15571;size=1						0	0	0	0	0	0	0	1	0	0	0	0	1
Cluster15574;size=2						0	0	0	1	0	0	0	0	0	0	0	0	1
Cluster15583	Acidobact	Acidobacteria_Gp2			Gp2	0	0	0	0	0	0	0	1	0	0	0	0	1
Cluster15589	Verrucom	Opituta	Opitu	Opituta	Opitutus	0	0	0	0	0	0	1	0	0	0	0	0	1
Cluster15594	Proteobac	Alphap	Rhizobiales			0	1	0	0	0	0	0	0	0	0	0	0	1
Cluster15597;size=3						0	0	1	0	0	0	0	0	0	0	0	0	1
Cluster15602	Acidobact	Acidobacteria_Gp2			Gp2	0	0	0	0	0	0	0	1	0	0	0	0	1
Cluster15604	Acidobact	Acidobacteria_Gp2			Gp2	0	0	0	0	0	0	0	0	0	0	1	0	1
Cluster15605	Bacteroid	Sphing	Sphir	Chitinophagaceae		0	1	0	0	0	0	0	0	0	0	0	0	1
Cluster15623	Acidobact	Acidobacteria_Gp1			Gp1	0	1	0	0	0	0	0	0	0	0	0	0	1
Cluster15630	Actinobac	Actinol	Soliru	Solirubr	Solirubrobacte	0	0	0	0	0	0	1	0	0	0	0	0	1
Cluster15636;size=2						0	0	0	0	0	0	0	1	0	0	0	0	1
Cluster15641	Proteobac	Betapr	Burkl	Oxalobacteraceae		0	0	0	0	0	0	0	1	0	0	0	0	1
Cluster15642;size=1						0	0	0	0	0	0	1	0	0	0	0	0	1
Cluster15644;size=2						0	0	0	1	0	0	0	0	0	0	0	0	1
Cluster15647;size=2						0	0	0	0	1	0	0	0	0	0	0	0	1
Cluster15657;size=4						0	0	0	0	0	0	0	0	0	1	0	0	1
Cluster15662;size=4						0	0	0	1	0	0	0	0	0	0	0	0	1
Cluster15666	Firmicutes	Bacilli	Bacill	Paeniba	Paenibacillus	0	0	0	0	0	0	0	1	0	0	0	0	1
Cluster15669	Proteobac	Gamm	Legio	Legione	Legionella	0	0	0	0	0	0	1	0	0	0	0	0	1
Cluster15675	Bacteroid	Sphing	Sphir	Chitinophagaceae		0	0	0	0	0	0	0	1	0	0	0	0	1
Cluster15682	Proteobac	Alphap	Rhizo	Xanthol	Pseudolabrys	0	0	0	0	0	0	1	0	0	0	0	0	1
Cluster15693;size=1						0	0	0	0	0	0	1	0	0	0	0	0	1

Cluster15694	Acidobact	Acidobacteria_Gp1			Gp1	0	0	0	0	0	0	0	1	0	0	0	0	1
Cluster15696	Acidobact	Acidobacteria_Gp2			Gp2	0	0	0	0	0	0	0	0	0	0	0	1	1
Cluster15697	Acidobact	Acidobacteria_Gp1			Gp1	0	0	0	0	0	0	0	1	0	0	0	0	1
Cluster15705	Verrucom	Opituta	Opitu	Opituta	Opitutus	0	0	0	0	0	0	0	1	0	0	0	0	1
Cluster15707	Acidobact	Acidobacteria_Gp3			Gp3	0	0	0	0	0	0	1	0	0	0	0	0	1
Cluster15708;size=6						0	0	0	0	0	0	1	0	0	0	0	0	1
Cluster15709	Acidobact	Acidobacteria_Gp1				0	0	0	0	0	0	0	0	0	0	1	0	1
Cluster15711	Proteobac	Betaproteobacteria				0	0	0	1	0	0	0	0	0	0	0	0	1
Cluster15713	Acidobact	Acidobacteria_Gp1			Granulicella	0	0	0	0	0	0	0	0	0	0	1	0	1
Cluster15714	Proteobac	Alphap	Rhod	Acetoba	Acidisphaera	0	1	0	0	0	0	0	0	0	0	0	0	1
Cluster15715	Bacteroid	Sphing	Sphir	Chitinophagaceae		0	0	0	0	1	0	0	0	0	0	0	0	1
Cluster15723;size=1						0	1	0	0	0	0	0	0	0	0	0	0	1
Cluster15726	Proteobac	Alphap	Rhizobiales			0	0	0	0	0	0	0	1	0	0	0	0	1
Cluster15731	Acidobact	Acidobacteria_Gp1			Gp1	0	0	0	1	0	0	0	0	0	0	0	0	1
Cluster15741;size=1						0	0	0	0	0	0	0	0	0	0	0	1	1
Cluster15743	Bacteroid	Sphing	Sphir	Chitinophagaceae		0	0	0	0	1	0	0	0	0	0	0	0	1
Cluster15749	Proteobac	Betapr	Burkl	Burkholderiaceae		0	0	0	0	0	0	0	1	0	0	0	0	1
Cluster15758	Proteobac	Alphaproteobacteria				0	0	0	0	0	0	0	1	0	0	0	0	1
Cluster15761	Verrucom	Opitutae				0	0	0	0	0	0	0	0	0	0	0	1	1
Cluster15764	Bacteroid	Sphing	Sphir	Chitinophagaceae		0	0	0	0	0	0	0	1	0	0	0	0	1
Cluster15768	Gemmatir	Gemm	Gemi	Gemma	Gemmatimona	0	0	0	0	0	0	1	0	0	0	0	0	1
Cluster15787	Proteobac	Deltap	Myxococcales			0	0	0	0	0	0	0	0	1	0	0	0	1
Cluster15790	Proteobac	Alphap	Rhizobiales			0	0	0	1	0	0	0	0	0	0	0	0	1
Cluster15803	Proteobacteria					0	0	1	0	0	0	0	0	0	0	0	0	1
Cluster15810	Acidobact	Acidobacteria_Gp2			Gp2	0	0	1	0	0	0	0	0	0	0	0	0	1
Cluster15816	Acidobact	Acidobacteria_Gp3			Gp3	0	0	0	0	1	0	0	0	0	0	0	0	1
Cluster15823;size=1						0	0	0	0	0	0	0	1	0	0	0	0	1
Cluster15837	Acidobact	Acidobacteria_Gp1			Granulicella	0	1	0	0	0	0	0	0	0	0	0	0	1
Cluster15851;size=2						0	0	0	0	0	0	0	0	0	0	1	0	1

Cluster15855	Chlamydia	Chlamy	Chlar	Parachlamydiaceae		0	1	0	0	0	0	0	0	0	0	0	0	1
Cluster15856	Proteobac	Alphap	Rhodospirillales			0	0	0	1	0	0	0	0	0	0	0	0	1
Cluster15859	Proteobac	Betaproteobacteria				0	0	0	0	0	0	0	1	0	0	0	0	1
Cluster15862	Proteobac	Alphap	Rhodospirillales			0	0	0	0	0	0	1	0	0	0	0	0	1
Cluster15871	Verrucom	Subdivision3			Subdivision3_g	0	0	0	0	0	0	0	1	0	0	0	0	1
Cluster15892;size=2						0	0	0	0	0	0	0	0	0	0	1	0	1
Cluster15900	Armatimonadetes				Armatimonade	0	0	0	0	0	0	0	0	1	0	0	0	1
Cluster15907	Proteobac	Alphap	Sphir	Sphingomonadaceae		0	0	0	0	0	0	0	1	0	0	0	0	1
Cluster15908	Acidobact	Acidobacteria_Gp2			Gp2	0	0	0	0	0	0	0	0	0	0	0	1	1
Cluster15910	Proteobac	Deltaproteobacteria				0	0	0	0	0	0	1	0	0	0	0	0	1
Cluster15927;size=1						0	0	0	0	0	0	1	0	0	0	0	0	1
Cluster15928;size=9						0	0	0	1	0	0	0	0	0	0	0	0	1
Cluster15934;size=3						0	1	0	0	0	0	0	0	0	0	0	0	1
Cluster15939	Acidobact	Acidobacteria_Gp1			Gp1	0	0	0	0	0	0	0	1	0	0	0	0	1
Cluster15943	Proteobac	Alphap	Rhizc	Bradyrh	Bradyrhizobiur	0	1	0	0	0	0	0	0	0	0	0	0	1
Cluster15945	Proteobac	Alphap	Caulc	Caulobacteraceae		0	0	0	0	0	0	1	0	0	0	0	0	1
Cluster15953	Acidobact	Acidobacteria_Gp1			Edaphobacter	0	0	0	0	0	0	0	0	0	1	0	0	1
Cluster15960	Actinobac	Actinol	Actin	Nocardi	Rhodococcus	0	0	0	0	0	0	0	0	0	0	0	1	1
Cluster15967	Acidobact	Acidobacteria_Gp1			Edaphobacter	0	0	0	0	0	0	0	0	0	0	1	0	1
Cluster15972;size=1						0	0	0	0	0	0	1	0	0	0	0	0	1
Cluster15976	Proteobac	Alphap	Alphaproteob		Rhizomicrobiu	0	0	0	0	1	0	0	0	0	0	0	0	1
Cluster15996	Acidobact	Acidobacteria_Gp1				0	0	1	0	0	0	0	0	0	0	0	0	1
Cluster16000	Acidobact	Acidobacteria_Gp2			Gp2	0	0	0	0	0	0	0	0	1	0	0	0	1
Cluster16003	Acidobact	Acidobacteria_Gp1			Edaphobacter	0	0	0	0	0	0	0	1	0	0	0	0	1
Cluster16006;size=1						0	0	0	0	0	0	0	0	0	0	0	1	1
Cluster16013	Acidobact	Acidobacteria_Gp2			Gp2	0	0	0	1	0	0	0	0	0	0	0	0	1
Cluster16017;size=3						0	0	0	1	0	0	0	0	0	0	0	0	1
Cluster16018	Acidobact	Acidobacteria_Gp3				0	0	0	0	0	0	0	1	0	0	0	0	1
Cluster16020	Proteobac	Gammaproteobacteria				0	0	0	0	0	0	1	0	0	0	0	0	1

Cluster16035	Acidobact	Acidobacteria_Gp10			Gp10	0	0	0	1	0	0	0	0	0	0	0	0	1
Cluster16052	Acidobact	Acidobacteria_Gp3			Gp3	0	0	0	0	0	0	0	0	0	0	0	1	1
Cluster16053	Proteobac	Alphap	Rhizobiales			0	0	0	0	0	0	0	1	0	0	0	0	1
Cluster16070	Proteobac	Alphap	Caulo	Cauloba	Phenylobacter	0	0	0	0	0	0	0	1	0	0	0	0	1
Cluster16076	Proteobac	Alphap	Rhizobiales			0	0	0	0	0	0	0	1	0	0	0	0	1
Cluster16078	Proteobac	Alphap	Rhizo	Bradyrhizobiaceae		0	0	0	0	0	0	0	1	0	0	0	0	1
Cluster16082	Chlamydia	Chlamy	Chlar	Parachla	Neochlamydia	0	0	0	0	0	0	1	0	0	0	0	0	1
Cluster16084	Actinobac	Actinob	Actinomycetales			0	0	0	0	0	0	0	1	0	0	0	0	1
Cluster16090	Acidobact	Acidobacteria_Gp1			Gp1	0	0	0	0	0	1	0	0	0	0	0	0	1
Cluster16091	Proteobac	Alphap	Rhod	Acetobacteraceae		0	1	0	0	0	0	0	0	0	0	0	0	1
Cluster16094	Proteobacteria					0	0	0	0	0	0	1	0	0	0	0	0	1
Cluster16104;size=1						0	0	0	0	0	0	0	1	0	0	0	0	1
Cluster16115	Proteobac	Alphaproteobacteria				0	0	0	0	0	0	0	1	0	0	0	0	1
Cluster16127	Verrucom	Spartobacteria			Spartobacteria	0	0	0	1	0	0	0	0	0	0	0	0	1
Cluster16132	Acidobact	Acidobacteria_Gp2			Gp2	0	0	0	0	0	0	0	0	0	0	1	0	1
Cluster16134	Acidobact	Acidobacteria_Gp6			Gp6	0	0	0	0	0	0	0	0	1	0	0	0	1
Cluster16137;size=1						0	0	0	1	0	0	0	0	0	0	0	0	1
Cluster16138	Proteobac	Alphap	Rhizobiales			0	0	0	0	1	0	0	0	0	0	0	0	1
Cluster16150;size=15						0	0	0	0	0	0	0	0	1	0	0	0	1
Cluster16168	Proteobac	Gamm	Xanth	Xanthor	Frateuria	0	0	0	0	0	0	1	0	0	0	0	0	1
Cluster16174	Proteobacteria					0	0	0	0	0	0	0	0	0	0	1	0	1
Cluster16181	Proteobac	Deltap	Myxococcales			0	0	0	0	0	0	0	0	0	0	1	0	1
Cluster16185	Acidobact	Acidobacteria_Gp1			Gp1	0	0	0	0	1	0	0	0	0	0	0	0	1
Cluster16186	Chlamydia	Chlamy	Chlamydiales			0	0	0	0	0	0	1	0	0	0	0	0	1
Cluster16191	Acidobact	Acidobacteria_Gp1				0	0	0	0	0	1	0	0	0	0	0	0	1
Cluster16195	Acidobact	Acidobacteria_Gp2			Gp2	0	0	1	0	0	0	0	0	0	0	0	0	1
Cluster16196	Verrucom	Opituta	Opitu	Opituta	Opitutus	0	0	0	0	0	1	0	0	0	0	0	0	1
Cluster16198	Proteobacteria					0	0	0	0	1	0	0	0	0	0	0	0	1
Cluster16200	Proteobacteria					0	0	1	0	0	0	0	0	0	0	0	0	1

Cluster16201	Armatimo	Armati	Arma	Armatin	Armatimonas/	0	0	0	1	0	0	0	0	0	0	0	0	1
Cluster16202	Proteobac	Alphap	Alphaproteob		Rhizomicrobiu	0	0	0	1	0	0	0	0	0	0	0	0	1
Cluster16209	Acidobact	Acidobacteria_Gp1				0	0	0	0	1	0	0	0	0	0	0	0	1
Cluster16210	Proteobac	Alphap	Rhizo	Xanthob	Pseudolabrys	0	0	0	0	1	0	0	0	0	0	0	0	1
Cluster16213	Acidobact	Acidobacteria_Gp1			Gp1	0	0	0	1	0	0	0	0	0	0	0	0	1
Cluster16220;size=1						0	0	0	0	0	1	0	0	0	0	0	0	1
Cluster16223	Acidobact	Acidobacteria_Gp1			Gp1	0	0	0	0	1	0	0	0	0	0	0	0	1
Cluster16224	Actinobac	Actinol	Actinomycetales			0	1	0	0	0	0	0	0	0	0	0	0	1
Cluster16225	Acidobact	Acidobacteria_Gp2			Gp2	0	0	0	0	0	0	0	0	0	0	1	0	1
Cluster16230	Proteobac	Alphaproteobacteria				0	0	0	0	0	1	0	0	0	0	0	0	1
Cluster16233	Proteobac	Deltap	Myxococcales			0	0	0	0	0	1	0	0	0	0	0	0	1
Cluster16238	Acidobact	Acidobacteria_Gp1			Gp1	0	0	0	0	1	0	0	0	0	0	0	0	1
Cluster16252;size=3						0	0	0	0	0	0	0	0	1	0	0	0	1
Cluster16254	Verrucom	Opituta	Opitu	Opituta	Opitutus	1	0	0	0	0	0	0	0	0	0	0	0	1
Cluster16256	Proteobac	Gammaproteobacteria				0	0	0	0	1	0	0	0	0	0	0	0	1
Cluster16257;size=4						0	0	0	1	0	0	0	0	0	0	0	0	1
Cluster16258	Acidobact	Acidobacteria_Gp1				0	0	1	0	0	0	0	0	0	0	0	0	1
Cluster16262	Acidobact	Acidobacteria_Gp3				0	0	0	0	0	1	0	0	0	0	0	0	1
Cluster16263	Acidobact	Acidobacteria_Gp2			Gp2	0	0	0	1	0	0	0	0	0	0	0	0	1
Cluster16269	Acidobact	Acidobacteria_Gp3			Gp3	0	0	0	0	0	0	0	0	1	0	0	0	1
Cluster16272;size=2						0	0	0	0	0	1	0	0	0	0	0	0	1
Cluster16278;size=5						0	0	0	0	0	0	0	0	0	0	1	0	1
Cluster16281	Verrucom	Opituta	Opitu	Opituta	Opitutus	0	0	0	0	0	0	0	0	0	1	0	0	1
Cluster16286	Acidobact	Acidobacteria_Gp1				0	0	0	0	0	0	0	1	0	0	0	0	1
Cluster16288	Acidobact	Acidobacteria_Gp1			Gp1	0	0	0	0	0	0	0	0	0	0	0	1	1
Cluster16289	Acidobact	Acidobacteria_Gp1			Gp1	0	0	0	1	0	0	0	0	0	0	0	0	1
Cluster16297	Acidobact	Acidobacteria_Gp1				0	0	0	0	0	0	0	0	0	0	0	1	1
Cluster16298	Proteobacteria					0	0	0	1	0	0	0	0	0	0	0	0	1
Cluster16300	Verrucom	Opituta	Opitu	Opituta	Opitutus	0	0	0	1	0	0	0	0	0	0	0	0	1

Cluster16301	Acidobact	Acidobacteria_Gp2			Gp2	0	0	0	1	0	0	0	0	0	0	0	0	1
Cluster16305	Acidobact	Acidobacteria_Gp2			Gp2	0	0	0	0	0	0	0	0	0	1	0	0	1
Cluster16307	Proteobac	Alphap	Rhod	Rhodospirillaceae		0	0	0	1	0	0	0	0	0	0	0	0	1
Cluster16309	Bacteroid	Sphing	Sphir	Chitinophagaceae		0	0	1	0	0	0	0	0	0	0	0	0	1
Cluster16310	Proteobac	Betaproteobacteria				0	0	0	1	0	0	0	0	0	0	0	0	1
Cluster16326;size=3						0	0	0	1	0	0	0	0	0	0	0	0	1
Cluster16327;size=3						0	0	0	0	0	0	0	0	0	0	1	0	1
Cluster16332;size=6						0	0	1	0	0	0	0	0	0	0	0	0	1
Cluster16353	Acidobact	Acidobacteria_Gp2			Gp2	0	0	0	1	0	0	0	0	0	0	0	0	1
Cluster16354	Proteobac	Deltaproteobacteria				0	0	0	0	0	0	0	0	0	0	1	0	1
Cluster16368	Acidobact	Acidobacteria_Gp3			Gp3	0	0	0	0	1	0	0	0	0	0	0	0	1
Cluster16370	Proteobac	Alphap	Rhizobiales			0	0	0	1	0	0	0	0	0	0	0	0	1
Cluster16372	Actinobac	Actinol	Soliru	Conexib	Conexibacter	0	0	0	0	0	0	0	0	0	0	1	0	1
Cluster16376	Acidobact	Acidobacteria_Gp1			Gp1	0	0	0	0	0	0	0	0	0	0	1	0	1
Cluster16379	Actinobac	Actinol	Actinomycetales			0	0	1	0	0	0	0	0	0	0	0	0	1
Cluster16387	Proteobac	Betaproteobacteria				0	0	0	0	1	0	0	0	0	0	0	0	1
Cluster16389	Proteobac	Alphaproteobacteria				0	0	1	0	0	0	0	0	0	0	0	0	1
Cluster16394	Acidobact	Acidobacteria_Gp2			Gp2	0	0	0	1	0	0	0	0	0	0	0	0	1
Cluster16400;size=2						0	0	1	0	0	0	0	0	0	0	0	0	1
Cluster16404	Proteobac	Alphap	Caulo	Cauloba	Phenylobacter	0	0	1	0	0	0	0	0	0	0	0	0	1
Cluster16413	Verrucom	Opitutae				0	0	0	1	0	0	0	0	0	0	0	0	1
Cluster16414	Actinobac	Actinol	Acidimicrobiales			0	0	1	0	0	0	0	0	0	0	0	0	1
Cluster16422	Actinobac	Actinol	Soliru	Conexib	Conexibacter	0	0	0	0	0	0	0	0	0	0	1	0	1
Cluster16424;size=2						0	0	0	0	0	0	0	0	0	0	1	0	1
Cluster16430	Actinobac	Actinol	Solirubrobacterales			0	0	0	0	0	0	0	0	0	0	1	0	1
Cluster16432	Bacteroid	Sphing	Sphir	Chitinophagaceae		0	0	0	1	0	0	0	0	0	0	0	0	1
Cluster16434	Acidobact	Acidobacteria_Gp2			Gp2	0	0	0	0	1	0	0	0	0	0	0	0	1
Cluster16438	Acidobact	Acidobacteria_Gp1				0	0	0	1	0	0	0	0	0	0	0	0	1
Cluster16439	Verrucom	Subdivision3			Subdivision3_g	0	0	0	0	0	0	0	0	0	1	0	0	1

Cluster16441	Acidobact	Acidobacteria_Gp1			Gp1	0	0	0	1	0	0	0	0	0	0	0	0	1
Cluster16443	Proteobac	Alphaproteobacteria				0	0	1	0	0	0	0	0	0	0	0	0	1
Cluster16454;size=1						0	0	0	0	0	0	0	0	1	0	0	0	1
Cluster16455;size=1						0	0	0	1	0	0	0	0	0	0	0	0	1
Cluster16456	Bacteroid	Sphing	Sphir	Chitinophagaceae		0	0	0	1	0	0	0	0	0	0	0	0	1
Cluster16461	Acidobact	Acidobacteria_Gp10			Gp10	0	0	0	0	0	0	0	0	1	0	0	0	1
Cluster16472	Acidobact	Acidobacteria_Gp2			Gp2	0	0	0	1	0	0	0	0	0	0	0	0	1
Cluster16476	Bacteroid	Sphing	Sphir	Chitinophagaceae		0	0	0	1	0	0	0	0	0	0	0	0	1
Cluster16481	Bacteroid	Sphing	Sphir	Chitinophagaceae		0	0	1	0	0	0	0	0	0	0	0	0	1
Cluster16487	Cyanobac	Chloroplast		Chlorop	Chlorophyta	0	0	0	0	0	0	0	0	0	0	1	0	1
Cluster16492	Verrucom	Opituta	Opitu	Opituta	Opitutus	0	0	0	0	0	1	0	0	0	0	0	0	1
Cluster16493	Acidobact	Acidobacteria_Gp1			Gp1	0	0	0	1	0	0	0	0	0	0	0	0	1
Cluster16501	Acidobact	Acidobacteria_Gp2			Gp2	1	0	0	0	0	0	0	0	0	0	0	0	1
Cluster16505	Proteobac	Gammaproteobacteria				0	0	0	0	0	0	0	0	0	0	1	0	1
Cluster16511	Acidobact	Acidobacteria_Gp10			Gp10	0	0	0	0	0	0	0	0	0	0	1	0	1
Cluster16517;size=1						0	0	0	1	0	0	0	0	0	0	0	0	1
Cluster16519	Proteobac	Deltap	Myxc	Cystobacteraceae		0	0	0	1	0	0	0	0	0	0	0	0	1
Cluster16522	Actinobac	Actinol	Acidi	Acidimi	Ferrithrix	0	0	0	0	1	0	0	0	0	0	0	0	1
Cluster16529	Bacteroid	Sphing	Sphir	Chitinophagaceae		0	0	0	0	0	0	0	0	0	1	0	0	1
Cluster16530	Proteobac	Gammaproteobacteria				0	0	0	0	1	0	0	0	0	0	0	0	1
Cluster16536	Verrucom	Subdivision3			Subdivision3_g	0	0	0	0	0	0	0	0	0	0	1	0	1
Cluster16544	Acidobact	Acidobacteria_Gp1				0	0	0	0	0	0	0	0	0	0	1	0	1
Cluster16545	Verrucom	Opituta	Opitu	Opituta	Opitutus	0	0	1	0	0	0	0	0	0	0	0	0	1
Cluster16546	Acidobact	Acidobacteria_Gp1			Gp1	0	0	0	1	0	0	0	0	0	0	0	0	1
Cluster16549;size=1						0	0	0	1	0	0	0	0	0	0	0	0	1
Cluster16558	Proteobac	Alphap	Rhod	Acetoba	Acidisphaera	0	0	0	1	0	0	0	0	0	0	0	0	1
Cluster16559	Proteobac	Deltap	Bdell	Bdellovi	Bdellovibrio	0	0	0	0	0	0	0	0	1	0	0	0	1
Cluster16574	Verrucom	Subdivision3			Subdivision3_g	0	0	1	0	0	0	0	0	0	0	0	0	1
Cluster16580	Verrucom	Subdivision3			Subdivision3_g	0	0	0	0	1	0	0	0	0	0	0	0	1

Cluster16584	Verrucom	Opituta	Opitu	Opituta	Opitutus	0	0	0	0	0	0	0	0	1	0	0	0	1
Cluster16590	Acidobact	Acidobacteria_Gp6			Gp6	0	0	0	0	1	0	0	0	0	0	0	0	1
Cluster16595	Acidobact	Acidobacteria_Gp3				0	0	0	1	0	0	0	0	0	0	0	0	1
Cluster16596;size=1						0	0	0	0	1	0	0	0	0	0	0	0	1
Cluster16601	Acidobact	Acidobacteria_Gp3			Bryobacter	0	0	0	0	0	0	0	0	0	0	1	0	1
Cluster16603	Acidobact	Acidobacteria_Gp1			Gp1	0	0	0	0	0	0	0	0	0	1	0	0	1
Cluster16610;size=1						0	0	0	0	1	0	0	0	0	0	0	0	1
Cluster16614	Acidobact	Acidobacteria_Gp1			Edaphobacter	0	0	0	1	0	0	0	0	0	0	0	0	1
Cluster16627	Acidobact	Acidobacteria_Gp1				0	0	0	0	0	1	0	0	0	0	0	0	1
Cluster16628	Proteobac	Alphap	Rhod	Acetoba	Acidisphaera	0	0	0	0	0	0	0	0	0	0	1	0	1
Cluster16637	Proteobac	Alphap	Rhizo	Methylocystaceae		0	0	0	1	0	0	0	0	0	0	0	0	1
Cluster16642	Actinobac	Actinol	Actinomycetales			0	0	0	0	0	0	0	0	0	0	0	1	1
Cluster16647	Acidobact	Acidobacteria_Gp1				0	0	0	1	0	0	0	0	0	0	0	0	1
Cluster16650	Proteobac	Gammaproteobacteria				0	0	0	0	0	0	0	0	0	1	0	0	1
Cluster16652	Bacteroid	Sphing	Sphir	Chitinophagaceae		0	0	0	0	0	0	0	0	0	0	1	0	1
Cluster16654	Actinobac	Actinol	Actin	Mycoba	Mycobacteriur	0	0	0	1	0	0	0	0	0	0	0	0	1
Cluster16660	Proteobac	Alphap	Rhizobiales			0	0	1	0	0	0	0	0	0	0	0	0	1
Cluster16661	Acidobact	Acidobacteria_Gp1			Gp1	0	0	0	1	0	0	0	0	0	0	0	0	1
Cluster16666	Verrucom	Subdivision3			Subdivision3_g	0	0	0	0	1	0	0	0	0	0	0	0	1
Cluster16669	Acidobact	Acidobacteria_Gp1			Gp1	0	0	0	0	0	1	0	0	0	0	0	0	1
Cluster16672	Proteobac	Deltap	Myxo	Polyangiaceae		0	0	0	0	1	0	0	0	0	0	0	0	1
Cluster16678;size=2						0	0	0	0	1	0	0	0	0	0	0	0	1
Cluster16686	Proteobac	Alphap	Rhodospirillales			0	0	0	0	1	0	0	0	0	0	0	0	1
Cluster16687	Proteobac	Alphap	Rhizobiales			0	0	1	0	0	0	0	0	0	0	0	0	1
Cluster16688	Proteobac	Betapr	Burkl	Oxalobacteraceae		0	0	1	0	0	0	0	0	0	0	0	0	1
Cluster16692	Actinobac	Actinol	Actin	Pseudonocardiaceae		0	0	0	0	0	0	0	0	0	0	1	0	1
Cluster16696	Proteobac	Alphap	Rhizo	Rhizobia	Vasilyevaea	0	0	1	0	0	0	0	0	0	0	0	0	1
Cluster16698	Verrucom	Opituta	Opitu	Opituta	Opitutus	0	0	0	0	0	0	0	0	0	1	0	0	1
Cluster16703	Acidobact	Acidobacteria_Gp1				0	0	0	0	0	0	0	0	0	0	1	0	1

Cluster16707	Proteobac	Deltap	Myxc	Polyangiaceae		0	0	0	0	1	0	0	0	0	0	0	0	1
Cluster16713;size=2						0	0	0	0	1	0	0	0	0	0	0	0	1
Cluster16715	Proteobac	Deltap	Myxc	Polyangiaceae		0	0	0	0	0	0	0	0	0	0	1	0	1
Cluster16720;size=1						0	0	0	0	0	0	0	0	0	1	0	0	1
Cluster16727;size=1						0	0	0	0	1	0	0	0	0	0	0	0	1
Cluster16733	Verrucom	Spartobacteria			Spartobacteria	0	0	0	0	1	0	0	0	0	0	0	0	1
Cluster16734	Proteobac	Alphap	Rhizc	Xanthol	Pseudolabrys	0	0	0	1	0	0	0	0	0	0	0	0	1
Cluster16744	Proteobac	Alphap	Alphaproteob		Rhizomicrobiu	0	0	0	1	0	0	0	0	0	0	0	0	1
Cluster16749	Acidobact	Acidobacteria_Gp2			Gp2	0	0	0	1	0	0	0	0	0	0	0	0	1
Cluster16751	Proteobac	Alphap	Alphaproteob		Rhizomicrobiu	0	0	0	0	0	0	0	0	0	1	0	0	1
Cluster16757	Chlamydia	Chlamy	Chlar	Parachla	Parachlamydia	0	1	0	0	0	0	0	0	0	0	0	0	1
Cluster16758	Proteobac	Deltap	Myxococcales			0	0	0	0	1	0	0	0	0	0	0	0	1
Cluster16762	Acidobact	Acidobacteria_Gp1				0	0	0	1	0	0	0	0	0	0	0	0	1
Cluster16767	Acidobact	Acidobacteria_Gp2			Gp2	0	0	0	0	1	0	0	0	0	0	0	0	1
Cluster16769	Proteobac	Gamm	Xantl	Sinobac	Steroidobacter	0	0	1	0	0	0	0	0	0	0	0	0	1
Cluster16770	Proteobac	Alphap	Alphaproteob		Rhizomicrobiu	0	0	1	0	0	0	0	0	0	0	0	0	1
Cluster16772	Proteobacteria					0	0	1	0	0	0	0	0	0	0	0	0	1
Cluster16773	Proteobac	Betapr	Burkl	Oxalobacteraceae		0	0	0	1	0	0	0	0	0	0	0	0	1
Cluster16775	Verrucom	Subdivision3			Subdivision3_g	0	0	0	0	0	0	0	0	0	0	1	0	1
Cluster16781;size=2						0	0	0	0	1	0	0	0	0	0	0	0	1
Cluster16788	Acidobact	Acidobacteria_Gp1				0	0	0	0	0	0	0	0	0	0	1	0	1
Cluster16789;size=1						0	0	0	1	0	0	0	0	0	0	0	0	1
Cluster16793	Proteobac	Betaproteobacteria				0	0	0	0	0	0	0	1	0	0	0	0	1
Cluster16797	Acidobact	Acidobacteria_Gp13			Gp13	0	0	0	0	0	0	0	0	0	0	1	0	1
Cluster16809	Proteobac	Deltap	Myxococcales			0	0	1	0	0	0	0	0	0	0	0	0	1
Cluster16810	Acidobact	Acidobacteria_Gp1			Gp1	0	0	0	1	0	0	0	0	0	0	0	0	1
Cluster16814	Proteobac	Gamm	Xanthomonadales			0	0	0	0	0	0	0	0	0	1	0	0	1
Cluster16821	Acidobact	Acidobacteria_Gp1			Granulicella	0	0	0	0	1	0	0	0	0	0	0	0	1
Cluster16827	Proteobac	Alphap	Alphaproteob		Rhizomicrobiu	0	0	0	0	0	0	0	0	0	0	1	0	1

Cluster16831	Acidobact	Acidobacteria_Gp1				0	0	0	0	0	0	0	0	0	0	1	0	1
Cluster16832	Gemmatii	Gemm	Gemi	Gemma	Gemmatimona	0	0	0	0	0	0	0	0	0	0	1	0	1
Cluster16835	Acidobact	Acidobacteria_Gp10			Gp10	0	0	0	1	0	0	0	0	0	0	0	0	1
Cluster16838	Acidobact	Acidobacteria_Gp1			Edaphobacter	0	0	0	1	0	0	0	0	0	0	0	0	1
Cluster16855	Proteobac	Betapr	Rhod	Rhodocyclaceae		0	0	1	0	0	0	0	0	0	0	0	0	1
Cluster16859	Proteobac	Gammaproteobacteria				0	0	1	0	0	0	0	0	0	0	0	0	1
Cluster16866	Proteobac	Alphap	Rhod	Acetoba	Acidisphaera	0	0	0	0	0	0	0	0	1	0	0	0	1
Cluster16874	Acidobact	Acidobacteria_Gp1			Gp1	0	0	0	0	0	1	0	0	0	0	0	0	1
Cluster16876	Acidobact	Acidobacteria_Gp3			Gp3	0	0	0	0	0	0	0	0	0	1	0	0	1
Cluster16878	Proteobac	Alphaproteobacteria				0	0	0	0	1	0	0	0	0	0	0	0	1
Cluster16880	Proteobac	Gammaproteobacteria				0	0	0	0	0	0	0	0	1	0	0	0	1
Cluster16881;size=1						0	0	0	0	1	0	0	0	0	0	0	0	1
Cluster16884	Acidobact	Acidobacteria_Gp2			Gp2	0	0	0	0	1	0	0	0	0	0	0	0	1
Cluster16889	Acidobact	Acidobacteria_Gp1			Gp1	0	0	0	1	0	0	0	0	0	0	0	0	1
Cluster16895	Proteobac	Gamm	Xantl	Sinobac	Steroidobacter	0	0	0	0	0	0	0	0	0	0	1	0	1
Cluster16897	Acidobact	Acidobacteria_Gp1			Gp1	0	0	0	0	0	0	0	0	0	0	1	0	1
Cluster16902	Acidobacteria					0	0	0	0	0	0	0	0	0	0	1	0	1
Cluster16917	Proteobac	Gammaproteobacteria				1	0	0	0	0	0	0	0	0	0	0	0	1
Cluster16918	Proteobac	Deltaproteobacteria				0	0	0	0	0	0	0	0	1	0	0	0	1
Cluster16923	Acidobact	Acidobacteria_Gp1				0	1	0	0	0	0	0	0	0	0	0	0	1
Cluster16926	Chloroflex	Ktedor	Ktedonobacterales			0	0	0	0	0	1	0	0	0	0	0	0	1
Cluster16927	Proteobac	Betapr	Burkl	Burkhol	Burkholderia	0	0	0	0	1	0	0	0	0	0	0	0	1
Cluster16928	Acidobact	Acidobacteria_Gp1			Gp1	0	0	1	0	0	0	0	0	0	0	0	0	1
Cluster16942	Proteobac	Alphap	Alphaproteol		Rhizomicrobiu	0	0	0	0	0	0	0	0	0	0	1	0	1
Cluster16951	Bacteroid	Sphing	Sphir	Chitinop	Flavitalea	0	0	0	1	0	0	0	0	0	0	0	0	1
Cluster16958	Proteobac	Alphap	Rhodospirillales			0	0	0	0	0	0	0	0	0	0	1	0	1
Cluster16959	Acidobact	Acidobacteria_Gp1				0	0	0	0	0	0	0	0	0	0	1	0	1
Cluster16967;size=1						0	0	0	1	0	0	0	0	0	0	0	0	1
Cluster16969	Acidobact	Acidobacteria_Gp2			Gp2	0	0	0	0	0	0	0	0	0	1	0	0	1

Cluster16972	Proteobac	Alphap	Alphaproteob		Rhizomicrobiu	0	0	1	0	0	0	0	0	0	0	0	0	1
Cluster16974	Proteobac	Alphap	Caulo	Culoba	Phenylobacter	1	0	0	0	0	0	0	0	0	0	0	0	1
Cluster16975	Verrucom	Opitut	Opitu	Opituta	Opitutus	0	0	0	1	0	0	0	0	0	0	0	0	1
Cluster16978	Bacteroid	Sphing	Sphir	Chitinophagaceae		0	0	0	1	0	0	0	0	0	0	0	0	1
Cluster16985	Firmicutes	Bacilli	Bacill	Paenibacillaceae1		0	0	0	1	0	0	0	0	0	0	0	0	1
Cluster16995	Gemmatir	Gemm	Gemi	Gemma	Gemmatimona	0	0	0	1	0	0	0	0	0	0	0	0	1
Cluster16997	Proteobac	Gamm	Xantl	Sinobac	Steroidobacter	0	0	0	0	0	1	0	0	0	0	0	0	1
Cluster16998	Acidobact	Acidobacteria_Gp6			Gp6	0	0	0	0	0	0	0	0	1	0	0	0	1
Cluster17004	Proteobac	Deltaproteobacteria				0	0	0	0	1	0	0	0	0	0	0	0	1
Cluster17005	Proteobac	Alphap	Alphaproteob		Rhizomicrobiu	0	0	1	0	0	0	0	0	0	0	0	0	1
Cluster17008	Actinobac	Actinol	Solirı	Conexib	Conexibacter	0	0	0	0	0	0	0	0	0	0	1	0	1
Cluster17010;size=1						0	0	0	0	0	0	0	0	0	0	1	0	1
Cluster17013	Proteobac	Alphap	Rhod	Acetobacteraceae		0	0	0	0	0	0	0	0	0	0	1	0	1
Cluster17015	Proteobac	Betapr	Burkl	Oxalobacteraceae		0	0	0	1	0	0	0	0	0	0	0	0	1
Cluster17018;size=2						0	0	1	0	0	0	0	0	0	0	0	0	1
Cluster17019;size=2						0	0	0	1	0	0	0	0	0	0	0	0	1
Cluster17022	Proteobacteria					0	0	0	1	0	0	0	0	0	0	0	0	1
Cluster17027	Acidobact	Acidobacteria_Gp10			Gp10	0	0	0	0	0	0	0	1	0	0	0	0	1
Cluster17029	Proteobac	Gammaproteobacteria				0	0	0	1	0	0	0	0	0	0	0	0	1
Cluster17030	Acidobact	Acidobacteria_Gp2			Gp2	0	0	0	0	0	0	0	0	0	0	1	0	1
Cluster17032	Acidobact	Acidobacteria_Gp3			Gp3	0	0	0	1	0	0	0	0	0	0	0	0	1
Cluster17037	Proteobac	Alphaproteobacteria				0	0	0	0	0	0	1	0	0	0	0	0	1
Cluster17039	Acidobact	Acidobacteria_Gp10			Gp10	0	0	0	1	0	0	0	0	0	0	0	0	1
Cluster17040	Acidobact	Acidobacteria_Gp2			Gp2	0	0	0	1	0	0	0	0	0	0	0	0	1
Cluster17043	Chlamydia	Chlamy	Chlar	Parachla	Neochlamydia	0	0	0	0	0	1	0	0	0	0	0	0	1
Cluster17046	Acidobact	Acidobacteria_Gp3			Bryobacter	0	0	0	0	0	0	0	0	0	0	0	1	1
Cluster17057	Actinobac	Actinol	Actinomycetales			0	0	0	0	0	0	0	0	0	0	0	1	1
Cluster17062;size=1						0	0	1	0	0	0	0	0	0	0	0	0	1
Cluster17082	Proteobac	Alphap	Rhod	Acetoba	Acidisphaera	0	0	0	1	0	0	0	0	0	0	0	0	1

Cluster17085	Proteobacteria					0	0	0	1	0	0	0	0	0	0	0	0	1
Cluster17086	Proteobac	Alphap	Rhod	Acetoba	Acidisphaera	0	0	0	0	0	0	0	1	0	0	0	0	1
Cluster17087;size=1						0	0	0	0	0	1	0	0	0	0	0	0	1
Cluster17092	Actinobac	Actinol	Acidimicrobiales			0	0	0	0	1	0	0	0	0	0	0	0	1
Cluster17093	Acidobact	Acidobacteria_Gp3				0	0	0	0	0	0	0	0	0	0	1	0	1
Cluster17096	Actinobac	Actinol	Actin	Mycoba	Mycobacteriur	0	0	0	1	0	0	0	0	0	0	0	0	1
Cluster17099	Proteobac	Deltaproteobacteria				0	0	0	0	0	1	0	0	0	0	0	0	1
Cluster17107	Acidobact	Acidobacteria_Gp1				0	0	1	0	0	0	0	0	0	0	0	0	1
Cluster17110	Proteobac	Alphap	Rhod	Acetoba	Rhodopila	0	0	0	0	0	0	0	0	0	0	1	0	1
Cluster17118	Verrucom	Opitutae				0	0	0	0	0	0	0	0	1	0	0	0	1
Cluster17121	Acidobact	Acidobacteria_Gp2			Gp2	0	0	0	1	0	0	0	0	0	0	0	0	1
Cluster17123	Proteobac	Gammaproteobacteria				0	0	1	0	0	0	0	0	0	0	0	0	1
Cluster17128	Acidobact	Acidobacteria_Gp1			Edaphobacter	0	0	0	0	1	0	0	0	0	0	0	0	1
Cluster17129	Acidobact	Acidobacteria_Gp3			Gp3	0	0	0	0	0	0	0	0	1	0	0	0	1
Cluster17130	Verrucom	Opitut	Opitu	Opituta	Opitutus	0	0	0	0	0	0	0	0	0	1	0	0	1
Cluster17133;size=1						0	0	0	0	0	1	0	0	0	0	0	0	1
Cluster17137	Proteobac	Gammaproteobacteria				0	0	0	1	0	0	0	0	0	0	0	0	1
Cluster17151	Acidobact	Acidobacteria_Gp2			Gp2	0	0	1	0	0	0	0	0	0	0	0	0	1
Cluster17152	Proteobac	Gamm	Xantl	Sinobac	Steroidobacter	0	0	0	0	0	1	0	0	0	0	0	0	1
Cluster17156	Actinobac	Actinol	Actinomycetales			0	0	0	0	0	0	0	0	0	0	0	1	1
Cluster17157	Proteobac	Alphap	Rhizobiales			0	0	0	0	0	0	0	0	0	0	1	0	1
Cluster17159	TM7				TM7_genera_i	0	0	1	0	0	0	0	0	0	0	0	0	1
Cluster17162	Acidobact	Acidobacteria_Gp2			Gp2	0	0	0	0	0	0	0	1	0	0	0	0	1
Cluster17163	Proteobac	Alphaproteobacteria				0	0	0	0	0	0	0	0	0	0	1	0	1
Cluster17167	Actinobac	Actinol	Actin	Mycoba	Mycobacteriur	0	0	0	0	0	0	0	0	0	0	1	0	1
Cluster17173	Acidobact	Acidobacteria_Gp2			Gp2	0	0	0	0	0	0	0	0	0	0	1	0	1
Cluster17174	Acidobact	Acidobacteria_Gp1				0	0	0	1	0	0	0	0	0	0	0	0	1
Cluster17179	Proteobac	Alphap	Rhizc	Hyphon	Rhodoplanes	0	0	0	1	0	0	0	0	0	0	0	0	1
Cluster17181;size=5						0	0	0	1	0	0	0	0	0	0	0	0	1

Cluster17182	Acidobact	Acidobacteria_Gp1			Gp1	0	0	1	0	0	0	0	0	0	0	0	0	1
Cluster17191	Acidobact	Acidobacteria_Gp2			Gp2	0	0	0	0	1	0	0	0	0	0	0	0	1
Cluster17194	Proteobac	Alphap	Alphaproteob		Rhizomicrobiu	0	0	1	0	0	0	0	0	0	0	0	0	1
Cluster17200;size=1						0	0	0	0	0	0	0	0	0	0	1	0	1
Cluster17202	Proteobac	Alphap	Rhodospirillales			0	0	0	0	1	0	0	0	0	0	0	0	1
Cluster17204	Firmicutes	Bacilli	Bacill	Paeniba	Cohnella	0	0	0	0	0	0	0	0	0	0	1	0	1
Cluster17214	Proteobac	Deltaproteobacteria				0	0	0	1	0	0	0	0	0	0	0	0	1
Cluster17215	Proteobac	Gammaproteobacteria				0	0	0	0	1	0	0	0	0	0	0	0	1
Cluster17218	Actinobac	Actinol	Actinomycetales			0	0	0	1	0	0	0	0	0	0	0	0	1
Cluster17220	Acidobact	Acidobacteria_Gp1				0	0	1	0	0	0	0	0	0	0	0	0	1
Cluster17228	Acidobact	Acidobacteria_Gp3			Gp3	0	0	0	0	0	0	0	0	0	0	1	0	1
Cluster17229	Acidobact	Acidobacteria_Gp6			Gp6	0	0	0	0	0	0	0	0	0	0	0	1	1
Cluster17233;size=2						0	0	0	1	0	0	0	0	0	0	0	0	1
Cluster17237	Actinobac	Actinol	Actin	Actinos	Actinospica	0	0	0	0	1	0	0	0	0	0	0	0	1
Cluster17239	Acidobact	Acidobacteria_Gp2			Gp2	0	0	0	0	0	1	0	0	0	0	0	0	1
Cluster17241;size=1						0	0	0	0	0	0	0	0	0	0	1	0	1
Cluster17252;size=1						0	0	0	1	0	0	0	0	0	0	0	0	1
Cluster17256	Proteobac	Gamm	Xantl	Sinobac	Steroidobacter	0	0	1	0	0	0	0	0	0	0	0	0	1
Cluster17258	Verrucom	Subdivision3			Subdivision3_g	0	0	0	0	1	0	0	0	0	0	0	0	1
Cluster17268	Verrucom	Opitut	Opitu	Opituta	Opitutus	0	0	0	0	0	0	0	0	1	0	0	0	1
Cluster17273	Proteobac	Alphap	Rhod	Acetobacteraceae		0	0	0	1	0	0	0	0	0	0	0	0	1
Cluster17285	Acidobact	Acidobacteria_Gp1			Acidobacteriur	0	0	0	1	0	0	0	0	0	0	0	0	1
Cluster17289	Verrucom	Spartobacteria			Spartobacteria	0	0	0	0	0	1	0	0	0	0	0	0	1
Cluster17294	Acidobact	Acidobacteria_Gp1			Gp1	0	0	0	0	0	0	0	0	0	1	0	0	1
Cluster17299	Proteobacteria					0	0	1	0	0	0	0	0	0	0	0	0	1
Cluster17300	Proteobac	Alphap	Rhod	Acetoba	Acidisphaera	0	0	0	0	0	1	0	0	0	0	0	0	1
Cluster17309	Verrucomicrobia					0	0	0	0	0	0	0	0	0	0	1	0	1
Cluster17311	Acidobact	Acidobacteria_Gp3			Gp3	0	0	0	1	0	0	0	0	0	0	0	0	1
Cluster17313	Acidobact	Acidobacteria_Gp2			Gp2	0	0	0	0	1	0	0	0	0	0	0	0	1

Cluster17326;size=2						0	0	0	0	1	0	0	0	0	0	0	0	1
Cluster1733	Proteobac	Alphap	Rhizo	Hyphon	Rhodoplanes	0	0	0	0	1	0	0	0	0	0	0	0	1
Cluster1734C	Proteobac	Deltap	Myxococcales			0	0	0	0	0	1	0	0	0	0	0	0	1
Cluster1734Z	Proteobac	Alphap	Rhodospirillales			0	0	0	1	0	0	0	0	0	0	0	0	1
Cluster1734	Proteobac	Alphap	Sphir	Sphingo	Novosphingob	0	0	1	0	0	0	0	0	0	0	0	0	1
Cluster1734E	Actinobac	Actinol	Actin	Mycoba	Mycobacteriur	1	0	0	0	0	0	0	0	0	0	0	0	1
Cluster1734E	Acidobact	Acidobacteria_Gp1			Gp1	0	0	0	0	0	0	0	0	0	0	1	0	1
Cluster17351	Armatimo	Armati	Arma	Armatir	Armatimonas/	0	0	1	0	0	0	0	0	0	0	0	0	1
Cluster1735Z	Actinobac	Actinol	Actinomycetales			0	0	0	0	0	0	0	0	0	0	0	1	1
Cluster1735	Acidobact	Acidobacteria_Gp2			Gp2	0	0	0	0	1	0	0	0	0	0	0	0	1
Cluster1735E	Proteobac	Alphap	Rhodospirillales			0	0	0	0	0	0	0	0	1	0	0	0	1
Cluster1736	Acidobact	Acidobacteria_Gp1				0	0	0	0	0	0	0	0	0	0	1	0	1
Cluster1737	Acidobact	Acidobacteria_Gp1			Edaphobacter	0	0	0	1	0	0	0	0	0	0	0	0	1
Cluster1738C	Acidobact	Acidobacteria_Gp2			Gp2	0	0	0	0	1	0	0	0	0	0	0	0	1
Cluster1738	Acidobacteria					0	0	0	0	0	0	0	0	0	0	1	0	1
Cluster1739	Planctom	Plancto	Planc	Planctor	Singulisphaera	0	0	0	1	0	0	0	0	0	0	0	0	1
Cluster1739E	Acidobact	Acidobacteria_Gp3			Gp3	0	0	0	0	0	0	0	0	0	1	0	0	1
Cluster1740	Acidobacteria					0	0	0	0	0	1	0	0	0	0	0	0	1
Cluster1740	Acidobact	Acidobacteria_Gp3				0	0	0	0	0	0	0	0	0	0	0	1	1
Cluster1740	Acidobact	Acidobacteria_Gp1				0	0	0	0	0	0	0	0	0	0	1	0	1
Cluster1741Z	Proteobacteria					0	0	0	1	0	0	0	0	0	0	0	0	1
Cluster1741E	Proteobac	Alphap	Rhod	Acetobacteraceae		0	0	0	1	0	0	0	0	0	0	0	0	1
Cluster17421	Acidobact	Acidobacteria_Gp1			Gp1	0	0	0	0	0	0	0	0	0	0	1	0	1
Cluster1742	Proteobac	Betaproteobacteria				0	0	0	0	0	1	0	0	0	0	0	0	1
Cluster17431	Acidobact	Acidobacteria_Gp1				0	0	0	0	0	0	0	0	0	1	0	0	1
Cluster1743E	Acidobact	Acidobacteria_Gp3				0	0	0	1	0	0	0	0	0	0	0	0	1
Cluster1744	Proteobac	Alphap	Alphaproteob		Rhizomicrobiu	0	0	0	0	0	0	1	0	0	0	0	0	1
Cluster17464;size=1						0	0	0	0	0	1	0	0	0	0	0	0	1
Cluster1747	Acidobact	Acidobacteria_Gp2			Gp2	0	0	1	0	0	0	0	0	0	0	0	0	1

Cluster17476	Proteobac	Alphap	Rhizobiales			0	0	0	1	0	0	0	0	0	0	0	0	1
Cluster17477	Acidobact	Acidobacteria_Gp5			Gp5	0	0	0	0	0	0	0	0	1	0	0	0	1
Cluster17481	Acidobact	Acidobacteria_Gp2			Gp2	0	0	0	1	0	0	0	0	0	0	0	0	1
Cluster17484;size=2						0	0	0	0	1	0	0	0	0	0	0	0	1
Cluster17495	Proteobac	Gammaproteobacteria				0	0	0	0	0	1	0	0	0	0	0	0	1
Cluster17498	Proteobac	Alphap	Rhodospirillales			0	0	0	0	0	0	0	1	0	0	0	0	1
Cluster17503	Acidobact	Acidobacteria_Gp3				0	0	1	0	0	0	0	0	0	0	0	0	1
Cluster17506	Acidobact	Acidobacteria_Gp2			Gp2	0	0	0	1	0	0	0	0	0	0	0	0	1
Cluster17514	Acidobact	Acidobacteria_Gp3				0	0	0	1	0	0	0	0	0	0	0	0	1
Cluster17521	Acidobact	Acidobacteria_Gp3			Gp3	0	0	0	0	0	0	0	0	0	0	1	0	1
Cluster17538	Proteobac	Deltaproteobacteria				0	0	0	0	0	0	0	0	0	0	0	1	1
Cluster17547	Acidobact	Acidobacteria_Gp2			Gp2	0	0	0	1	0	0	0	0	0	0	0	0	1
Cluster17553	Proteobac	Alphap	Caulo	Cauloba	Phenylobacter	0	0	0	1	0	0	0	0	0	0	0	0	1
Cluster17564	Acidobacteria					0	0	0	0	0	0	0	0	0	0	1	0	1
Cluster17565	Acidobact	Acidobacteria_Gp3			Gp3	0	0	0	0	0	0	0	0	0	0	1	0	1
Cluster17566	Proteobac	Alphap	Caulo	Caulobacteraceae		0	0	0	0	0	0	0	0	0	0	1	0	1
Cluster17569	Proteobac	Alphaproteobacteria				0	0	0	0	0	0	0	1	0	0	0	0	1
Cluster17575	Acidobact	Acidobacteria_Gp10			Gp10	0	0	0	0	0	1	0	0	0	0	0	0	1
Cluster17576	Acidobact	Acidobacteria_Gp1			Gp1	0	0	1	0	0	0	0	0	0	0	0	0	1
Cluster17577;size=1						0	0	0	0	0	0	0	0	0	0	1	0	1
Cluster17585	Proteobac	Alphap	Rhod	Acetobacteraceae		0	0	0	0	0	0	0	0	0	0	1	0	1
Cluster17587	Verrucom	Subdivision3			Subdivision3_g	0	0	0	0	1	0	0	0	0	0	0	0	1
Cluster17590	Proteobacteria					0	0	0	0	0	0	0	0	0	0	1	0	1
Cluster17591	Gemmatir	Gemm	Gemi	Gemma	Gemmatimona	0	0	0	0	1	0	0	0	0	0	0	0	1
Cluster17594	Acidobact	Acidobacteria_Gp1				0	0	0	0	1	0	0	0	0	0	0	0	1
Cluster17608	Acidobacteria					0	0	0	0	0	0	0	0	0	0	0	1	1
Cluster17609	Actinobac	Actinol	Acidi	Acidimi	Aciditerrimona	0	0	0	0	1	0	0	0	0	0	0	0	1
Cluster17619	Acidobacteria					0	0	0	1	0	0	0	0	0	0	0	0	1
Cluster17620	Acidobact	Acidobacteria_Gp1			Gp1	0	0	0	0	1	0	0	0	0	0	0	0	1

Cluster17625	Verrucom	Opitut	Opitu	Opituta	Opitutus	0	0	0	0	0	1	0	0	0	0	0	0	1
Cluster17643	Actinobac	Actinol	Actinomycetales			0	0	0	1	0	0	0	0	0	0	0	0	1
Cluster17660	Acidobact	Acidobacteria_Gp10			Gp10	0	0	0	0	0	0	0	0	0	0	1	0	1
Cluster17664;size=2						0	0	0	0	0	1	0	0	0	0	0	0	1
Cluster17668	Acidobact	Acidobacteria_Gp2			Gp2	0	0	0	1	0	0	0	0	0	0	0	0	1
Cluster17672;size=1						0	0	0	0	0	1	0	0	0	0	0	0	1
Cluster17679	Acidobact	Acidobacteria_Gp1				0	0	0	0	1	0	0	0	0	0	0	0	1
Cluster17683	Acidobact	Acidobacteria_Gp1				0	0	0	0	0	1	0	0	0	0	0	0	1
Cluster17685	Proteobac	Deltap	Myxc	Polyangiaceae		0	0	1	0	0	0	0	0	0	0	0	0	1
Cluster17693	Acidobact	Acidobacteria_Gp1			Granulicella	0	0	0	1	0	0	0	0	0	0	0	0	1
Cluster17699	Acidobact	Acidobacteria_Gp2			Gp2	0	0	0	1	0	0	0	0	0	0	0	0	1
Cluster17702	Proteobacteria					0	0	0	0	0	1	0	0	0	0	0	0	1
Cluster17705	Acidobact	Acidobacteria_Gp1				0	0	1	0	0	0	0	0	0	0	0	0	1
Cluster17709	Verrucom	Opitut	Opitu	Opituta	Opitutus	0	0	0	1	0	0	0	0	0	0	0	0	1
Cluster17714	Armatimonadetes				Armatimonade	0	0	0	1	0	0	0	0	0	0	0	0	1
Cluster17715	Acidobact	Acidobacteria_Gp1				0	0	0	0	0	1	0	0	0	0	0	0	1
Cluster17717	Acidobact	Acidobacteria_Gp2			Gp2	0	1	0	0	0	0	0	0	0	0	0	0	1
Cluster17718	Proteobac	Gammaproteobacteria				0	0	0	0	0	0	0	0	0	0	1	0	1
Cluster17719	Acidobact	Acidobacteria_Gp2			Gp2	0	0	0	0	1	0	0	0	0	0	0	0	1
Cluster17725	Acidobact	Acidobacteria_Gp1				0	0	0	0	0	0	0	1	0	0	0	0	1
Cluster17747	Acidobact	Acidobacteria_Gp2			Gp2	0	0	0	0	1	0	0	0	0	0	0	0	1
Cluster17754	Actinobac	Actinol	Soliru	Conexib	Conexibacter	0	0	0	0	0	0	0	0	0	0	0	1	1
Cluster17755	Acidobact	Acidobacteria_Gp2			Gp2	0	0	0	0	0	0	0	1	0	0	0	0	1
Cluster17757	Chloroflex	Ktedor	Ktedo	Thermo	Thermosporot	0	0	0	0	0	0	0	0	0	0	0	1	1
Cluster17761;size=1						0	0	0	0	0	0	0	0	0	0	1	0	1
Cluster17786	Acidobact	Acidobacteria_Gp2			Gp2	0	0	0	0	0	0	0	1	0	0	0	0	1
Cluster17795	Acidobact	Acidobacteria_Gp2			Gp2	0	0	0	0	0	1	0	0	0	0	0	0	1
Cluster17796	Proteobac	Deltaproteobacteria				0	0	0	0	0	0	0	0	0	0	0	1	1
Cluster17797	Actinobac	Actinol	Actin	Nocardi	Nocardia	0	0	0	0	0	0	0	1	0	0	0	0	1

Cluster17801	Actinobac	Actinol	Actinomycetales			0	0	0	0	0	0	0	1	0	0	0	0	1
Cluster17805	Proteobac	Alphap	Caulc	Caulobacteraceae		0	0	0	0	0	0	0	1	0	0	0	0	1
Cluster17811	Acidobact	Acidobacteria_Gp2			Gp2	0	0	0	0	0	0	0	0	0	0	0	1	1
Cluster17814	Acidobacteria					0	0	0	0	0	0	0	1	0	0	0	0	1
Cluster17829	Proteobac	Alphap	Rhod	Acetobacteraceae		0	0	0	0	0	0	0	1	0	0	0	0	1
Cluster17831	Acidobact	Acidobacteria_Gp1				0	0	0	0	0	0	0	0	0	0	1	0	1
Cluster17832	Proteobac	Betapr	Burkl	Burkhol	Burkholderia	0	0	0	0	0	0	0	1	0	0	0	0	1
Cluster17833	Acidobact	Acidobacteria_Gp2			Gp2	0	0	0	0	0	0	0	1	0	0	0	0	1
Cluster17835	Proteobacteria					0	0	0	0	0	0	0	0	0	0	1	0	1
Cluster17840	Proteobac	Gammaproteobacteria				0	0	0	0	0	0	0	0	0	0	1	0	1
Cluster17841	Acidobact	Acidobacteria_Gp1				0	0	0	0	0	0	1	0	0	0	0	0	1
Cluster17842	Proteobac	Gammaproteobacteria				0	0	0	0	0	0	0	1	0	0	0	0	1
Cluster17847;size=1						0	0	0	0	0	0	0	1	0	0	0	0	1
Cluster17850	Proteobac	Alphap	Rhizc	Beijerinckiaceae		0	0	0	0	0	0	0	0	0	0	0	1	1
Cluster17852	Proteobac	Alphap	Rhizobiales			0	0	0	0	0	0	0	0	1	0	0	0	1
Cluster17868	Acidobact	Acidobacteria_Gp3			Gp3	0	1	0	0	0	0	0	0	0	0	0	0	1
Cluster17873	Acidobact	Acidobacteria_Gp2			Gp2	0	0	0	1	0	0	0	0	0	0	0	0	1
Cluster17874	Acidobact	Acidobacteria_Gp2			Gp2	0	0	0	0	0	0	0	0	0	0	1	0	1
Cluster17876	Proteobac	Alphap	Rhizc	Xanthol	Pseudolabrys	0	0	0	0	0	0	0	0	0	0	1	0	1
Cluster17877	Proteobac	Alphap	Rhizobiales			0	0	0	0	0	0	0	0	0	0	0	1	1
Cluster17883	Acidobact	Acidobacteria_Gp3			Gp3	0	0	0	0	0	0	0	1	0	0	0	0	1
Cluster17886	Proteobac	Gamm	Legio	Legione	Legionella	0	0	0	0	0	1	0	0	0	0	0	0	1
Cluster17892	Proteobac	Alphap	Alphaproteol		Rhizomicrobiu	0	0	0	0	0	0	0	0	0	0	1	0	1
Cluster17893	Acidobact	Acidobacteria_Gp1				0	0	0	0	1	0	0	0	0	0	0	0	1
Cluster17901	Proteobac	Alphap	Rhod	Rhodos	Dongia	0	0	0	0	0	0	0	0	0	0	1	0	1
Cluster17903	Acidobact	Acidobacteria_Gp1				0	0	0	0	0	0	1	0	0	0	0	0	1
Cluster17912;size=1						0	0	0	0	0	0	0	0	0	0	1	0	1
Cluster17913	Actinobac	Actinol	Solirt	Solirubr	Solirubrobacte	0	0	0	0	0	0	0	0	0	0	1	0	1
Cluster17917	Proteobac	Gammaproteobacteria				0	0	0	0	0	0	0	0	0	0	1	0	1

Cluster17919	Proteobac	Alphap	Rhizobiales			0	0	0	0	0	0	0	0	0	0	1	0	1
Cluster17923	Verrucom	Opituta	Opitu	Opituta	Opitutus	0	0	0	0	0	0	0	0	0	1	0	0	1
Cluster17927	Acidobact	Acidobacteria_Gp2			Gp2	0	0	0	0	0	0	0	0	0	0	0	1	1
Cluster17942	Actinobac	Actinol	Soliru	Solirubr	Solirubrobacte	0	0	0	0	0	0	0	0	0	0	0	1	1
Cluster17945	Proteobac	Deltaproteobacteria				0	0	0	0	0	0	0	0	0	0	1	0	1
Cluster17952	Proteobac	Alphaproteobacteria				1	0	0	0	0	0	0	0	0	0	0	0	1
Cluster17954	Acidobact	Acidobacteria_Gp2			Gp2	0	0	0	0	0	0	1	0	0	0	0	0	1
Cluster17962	Proteobac	Betaproteobacteria				0	0	0	0	1	0	0	0	0	0	0	0	1
Cluster17965	Acidobact	Acidobacteria_Gp3			Gp3	1	0	0	0	0	0	0	0	0	0	0	0	1
Cluster17967;size=1						0	0	0	0	0	0	0	1	0	0	0	0	1
Cluster17968	Acidobact	Acidobacteria_Gp2			Gp2	0	0	1	0	0	0	0	0	0	0	0	0	1
Cluster17971	Acidobact	Acidobacteria_Gp2			Gp2	0	0	0	1	0	0	0	0	0	0	0	0	1
Cluster17976	Acidobact	Acidobacteria_Gp1			Gp1	0	0	0	0	0	0	1	0	0	0	0	0	1
Cluster17984	Proteobac	Deltap	Bdell	Bdellovi	Bdellovibrio	0	0	0	0	0	0	0	0	0	0	0	1	1
Cluster17987	Bacteroid	Sphing	Sphingobacteriales			0	0	1	0	0	0	0	0	0	0	0	0	1
Cluster17995	Proteobac	Alphap	Rhizobiales			0	0	1	0	0	0	0	0	0	0	0	0	1
Cluster17998	Armatimo	Armati	Arma	Armatir	Armatimonas/	0	0	0	0	0	0	0	0	0	0	0	1	1
Cluster18004	Actinobac	Actinol	Acidi	Acidimi	Aciditerrimona	0	0	0	1	0	0	0	0	0	0	0	0	1
Cluster18016	Proteobac	Gamm	Xantl	Xanthor	Stenotrophom	0	0	0	0	0	0	0	0	0	0	0	1	1
Cluster18018	Proteobac	Alphap	Rhod	Acetobacteraceae		0	0	0	0	0	0	0	0	0	1	0	0	1
Cluster18024;size=2						0	0	0	0	0	0	0	0	0	0	1	0	1
Cluster18029	Proteobac	Alphap	Rhizo	Hyphon	Rhodoplanes	0	0	0	0	0	0	0	0	0	0	1	0	1
Cluster18035	Proteobac	Betapr	Burkholderiales			0	0	0	0	0	0	0	1	0	0	0	0	1
Cluster18036	Proteobac	Deltaproteobacteria				0	0	0	0	0	0	0	0	0	0	1	0	1
Cluster18039	Acidobact	Acidobacteria_Gp2			Gp2	0	0	0	0	0	0	0	0	0	0	1	0	1
Cluster18044	Chloroflex	Ktedor	Kted	Ktedon	Ktedonobacter	0	0	0	0	0	0	0	0	0	0	1	0	1
Cluster18053	Proteobac	Gamm	Xantl	Sinobacteraceae		0	0	0	0	0	0	0	0	0	0	1	0	1
Cluster18057;size=5						0	0	0	0	0	0	0	0	0	0	1	0	1
Cluster18059	TM7				TM7_genera_i	0	0	0	0	0	0	0	0	0	0	1	0	1

Cluster18060	Proteobac	Deltaproteobacteria				0	0	0	0	0	0	1	0	0	0	0	0	1
Cluster18063	Acidobact	Acidobacteria_Gp1				0	0	0	0	0	0	0	0	1	0	0	0	1
Cluster18069	Proteobac	Alphaproteobacteria				0	0	0	1	0	0	0	0	0	0	0	0	1
Cluster18073	Acidobact	Acidobacteria_Gp1			Gp1	1	0	0	0	0	0	0	0	0	0	0	0	1
Cluster18082	Proteobac	Alphaproteobacteria				0	0	0	0	0	0	0	1	0	0	0	0	1
Cluster18085	Proteobac	Alphap	Rhizc	Methylc	Methylosinus	0	0	0	0	0	0	0	1	0	0	0	0	1
Cluster18097	Acidobact	Acidobacteria_Gp2			Gp2	0	0	0	0	0	0	0	0	1	0	0	0	1
Cluster18121	Cyanobac	Chloroplast		Chlorop	Streptophyta	0	0	0	0	0	0	0	0	0	0	1	0	1
Cluster18125;size=4						0	0	0	0	1	0	0	0	0	0	0	0	1
Cluster18126	Proteobac	Gammaproteobacteria				0	0	0	0	0	0	0	0	0	1	0	0	1
Cluster18128	Actinobac	Actinol	Actin	Actinos	Actinospica	0	1	0	0	0	0	0	0	0	0	0	0	1
Cluster18137	OD1				OD1_genera_i	0	0	0	0	0	0	0	0	0	0	0	1	1
Cluster18138	Proteobac	Gamm	Xantl	Sinobac	Steroidobacter	0	0	0	0	0	0	0	0	0	1	0	0	1
Cluster18139;size=3						0	0	0	0	0	0	0	0	0	0	1	0	1
Cluster18145	Acidobact	Acidobacteria_Gp1				0	0	0	0	0	0	0	0	0	0	1	0	1
Cluster18152	Actinobac	Actinol	Actinomycetales			0	0	0	0	0	0	0	0	0	0	1	0	1
Cluster18161	Proteobac	Gammaproteobacteria				0	0	0	0	0	0	0	0	0	0	0	1	1
Cluster18167	Acidobact	Acidobacteria_Gp3			Gp3	0	0	0	1	0	0	0	0	0	0	0	0	1
Cluster18171	Proteobac	Betapr	Burkl	Burkhol	Ralstonia	0	0	0	0	0	0	0	1	0	0	0	0	1
Cluster18182	Proteobac	Gamm	Xantl	Sinobac	Steroidobacter	0	0	0	0	0	0	0	0	0	1	0	0	1
Cluster18183	Verrucom	Opituta	Opitu	Opituta	Opitutus	0	0	0	0	0	0	0	0	0	0	0	1	1
Cluster18190	TM7				TM7_genera_i	0	1	0	0	0	0	0	0	0	0	0	0	1
Cluster18191	Acidobact	Acidobacteria_Gp3				0	1	0	0	0	0	0	0	0	0	0	0	1
Cluster18206	Proteobac	Gammaproteobacteria				0	0	0	0	0	0	0	0	1	0	0	0	1
Cluster18209	Proteobac	Alphap	Rhizobiales			0	0	0	0	0	0	0	0	0	0	1	0	1
Cluster18212	Acidobact	Acidobacteria_Gp1				0	0	0	0	0	0	0	0	0	0	0	1	1
Cluster18217	Proteobac	Alphap	Caulc	Caulobacteraceae		0	0	1	0	0	0	0	0	0	0	0	0	1
Cluster18219	Proteobac	Alphaproteobacteria				1	0	0	0	0	0	0	0	0	0	0	0	1
Cluster18235	Proteobac	Alphap	Rhod	Acetobacteraceae		0	0	0	0	0	0	0	0	0	0	1	0	1

Cluster18236	TM7				TM7_genera_i	0	0	0	0	0	0	0	1	0	0	0	0	1
Cluster18240	Proteobac	Alphap	Rhod	Acetobacteraceae		0	0	0	0	0	0	0	0	0	0	1	0	1
Cluster18242	Acidobact	Acidobacteria_Gp1			Edaphobacter	1	0	0	0	0	0	0	0	0	0	0	0	1
Cluster18248	Chlamydia	Chlamy	Chlar	Parachlamydiaceae		0	0	0	0	0	0	1	0	0	0	0	0	1
Cluster18252	Proteobac	Alphap	Rhizc	Methylc	Methylocystis	0	0	0	0	0	0	0	0	0	0	1	0	1
Cluster18257;size=1						0	0	0	0	0	0	0	0	0	0	1	0	1
Cluster18258	Proteobac	Alphap	Rhodospirillales			0	0	0	0	0	0	0	1	0	0	0	0	1
Cluster18262	Proteobac	Deltap	Myxc	Polyangiaceae		0	0	0	0	0	0	0	1	0	0	0	0	1
Cluster18268	Acidobact	Acidobacteria_Gp2			Gp2	0	0	0	0	0	0	0	0	0	0	0	1	1
Cluster18270	Acidobact	Acidobacteria_Gp1			Gp1	0	0	0	0	1	0	0	0	0	0	0	0	1
Cluster18274	Actinobac	Actinol	Actinomycetales			0	0	1	0	0	0	0	0	0	0	0	0	1
Cluster18284	Proteobac	Alphap	Rhod	Rhodospirillaceae		0	0	0	0	0	0	0	0	0	0	1	0	1
Cluster18292	Proteobac	Alphaproteobacteria				0	0	0	0	0	0	0	1	0	0	0	0	1
Cluster18297	Acidobact	Acidobacteria_Gp2			Gp2	0	0	0	0	1	0	0	0	0	0	0	0	1
Cluster18298	Acidobact	Acidobacteria_Gp2			Gp2	0	0	0	0	0	0	0	0	0	0	1	0	1
Cluster18300	Acidobact	Acidobacteria_Gp2			Gp2	0	0	0	0	0	0	0	0	1	0	0	0	1
Cluster18307	Proteobac	Deltap	Myxc	Polyangiaceae		0	0	0	0	0	0	0	0	0	0	0	1	1
Cluster18317	Proteobac	Alphaproteobacteria				0	0	1	0	0	0	0	0	0	0	0	0	1
Cluster18319	Actinobac	Actinobacteria				0	0	0	0	0	0	0	0	0	0	1	0	1
Cluster18322	Proteobac	Alphap	Rhod	Acetoba	Acidisphaera	0	0	0	0	0	0	0	0	0	0	1	0	1
Cluster18325	Acidobact	Acidobacteria_Gp1				0	0	0	0	0	0	0	1	0	0	0	0	1
Cluster18328	Acidobact	Acidobacteria_Gp3			Gp3	0	0	0	0	0	0	0	0	0	0	1	0	1
Cluster18329	TM7				TM7_genera_i	0	1	0	0	0	0	0	0	0	0	0	0	1
Cluster18330	Actinobac	Actinol	Actinomycetales			0	0	0	0	0	0	1	0	0	0	0	0	1
Cluster18338	Acidobact	Acidobacteria_Gp2			Gp2	0	0	0	1	0	0	0	0	0	0	0	0	1
Cluster18348	Proteobac	Gammaproteobacteria				0	0	0	0	0	0	0	0	0	0	1	0	1
Cluster18353	Acidobact	Acidobacteria_Gp2			Gp2	0	0	0	0	0	0	0	0	0	1	0	0	1
Cluster18356	Acidobact	Acidobacteria_Gp1				0	0	0	0	0	0	0	0	0	0	0	1	1
Cluster18357	Proteobac	Alphap	Rhizobiales			0	0	0	0	0	0	0	1	0	0	0	0	1

Cluster1835	Armatimo	Chthor	Chth	Chthono	Chthonomona	0	0	0	0	0	0	0	1	0	0	0	0	1
Cluster18361	Proteobac	Betapr	Burkl	Burkhol	Paucimonas	0	0	0	0	0	0	0	0	0	0	0	1	1
Cluster18362	Proteobac	Alphap	Rhod	Acetoba	Acidisphaera	0	0	1	0	0	0	0	0	0	0	0	0	1
Cluster18364	Proteobac	Alphap	Rhodospirillales			0	0	0	1	0	0	0	0	0	0	0	0	1
Cluster1836	Acidobact	Acidobacteria_Gp3			Gp3	0	0	0	0	0	0	0	0	1	0	0	0	1
Cluster18371	Proteobac	Gammaproteobacteria				0	0	0	1	0	0	0	0	0	0	0	0	1
Cluster18373	Proteobac	Alphap	Rhod	Rhodos	Telmatospirillu	0	0	0	0	0	0	0	1	0	0	0	0	1
Cluster18382	Acidobact	Acidobacteria_Gp3			Bryobacter	0	0	0	0	0	0	0	0	0	0	0	1	1
Cluster18385	Chloroflex	Ktedor	Ktedonobacterales			0	0	0	0	0	0	0	0	0	0	1	0	1
Cluster18387	Acidobact	Acidobacteria_Gp1			Acidobacteriur	0	0	0	0	0	0	0	0	0	0	1	0	1
Cluster18388	Acidobact	Acidobacteria_Gp3			Gp3	0	0	0	0	0	0	0	0	0	0	0	1	1
Cluster18389	Acidobact	Acidobacteria_Gp3			Gp3	0	0	0	1	0	0	0	0	0	0	0	0	1
Cluster18395	Bacteroidetes					0	0	0	0	0	0	0	0	0	0	1	0	1
Cluster18397	Proteobac	Alphaproteobacteria				0	0	0	0	0	0	0	1	0	0	0	0	1
Cluster18399	Proteobac	Alphap	Rhod	Acetobacteraceae		0	0	0	0	0	0	0	0	0	0	1	0	1
Cluster18400	Actinobac	Actinol	Actinomycetales			0	0	0	0	0	0	0	0	0	0	1	0	1
Cluster18406	Acidobact	Acidobacteria_Gp2			Gp2	0	0	0	0	0	0	1	0	0	0	0	0	1
Cluster18408	Acidobact	Acidobacteria_Gp2			Gp2	0	0	0	0	0	0	0	0	0	0	0	1	1
Cluster18411	Proteobac	Alphap	Rhodospirillales			0	0	0	0	0	0	0	1	0	0	0	0	1
Cluster18420	Proteobac	Betapr	Rhod	Rhodocyclaceae		0	0	1	0	0	0	0	0	0	0	0	0	1
Cluster18427	Proteobac	Gamm	Xantl	Sinobac	Steroidobacter	1	0	0	0	0	0	0	0	0	0	0	0	1
Cluster18430	Acidobact	Acidobacteria_Gp2			Gp2	0	0	0	0	0	0	0	0	0	1	0	0	1
Cluster18432	Acidobact	Acidobacteria_Gp1				0	0	0	0	0	0	0	0	0	0	1	0	1
Cluster18438	Acidobact	Acidobacteria_Gp2			Gp2	0	0	0	0	1	0	0	0	0	0	0	0	1
Cluster18448	Acidobact	Acidobacteria_Gp2			Gp2	0	0	0	0	0	0	0	0	0	0	0	1	1
Cluster18451	Proteobac	Alphap	Alphaproteot		Rhizomicrobiu	0	0	0	0	0	0	0	0	0	0	1	0	1
Cluster18458	Bacteroid	Sphing	Sphir	Cytopha	Adhaeribacter	0	1	0	0	0	0	0	0	0	0	0	0	1
Cluster18463	Acidobact	Acidobacteria_Gp1			Gp1	0	0	0	0	0	0	0	0	0	0	1	0	1
Cluster18474	Proteobac	Alphaproteobacteria				0	0	0	0	0	0	0	1	0	0	0	0	1

Cluster18478	Acidobact	Acidobacteria_Gp3				0	0	0	1	0	0	0	0	0	0	0	0	1
Cluster18483	Proteobac	Gamm	Xantl	Sinobac	Steroidobacter	0	0	0	0	0	0	0	1	0	0	0	0	1
Cluster18495	Acidobact	Acidobacteria_Gp2			Gp2	0	0	0	0	0	0	0	0	0	0	1	0	1
Cluster18498	Proteobac	Gammaproteobacteria				0	0	0	0	0	0	0	0	0	0	1	0	1
Cluster18499	Acidobact	Acidobacteria_Gp2			Gp2	0	0	0	0	0	0	0	0	1	0	0	0	1
Cluster18502;size=1						0	0	0	0	0	0	0	0	0	0	1	0	1
Cluster18503	Proteobac	Alphap	Rhodospirillales			0	0	0	0	0	0	0	0	0	0	0	1	1
Cluster18507	Acidobact	Acidobacteria_Gp2			Gp2	0	1	0	0	0	0	0	0	0	0	0	0	1
Cluster18518	Verrucom	Subdivision3			Subdivision3_g	0	0	0	0	0	1	0	0	0	0	0	0	1
Cluster18519	Acidobact	Acidobacteria_Gp1				0	0	0	0	0	0	0	0	1	0	0	0	1
Cluster18521	Acidobact	Acidobacteria_Gp1				0	0	0	0	0	0	0	0	0	0	1	0	1
Cluster18525	Acidobact	Acidobacteria_Gp2			Gp2	0	0	0	0	0	0	0	0	1	0	0	0	1
Cluster18528	Proteobac	Betaproteobacteria				0	0	0	0	0	0	0	0	0	0	0	1	1
Cluster18541	Acidobact	Acidobacteria_Gp2			Gp2	0	0	0	0	0	0	0	1	0	0	0	0	1
Cluster18543	Acidobact	Acidobacteria_Gp13			Gp13	0	0	0	0	0	0	0	0	1	0	0	0	1
Cluster18545;size=1						0	0	0	0	0	0	0	0	0	0	0	1	1
Cluster18548	Acidobact	Acidobacteria_Gp2			Gp2	0	0	0	0	0	0	0	0	1	0	0	0	1
Cluster18552	Acidobact	Acidobacteria_Gp2			Gp2	1	0	0	0	0	0	0	0	0	0	0	0	1
Cluster18556	Chlamydia	Chlamy	Chlar	Parachlamydiaceae		0	0	0	0	0	0	1	0	0	0	0	0	1
Cluster18560	Acidobact	Acidobacteria_Gp3				0	0	0	0	0	0	0	0	0	0	0	1	1
Cluster18562	Acidobact	Acidobacteria_Gp2			Gp2	0	0	0	1	0	0	0	0	0	0	0	0	1
Cluster18571	Proteobac	Deltap	Bdell	Bdellovi	Bdellovibrio	0	0	0	0	0	0	0	0	1	0	0	0	1
Cluster18574	Proteobac	Gamm	Xantl	Sinobac	Steroidobacter	0	0	0	0	0	0	0	0	0	0	0	1	1
Cluster18575	Acidobact	Acidobacteria_Gp3			Gp3	0	0	0	0	0	0	0	0	0	0	0	1	1
Cluster18578	Chloroflex	Ktedor	Ktedonobacterales			0	0	0	0	0	1	0	0	0	0	0	0	1
Cluster18581	Proteobac	Alphap	Caulc	Caulobacteraceae		0	0	0	0	0	0	0	0	1	0	0	0	1
Cluster18590	Proteobac	Alphaproteobacteria				0	0	0	0	0	0	0	0	1	0	0	0	1
Cluster18592	Acidobact	Acidobacteria_Gp2			Gp2	0	0	0	0	0	0	0	0	1	0	0	0	1
Cluster18594	Proteobacteria					0	0	0	0	0	0	0	0	1	0	0	0	1

Cluster18609	Acidobact	Acidobacteria_Gp2			Gp2	0	0	0	0	0	0	0	0	0	0	0	1	1
Cluster18612;size=1						0	0	0	0	0	0	0	0	1	0	0	0	1
Cluster18620;size=1						0	0	0	0	0	0	0	0	0	0	0	1	1
Cluster18630	Acidobact	Acidobacteria_Gp3			Gp3	0	0	0	0	0	0	0	1	0	0	0	0	1
Cluster18634	Proteobac	Alphap	Rhizc	Bradyrh	Bosea	0	0	0	0	0	0	0	0	0	0	0	1	1
Cluster18653	Actinobac	Actinol	Solirt	Solirubr	Solirubrobacte	0	0	0	0	0	0	1	0	0	0	0	0	1
Cluster18655	Acidobacteria					0	0	0	0	0	0	0	0	0	0	0	1	1
Cluster18669	Actinobac	Actinol	Actin	Actinos	Actinospica	0	0	0	0	0	0	0	1	0	0	0	0	1
Cluster18676	Acidobact	Acidobacteria_Gp2			Gp2	0	0	0	0	0	0	1	0	0	0	0	0	1
Cluster18691	Acidobact	Acidobacteria_Gp3			Gp3	1	0	0	0	0	0	0	0	0	0	0	0	1
Cluster18704	Proteobac	Deltap	Myxococcales			0	0	0	0	0	0	0	0	0	0	0	1	1
Cluster18705;size=1						0	0	0	0	0	0	0	0	0	0	1	0	1
Cluster18707	Acidobact	Acidobacteria_Gp2			Gp2	0	0	1	0	0	0	0	0	0	0	0	0	1
Cluster18710	Actinobac	Actinol	Acidi	Acidimi	Aciditerrimona	0	0	0	0	0	0	0	0	0	0	0	1	1
Cluster18716	Proteobac	Gamm	Legio	Coxiella	Aquicella	0	0	0	0	0	0	0	0	1	0	0	0	1
Cluster18726	Proteobac	Alphap	Rhodospirillales			0	0	0	0	0	0	0	0	0	0	0	1	1
Cluster18736	Acidobact	Acidobacteria_Gp3			Gp3	0	0	0	0	0	0	0	0	1	0	0	0	1
Cluster18743	Acidobact	Acidobacteria_Gp2			Gp2	0	0	0	0	0	0	0	0	0	0	0	1	1
Cluster18759;size=4						0	0	0	0	0	0	0	0	1	0	0	0	1
Cluster18777	Proteobac	Deltaproteobacteria				0	0	0	0	0	0	0	0	0	0	0	1	1
Cluster18778;size=1						0	0	0	0	0	0	0	0	0	0	0	1	1
Cluster18783	Proteobac	Alphaproteobacteria				0	1	0	0	0	0	0	0	0	0	0	0	1
Cluster18784	Acidobact	Acidobacteria_Gp1			Gp1	0	0	0	0	0	1	0	0	0	0	0	0	1
Cluster18790	Proteobac	Gamm	Legio	Coxiella	Aquicella	0	0	0	1	0	0	0	0	0	0	0	0	1
Cluster18791	Armatimc	Armati	Arma	Armatir	Armatimonas/	0	0	0	0	0	0	0	0	1	0	0	0	1
Cluster18805	Acidobact	Acidobacteria_Gp3				1	0	0	0	0	0	0	0	0	0	0	0	1
Cluster18815;size=1						0	0	0	0	0	0	0	0	0	0	0	1	1
Cluster18817;size=1						0	0	0	0	0	0	0	0	0	0	0	1	1
Cluster18823;size=1						0	0	0	0	0	0	0	0	1	0	0	0	1

Cluster1882€	Acidobact	Acidobacteria_Gp3			Gp3	0	0	0	0	0	0	0	0	0	0	0	1	1
Cluster18831	Acidobact	Acidobacteria_Gp2			Gp2	0	0	0	0	0	0	0	0	0	1	0	0	1
Cluster18843;size=2						0	0	0	0	0	0	0	0	0	0	0	1	1
Cluster18851	Acidobact	Acidobacteria_Gp3				0	0	0	0	0	0	0	0	1	0	0	0	1
Cluster18856;size=3						0	0	0	0	0	0	0	0	0	0	0	1	1
Cluster18864	Proteobac	Gamm	Legio	Coxiella	Aquicella	0	0	0	1	0	0	0	0	0	0	0	0	1
Cluster1886{	Verrucom	Opituta	Opitu	Opituta	Opitutus	0	0	0	0	0	0	0	0	0	0	0	1	1
Cluster1886{	Acidobact	Acidobacteria_Gp2			Gp2	0	0	0	0	0	0	0	0	0	0	1	0	1
Cluster18871	Proteobac	Deltap	Bdell	Bdellovi	Bdellovibrio	0	0	0	0	0	0	0	0	1	0	0	0	1
Cluster1887€	Acidobact	Acidobacteria_Gp3			Gp3	0	0	0	0	0	0	0	0	1	0	0	0	1
Cluster1887	Acidobact	Acidobacteria_Gp1				0	0	0	0	0	0	0	0	1	0	0	0	1
Cluster18881	Proteobac	Betapr	Burkholderiales			0	0	0	0	0	0	1	0	0	0	0	0	1
Cluster1888	Acidobact	Acidobacteria_Gp2			Gp2	0	0	0	0	0	0	0	0	0	0	0	1	1
Cluster18891	Acidobact	Acidobacteria_Gp2			Gp2	0	0	0	0	0	0	1	0	0	0	0	0	1
Cluster18893;size=12						0	0	0	0	0	1	0	0	0	0	0	0	1
Cluster18894	Gemmatir	Gemm	Gemı	Gemma	Gemmatimona	0	0	0	0	0	0	0	0	0	0	0	1	1
Cluster1889{	Chloroflex	Ktedor	Ktedo	Ktedonc	Ktedonobacter	0	0	0	0	0	0	0	0	0	0	0	1	1
Cluster18899;size=1						0	0	0	0	0	0	0	0	1	0	0	0	1
Cluster18901	Acidobact	Acidobacteria_Gp2			Gp2	0	0	0	0	0	0	0	0	0	0	1	0	1
Cluster18904	Acidobact	Acidobacteria_Gp3			Gp3	0	0	0	0	0	0	0	0	0	1	0	0	1
Cluster1890{	Acidobact	Acidobacteria_Gp2			Gp2	0	0	0	0	0	0	0	0	0	1	0	0	1
Cluster1891(	Acidobact	Acidobacteria_Gp5			Gp5	0	0	0	0	0	0	0	0	0	1	0	0	1
Cluster18911	Actinobac	Actinol	Actinomycetales			0	0	0	0	0	0	0	0	0	1	0	0	1
Cluster18915;size=5						0	0	0	0	0	0	0	1	0	0	0	0	1
Cluster1891{	Proteobac	Alphap	Rhod	Acetoba	Acidisphaera	0	0	0	0	0	0	0	0	0	1	0	0	1
Cluster1892(	Proteobac	Alphap	Rhodospirillales			0	0	0	0	0	0	0	1	0	0	0	0	1
Cluster18921	Acidobact	Acidobacteria_Gp3				0	0	0	0	1	0	0	0	0	0	0	0	1
Cluster1892	Acidobact	Acidobacteria_Gp3			Gp3	0	0	0	0	0	0	0	0	0	0	1	0	1
Cluster1892	Acidobact	Acidobacteria_Gp1				0	0	0	0	0	0	0	0	0	1	0	0	1

Cluster18932	Verrucom	Subdivision3			Subdivision3_g	0	0	0	0	0	0	0	0	0	1	0	0	1
Cluster18933	Proteobac	Alphap	Rhod	Acetobacteraceae		0	0	0	0	0	0	0	0	0	1	0	0	1
Cluster18937	Firmicutes	Bacilli	Bacill	Alicyclobacillaceae		0	0	0	0	0	0	0	0	0	0	0	1	1
Cluster18939	Proteobac	Alphap	Rhizobiales			0	0	0	0	0	0	0	0	0	1	0	0	1
Cluster18944	Armatimonadetes				Armatimonade	0	0	0	0	0	0	0	0	0	1	0	0	1
Cluster18947	Proteobacteria					0	0	0	0	0	0	0	0	1	0	0	0	1
Cluster18951	Acidobact	Acidobacteria_Gp2			Gp2	0	0	0	0	0	0	0	1	0	0	0	0	1
Cluster18953	Proteobac	Alphaproteobacteria				1	0	0	0	0	0	0	0	0	0	0	0	1
Cluster18967;size=3						0	0	0	0	0	1	0	0	0	0	0	0	1
Cluster18975	Proteobac	Betapr	Rhod	Rhodocyclaceae		0	0	0	0	1	0	0	0	0	0	0	0	1
Cluster18976	Bacteroid	Sphing	Sphir	Chitinophagaceae		0	0	0	0	0	0	0	0	0	0	0	1	1
Cluster18984	Bacteroid	Sphing	Sphir	Chitinophagaceae		0	0	1	0	0	0	0	0	0	0	0	0	1
Cluster18986	Proteobac	Betapr	Burkl	Burkhol	Burkholderia	0	0	0	0	0	0	0	0	0	1	0	0	1
Cluster18989	Proteobac	Gamm	Pseu	Pseudomonadaceae		0	0	0	0	0	0	0	0	1	0	0	0	1
Cluster18994	Acidobact	Acidobacteria_Gp2			Gp2	0	0	0	0	0	1	0	0	0	0	0	0	1
Cluster18998	Proteobac	Alphaproteobacteria				0	0	1	0	0	0	0	0	0	0	0	0	1
Cluster18999;size=3						0	0	0	0	0	0	0	1	0	0	0	0	1
Cluster19003	Acidobact	Acidobacteria_Gp1				0	0	0	0	0	0	0	0	0	1	0	0	1
Cluster19010	TM7				TM7_genera_i	0	0	1	0	0	0	0	0	0	0	0	0	1
Cluster19011	Gemmatir	Gemm	Gemi	Gemma	Gemmatimona	0	0	0	0	0	0	0	0	0	0	1	0	1
Cluster19012	Acidobacteria					0	0	0	0	0	0	0	0	0	0	1	0	1
Cluster19014;size=2						0	0	0	0	0	0	0	0	0	0	0	1	1
Cluster19017;size=7						0	0	0	0	0	0	0	1	0	0	0	0	1
Cluster19019	Proteobac	Deltaproteobacteria				0	0	0	0	0	0	0	0	0	1	0	0	1
Cluster19028	Actinobac	Actinol	Actinomycetales			0	0	0	0	0	0	0	0	0	0	0	1	1
Cluster19032;size=1						0	0	0	0	0	0	0	1	0	0	0	0	1
Cluster19038	Acidobact	Acidobacteria_Gp3			Gp3	0	0	0	0	0	1	0	0	0	0	0	0	1
Cluster19039	Proteobac	Alphaproteobacteria				0	0	0	0	0	0	0	0	0	0	1	0	1
Cluster19043	Proteobac	Alphap	Rhizobiales			0	0	0	0	0	0	0	0	0	0	1	0	1

Cluster19052	Acidobact	Acidobacteria_Gp2			Gp2	0	0	0	0	0	0	0	1	0	0	0	0	1
Cluster19054	Acidobact	Acidobacteria_Gp2			Gp2	0	0	0	0	0	1	0	0	0	0	0	0	1
Cluster19072	Proteobac	Alphap	Rhizobiales			0	0	0	0	0	0	0	1	0	0	0	0	1
Cluster19084	Proteobac	Gammaproteobacteria				0	0	0	0	0	0	0	0	0	0	1	0	1
Cluster19088	Armatimo	Chthor	Chth	Chthono	Chthonomona	0	0	0	0	0	0	0	0	0	0	1	0	1
Cluster19089	Acidobact	Acidobacteria_Gp3			Gp3	0	0	0	0	0	0	0	0	0	0	1	0	1
Cluster19099	Proteobac	Alphap	Rhod	Acetobacteraceae		0	0	0	1	0	0	0	0	0	0	0	0	1
Cluster19101;size=7						0	0	0	1	0	0	0	0	0	0	0	0	1
Cluster19102	Proteobac	Deltaproteobacteria				0	0	0	0	0	0	0	0	0	1	0	0	1
Cluster19103	Verrucom	Opituta	Opitu	Opituta	Opitutus	0	0	0	0	0	0	0	0	0	1	0	0	1
Cluster19110	Proteobac	Deltaproteobacteria				0	0	0	0	0	0	0	0	0	1	0	0	1
Cluster19117	Proteobac	Gammaproteobacteria				0	0	0	0	0	0	0	1	0	0	0	0	1
Cluster19129	Acidobact	Acidobacteria_Gp1			Gp1	0	0	0	0	0	0	0	0	0	1	0	0	1
Cluster19139	Acidobact	Acidobacteria_Gp2			Gp2	0	0	1	0	0	0	0	0	0	0	0	0	1
Cluster19145	Acidobact	Acidobacteria_Gp1				0	0	0	1	0	0	0	0	0	0	0	0	1
Cluster19150	Proteobac	Gamm	Xanth	Sinobac	Steroidobacter	0	0	0	0	0	0	0	0	0	0	1	0	1
Cluster19151	Proteobac	Alphaproteobacteria				0	0	0	0	0	0	0	0	0	1	0	0	1
Cluster19152	Proteobac	Alphap	Caulo	Caulobac	Caulobacter	0	0	0	0	0	0	0	0	0	1	0	0	1
Cluster19155	Proteobac	Alphap	Rhizobiales			0	0	0	0	0	0	0	1	0	0	0	0	1
Cluster19156	Proteobacteria					0	0	0	0	0	0	0	0	0	1	0	0	1
Cluster19166	Acidobact	Acidobacteria_Gp2			Gp2	0	0	0	0	0	0	0	1	0	0	0	0	1
Cluster19168	Acidobact	Acidobacteria_Gp3			Gp3	0	0	0	0	0	0	0	0	0	0	0	1	1
Cluster19169;size=1						0	0	0	0	0	0	0	0	1	0	0	0	1
Cluster19170	Proteobac	Gammaproteobacteria				0	0	1	0	0	0	0	0	0	0	0	0	1
Cluster19173	Proteobac	Gamm	Xanth	Sinobac	Steroidobacter	0	0	0	0	0	0	0	0	0	0	0	1	1
Cluster19176	Acidobact	Acidobacteria_Gp2			Gp2	0	0	0	1	0	0	0	0	0	0	0	0	1
Cluster19177	Proteobac	Gammaproteobacteria				0	1	0	0	0	0	0	0	0	0	0	0	1
Cluster19179	Proteobac	Gamm	Xanth	Sinobac	Steroidobacter	0	0	0	0	0	0	0	0	0	0	0	1	1
Cluster19190	Acidobact	Acidobacteria_Gp1				0	0	0	0	0	0	0	1	0	0	0	0	1

Cluster19197	Bacteroid	Sphing	Sphir	Chitinophagaceae		0	0	0	0	0	0	0	0	0	0	0	1	1
Cluster19199	Acidobact	Acidobacteria_Gp3				0	0	0	0	0	0	0	1	0	0	0	0	1
Cluster19203	Chlamydia	Chlamy	Chlamydiales			0	0	0	0	0	0	0	0	1	0	0	0	1
Cluster19210	Acidobact	Acidobacteria_Gp2			Gp2	0	0	0	0	0	0	0	0	1	0	0	0	1
Cluster19215	Bacteroid	Sphing	Sphir	Sphingo	Mucilaginibact	0	0	0	0	0	0	0	0	1	0	0	0	1
Cluster19224	Proteobac	Gamm	Legio	Coxiella	Aquicella	0	0	0	0	1	0	0	0	0	0	0	0	1
Cluster19239	Acidobact	Acidobacteria_Gp3			Gp3	0	0	1	0	0	0	0	0	0	0	0	0	1
Cluster19243;size=2						0	0	0	0	0	1	0	0	0	0	0	0	1
Cluster19267	Proteobac	Gammaproteobacteria				0	0	0	0	0	0	0	1	0	0	0	0	1
Cluster19271	Acidobact	Acidobacteria_Gp1				0	0	0	0	0	0	0	0	1	0	0	0	1
Cluster19281	Verrucom	Opituta	Opitu	Opituta	Opitutus	0	0	0	0	0	0	0	0	0	0	0	1	1
Cluster19283	Acidobact	Acidobacteria_Gp2			Gp2	0	0	0	0	0	0	0	1	0	0	0	0	1
Cluster19285	Verrucom	Opituta	Opitu	Opituta	Opitutus	0	0	0	0	0	0	0	0	0	0	0	1	1
Cluster19286	Acidobact	Acidobacteria_Gp2			Gp2	0	0	1	0	0	0	0	0	0	0	0	0	1
Cluster19288	Proteobac	Alphap	Alphaproteob		Rhizomicrobiu	0	0	0	0	0	0	0	0	0	0	1	0	1
Cluster19289	Acidobact	Acidobacteria_Gp2			Gp2	0	0	0	1	0	0	0	0	0	0	0	0	1
Cluster19290	Acidobact	Acidobacteria_Gp2			Gp2	0	1	0	0	0	0	0	0	0	0	0	0	1
Cluster19298	Acidobact	Acidobacteria_Gp1			Gp1	0	0	0	0	0	0	0	1	0	0	0	0	1
Cluster19302	Acidobact	Acidobacteria_Gp1			Gp1	0	0	0	0	0	0	0	0	1	0	0	0	1
Cluster19303	Proteobac	Gamm	Xantl	Xanthor	Rudaea	0	0	0	0	0	1	0	0	0	0	0	0	1
Cluster19308	Proteobac	Gammaproteobacteria				0	0	0	0	0	0	0	0	0	0	1	0	1
Cluster19313	Acidobact	Acidobacteria_Gp3				0	0	0	0	0	0	0	1	0	0	0	0	1
Cluster19324	Proteobacteria					0	0	0	0	1	0	0	0	0	0	0	0	1
Cluster19349	Acidobact	Acidobacteria_Gp2			Gp2	0	0	0	0	0	0	1	0	0	0	0	0	1
Cluster19353	Proteobac	Gammaproteobacteria				0	0	0	0	0	0	0	0	0	0	1	0	1
Cluster19369	Proteobac	Alphap	Rhizo	Methylo	Methylosinus	1	0	0	0	0	0	0	0	0	0	0	0	1
Cluster19370	Actinobac	Actinobacteria				0	0	0	0	0	1	0	0	0	0	0	0	1
Cluster19374	Acidobact	Acidobacteria_Gp1				0	0	0	1	0	0	0	0	0	0	0	0	1
Cluster19377	Chlamydia	Chlamy	Chlamydiales			1	0	0	0	0	0	0	0	0	0	0	0	1

Cluster19378	Acidobact	Acidobacteria_Gp3				0	0	0	0	0	0	0	0	0	0	0	1	1
Cluster19382	Proteobac	Alphap	Rhod	Acetobacteraceae		0	0	1	0	0	0	0	0	0	0	0	0	1
Cluster19383;size=4						0	0	0	0	1	0	0	0	0	0	0	0	1
Cluster19386	Acidobact	Acidobacteria_Gp2			Gp2	0	0	0	1	0	0	0	0	0	0	0	0	1
Cluster19387	Proteobac	Gammaproteobacteria				0	0	0	1	0	0	0	0	0	0	0	0	1
Cluster19389	Planctom	Plancto	Planc	Planctomycetaceae		0	0	0	1	0	0	0	0	0	0	0	0	1
Cluster19394	Acidobact	Acidobacteria_Gp1				0	0	0	1	0	0	0	0	0	0	0	0	1
Cluster19399;size=2						0	0	0	1	0	0	0	0	0	0	0	0	1
Cluster19408	Acidobact	Acidobacteria_Gp2			Gp2	1	0	0	0	0	0	0	0	0	0	0	0	1
Cluster19410	Proteobac	Alphap	Rhizobiales			0	0	0	1	0	0	0	0	0	0	0	0	1
Cluster19411	Proteobac	Alphap	Rhizo	Bradyrh	Agromonas	0	0	1	0	0	0	0	0	0	0	0	0	1
Cluster19412	Actinobac	Actino	Soliru	Solirubr	Solirubrobacte	0	0	1	0	0	0	0	0	0	0	0	0	1
Cluster19419	Proteobacteria					0	0	1	0	0	0	0	0	0	0	0	0	1
Cluster19422	Proteobacteria					0	0	1	0	0	0	0	0	0	0	0	0	1
Cluster19425	Acidobact	Acidobacteria_Gp2			Gp2	0	0	0	0	0	0	1	0	0	0	0	0	1
Cluster19426	Proteobac	Gammaproteobacteria				1	0	0	0	0	0	0	0	0	0	0	0	1
Cluster19431;size=1						0	0	0	0	0	0	1	0	0	0	0	0	1
Cluster19434	Proteobac	Betaproteobacteria				0	0	0	0	0	1	0	0	0	0	0	0	1
Cluster19442;size=3						1	0	0	0	0	0	0	0	0	0	0	0	1
Cluster19452	Acidobact	Acidobacteria_Gp1				0	0	0	0	0	0	0	0	0	1	0	0	1
Cluster19455	Acidobact	Acidobacteria_Gp1			Gp1	0	0	1	0	0	0	0	0	0	0	0	0	1
Cluster19458	Proteobac	Alphap	Rhod	Acetoba	Acidisphaera	0	0	0	1	0	0	0	0	0	0	0	0	1
Cluster19482	Acidobact	Acidobacteria_Gp1			Gp1	0	0	0	0	0	0	0	0	0	0	0	1	1
Cluster19483	Acidobact	Acidobacteria_Gp2			Gp2	0	0	0	0	1	0	0	0	0	0	0	0	1
Cluster19491	OD1				OD1_genera_i	0	0	0	1	0	0	0	0	0	0	0	0	1
Cluster19494	Acidobact	Acidobacteria_Gp3			Gp3	0	0	1	0	0	0	0	0	0	0	0	0	1
Cluster19507	Acidobact	Acidobacteria_Gp2			Gp2	0	0	0	1	0	0	0	0	0	0	0	0	1
Cluster19517	Acidobact	Acidobacteria_Gp2			Gp2	0	0	0	1	0	0	0	0	0	0	0	0	1
Cluster19519	Proteobacteria					0	0	0	0	0	1	0	0	0	0	0	0	1

Cluster19533	Proteobac	Gamm	Xantl	Sinobac	Steroidobacter	0	0	1	0	0	0	0	0	0	0	0	0	1
Cluster19536	Armatimonadetes				Armatimonade	0	0	0	0	0	0	1	0	0	0	0	0	1
Cluster19547	Proteobac	Gamm	Legio	Coxiella	Coxiella	0	0	0	0	0	0	1	0	0	0	0	0	1
Cluster19549	Acidobact	Acidobacteria_Gp1				0	0	0	0	0	0	1	0	0	0	0	0	1
Cluster19577	Proteobac	Alphap	Alphaproteob		Rhizomicrobiu	0	0	1	0	0	0	0	0	0	0	0	0	1
Cluster19581	Verrucom	Opituta	Opitu	Opituta	Opitutus	0	0	0	0	0	0	0	0	0	0	0	1	1
Cluster19585	Proteobac	Alphap	Rhod	Acetobacteraceae		0	0	1	0	0	0	0	0	0	0	0	0	1
Cluster19589	Proteobac	Gamm	Xantl	Sinobac	Steroidobacter	0	0	0	1	0	0	0	0	0	0	0	0	1
Cluster19590	Proteobac	Betapr	Burkl	Oxaloba	Herbaspirillum	0	0	1	0	0	0	0	0	0	0	0	0	1
Cluster19592	Proteobac	Alphap	Caulo	Caulobacteraceae		0	0	1	0	0	0	0	0	0	0	0	0	1
Cluster19611	Acidobact	Acidobacteria_Gp2			Gp2	0	0	0	0	0	1	0	0	0	0	0	0	1
Cluster19622	Proteobac	Betapr	Burkl	Oxalobacteraceae		0	0	1	0	0	0	0	0	0	0	0	0	1
Cluster19641	Acidobact	Acidobacteria_Gp3			Gp3	0	0	0	1	0	0	0	0	0	0	0	0	1
Cluster19650	Acidobact	Acidobacteria_Gp2			Gp2	0	0	0	0	0	1	0	0	0	0	0	0	1
Cluster19667	Proteobac	Alphap	Alphaproteob		Rhizomicrobiu	0	0	0	1	0	0	0	0	0	0	0	0	1
Cluster19678	Proteobacteria					0	0	1	0	0	0	0	0	0	0	0	0	1
Cluster19699	Proteobac	Gamm	Xantl	Sinobacteraceae		0	0	0	0	0	1	0	0	0	0	0	0	1
Cluster19703	Proteobac	Alphap	Rhizo	Bradyrh	Agromonas	0	0	0	1	0	0	0	0	0	0	0	0	1
Cluster19707	Proteobac	Alphap	Rhod	Acetobacteraceae		0	0	1	0	0	0	0	0	0	0	0	0	1
Cluster19720	Proteobac	Betaproteobacteria				0	0	0	1	0	0	0	0	0	0	0	0	1
Cluster19723	Proteobac	Deltap	Bdell	Bdellovi	Bdellovibrio	0	0	0	0	0	0	1	0	0	0	0	0	1
Cluster19725	Proteobac	Alphap	Rhod	Acetobacteraceae		0	0	1	0	0	0	0	0	0	0	0	0	1
Cluster19733;size=2						0	0	0	0	0	0	1	0	0	0	0	0	1
Cluster19734	Acidobact	Acidobacteria_Gp2			Gp2	0	0	0	1	0	0	0	0	0	0	0	0	1
Cluster19739	Proteobac	Alphap	Caulo	Caulob	Phenylobacter	0	0	1	0	0	0	0	0	0	0	0	0	1
Cluster19761	Proteobac	Gamm	Xantl	Xanthomonadaceae		0	0	0	0	1	0	0	0	0	0	0	0	1
Cluster19763	Acidobact	Acidobacteria_Gp2			Gp2	0	0	0	1	0	0	0	0	0	0	0	0	1
Cluster19769;size=1						0	0	1	0	0	0	0	0	0	0	0	0	1
Cluster19770;size=5						0	0	1	0	0	0	0	0	0	0	0	0	1

Cluster19774	Acidobact	Acidobacteria_Gp1				0	0	0	1	0	0	0	0	0	0	0	0	1
Cluster19781	Acidobacteria					0	0	0	1	0	0	0	0	0	0	0	0	1
Cluster19785	Verrucom	Opituta	Opitu	Opituta	Opitutus	0	0	0	0	1	0	0	0	0	0	0	0	1
Cluster19787	Gemmatir	Gemm	Gem	Gemma	Gemmatimona	0	0	1	0	0	0	0	0	0	0	0	0	1
Cluster19788	Proteobac	Alphap	Sphir	Sphingo	Novosphingob	0	0	0	0	0	0	1	0	0	0	0	0	1
Cluster19794	Proteobac	Gamm	Legio	Coxiella	Aquicella	0	0	0	0	1	0	0	0	0	0	0	0	1
Cluster19799	Proteobac	Alphap	Rhizobiales			1	0	0	0	0	0	0	0	0	0	0	0	1
Cluster19800	Acidobact	Acidobacteria_Gp3				0	0	0	0	1	0	0	0	0	0	0	0	1
Cluster19803	Acidobact	Acidobacteria_Gp1			Gp1	0	0	1	0	0	0	0	0	0	0	0	0	1
Cluster19804	Actinobac	Actinol	Actinomycetales			0	0	0	1	0	0	0	0	0	0	0	0	1
Cluster19813	Actinobac	Actinol	Solirt	Conexib	Conexibacter	0	0	0	0	1	0	0	0	0	0	0	0	1
Cluster19817	Acidobact	Acidobacteria_Gp2			Gp2	0	0	1	0	0	0	0	0	0	0	0	0	1
Cluster19820	Verrucom	Opituta	Opitu	Opituta	Opitutus	0	0	0	0	1	0	0	0	0	0	0	0	1
Cluster19825	Proteobac	Gamm	Enter	Enterobacteriaceae		0	0	1	0	0	0	0	0	0	0	0	0	1
Cluster19834	Actinobac	Actinol	Solirt	Conexib	Conexibacter	0	0	0	0	1	0	0	0	0	0	0	0	1
Cluster19843	Proteobacteria					0	0	0	0	1	0	0	0	0	0	0	0	1
Cluster19845	Bacteroid	Sphing	Sphir	Chitinophagaceae		0	0	1	0	0	0	0	0	0	0	0	0	1
Cluster19852	Proteobac	Alphap	Rhizc	Bradyrhizobiaceae		0	0	0	0	1	0	0	0	0	0	0	0	1
Cluster19882	Proteobac	Alphap	Alphaproteol		Rhizomicrobiu	0	0	0	0	1	0	0	0	0	0	0	0	1
Cluster19884	Proteobac	Alphap	Rhod	Acetoba	Rhodopila	0	0	0	0	1	0	0	0	0	0	0	0	1
Cluster19886	Acidobacteria					0	0	0	0	1	0	0	0	0	0	0	0	1
Cluster19891	Proteobac	Gamm	Xanthomonadales			0	0	1	0	0	0	0	0	0	0	0	0	1
Cluster19892	Proteobac	Betapr	Burkl	Burkhol	Burkholderia	0	0	0	0	1	0	0	0	0	0	0	0	1
Cluster19895	Acidobact	Acidobacteria_Gp3			Gp3	0	0	0	0	1	0	0	0	0	0	0	0	1
Cluster19898	Proteobac	Alphap	Rhod	Acetobacteraceae		0	0	1	0	0	0	0	0	0	0	0	0	1
Cluster19900	Acidobact	Acidobacteria_Gp1				0	0	0	1	0	0	0	0	0	0	0	0	1
Cluster19901	TM7				TM7_genera_i	0	0	0	1	0	0	0	0	0	0	0	0	1
Cluster19902	Acidobact	Acidobacteria_Gp2			Gp2	0	0	0	0	1	0	0	0	0	0	0	0	1
Cluster19903;size=1						0	0	0	1	0	0	0	0	0	0	0	0	1

Cluster19911	Proteobac	Gammaproteobacteria				0	0	0	0	0	0	1	0	0	0	0	0	1
Cluster19922	Acidobact	Acidobacteria_Gp2			Gp2	0	0	0	1	0	0	0	0	0	0	0	0	1
Cluster19928	Proteobac	Alphap	Rhod	Acetobacteraceae		0	0	1	0	0	0	0	0	0	0	0	0	1
Cluster19930	Proteobac	Alphap	Rhizo	Xanthol	Pseudolabrys	0	0	0	0	1	0	0	0	0	0	0	0	1
Cluster19936	Acidobact	Acidobacteria_Gp3				0	0	1	0	0	0	0	0	0	0	0	0	1
Cluster19937	Proteobac	Alphap	Rhizobiales			0	0	0	1	0	0	0	0	0	0	0	0	1
Cluster19938	Acidobact	Acidobacteria_Gp1			Gp1	0	0	1	0	0	0	0	0	0	0	0	0	1
Cluster19942;size=1						0	0	1	0	0	0	0	0	0	0	0	0	1
Cluster19954	Proteobac	Alphap	Rhod	Acetobacteraceae		0	0	0	1	0	0	0	0	0	0	0	0	1
Cluster19956	Proteobac	Betaproteobacteria				0	0	1	0	0	0	0	0	0	0	0	0	1
Cluster19971	Acidobact	Acidobacteria_Gp2			Gp2	0	0	0	0	1	0	0	0	0	0	0	0	1
Cluster19974	Bacteroid	Sphing	Sphir	Sphingo	Pedobacter	0	0	1	0	0	0	0	0	0	0	0	0	1
Cluster19978;size=1						0	0	1	0	0	0	0	0	0	0	0	0	1
Cluster19979	Acidobact	Acidobacteria_Gp2			Gp2	0	0	0	0	0	0	0	0	0	0	0	1	1
Cluster19995	Proteobac	Deltap	Myxo	Polyangiaceae		0	0	1	0	0	0	0	0	0	0	0	0	1
Cluster19996	Proteobac	Gamm	Xanth	Xanthor	Frateuria	0	0	0	0	0	0	1	0	0	0	0	0	1
Cluster20009	Proteobac	Betapr	Burkl	Burkhol	Burkholderia	0	0	1	0	0	0	0	0	0	0	0	0	1
Cluster20012	Proteobac	Alphap	Rhizo	Methylocystaceae		0	0	0	0	0	0	1	0	0	0	0	0	1
Cluster20015	Proteobac	Alphap	Rhizo	Bradyrhizobiaceae		0	0	1	0	0	0	0	0	0	0	0	0	1
Cluster20018	Proteobacteria					0	0	0	1	0	0	0	0	0	0	0	0	1
Cluster20019;size=1						0	0	0	0	1	0	0	0	0	0	0	0	1
Cluster20020;size=1						0	0	0	0	0	0	1	0	0	0	0	0	1
Cluster20021	Proteobacteria					0	0	1	0	0	0	0	0	0	0	0	0	1
Cluster20028	Chlamydia	Chlamy	Chlar	Parachlamydiaceae		0	0	1	0	0	0	0	0	0	0	0	0	1
Cluster20030	Acidobact	Acidobacteria_Gp1				0	0	0	1	0	0	0	0	0	0	0	0	1
Cluster20036	Proteobac	Alphap	Rhod	Acetoba	Acidisphaera	0	0	0	0	1	0	0	0	0	0	0	0	1
Cluster20039	Acidobacteria					0	0	0	1	0	0	0	0	0	0	0	0	1
Cluster20041	Acidobact	Acidobacteria_Gp2			Gp2	0	0	0	0	0	1	0	0	0	0	0	0	1
Cluster20049	Acidobact	Acidobacteria_Gp2			Gp2	0	0	0	1	0	0	0	0	0	0	0	0	1

Cluster20053	Firmicutes	Bacilli	Bacill	Staphyl	Staphylococcu	0	0	0	0	0	0	1	0	0	0	0	0	1
Cluster20057	Acidobact	Acidobacteria_Gp2			Gp2	0	0	1	0	0	0	0	0	0	0	0	0	1
Cluster20062;size=9						0	0	0	0	0	0	0	1	0	0	0	0	1
Cluster20063	Proteobacteria					0	0	0	0	1	0	0	0	0	0	0	0	1
Cluster20065;size=1						0	0	1	0	0	0	0	0	0	0	0	0	1
Cluster20084	Actinobac	Actinobacteria				0	0	0	0	0	0	1	0	0	0	0	0	1
Cluster20091	Actinobac	Actinol	Solirı	Conexib	Conexibacter	0	0	0	0	0	0	1	0	0	0	0	0	1
Cluster20095	Acidobact	Acidobacteria_Gp3			Gp3	0	0	0	0	0	1	0	0	0	0	0	0	1
Cluster20097	Proteobac	Deltap	Myxococcales			0	0	1	0	0	0	0	0	0	0	0	0	1
Cluster20099	Acidobact	Acidobacteria_Gp1			Acidobacteriur	0	0	1	0	0	0	0	0	0	0	0	0	1
Cluster20115	Bacteroid	Sphing	Sphir	Chitinophagaceae		0	0	0	0	0	0	1	0	0	0	0	0	1
Cluster20131	Proteobac	Gamm	Xantl	Xanthomonadaceae		0	0	0	0	0	0	1	0	0	0	0	0	1
Cluster20132	Acidobact	Acidobacteria_Gp2			Gp2	0	0	0	0	1	0	0	0	0	0	0	0	1
Cluster20133	Proteobac	Deltaproteobacteria				0	0	1	0	0	0	0	0	0	0	0	0	1
Cluster20134	Acidobact	Acidobacteria_Gp3			Gp3	0	0	0	0	1	0	0	0	0	0	0	0	1
Cluster20137	Acidobact	Acidobacteria_Gp2			Gp2	0	0	0	0	1	0	0	0	0	0	0	0	1
Cluster20141	Acidobact	Acidobacteria_Gp3			Gp3	0	0	1	0	0	0	0	0	0	0	0	0	1
Cluster20143	Acidobact	Acidobacteria_Gp1				0	0	0	0	0	1	0	0	0	0	0	0	1
Cluster20145	Acidobact	Acidobacteria_Gp1				0	0	1	0	0	0	0	0	0	0	0	0	1
Cluster20146	Proteobac	Betapr	Burkholderiales			0	0	0	0	0	1	0	0	0	0	0	0	1
Cluster20154	Acidobact	Acidobacteria_Gp3			Gp3	0	0	0	0	0	0	1	0	0	0	0	0	1
Cluster20159;size=2						0	0	0	0	0	1	0	0	0	0	0	0	1
Cluster20165	Proteobac	Deltap	Myxococcales			0	0	0	0	0	1	0	0	0	0	0	0	1
Cluster20170;size=1						0	0	1	0	0	0	0	0	0	0	0	0	1
Cluster20173;size=1						0	0	1	0	0	0	0	0	0	0	0	0	1
Cluster20180	Proteobac	Alphap	Rhizc	Xanthol	Pseudolabrys	0	0	0	1	0	0	0	0	0	0	0	0	1
Cluster20186	Proteobac	Gammaproteobacteria				0	0	1	0	0	0	0	0	0	0	0	0	1
Cluster20195	Proteobacteria					0	0	0	0	0	1	0	0	0	0	0	0	1
Cluster20198	Acidobact	Acidobacteria_Gp2			Gp2	0	0	0	1	0	0	0	0	0	0	0	0	1

Cluster20199	Proteobac	Gamm	Xanthomonadales			0	0	0	0	0	0	0	0	0	1	0	0	1
Cluster20205	Acidobact	Acidobacteria_Gp10			Gp10	0	0	0	1	0	0	0	0	0	0	0	0	1
Cluster20206	Acidobact	Acidobacteria_Gp2			Gp2	0	0	1	0	0	0	0	0	0	0	0	0	1
Cluster20207	Proteobac	Alphap	Sphir	Sphingo	Novosphingob	0	0	0	0	0	1	0	0	0	0	0	0	1
Cluster20213	Proteobac	Deltap	Myxo	Kofleria	Kofleria	0	0	0	0	0	1	0	0	0	0	0	0	1
Cluster20214;size=3						0	0	0	0	0	1	0	0	0	0	0	0	1
Cluster20216	Acidobact	Acidobacteria_Gp2			Gp2	0	0	0	0	0	1	0	0	0	0	0	0	1
Cluster20219	Acidobact	Acidobacteria_Gp3			Gp3	0	0	1	0	0	0	0	0	0	0	0	0	1
Cluster20227	Bacteroid	Sphing	Sphir	Chitinophagaceae		0	0	1	0	0	0	0	0	0	0	0	0	1
Cluster20231	Proteobac	Alphap	Caulo	Caulobа	Phenylobacter	0	0	0	1	0	0	0	0	0	0	0	0	1
Cluster20232	Acidobact	Acidobacteria_Gp3			Bryobacter	0	0	0	0	0	0	0	0	1	0	0	0	1
Cluster20234;size=3						0	0	0	1	0	0	0	0	0	0	0	0	1
Cluster20238	Proteobacteria					0	0	1	0	0	0	0	0	0	0	0	0	1
Cluster20245	Acidobact	Acidobacteria_Gp3			Gp3	0	0	0	0	0	1	0	0	0	0	0	0	1
Cluster20246	Proteobac	Alphaproteobacteria				0	0	0	0	0	1	0	0	0	0	0	0	1
Cluster20253	Acidobact	Acidobacteria_Gp1			Gp1	0	0	1	0	0	0	0	0	0	0	0	0	1
Cluster20255;size=3						0	0	1	0	0	0	0	0	0	0	0	0	1
Cluster20261;size=1						0	0	1	0	0	0	0	0	0	0	0	0	1
Cluster20264	Proteobac	Gammaproteobacteria				0	0	0	0	0	1	0	0	0	0	0	0	1
Cluster20268	Proteobac	Alphap	Alphaproteob		Rhizomicrobiu	0	0	0	0	1	0	0	0	0	0	0	0	1
Cluster20270	Proteobac	Alphap	Rhod	Rhodospirillaceae		0	0	0	1	0	0	0	0	0	0	0	0	1
Cluster20272	Acidobact	Acidobacteria_Gp2			Gp2	0	0	0	1	0	0	0	0	0	0	0	0	1
Cluster20274	Proteobac	Gammaproteobacteria				0	0	0	0	0	0	1	0	0	0	0	0	1
Cluster20275	Verrucom	Subdivision3			Subdivision3_g	0	0	0	1	0	0	0	0	0	0	0	0	1
Cluster20279	Proteobac	Alphap	Rhodospirillales			0	0	0	0	0	1	0	0	0	0	0	0	1
Cluster20285	Proteobac	Alphap	Rhizobiales			0	0	0	0	1	0	0	0	0	0	0	0	1
Cluster20289	Acidobact	Acidobacteria_Gp2			Gp2	0	0	0	1	0	0	0	0	0	0	0	0	1
Cluster20290	Proteobac	Betaproteobacteria				0	0	0	0	0	0	0	1	0	0	0	0	1
Cluster20291	Proteobac	Alphap	Rhodospirillales			0	0	0	1	0	0	0	0	0	0	0	0	1

Cluster20293	Proteobac	Gammaproteobacteria				0	0	0	0	0	1	0	0	0	0	0	0	1
Cluster20310	Proteobac	Betapr	Burkl	Burkhol	Burkholderia	0	0	0	0	0	0	1	0	0	0	0	0	1
Cluster20315	Armatimo	Armati	Arma	Armatin	Armatimonas/	0	0	0	0	0	1	0	0	0	0	0	0	1
Cluster20324;size=1						0	0	0	1	0	0	0	0	0	0	0	0	1
Cluster20337;size=1						0	0	0	1	0	0	0	0	0	0	0	0	1
Cluster20338	Acidobact	Acidobacteria_Gp1				0	0	1	0	0	0	0	0	0	0	0	0	1
Cluster20344	Acidobact	Acidobacteria_Gp3			Gp3	0	0	0	1	0	0	0	0	0	0	0	0	1
Cluster20347	Bacteroid	Sphing	Sphingobacteriales			0	0	0	0	0	1	0	0	0	0	0	0	1
Cluster20349	Armatimo	Armati	Arma	Armatin	Armatimonas/	0	0	0	0	0	1	0	0	0	0	0	0	1
Cluster20350	Proteobac	Alphap	Alphaproteol		Rhizomicrobiu	0	0	0	1	0	0	0	0	0	0	0	0	1
Cluster20352	Acidobact	Acidobacteria_Gp2			Gp2	0	0	0	1	0	0	0	0	0	0	0	0	1
Cluster20365	Acidobact	Acidobacteria_Gp2			Gp2	0	0	0	1	0	0	0	0	0	0	0	0	1
Cluster20367	Verrucom	Opituta	Opitu	Opituta	Opitutus	0	0	0	1	0	0	0	0	0	0	0	0	1
Cluster20372	Proteobacteria					0	0	0	1	0	0	0	0	0	0	0	0	1
Cluster20375	Proteobac	Alphap	Rhizobiales			0	0	0	0	1	0	0	0	0	0	0	0	1
Cluster20377	Acidobact	Acidobacteria_Gp2			Gp2	0	0	0	0	0	0	0	1	0	0	0	0	1
Cluster20379	Proteobac	Alphap	Rhod	Rhodospirillaceae		0	0	0	0	0	0	0	1	0	0	0	0	1
Cluster20380	Proteobac	Alphap	Alphaproteol		Rhizomicrobiu	0	0	0	1	0	0	0	0	0	0	0	0	1
Cluster20385	Chlamydia	Chlamy	Chlar	Parachlamydiaceae		0	0	0	0	0	1	0	0	0	0	0	0	1
Cluster20386	Proteobac	Gamm	Xantl	Xanthor	Thermomonas	0	0	0	0	0	1	0	0	0	0	0	0	1
Cluster20389	Proteobac	Alphap	Rhodospirillales			0	0	0	1	0	0	0	0	0	0	0	0	1
Cluster20392	Proteobac	Alphap	Rhod	Acetoba	Acidisphaera	0	0	0	1	0	0	0	0	0	0	0	0	1
Cluster20405	Acidobact	Acidobacteria_Gp2			Gp2	0	0	0	1	0	0	0	0	0	0	0	0	1
Cluster20412	Actinobac	Actinol	Solirı	Solirubr	Solirubrobacte	0	0	0	1	0	0	0	0	0	0	0	0	1
Cluster20416;size=2						0	0	0	0	0	0	1	0	0	0	0	0	1
Cluster20419	Acidobact	Acidobacteria_Gp1				0	0	0	0	0	1	0	0	0	0	0	0	1
Cluster20422	Acidobact	Acidobacteria_Gp3			Gp3	0	0	1	0	0	0	0	0	0	0	0	0	1
Cluster20427	Proteobac	Alphap	Alphaproteol		Rhizomicrobiu	0	0	0	1	0	0	0	0	0	0	0	0	1
Cluster20430;size=1						0	0	0	1	0	0	0	0	0	0	0	0	1

Cluster20431;size=2						0	0	1	0	0	0	0	0	0	0	0	0	1
Cluster20432	Actinobac	Actinol	Actinomycetales			0	0	1	0	0	0	0	0	0	0	0	0	1
Cluster20438;size=2						1	0	0	0	0	0	0	0	0	0	0	0	1
Cluster20444	Proteobac	Alphaproteobacteria				0	0	0	1	0	0	0	0	0	0	0	0	1
Cluster20447	Proteobac	Alphaproteobacteria				0	0	0	1	0	0	0	0	0	0	0	0	1
Cluster20453	Proteobac	Gamm	Xantl	Sinobac	Steroidobacter	0	0	0	0	0	0	0	0	0	0	0	1	1
Cluster20456	Proteobac	Alphaproteobacteria				0	0	0	0	0	0	0	0	0	1	0	0	1
Cluster20469	Acidobact	Acidobacteria_Gp1				0	0	0	0	0	0	0	0	0	0	1	0	1
Cluster20471	Acidobact	Acidobacteria_Gp1				0	0	1	0	0	0	0	0	0	0	0	0	1
Cluster20472	Proteobac	Alphaproteobacteria				0	0	0	0	0	1	0	0	0	0	0	0	1
Cluster20484	Acidobact	Acidobacteria_Gp1			Granulicella	0	0	1	0	0	0	0	0	0	0	0	0	1
Cluster20486	Actinobac	Actinol	Solirı	Conexib	Conexibacter	0	0	0	0	0	0	0	0	0	1	0	0	1
Cluster20497	Proteobac	Alphap	Alphaproteol		Rhizomicrobiu	0	0	1	0	0	0	0	0	0	0	0	0	1
Cluster20503	Proteobac	Alphap	Alphaproteol		Rhizomicrobiu	0	0	1	0	0	0	0	0	0	0	0	0	1
Cluster20507;size=1						0	0	0	1	0	0	0	0	0	0	0	0	1
Cluster20517	Proteobac	Alphap	Alphaproteol		Rhizomicrobiu	0	0	0	0	0	0	1	0	0	0	0	0	1
Cluster20532	Proteobac	Gamm	Xantl	Sinobacteraceae		0	0	0	0	0	0	1	0	0	0	0	0	1
Cluster20534	Proteobac	Alphap	Rhizobiales			0	0	1	0	0	0	0	0	0	0	0	0	1
Cluster20544	Acidobact	Acidobacteria_Gp2			Gp2	1	0	0	0	0	0	0	0	0	0	0	0	1
Cluster20555	Chloroflex	Ktedor	Kted	Ktedon	Ktedonobacter	0	0	1	0	0	0	0	0	0	0	0	0	1
Cluster20557	Proteobac	Alphap	Rhod	Acetobacteraceae		0	0	1	0	0	0	0	0	0	0	0	0	1
Cluster20563	Bacteroidetes					0	1	0	0	0	0	0	0	0	0	0	0	1
Cluster20564	Bacteroid	Sphing	Sphir	Sphingobacteriaceae		0	0	0	0	0	0	1	0	0	0	0	0	1
Cluster20568	Proteobac	Gammaproteobacteria				0	0	0	1	0	0	0	0	0	0	0	0	1
Cluster20569	Verrucom	Opituta	Opitu	Opituta	Opitutus	0	0	1	0	0	0	0	0	0	0	0	0	1
Cluster20602	Verrucom	Opitutae				0	0	0	1	0	0	0	0	0	0	0	0	1
Cluster20618	Acidobact	Acidobacteria_Gp2			Gp2	0	0	0	1	0	0	0	0	0	0	0	0	1
Cluster20622	Acidobact	Acidobacteria_Gp2			Gp2	0	0	0	1	0	0	0	0	0	0	0	0	1
Cluster20623	Proteobac	Alphap	Rhod	Acetoba	Acidisphaera	0	0	0	1	0	0	0	0	0	0	0	0	1

Cluster20625	Proteobac	Alphap	Rhod	Acetoba	Acidisphaera	0	0	0	1	0	0	0	0	0	0	0	0	1
Cluster20633	Acidobact	Acidobacteria_Gp1				0	0	0	0	0	1	0	0	0	0	0	0	1
Cluster20637	Acidobact	Acidobacteria_Gp1				1	0	0	0	0	0	0	0	0	0	0	0	1
Cluster20640	Proteobacteria					0	0	0	0	0	1	0	0	0	0	0	0	1
Cluster20648	Proteobac	Alphap	Rhodospirillales			0	0	0	0	0	1	0	0	0	0	0	0	1
Cluster20653	Proteobac	Gammaproteobacteria				0	0	1	0	0	0	0	0	0	0	0	0	1
Cluster20658	Proteobac	Alphap	Rhod	Acetoba	Acidisphaera	0	0	0	1	0	0	0	0	0	0	0	0	1
Cluster20659	Bacteroid	Sphing	Sphir	Chitinophagaceae		0	0	0	1	0	0	0	0	0	0	0	0	1
Cluster20660	Acidobacteria					0	0	1	0	0	0	0	0	0	0	0	0	1
Cluster20664	Proteobac	Deltap	Bdell	Bdellovi	Bdellovibrio	0	0	1	0	0	0	0	0	0	0	0	0	1
Cluster20667	Verrucom	Opitut	Opitu	Opituta	Opitutus	1	0	0	0	0	0	0	0	0	0	0	0	1
Cluster20672	Actinobac	Actinob	Soliru	Solirubr	Solirubrobacte	0	0	0	1	0	0	0	0	0	0	0	0	1
Cluster20677	Proteobac	Alphap	Rhizobiales			0	0	0	1	0	0	0	0	0	0	0	0	1
Cluster20712	Acidobact	Acidobacteria_Gp2			Gp2	0	0	1	0	0	0	0	0	0	0	0	0	1
Cluster20717	Proteobac	Alphap	Caulo	Cauloba	Caulobacter	0	0	1	0	0	0	0	0	0	0	0	0	1
Cluster20718	Verrucomicrobia					0	0	0	0	0	0	1	0	0	0	0	0	1
Cluster20720	Acidobact	Acidobacteria_Gp2			Gp2	0	0	0	1	0	0	0	0	0	0	0	0	1
Cluster20724	Acidobact	Acidobacteria_Gp2			Gp2	0	0	0	0	1	0	0	0	0	0	0	0	1
Cluster20727	Acidobacteria					0	0	0	0	1	0	0	0	0	0	0	0	1
Cluster20735	Armatimo	Chthon	Chth	Chthono	Chthonomona	0	0	0	0	1	0	0	0	0	0	0	0	1
Cluster20738	Acidobact	Acidobacteria_Gp1			Gp1	0	0	0	0	1	0	0	0	0	0	0	0	1
Cluster20747	Acidobact	Acidobacteria_Gp3			Gp3	0	0	0	1	0	0	0	0	0	0	0	0	1
Cluster20754	Acidobact	Acidobacteria_Gp2			Gp2	0	0	0	1	0	0	0	0	0	0	0	0	1
Cluster20760	Acidobact	Acidobacteria_Gp2			Gp2	0	0	0	1	0	0	0	0	0	0	0	0	1
Cluster20762;size=1						0	0	0	0	0	0	1	0	0	0	0	0	1
Cluster20764	Acidobact	Acidobacteria_Gp3			Gp3	0	1	0	0	0	0	0	0	0	0	0	0	1
Cluster20765	Acidobact	Acidobacteria_Gp1			Gp1	0	0	0	0	0	0	1	0	0	0	0	0	1
Cluster20772	Actinobac	Actinob	Actin	Actinos	Actinospica	0	0	0	0	0	0	0	0	0	0	0	1	1
Cluster20784	Acidobact	Acidobacteria_Gp2			Gp2	1	0	0	0	0	0	0	0	0	0	0	0	1

Cluster20785	Proteobac	Deltap	Myxococcales			0	0	0	0	0	0	1	0	0	0	0	0	1
Cluster20787	Proteobac	Alphap	Rhod	Acetobacteraceae		0	0	0	1	0	0	0	0	0	0	0	0	1
Cluster20788	Proteobac	Alphaproteobacteria				0	1	0	0	0	0	0	0	0	0	0	0	1
Cluster20789	Proteobac	Alphaproteobacteria				0	0	0	0	1	0	0	0	0	0	0	0	1
Cluster20791;size=1						0	0	0	1	0	0	0	0	0	0	0	0	1
Cluster20793	Acidobact	Acidobacteria_Gp3			Bryobacter	0	0	0	0	0	0	1	0	0	0	0	0	1
Cluster20796	Acidobact	Acidobacteria_Gp1			Gp1	0	0	0	0	0	0	0	0	1	0	0	0	1
Cluster20799	Acidobact	Acidobacteria_Gp3			Gp3	1	0	0	0	0	0	0	0	0	0	0	0	1
Cluster20804	Proteobac	Deltaproteobacteria				0	1	0	0	0	0	0	0	0	0	0	0	1
Cluster20806	Proteobac	Alphap	Rhizobiales			0	0	0	0	0	0	1	0	0	0	0	0	1
Cluster20811	Acidobact	Acidobacteria_Gp2			Gp2	0	1	0	0	0	0	0	0	0	0	0	0	1
Cluster20816;size=3						0	0	1	0	0	0	0	0	0	0	0	0	1
Cluster20819	Actinobac	Actinol	Acidi	Acidimi	Aciditerrimona	0	0	0	0	1	0	0	0	0	0	0	0	1
Cluster20825	Acidobact	Acidobacteria_Gp1				0	1	0	0	0	0	0	0	0	0	0	0	1
Cluster20834	Acidobact	Acidobacteria_Gp1				0	0	0	0	1	0	0	0	0	0	0	0	1
Cluster20841	Acidobact	Acidobacteria_Gp1			Gp1	0	0	0	0	1	0	0	0	0	0	0	0	1
Cluster20843	Bacteroid	Sphing	Sphir	Chitinophagaceae		0	0	0	0	1	0	0	0	0	0	0	0	1
Cluster20852	Proteobac	Alphap	Rhizobiales			0	1	0	0	0	0	0	0	0	0	0	0	1
Cluster20861	Actinobac	Actinol	Solirubrobacterales			0	1	0	0	0	0	0	0	0	0	0	0	1
Cluster20863	Acidobact	Acidobacteria_Gp10			Gp10	0	0	0	0	0	0	0	0	0	0	1	0	1
Cluster20868	Proteobac	Alphap	Rhod	Acetoba	Acidisphaera	0	0	0	0	1	0	0	0	0	0	0	0	1
Cluster20878	Proteobac	Gammaproteobacteria				0	0	0	0	1	0	0	0	0	0	0	0	1
Cluster20889;size=3						0	1	0	0	0	0	0	0	0	0	0	0	1
Cluster20892	Acidobact	Acidobacteria_Gp3			Gp3	0	0	0	0	1	0	0	0	0	0	0	0	1
Cluster20895	Proteobac	Betaproteobacteria				0	0	0	0	0	0	1	0	0	0	0	0	1
Cluster20898	Proteobac	Gamm	Xantl	Sinobac	Steroidobacter	0	0	0	0	1	0	0	0	0	0	0	0	1
Cluster20903	Acidobact	Acidobacteria_Gp3			Gp3	0	0	0	0	1	0	0	0	0	0	0	0	1
Cluster20904	Proteobac	Alphap	Rhod	Acetobacteraceae		0	0	0	1	0	0	0	0	0	0	0	0	1
Cluster20907	Actinobac	Actinol	Acidimicrobiales			0	0	0	0	0	0	1	0	0	0	0	0	1

Cluster20908	Proteobac	Alphap	Rhodospirillales			0	0	0	0	1	0	0	0	0	0	0	0	1
Cluster20909	Acidobacteria					0	0	0	0	1	0	0	0	0	0	0	0	1
Cluster20911	Proteobac	Alphap	Rhod	Acetoba	Acidisphaera	0	0	0	0	1	0	0	0	0	0	0	0	1
Cluster20916	Proteobac	Gammaproteobacteria				0	0	0	1	0	0	0	0	0	0	0	0	1
Cluster20921	Acidobact	Acidobacteria_Gp2			Gp2	0	0	1	0	0	0	0	0	0	0	0	0	1
Cluster20930	Acidobact	Acidobacteria_Gp1			Gp1	0	0	0	0	1	0	0	0	0	0	0	0	1
Cluster20937	Proteobac	Betapr	Burkl	Burkhol	Ralstonia	0	0	0	0	1	0	0	0	0	0	0	0	1
Cluster20938	Proteobac	Gamm	Legio	Coxiella	Aquicella	0	1	0	0	0	0	0	0	0	0	0	0	1
Cluster20943	Acidobact	Acidobacteria_Gp2			Gp2	0	0	0	0	0	0	1	0	0	0	0	0	1
Cluster20945	Acidobacteria					1	0	0	0	0	0	0	0	0	0	0	0	1
Cluster20949	Proteobac	Betapr	Burkl	Oxalobacteraceae		0	0	0	1	0	0	0	0	0	0	0	0	1
Cluster20953	Proteobac	Alphaproteobacteria				1	0	0	0	0	0	0	0	0	0	0	0	1
Cluster20954	Acidobact	Acidobacteria_Gp2			Gp2	0	0	0	0	0	0	1	0	0	0	0	0	1
Cluster20958	Proteobac	Gammaproteobacteria				1	0	0	0	0	0	0	0	0	0	0	0	1
Cluster20961;size=1						1	0	0	0	0	0	0	0	0	0	0	0	1
Cluster20962	Proteobac	Gammaproteobacteria				1	0	0	0	0	0	0	0	0	0	0	0	1
Cluster20964	Actinobac	Actinol	Actin	Nocardiaceae		1	0	0	0	0	0	0	0	0	0	0	0	1
Cluster20973	Proteobacteria					0	0	0	0	1	0	0	0	0	0	0	0	1
Cluster20974	Proteobac	Alphap	Rhizc	Beijerin	Methylocapsa	1	0	0	0	0	0	0	0	0	0	0	0	1
Cluster20977	Proteobac	Alphap	Alphaproteol		Rhizomicrobiu	1	0	0	0	0	0	0	0	0	0	0	0	1
Cluster20981;size=1						1	0	0	0	0	0	0	0	0	0	0	0	1
Cluster20982	Bacteroid	Sphing	Sphir	Chitinophagaceae		1	0	0	0	0	0	0	0	0	0	0	0	1
Cluster20988	Proteobacteria					0	0	0	1	0	0	0	0	0	0	0	0	1
Cluster20989	Acidobact	Acidobacteria_Gp2			Gp2	0	0	0	1	0	0	0	0	0	0	0	0	1
Cluster20994	Bacteroid	Sphing	Sphir	Sphingo	Pedobacter	0	1	0	0	0	0	0	0	0	0	0	0	1
Cluster20999	Acidobact	Acidobacteria_Gp13			Gp13	1	0	0	0	0	0	0	0	0	0	0	0	1
Cluster21001	Acidobact	Acidobacteria_Gp5			Gp5	0	0	0	0	0	0	1	0	0	0	0	0	1
Cluster21006	Acidobact	Acidobacteria_Gp2			Gp2	1	0	0	0	0	0	0	0	0	0	0	0	1
Cluster21007	Acidobact	Acidobacteria_Gp10			Gp10	0	0	0	1	0	0	0	0	0	0	0	0	1

Cluster2100{illegible}	Acidobact	Acidobacteria_Gp2			Gp2	1	0	0	0	0	0	0	0	0	0	0	0	1
Cluster21009;size=2						0	0	0	0	1	0	0	0	0	0	0	0	1
Cluster21011	Proteobac	Gammaproteobacteria				0	0	0	0	0	0	0	0	0	1	0	0	1
Cluster2101[illegible]	Acidobact	Acidobacteria_Gp1			Edaphobacter	1	0	0	0	0	0	0	0	0	0	0	0	1
Cluster2101[illegible]	Proteobac	Betapr	Burkholderiales			1	0	0	0	0	0	0	0	0	0	0	0	1
Cluster2101[illegible]	Acidobact	Acidobacteria_Gp1			Granulicella	0	0	0	0	1	0	0	0	0	0	0	0	1
Cluster2102[illegible]	Proteobac	Alphaproteobacteria				0	1	0	0	0	0	0	0	0	0	0	0	1
Cluster21024;size=1						1	0	0	0	0	0	0	0	0	0	0	0	1
Cluster2102[illegible]	Acidobact	Acidobacteria_Gp3			Gp3	1	0	0	0	0	0	0	0	0	0	0	0	1
Cluster2102[illegible]	Actinobac	Actinol	Acidi	Acidimi	Aciditerrimona	0	0	0	0	0	0	0	1	0	0	0	0	1
Cluster2102[illegible]	Acidobact	Acidobacteria_Gp1			Gp1	1	0	0	0	0	0	0	0	0	0	0	0	1
Cluster21031	Proteobac	Betapr	Burkl	Burkhol	Burkholderia	1	0	0	0	0	0	0	0	0	0	0	0	1
Cluster21034;size=1						1	0	0	0	0	0	0	0	0	0	0	0	1
Cluster2103[illegible]	Acidobact	Acidobacteria_Gp1			Gp1	0	0	0	0	0	0	1	0	0	0	0	0	1
Cluster2103[illegible]	Proteobac	Alphap	Rhod	Rhodospirillaceae		1	0	0	0	0	0	0	0	0	0	0	0	1
Cluster2103[illegible]	Acidobact	Acidobacteria_Gp2			Gp2	1	0	0	0	0	0	0	0	0	0	0	0	1
Cluster2103[illegible]	Acidobact	Acidobacteria_Gp10			Gp10	1	0	0	0	0	0	0	0	0	0	0	0	1
Cluster21041	Acidobact	Acidobacteria_Gp10			Gp10	1	0	0	0	0	0	0	0	0	0	0	0	1
Cluster2104[illegible]	Gemmatii	Gemm	Gemi	Gemma	Gemmatimona	1	0	0	0	0	0	0	0	0	0	0	0	1
Cluster2104[illegible]	Acidobacteria					1	0	0	0	0	0	0	0	0	0	0	0	1
Cluster2105[illegible]	Proteobac	Gammaproteobacteria				1	0	0	0	0	0	0	0	0	0	0	0	1
Cluster2105[illegible]	Actinobac	Actinol	Actinomycetales			0	0	0	0	0	0	0	1	0	0	0	0	1
Cluster2105[illegible]	Acidobact	Acidobacteria_Gp1				1	0	0	0	0	0	0	0	0	0	0	0	1
Cluster2105[illegible]	Proteobac	Alphap	Rhod	Rhodospirillaceae		1	0	0	0	0	0	0	0	0	0	0	0	1
Cluster2105[illegible]	Acidobact	Acidobacteria_Gp1			Gp1	1	0	0	0	0	0	0	0	0	0	0	0	1
Cluster2105[illegible]	Acidobact	Acidobacteria_Gp2			Gp2	1	0	0	0	0	0	0	0	0	0	0	0	1
Cluster2106[illegible]	Acidobact	Acidobacteria_Gp1				0	0	0	1	0	0	0	0	0	0	0	0	1
Cluster2106[illegible]	Proteobac	Gammaproteobacteria				1	0	0	0	0	0	0	0	0	0	0	0	1
Cluster2106[illegible]	Acidobact	Acidobacteria_Gp3			Gp3	1	0	0	0	0	0	0	0	0	0	0	0	1

Cluster21070	Acidobact	Acidobacteria_Gp2			Gp2	0	1	0	0	0	0	0	0	0	0	0	0	1
Cluster21071	Proteobac	Betapr	Burkl	Oxalobacteraceae		1	0	0	0	0	0	0	0	0	0	0	0	1
Cluster21074	Proteobac	Gammaproteobacteria				1	0	0	0	0	0	0	0	0	0	0	0	1
Cluster21080	Proteobac	Deltap	Myxc	Polyangiaceae		1	0	0	0	0	0	0	0	0	0	0	0	1
Cluster21082	Proteobac	Alphap	Rhodospirillales			1	0	0	0	0	0	0	0	0	0	0	0	1
Cluster21084	TM7				TM7_genera_i	1	0	0	0	0	0	0	0	0	0	0	0	1
Cluster21086	Actinobac	Actinol	Actinomycetales			0	0	0	0	0	1	0	0	0	0	0	0	1
Cluster21090	Acidobact	Acidobacteria_Gp3				0	0	0	0	1	0	0	0	0	0	0	0	1
Cluster21094	Firmicute:	Bacilli	Bacill	Paeniba	Paenibacillus	0	0	0	0	0	0	0	0	1	0	0	0	1
Cluster21098	Nitrospira	Nitrosp	Nitro	Nitrospi	Nitrospira	0	1	0	0	0	0	0	0	0	0	0	0	1
Cluster21101	Acidobact	Acidobacteria_Gp2			Gp2	0	0	0	0	1	0	0	0	0	0	0	0	1
Cluster21102	Actinobac	Actinol	Actin	Actinos	Actinospica	0	0	0	0	0	0	1	0	0	0	0	0	1
Cluster21104	Proteobacteria					0	1	0	0	0	0	0	0	0	0	0	0	1
Cluster21109	TM7				TM7_genera_i	0	1	0	0	0	0	0	0	0	0	0	0	1
Cluster21117	Proteobacteria					0	0	1	0	0	0	0	0	0	0	0	0	1
Cluster21126	Acidobact	Acidobacteria_Gp3				0	0	0	1	0	0	0	0	0	0	0	0	1
Cluster21139	Actinobac	Actinol	Actinomycetales			0	0	0	0	1	0	0	0	0	0	0	0	1
Cluster21150	Proteobac	Deltaproteobacteria				0	1	0	0	0	0	0	0	0	0	0	0	1
Cluster21153	Acidobact	Acidobacteria_Gp2			Gp2	0	0	0	0	1	0	0	0	0	0	0	0	1
Cluster21155;size=1						0	1	0	0	0	0	0	0	0	0	0	0	1
Cluster21156;size=3						1	0	0	0	0	0	0	0	0	0	0	0	1
Cluster21157	Acidobact	Acidobacteria_Gp2			Gp2	0	0	0	0	1	0	0	0	0	0	0	0	1
Cluster21158	Acidobact	Acidobacteria_Gp2			Gp2	0	0	0	0	1	0	0	0	0	0	0	0	1
Cluster21160	Proteobac	Alphap	Rhodospirillales			0	0	0	0	0	0	1	0	0	0	0	0	1
Cluster21165	Acidobact	Acidobacteria_Gp1			Granulicella	0	0	0	1	0	0	0	0	0	0	0	0	1
Cluster21167	Proteobacteria					0	1	0	0	0	0	0	0	0	0	0	0	1
Cluster21172	Proteobac	Gamm	Xantl	Sinobac	Steroidobacter	0	1	0	0	0	0	0	0	0	0	0	0	1
Cluster21181	Chlamydia	Chlamy	Chlar	Parachla	Neochlamydia	0	0	0	0	1	0	0	0	0	0	0	0	1
Cluster21182	Actinobac	Actinol	Soliru	Solirubr	Solirubrobacte	0	0	0	0	0	0	1	0	0	0	0	0	1

Cluster21187	Actinobac	Actinob	Soliru	Solirubr	Solirubrobacte	0	0	0	0	1	0	0	0	0	0	0	0	1
Cluster21192	Acidobact	Acidobacteria_Gp1			Edaphobacter	0	0	0	0	1	0	0	0	0	0	0	0	1
Cluster21193	Proteobac	Alphap	Rhod	Acetobacteraceae		0	0	0	0	0	0	1	0	0	0	0	0	1
Cluster21204	Actinobac	Actinob	Acidimicrobiales			0	0	0	0	1	0	0	0	0	0	0	0	1
Cluster21210	Proteobac	Alphap	Alphaproteob		Rhizomicrobiu	0	0	0	1	0	0	0	0	0	0	0	0	1
Cluster21215	Acidobact	Acidobacteria_Gp2			Gp2	0	1	0	0	0	0	0	0	0	0	0	0	1
Cluster21225	Proteobac	Alphap	Rhod	Acetoba	Acidisphaera	0	0	0	0	0	0	1	0	0	0	0	0	1
Cluster21234	Verrucom	Subdivision3			Subdivision3_g	0	0	0	0	1	0	0	0	0	0	0	0	1
Cluster21243	Actinobac	Actinob	Solirubrobacterales			0	0	0	0	0	0	1	0	0	0	0	0	1
Cluster21253	Proteobac	Gammaproteobacteria				0	1	0	0	0	0	0	0	0	0	0	0	1
Cluster21254	Proteobac	Gammaproteobacteria				0	1	0	0	0	0	0	0	0	0	0	0	1
Cluster21259	Proteobac	Gammaproteobacteria				0	0	0	0	0	0	1	0	0	0	0	0	1
Cluster21266	Acidobact	Acidobacteria_Gp2			Gp2	0	0	0	0	0	0	1	0	0	0	0	0	1
Cluster21270	Acidobact	Acidobacteria_Gp3			Gp3	0	0	0	0	0	0	1	0	0	0	0	0	1
Cluster21272	Acidobact	Acidobacteria_Gp2			Gp2	0	0	1	0	0	0	0	0	0	0	0	0	1
Cluster21275	Proteobac	Gamm	Xanth	Sinobac	Steroidobacter	0	0	1	0	0	0	0	0	0	0	0	0	1
Cluster21279;size=1						0	1	0	0	0	0	0	0	0	0	0	0	1
Cluster21282	Proteobac	Alphap	Rhizobiales			0	0	1	0	0	0	0	0	0	0	0	0	1
Cluster21285	Acidobact	Acidobacteria_Gp3			Gp3	0	0	0	0	0	0	0	0	0	0	0	1	1
Cluster21291	Proteobac	Gamm	Xanth	Xanthom	Frateuria	0	0	0	0	0	0	1	0	0	0	0	0	1
Cluster21313	Proteobac	Betapr	Burkholderiales			0	0	0	1	0	0	0	0	0	0	0	0	1
Cluster21318	Proteobac	Alphap	Rhod	Acetobacteraceae		0	0	0	1	0	0	0	0	0	0	0	0	1
Cluster21322;size=1						0	0	1	0	0	0	0	0	0	0	0	0	1
Cluster21328	Proteobac	Alphap	Rhizo	Hyphomicrobiaceae		0	0	0	0	1	0	0	0	0	0	0	0	1
Cluster21336	Proteobac	Alphap	Rhod	Acetobacteraceae		1	0	0	0	0	0	0	0	0	0	0	0	1
Cluster21345	Acidobact	Acidobacteria_Gp10			Gp10	0	0	1	0	0	0	0	0	0	0	0	0	1
Cluster21350	Acidobact	Acidobacteria_Gp1			Edaphobacter	0	0	1	0	0	0	0	0	0	0	0	0	1
Cluster21361	Proteobac	Alphap	Alphaproteob		Rhizomicrobiu	0	0	0	1	0	0	0	0	0	0	0	0	1
Cluster21365	Verrucom	Subdivision3			Subdivision3_g	0	0	0	0	0	0	1	0	0	0	0	0	1

Cluster21367	Acidobact	Acidobacteria_Gp2			Gp2	0	0	0	0	0	0	0	0	0	1	0	0	1
Cluster21369	Proteobac	Gammaproteobacteria				0	0	0	0	0	0	0	0	0	0	1	0	1
Cluster21374	Proteobac	Alphaproteobacteria				0	0	0	0	1	0	0	0	0	0	0	0	1
Cluster21388	Acidobact	Acidobacteria_Gp1				0	0	0	0	1	0	0	0	0	0	0	0	1
Cluster21391	Chlamydia	Chlamy	Chlar	Parachla	Parachlamydia	0	1	0	0	0	0	0	0	0	0	0	0	1
Cluster21394	Acidobact	Acidobacteria_Gp1			Gp1	0	0	0	0	0	0	1	0	0	0	0	0	1
Cluster21399	Proteobac	Alphap	Rhizo	Beijerinckiaceae		0	0	1	0	0	0	0	0	0	0	0	0	1
Cluster21408	Acidobacteria					0	0	0	0	0	0	0	0	0	0	0	1	1
Cluster21410	Acidobact	Acidobacteria_Gp2			Gp2	0	0	1	0	0	0	0	0	0	0	0	0	1
Cluster21415	Verrucom	Spartobacteria			Spartobacteria	0	0	0	0	1	0	0	0	0	0	0	0	1
Cluster21418	Chlamydia	Chlamy	Chlamydiales			0	0	0	0	0	0	0	0	1	0	0	0	1
Cluster21421	Proteobac	Alphap	Rhod	Acetobacteraceae		0	0	0	1	0	0	0	0	0	0	0	0	1
Cluster21432	Elusimicro	Elusim	Elusi	Elusimic	Elusimicrobium	0	0	0	0	1	0	0	0	0	0	0	0	1
Cluster21441	Actinobac	Actinol	Soliru	Conexib	Conexibacter	0	0	0	0	1	0	0	0	0	0	0	0	1
Cluster21444	Proteobac	Alphap	Alphaproteob		Rhizomicrobiu	0	0	0	1	0	0	0	0	0	0	0	0	1
Cluster21450	Acidobact	Acidobacteria_Gp2			Gp2	0	0	1	0	0	0	0	0	0	0	0	0	1
Cluster21456	Bacteroid	Sphing	Sphir	Chitinop	Sediminibacter	0	0	0	0	0	0	1	0	0	0	0	0	1
Cluster21459	Proteobac	Alphap	Sphir	Sphingo	Novosphingob	0	0	0	0	1	0	0	0	0	0	0	0	1
Cluster21461	Acidobact	Acidobacteria_Gp6			Gp6	0	0	0	0	1	0	0	0	0	0	0	0	1
Cluster21479	Proteobac	Betapr	Burkholderiales			0	0	0	0	1	0	0	0	0	0	0	0	1
Cluster21484	Proteobac	Alphap	Caulo	Cauloba	Caulobacter	1	0	0	0	0	0	0	0	0	0	0	0	1
Cluster21486	Acidobact	Acidobacteria_Gp1				0	0	0	0	0	0	1	0	0	0	0	0	1
Cluster21494	Proteobac	Alphap	Alphaproteob		Rhizomicrobiu	0	1	0	0	0	0	0	0	0	0	0	0	1
Cluster21497	Chlamydia	Chlamy	Chlar	Parachla	Parachlamydia	0	0	0	0	0	1	0	0	0	0	0	0	1
Cluster21498	Proteobac	Deltap	Myxococcales			0	0	0	0	1	0	0	0	0	0	0	0	1
Cluster21500	Acidobact	Acidobacteria_Gp2			Gp2	0	0	0	1	0	0	0	0	0	0	0	0	1
Cluster21502	Acidobact	Acidobacteria_Gp2			Gp2	0	0	0	0	1	0	0	0	0	0	0	0	1
Cluster21505	Acidobact	Acidobacteria_Gp1			Edaphobacter	0	0	1	0	0	0	0	0	0	0	0	0	1
Cluster21511	Proteobac	Betapr	Burkl	Oxaloba	Undibacterium	0	0	0	0	1	0	0	0	0	0	0	0	1

Cluster21512;size=1						0	0	0	0	1	0	0	0	0	0	0	0	1
Cluster21514	Proteobac	Alphap	Rhod	Acetobacteraceae		0	0	0	0	0	0	1	0	0	0	0	0	1
Cluster21516	Proteobac	Gamm	Enter	Enterobacteriaceae		0	0	0	0	1	0	0	0	0	0	0	0	1
Cluster21521	Proteobac	Alphap	Alphaproteob		Rhizomicrobiu	0	0	0	1	0	0	0	0	0	0	0	0	1
Cluster21535	Proteobac	Alphap	Rhizc	Bradyrhizobiaceae		0	0	0	0	0	0	1	0	0	0	0	0	1
Cluster21542	Proteobac	Alphaproteobacteria				0	0	0	0	0	0	1	0	0	0	0	0	1
Cluster21546	Actinobac	Actinobacteria				0	1	0	0	0	0	0	0	0	0	0	0	1
Cluster21558	Proteobac	Deltaproteobacteria				0	0	0	0	1	0	0	0	0	0	0	0	1
Cluster21561;size=1						0	0	1	0	0	0	0	0	0	0	0	0	1
Cluster21563	Proteobac	Alphap	Alphaproteob		Rhizomicrobiu	0	0	0	0	0	0	1	0	0	0	0	0	1
Cluster21569	Proteobac	Alphap	Rhod	Acetobacteraceae		0	0	0	0	1	0	0	0	0	0	0	0	1
Cluster21578	Acidobact	Acidobacteria_Gp1			Gp1	0	0	0	0	1	0	0	0	0	0	0	0	1
Cluster21579	Proteobac	Gammaproteobacteria				0	0	0	0	1	0	0	0	0	0	0	0	1
Cluster21588	Acidobact	Acidobacteria_Gp2			Gp2	0	0	0	0	0	0	1	0	0	0	0	0	1
Cluster21589;size=1						0	0	1	0	0	0	0	0	0	0	0	0	1
Cluster21594;size=1						0	0	1	0	0	0	0	0	0	0	0	0	1
Cluster21595	Proteobac	Deltap	Myxc	Polyangiaceae		0	0	0	0	1	0	0	0	0	0	0	0	1
Cluster21596	Acidobact	Acidobacteria_Gp1				0	1	0	0	0	0	0	0	0	0	0	0	1
Cluster21604	Proteobac	Gamm	Xantl	Xanthor	Lysobacter	0	0	0	0	0	0	1	0	0	0	0	0	1
Cluster21613	Actinobac	Actinol	Actinomycetales			0	0	1	0	0	0	0	0	0	0	0	0	1
Cluster21615;size=1						0	0	0	0	1	0	0	0	0	0	0	0	1
Cluster21617	OP11				OP11_genera_	0	0	0	0	0	1	0	0	0	0	0	0	1
Cluster21621	Proteobac	Alphap	Rhizc	Xanthol	Pseudolabrys	0	0	0	1	0	0	0	0	0	0	0	0	1
Cluster21631	Proteobac	Alphap	Caulc	Caulobacteraceae		0	0	0	0	1	0	0	0	0	0	0	0	1
Cluster21632	Proteobac	Alphap	Rhod	Acetobacteraceae		0	0	1	0	0	0	0	0	0	0	0	0	1
Cluster21640	Acidobact	Acidobacteria_Gp2			Gp2	0	0	0	0	0	0	0	0	0	0	1	0	1
Cluster21649	Proteobac	Alphap	Rhizc	Bradyrhizobiaceae		0	0	1	0	0	0	0	0	0	0	0	0	1
Cluster21653	Proteobac	Alphap	Rhod	Acetobacteraceae		0	0	0	1	0	0	0	0	0	0	0	0	1
Cluster21654	Acidobact	Acidobacteria_Gp1				0	0	0	1	0	0	0	0	0	0	0	0	1

Cluster21655	Proteobacteria					0	0	0	1	0	0	0	0	0	0	0	0	1
Cluster21663	Acidobact	Acidobacteria_Gp2			Gp2	0	0	1	0	0	0	0	0	0	0	0	0	1
Cluster21665	Verrucom	Opituta	Opitu	Opituta	Opitutus	0	0	0	0	0	1	0	0	0	0	0	0	1
Cluster21670;size=1						0	0	0	1	0	0	0	0	0	0	0	0	1
Cluster21672	Acidobact	Acidobacteria_Gp3			Gp3	0	0	0	0	1	0	0	0	0	0	0	0	1
Cluster21673	Proteobac	Gammaproteobacteria				0	0	0	0	0	0	0	0	0	0	1	0	1
Cluster21674	Actinobac	Actinol	Soliru	Conexib	Conexibacter	0	0	0	1	0	0	0	0	0	0	0	0	1
Cluster21677	Acidobact	Acidobacteria_Gp2			Gp2	0	0	0	1	0	0	0	0	0	0	0	0	1
Cluster21678;size=5						0	0	0	0	1	0	0	0	0	0	0	0	1
Cluster21680;size=2						0	0	0	0	1	0	0	0	0	0	0	0	1
Cluster21687	Acidobact	Acidobacteria_Gp10			Gp10	0	0	0	0	0	0	0	1	0	0	0	0	1
Cluster21689;size=1						0	0	0	0	0	0	0	0	0	0	1	0	1
Cluster21698;size=9						0	0	0	0	0	0	0	0	0	0	1	0	1
Cluster21701	Proteobac	Alphaproteobacteria				0	0	0	0	1	0	0	0	0	0	0	0	1
Cluster21705	Acidobact	Acidobacteria_Gp1				0	0	0	1	0	0	0	0	0	0	0	0	1
Cluster21713	Acidobact	Acidobacteria_Gp1				0	0	1	0	0	0	0	0	0	0	0	0	1
Cluster21715	Armatimo	Chthor	Chth	Chthon	Chthonomona	0	0	0	1	0	0	0	0	0	0	0	0	1
Cluster21717	Actinobac	Actinol	Actinomycetales			0	0	0	0	0	0	0	0	0	1	0	0	1
Cluster21719	Acidobact	Acidobacteria_Gp6			Gp6	0	0	0	1	0	0	0	0	0	0	0	0	1
Cluster21723	Proteobac	Deltaproteobacteria				1	0	0	0	0	0	0	0	0	0	0	0	1
Cluster21728	Bacteroid	Sphing	Sphir	Chitinophagaceae		0	0	0	1	0	0	0	0	0	0	0	0	1
Cluster21731	Acidobact	Acidobacteria_Gp4			Gp4	0	0	0	0	0	0	0	0	0	0	1	0	1
Cluster21735	Acidobact	Acidobacteria_Gp1				0	0	0	0	0	0	1	0	0	0	0	0	1
Cluster21737;size=2						0	0	0	0	0	0	0	0	0	0	1	0	1
Cluster21741	Chlamydia	Chlamy	Chlar	Parachlamydiaceae		0	0	0	0	0	0	0	0	0	0	1	0	1
Cluster21745	Proteobac	Gammaproteobacteria				0	0	1	0	0	0	0	0	0	0	0	0	1
Cluster21747	Chlamydia	Chlamy	Chlamydiales			0	0	0	1	0	0	0	0	0	0	0	0	1
Cluster21748	Proteobac	Betapr	Burkl	Oxaloba	Naxibacter	0	0	1	0	0	0	0	0	0	0	0	0	1
Cluster21750	Proteobac	Alphap	Rhod	Acetobacteraceae		0	0	0	1	0	0	0	0	0	0	0	0	1

Cluster21764	Acidobact	Acidobacteria_Gp3				0	0	0	0	0	0	0	0	0	0	1	0	1
Cluster21768;size=14						1	0	0	0	0	0	0	0	0	0	0	0	1
Cluster21772	Acidobact	Acidobacteria_Gp3				0	0	1	0	0	0	0	0	0	0	0	0	1
Cluster21774	Proteobacteria					0	0	0	1	0	0	0	0	0	0	0	0	1
Cluster21777	Proteobac	Alphap	Rhizc	Bradyrhizobiaceae		0	0	0	0	0	0	0	0	0	0	1	0	1
Cluster21782;size=2						0	0	0	0	0	0	0	0	0	0	0	1	1
Cluster21786	Acidobact	Acidobacteria_Gp3				0	0	0	0	0	0	0	0	0	0	1	0	1
Cluster21788	Proteobac	Alphap	Alphaproteob		Rhizomicrobiu	0	0	1	0	0	0	0	0	0	0	0	0	1
Cluster21792;size=7						0	0	0	1	0	0	0	0	0	0	0	0	1
Cluster21806	Acidobact	Acidobacteria_Gp1				0	0	0	1	0	0	0	0	0	0	0	0	1
Cluster21810	Proteobac	Gammaproteobacteria				0	0	0	0	1	0	0	0	0	0	0	0	1
Cluster21814	Chlamydia	Chlamy	Chlamydiales			0	0	0	0	1	0	0	0	0	0	0	0	1
Cluster21821	Proteobac	Alphap	Rhod	Rhodospirillaceae		0	0	0	1	0	0	0	0	0	0	0	0	1
Cluster21834;size=12						0	0	0	0	0	0	0	1	0	0	0	0	1
Cluster21835;size=1						0	0	0	0	0	0	0	0	0	0	1	0	1
Cluster21845	Acidobact	Acidobacteria_Gp10			Gp10	0	0	0	1	0	0	0	0	0	0	0	0	1
Cluster21847	Acidobact	Acidobacteria_Gp2			Gp2	0	0	0	0	0	0	0	0	0	0	0	1	1
Cluster21852	Acidobact	Acidobacteria_Gp3				0	0	0	0	0	0	0	0	0	0	1	0	1
Cluster21855	Actinobac	Actinol	Solirı	Conexib	Conexibacter	0	0	0	1	0	0	0	0	0	0	0	0	1
Cluster21859	Acidobact	Acidobacteria_Gp3			Gp3	0	0	0	0	0	0	0	0	0	0	1	0	1
Cluster21860	Acidobact	Acidobacteria_Gp2			Gp2	0	0	0	1	0	0	0	0	0	0	0	0	1
Cluster21863	Proteobac	Alphap	Rhod	Rhodospirillaceae		0	0	0	1	0	0	0	0	0	0	0	0	1
Cluster21864;size=6						0	0	1	0	0	0	0	0	0	0	0	0	1
Cluster21868	Chlamydia	Chlamy	Chlar	Parachlamydiaceae		0	0	0	1	0	0	0	0	0	0	0	0	1
Cluster21875	Actinobac	Actinobacteria				0	0	0	0	0	0	0	0	0	0	1	0	1
Cluster21881	Proteobac	Gammaproteobacteria				0	0	0	1	0	0	0	0	0	0	0	0	1
Cluster21891	Acidobact	Acidobacteria_Gp6			Gp6	0	0	0	0	1	0	0	0	0	0	0	0	1
Cluster21902;size=5						0	0	0	0	0	0	0	0	0	0	1	0	1
Cluster21905	Acidobact	Acidobacteria_Gp2			Gp2	0	0	0	1	0	0	0	0	0	0	0	0	1

Cluster21908	Proteobac	Alphap	Alphaproteob		Rhizomicrobiu	0	0	0	0	0	0	0	1	0	0	0	0	1
Cluster21914	Proteobac	Alphap	Rhizo	Bradyrhizobiaceae		0	0	0	0	0	0	0	0	0	0	0	1	1
Cluster21920	Acidobact	Acidobacteria_Gp3				0	0	0	1	0	0	0	0	0	0	0	0	1
Cluster21926;size=1						0	0	0	1	0	0	0	0	0	0	0	0	1
Cluster21929	Acidobact	Acidobacteria_Gp2			Gp2	0	0	0	0	0	0	1	0	0	0	0	0	1
Cluster21937;size=3						0	0	1	0	0	0	0	0	0	0	0	0	1
Cluster21938	Actinobac	Actinob	Solirubrobacterales			0	0	0	0	0	0	0	0	0	0	1	0	1
Cluster21940	Actinobac	Actinob	Acidimicrobiales			0	0	0	1	0	0	0	0	0	0	0	0	1
Cluster21944	Acidobact	Acidobacteria_Gp2			Gp2	0	0	0	1	0	0	0	0	0	0	0	0	1
Cluster21952	Acidobact	Acidobacteria_Gp2			Gp2	0	0	1	0	0	0	0	0	0	0	0	0	1
Cluster21957	Proteobac	Gamm	Xanth	Sinobac	Steroidobacter	0	0	0	1	0	0	0	0	0	0	0	0	1
Cluster21960	Proteobac	Alphap	Rhizo	Bradyrhizobiaceae		0	0	0	1	0	0	0	0	0	0	0	0	1
Cluster21961	Proteobac	Gamm	Legio	Coxiella	Aquicella	0	0	0	1	0	0	0	0	0	0	0	0	1
Cluster21964;size=3						0	0	0	0	0	0	0	0	0	0	1	0	1
Cluster21967	Acidobact	Acidobacteria_Gp1			Granulicella	0	0	0	1	0	0	0	0	0	0	0	0	1
Cluster21972	Proteobac	Alphap	Alphaproteob		Rhizomicrobiu	0	0	0	0	0	0	0	0	0	0	1	0	1
Cluster21974	Actinobac	Actinob	Soliru	Conexib	Conexibacter	0	0	0	0	0	0	0	0	0	0	1	0	1
Cluster21978;size=3						0	0	0	0	0	0	0	0	0	0	1	0	1
Cluster21979	Proteobac	Gamm	Xanth	Sinobac	Steroidobacter	0	0	0	0	0	1	0	0	0	0	0	0	1
Cluster21980	Bacteroid	Bacteroidetes_incert			Ohtaekwangia	0	0	0	0	0	0	0	0	0	0	1	0	1
Cluster21981	Proteobac	Alphap	Alphaproteob		Rhizomicrobiu	0	0	0	0	0	0	0	1	0	0	0	0	1
Cluster21992	Acidobact	Acidobacteria_Gp1			Granulicella	0	0	0	0	0	0	0	1	0	0	0	0	1
Cluster21997	Proteobac	Alphap	Rhod	Rhodospirillaceae		0	0	0	0	0	0	1	0	0	0	0	0	1
Cluster22000	Chlamydia	Chlamy	Chlam	Parachlamydiaceae		0	0	0	1	0	0	0	0	0	0	0	0	1
Cluster22020	Chloroflex	Ktedor	Ktedo	Ktedono	Ktedonobacter	0	0	0	1	0	0	0	0	0	0	0	0	1
Cluster22030	Acidobact	Acidobacteria_Gp2			Gp2	0	0	0	0	0	0	0	0	0	0	0	1	1
Cluster22041	Actinobac	Actinob	Soliru	Conexib	Conexibacter	0	0	0	1	0	0	0	0	0	0	0	0	1
Cluster22047	Proteobac	Alphaproteobacteria				0	0	0	1	0	0	0	0	0	0	0	0	1
Cluster22055	Proteobac	Alphap	Caulo	Caulobac	Phenylobacter	0	0	0	0	0	0	0	0	0	0	1	0	1

Cluster22058	Acidobact	Acidobacteria_Gp1			Gp1	0	0	0	1	0	0	0	0	0	0	0	0	1
Cluster22059	Acidobact	Acidobacteria_Gp3			Gp3	0	0	0	0	0	0	0	0	0	0	1	0	1
Cluster22075	Acidobact	Acidobacteria_Gp3			Gp3	0	0	0	0	0	0	0	1	0	0	0	0	1
Cluster22083;size=3						0	0	0	0	0	0	1	0	0	0	0	0	1
Cluster22084	Chlamydia	Chlamy	Chlar	Parachlamydiaceae		0	0	0	0	0	1	0	0	0	0	0	0	1
Cluster22086	Proteobac	Betapr	Burkl	Oxaloba	Massilia	0	0	0	1	0	0	0	0	0	0	0	0	1
Cluster22087	Verrucom	Opituta	Opitu	Opituta	Opitutus	0	0	0	1	0	0	0	0	0	0	0	0	1
Cluster22109;size=1						0	0	0	1	0	0	0	0	0	0	0	0	1
Cluster22110	Proteobac	Alphap	Rhod	Acetobacteraceae		0	0	0	1	0	0	0	0	0	0	0	0	1
Cluster22111	Chlamydia	Chlamy	Chlamydiales			0	0	0	1	0	0	0	0	0	0	0	0	1
Cluster22113	Proteobac	Alphap	Alphaproteob		Rhizomicrobiu	0	0	0	1	0	0	0	0	0	0	0	0	1
Cluster22125	Acidobacteria					0	0	0	0	0	1	0	0	0	0	0	0	1
Cluster22127	Proteobac	Gammaproteobacteria				0	0	0	1	0	0	0	0	0	0	0	0	1
Cluster22133	Acidobact	Holoph	Holop	Holopha	Geothrix	0	1	0	0	0	0	0	0	0	0	0	0	1
Cluster22140	Acidobact	Acidobacteria_Gp2			Gp2	0	0	0	0	0	0	0	0	0	0	1	0	1
Cluster22144	Proteobac	Betapr	Burkl	Burkholderiaceae		0	0	0	1	0	0	0	0	0	0	0	0	1
Cluster22147	Proteobac	Alphaproteobacteria				0	0	0	1	0	0	0	0	0	0	0	0	1
Cluster22151	Proteobac	Gammaproteobacteria				0	0	0	1	0	0	0	0	0	0	0	0	1
Cluster22158;size=5						0	0	0	0	0	0	0	0	0	0	0	1	1
Cluster22159	Proteobac	Gamm	Legio	Coxiellaceae		0	1	0	0	0	0	0	0	0	0	0	0	1
Cluster22161	Proteobac	Alphap	Rhod	Acetobacteraceae		0	0	0	0	0	0	0	0	0	1	0	0	1
Cluster22181	Acidobact	Acidobacteria_Gp3			Gp3	0	0	0	0	0	0	1	0	0	0	0	0	1
Cluster22184;size=7						0	0	0	0	0	0	0	0	0	0	0	1	1
Cluster22188	Acidobacteria					0	0	1	0	0	0	0	0	0	0	0	0	1
Cluster22191	Acidobacteria					0	0	1	0	0	0	0	0	0	0	0	0	1
Cluster22204	Acidobact	Acidobacteria_Gp1				0	0	0	1	0	0	0	0	0	0	0	0	1
Cluster22205	Proteobac	Alphap	Caulo	Cauloba	Phenylobacter	0	0	1	0	0	0	0	0	0	0	0	0	1
Cluster22206	Proteobac	Alphap	Caulo	Caulobacteraceae		0	0	0	1	0	0	0	0	0	0	0	0	1
Cluster22213	Acidobact	Acidobacteria_Gp2			Gp2	0	0	0	0	0	0	0	0	1	0	0	0	1

Cluster22216;size=4					0	0	0	0	0	0	0	1	0	0	0	0	1
Cluster22221	Proteobac	Alphap	Rhod	Acetobacteraceae	0	0	1	0	0	0	0	0	0	0	0	0	1
Cluster22222	Proteobac	Betaproteobacteria			0	0	1	0	0	0	0	0	0	0	0	0	1
Cluster22229	Proteobac	Alphaproteobacteria			0	0	0	0	0	0	0	0	0	0	0	1	1
Cluster22237	Acidobact	Acidobacteria_Gp1		Gp1	0	1	0	0	0	0	0	0	0	0	0	0	1
Cluster22241	Proteobac	Betapr	Burkl	Burkholderiaceae	0	0	0	0	0	0	0	0	0	0	0	1	1
Cluster22242	Proteobac	Gammaproteobacteria			0	0	0	0	0	0	0	0	0	0	0	1	1
Cluster22245	Nitrospira	Nitrosp	Nitro	Nitrospi Nitrospira	0	1	0	0	0	0	0	0	0	0	0	0	1
Cluster22246;size=7					0	0	0	0	0	0	0	1	0	0	0	0	1
Cluster22252	Acidobact	Acidobacteria_Gp2		Gp2	0	0	0	0	0	0	0	0	0	0	0	1	1
Cluster22261	Acidobact	Acidobacteria_Gp2		Gp2	0	0	0	1	0	0	0	0	0	0	0	0	1
Cluster22270	Acidobact	Acidobacteria_Gp1			0	0	1	0	0	0	0	0	0	0	0	0	1
Cluster22272	Proteobac	Betapr	Burkl	Burkhol Paucimonas	0	0	0	0	0	1	0	0	0	0	0	0	1
Cluster22273	Acidobact	Acidobacteria_Gp1			0	0	1	0	0	0	0	0	0	0	0	0	1
Cluster22278	Proteobac	Alphap	Rhodospirillales		0	0	0	0	1	0	0	0	0	0	0	0	1
Cluster22279;size=2					0	0	0	0	0	0	0	0	0	0	0	1	1
Cluster22281	Proteobac	Alphap	Rhod	Acetobacteraceae	0	0	1	0	0	0	0	0	0	0	0	0	1
Cluster22282	Proteobac	Alphap	Rhizobiales		0	0	0	0	0	0	0	0	0	0	0	1	1
Cluster22286	Acidobact	Acidobacteria_Gp1		Gp1	0	0	1	0	0	0	0	0	0	0	0	0	1
Cluster22287	Acidobact	Acidobacteria_Gp3		Bryobacter	0	0	0	1	0	0	0	0	0	0	0	0	1
Cluster22296	Acidobact	Acidobacteria_Gp1			0	0	0	0	0	0	0	0	1	0	0	0	1
Cluster22301;size=1					0	0	1	0	0	0	0	0	0	0	0	0	1
Cluster22304;size=3					0	0	0	0	0	0	0	0	0	0	0	1	1
Cluster22305	Acidobact	Acidobacteria_Gp2		Gp2	0	0	1	0	0	0	0	0	0	0	0	0	1
Cluster22309	Acidobact	Acidobacteria_Gp2		Gp2	0	0	0	0	0	0	1	0	0	0	0	0	1
Cluster22312	Proteobac	Alphap	Rhizc	Bradyrhizobiaceae	0	0	0	0	0	0	0	0	0	0	0	1	1
Cluster22315	Actinobac	Actinol	Acidi	Acidimi Aciditerrimona	0	1	0	0	0	0	0	0	0	0	0	0	1
Cluster22318	Proteobac	Betapr	Burkl	Burkhol Burkholderia	0	0	0	0	0	0	0	0	0	0	0	1	1
Cluster22322	Acidobact	Acidobacteria_Gp1			0	0	0	0	0	0	1	0	0	0	0	0	1

Cluster22324	Proteobac	Gammaproteobacteria				0	0	0	0	0	0	0	0	0	0	0	1	1
Cluster22325	Acidobact	Acidobacteria_Gp1				0	0	0	0	1	0	0	0	0	0	0	0	1
Cluster22328	Proteobac	Alphap	Alphaproteob		Rhizomicrobiu	0	1	0	0	0	0	0	0	0	0	0	0	1
Cluster22338	Verrucom	Opitut	Punic	Puniceicoccaceae		0	0	0	0	0	0	0	0	0	0	0	1	1
Cluster22348	Proteobac	Gammaproteobacteria				0	1	0	0	0	0	0	0	0	0	0	0	1
Cluster22354	Proteobac	Gamm	Enter	Enterobacteriaceae		0	0	0	0	0	0	0	0	0	0	0	1	1
Cluster22356	Proteobac	Gammaproteobacteria				0	1	0	0	0	0	0	0	0	0	0	0	1
Cluster22360	Chlamydia	Chlamy	Chlamydiales			0	1	0	0	0	0	0	0	0	0	0	0	1
Cluster22362	Acidobact	Acidobacteria_Gp7			Gp7	0	0	0	0	0	0	0	0	0	0	0	1	1
Cluster22364	Chlamydia	Chlamy	Chlar	Parachlamydiaceae		0	0	0	0	0	0	0	0	0	0	0	1	1
Cluster22365	Proteobac	Gammaproteobacteria				0	0	1	0	0	0	0	0	0	0	0	0	1
Cluster22371	Proteobac	Betapr	Burkl	Burkholderiaceae		0	0	1	0	0	0	0	0	0	0	0	0	1
Cluster22384	Proteobac	Gamm	Xantl	Sinobac	Steroidobacter	0	0	0	0	0	0	1	0	0	0	0	0	1
Cluster22401	Proteobac	Gamm	Xantl	Sinobac	Steroidobacter	0	0	0	0	0	0	0	0	0	0	0	1	1
Cluster22410	Bacteroid	Sphing	Sphir	Chitinophagaceae		0	0	0	0	0	0	0	0	0	0	0	1	1
Cluster22433	Proteobac	Alphap	Caulc	Cauloba	Phenylobacter	0	0	0	0	0	0	0	0	0	0	0	1	1
Cluster22434	Acidobact	Acidobacteria_Gp3			Gp3	1	0	0	0	0	0	0	0	0	0	0	0	1
Cluster22440	Proteobac	Gammaproteobacteria				0	0	0	0	0	0	0	0	0	0	0	1	1
Cluster22451	Proteobac	Alphaproteobacteria				0	0	0	0	0	0	0	0	0	0	1	0	1
Cluster22457	Proteobac	Gammaproteobacteria				0	0	0	0	0	0	0	1	0	0	0	0	1
Cluster22458	Verrucom	Subdivision3			Subdivision3_g	0	0	1	0	0	0	0	0	0	0	0	0	1
Cluster22462	Acidobact	Acidobacteria_Gp1				0	0	1	0	0	0	0	0	0	0	0	0	1
Cluster22467	Proteobac	Alphap	Rhizc	Bradyrhizobiaceae		0	0	1	0	0	0	0	0	0	0	0	0	1
Cluster22468	Proteobac	Gammaproteobacteria				0	0	1	0	0	0	0	0	0	0	0	0	1
Cluster22476	Acidobact	Acidobacteria_Gp2			Gp2	0	0	0	0	0	0	0	1	0	0	0	0	1
Cluster22482	Proteobac	Alphap	Caulc	Cauloba	Phenylobacter	0	0	1	0	0	0	0	0	0	0	0	0	1
Cluster22486	Proteobac	Deltap	Myxc	Polyangiaceae		0	0	1	0	0	0	0	0	0	0	0	0	1
Cluster22487	Acidobact	Acidobacteria_Gp1				0	0	0	0	0	0	0	0	0	0	0	1	1
Cluster22489	Proteobac	Alphaproteobacteria				0	0	0	0	0	0	0	0	0	0	0	1	1

Cluster22490	Chlamydia	Chlamy	Chlar	Parachlamydiaceae		0	0	1	0	0	0	0	0	0	0	0	0	1
Cluster22494	Acidobact	Acidobacteria_Gp1				0	0	1	0	0	0	0	0	0	0	0	0	1
Cluster22501	Verrucom	Opituta	Opitu	Opituta	Opitutus	0	0	0	0	0	0	0	0	0	0	0	1	1
Cluster22503;size=1						0	0	0	0	0	0	0	0	0	0	0	1	1
Cluster22504	Proteobac	Alphap	Alphaproteob		Rhizomicrobiu	0	0	0	0	0	0	0	0	0	0	0	1	1
Cluster22507	Acidobact	Acidobacteria_Gp1				0	0	0	0	0	0	1	0	0	0	0	0	1
Cluster22513	Acidobact	Acidobacteria_Gp2			Gp2	0	0	1	0	0	0	0	0	0	0	0	0	1
Cluster22535	Proteobac	Alphap	Rhodospirillales			0	0	0	0	0	0	0	1	0	0	0	0	1
Cluster22538	Acidobact	Acidobacteria_Gp2			Gp2	0	0	0	0	1	0	0	0	0	0	0	0	1
Cluster22539	Acidobact	Acidobacteria_Gp1			Gp1	0	0	0	0	0	0	0	0	0	0	0	1	1
Cluster22543	Acidobact	Acidobacteria_Gp2			Gp2	0	0	0	0	0	0	1	0	0	0	0	0	1
Cluster22559	Verrucom	Subdivision3			Subdivision3_g	0	0	0	0	0	0	0	0	0	0	0	1	1
Cluster22564	Acidobact	Acidobacteria_Gp1				0	0	0	0	0	0	1	0	0	0	0	0	1
Cluster22573	Chlamydia	Chlamy	Chlar	Parachla	Parachlamydia	0	0	0	0	0	0	0	1	0	0	0	0	1
Cluster22580	Proteobac	Gamm	Xanth	Sinobac	Steroidobacter	0	0	0	0	0	0	0	0	0	0	0	1	1
Cluster22586	Acidobact	Acidobacteria_Gp3			Gp3	0	0	0	0	0	0	0	0	0	0	0	1	1
Cluster22587	Chlamydia	Chlamy	Chlar	Parachla	Parachlamydia	0	1	0	0	0	0	0	0	0	0	0	0	1
Cluster22590	Proteobac	Alphap	Rhodospirillales			0	1	0	0	0	0	0	0	0	0	0	0	1
Cluster22592	Actinobac	Actinob	Actinomycetales			0	0	0	0	0	0	0	0	0	0	0	1	1
Cluster22593	Verrucom	Opituta	Opitu	Opituta	Opitutus	0	0	0	0	0	0	0	0	0	0	0	1	1
Cluster22595	Actinobac	Actinobacteria				0	0	0	0	0	1	0	0	0	0	0	0	1
Cluster22598	Acidobact	Acidobacteria_Gp3			Gp3	0	0	0	0	0	0	0	1	0	0	0	0	1
Cluster22599	Proteobac	Deltap	Myxo	Kofleria	Kofleria	0	1	0	0	0	0	0	0	0	0	0	0	1
Cluster22601	Verrucom	Opituta	Opitu	Opituta	Opitutus	1	0	0	0	0	0	0	0	0	0	0	0	1
Cluster22602	Acidobact	Acidobacteria_Gp1				1	0	0	0	0	0	0	0	0	0	0	0	1
Cluster22603	Acidobact	Acidobacteria_Gp2			Gp2	1	0	0	0	0	0	0	0	0	0	0	0	1
Cluster22604	Acidobact	Acidobacteria_Gp2			Gp2	1	0	0	0	0	0	0	0	0	0	0	0	1
Cluster22607	Proteobac	Alphap	Alphaproteob		Rhizomicrobiu	1	0	0	0	0	0	0	0	0	0	0	0	1
Cluster22609	Acidobact	Holoph	Holop	Holophagaceae		1	0	0	0	0	0	0	0	0	0	0	0	1

Cluster22613	Acidobact	Acidobacteria_Gp1				1	0	0	0	0	0	0	0	0	0	0	0	1
Cluster22614	Proteobacteria					1	0	0	0	0	0	0	0	0	0	0	0	1
Cluster22615	Acidobact	Acidobacteria_Gp2			Gp2	1	0	0	0	0	0	0	0	0	0	0	0	1
Cluster22616	Proteobac	Alphap	Rhizo	Methylo	Microvirga	0	0	0	0	0	0	0	0	0	0	0	1	1
Cluster22617	Acidobact	Acidobacteria_Gp1				1	0	0	0	0	0	0	0	0	0	0	0	1
Cluster22618	Verrucom	Opituta	Opitu	Opituta	Opitutus	1	0	0	0	0	0	0	0	0	0	0	0	1
Cluster22619	Proteobac	Gammaproteobacteria				1	0	0	0	0	0	0	0	0	0	0	0	1
Cluster22621	Proteobac	Betapr	Burkl	Comamonadaceae		1	0	0	0	0	0	0	0	0	0	0	0	1
Cluster22622	Actinobac	Actinol	Soliru	Conexib	Conexibacter	1	0	0	0	0	0	0	0	0	0	0	0	1
Cluster22623	Proteobac	Gammaproteobacteria				1	0	0	0	0	0	0	0	0	0	0	0	1
Cluster22625	Proteobac	Alphap	Alphaproteob		Rhizomicrobiu	0	0	0	0	0	0	0	0	0	0	0	1	1
Cluster22627	Acidobact	Acidobacteria_Gp1				0	0	0	0	0	0	0	0	0	0	1	0	1
Cluster22628	Acidobact	Acidobacteria_Gp3				1	0	0	0	0	0	0	0	0	0	0	0	1
Cluster22629	Chloroflex	Ktedor	Ktedonobacterales			0	0	0	0	0	0	0	0	0	0	0	1	1
Cluster22630;size=1						0	0	0	0	0	0	0	0	0	0	0	1	1
Cluster22632	Acidobact	Acidobacteria_Gp3			Gp3	1	0	0	0	0	0	0	0	0	0	0	0	1
Cluster22633	Acidobact	Acidobacteria_Gp1			Gp1	1	0	0	0	0	0	0	0	0	0	0	0	1
Cluster22634	Acidobact	Acidobacteria_Gp2			Gp2	1	0	0	0	0	0	0	0	0	0	0	0	1
Cluster22635	Chlamydia	Chlamy	Chlar	Parachla	Parachlamydia	1	0	0	0	0	0	0	0	0	0	0	0	1
Cluster22636	Proteobac	Alphap	Alphaproteob		Rhizomicrobiu	1	0	0	0	0	0	0	0	0	0	0	0	1
Cluster22639	Verrucom	Opituta	Opitu	Opituta	Opitutus	1	0	0	0	0	0	0	0	0	0	0	0	1
Cluster22641	Acidobact	Acidobacteria_Gp2			Gp2	1	0	0	0	0	0	0	0	0	0	0	0	1
Cluster22647	Proteobac	Alphap	Rhizobiales			0	0	1	0	0	0	0	0	0	0	0	0	1
Cluster22648	Acidobact	Acidobacteria_Gp2			Gp2	1	0	0	0	0	0	0	0	0	0	0	0	1
Cluster22651	Acidobact	Acidobacteria_Gp1			Gp1	0	0	0	0	0	1	0	0	0	0	0	0	1
Cluster22655	Acidobact	Acidobacteria_Gp1			Gp1	1	0	0	0	0	0	0	0	0	0	0	0	1
Cluster22659	Verrucom	Opituta	Opitu	Opituta	Opitutus	1	0	0	0	0	0	0	0	0	0	0	0	1
Cluster22660	Proteobac	Gammaproteobacteria				1	0	0	0	0	0	0	0	0	0	0	0	1
Cluster22661	Proteobac	Gamm	Xantl	Xanthomonadaceae		1	0	0	0	0	0	0	0	0	0	0	0	1

Cluster22664	Proteobac	Alphap	Rhizobiales			1	0	0	0	0	0	0	0	0	0	0	0	1
Cluster22665;size=1						1	0	0	0	0	0	0	0	0	0	0	0	1
Cluster22666	Proteobacteria					1	0	0	0	0	0	0	0	0	0	0	0	1
Cluster22668	Acidobact	Acidobacteria_Gp10			Gp10	1	0	0	0	0	0	0	0	0	0	0	0	1
Cluster22671	Acidobact	Acidobacteria_Gp3			Gp3	0	1	0	0	0	0	0	0	0	0	0	0	1
Cluster22680	Acidobact	Acidobacteria_Gp3			Gp3	0	0	0	0	1	0	0	0	0	0	0	0	1
Cluster22682	Proteobac	Gammaproteobacteria				0	0	0	0	0	0	0	0	0	0	0	1	1
Cluster22683	Actinobac	Actinobacteria				0	1	0	0	0	0	0	0	0	0	0	0	1
Cluster22684	Chlamydia	Chlamy	Chlar	Parachlamydiaceae		0	1	0	0	0	0	0	0	0	0	0	0	1
Cluster22693;size=1						0	0	0	0	0	0	0	0	0	0	0	1	1
Cluster22695	Acidobact	Acidobacteria_Gp2			Gp2	0	1	0	0	0	0	0	0	0	0	0	0	1
Cluster22696	Chlamydia	Chlamy	Chlar	Parachlamydiaceae		0	0	0	0	0	0	0	0	0	0	0	1	1
Cluster22704	Chloroflex	Ktedor	Ktedo	Thermo	Thermosporot	0	1	0	0	0	0	0	0	0	0	0	0	1
Cluster22705	Acidobact	Acidobacteria_Gp5			Gp5	0	0	0	0	1	0	0	0	0	0	0	0	1
Cluster22717	Acidobact	Acidobacteria_Gp10			Gp10	1	0	0	0	0	0	0	0	0	0	0	0	1
Cluster22719	Proteobac	Alphap	Rhodospirillales			0	1	0	0	0	0	0	0	0	0	0	0	1
Cluster22722	Proteobac	Gammaproteobacteria				0	0	0	0	0	0	0	0	0	0	0	1	1
Cluster22726;size=2						0	0	0	0	0	0	0	0	0	0	0	1	1
Cluster22733	Bacteroid	Sphing	Sphir	Chitinophagaceae		0	0	0	0	0	0	0	0	0	0	0	1	1
Cluster22742	Verrucom	Opitut	Opitu	Opituta	Opitutus	0	1	0	0	0	0	0	0	0	0	0	0	1
Cluster22748	Actinobac	Actinobacteria				0	0	0	0	0	0	0	1	0	0	0	0	1
Cluster22753	Proteobac	Gammaproteobacteria				0	1	0	0	0	0	0	0	0	0	0	0	1
Cluster22756	Proteobac	Gammaproteobacteria				0	0	0	0	0	0	0	0	0	0	0	1	1
Cluster22760;size=1						0	0	0	0	0	0	0	0	0	0	0	1	1
Cluster22764	Acidobact	Acidobacteria_Gp3				0	0	0	0	0	0	0	0	0	0	0	1	1
Cluster22769	Proteobac	Gammaproteobacteria				0	0	1	0	0	0	0	0	0	0	0	0	1
Cluster22783	Acidobact	Acidobacteria_Gp1			Granulicella	0	0	1	0	0	0	0	0	0	0	0	0	1
Cluster22784	Proteobac	Alphap	Rhod	Acetobacteraceae		0	0	1	0	0	0	0	0	0	0	0	0	1
Cluster22789;size=1						0	0	1	0	0	0	0	0	0	0	0	0	1

Cluster22790	Acidobact	Acidobacteria_Gp2			Gp2	0	0	0	0	0	0	0	1	0	0	0	0	1
Cluster22799	Proteobac	Alphap	Rhod	Rhodospirillaceae		0	0	1	0	0	0	0	0	0	0	0	0	1
Cluster22805	Acidobact	Acidobacteria_Gp6			Gp6	0	0	1	0	0	0	0	0	0	0	0	0	1
Cluster22817	Acidobact	Acidobacteria_Gp2			Gp2	0	0	0	0	0	0	0	0	0	1	0	0	1
Cluster22820	Proteobac	Alphap	Rhod	Acetobacteraceae		0	0	1	0	0	0	0	0	0	0	0	0	1
Cluster22823	Acidobact	Acidobacteria_Gp1				0	0	1	0	0	0	0	0	0	0	0	0	1
Cluster22824	Proteobac	Betapr	Burkholderiales			0	0	1	0	0	0	0	0	0	0	0	0	1
Cluster22830	Proteobac	Alphap	Caulo	Caulobacteraceae		0	0	0	0	0	0	0	0	0	1	0	0	1
Cluster22835	Proteobac	Gamm	Xanth	Sinobacteraceae		0	0	0	0	0	0	0	0	0	1	0	0	1
Cluster22836	Proteobac	Alphap	Rhod	Acetobacteraceae		0	0	1	0	0	0	0	0	0	0	0	0	1
Cluster22838	Proteobac	Alphap	Caulo	Caulobacteraceae		0	0	0	0	0	0	0	0	0	0	1	0	1
Cluster22839	Chlamydia	Chlamy	Chlamydiales			0	0	1	0	0	0	0	0	0	0	0	0	1
Cluster22841;size=1						0	0	1	0	0	0	0	0	0	0	0	0	1
Cluster22850	Proteobac	Alphaproteobacteria				0	0	1	0	0	0	0	0	0	0	0	0	1
Cluster22852	Proteobac	Alphap	Rhod	Acetobacteraceae		0	0	1	0	0	0	0	0	0	0	0	0	1
Cluster22857	Acidobact	Acidobacteria_Gp2			Gp2	0	0	0	0	0	0	0	0	0	0	1	0	1
Cluster22858	Actinobac	Actinobacteria				0	0	0	0	0	0	0	0	0	0	1	0	1
Cluster22879	Acidobact	Acidobacteria_Gp1			Gp1	0	0	0	0	0	0	0	0	0	0	1	0	1
Cluster22887	Acidobact	Acidobacteria_Gp3				0	0	0	1	0	0	0	0	0	0	0	0	1
Cluster22893	Acidobact	Acidobacteria_Gp1				0	0	0	0	0	0	0	0	0	0	1	0	1
Cluster22894	Acidobact	Acidobacteria_Gp2			Gp2	0	0	0	1	0	0	0	0	0	0	0	0	1
Cluster22896	Proteobac	Alphap	Rhizobiales			0	0	0	0	0	0	0	0	0	1	0	0	1
Cluster22901	Bacteroidetes					0	0	0	0	0	0	0	0	0	0	1	0	1
Cluster22903	Proteobac	Betaproteobacteria				0	0	0	1	0	0	0	0	0	0	0	0	1
Cluster22913	Acidobact	Acidobacteria_Gp1				0	0	0	0	0	0	0	1	0	0	0	0	1
Cluster22914	Acidobact	Acidobacteria_Gp2			Gp2	0	0	0	0	0	0	1	0	0	0	0	0	1
Cluster22915	Chlamydia	Chlamy	Chlamydiales			0	0	0	0	0	0	0	0	1	0	0	0	1
Cluster22916	Acidobact	Acidobacteria_Gp1			Gp1	0	0	1	0	0	0	0	0	0	0	0	0	1
Cluster22921	Acidobact	Acidobacteria_Gp2			Gp2	0	0	0	1	0	0	0	0	0	0	0	0	1

Cluster22923	Chlamydia	Chlamy	Chlar	Parachlamydiaceae		0	0	0	0	0	0	0	0	0	0	1	0	1
Cluster22926	Bacteroid	Sphing	Sphir	Chitinophagaceae		0	0	0	1	0	0	0	0	0	0	0	0	1
Cluster22935	Actinobac	Actinobacteria				0	0	0	1	0	0	0	0	0	0	0	0	1
Cluster22939	Acidobact	Acidobacteria_Gp5			Gp5	0	0	0	1	0	0	0	0	0	0	0	0	1
Cluster22940;size=2						0	0	0	0	0	0	0	0	0	0	1	0	1
Cluster22948	Acidobact	Acidobacteria_Gp1				0	0	0	0	0	0	0	0	0	0	1	0	1
Cluster22949	Proteobac	Gammaproteobacteria				0	0	0	0	0	1	0	0	0	0	0	0	1
Cluster22950	Acidobact	Acidobacteria_Gp1			Gp1	0	0	1	0	0	0	0	0	0	0	0	0	1
Cluster22955	Acidobact	Acidobacteria_Gp2			Gp2	0	0	1	0	0	0	0	0	0	0	0	0	1
Cluster22963;size=2						0	0	1	0	0	0	0	0	0	0	0	0	1
Cluster22967	Actinobac	Actinol	Actinomycetales			0	0	1	0	0	0	0	0	0	0	0	0	1
Cluster22982	Proteobac	Alphap	Rhizo	Bradyrh	Agromonas	0	0	0	0	0	0	0	0	0	0	1	0	1
Cluster22991;size=5						0	0	1	0	0	0	0	0	0	0	0	0	1
Cluster22995	Bacteroid	Sphing	Sphir	Chitinophagaceae		0	0	0	0	0	0	0	0	0	0	1	0	1
Cluster22998	Proteobac	Alphap	Caulo	Caulobacteraceae		0	0	1	0	0	0	0	0	0	0	0	0	1
Cluster23011	Acidobact	Acidobacteria_Gp2			Gp2	0	0	0	0	0	0	0	0	1	0	0	0	1
Cluster23018	Acidobact	Acidobacteria_Gp1				0	0	0	0	0	0	0	0	0	0	0	1	1
Cluster23028	Acidobact	Acidobacteria_Gp2			Gp2	0	0	1	0	0	0	0	0	0	0	0	0	1
Cluster23039;size=1						0	0	1	0	0	0	0	0	0	0	0	0	1
Cluster23045	Acidobact	Acidobacteria_Gp2			Gp2	0	0	0	0	0	0	0	0	0	0	0	1	1
Cluster23049	Proteobacteria					0	0	0	0	0	0	0	0	0	0	0	1	1
Cluster23051	Gemmatir	Gemm	Gemı	Gemma	Gemmatimona	0	0	0	0	0	0	0	0	0	0	0	1	1
Cluster23052;size=1						0	0	0	0	0	0	0	0	0	0	0	1	1
Cluster23055	Chloroflex	Ktedor	Ktedonobacterales			0	0	0	0	0	0	0	0	0	0	0	1	1
Cluster23062	Proteobac	Alphap	Rhod	Acetoba	Acidisphaera	0	0	1	0	0	0	0	0	0	0	0	0	1
Cluster23064	Proteobac	Gamm	Legio	Coxiella	Coxiella	0	0	0	0	0	0	0	1	0	0	0	0	1
Cluster23067	Proteobac	Gamm	Enter	Enterobacteriaceae		0	0	0	0	0	1	0	0	0	0	0	0	1
Cluster23068	Chlamydia	Chlamy	Chlar	Parachlamydiaceae		0	0	0	0	0	0	0	0	0	0	0	1	1
Cluster23075	Proteobacteria					0	0	0	0	1	0	0	0	0	0	0	0	1

Cluster23092	Proteobac	Gammaproteobacteria				0	0	1	0	0	0	0	0	0	0	0	0	1
Cluster23093	Proteobac	Alphap	Rhod	Acetobacteraceae		0	0	0	0	0	0	0	0	0	0	0	1	1
Cluster23095	Acidobact	Acidobacteria_Gp1				0	0	1	0	0	0	0	0	0	0	0	0	1
Cluster23096;size=1						0	0	1	0	0	0	0	0	0	0	0	0	1
Cluster23098	Proteobac	Gammaproteobacteria				0	0	1	0	0	0	0	0	0	0	0	0	1
Cluster23101	Acidobact	Acidobacteria_Gp1			Gp1	0	0	0	0	0	0	0	0	0	0	0	1	1
Cluster23104	Proteobac	Betapr	Burkl	Burkhol	Burkholderia	0	0	1	0	0	0	0	0	0	0	0	0	1
Cluster23110	Acidobact	Acidobacteria_Gp1			Granulicella	0	0	1	0	0	0	0	0	0	0	0	0	1
Cluster23111	Actinobac	Actinol	Solirubrobacterales			0	0	1	0	0	0	0	0	0	0	0	0	1
Cluster23116	Proteobac	Alphap	Rhizobiales			0	0	1	0	0	0	0	0	0	0	0	0	1
Cluster23122	Actinobac	Actinol	Acidimicrobiales			0	0	1	0	0	0	0	0	0	0	0	0	1
Cluster23124	Acidobact	Acidobacteria_Gp2			Gp2	0	0	1	0	0	0	0	0	0	0	0	0	1
Cluster23125	Proteobac	Gammaproteobacteria				0	0	0	0	0	0	0	1	0	0	0	0	1
Cluster23129	Proteobac	Alphap	Rhizobiales			0	0	1	0	0	0	0	0	0	0	0	0	1
Cluster23134;size=7						1	0	0	0	0	0	0	0	0	0	0	0	1
Cluster23136;size=1						0	0	1	0	0	0	0	0	0	0	0	0	1
Cluster23143	Proteobac	Alphap	Caulc	Cauloba	Asticcacaulis	0	0	1	0	0	0	0	0	0	0	0	0	1
Cluster23147	Proteobac	Alphap	Rhodospirillales			0	0	1	0	0	0	0	0	0	0	0	0	1
Cluster23155	Acidobacteria					0	0	0	0	0	0	0	0	0	0	0	1	1
Cluster23161	Actinobac	Actinol	Solirı	Conexib	Conexibacter	0	0	0	0	0	0	0	0	0	0	0	1	1
Cluster23163	Proteobac	Alphap	Rhizc	Bradyrhizobiaceae		0	0	1	0	0	0	0	0	0	0	0	0	1
Cluster23166	Acidobact	Acidobacteria_Gp1				0	0	1	0	0	0	0	0	0	0	0	0	1
Cluster23171	Actinobac	Actinol	Actinomycetales			0	0	1	0	0	0	0	0	0	0	0	0	1
Cluster23175	Acidobact	Acidobacteria_Gp3			Gp3	0	0	0	0	0	0	0	0	0	0	0	1	1
Cluster23177	Actinobac	Actinol	Acidi	Acidimi	Aciditerrimona	0	0	0	0	1	0	0	0	0	0	0	0	1
Cluster23179	Proteobac	Alphap	Rhod	Acetobacteraceae		0	0	0	0	1	0	0	0	0	0	0	0	1
Cluster23181	Elusimicro	Elusim	Elusi	Elusimic	Elusimicrobiun	0	0	0	0	0	0	0	0	0	0	0	1	1
Cluster23195;size=2						0	0	0	0	0	0	1	0	0	0	0	0	1
Cluster23206	Proteobac	Betapr	Burkl	Burkhol	Burkholderia	0	0	1	0	0	0	0	0	0	0	0	0	1

Cluster23208	Chlamydia	Chlamy	Chlar	Parachla	Parachlamydia	0	0	1	0	0	0	0	0	0	0	0	0	1
Cluster23217	Proteobac	Deltaproteobacteria				0	0	0	0	0	0	0	0	0	0	1	0	1
Cluster23225	Actinobac	Actinobacteria				0	0	1	0	0	0	0	0	0	0	0	0	1
Cluster23228	Actinobac	Actinol	Solirι	Conexib	Conexibacter	0	0	0	0	0	0	0	0	0	0	1	0	1
Cluster23232	Acidobact	Acidobacteria_Gp3			Bryobacter	0	0	1	0	0	0	0	0	0	0	0	0	1
Cluster23233	Proteobac	Gammaproteobacteria				0	0	1	0	0	0	0	0	0	0	0	0	1
Cluster23240	Proteobac	Alphap	Rhizc	Methylc	Methylocystis	0	0	0	0	0	0	0	0	0	0	1	0	1
Cluster23258	Acidobact	Acidobacteria_Gp3			Gp3	0	0	0	0	0	0	1	0	0	0	0	0	1
Cluster23261	Proteobac	Gammaproteobacteria				0	0	0	0	0	0	0	0	0	0	0	1	1
Cluster23264	Actinobac	Actinol	Acidimicrobiales			0	0	1	0	0	0	0	0	0	0	0	0	1
Cluster23266	Proteobacteria					0	0	0	0	0	1	0	0	0	0	0	0	1
Cluster23279	Acidobact	Acidobacteria_Gp3				0	0	1	0	0	0	0	0	0	0	0	0	1
Cluster23287	Acidobact	Acidobacteria_Gp2			Gp2	0	0	1	0	0	0	0	0	0	0	0	0	1
Cluster23289	Proteobac	Gammaproteobacteria				0	0	1	0	0	0	0	0	0	0	0	0	1
Cluster23290;size=2						0	0	0	0	0	0	0	0	0	0	1	0	1
Cluster23293	Chlamydia	Chlamy	Chlar	Parachlamydiaceae		0	0	0	0	0	0	1	0	0	0	0	0	1
Cluster23304	Verrucom	Opituta	Opitι	Opituta	Opitutus	0	0	0	0	0	0	0	0	1	0	0	0	1
Cluster23314;size=4						0	0	0	0	0	0	0	1	0	0	0	0	1
Cluster23317	Acidobacteria					0	0	0	0	1	0	0	0	0	0	0	0	1
Cluster23319	Chlamydia	Chlamy	Chlar	Parachlamydiaceae		0	0	0	0	1	0	0	0	0	0	0	0	1
Cluster23325	Proteobac	Gammaproteobacteria				0	0	0	0	0	0	1	0	0	0	0	0	1
Cluster23330;size=19						0	0	0	0	0	0	1	0	0	0	0	0	1
Cluster23332	Proteobac	Gamm	Xantl	Sinobac	Steroidobacter	0	0	0	0	0	1	0	0	0	0	0	0	1
Cluster23334	Bacteroid	Sphing	Sphir	Chitinophagaceae		0	0	0	0	0	1	0	0	0	0	0	0	1
Cluster23337	Actinobac	Actinol	Actin	Actinos	Actinospica	0	0	0	0	1	0	0	0	0	0	0	0	1
Cluster23338	Acidobact	Acidobacteria_Gp3			Gp3	0	0	0	0	0	1	0	0	0	0	0	0	1
Cluster23339	Planctomy	Plancto	Planc	Plancto	Zavarzinella	0	0	0	0	0	0	1	0	0	0	0	0	1
Cluster23340	Acidobact	Acidobacteria_Gp3			Gp3	0	0	0	0	0	1	0	0	0	0	0	0	1
Cluster23353	Chlamydia	Chlamy	Chlar	Parachlamydiaceae		0	0	0	0	0	0	0	0	1	0	0	0	1

Cluster23356	Proteobac	Gammaproteobacteria				0	0	0	0	0	1	0	0	0	0	0	0	1
Cluster23365	Acidobact	Acidobacteria_Gp3			Gp3	0	0	0	0	1	0	0	0	0	0	0	0	1
Cluster23367	Proteobac	Alphap	Caulo	Cauloba	Caulobacter	0	0	0	1	0	0	0	0	0	0	0	0	1
Cluster23371	Proteobac	Gammaproteobacteria				0	0	0	0	0	0	0	0	0	0	0	1	1
Cluster23375	Planctomy	Plancto	Planc	Plancto	Planctomyces	0	0	0	0	0	0	1	0	0	0	0	0	1
Cluster23388	Chlamydia	Chlamy	Chlam	Parachla	Parachlamydia	0	0	0	0	0	1	0	0	0	0	0	0	1
Cluster23390	Verrucom	Opituta	Opitu	Opituta	Opitutus	1	0	0	0	0	0	0	0	0	0	0	0	1
Cluster23393	Actinobac	Actinob	Soliru	Conexib	Conexibacter	0	0	0	1	0	0	0	0	0	0	0	0	1
Cluster23395	Acidobact	Acidobacteria_Gp6			Gp6	0	0	0	0	0	0	0	1	0	0	0	0	1
Cluster23398	Acidobacteria					0	0	0	0	0	0	1	0	0	0	0	0	1
Cluster23400	Proteobac	Alphap	Caulo	Caulobacteraceae		0	0	0	0	0	1	0	0	0	0	0	0	1
Cluster23402	Acidobacteria					0	0	0	0	0	0	0	1	0	0	0	0	1
Cluster23403	Bacteroid	Sphing	Sphin	Chitinophagaceae		0	0	0	0	0	0	1	0	0	0	0	0	1
Cluster23404	Acidobact	Acidobacteria_Gp2			Gp2	0	0	0	0	0	0	0	0	1	0	0	0	1
Cluster23407	Proteobac	Betapr	Burkl	Burkhol	Burkholderia	0	0	0	0	0	1	0	0	0	0	0	0	1
Cluster23411	Chlamydia	Chlamy	Chlam	Parachlamydiaceae		0	0	0	0	0	0	1	0	0	0	0	0	1
Cluster23416	Chlamydia	Chlamy	Chlam	Parachla	Parachlamydia	0	0	0	0	0	0	1	0	0	0	0	0	1
Cluster23422	Chlamydia	Chlamy	Chlam	Parachla	Neochlamydia	0	0	0	0	0	0	0	0	1	0	0	0	1
Cluster23433	Chloroflex	Ktedor	Ktedonobacterales			0	0	0	0	0	1	0	0	0	0	0	0	1
Cluster23436	Acidobact	Acidobacteria_Gp2			Gp2	0	0	0	1	0	0	0	0	0	0	0	0	1
Cluster23446	Proteobac	Deltap	Myxococcales			0	0	0	0	0	0	0	0	1	0	0	0	1
Cluster23453	Acidobact	Acidobacteria_Gp1			Gp1	0	0	0	0	0	0	1	0	0	0	0	0	1
Cluster23457	Acidobacteria					0	0	0	0	0	0	1	0	0	0	0	0	1
Cluster23458	Actinobac	Actinob	Soliru	Conexib	Conexibacter	0	0	0	0	0	1	0	0	0	0	0	0	1
Cluster23464;size=1						0	0	0	0	0	0	0	0	1	0	0	0	1
Cluster23468	Acidobact	Acidobacteria_Gp2			Gp2	0	0	0	0	0	0	0	0	1	0	0	0	1
Cluster23471	Proteobac	Alphap	Rhizo	Methylocystaceae		0	0	0	0	0	0	0	0	1	0	0	0	1
Cluster23480	Actinobac	Actinob	Actinomycetales			0	0	0	0	0	1	0	0	0	0	0	0	1
Cluster23481	Proteobac	Alphap	Rhizobiales			0	0	0	0	0	1	0	0	0	0	0	0	1

Cluster23482;size=2						0	0	0	0	1	0	0	0	0	0	0	0	1
Cluster23483	Acidobact	Acidobacteria_Gp2			Gp2	0	0	0	0	0	0	0	0	1	0	0	0	1
Cluster23493	Proteobacteria					0	0	0	0	0	0	1	0	0	0	0	0	1
Cluster23494	Acidobact	Acidobacteria_Gp2			Gp2	0	0	0	0	0	1	0	0	0	0	0	0	1
Cluster23498	Chlamydia	Chlamy	Chlamydiales			0	0	0	0	0	0	0	0	0	1	0	0	1
Cluster23499	Proteobac	Gammaproteobacteria				0	0	0	0	0	0	1	0	0	0	0	0	1
Cluster23506;size=1						0	0	0	0	0	1	0	0	0	0	0	0	1
Cluster23512	Acidobact	Acidobacteria_Gp3			Gp3	0	0	0	0	0	0	1	0	0	0	0	0	1
Cluster23513	Proteobac	Gamm	Xantl	Sinobac	Steroidobacter	0	0	0	0	0	1	0	0	0	0	0	0	1
Cluster23520;size=3						0	0	0	0	0	1	0	0	0	0	0	0	1
Cluster23537	Verrucom	Opituta	Opitu	Opituta	Opitutus	0	0	0	0	0	1	0	0	0	0	0	0	1
Cluster23543	Acidobact	Acidobacteria_Gp1				0	0	0	0	0	0	0	0	1	0	0	0	1
Cluster23545	Proteobac	Deltaproteobacteria				0	0	0	0	0	0	1	0	0	0	0	0	1
Cluster23548	Acidobact	Acidobacteria_Gp3				0	0	0	0	0	1	0	0	0	0	0	0	1
Cluster23550	Proteobac	Alphap	Sphingomonadales			0	0	0	0	0	1	0	0	0	0	0	0	1
Cluster23556	Proteobac	Alphap	Caulc	Cauloba	Phenylobacter	0	0	0	0	0	0	0	1	0	0	0	0	1
Cluster23559	Proteobac	Betaproteobacteria				0	0	0	0	0	0	1	0	0	0	0	0	1
Cluster23563	Acidobact	Acidobacteria_Gp3			Gp3	0	0	0	0	0	1	0	0	0	0	0	0	1
Cluster23565	Chlamydia	Chlamy	Chlamydiales			0	0	0	0	0	1	0	0	0	0	0	0	1
Cluster23568	Proteobac	Alphap	Rhodospirillales			0	0	0	0	0	0	1	0	0	0	0	0	1
Cluster23569	Chlamydia	Chlamy	Chlamydiales			0	0	0	0	0	0	0	0	1	0	0	0	1
Cluster23573	Acidobact	Acidobacteria_Gp3			Gp3	0	0	0	0	1	0	0	0	0	0	0	0	1
Cluster23575	Proteobac	Alphap	Caulc	Caulobacteraceae		0	0	0	0	1	0	0	0	0	0	0	0	1
Cluster23576	Chlamydia	Chlamy	Chlar	Parachlamydiaceae		0	0	0	0	0	0	0	0	1	0	0	0	1
Cluster23578	Proteobac	Alphap	Alphaproteob		Rhizomicrobiu	0	0	0	0	0	0	0	1	0	0	0	0	1
Cluster23579	Armatimo	Chthor	Chth	Chthon	Chthonomona	0	0	0	0	0	0	0	1	0	0	0	0	1
Cluster23582	Gemmatir	Gemm	Gem	Gemma	Gemmatimona	0	0	0	0	0	1	0	0	0	0	0	0	1
Cluster23587	Acidobact	Acidobacteria_Gp3				0	0	0	0	0	1	0	0	0	0	0	0	1
Cluster23594	Proteobac	Alphap	Rhizc	Bradyrh	Nitrobacter	0	0	0	0	0	0	1	0	0	0	0	0	1

Cluster23598;size=3						0	0	0	0	0	1	0	0	0	0	0	0	1
Cluster23603	Proteobac	Gammaproteobacteria				0	0	0	0	0	1	0	0	0	0	0	0	1
Cluster23608	Acidobact	Acidobacteria_Gp1			Gp1	0	0	0	0	0	0	0	1	0	0	0	0	1
Cluster23615;size=2						0	0	0	0	0	0	0	1	0	0	0	0	1
Cluster23619	Proteobacteria					0	0	0	0	0	0	0	1	0	0	0	0	1
Cluster23627	Acidobact	Acidobacteria_Gp2			Gp2	0	0	0	0	0	0	0	1	0	0	0	0	1
Cluster23629	Proteobac	Alphap	Rhodospirillales			0	0	0	0	0	1	0	0	0	0	0	0	1
Cluster23630	Proteobac	Gamm	Pseu	Pseudo	Rhizobacter	0	0	0	0	0	0	0	1	0	0	0	0	1
Cluster23631	Proteobacteria					0	0	0	0	0	0	1	0	0	0	0	0	1
Cluster23643	Chlamydia	Chlamy	Chlar	Parachlamydiaceae		0	0	0	0	0	0	1	0	0	0	0	0	1
Cluster23645	Acidobact	Acidobacteria_Gp1			Gp1	0	0	0	0	0	1	0	0	0	0	0	0	1
Cluster23665	Acidobact	Acidobacteria_Gp1			Gp1	0	0	0	0	1	0	0	0	0	0	0	0	1
Cluster23682	Proteobac	Gamm	Legio	Coxiella	Aquicella	0	0	0	0	0	1	0	0	0	0	0	0	1
Cluster23683	Actinobac	Actinobacteria				0	0	0	0	0	1	0	0	0	0	0	0	1
Cluster23693	Proteobac	Alphap	Rhizo	Bradyrhizobiaceae		0	0	0	0	0	0	0	1	0	0	0	0	1
Cluster23700	Proteobac	Gamm	Pseu	Pseudo	Pseudomonas	0	0	0	0	0	1	0	0	0	0	0	0	1
Cluster23706	Acidobact	Acidobacteria_Gp3				0	0	0	0	0	1	0	0	0	0	0	0	1
Cluster23714	Acidobacteria					0	0	0	0	1	0	0	0	0	0	0	0	1
Cluster23719	Proteobac	Betapr	Burkl	Burkhol	Burkholderia	0	0	0	0	0	0	0	1	0	0	0	0	1
Cluster23728	Proteobac	Gamm	Legio	Coxiella	Aquicella	0	0	0	0	0	1	0	0	0	0	0	0	1
Cluster23739;size=1						0	0	0	0	0	0	1	0	0	0	0	0	1
Cluster23760	Acidobact	Acidobacteria_Gp1				0	0	0	0	0	0	0	0	1	0	0	0	1
Cluster23766	Acidobact	Acidobacteria_Gp1			Gp1	0	0	0	0	0	0	1	0	0	0	0	0	1
Cluster23768	Acidobact	Acidobacteria_Gp1			Gp1	0	0	0	0	0	0	0	1	0	0	0	0	1
Cluster23783	Proteobacteria					0	0	0	0	0	1	0	0	0	0	0	0	1
Cluster23785	Acidobact	Acidobacteria_Gp1			Gp1	0	0	0	0	0	0	0	1	0	0	0	0	1
Cluster23786	Acidobact	Acidobacteria_Gp1			Gp1	0	0	0	0	0	0	1	0	0	0	0	0	1
Cluster23790;size=4						0	0	0	0	0	0	0	0	0	0	1	0	1
Cluster23791	Acidobact	Acidobacteria_Gp1			Gp1	0	0	0	0	0	1	0	0	0	0	0	0	1

Cluster23812	Verrucom	Subdivision3			Subdivision3_g	0	0	0	0	0	0	1	0	0	0	0	0	1
Cluster23822	Acidobact	Acidobacteria_Gp1				0	0	0	0	1	0	0	0	0	0	0	0	1
Cluster23826	Proteobac	Gammaproteobacteria				0	0	0	0	0	0	1	0	0	0	0	0	1
Cluster23829	Proteobac	Alphaproteobacteria				0	0	0	0	0	0	1	0	0	0	0	0	1
Cluster23843	Proteobac	Alphap	Rhodospirillales			0	0	0	0	1	0	0	0	0	0	0	0	1
Cluster23862	Proteobac	Gamm	Xantl	Sinobac	Nevskia	0	0	0	0	0	0	1	0	0	0	0	0	1
Cluster23869	Proteobac	Alphap	Rhizc	Bradyrhizobiaceae		0	0	0	0	0	0	0	1	0	0	0	0	1
Cluster23871	Chlamydia	Chlamy	Chlar	Parachlamydiaceae		1	0	0	0	0	0	0	0	0	0	0	0	1
Cluster23872;size=5						0	0	0	0	0	0	1	0	0	0	0	0	1
Cluster23874;size=3						0	0	0	0	0	0	0	0	1	0	0	0	1
Cluster23883	Proteobac	Gammaproteobacteria				1	0	0	0	0	0	0	0	0	0	0	0	1
Cluster23901	Acidobact	Acidobacteria_Gp1			Gp1	0	0	0	0	0	0	1	0	0	0	0	0	1
Cluster23902	Proteobac	Alphap	Rhodospirillales			0	0	0	0	0	0	0	1	0	0	0	0	1
Cluster23904	Chlamydia	Chlamy	Chlar	Parachla	Parachlamydia	0	0	0	0	0	0	1	0	0	0	0	0	1
Cluster23905;size=2						0	0	0	0	1	0	0	0	0	0	0	0	1
Cluster23923	Proteobac	Gammaproteobacteria				0	0	0	0	0	1	0	0	0	0	0	0	1
Cluster23930	Actinobac	Actinobacteria				0	0	0	0	0	0	1	0	0	0	0	0	1
Cluster23933	Proteobac	Alphaproteobacteria				0	0	0	0	0	1	0	0	0	0	0	0	1
Cluster23934;size=1						0	0	0	0	0	1	0	0	0	0	0	0	1
Cluster23935	Acidobact	Acidobacteria_Gp1				0	0	0	0	0	1	0	0	0	0	0	0	1
Cluster23942	Bacteroid	Sphing	Sphir	Sphingc	Pedobacter	0	0	0	0	0	0	1	0	0	0	0	0	1
Cluster23943	Acidobact	Acidobacteria_Gp1				0	0	0	1	0	0	0	0	0	0	0	0	1
Cluster23954;size=15						0	0	1	0	0	0	0	0	0	0	0	0	1
Cluster23956	Proteobac	Alphaproteobacteria				0	0	0	0	0	0	1	0	0	0	0	0	1
Cluster23966	Actinobac	Actinol	Actin	Actinos	Actinospica	0	0	0	0	0	0	0	0	1	0	0	0	1
Cluster23970	Proteobac	Gammaproteobacteria				0	0	0	0	0	1	0	0	0	0	0	0	1
Cluster23977	Actinobac	Actinol	Solirubrobacterales			0	0	0	0	0	0	0	0	0	1	0	0	1
Cluster23979	Actinobac	Actinol	Actin	Mycoba	Mycobacteriur	0	0	0	0	0	0	1	0	0	0	0	0	1
Cluster23980	Proteobac	Gamm	Xantl	Xanthomonadaceae		0	0	0	0	0	1	0	0	0	0	0	0	1

Cluster23986	Bacteroid	Sphing	Sphir	Sphingobacteriaceae		0	0	0	0	0	0	1	0	0	0	0	0	1
Cluster24001	Proteobac	Gamm	Xanth	Sinobac	Steroidobacter	1	0	0	0	0	0	0	0	0	0	0	0	1
Cluster24012	Chlamydia	Chlamy	Chlam	Parachlamydiaceae		0	0	0	0	0	1	0	0	0	0	0	0	1
Cluster24014	Proteobac	Deltap	Myxococcales			0	0	0	0	0	1	0	0	0	0	0	0	1
Cluster24028	Proteobac	Gammaproteobacteria				0	0	0	0	0	1	0	0	0	0	0	0	1
Cluster24030	Proteobac	Alphap	Caulo	Caulobacteraceae		0	0	0	0	0	0	0	1	0	0	0	0	1
Cluster24036	Proteobacteria					0	0	0	0	0	0	1	0	0	0	0	0	1
Cluster24046	Acidobact	Acidobacteria_Gp2			Gp2	0	0	0	0	0	0	1	0	0	0	0	0	1
Cluster24048;size=1						0	0	0	0	0	0	0	0	1	0	0	0	1
Cluster24049	Proteobac	Gammaproteobacteria				0	0	0	0	1	0	0	0	0	0	0	0	1
Cluster24059	TM7				TM7_genera_i	0	0	0	0	0	0	0	1	0	0	0	0	1
Cluster24066	Chlamydia	Chlamy	Chlamydiales			0	0	0	0	0	0	0	0	1	0	0	0	1
Cluster24071	Proteobac	Alphaproteobacteria				0	0	0	0	0	0	0	1	0	0	0	0	1
Cluster24073	Proteobac	Alphap	Rhodospirillales			0	0	0	0	0	0	0	0	0	1	0	0	1
Cluster24078	Acidobact	Acidobacteria_Gp1				0	0	0	0	0	0	1	0	0	0	0	0	1
Cluster24080	Proteobac	Betapr	Burkh	Burkholderiaceae		0	0	0	0	0	0	0	1	0	0	0	0	1
Cluster24084	Acidobact	Acidobacteria_Gp1			Gp1	0	0	0	0	0	0	0	0	0	1	0	0	1
Cluster24088	Proteobac	Alphap	Rhod	Acetobacteraceae		0	0	0	0	1	0	0	0	0	0	0	0	1
Cluster24090	Armatimo	Chthon	Chtho	Chthono	Chthonomona	0	0	0	0	0	0	0	1	0	0	0	0	1
Cluster24101	Proteobac	Alphaproteobacteria				0	0	0	0	0	0	0	1	0	0	0	0	1
Cluster24106	Bacteroid	Sphing	Sphir	Chitinophagaceae		0	0	1	0	0	0	0	0	0	0	0	0	1
Cluster24108;size=1						0	0	0	0	0	0	0	0	0	1	0	0	1
Cluster24121	Proteobac	Alphap	Rhizobiales			0	0	0	0	0	0	0	0	0	1	0	0	1
Cluster24128	Acidobact	Acidobacteria_Gp1				0	0	0	0	1	0	0	0	0	0	0	0	1
Cluster24138	Proteobac	Gammaproteobacteria				0	0	0	0	1	0	0	0	0	0	0	0	1
Cluster24142	Proteobac	Gammaproteobacteria				0	0	0	0	1	0	0	0	0	0	0	0	1
Cluster24144	Proteobac	Gamm	Xanth	Sinobacteraceae		0	0	0	0	0	0	0	0	0	1	0	0	1
Cluster24146	Proteobac	Gammaproteobacteria				0	0	0	0	0	0	0	0	0	1	0	0	1
Cluster24148;size=3						0	0	0	0	0	0	0	1	0	0	0	0	1

Cluster24151;size=1						0	0	0	0	1	0	0	0	0	0	0	0	1
Cluster24153	Acidobact	Acidobacteria_Gp1				0	0	0	0	0	0	1	0	0	0	0	0	1
Cluster24155	Acidobact	Acidobacteria_Gp2			Gp2	0	0	0	0	0	0	0	0	0	0	0	1	1
Cluster24158	Proteobac	Alphap	Rhizc	Brucella	Crabtreella	0	0	0	0	0	0	0	1	0	0	0	0	1
Cluster24163	Proteobac	Alphap	Rhod	Acetobacteraceae		0	0	0	0	1	0	0	0	0	0	0	0	1
Cluster24165	Acidobacteria					0	0	0	0	1	0	0	0	0	0	0	0	1
Cluster24167	Proteobac	Alphaproteobacteria				0	0	0	0	1	0	0	0	0	0	0	0	1
Cluster24168	Proteobac	Gamm	Pseu	Pseudo	Rhizobacter	0	0	0	0	0	0	0	0	0	1	0	0	1
Cluster24170	Acidobact	Holoph	Holo	Holophagaceae		0	0	0	0	0	0	0	0	0	1	0	0	1
Cluster24173	Acidobact	Acidobacteria_Gp1			Edaphobacter	0	0	0	0	0	0	0	0	0	1	0	0	1
Cluster24174	Chlamydia	Chlamy	Chlar	Parachlamydiaceae		0	0	0	0	1	0	0	0	0	0	0	0	1
Cluster24179	Acidobact	Acidobacteria_Gp3			Gp3	0	0	0	0	0	0	1	0	0	0	0	0	1
Cluster24182	Proteobac	Deltap	Myxococcales			0	0	0	0	0	0	1	0	0	0	0	0	1
Cluster24184	Acidobact	Acidobacteria_Gp1				0	0	0	0	0	0	1	0	0	0	0	0	1
Cluster24185	Acidobacteria					0	0	0	0	0	0	1	0	0	0	0	0	1
Cluster24188	Proteobac	Alphaproteobacteria				0	0	0	0	1	0	0	0	0	0	0	0	1
Cluster24191;size=1						0	0	0	0	1	0	0	0	0	0	0	0	1
Cluster24196	Proteobac	Deltap	Myxococcales			0	0	0	0	1	0	0	0	0	0	0	0	1
Cluster24202	Acidobact	Acidobacteria_Gp3			Gp3	0	0	0	0	0	0	0	0	0	1	0	0	1
Cluster24208	Proteobac	Deltaproteobacteria				0	0	0	0	0	0	0	0	0	0	1	0	1
Cluster24209	Proteobac	Alphap	Rhod	Acetobacteraceae		0	0	0	0	1	0	0	0	0	0	0	0	1
Cluster24219	Proteobacteria					0	0	0	0	0	0	0	1	0	0	0	0	1
Cluster24220;size=1						0	0	0	0	1	0	0	0	0	0	0	0	1
Cluster24226	Proteobac	Alphap	Sphir	Sphingc	Novosphingob	0	0	0	0	0	0	1	0	0	0	0	0	1
Cluster24230	Proteobac	Gammaproteobacteria				0	0	0	0	0	0	0	1	0	0	0	0	1
Cluster24239	Acidobact	Acidobacteria_Gp3				0	0	0	0	0	0	0	1	0	0	0	0	1
Cluster24241	Acidobact	Holoph	Holo	Holophagaceae		0	0	0	0	1	0	0	0	0	0	0	0	1
Cluster24244	Proteobacteria					0	0	0	0	0	0	0	1	0	0	0	0	1
Cluster24245;size=3						0	0	0	0	0	0	0	0	0	0	1	0	1

Cluster24248	Acidobact	Acidobacteria_Gp2			Gp2	0	0	0	0	1	0	0	0	0	0	0	0	1
Cluster24254	Proteobac	Gamm	Legio	Coxiella	Aquicella	0	0	0	0	0	0	0	0	1	0	0	0	1
Cluster24256	Proteobac	Alphap	Rhizo	Xanthob	Pseudolabrys	0	0	0	0	0	0	0	0	0	0	1	0	1
Cluster24258	Acidobact	Acidobacteria_Gp2			Gp2	0	0	0	0	1	0	0	0	0	0	0	0	1
Cluster24262;size=1						0	0	0	0	1	0	0	0	0	0	0	0	1
Cluster24264	Proteobac	Alphap	Alphaproteob		Rhizomicrobiu	0	0	0	0	0	0	0	1	0	0	0	0	1
Cluster24265	Proteobac	Alphap	Caulo	Cauloba	Phenylobacter	0	0	0	0	1	0	0	0	0	0	0	0	1
Cluster24268	Acidobacteria					0	0	0	0	1	0	0	0	0	0	0	0	1
Cluster24274	Chlamydia	Chlamy	Chlam	Parachla	Parachlamydia	0	0	0	0	0	0	1	0	0	0	0	0	1
Cluster24275	Acidobact	Acidobacteria_Gp1				0	0	0	0	0	0	1	0	0	0	0	0	1
Cluster24277	Actinobac	Actinobacteria				0	0	0	0	1	0	0	0	0	0	0	0	1
Cluster24278	Actinobac	Actinob	Soliru	Conexib	Conexibacter	0	0	0	0	0	0	0	1	0	0	0	0	1
Cluster24280;size=1						0	0	0	0	0	0	0	1	0	0	0	0	1
Cluster24282	Proteobac	Alphap	Rhizo	Bradyrhizobiaceae		0	0	0	0	1	0	0	0	0	0	0	0	1
Cluster24288	Chloroflex	Ktedon	Ktedonobacterales			0	0	0	0	0	0	0	1	0	0	0	0	1
Cluster24298	Proteobac	Gammaproteobacteria				0	0	0	0	1	0	0	0	0	0	0	0	1
Cluster24299	Acidobact	Acidobacteria_Gp3			Gp3	0	0	0	0	1	0	0	0	0	0	0	0	1
Cluster24306	Actinobac	Actinob	Solirubrobacterales			0	0	0	0	0	0	0	1	0	0	0	0	1
Cluster24308;size=2						0	0	0	0	0	0	1	0	0	0	0	0	1
Cluster24310;size=1						0	0	0	0	1	0	0	0	0	0	0	0	1
Cluster24311	Proteobac	Alphap	Sphin	Sphingo	Sphingomonas	0	0	0	0	0	0	0	1	0	0	0	0	1
Cluster24316	Actinobac	Actinob	Actin	Actinosp	Actinospica	0	0	0	0	0	0	0	0	0	1	0	0	1
Cluster24320	Acidobact	Acidobacteria_Gp1			Acidobacteriu	0	0	0	0	0	0	0	0	0	1	0	0	1
Cluster24324	Proteobac	Betapr	Burkl	Comamonadaceae		0	0	0	0	0	0	1	0	0	0	0	0	1
Cluster24326	Acidobact	Acidobacteria_Gp1				0	0	0	0	1	0	0	0	0	0	0	0	1
Cluster24338;size=1						0	0	0	0	1	0	0	0	0	0	0	0	1
Cluster24339	Actinobac	Actinob	Actinomycetales			0	0	0	0	1	0	0	0	0	0	0	0	1
Cluster24346;size=1						0	0	0	0	0	0	1	0	0	0	0	0	1
Cluster24349	Bacteroid	Sphing	Sphin	Sphingo	Mucilaginibact	0	0	0	0	0	0	0	1	0	0	0	0	1

Cluster24354	Proteobac	Betapr	Burkl	Oxaloba	Duganella	0	0	0	1	0	0	0	0	0	0	0	0	1
Cluster24358	Acidobact	Acidobacteria_Gp10			Gp10	0	0	0	0	1	0	0	0	0	0	0	0	1
Cluster24366	Acidobact	Acidobacteria_Gp1				0	0	0	0	1	0	0	0	0	0	0	0	1
Cluster24371	Chlamydia	Chlamy	Chlar	Parachla	Parachlamydia	0	0	0	0	0	0	1	0	0	0	0	0	1
Cluster24373	Proteobac	Alphap	Caulc	Caulobacteraceae		0	0	0	0	0	0	0	1	0	0	0	0	1
Cluster24376	Acidobact	Acidobacteria_Gp2			Gp2	0	0	0	0	0	0	0	1	0	0	0	0	1
Cluster24378	Proteobac	Gammaproteobacteria				0	0	0	0	0	0	1	0	0	0	0	0	1
Cluster24381	Acidobact	Acidobacteria_Gp2			Gp2	0	0	0	0	0	0	0	0	0	1	0	0	1
Cluster24383	Proteobac	Alphaproteobacteria				0	0	0	0	0	0	0	1	0	0	0	0	1
Cluster24384;size=1						0	0	0	0	0	0	0	1	0	0	0	0	1
Cluster24386	Proteobac	Gamm	Xantl	Sinobacteraceae		0	0	0	0	0	0	0	1	0	0	0	0	1
Cluster24389	Proteobacteria					0	0	0	0	0	0	0	1	0	0	0	0	1
Cluster24391	Proteobac	Alphaproteobacteria				0	0	0	0	1	0	0	0	0	0	0	0	1
Cluster24393	Proteobac	Alphaproteobacteria				0	0	0	1	0	0	0	0	0	0	0	0	1
Cluster24395	Bacteroid	Sphing	Sphir	Chitinophagaceae		0	0	0	0	0	0	0	1	0	0	0	0	1
Cluster24398	Bacteroid	Sphing	Sphir	Chitinop	Lacibacter	0	0	0	0	0	0	1	0	0	0	0	0	1
Cluster24408	Verrucom	Spartobacteria			Spartobacteria	0	0	0	0	1	0	0	0	0	0	0	0	1
Cluster24410	Acidobact	Acidobacteria_Gp1			Gp1	0	0	0	0	0	0	0	1	0	0	0	0	1
Cluster24413	Chlamydia	Chlamy	Chlar	Parachlamydiaceae		0	0	0	0	0	0	1	0	0	0	0	0	1
Cluster24423	Acidobact	Acidobacteria_Gp1				0	0	0	0	0	0	0	0	0	0	1	0	1
Cluster24447	Acidobact	Acidobacteria_Gp2			Gp2	0	0	0	0	0	0	0	1	0	0	0	0	1
Cluster24459	Actinobac	Actinol	Actinomycetales			0	0	0	0	0	0	0	1	0	0	0	0	1
Cluster24475	Proteobac	Alphap	Alphaproteob		Rhizomicrobiu	0	0	0	0	0	0	0	1	0	0	0	0	1
Cluster24476;size=1						0	0	0	0	0	0	0	1	0	0	0	0	1
Cluster24488	Acidobact	Acidobacteria_Gp1			Gp1	0	1	0	0	0	0	0	0	0	0	0	0	1
Cluster24491	Acidobact	Acidobacteria_Gp2			Gp2	0	0	0	0	1	0	0	0	0	0	0	0	1
Cluster24496	Acidobact	Acidobacteria_Gp1				0	0	0	0	0	0	0	0	0	0	1	0	1
Cluster24498	Proteobacteria					0	0	0	0	0	0	0	0	1	0	0	0	1
Cluster24500	Acidobact	Acidobacteria_Gp1			Gp1	0	0	0	0	0	0	0	1	0	0	0	0	1

Cluster24502	Proteobac	Alphap	Rhodospirillales			0	0	0	0	0	0	0	1	0	0	0	0	1
Cluster24508	Acidobact	Acidobacteria_Gp1				0	0	0	0	1	0	0	0	0	0	0	0	1
Cluster24511	Proteobac	Gammaproteobacteria				0	0	0	0	0	0	0	1	0	0	0	0	1
Cluster24517	Acidobact	Acidobacteria_Gp2			Gp2	0	0	0	0	0	0	1	0	0	0	0	0	1
Cluster24521	Chlamydia	Chlamy	Chlar	Parachla	Parachlamydia	0	0	0	0	0	0	0	0	1	0	0	0	1
Cluster24528	Proteobac	Alphap	Alphaproteob		Rhizomicrobiu	0	0	0	1	0	0	0	0	0	0	0	0	1
Cluster24529	Acidobact	Acidobacteria_Gp2			Gp2	0	0	0	0	0	0	0	1	0	0	0	0	1
Cluster24531	Acidobacteria					0	0	0	0	1	0	0	0	0	0	0	0	1
Cluster24541	Proteobacteria					0	0	0	0	0	0	0	0	0	0	0	1	1
Cluster24542	Acidobacteria					0	0	0	0	1	0	0	0	0	0	0	0	1
Cluster24543	Acidobact	Acidobacteria_Gp3			Bryobacter	0	0	0	0	0	0	0	0	0	0	1	0	1
Cluster24552	Acidobact	Acidobacteria_Gp6			Gp6	0	0	0	0	1	0	0	0	0	0	0	0	1
Cluster24577	Proteobac	Gammaproteobacteria				0	0	0	0	0	0	0	0	0	0	1	0	1
Cluster24578	Acidobact	Acidobacteria_Gp2			Gp2	0	0	0	1	0	0	0	0	0	0	0	0	1
Cluster24581	Actinobac	Actinol	Actin	Mycoba	Mycobacteriur	0	0	0	0	0	0	0	0	0	0	1	0	1
Cluster24583	Chloroflex	Ktedor	Ktedo	Ktedonc	Ktedonobacter	0	0	0	0	0	0	1	0	0	0	0	0	1
Cluster24585	Bacteroid	Sphing	Sphir	Sphingo	Mucilaginibact	0	0	0	0	0	0	1	0	0	0	0	0	1
Cluster24587	Proteobac	Alphap	Rhizobiales			0	0	0	0	0	0	1	0	0	0	0	0	1
Cluster24589	Acidobact	Acidobacteria_Gp1			Gp1	0	1	0	0	0	0	0	0	0	0	0	0	1
Cluster24600	Proteobac	Alphap	Rhod	Rhodospirillaceae		0	0	0	0	0	0	1	0	0	0	0	0	1
Cluster24601	Acidobact	Acidobacteria_Gp3				0	1	0	0	0	0	0	0	0	0	0	0	1
Cluster24607	Chloroflex	Ktedor	Ktedonobacterales			0	0	1	0	0	0	0	0	0	0	0	0	1
Cluster24616	Chloroflex	Ktedor	Ktedonobacterales			0	1	0	0	0	0	0	0	0	0	0	0	1
Cluster24617	Proteobac	Gammaproteobacteria				0	0	0	0	0	0	0	1	0	0	0	0	1
Cluster24627	Actinobac	Actinol	Actin	Mycoba	Mycobacteriur	0	1	0	0	0	0	0	0	0	0	0	0	1
Cluster24628	Acidobact	Acidobacteria_Gp6			Gp6	0	0	0	0	0	1	0	0	0	0	0	0	1
Cluster24632	Actinobac	Actinol	Solirt	Conexib	Conexibacter	0	0	0	0	0	0	0	0	0	0	0	1	1
Cluster24636	Proteobac	Gamm	Pseu	Pseudoi	Rugamonas	0	0	1	0	0	0	0	0	0	0	0	0	1
Cluster24639	Firmicutes	Bacilli	Bacillales			0	0	0	0	0	0	1	0	0	0	0	0	1

Cluster24640	Proteobacteria					0	0	1	0	0	0	0	0	0	0	0	0	1
Cluster24643	Proteobac	Alphap	Alphaproteob		Rhizomicrobiu	0	0	0	0	0	0	0	1	0	0	0	0	1
Cluster24644	Proteobac	Gammaproteobacteria				0	0	1	0	0	0	0	0	0	0	0	0	1
Cluster24648	Proteobac	Alphap	Caulc	Caulobа	Caulobacter	0	0	0	0	0	0	0	0	0	0	1	0	1
Cluster24649;size=3						0	0	0	0	0	0	0	0	0	0	0	1	1
Cluster24652	Bacteroid	Sphing	Sphir	Sphingobacteriaceae		0	0	0	0	0	0	0	1	0	0	0	0	1
Cluster24662	Proteobac	Alphaproteobacteria				0	0	0	0	0	0	1	0	0	0	0	0	1
Cluster24663	Chloroflex	Ktedor	Ktedc	Ktedonc	Ktedonobacter	0	0	0	0	0	0	1	0	0	0	0	0	1
Cluster24673	Acidobact	Acidobacteria_Gp3			Gp3	0	0	1	0	0	0	0	0	0	0	0	0	1
Cluster24686	Acidobact	Acidobacteria_Gp2			Gp2	0	0	0	0	0	0	1	0	0	0	0	0	1
Cluster24688	Proteobac	Alphap	Rhizobiales			0	0	0	0	0	0	0	0	0	0	0	1	1
Cluster24695	Proteobac	Alphaproteobacteria				0	0	0	0	0	0	0	0	1	0	0	0	1
Cluster24697	Acidobact	Acidobacteria_Gp2			Gp2	0	0	1	0	0	0	0	0	0	0	0	0	1
Cluster24704	Actinobac	Actinol	Acidimicrobiales			0	0	0	0	0	0	1	0	0	0	0	0	1
Cluster24716	Bacteroid	Sphing	Sphir	Sphingc	Mucilaginibact	0	0	0	0	0	0	0	1	0	0	0	0	1
Cluster24721	Acidobact	Acidobacteria_Gp3			Gp3	0	0	0	1	0	0	0	0	0	0	0	0	1
Cluster24723	Acidobact	Acidobacteria_Gp2			Gp2	0	1	0	0	0	0	0	0	0	0	0	0	1
Cluster24724	Proteobac	Gammaproteobacteria				0	0	0	0	0	0	0	0	1	0	0	0	1
Cluster24728	Proteobac	Alphap	Rhod	Acetobacteraceae		0	0	1	0	0	0	0	0	0	0	0	0	1
Cluster24732	Proteobac	Gammaproteobacteria				0	0	0	0	0	0	0	0	0	0	1	0	1
Cluster24738	Acidobact	Acidobacteria_Gp2			Gp2	0	0	1	0	0	0	0	0	0	0	0	0	1
Cluster24742	Bacteroid	Sphing	Sphir	Sphingc	Sphingobacter	0	0	1	0	0	0	0	0	0	0	0	0	1
Cluster24751	Acidobact	Acidobacteria_Gp3			Bryobacter	0	0	1	0	0	0	0	0	0	0	0	0	1
Cluster24753	Chloroflex	Ktedor	Ktedc	Ktedonc	Ktedonobacter	0	0	0	0	0	0	0	0	0	0	0	1	1
Cluster24756	Proteobac	Gamm	Legio	Coxiella	Aquicella	0	0	1	0	0	0	0	0	0	0	0	0	1
Cluster24760	Proteobac	Gamm	Pseu	Pseudo	Pseudomonas	0	0	0	0	0	0	0	0	0	0	1	0	1
Cluster24761	Bacteroid	Sphing	Sphir	Chitinophagaceae		0	0	0	0	0	0	0	0	0	1	0	0	1
Cluster24768	Acidobact	Acidobacteria_Gp2			Gp2	0	1	0	0	0	0	0	0	0	0	0	0	1
Cluster24787;size=1						0	0	1	0	0	0	0	0	0	0	0	0	1

Cluster24793	Acidobact	Acidobacteria_Gp4			Gp4	0	0	0	0	0	0	0	0	1	0	0	0	1
Cluster24795;size=5						1	0	0	0	0	0	0	0	0	0	0	0	1
Cluster24820	Proteobac	Alphap	Alphaproteob		Rhizomicrobiu	0	0	0	1	0	0	0	0	0	0	0	0	1
Cluster24821	Acidobact	Acidobacteria_Gp13			Gp13	0	0	0	0	0	0	1	0	0	0	0	0	1
Cluster24830	Acidobact	Acidobacteria_Gp2			Gp2	0	0	0	0	1	0	0	0	0	0	0	0	1
Cluster24831	Proteobac	Gamm	Xanth	Sinobacteraceae		0	0	1	0	0	0	0	0	0	0	0	0	1
Cluster24834	Proteobac	Alphaproteobacteria				0	0	1	0	0	0	0	0	0	0	0	0	1
Cluster24837	Actinobac	Actinob	Actinomycetales			0	0	1	0	0	0	0	0	0	0	0	0	1
Cluster24839	Proteobac	Alphaproteobacteria				0	0	0	0	0	0	1	0	0	0	0	0	1
Cluster24843	Chloroflex	Ktedon	Ktedo	Ktedono	Ktedonobacter	0	0	0	0	0	0	0	0	0	0	0	1	1
Cluster24851	Actinobac	Actinob	Actinomycetales			0	0	0	0	0	0	0	0	0	0	1	0	1
Cluster24853	Proteobac	Gamm	Xanth	Sinobac	Steroidobacter	0	0	0	0	0	0	1	0	0	0	0	0	1
Cluster24856	Bacteroid	Sphing	Sphin	Sphingo	Mucilaginibact	0	0	0	0	0	0	0	1	0	0	0	0	1
Cluster24861	Acidobact	Acidobacteria_Gp1			Gp1	0	0	0	0	0	0	0	0	0	0	0	1	1
Cluster24865	Acidobact	Acidobacteria_Gp3			Gp3	0	0	0	0	0	0	0	1	0	0	0	0	1
Cluster24866	Actinobac	Actinob	Actinomycetales			0	0	0	0	0	0	0	0	0	0	0	1	1
Cluster24868	Proteobac	Alphap	Rhod	Acetobacteraceae		0	0	0	0	0	0	1	0	0	0	0	0	1
Cluster24876	Bacteroid	Sphing	Sphin	Chitinop	Terrimonas	0	0	0	0	0	0	0	0	0	0	1	0	1
Cluster24880	Bacteroid	Sphing	Sphin	Sphingo	Mucilaginibact	0	0	0	0	0	0	1	0	0	0	0	0	1
Cluster24895	Proteobac	Gamm	Xanth	Sinobacteraceae		0	0	0	0	0	0	0	1	0	0	0	0	1
Cluster24896	Bacteroid	Bacteroidetes_incert			Ohtaekwangia	0	0	1	0	0	0	0	0	0	0	0	0	1
Cluster24898	Acidobact	Acidobacteria_Gp3				0	0	0	0	0	0	0	1	0	0	0	0	1
Cluster24899;size=1						0	0	0	0	0	0	0	0	0	0	0	1	1
Cluster24903	Actinobac	Actinob	Actinomycetales			0	1	0	0	0	0	0	0	0	0	0	0	1
Cluster24908	Proteobac	Alphap	Rhizobiales			0	0	1	0	0	0	0	0	0	0	0	0	1
Cluster24931	Actinobac	Actinob	Actinomycetales			0	0	0	0	0	0	0	0	0	0	0	1	1
Cluster24934	Proteobac	Gamm	Legio	Coxiella	Aquicella	0	0	0	0	0	1	0	0	0	0	0	0	1
Cluster24942	Proteobac	Betaproteobacteria				0	0	1	0	0	0	0	0	0	0	0	0	1
Cluster24943	Proteobac	Deltap	Myxo	Polyangiaceae		0	0	0	0	0	0	0	0	0	0	1	0	1

Cluster2494	Proteobac	Alphaproteobacteria				0	0	0	0	0	0	0	0	0	1	0	0	1
Cluster24947;size=1						0	0	1	0	0	0	0	0	0	0	0	0	1
Cluster2495	Proteobac	Alphap	Alphaproteol		Rhizomicrobiu	0	0	0	0	0	0	0	0	0	1	0	0	1
Cluster2496	Proteobac	Gammaproteobacteria				0	0	1	0	0	0	0	0	0	0	0	0	1
Cluster2496	Proteobac	Gamm	Xantl	Sinobac	Steroidobacter	0	0	0	0	0	0	1	0	0	0	0	0	1
Cluster2497	Bacteroid	Sphing	Sphir	Chitinophagaceae		0	0	0	0	0	0	0	1	0	0	0	0	1
Cluster2497	Proteobac	Alphaproteobacteria				0	0	1	0	0	0	0	0	0	0	0	0	1
Cluster2497	Proteobac	Betapr	Burkl	Burkholderiales_incert		0	0	0	0	0	0	0	1	0	0	0	0	1
Cluster2498	Proteobac	Alphap	Caulo	Caulobа	Caulobacter	0	0	0	0	0	0	0	0	0	1	0	0	1
Cluster2498	Acidobact	Acidobacteria_Gp1				0	0	0	0	0	0	0	0	0	0	1	0	1
Cluster2499	Acidobact	Acidobacteria_Gp1			Granulicella	0	0	1	0	0	0	0	0	0	0	0	0	1
Cluster2501	Chloroflex	Ktedor	Ktedonobacterales			0	0	0	1	0	0	0	0	0	0	0	0	1
Cluster2501	Proteobac	Alphap	Caulo	Caulobа	Phenylobacter	0	1	0	0	0	0	0	0	0	0	0	0	1
Cluster2502	Bacteroid	Sphing	Sphir	Sphingo	Mucilaginibact	0	0	0	0	0	0	0	1	0	0	0	0	1
Cluster25024;size=2						0	0	0	0	0	1	0	0	0	0	0	0	1
Cluster2504	Acidobact	Acidobacteria_Gp2			Gp2	0	0	0	0	0	0	1	0	0	0	0	0	1
Cluster2504	Proteobac	Gamm	Pseu	Pseudoı	Pseudomonas	0	0	1	0	0	0	0	0	0	0	0	0	1
Cluster2504	Proteobacteria					0	0	0	0	1	0	0	0	0	0	0	0	1
Cluster2506	Actinobac	Actinol	Actinomycetales			0	0	1	0	0	0	0	0	0	0	0	0	1
Cluster2507	Acidobact	Acidobacteria_Gp1			Gp1	0	0	0	0	0	0	0	1	0	0	0	0	1
Cluster2509	Chloroflex	Ktedor	Kted	Ktedonc	Ktedonobacter	0	0	0	0	0	1	0	0	0	0	0	0	1
Cluster2509	Acidobact	Acidobacteria_Gp6			Gp6	0	0	1	0	0	0	0	0	0	0	0	0	1
Cluster2509	Proteobac	Betaproteobacteria				0	0	0	0	0	0	1	0	0	0	0	0	1
Cluster2509	Proteobac	Alphaproteobacteria				0	0	0	0	0	0	0	1	0	0	0	0	1
Cluster2510	Acidobact	Acidobacteria_Gp3				0	0	1	0	0	0	0	0	0	0	0	0	1
Cluster2510	Verrucom	Opitut	Opitu	Opituta	Opitutus	0	1	0	0	0	0	0	0	0	0	0	0	1
Cluster2511	Acidobact	Acidobacteria_Gp1				0	0	0	0	0	0	1	0	0	0	0	0	1
Cluster2511	Proteobac	Gammaproteobacteria				0	0	0	0	0	0	0	1	0	0	0	0	1
Cluster2511	Actinobac	Actinol	Actinomycetales			0	0	0	1	0	0	0	0	0	0	0	0	1

Cluster25124	Proteobac	Betaproteobacteria				0	1	0	0	0	0	0	0	0	0	0	0	1
Cluster25125	Acidobact	Acidobacteria_Gp3				0	0	0	0	0	0	1	0	0	0	0	0	1
Cluster25127	Proteobac	Gamm	Legio	Coxiella	Coxiella	0	0	0	0	0	1	0	0	0	0	0	0	1
Cluster25140	Verrucom	Opituta	Opitu	Opituta	Opitutus	0	0	0	0	0	0	0	0	0	0	0	1	1
Cluster25147	Bacteroid	Sphing	Sphir	Chitinop	Flavitalea	0	0	0	0	0	0	1	0	0	0	0	0	1
Cluster25149	Proteobac	Gamm	Legio	Coxiella	Aquicella	0	0	1	0	0	0	0	0	0	0	0	0	1
Cluster25152	Actinobac	Actinol	Actinomycetales			0	0	0	0	0	0	1	0	0	0	0	0	1
Cluster25155	Firmicutes	Bacilli	Bacill	Alicyclo	Alicyclobacillus	0	0	0	0	0	0	0	0	0	0	1	0	1
Cluster25161	Proteobac	Gammaproteobacteria				0	0	0	0	0	1	0	0	0	0	0	0	1
Cluster25175	Acidobacteria					0	0	0	0	0	0	0	0	0	0	0	1	1
Cluster25179	Acidobact	Acidobacteria_Gp1				0	0	0	0	0	0	0	0	0	0	0	1	1
Cluster25185	Acidobact	Acidobacteria_Gp2			Gp2	0	0	0	0	0	0	0	1	0	0	0	0	1
Cluster25188	Acidobact	Acidobacteria_Gp2			Gp2	0	0	0	0	0	0	0	0	0	0	0	1	1
Cluster25192;size=3						0	0	0	1	0	0	0	0	0	0	0	0	1
Cluster25202	Proteobac	Alphaproteobacteria				0	0	1	0	0	0	0	0	0	0	0	0	1
Cluster25204	Proteobac	Alphap	Rhizobiales			0	0	0	0	0	0	0	0	0	0	0	1	1
Cluster25205	Chloroflex	Ktedor	Ktedonobacterales			0	0	0	0	0	0	1	0	0	0	0	0	1
Cluster25216	Proteobac	Alphap	Caulc	Cauloba	Phenylobacter	0	0	0	0	0	0	0	1	0	0	0	0	1
Cluster25219	Acidobact	Acidobacteria_Gp2			Gp2	0	0	0	0	0	0	0	1	0	0	0	0	1
Cluster25231	Bacteroid	Sphing	Sphir	Chitinophagaceae		0	0	1	0	0	0	0	0	0	0	0	0	1
Cluster25233	Proteobac	Gammaproteobacteria				0	0	0	0	1	0	0	0	0	0	0	0	1
Cluster25237	Proteobac	Gamm	Xantl	Xanthomonadaceae		0	0	0	0	0	0	0	1	0	0	0	0	1
Cluster25241	Acidobact	Acidobacteria_Gp1			Gp1	0	0	0	0	0	0	0	0	0	0	0	1	1
Cluster25249	Proteobac	Gamm	Xantl	Sinobacteraceae		0	0	0	0	0	0	0	0	0	0	0	1	1
Cluster25251	Bacteroid	Sphing	Sphir	Sphingo	Sphingobacter	0	0	0	0	0	0	0	0	0	0	0	1	1
Cluster25270	Proteobac	Alphaproteobacteria				0	0	0	0	0	0	1	0	0	0	0	0	1
Cluster25272	Bacteroid	Sphing	Sphir	Sphingo	Mucilaginibact	1	0	0	0	0	0	0	0	0	0	0	0	1
Cluster25275	Proteobac	Gamm	Legio	Coxiella	Aquicella	0	0	0	0	0	0	1	0	0	0	0	0	1
Cluster25282	Proteobac	Alphap	Alphaproteob	Rhizomicrobiu		0	0	0	0	0	0	0	0	1	0	0	0	1

Cluster25286	Proteobac	Gammaproteobacteria				0	0	0	0	0	0	0	0	0	0	0	1	1
Cluster25288	Acidobact	Acidobacteria_Gp3				0	0	0	0	0	0	0	1	0	0	0	0	1
Cluster25289	Actinobac	Actinol	Acidi	Acidimi	Aciditerrimona	0	0	0	1	0	0	0	0	0	0	0	0	1
Cluster25290;size=2						0	0	0	0	0	0	1	0	0	0	0	0	1
Cluster25296	Proteobac	Alphap	Caulo	Cauloba	Caulobacter	0	0	1	0	0	0	0	0	0	0	0	0	1
Cluster25297	Proteobac	Betapr	Burkl	Burkhol	Burkholderia	0	0	0	0	0	0	0	0	0	0	0	1	1
Cluster25299	Proteobac	Alphap	Rhod	Acetoba	Acidisphaera	0	0	1	0	0	0	0	0	0	0	0	0	1
Cluster25300	Bacteroid	Sphing	Sphir	Sphingo	Pedobacter	0	0	0	0	0	0	0	1	0	0	0	0	1
Cluster25312	Proteobacteria					0	0	0	0	0	0	0	1	0	0	0	0	1
Cluster25317	Acidobact	Acidobacteria_Gp2			Gp2	0	0	0	0	0	0	0	1	0	0	0	0	1
Cluster25318	Acidobact	Acidobacteria_Gp3			Bryobacter	0	0	0	0	1	0	0	0	0	0	0	0	1
Cluster25325	Proteobac	Alphap	Alphaproteob		Rhizomicrobiu	0	0	0	0	0	0	0	1	0	0	0	0	1
Cluster25330	Proteobac	Gamm	Xantl	Sinobac	Steroidobacter	0	0	0	0	0	0	0	1	0	0	0	0	1
Cluster25333	Chloroflex	Ktedor	Ktedo	Ktedono	Ktedonobacter	0	0	0	0	0	0	0	0	0	0	0	1	1
Cluster25350	Acidobact	Acidobacteria_Gp13			Gp13	0	0	0	0	0	0	1	0	0	0	0	0	1
Cluster25351	Acidobact	Acidobacteria_Gp1				0	0	0	0	0	0	0	1	0	0	0	0	1
Cluster25360	Acidobact	Acidobacteria_Gp3			Gp3	0	0	0	0	0	1	0	0	0	0	0	0	1
Cluster25362	Bacteroid	Sphing	Sphir	Sphingo	Solitalea	0	0	0	1	0	0	0	0	0	0	0	0	1
Cluster25364	Chloroflex	Ktedor	Ktedonobacterales			0	0	0	0	0	0	0	0	1	0	0	0	1
Cluster25380	Proteobac	Alphaproteobacteria				0	0	0	0	0	0	0	1	0	0	0	0	1
Cluster25394	Chloroflex	Ktedor	Ktedonobacterales			0	0	0	0	0	0	0	0	0	0	1	0	1
Cluster25396	Acidobact	Acidobacteria_Gp17			Gp17	0	0	0	1	0	0	0	0	0	0	0	0	1
Cluster25397	Acidobact	Acidobacteria_Gp3				0	0	0	0	0	0	0	0	0	0	0	1	1
Cluster25410	Bacteroid	Sphing	Sphir	Sphingo	Pedobacter	0	0	0	0	1	0	0	0	0	0	0	0	1
Cluster25411	Acidobact	Acidobacteria_Gp2			Gp2	0	0	1	0	0	0	0	0	0	0	0	0	1
Cluster25412	Actinobac	Actinol	Actinomycetales			0	0	0	0	0	0	1	0	0	0	0	0	1
Cluster25417	Bacteroid	Bacteroidetes_incert			Ohtaekwangia	0	0	0	0	0	0	1	0	0	0	0	0	1
Cluster25423;size=1						0	0	0	0	0	0	0	1	0	0	0	0	1
Cluster25425	Proteobac	Alphaproteobacteria				0	0	0	0	0	0	0	1	0	0	0	0	1

Cluster2542ɛ	Proteobac	Alphap	Rhizc	Hyphon	Rhodoplanes	0	0	0	0	0	0	0	0	0	0	1	0	1
Cluster2543ż	Proteobac	Gamm	Legio	Legione	Legionella	0	1	0	0	0	0	0	0	0	0	0	0	1
Cluster2544ꜱ	Chloroflex	Ktedor	Ktedonobacterales			0	0	0	0	0	0	0	0	0	0	0	1	1
Cluster2545ż	Proteobac	Betapr	Burkholderiales			0	0	0	0	0	0	0	1	0	0	0	0	1
Cluster2545ᐩ	Proteobac	Gamm	Xantl	Sinobac	Steroidobacter	0	1	0	0	0	0	0	0	0	0	0	0	1
Cluster2546C	Proteobacteria					0	0	0	1	0	0	0	0	0	0	0	0	1
Cluster2548ꜱ	Acidobact	Acidobacteria_Gp3				0	0	1	0	0	0	0	0	0	0	0	0	1
Cluster25489;size=1						0	0	0	0	0	0	1	0	0	0	0	0	1
Cluster2549ϵ	Bacteroid	Sphing	Sphir	Sphingc	Mucilaginibact	0	0	0	0	0	0	0	0	0	0	0	1	1
Cluster2550ɛ	Bacteroid	Sphing	Sphir	Sphingobacteriaceae		0	0	0	0	0	0	0	1	0	0	0	0	1
Cluster25513;size=2						0	0	0	0	0	0	1	0	0	0	0	0	1
Cluster2551ꜱ	Proteobac	Gamm	Legio	Coxiella	Aquicella	0	0	0	0	0	0	1	0	0	0	0	0	1
Cluster2551ϵ	Verrucom	Opituta	Opitu	Opituta	Opitutus	0	0	0	0	0	0	1	0	0	0	0	0	1
Cluster25521	Bacteroid	Sphing	Sphir	Sphingc	Mucilaginibact	0	0	1	0	0	0	0	0	0	0	0	0	1
Cluster25526;size=1						0	1	0	0	0	0	0	0	0	0	0	0	1
Cluster2552ꜱ	Acidobact	Acidobacteria_Gp1				0	0	0	0	0	0	0	0	0	0	0	1	1
Cluster2553ɛ	Acidobacteria					0	0	0	0	0	0	0	0	0	1	0	0	1
Cluster2555C	Actinobac	Actinobacteria				0	0	1	0	0	0	0	0	0	0	0	0	1
Cluster2555ʔ	Proteobac	Deltap	Myxococcales			0	0	0	0	0	0	1	0	0	0	0	0	1
Cluster2555ɛ	Proteobac	Alphap	Rhod	Acetobacteraceae		0	0	1	0	0	0	0	0	0	0	0	0	1
Cluster25561	Actinobac	Actinol	Solirubrobacterales			0	0	0	0	0	0	1	0	0	0	0	0	1
Cluster2556ᐩ	Proteobac	Gamm	Legio	Coxiella	Aquicella	0	0	0	1	0	0	0	0	0	0	0	0	1
Cluster2557ż	Proteobac	Gammaproteobacteria				0	0	0	0	0	0	0	1	0	0	0	0	1
Cluster2557ꜱ	Acidobact	Acidobacteria_Gp1				0	0	1	0	0	0	0	0	0	0	0	0	1
Cluster2558ϵ	Acidobact	Acidobacteria_Gp3			Gp3	0	0	0	0	0	0	0	1	0	0	0	0	1
Cluster25591	Acidobact	Acidobacteria_Gp2			Gp2	0	0	0	1	0	0	0	0	0	0	0	0	1
Cluster2559ȝ	Bacteroid	Sphing	Sphir	Chitinoߵ	Sediminibacter	0	0	0	0	0	0	0	0	0	0	0	1	1
Cluster2560ż	Proteobac	Alphap	Rhod	Acetoba	Acidisphaera	0	0	0	0	0	0	1	0	0	0	0	0	1
Cluster2560ȝ	Proteobac	Alphap	Rhodospirillales			0	0	0	0	0	0	0	0	0	0	0	1	1

Cluster25613	Proteobac	Gammaproteobacteria				0	0	0	0	0	0	1	0	0	0	0	0	1
Cluster25614	Proteobac	Gammaproteobacteria				0	0	1	0	0	0	0	0	0	0	0	0	1
Cluster25617;size=1						0	0	1	0	0	0	0	0	0	0	0	0	1
Cluster25619	Proteobac	Alphap	Rhod	Acetobacteraceae		0	0	1	0	0	0	0	0	0	0	0	0	1
Cluster25620;size=2						0	0	1	0	0	0	0	0	0	0	0	0	1
Cluster25625	Proteobac	Betaproteobacteria				0	0	1	0	0	0	0	0	0	0	0	0	1
Cluster25630	Proteobacteria					0	0	0	0	0	0	0	0	0	0	0	1	1
Cluster25645;size=3						0	0	0	0	0	0	1	0	0	0	0	0	1
Cluster25646	Acidobact	Acidobacteria_Gp1				0	0	1	0	0	0	0	0	0	0	0	0	1
Cluster25655	Acidobact	Acidobacteria_Gp1				0	0	1	0	0	0	0	0	0	0	0	0	1
Cluster25667	Acidobact	Acidobacteria_Gp1			Acidobacteriur	0	0	1	0	0	0	0	0	0	0	0	0	1
Cluster25688	Proteobac	Alphap	Alphaproteob		Rhizomicrobiu	0	0	1	0	0	0	0	0	0	0	0	0	1
Cluster25700	Actinobac	Actinobacteria				0	0	0	1	0	0	0	0	0	0	0	0	1
Cluster25701	Proteobac	Alphap	Caulc	Caulobacteraceae		0	0	0	0	0	0	1	0	0	0	0	0	1
Cluster25715	Acidobact	Acidobacteria_Gp1			Gp1	0	0	0	0	0	0	0	0	1	0	0	0	1
Cluster25726	Acidobact	Acidobacteria_Gp3			Gp3	0	0	1	0	0	0	0	0	0	0	0	0	1
Cluster25739	Proteobac	Alphap	Caulc	Cauloba	Caulobacter	0	0	0	0	0	0	0	0	0	1	0	0	1
Cluster25748	Proteobac	Alphap	Rhodospirillales			0	0	1	0	0	0	0	0	0	0	0	0	1
Cluster25750	Chloroflex	Ktedor	Ktedc	Ktedonc	Ktedonobacter	0	0	0	0	0	0	0	0	0	0	0	1	1
Cluster25758	Proteobac	Alphap	Alphaproteob		Rhizomicrobiu	0	0	0	0	1	0	0	0	0	0	0	0	1
Cluster25759	Proteobac	Alphap	Alphaproteob		Rhizomicrobiu	0	0	0	0	1	0	0	0	0	0	0	0	1
Cluster25763	Acidobact	Acidobacteria_Gp1			Gp1	0	0	0	0	0	0	0	0	0	0	1	0	1
Cluster25767	Bacteroid	Sphing	Sphir	Chitinophagaceae		1	0	0	0	0	0	0	0	0	0	0	0	1
Cluster25771	Actinobac	Actinol	Solirt	Conexib	Conexibacter	0	0	0	1	0	0	0	0	0	0	0	0	1
Cluster25774	Proteobac	Betapr	Burkholderiales			0	0	0	0	0	0	0	0	0	1	0	0	1
Cluster25780	Bacteroidetes					0	0	0	0	0	0	0	0	0	1	0	0	1
Cluster25784	Actinobac	Actinol	Actin	Mycoba	Mycobacteriur	0	0	0	0	0	0	0	0	0	0	1	0	1
Cluster25789	Acidobact	Acidobacteria_Gp1				0	0	0	0	0	0	0	0	0	0	1	0	1
Cluster25790	Actinobac	Actinol	Actin	Catenul	Catenulispora	1	0	0	0	0	0	0	0	0	0	0	0	1

Cluster25793;size=1						0	0	0	0	1	0	0	0	0	0	0	0	1
Cluster25797	Acidobact	Acidobacteria_Gp3			Gp3	0	0	0	0	0	0	0	0	0	1	0	0	1
Cluster25800	Acidobact	Acidobacteria_Gp10			Gp10	0	0	0	0	0	0	0	0	0	0	0	1	1
Cluster25804	Proteobac	Alphap	Rhizobiales			0	0	0	0	0	0	0	0	0	0	1	0	1
Cluster25805	Actinobac	Actinol	Soliru	Conexib	Conexibacter	0	0	0	1	0	0	0	0	0	0	0	0	1
Cluster25808	Proteobacteria					0	0	0	0	1	0	0	0	0	0	0	0	1
Cluster25809	Actinobac	Actinol	Actin	Mycoba	Mycobacteriur	0	0	0	0	0	1	0	0	0	0	0	0	1
Cluster25816	Proteobac	Gamm	Legio	Coxiella	Aquicella	0	0	0	0	0	1	0	0	0	0	0	0	1
Cluster25830	Verrucom	Opituta	Opitu	Opituta	Opitutus	0	0	0	0	0	0	0	0	0	0	0	1	1
Cluster25831	Acidobact	Acidobacteria_Gp1			Gp1	0	0	0	0	0	0	0	0	0	0	1	0	1
Cluster25832	Proteobac	Alphap	Alphaproteob		Rhizomicrobiu	0	0	0	0	1	0	0	0	0	0	0	0	1
Cluster25840	Proteobac	Gamm	Legio	Coxiella	Aquicella	0	0	0	1	0	0	0	0	0	0	0	0	1
Cluster25844	Acidobact	Acidobacteria_Gp1			Gp1	0	0	0	1	0	0	0	0	0	0	0	0	1
Cluster25853	Acidobact	Acidobacteria_Gp1			Gp1	0	0	0	1	0	0	0	0	0	0	0	0	1
Cluster25856;size=1						0	0	0	1	0	0	0	0	0	0	0	0	1
Cluster25858	Proteobac	Alphap	Caulc	Cauloba	Phenylobacter	0	0	0	1	0	0	0	0	0	0	0	0	1
Cluster25868	Proteobac	Alphap	Rhizobiales			0	0	0	0	1	0	0	0	0	0	0	0	1
Cluster25873	Proteobac	Alphap	Rhizc	Methylc	Methylocystis	0	0	0	0	0	0	0	0	0	1	0	0	1
Cluster25874	Chlamydia	Chlamy	Chlar	Parachla	Parachlamydia	0	0	0	0	0	0	0	0	0	0	1	0	1
Cluster25879;size=2						0	0	0	1	0	0	0	0	0	0	0	0	1
Cluster25884	Bacteroid	Sphing	Sphir	Sphingc	Mucilaginibact	0	0	0	1	0	0	0	0	0	0	0	0	1
Cluster25887	Acidobact	Acidobacteria_Gp3			Gp3	0	0	0	1	0	0	0	0	0	0	0	0	1
Cluster25891	Proteobac	Gamm	Xantl	Sinobacteraceae		0	0	0	1	0	0	0	0	0	0	0	0	1
Cluster25897	Proteobac	Betapr	Burkl	Oxaloba	Janthinobacter	0	0	0	1	0	0	0	0	0	0	0	0	1
Cluster25898	Acidobact	Acidobacteria_Gp1				0	0	0	0	0	0	0	0	0	1	0	0	1
Cluster25899;size=1						0	0	0	0	0	1	0	0	0	0	0	0	1
Cluster25900	Proteobac	Alphap	Rhizc	Hyphomicrobiaceae		0	0	0	0	1	0	0	0	0	0	0	0	1
Cluster25904;size=2						0	0	0	1	0	0	0	0	0	0	0	0	1
Cluster25907	Proteobac	Betapr	Neiss	Neisseri	Iodobacter	1	0	0	0	0	0	0	0	0	0	0	0	1

Cluster25910	Proteobac	Gamm	Legio	Coxiella	Aquicella	0	0	0	0	0	0	0	0	1	0	0	0	1
Cluster25913	Acidobact	Acidobacteria_Gp3				0	0	0	1	0	0	0	0	0	0	0	0	1
Cluster25914	Acidobact	Acidobacteria_Gp5			Gp5	0	1	0	0	0	0	0	0	0	0	0	0	1
Cluster25920	Acidobact	Acidobacteria_Gp2			Gp2	0	1	0	0	0	0	0	0	0	0	0	0	1
Cluster25921	Acidobacteria					0	0	0	0	0	0	0	0	1	0	0	0	1
Cluster25936	Proteobac	Alphap	Caulo	Caulobа	Phenylobacter	0	0	0	0	0	0	0	0	0	0	1	0	1
Cluster25942	Actinobac	Actinol	Actinomycetales			0	0	0	1	0	0	0	0	0	0	0	0	1
Cluster25960	Gemmatir	Gemm	Gemı	Gemma	Gemmatimona	1	0	0	0	0	0	0	0	0	0	0	0	1
Cluster25964	Proteobac	Alphap	Caulo	Caulobа	Asticcacaulis	0	0	1	0	0	0	0	0	0	0	0	0	1
Cluster25967	Proteobac	Gamm	Legio	Coxiella	Aquicella	0	0	0	1	0	0	0	0	0	0	0	0	1
Cluster25970	Proteobac	Alphap	Rhod	Acetobacteraceae		0	0	1	0	0	0	0	0	0	0	0	0	1
Cluster25972	Proteobac	Gammaproteobacteria				0	0	0	0	0	0	0	0	0	1	0	0	1
Cluster25979	Proteobac	Deltaproteobacteria				0	0	0	0	1	0	0	0	0	0	0	0	1
Cluster25983	Bacteroid	Sphing	Sphir	Chitinoр	Ferruginibacte	0	0	0	1	0	0	0	0	0	0	0	0	1
Cluster25987	Proteobac	Betaproteobacteria				0	0	0	0	1	0	0	0	0	0	0	0	1
Cluster25988	Bacteroid	Sphing	Sphir	Chitinophagaceae		0	0	0	1	0	0	0	0	0	0	0	0	1
Cluster25991;size=7						0	0	0	0	0	0	0	0	1	0	0	0	1
Cluster26000	Bacteroid	Sphing	Sphir	Chitinoр	Sediminibacter	1	0	0	0	0	0	0	0	0	0	0	0	1
Cluster26001	Chloroflex	Ktedor	Ktedonobacterales			0	0	0	0	0	0	0	0	0	0	1	0	1
Cluster26010	Acidobact	Acidobacteria_Gp1				0	0	0	0	0	0	1	0	0	0	0	0	1
Cluster26015	Proteobac	Alphap	Rhod	Acetobacteraceae		0	0	0	0	0	0	0	0	0	0	1	0	1
Cluster26016	Proteobac	Alphaproteobacteria				0	0	0	1	0	0	0	0	0	0	0	0	1
Cluster26018	Proteobac	Gammaproteobacteria				1	0	0	0	0	0	0	0	0	0	0	0	1
Cluster26019	Proteobac	Alphap	Rhizobiales			0	0	0	1	0	0	0	0	0	0	0	0	1
Cluster26023	Proteobacteria					1	0	0	0	0	0	0	0	0	0	0	0	1
Cluster26027	Proteobac	Gamm	Pseu	Pseudoı	Pseudomonas	0	0	0	0	1	0	0	0	0	0	0	0	1
Cluster26029	Proteobac	Alphap	Rhizobiales			0	0	0	0	0	0	0	0	1	0	0	0	1
Cluster26030	Proteobac	Alphap	Rhizobiales			0	0	0	1	0	0	0	0	0	0	0	0	1
Cluster26031;size=1						0	0	0	0	0	0	0	0	0	0	1	0	1

Cluster26040	Proteobac	Deltap	Bdell	Bdellovi	Bdellovibrio	0	0	0	1	0	0	0	0	0	0	0	0	1
Cluster26041	Proteobac	Betapr	Burkl	Burkhol	Burkholderia	0	0	0	1	0	0	0	0	0	0	0	0	1
Cluster26048	Acidobact	Acidobacteria_Gp2			Gp2	0	0	0	0	1	0	0	0	0	0	0	0	1
Cluster26052	Acidobact	Acidobacteria_Gp2			Gp2	0	0	0	0	0	0	0	0	0	0	0	1	1
Cluster26054	Proteobac	Gamm	Xantl	Sinobac	Steroidobacter	0	0	0	0	0	0	0	0	0	0	0	1	1
Cluster26055	Acidobact	Acidobacteria_Gp2			Gp2	0	0	1	0	0	0	0	0	0	0	0	0	1
Cluster26063	Proteobac	Alphaproteobacteria				1	0	0	0	0	0	0	0	0	0	0	0	1
Cluster26072	Proteobac	Alphap	Rhizobiales			0	0	0	0	0	1	0	0	0	0	0	0	1
Cluster26083	Acidobact	Acidobacteria_Gp1				0	0	0	0	0	1	0	0	0	0	0	0	1
Cluster26089	Proteobac	Gamm	Enter	Enterob	Xenorhabdus	0	0	0	0	0	0	0	0	1	0	0	0	1
Cluster26093	Proteobac	Gamm	Legio	Coxiella	Aquicella	1	0	0	0	0	0	0	0	0	0	0	0	1
Cluster26094	Bacteroid	Sphing	Sphir	Sphingobacteriaceae		0	0	0	1	0	0	0	0	0	0	0	0	1
Cluster26101	Acidobact	Acidobacteria_Gp3			Gp3	0	0	0	0	0	0	0	0	0	0	1	0	1
Cluster26103	Chloroflex	Ktedor	Ktedonobacterales			0	0	0	0	0	0	0	0	0	0	0	1	1
Cluster26108	Proteobac	Gammaproteobacteria				0	0	0	0	0	1	0	0	0	0	0	0	1
Cluster26113	Bacteroid	Sphing	Sphir	Chitinop	Sediminibacter	0	0	0	0	0	0	0	0	0	0	1	0	1
Cluster26121	Proteobac	Betaproteobacteria				0	0	0	0	0	1	0	0	0	0	0	0	1
Cluster26123	Acidobact	Acidobacteria_Gp2			Gp2	0	0	0	0	1	0	0	0	0	0	0	0	1
Cluster26126	Bacteroid	Sphing	Sphir	Chitinophagaceae		0	0	0	1	0	0	0	0	0	0	0	0	1
Cluster26129	Proteobac	Alphap	Caulc	Cauloba	Phenylobacter	0	0	0	0	1	0	0	0	0	0	0	0	1
Cluster26130	Acidobact	Acidobacteria_Gp3			Gp3	0	0	0	1	0	0	0	0	0	0	0	0	1
Cluster26137	Verrucom	Opitut	Opitu	Opituta	Opitutus	0	0	0	0	0	1	0	0	0	0	0	0	1
Cluster26144	Acidobact	Acidobacteria_Gp3			Bryobacter	0	0	0	1	0	0	0	0	0	0	0	0	1
Cluster26145;size=2						0	0	0	1	0	0	0	0	0	0	0	0	1
Cluster26150	Planctomy	Plancto	Planc	Planctor	Singulisphaera	0	0	0	1	0	0	0	0	0	0	0	0	1
Cluster26154;size=3						0	0	0	0	0	0	0	0	1	0	0	0	1
Cluster26162	Actinobac	Actinol	Actin	Micromonosporaceae		0	0	0	0	0	0	0	0	1	0	0	0	1
Cluster26163	Actinobac	Actinol	Acidi	Acidimi	Aciditerrimona	0	0	0	0	0	0	0	0	0	0	1	0	1
Cluster26176	Proteobacteria					1	0	0	0	0	0	0	0	0	0	0	0	1

Cluster26177	Proteobac	Alphap	Caulo	Caulobacteraceae		1	0	0	0	0	0	0	0	0	0	0	0	1
Cluster26178	Acidobact	Acidobacteria_Gp1				0	0	0	0	0	0	0	0	0	0	1	0	1
Cluster26186	Verrucom	Opituta	Opitu	Opituta	Opitutus	1	0	0	0	0	0	0	0	0	0	0	0	1
Cluster26187	Verrucom	Spartobacteria			Spartobacteria	0	0	0	0	0	1	0	0	0	0	0	0	1
Cluster26188	Proteobac	Gammaproteobacteria				0	0	0	1	0	0	0	0	0	0	0	0	1
Cluster26189;size=2						0	0	0	0	0	0	0	0	0	0	1	0	1
Cluster26190	Acidobact	Acidobacteria_Gp3				0	0	0	0	0	0	0	0	0	1	0	0	1
Cluster26199	Actinobac	Actinol	Soliru	Conexib	Conexibacter	0	0	0	0	0	0	0	0	0	1	0	0	1
Cluster26208;size=1						0	0	0	0	0	0	0	0	0	1	0	0	1
Cluster26212	Proteobac	Gamm	Xanth	Sinobac	Steroidobacter	0	0	0	0	0	0	0	0	0	1	0	0	1
Cluster26213	Proteobac	Deltap	Bdell	Bdellovi	Bdellovibrio	0	0	0	0	0	0	1	0	0	0	0	0	1
Cluster26221	Chlamydia	Chlamy	Chlar	Parachlamydiaceae		0	0	0	0	0	0	0	0	0	1	0	0	1
Cluster26228	Bacteroid	Sphing	Sphir	Chitinop	Sediminibacter	0	0	0	0	0	0	0	0	0	0	0	1	1
Cluster26231;size=1						0	0	0	0	0	0	0	0	0	1	0	0	1
Cluster26237	Proteobac	Alphap	Caulo	Caulobacteraceae		0	0	0	0	0	0	0	0	0	0	1	0	1
Cluster26243	Proteobac	Alphaproteobacteria				0	0	0	0	1	0	0	0	0	0	0	0	1
Cluster26244;size=1						0	0	0	0	0	0	0	0	0	0	1	0	1
Cluster26246	Bacteroid	Sphing	Sphir	Chitinophagaceae		0	0	0	1	0	0	0	0	0	0	0	0	1
Cluster26248;size=2						0	0	0	0	0	0	0	0	0	0	0	1	1
Cluster26259	Proteobac	Alphap	Rhod	Acetoba	Acidisphaera	0	0	0	0	0	0	0	0	0	0	1	0	1
Cluster26260	Acidobact	Acidobacteria_Gp2			Gp2	0	0	0	1	0	0	0	0	0	0	0	0	1
Cluster26261	Proteobac	Betapr	Burkl	Burkhol	Ralstonia	0	0	0	0	0	0	0	0	0	0	0	1	1
Cluster26262;size=2						0	0	0	1	0	0	0	0	0	0	0	0	1
Cluster26263	Acidobact	Acidobacteria_Gp2			Gp2	0	0	0	0	1	0	0	0	0	0	0	0	1
Cluster26264	Actinobac	Actinobacteria				1	0	0	0	0	0	0	0	0	0	0	0	1
Cluster26270	Proteobac	Alphap	Alphaproteob		Rhizomicrobiu	0	0	0	0	0	0	0	0	0	1	0	0	1
Cluster26273	Actinobac	Actinol	Actinomycetales			0	0	0	1	0	0	0	0	0	0	0	0	1
Cluster26275	Acidobact	Acidobacteria_Gp6			Gp6	1	0	0	0	0	0	0	0	0	0	0	0	1
Cluster26283	Proteobac	Alphap	Caulo	Caulobacteraceae		0	0	0	1	0	0	0	0	0	0	0	0	1

Cluster26287	Proteobac	Alphap	Caulo	Cauloba	Phenylobacter	0	0	0	0	1	0	0	0	0	0	0	0	1
Cluster26289	Proteobac	Gamm	Legio	Coxiella	Aquicella	0	1	0	0	0	0	0	0	0	0	0	0	1
Cluster26296	Nitrospira	Nitrosp	Nitro	Nitrospi	Nitrospira	0	0	0	0	0	0	0	0	0	0	1	0	1
Cluster26301	Actinobac	Actinol	Actinomycetales			0	0	0	1	0	0	0	0	0	0	0	0	1
Cluster26314	Proteobac	Alphap	Rhizo	Bradyrh	Salinarimonas	0	0	0	0	0	0	0	0	0	0	0	1	1
Cluster26318	Acidobact	Acidobacteria_Gp2			Gp2	0	0	0	0	0	0	1	0	0	0	0	0	1
Cluster26324	Proteobac	Gammaproteobacteria				1	0	0	0	0	0	0	0	0	0	0	0	1
Cluster26327	Acidobact	Acidobacteria_Gp1			Gp1	1	0	0	0	0	0	0	0	0	0	0	0	1
Cluster26329	Actinobac	Actinol	Actinomycetales			0	0	0	0	0	0	0	0	0	1	0	0	1
Cluster26333	Acidobact	Acidobacteria_Gp3			Gp3	0	1	0	0	0	0	0	0	0	0	0	0	1
Cluster26335	Bacteroid	Sphing	Sphir	Sphingo	Mucilaginibact	1	0	0	0	0	0	0	0	0	0	0	0	1
Cluster26357	Actinobac	Actinobacteria				0	0	1	0	0	0	0	0	0	0	0	0	1
Cluster26364	Chloroflex	Ktedor	Ktedonobacterales			0	0	0	0	1	0	0	0	0	0	0	0	1
Cluster26370	Proteobac	Gamm	Legio	Legione	Legionella	1	0	0	0	0	0	0	0	0	0	0	0	1
Cluster26375;size=1						0	0	0	0	0	0	0	0	0	0	1	0	1
Cluster26379	Acidobact	Acidobacteria_Gp2			Gp2	0	0	0	0	0	0	0	0	0	0	0	1	1
Cluster26392	Gemmatir	Gemm	Gemı	Gemma	Gemmatimona	1	0	0	0	0	0	0	0	0	0	0	0	1
Cluster26405;size=1						0	0	0	0	0	0	0	0	0	0	1	0	1
Cluster26407	Proteobac	Gammaproteobacteria				0	0	0	0	0	0	0	0	0	0	1	0	1
Cluster26410	Proteobac	Alphap	Rhod	Acetobacteraceae		0	0	0	0	0	0	1	0	0	0	0	0	1
Cluster26422	Proteobac	Alphap	Rhod	Acetoba	Acidisphaera	0	0	1	0	0	0	0	0	0	0	0	0	1
Cluster26423	Actinobac	Actinobacteria				1	0	0	0	0	0	0	0	0	0	0	0	1
Cluster26441	Chloroflex	Ktedor	Ktedonobacterales			0	0	0	0	0	0	1	0	0	0	0	0	1
Cluster26442	Proteobac	Alphaproteobacteria				0	0	0	1	0	0	0	0	0	0	0	0	1
Cluster26450	Proteobac	Alphap	Rhizobiales			0	0	0	0	0	0	1	0	0	0	0	0	1
Cluster26451	Proteobac	Gammaproteobacteria				1	0	0	0	0	0	0	0	0	0	0	0	1
Cluster26455	Proteobac	Betapr	Burkl	Oxalobacteraceae		0	0	1	0	0	0	0	0	0	0	0	0	1
Cluster26480	Acidobact	Acidobacteria_Gp2			Gp2	0	0	0	0	0	0	0	0	0	0	1	0	1
Cluster26482	Acidobact	Acidobacteria_Gp1				0	0	0	0	1	0	0	0	0	0	0	0	1

Cluster26484	Proteobac	Gammaproteobacteria				0	0	0	0	0	0	1	0	0	0	0	0	1
Cluster26486	Actinobac	Actinol	Actin	Streptoı	Streptacidiphil	1	0	0	0	0	0	0	0	0	0	0	0	1
Cluster26490	Acidobact	Acidobacteria_Gp1				0	0	0	0	1	0	0	0	0	0	0	0	1
Cluster26503;size=3						0	0	0	0	0	1	0	0	0	0	0	0	1
Cluster26508	Acidobacteria					1	0	0	0	0	0	0	0	0	0	0	0	1
Cluster26519;size=1						0	0	0	0	0	0	0	0	1	0	0	0	1
Cluster26522	Bacteroid	Sphing	Sphir	Chitinophagaceae		0	0	0	0	0	0	1	0	0	0	0	0	1
Cluster26524	Acidobact	Acidobacteria_Gp2			Gp2	0	0	0	0	1	0	0	0	0	0	0	0	1
Cluster26525	Proteobac	Gamm	Xantl	Sinobac	Steroidobacter	0	0	0	0	1	0	0	0	0	0	0	0	1
Cluster26529	Bacteroid	Sphing	Sphir	Chitinophagaceae		0	0	0	0	0	1	0	0	0	0	0	0	1
Cluster26533	Actinobac	Actinol	Actinomycetales			0	0	0	0	0	0	0	0	0	0	1	0	1
Cluster26539	Actinobac	Actinol	Solirı	Conexib	Conexibacter	0	0	0	0	1	0	0	0	0	0	0	0	1
Cluster26540	Proteobacteria					0	0	0	1	0	0	0	0	0	0	0	0	1
Cluster26543	Verrucom	Subdivision3			Subdivision3_g	0	0	0	0	1	0	0	0	0	0	0	0	1
Cluster26545	Proteobac	Alphap	Caulc	Cauloba	Phenylobacter	0	0	1	0	0	0	0	0	0	0	0	0	1
Cluster26546	Acidobact	Acidobacteria_Gp2			Gp2	0	0	0	0	0	0	0	0	0	0	1	0	1
Cluster26548	Proteobac	Gamm	Xantl	Xanthor	Stenotrophom	0	0	0	0	0	1	0	0	0	0	0	0	1
Cluster26551	Actinobac	Actinol	Actin	Nocardi	Rhodococcus	0	0	0	0	1	0	0	0	0	0	0	0	1
Cluster26554	Gemmatir	Gemm	Gemı	Gemma	Gemmatimona	1	0	0	0	0	0	0	0	0	0	0	0	1
Cluster26557	Proteobac	Alphaproteobacteria				0	0	0	0	0	0	0	0	0	0	1	0	1
Cluster26558	Acidobact	Acidobacteria_Gp1				0	0	0	1	0	0	0	0	0	0	0	0	1
Cluster26564	Acidobact	Acidobacteria_Gp3			Gp3	1	0	0	0	0	0	0	0	0	0	0	0	1
Cluster26565	Acidobact	Acidobacteria_Gp6			Gp6	0	0	1	0	0	0	0	0	0	0	0	0	1
Cluster26567	Proteobac	Alphap	Caulc	Cauloba	Phenylobacter	0	0	0	0	0	1	0	0	0	0	0	0	1
Cluster26579;size=1						0	0	0	0	0	0	0	0	0	0	1	0	1
Cluster26585	Proteobac	Alphaproteobacteria				0	0	0	0	0	0	0	0	0	0	1	0	1
Cluster26589	Proteobac	Alphap	Alphaproteob		Rhizomicrobiu	0	0	1	0	0	0	0	0	0	0	0	0	1
Cluster26592	Proteobac	Alphaproteobacteria				0	0	0	1	0	0	0	0	0	0	0	0	1
Cluster26604	Proteobac	Alphap	Rhod	Acetobacteraceae		0	0	0	0	1	0	0	0	0	0	0	0	1

Cluster26606	Bacteroidetes					0	0	0	1	0	0	0	0	0	0	0	0	1
Cluster26612	Proteobac	Betapr	Burkholderiales			0	0	0	0	1	0	0	0	0	0	0	0	1
Cluster26613	Acidobact	Acidobacteria_Gp1			Gp1	0	0	0	0	1	0	0	0	0	0	0	0	1
Cluster26615	Actinobac	Actinob	Actin	Nocardioidaceae		0	0	0	0	0	0	0	0	1	0	0	0	1
Cluster26616	Proteobac	Alphap	Rhizobiales			0	0	0	0	0	1	0	0	0	0	0	0	1
Cluster26618	Actinobac	Actinob	Actinomycetales			0	0	0	0	0	0	0	1	0	0	0	0	1
Cluster26624	Acidobact	Acidobacteria_Gp2			Gp2	0	0	0	0	0	0	0	1	0	0	0	0	1
Cluster26628	Proteobac	Alphap	Caulo	Caulobacteraceae		1	0	0	0	0	0	0	0	0	0	0	0	1
Cluster26632	Acidobact	Acidobacteria_Gp2			Gp2	0	0	0	0	1	0	0	0	0	0	0	0	1
Cluster26638	Proteobac	Alphaproteobacteria				0	0	0	0	0	0	0	0	0	0	1	0	1
Cluster26644	Bacteroid	Sphing	Sphin	Chitinop	Flavitalea	0	0	0	0	1	0	0	0	0	0	0	0	1
Cluster26649	Proteobac	Alphap	Caulo	Cauloba	Phenylobacter	0	0	0	1	0	0	0	0	0	0	0	0	1
Cluster26659	Actinobac	Actinob	Actin	Pseudonocardiaceae		0	0	1	0	0	0	0	0	0	0	0	0	1
Cluster26660	Actinobac	Actinob	Acidi	Acidimi	Aciditerrimona	0	0	0	0	0	0	0	0	0	0	1	0	1
Cluster26665	Actinobac	Actinob	Actin	Pseudon	Kibdelosporan	0	0	0	0	0	0	1	0	0	0	0	0	1
Cluster26670	Proteobac	Deltap	Bdell	Bdellovi	Bdellovibrio	1	0	0	0	0	0	0	0	0	0	0	0	1
Cluster26681	Acidobact	Acidobacteria_Gp1			Acidobacteriur	1	0	0	0	0	0	0	0	0	0	0	0	1
Cluster26691	Proteobac	Alphap	Rhizo	Methylocystaceae		0	0	0	0	1	0	0	0	0	0	0	0	1
Cluster26694	Bacteroid	Sphing	Sphin	Chitinophagaceae		0	0	1	0	0	0	0	0	0	0	0	0	1
Cluster26695	Acidobact	Acidobacteria_Gp1				0	0	0	0	0	0	0	0	0	0	1	0	1
Cluster26696	Actinobac	Actinob	Actin	Actinosp	Actinospica	0	0	0	0	0	0	0	1	0	0	0	0	1
Cluster26702	Chloroflex	Ktedor	Ktedonobacterales			0	0	0	0	0	0	1	0	0	0	0	0	1
Cluster26703;size=1						0	0	0	0	1	0	0	0	0	0	0	0	1
Cluster26704;size=7						0	0	0	0	0	1	0	0	0	0	0	0	1
Cluster26707	Bacteroid	Sphing	Sphin	Chitinophagaceae		0	0	0	0	1	0	0	0	0	0	0	0	1
Cluster26708	Bacteroid	Sphing	Sphin	Chitinop	Terrimonas	0	0	0	0	0	0	0	0	0	0	0	1	1
Cluster26711	Bacteroid	Sphing	Sphin	Sphingo	Mucilaginibact	0	0	0	0	0	0	0	1	0	0	0	0	1
Cluster26728	Verrucom	Subdivision3			Subdivision3_g	0	0	0	0	0	0	1	0	0	0	0	0	1
Cluster26743	Actinobac	Actinob	Actinomycetales			0	0	0	1	0	0	0	0	0	0	0	0	1

Cluster26745	Actinobac	Actinol	Acidimicrobiales			0	0	0	0	1	0	0	0	0	0	0	0	1
Cluster26753	Bacteroid	Sphing	Sphir	Chitinop	Segetibacter	0	0	0	0	0	0	0	0	0	0	0	1	1
Cluster26755	Actinobac	Actinol	Actinomycetales			1	0	0	0	0	0	0	0	0	0	0	0	1
Cluster26760	Actinobac	Actinol	Actinomycetales			0	0	0	0	0	0	0	0	0	1	0	0	1
Cluster26766	Bacteroid	Sphing	Sphir	Chitinophagaceae		0	0	1	0	0	0	0	0	0	0	0	0	1
Cluster26774	Actinobac	Actinol	Actinomycetales			0	0	0	0	1	0	0	0	0	0	0	0	1
Cluster26778	Acidobact	Acidobacteria_Gp2			Gp2	0	0	0	0	1	0	0	0	0	0	0	0	1
Cluster26784	Actinobac	Actinol	Acidi	Acidimi	Aciditerrimona	0	0	0	0	1	0	0	0	0	0	0	0	1
Cluster26790	Proteobacteria					0	0	1	0	0	0	0	0	0	0	0	0	1
Cluster26793	Bacteroid	Sphing	Sphir	Chitinophagaceae		0	0	0	1	0	0	0	0	0	0	0	0	1
Cluster26794;size=1						0	0	1	0	0	0	0	0	0	0	0	0	1
Cluster26795	Acidobact	Acidobacteria_Gp3			Gp3	0	0	0	1	0	0	0	0	0	0	0	0	1
Cluster26801	Acidobact	Acidobacteria_Gp3				0	0	0	0	0	0	0	1	0	0	0	0	1
Cluster26807	Acidobact	Acidobacteria_Gp3				0	0	0	0	1	0	0	0	0	0	0	0	1
Cluster26813	Acidobact	Acidobacteria_Gp2			Gp2	0	0	0	0	0	0	0	0	0	0	1	0	1
Cluster26821	Proteobac	Gammaproteobacteria				0	0	0	0	0	0	0	0	1	0	0	0	1
Cluster26829	Proteobacteria					0	0	0	0	1	0	0	0	0	0	0	0	1
Cluster26831	Bacteroid	Sphing	Sphir	Chitinop	Terrimonas	0	0	0	0	0	0	0	0	1	0	0	0	1
Cluster26832	Chlamydia	Chlamy	Chlamydiales			0	0	0	0	1	0	0	0	0	0	0	0	1
Cluster26833	Proteobac	Alphap	Rhod	Acetobacteraceae		0	0	0	1	0	0	0	0	0	0	0	0	1
Cluster26834;size=7						0	0	1	0	0	0	0	0	0	0	0	0	1
Cluster26840;size=1						1	0	0	0	0	0	0	0	0	0	0	0	1
Cluster26845	Proteobac	Gammaproteobacteria				0	0	0	0	1	0	0	0	0	0	0	0	1
Cluster26847	Proteobac	Alphap	Rhizobiales			0	0	0	0	0	0	0	0	0	0	1	0	1
Cluster26850	Bacteroid	Sphing	Sphir	Chitinophagaceae		0	0	0	0	0	0	0	1	0	0	0	0	1
Cluster26851	Acidobact	Acidobacteria_Gp1			Gp1	0	0	0	0	0	0	1	0	0	0	0	0	1
Cluster26852	Proteobacteria					1	0	0	0	0	0	0	0	0	0	0	0	1
Cluster26858	Actinobac	Actinol	Acidi	Acidimi	Aciditerrimona	0	0	0	0	0	0	0	1	0	0	0	0	1
Cluster26867	Actinobac	Actinol	Actinomycetales			0	0	0	0	0	0	0	0	0	0	0	1	1

Cluster26876	Actinobac	Actinob	Actinomycetales			0	0	0	0	0	0	0	0	0	0	1	0	1
Cluster26877;size=2						0	0	0	1	0	0	0	0	0	0	0	0	1
Cluster26879	Actinobac	Actinob	Actinomycetales			0	0	0	0	0	0	0	1	0	0	0	0	1
Cluster26882	Bacteroid	Sphing	Sphir	Chitinop	Segetibacter	0	0	0	0	1	0	0	0	0	0	0	0	1
Cluster26884	Proteobac	Alphap	Caulo	Caulobacteraceae		0	0	0	1	0	0	0	0	0	0	0	0	1
Cluster26887	Proteobac	Betapr	Burkholderiales			0	0	0	0	1	0	0	0	0	0	0	0	1
Cluster26888	Acidobact	Acidobacteria_Gp1				0	0	0	0	0	0	1	0	0	0	0	0	1
Cluster26901	Bacteroid	Sphing	Sphir	Sphingo	Mucilaginibact	0	0	0	0	0	0	0	0	0	1	0	0	1
Cluster26902	Acidobact	Acidobacteria_Gp1			Gp1	1	0	0	0	0	0	0	0	0	0	0	0	1
Cluster26909	Acidobact	Acidobacteria_Gp2			Gp2	0	0	0	1	0	0	0	0	0	0	0	0	1
Cluster26917	Bacteroid	Sphing	Sphir	Chitinophagaceae		0	0	0	0	1	0	0	0	0	0	0	0	1
Cluster26921	Actinobac	Actinob	Actin	Actinosp	Actinospica	0	0	0	0	0	0	1	0	0	0	0	0	1
Cluster26930	Actinobac	Actinobacteria				0	0	0	0	0	0	0	1	0	0	0	0	1
Cluster26943	Proteobac	Alphap	Ricke	Ricketts	Orientia	0	0	0	0	0	0	0	0	0	0	0	1	1
Cluster26944	Acidobact	Acidobacteria_Gp2			Gp2	0	0	0	0	1	0	0	0	0	0	0	0	1
Cluster26949	Acidobact	Acidobacteria_Gp2			Gp2	0	0	0	0	1	0	0	0	0	0	0	0	1
Cluster26955	Proteobac	Alphap	Alphaproteob		Rhizomicrobiu	0	0	0	1	0	0	0	0	0	0	0	0	1
Cluster26959	Acidobact	Acidobacteria_Gp6			Gp6	0	0	0	0	1	0	0	0	0	0	0	0	1
Cluster26964	Proteobac	Alphap	Caulo	Caulobac	Phenylobacter	0	0	0	0	0	1	0	0	0	0	0	0	1
Cluster26967	Proteobac	Deltap	Myxococcales			0	0	0	0	1	0	0	0	0	0	0	0	1
Cluster26970	Acidobact	Acidobacteria_Gp2			Gp2	0	0	0	1	0	0	0	0	0	0	0	0	1
Cluster26974	Actinobac	Actinob	Actinomycetales			0	0	0	0	0	0	0	0	0	0	1	0	1
Cluster26975	Proteobac	Alphaproteobacteria				0	0	0	0	0	0	0	0	0	1	0	0	1
Cluster26983	Actinobac	Actinobacteria				0	0	0	1	0	0	0	0	0	0	0	0	1
Cluster26992	Bacteroid	Sphing	Sphir	Chitinop	Terrimonas	0	0	0	0	0	0	0	0	0	0	0	1	1
Cluster26998	Proteobac	Gammaproteobacteria				0	0	0	1	0	0	0	0	0	0	0	0	1
Cluster27004	Bacteroid	Sphing	Sphir	Chitinophagaceae		0	0	0	0	0	0	0	1	0	0	0	0	1
Cluster27016	Acidobact	Acidobacteria_Gp13			Gp13	1	0	0	0	0	0	0	0	0	0	0	0	1
Cluster27020	Proteobac	Betapr	Burkl	Oxalobacteraceae		0	0	0	0	0	0	0	1	0	0	0	0	1

Cluster27041	Actinobac	Actinol	Actin	Actinos|	Actinospica	1	0	0	0	0	0	0	0	0	0	0	0	1
Cluster27045	Actinobac	Actinol	Acidi	Acidimi	Aciditerrimona	0	0	0	0	0	0	0	0	1	0	0	0	1
Cluster27047;size=1						0	0	0	1	0	0	0	0	0	0	0	0	1
Cluster27052;size=1						0	1	0	0	0	0	0	0	0	0	0	0	1
Cluster27056	Acidobact	Acidobacteria_Gp1			Gp1	0	0	0	0	0	0	1	0	0	0	0	0	1
Cluster27067	Acidobacteria					0	0	0	0	0	0	0	1	0	0	0	0	1
Cluster27070;size=1						0	0	0	0	0	0	1	0	0	0	0	0	1
Cluster27071	Actinobac	Actinol	Actinomycetales			0	0	0	0	0	0	0	1	0	0	0	0	1
Cluster27072;size=1						0	0	0	0	1	0	0	0	0	0	0	0	1
Cluster27074	Actinobac	Actinol	Actin	Actinos|	Actinospica	0	0	0	0	0	0	1	0	0	0	0	0	1
Cluster27077	Actinobac	Actinol	Actinomycetales			1	0	0	0	0	0	0	0	0	0	0	0	1
Cluster27081	Proteobac	Deltap	Bdell	Bdellovi	Bdellovibrio	0	0	0	0	1	0	0	0	0	0	0	0	1
Cluster27082	Acidobact	Acidobacteria_Gp13			Gp13	0	0	0	0	0	0	1	0	0	0	0	0	1
Cluster27086;size=1						0	0	0	0	0	0	1	0	0	0	0	0	1
Cluster27089;size=1						0	0	0	0	1	0	0	0	0	0	0	0	1
Cluster27092	Acidobact	Acidobacteria_Gp2			Gp2	0	0	0	0	0	0	0	0	0	0	1	0	1
Cluster27096	Acidobact	Acidobacteria_Gp6			Gp6	0	0	0	1	0	0	0	0	0	0	0	0	1
Cluster27097	Firmicute:	Clostri	Clost	Clostrid	Clostridiumser	0	0	0	1	0	0	0	0	0	0	0	0	1
Cluster27099	Proteobacteria					1	0	0	0	0	0	0	0	0	0	0	0	1
Cluster27104	Acidobacteria					0	0	0	0	0	0	0	1	0	0	0	0	1
Cluster27108	Actinobac	Actinol	Acidimicrobiales			0	0	0	0	0	0	1	0	0	0	0	0	1
Cluster27119	Actinobac	Actinobacteria				0	0	0	0	1	0	0	0	0	0	0	0	1
Cluster27120	Bacteroid	Sphing	Sphir	Chitino|	Sediminibacter	1	0	0	0	0	0	0	0	0	0	0	0	1
Cluster27123	Bacteroidetes					0	0	0	0	1	0	0	0	0	0	0	0	1
Cluster27125	Chloroflexi					0	0	0	1	0	0	0	0	0	0	0	0	1
Cluster27126	Actinobac	Actinol	Acidi	Acidimi	Aciditerrimona	0	0	0	0	1	0	0	0	0	0	0	0	1
Cluster27138	Bacteroid	Sphing	Sphir	Chitino|	Segetibacter	0	0	0	1	0	0	0	0	0	0	0	0	1
Cluster27141	Proteobac	Alphaproteobacteria				0	0	0	0	0	0	0	1	0	0	0	0	1
Cluster27143;size=1						0	0	0	0	0	0	0	0	0	0	0	1	1

Cluster27144	Bacteroid	Sphing	Sphir	Sphingo	Mucilaginibact	1	0	0	0	0	0	0	0	0	0	0	0	1
Cluster27151	Actinobac	Actinol	Acidi	Acidimi	Aciditerrimona	0	0	0	0	1	0	0	0	0	0	0	0	1
Cluster27152	Chlamydia	Chlamy	Chlamydiales			0	0	0	1	0	0	0	0	0	0	0	0	1
Cluster27158;size=1						1	0	0	0	0	0	0	0	0	0	0	0	1
Cluster27162;size=3						0	0	0	0	0	0	0	0	0	0	0	1	1
Cluster27166	Armatimonadetes				Armatimonade	1	0	0	0	0	0	0	0	0	0	0	0	1
Cluster27171	Acidobact	Acidobacteria_Gp5			Gp5	1	0	0	0	0	0	0	0	0	0	0	0	1
Cluster27181	Chloroflex	Ktedor	Ktedonobacterales			0	0	0	1	0	0	0	0	0	0	0	0	1
Cluster27186	Proteobac	Betapr	Neiss	Neisseriaceae		0	0	0	0	1	0	0	0	0	0	0	0	1
Cluster27190	Bacteroid	Sphing	Sphir	Chitinophagaceae		0	0	1	0	0	0	0	0	0	0	0	0	1
Cluster27191	Acidobact	Acidobacteria_Gp13			Gp13	0	0	0	0	1	0	0	0	0	0	0	0	1
Cluster27202	Acidobact	Acidobacteria_Gp1				0	0	0	0	0	0	0	0	0	1	0	0	1
Cluster27204	Bacteroid	Sphing	Sphir	Chitino	Sediminibacter	0	0	0	0	1	0	0	0	0	0	0	0	1
Cluster27217	Proteobac	Alphaproteobacteria				0	0	0	0	1	0	0	0	0	0	0	0	1
Cluster27218	Actinobac	Actinol	Actin	Thermomonosporacea		1	0	0	0	0	0	0	0	0	0	0	0	1
Cluster27220	Actinobac	Actinol	Actin	Thermomonosporacea		0	0	0	0	1	0	0	0	0	0	0	0	1
Cluster27223	Actinobac	Actinol	Acidi	Acidimi	Aciditerrimona	0	0	0	0	0	0	0	1	0	0	0	0	1
Cluster27224	Proteobacteria					0	0	0	0	0	0	1	0	0	0	0	0	1
Cluster27230	Acidobact	Acidobacteria_Gp1				0	0	0	0	0	0	0	0	0	1	0	0	1
Cluster27231	Bacteroid	Sphing	Sphir	Chitinophagaceae		0	0	0	0	0	0	0	0	0	0	1	0	1
Cluster27237	Actinobac	Actinol	Actinomycetales			0	0	0	0	0	0	0	0	0	1	0	0	1
Cluster27238;size=1						0	0	0	0	1	0	0	0	0	0	0	0	1
Cluster27249	Verrucom	Opituta	Opitu	Opituta	Opitutus	0	0	1	0	0	0	0	0	0	0	0	0	1
Cluster27257	Acidobact	Acidobacteria_Gp2			Gp2	0	0	0	0	0	1	0	0	0	0	0	0	1
Cluster27277	Acidobact	Acidobacteria_Gp13			Gp13	0	0	0	0	0	1	0	0	0	0	0	0	1
Cluster27285	Proteobac	Alphap	Rhod	Rhodos	Dongia	0	0	0	0	0	0	0	0	0	0	0	1	1
Cluster27294	Proteobacteria					0	0	0	0	0	1	0	0	0	0	0	0	1
Cluster27314	Acidobact	Acidobacteria_Gp2			Gp2	0	0	1	0	0	0	0	0	0	0	0	0	1
Cluster27327	Proteobac	Betaproteobacteria				0	0	0	0	1	0	0	0	0	0	0	0	1

Cluster27329	Bacteroid	Sphing	Sphir	Sphingc	Mucilaginibact	0	0	0	0	0	0	0	0	0	0	1	0	1
Cluster27331	Bacteroid	Sphing	Sphir	Chitinophagaceae		0	0	0	0	0	1	0	0	0	0	0	0	1
Cluster27340	Acidobact	Acidobacteria_Gp2			Gp2	0	0	1	0	0	0	0	0	0	0	0	0	1
Cluster27341	Proteobac	Alphaproteobacteria				0	0	0	0	1	0	0	0	0	0	0	0	1
Cluster27344	Bacteroid	Sphing	Sphir	Chitinop	Ferruginibacte	0	0	0	0	0	0	0	0	0	0	0	1	1
Cluster27346	Nitrospira	Nitrosp	Nitro	Nitrospi	Nitrospira	0	0	0	0	0	0	1	0	0	0	0	0	1
Cluster27349	Verrucom	Spartobacteria			Spartobacteria	0	0	0	1	0	0	0	0	0	0	0	0	1
Cluster27353	Proteobac	Gamm	Xantl	Sinobac	Steroidobacter	0	0	0	0	0	0	1	0	0	0	0	0	1
Cluster27356	TM7				TM7_genera_i	0	0	0	0	0	0	0	1	0	0	0	0	1
Cluster27358	Acidobact	Acidobacteria_Gp1			Gp1	0	0	0	0	0	0	1	0	0	0	0	0	1
Cluster27361;size=1						0	0	0	0	0	0	0	0	1	0	0	0	1
Cluster27375;size=1						0	0	1	0	0	0	0	0	0	0	0	0	1
Cluster27376	Proteobac	Gamm	Pseudomonadales			0	0	0	0	0	0	0	0	0	0	1	0	1
Cluster27383	Acidobact	Acidobacteria_Gp2			Gp2	0	0	0	0	0	0	1	0	0	0	0	0	1
Cluster27387	Acidobact	Acidobacteria_Gp2			Gp2	0	0	0	0	0	0	0	0	0	0	0	1	1
Cluster27388;size=3						0	0	0	0	0	0	0	0	0	0	1	0	1
Cluster27395	Acidobact	Acidobacteria_Gp3			Gp3	0	0	0	0	0	0	0	0	0	0	0	1	1
Cluster27406;size=1						0	0	1	0	0	0	0	0	0	0	0	0	1
Cluster27407;size=2						0	0	0	0	0	0	1	0	0	0	0	0	1
Cluster27408;size=1						0	0	0	0	0	0	0	1	0	0	0	0	1
Cluster27410	Proteobac	Gammaproteobacteria				0	0	0	0	0	0	0	0	0	0	0	1	1
Cluster27411	Proteobac	Alphaproteobacteria				0	0	1	0	0	0	0	0	0	0	0	0	1
Cluster27420	Bacteroid	Sphing	Sphir	Chitinophagaceae		0	0	0	0	0	0	1	0	0	0	0	0	1
Cluster27422;size=1						0	0	1	0	0	0	0	0	0	0	0	0	1
Cluster27439	Actinobac	Actinol	Actin	Actinos	Actinospica	0	0	0	0	0	0	0	0	0	0	0	1	1
Cluster27441	Proteobac	Alphap	Caulc	Caulobа	Phenylobacter	0	0	1	0	0	0	0	0	0	0	0	0	1
Cluster27442	Acidobact	Acidobacteria_Gp1				0	0	0	0	0	0	1	0	0	0	0	0	1
Cluster27444	Actinobac	Actinol	Actinomycetales			0	0	0	0	0	0	0	0	0	0	0	1	1
Cluster27445	Acidobact	Acidobacteria_Gp13			Gp13	0	0	0	0	0	0	0	0	1	0	0	0	1

Cluster27448	Bacteroid	Sphing	Sphir	Chitinop	Segetibacter	0	0	0	0	0	0	1	0	0	0	0	0	1
Cluster27451	Acidobact	Acidobacteria_Gp13			Gp13	0	0	0	0	0	0	1	0	0	0	0	0	1
Cluster27457	Bacteroid	Sphing	Sphir	Chitinophagaceae		0	0	1	0	0	0	0	0	0	0	0	0	1
Cluster27472	Acidobact	Acidobacteria_Gp7			Gp7	0	0	1	0	0	0	0	0	0	0	0	0	1
Cluster27474	Actinobac	Actinol	Actinomycetales			0	0	0	0	0	0	1	0	0	0	0	0	1
Cluster27475	Verrucom	Spartobacteria			Spartobacteria	0	0	1	0	0	0	0	0	0	0	0	0	1
Cluster27494	Bacteroid	Sphing	Sphir	Chitinophagaceae		0	0	1	0	0	0	0	0	0	0	0	0	1
Cluster27497	Acidobact	Acidobacteria_Gp13			Gp13	0	0	0	0	0	0	1	0	0	0	0	0	1
Cluster27499	Acidobact	Acidobacteria_Gp1				0	0	0	0	0	1	0	0	0	0	0	0	1
Cluster27508;size=3						0	0	1	0	0	0	0	0	0	0	0	0	1
Cluster27510;size=3						0	0	1	0	0	0	0	0	0	0	0	0	1
Cluster27515	Proteobac	Alphap	Rhod	Acetoba	Acidisphaera	0	0	0	1	0	0	0	0	0	0	0	0	1
Cluster27520;size=1						0	0	0	0	0	0	0	0	1	0	0	0	1
Cluster27529	Actinobac	Actinol	Actinomycetales			0	0	0	0	0	0	1	0	0	0	0	0	1
Cluster27545	Acidobact	Acidobacteria_Gp2			Gp2	0	0	0	0	0	0	1	0	0	0	0	0	1
Cluster27550	Proteobac	Gamm	Xantl	Sinobac	Steroidobacter	0	0	0	0	0	0	1	0	0	0	0	0	1
Cluster27551	Proteobac	Gamm	Xantl	Sinobac	Steroidobacter	0	0	1	0	0	0	0	0	0	0	0	0	1
Cluster27552	Verrucom	Opituta	Opitu	Opituta	Opitutus	0	0	0	0	0	0	0	0	0	0	0	1	1
Cluster27580	Actinobac	Actinol	Actinomycetales			0	0	0	0	0	0	0	0	0	0	1	0	1
Cluster27581	Chloroflex	Ktedor	Kted	Ktedono	Ktedonobacter	0	0	0	0	0	0	0	1	0	0	0	0	1
Cluster27585	Bacteroid	Sphing	Sphir	Chitinop	Ferruginibacte	0	0	0	0	0	0	1	0	0	0	0	0	1
Cluster27592	Firmicutes					0	0	0	0	0	0	0	0	0	0	0	1	1
Cluster27600	Acidobact	Acidobacteria_Gp3			Gp3	0	0	0	1	0	0	0	0	0	0	0	0	1
Cluster27603	Acidobact	Acidobacteria_Gp3			Bryobacter	0	0	0	0	0	0	1	0	0	0	0	0	1
Cluster27605	Actinobac	Actinol	Soliru	Conexib	Conexibacter	0	0	1	0	0	0	0	0	0	0	0	0	1
Cluster27606	Proteobac	Alphap	Alphaproteob		Rhizomicrobiu	0	0	0	0	0	0	1	0	0	0	0	0	1
Cluster27618	Bacteroid	Sphing	Sphir	Chitinophagaceae		0	0	1	0	0	0	0	0	0	0	0	0	1
Cluster27621	Chloroflex	Ktedor	Kted	Ktedono	Ktedonobacter	0	0	0	1	0	0	0	0	0	0	0	0	1
Cluster27623	Acidobact	Acidobacteria_Gp3			Bryobacter	0	0	0	0	0	0	0	0	1	0	0	0	1

Cluster27635	Proteobac	Deltaproteobacteria				0	0	0	0	0	0	0	0	0	0	1	0	1
Cluster27649	Bacteroidetes					0	0	0	0	0	0	0	0	0	0	1	0	1
Cluster27660	Bacteroid	Bacteroidetes_incert			Ohtaekwangia	0	0	0	0	0	0	1	0	0	0	0	0	1
Cluster27661	Proteobac	Deltap	Myxococcales			0	0	1	0	0	0	0	0	0	0	0	0	1
Cluster27669	Proteobac	Alphap	Rhodospirillales			0	0	0	0	0	0	0	0	0	0	0	1	1
Cluster27674	Bacteroid	Sphing	Sphir	Chitinophagaceae		0	0	0	0	0	0	0	0	0	0	1	0	1
Cluster27676	Proteobac	Alphap	Caulc	Caulobа	Caulobacter	0	0	0	1	0	0	0	0	0	0	0	0	1
Cluster27700	Proteobac	Alphap	Alphaproteob		Rhizomicrobiu	0	0	0	0	0	1	0	0	0	0	0	0	1
Cluster27703	Proteobac	Alphap	Rhizc	Bradyrh	Agromonas	0	0	0	0	0	0	0	0	1	0	0	0	1
Cluster27704	Bacteroid	Sphing	Sphir	Sphingc	Pedobacter	0	0	0	0	0	0	1	0	0	0	0	0	1
Cluster27706	Bacteroid	Sphing	Sphir	Chitinophagaceae		0	0	0	1	0	0	0	0	0	0	0	0	1
Cluster27708	Verrucom	Opituta	Opitu	Opituta	Opitutus	0	0	0	0	0	1	0	0	0	0	0	0	1
Cluster27710	Actinobac	Actinol	Actin	Actinos	Actinospica	0	0	0	0	0	0	0	0	0	0	1	0	1
Cluster27719	Actinobac	Actinol	Acidimicrobiales			0	0	0	0	0	0	0	1	0	0	0	0	1
Cluster27720	Bacteroid	Sphing	Sphir	Chitinophagaceae		0	0	0	1	0	0	0	0	0	0	0	0	1
Cluster27721;size=2						0	0	0	1	0	0	0	0	0	0	0	0	1
Cluster27730	Bacteroid	Sphing	Sphir	Chitinophagaceae		0	0	0	0	0	0	0	0	0	0	1	0	1
Cluster27731	Proteobac	Deltaproteobacteria				0	0	1	0	0	0	0	0	0	0	0	0	1
Cluster27737	Acidobact	Acidobacteria_Gp1				0	0	1	0	0	0	0	0	0	0	0	0	1
Cluster27743	Acidobact	Acidobacteria_Gp1				0	0	0	0	0	0	1	0	0	0	0	0	1
Cluster27750;size=1						0	0	0	0	0	0	0	1	0	0	0	0	1
Cluster27751	Actinobac	Actinol	Actin	Pseudonocardiaceae		0	0	1	0	0	0	0	0	0	0	0	0	1
Cluster27764	Actinobac	Actinol	Actin	Sporich	Sporichthya	0	0	0	1	0	0	0	0	0	0	0	0	1
Cluster27765	Acidobact	Acidobacteria_Gp3			Bryobacter	0	0	0	0	0	0	0	0	0	0	1	0	1
Cluster27769	Acidobact	Acidobacteria_Gp1				0	0	0	0	0	1	0	0	0	0	0	0	1
Cluster27774	Actinobac	Actinol	Acidi	Acidimi	Aciditerrimona	1	0	0	0	0	0	0	0	0	0	0	0	1
Cluster27781	Acidobact	Acidobacteria_Gp3				1	0	0	0	0	0	0	0	0	0	0	0	1
Cluster27786	Proteobac	Alphap	Rhizc	Beijerinckiaceae		0	0	0	0	0	0	1	0	0	0	0	0	1
Cluster27796	Proteobac	Gammaproteobacteria				0	0	1	0	0	0	0	0	0	0	0	0	1

Cluster27805	Acidobacteria					0	0	0	0	0	0	0	1	0	0	0	0	1
Cluster27806	Proteobac	Alphap	Rhizo	Methylocystaceae		0	0	0	0	0	0	0	1	0	0	0	0	1
Cluster27810	Acidobacteria					0	0	0	0	0	0	1	0	0	0	0	0	1
Cluster27814	Proteobac	Alphap	Rhizo	Bradyrh	Agromonas	0	0	0	0	0	0	0	0	0	0	1	0	1
Cluster27823;size=1						1	0	0	0	0	0	0	0	0	0	0	0	1
Cluster27824	Acidobact	Acidobacteria_Gp1				0	0	0	1	0	0	0	0	0	0	0	0	1
Cluster27837	Actinobac	Actinol	Soliru	Solirubr	Solirubrobacte	0	0	0	0	0	1	0	0	0	0	0	0	1
Cluster27838	Actinobac	Actinol	Acidimicrobiales			0	0	0	0	0	0	1	0	0	0	0	0	1
Cluster27859	Proteobac	Gammaproteobacteria				0	0	1	0	0	0	0	0	0	0	0	0	1
Cluster27860	Acidobact	Acidobacteria_Gp2			Gp2	0	0	0	0	0	0	1	0	0	0	0	0	1
Cluster27873	Proteobac	Alphaproteobacteria				0	0	0	0	0	0	0	0	1	0	0	0	1
Cluster27878	Proteobac	Alphap	Rhod	Acetobacteraceae		0	0	0	1	0	0	0	0	0	0	0	0	1
Cluster27883	Planctomy	Plancto	Planc	Plancto	Singulisphaera	0	0	0	1	0	0	0	0	0	0	0	0	1
Cluster27884	Acidobact	Acidobacteria_Gp3				0	0	0	0	0	0	0	1	0	0	0	0	1
Cluster27893	Proteobac	Alphap	Rhizo	Bradyrh	Agromonas	0	0	0	0	0	0	1	0	0	0	0	0	1
Cluster27898	Actinobac	Actinol	Actinomycetales			0	0	0	0	0	0	1	0	0	0	0	0	1
Cluster27906	Chlamydia	Chlamy	Chlar	Parachla	Neochlamydia	0	0	0	0	0	0	1	0	0	0	0	0	1
Cluster27938	Proteobac	Betapr	Burkl	Burkholderiaceae		0	0	0	0	0	0	0	0	0	0	1	0	1
Cluster27939	Proteobac	Gammaproteobacteria				0	0	0	0	0	0	0	1	0	0	0	0	1
Cluster27949;size=2						0	0	0	0	0	0	0	0	0	0	1	0	1
Cluster27950;size=1						0	0	0	0	0	1	0	0	0	0	0	0	1
Cluster27955	Actinobac	Actinol	Acidimicrobiales			0	0	0	0	0	0	0	0	0	0	1	0	1
Cluster27958	Acidobact	Acidobacteria_Gp3			Gp3	0	0	1	0	0	0	0	0	0	0	0	0	1
Cluster27962	Bacteroid	Sphing	Sphir	Sphingo	Pedobacter	0	0	1	0	0	0	0	0	0	0	0	0	1
Cluster27964	Bacteroid	Sphing	Sphir	Chitinop	Sediminibacter	0	0	0	0	0	0	1	0	0	0	0	0	1
Cluster27966	Bacteroid	Sphing	Sphir	Sphingo	Sphingobacter	0	0	0	0	0	0	0	1	0	0	0	0	1
Cluster27969	Bacteroid	Sphing	Sphir	Chitinophagaceae		0	0	1	0	0	0	0	0	0	0	0	0	1
Cluster27971;size=1						0	0	0	0	0	0	1	0	0	0	0	0	1
Cluster27974;size=2						0	0	0	1	0	0	0	0	0	0	0	0	1

Cluster27980	Proteobac	Alphap	Alphaproteob		Rhizomicrobiu	0	0	1	0	0	0	0	0	0	0	0	0	1
Cluster27989;size=1						0	0	0	0	0	1	0	0	0	0	0	0	1
Cluster27990	Acidobact	Acidobacteria_Gp2			Gp2	0	0	1	0	0	0	0	0	0	0	0	0	1
Cluster27994;size=1						0	0	0	0	0	0	0	0	1	0	0	0	1
Cluster28003	Proteobac	Alphap	Rhizo	Hyphon	Rhodoplanes	0	0	0	0	0	1	0	0	0	0	0	0	1
Cluster28009	Acidobact	Acidobacteria_Gp1				0	0	1	0	0	0	0	0	0	0	0	0	1
Cluster28022	Acidobacteria					0	0	0	0	0	1	0	0	0	0	0	0	1
Cluster28024;size=1						1	0	0	0	0	0	0	0	0	0	0	0	1
Cluster28025	Actinobac	Actinol	Actinomycetales			1	0	0	0	0	0	0	0	0	0	0	0	1
Cluster28029	Proteobac	Alphap	Caulo	Caulobа	Brevundimona	0	0	0	1	0	0	0	0	0	0	0	0	1
Cluster28032	Gemmatir	Gemm	Gemı	Gemma	Gemmatimona	1	0	0	0	0	0	0	0	0	0	0	0	1
Cluster28035	Proteobac	Alphap	Rhizobiales			1	0	0	0	0	0	0	0	0	0	0	0	1
Cluster28037	Proteobac	Alphap	Caulo	Caulobа	Phenylobacter	1	0	0	0	0	0	0	0	0	0	0	0	1
Cluster28047;size=6						1	0	0	0	0	0	0	0	0	0	0	0	1
Cluster28051	Bacteroid	Bacteroidetes_incert			Ohtaekwangia	0	1	0	0	0	0	0	0	0	0	0	0	1
Cluster28060	Acidobact	Acidobacteria_Gp2			Gp2	1	0	0	0	0	0	0	0	0	0	0	0	1
Cluster28064	Proteobac	Gamm	Legio	Coxiella	Coxiella	0	1	0	0	0	0	0	0	0	0	0	0	1
Cluster28067	Acidobact	Acidobacteria_Gp1				0	0	0	0	0	0	1	0	0	0	0	0	1
Cluster28068;size=2						0	0	0	0	0	1	0	0	0	0	0	0	1
Cluster28071	Proteobac	Gammaproteobacteria				0	0	0	0	0	0	0	1	0	0	0	0	1
Cluster28073;size=5						1	0	0	0	0	0	0	0	0	0	0	0	1
Cluster28076	Proteobac	Gamm	Xantl	Sinobac	Steroidobacter	0	0	0	0	0	0	0	0	0	0	0	1	1
Cluster28077	Chlorobi					0	0	0	0	0	0	0	0	0	0	0	1	1
Cluster28078	Acidobact	Acidobacteria_Gp2			Gp2	0	0	0	0	0	0	0	0	0	0	0	1	1
Cluster28083	Proteobac	Betapr	Burkl	Burkholderiales_incert		1	0	0	0	0	0	0	0	0	0	0	0	1
Cluster28089	Acidobact	Acidobacteria_Gp1				1	0	0	0	0	0	0	0	0	0	0	0	1
Cluster28101;size=1						0	0	0	0	0	0	0	0	0	0	0	1	1
Cluster28106	Proteobac	Gammaproteobacteria				1	0	0	0	0	0	0	0	0	0	0	0	1
Cluster28107	Bacteroidetes					1	0	0	0	0	0	0	0	0	0	0	0	1

Cluster28108	Proteobac	Alphaproteobacteria				1	0	0	0	0	0	0	0	0	0	0	0	1
Cluster28118	Bacteroid	Sphing	Sphir	Cytophagaceae		0	0	1	0	0	0	0	0	0	0	0	0	1
Cluster28119	Actinobac	Actinol	Acidimicrobiales			0	0	0	0	0	0	0	0	0	0	1	0	1
Cluster28124	Proteobac	Alphap	Alphaproteok		Rhizomicrobiu	0	1	0	0	0	0	0	0	0	0	0	0	1
Cluster28132	Actinobac	Actinol	Actin	Catenul	Catenulispora	0	0	0	0	0	0	0	1	0	0	0	0	1
Cluster28143	Actinobac	Actinol	Acidimicrobiales			0	0	1	0	0	0	0	0	0	0	0	0	1
Cluster28147	Proteobac	Alphap	Rhizc	Bradyrh	Agromonas	0	0	0	0	0	0	0	0	0	0	1	0	1
Cluster28149;size=2						0	0	0	0	0	0	1	0	0	0	0	0	1
Cluster28162	Bacteroidetes					0	0	0	1	0	0	0	0	0	0	0	0	1
Cluster28163	Acidobact	Acidobacteria_Gp2			Gp2	0	0	0	0	0	0	0	1	0	0	0	0	1
Cluster28178	Actinobac	Actinol	Actin	Mycoba	Mycobacteriur	0	0	1	0	0	0	0	0	0	0	0	0	1
Cluster28186;size=4						0	0	0	1	0	0	0	0	0	0	0	0	1
Cluster28190	Acidobact	Acidobacteria_Gp3			Gp3	0	0	1	0	0	0	0	0	0	0	0	0	1
Cluster28193	Proteobac	Alphap	Rhizc	Bradyrhizobiaceae		0	0	1	0	0	0	0	0	0	0	0	0	1
Cluster28196	Proteobac	Alphaproteobacteria				0	0	1	0	0	0	0	0	0	0	0	0	1
Cluster28204	Actinobac	Actinol	Actinomycetales			0	0	0	0	0	0	0	0	1	0	0	0	1
Cluster28206;size=1						0	0	1	0	0	0	0	0	0	0	0	0	1
Cluster28207	Proteobac	Gammaproteobacteria				0	0	0	0	0	0	1	0	0	0	0	0	1
Cluster28208	Proteobac	Alphaproteobacteria				0	0	1	0	0	0	0	0	0	0	0	0	1
Cluster28210;size=2						0	0	0	0	0	0	1	0	0	0	0	0	1
Cluster28212	Proteobac	Gamm	Xantl	Sinobac	Steroidobacter	0	0	0	0	0	0	0	1	0	0	0	0	1
Cluster28232;size=4						0	0	0	0	0	0	0	1	0	0	0	0	1
Cluster28233	Proteobac	Gamm	Legio	Coxiella	Coxiella	0	0	1	0	0	0	0	0	0	0	0	0	1
Cluster28237	Proteobac	Alphaproteobacteria				0	0	0	0	0	1	0	0	0	0	0	0	1
Cluster28238	Actinobac	Actinol	Acidi	Acidimi	Aciditerrimona	0	0	0	0	0	0	0	0	0	0	1	0	1
Cluster28248	Proteobac	Betaproteobacteria				0	0	0	0	0	1	0	0	0	0	0	0	1
Cluster28250	Proteobacteria					0	0	0	1	0	0	0	0	0	0	0	0	1
Cluster28270	Actinobac	Actinol	Acidi	Acidimi	Aciditerrimona	0	0	0	1	0	0	0	0	0	0	0	0	1
Cluster28275;size=4						0	0	0	0	0	0	0	0	1	0	0	0	1

Cluster28284	Actinobac	Actinol	Acidi	Iamiace	Iamia	0	0	0	0	1	0	0	0	0	0	0	0	1
Cluster28291	Actinobac	Actinol	Actin	Streptoi	Streptomyces	0	0	0	0	0	0	0	0	0	0	1	0	1
Cluster28292	Proteobac	Gamm	Legio	Legione	Legionella	0	0	1	0	0	0	0	0	0	0	0	0	1
Cluster28301;size=1						0	0	1	0	0	0	0	0	0	0	0	0	1
Cluster28305	Actinobac	Actinol	Solirt	Conexib	Conexibacter	0	0	1	0	0	0	0	0	0	0	0	0	1
Cluster28328	Actinobac	Actinol	Actin	Thermo	Actinoallomuri	0	0	0	0	1	0	0	0	0	0	0	0	1
Cluster28336	Proteobac	Betapr	Burkl	Comamonadaceae		0	0	0	0	0	0	0	0	0	0	0	1	1
Cluster28339	Actinobac	Actinobacteria				0	0	0	0	0	0	0	0	0	0	1	0	1
Cluster28340	Proteobac	Alphap	Rhizobiales			0	0	1	0	0	0	0	0	0	0	0	0	1
Cluster28345	Proteobacteria					0	0	1	0	0	0	0	0	0	0	0	0	1
Cluster28350	Proteobac	Gamm	Xantl	Sinobac	Steroidobacter	0	0	1	0	0	0	0	0	0	0	0	0	1
Cluster28356;size=1						0	0	0	0	0	0	1	0	0	0	0	0	1
Cluster28358	Proteobac	Alphap	Rhod	Acetoba	Acidisphaera	0	0	1	0	0	0	0	0	0	0	0	0	1
Cluster28359	Proteobac	Betapr	Burkl	Burkholderiaceae		0	0	0	0	0	0	1	0	0	0	0	0	1
Cluster28369	Bacteroid	Sphing	Sphir	Sphingo	Mucilaginibact	0	0	1	0	0	0	0	0	0	0	0	0	1
Cluster28374	Actinobac	Actinobacteria				0	0	1	0	0	0	0	0	0	0	0	0	1
Cluster28379	Bacteroidetes					0	0	1	0	0	0	0	0	0	0	0	0	1
Cluster28388	Acidobact	Acidobacteria_Gp2			Gp2	0	0	0	0	0	0	1	0	0	0	0	0	1
Cluster28404	Acidobact	Acidobacteria_Gp1			Gp1	0	0	1	0	0	0	0	0	0	0	0	0	1
Cluster28407	Actinobac	Actinol	Actinomycetales			0	0	0	0	0	0	1	0	0	0	0	0	1
Cluster28411	Acidobact	Acidobacteria_Gp3			Gp3	0	0	0	0	0	0	1	0	0	0	0	0	1
Cluster28418;size=1						0	0	0	0	0	0	1	0	0	0	0	0	1
Cluster28426	Verrucom	Opituta	Opitu	Opituta	Opitutus	0	1	0	0	0	0	0	0	0	0	0	0	1
Cluster28439	Proteobac	Alphap	Caulo	Caulobа	Caulobacter	0	0	0	0	0	1	0	0	0	0	0	0	1
Cluster28440	Bacteroid	Sphing	Sphir	Sphingo	Mucilaginibact	0	0	0	1	0	0	0	0	0	0	0	0	1
Cluster28442	Proteobac	Gammaproteobacteria				0	0	0	0	1	0	0	0	0	0	0	0	1
Cluster28443	Actinobac	Actinol	Actinomycetales			0	0	0	1	0	0	0	0	0	0	0	0	1
Cluster28452	Proteobac	Alphap	Rhizobiales			0	0	0	0	0	0	0	0	0	1	0	0	1
Cluster28454	Bacteroid	Sphing	Sphir	Chitinophagaceae		0	0	1	0	0	0	0	0	0	0	0	0	1

Cluster2845ȿ	Chlorobi					0	0	0	1	0	0	0	0	0	0	0	0	1
Cluster2846(	Acidobact	Acidobacteria_Gp3			Gp3	0	0	0	0	0	0	0	0	0	1	0	0	1
Cluster2846ʑ	Bacteroid	Sphing	Sphir	Chitinophagaceae		0	0	0	0	1	0	0	0	0	0	0	0	1
Cluster2846ʌ	Acidobact	Acidobacteria_Gp2			Gp2	0	0	0	1	0	0	0	0	0	0	0	0	1
Cluster2846ϵ	Acidobact	Acidobacteria_Gp3			Bryobacter	0	0	0	0	1	0	0	0	0	0	0	0	1
Cluster2846ʤ	Proteobac	Alphap	Rhod	Acetobacteraceae		0	0	0	0	0	0	0	0	0	1	0	0	1
Cluster2847ʤ	Gemmatir	Gemm	Gemı	Gemma	Gemmatimona	0	0	0	0	1	0	0	0	0	0	0	0	1
Cluster28481	Bacteroid	Sphing	Sphir	Sphingc	Mucilaginibact	0	0	0	1	0	0	0	0	0	0	0	0	1
Cluster2848ȝ	Proteobac	Alphap	Caulc	Caulobа	Caulobacter	0	0	0	0	0	0	0	0	0	0	1	0	1
Cluster2848ʖ	Bacteroid	Sphing	Sphir	Chitinophagaceae		0	0	0	0	0	0	0	0	0	0	1	0	1
Cluster2848ȿ	Firmicute:	Clostri	Clost	Lachnospiraceae		0	0	0	0	0	0	0	0	0	0	1	0	1
Cluster2849(	Proteobac	Alphap	Alphaproteok		Rhizomicrobiu	0	0	0	0	1	0	0	0	0	0	0	0	1
Cluster2849ʖ	Bacteroid	Sphing	Sphir	Sphingc	Mucilaginibact	0	0	0	0	0	0	0	1	0	0	0	0	1
Cluster2849ϵ	Proteobac	Alphaproteobacteria				0	0	0	0	1	0	0	0	0	0	0	0	1
Cluster2849⁊	Proteobac	Gammaproteobacteria				0	0	0	0	1	0	0	0	0	0	0	0	1
Cluster2849ȿ	Proteobac	Alphap	Rhizc	Bradyrhizobiaceae		0	0	0	0	0	0	0	0	0	0	1	0	1
Cluster2851(	Actinobac	Actinol	Actinomycetales			0	0	0	0	0	0	0	0	0	0	1	0	1
Cluster28514;size=1						0	0	0	0	1	0	0	0	0	0	0	0	1
Cluster2851ʤ	Proteobac	Betapr	Burkl	Burkhol	Burkholderia	0	0	0	0	1	0	0	0	0	0	0	0	1
Cluster28521	Actinobac	Actinol	Acidimicrobiales			0	0	0	0	1	0	0	0	0	0	0	0	1
Cluster2852ʖ	Proteobac	Alphap	Rhizc	Methylocystaceae		0	0	0	0	0	0	0	0	0	0	1	0	1
Cluster2852⁊	Acidobact	Acidobacteria_Gp6			Gp6	0	0	0	0	0	0	0	0	0	0	1	0	1
Cluster2852ʤ	Bacteroidetes					0	0	0	1	0	0	0	0	0	0	0	0	1
Cluster2853ʑ	Bacteroid	Sphing	Sphir	Chitinophagaceae		0	0	0	0	1	0	0	0	0	0	0	0	1
Cluster2853ʖ	Actinobac	Actinol	Actinomycetales			0	0	0	0	1	0	0	0	0	0	0	0	1
Cluster2853ʤ	Planctomy	Planctc	Planc	Planctoı	Singulisphaera	0	0	0	0	0	0	0	0	0	0	1	0	1
Cluster2854⁊	Bacteroid	Sphing	Sphir	Sphingc	Parapedobactε	0	0	0	1	0	0	0	0	0	0	0	0	1
Cluster2856ϵ	Proteobac	Deltap	Myxc	Polyangiaceae		0	0	0	1	0	0	0	0	0	0	0	0	1
Cluster2856⁊	Acidobact	Acidobacteria_Gp2			Gp2	0	0	0	1	0	0	0	0	0	0	0	0	1

Cluster28569	Acidobact	Acidobacteria_Gp3				0	0	0	1	0	0	0	0	0	0	0	0	1
Cluster28571;size=1						0	0	0	0	0	0	0	0	0	0	1	0	1
Cluster28574;size=1						0	0	0	1	0	0	0	0	0	0	0	0	1
Cluster28589	Proteobac	Alphap	Alphaproteob		Rhizomicrobiu	0	0	0	0	0	1	0	0	0	0	0	0	1
Cluster28590	Acidobact	Acidobacteria_Gp6			Gp6	0	0	0	1	0	0	0	0	0	0	0	0	1
Cluster28596	Acidobacteria					0	0	0	0	0	0	0	0	1	0	0	0	1
Cluster28598	Actinobac	Actinol	Acidimicrobiales			0	0	0	1	0	0	0	0	0	0	0	0	1
Cluster28601	Proteobac	Betapr	Burkholderiales			0	0	0	0	0	1	0	0	0	0	0	0	1
Cluster28605	Actinobac	Actinol	Acidi	Acidimi	Aciditerrimona	0	0	0	0	0	0	0	0	0	0	1	0	1
Cluster28617	Proteobac	Betapr	Burkl	Comamonadaceae		0	0	0	1	0	0	0	0	0	0	0	0	1
Cluster28620	Proteobac	Deltap	Myxc	Cystobacteraceae		0	0	0	1	0	0	0	0	0	0	0	0	1
Cluster28621	Acidobact	Acidobacteria_Gp3				0	0	0	0	0	0	0	0	0	0	1	0	1
Cluster28636	Bacteroidetes					0	0	0	0	0	1	0	0	0	0	0	0	1
Cluster28641	Bacteroid	Sphing	Sphir	Chitinophagaceae		0	0	0	1	0	0	0	0	0	0	0	0	1
Cluster28642	Acidobact	Acidobacteria_Gp2			Gp2	0	0	0	1	0	0	0	0	0	0	0	0	1
Cluster28649	Bacteroid	Sphing	Sphir	Chitinophagaceae		0	0	1	0	0	0	0	0	0	0	0	0	1
Cluster28653	Acidobact	Acidobacteria_Gp6			Gp6	0	0	0	0	0	0	1	0	0	0	0	0	1
Cluster28672	Proteobac	Alphap	Rhizc	Xanthol	Pseudolabrys	0	0	1	0	0	0	0	0	0	0	0	0	1
Cluster28673	Actinobac	Actinol	Acidimicrobiales			0	0	0	0	0	0	0	1	0	0	0	0	1
Cluster28679	Actinobac	Actinol	Actinomycetales			0	0	0	0	0	0	1	0	0	0	0	0	1
Cluster28683	Acidobact	Acidobacteria_Gp1			Edaphobacter	0	0	0	0	0	0	1	0	0	0	0	0	1
Cluster28688	Bacteroid	Sphing	Sphir	Chitinophagaceae		0	0	0	0	0	0	0	0	0	0	0	1	1
Cluster28699	Proteobac	Betapr	Burkl	Burkhol	Burkholderia	0	0	0	0	0	0	0	0	0	0	1	0	1
Cluster28700	Proteobac	Gamm	Xantl	Xanthor	Rhodanobacte	0	0	0	0	0	0	1	0	0	0	0	0	1
Cluster28701	Chloroflex	Ktedor	Kted	Ktedon	Ktedonobacter	0	0	0	0	0	0	0	1	0	0	0	0	1
Cluster28704	Proteobac	Gamm	Xantl	Sinobacteraceae		0	0	0	0	0	0	0	0	0	0	0	1	1
Cluster28712	Proteobac	Betaproteobacteria				0	0	1	0	0	0	0	0	0	0	0	0	1
Cluster28713	Bacteroid	Sphing	Sphir	Sphingc	Mucilaginibact	0	0	0	0	0	0	0	1	0	0	0	0	1
Cluster28715	Actinobac	Actinol	Actinomycetales			0	0	0	0	0	0	1	0	0	0	0	0	1

Cluster28720	Proteobac	Alphaproteobacteria				0	0	0	0	0	0	0	1	0	0	0	0	1
Cluster28721	Proteobac	Alphaproteobacteria				0	0	0	0	0	0	0	1	0	0	0	0	1
Cluster28727	Proteobac	Alphap	Rhizobiales			0	0	0	0	0	0	1	0	0	0	0	0	1
Cluster28728	Proteobac	Alphap	Sphir	Sphingomonadaceae		0	0	0	0	0	0	1	0	0	0	0	0	1
Cluster28731	Actinobac	Actinob	Actinomycetales			0	0	1	0	0	0	0	0	0	0	0	0	1
Cluster28743	Bacteroid	Sphing	Sphir	Sphingo	Pedobacter	0	0	1	0	0	0	0	0	0	0	0	0	1
Cluster28748	Proteobac	Gamm	Xanth	Sinobac	Steroidobacter	0	0	1	0	0	0	0	0	0	0	0	0	1
Cluster28749	Acidobact	Acidobacteria_Gp2			Gp2	0	0	0	0	0	0	1	0	0	0	0	0	1
Cluster28750	Proteobac	Gammaproteobacteria				0	0	0	0	0	0	0	0	0	0	0	1	1
Cluster28758	Actinobac	Actinobacteria				0	0	0	0	0	0	1	0	0	0	0	0	1
Cluster28780	Proteobac	Betaproteobacteria				0	0	0	0	0	0	0	0	0	1	0	0	1
Cluster28781	Actinobac	Actinob	Actinomycetales			0	0	0	0	0	0	0	0	0	0	1	0	1
Cluster28793	Proteobac	Gamm	Xanth	Sinobac	Steroidobacter	0	0	0	1	0	0	0	0	0	0	0	0	1
Cluster28797	Bacteroid	Sphing	Sphir	Chitinop	Sediminibacter	0	0	0	0	0	1	0	0	0	0	0	0	1
Cluster28806	Proteobacteria					0	0	0	0	0	1	0	0	0	0	0	0	1
Cluster28807	Proteobac	Alphap	Caulo	Cauloba	Brevundimona	0	0	0	0	0	0	0	0	0	0	1	0	1
Cluster28811	Acidobact	Acidobacteria_Gp1			Gp1	0	0	1	0	0	0	0	0	0	0	0	0	1
Cluster28814	Actinobac	Actinob	Actinomycetales			0	0	1	0	0	0	0	0	0	0	0	0	1
Cluster28838	Proteobac	Betaproteobacteria				0	1	0	0	0	0	0	0	0	0	0	0	1
Cluster28839	Bacteroid	Sphing	Sphir	Chitinophagaceae		0	0	0	0	0	0	0	1	0	0	0	0	1
Cluster28855	Proteobac	Betaproteobacteria				0	0	1	0	0	0	0	0	0	0	0	0	1
Cluster28862	Actinobac	Actinob	Actin	Strepto	Streptomyces	0	0	1	0	0	0	0	0	0	0	0	0	1
Cluster28872	Proteobac	Alphap	Caulo	Cauloba	Brevundimona	0	0	0	0	0	0	0	0	0	0	1	0	1
Cluster28877	Actinobac	Actinob	Actinomycetales			0	0	1	0	0	0	0	0	0	0	0	0	1
Cluster28880	Actinobac	Actinob	Actinomycetales			0	0	0	0	0	0	0	0	1	0	0	0	1
Cluster28888	Actinobac	Actinobacteria				0	0	0	0	1	0	0	0	0	0	0	0	1
Cluster28890;size=1						0	0	0	0	0	0	0	0	0	0	0	1	1
Cluster28892	Actinobac	Actinob	Acidi	Acidimi	Aciditerrimona	0	0	1	0	0	0	0	0	0	0	0	0	1
Cluster28893	Bacteroid	Sphing	Sphir	Chitinophagaceae		0	0	0	0	1	0	0	0	0	0	0	0	1

Cluster28895	Bacteroid	Sphing	Sphir	Chitinophagaceae		0	0	0	0	0	0	1	0	0	0	0	0	1
Cluster28898;size=3						0	0	0	0	0	0	1	0	0	0	0	0	1
Cluster28899	Acidobact	Acidobacteria_Gp2			Gp2	0	0	0	0	0	0	0	0	0	0	0	1	1
Cluster28904;size=1						0	0	0	0	0	0	0	1	0	0	0	0	1
Cluster28911	Proteobac	Alphap	Alphaproteob		Rhizomicrobiu	0	0	0	0	0	0	0	0	0	0	1	0	1
Cluster28916	Bacteroid	Sphing	Sphir	Chitinophagaceae		0	0	0	0	1	0	0	0	0	0	0	0	1
Cluster28921	Acidobact	Acidobacteria_Gp1			Gp1	0	0	0	1	0	0	0	0	0	0	0	0	1
Cluster28929	Bacteroid	Sphing	Sphir	Chitinophagaceae		0	0	0	0	1	0	0	0	0	0	0	0	1
Cluster28930	Chloroflex	Ktedor	Ktedo	Ktedono	Ktedonobacter	0	0	0	1	0	0	0	0	0	0	0	0	1
Cluster28945	Proteobac	Gammaproteobacteria				0	0	0	0	1	0	0	0	0	0	0	0	1
Cluster28951	Actinobac	Actinol	Actinomycetales			1	0	0	0	0	0	0	0	0	0	0	0	1
Cluster28954	Proteobac	Alphap	Rhod	Acetoba	Stella	0	0	0	0	0	0	0	0	0	0	1	0	1
Cluster28955	Acidobact	Acidobacteria_Gp2			Gp2	0	0	0	0	0	0	0	1	0	0	0	0	1
Cluster28959	Bacteroid	Sphing	Sphir	Chitino	Flavitalea	1	0	0	0	0	0	0	0	0	0	0	0	1
Cluster28964	Acidobact	Acidobacteria_Gp2			Gp2	0	0	0	0	1	0	0	0	0	0	0	0	1
Cluster28974	Proteobac	Alphaproteobacteria				0	0	0	0	0	0	0	1	0	0	0	0	1
Cluster28976	Proteobac	Alphap	Rhod	Acetobacteraceae		0	0	0	0	0	0	0	0	0	0	1	0	1
Cluster28977	Actinobac	Actinol	Acidimicrobiales			0	0	0	0	0	0	0	0	0	0	0	1	1
Cluster28982	Bacteroid	Sphing	Sphir	Sphingo	Mucilaginibact	0	0	0	0	1	0	0	0	0	0	0	0	1
Cluster28983	Actinobac	Actinol	Actinomycetales			1	0	0	0	0	0	0	0	0	0	0	0	1
Cluster28984	Proteobacteria					1	0	0	0	0	0	0	0	0	0	0	0	1
Cluster28987	Proteobac	Alphaproteobacteria				1	0	0	0	0	0	0	0	0	0	0	0	1
Cluster29003	Proteobac	Alphap	Rhodospirillales			0	0	1	0	0	0	0	0	0	0	0	0	1
Cluster29020	Acidobact	Acidobacteria_Gp1			Gp1	0	0	0	0	0	1	0	0	0	0	0	0	1
Cluster29026	Bacteroidetes					0	0	0	0	0	1	0	0	0	0	0	0	1
Cluster29027	Bacteroid	Sphing	Sphir	Sphingo	Mucilaginibact	0	0	0	0	0	1	0	0	0	0	0	0	1
Cluster29036	Actinobac	Actinol	Acidi	Acidimi	Aciditerrimona	1	0	0	0	0	0	0	0	0	0	0	0	1
Cluster29040	Acidobact	Acidobacteria_Gp3			Gp3	0	0	0	0	0	0	0	0	0	0	0	1	1
Cluster29041	Acidobact	Acidobacteria_Gp1				0	0	0	1	0	0	0	0	0	0	0	0	1

Cluster2905	Actinobac	Actinol	Actinomycetales			0	0	0	0	0	0	0	1	0	0	0	0	1
Cluster29081	Actinobac	Actinol	Actin	Pseudonocardiaceae		0	0	0	0	0	0	0	0	0	0	0	1	1
Cluster29086;size=3						0	0	0	0	0	0	1	0	0	0	0	0	1
Cluster2909	Proteobac	Alphap	Caulc	Culoba	Phenylobacter	0	1	0	0	0	0	0	0	0	0	0	0	1
Cluster2909	Proteobac	Alphap	Alphaproteob		Rhizomicrobiu	0	0	0	0	0	0	0	0	0	0	0	1	1
Cluster2909	Acidobact	Acidobacteria_Gp2			Gp2	0	0	1	0	0	0	0	0	0	0	0	0	1
Cluster2910	Acidobact	Acidobacteria_Gp1				0	0	1	0	0	0	0	0	0	0	0	0	1
Cluster2911	Bacteroid	Sphing	Sphir	Chitinophagaceae		0	0	1	0	0	0	0	0	0	0	0	0	1
Cluster29111	Bacteroid	Sphing	Sphir	Sphingc	Pedobacter	0	0	0	1	0	0	0	0	0	0	0	0	1
Cluster2912	Acidobact	Acidobacteria_Gp3			Gp3	0	0	1	0	0	0	0	0	0	0	0	0	1
Cluster29131	Proteobac	Alphap	Caulc	Culoba	Brevundimona	0	0	0	0	0	0	1	0	0	0	0	0	1
Cluster2914	Bacteroid	Sphing	Sphir	Chitinophagaceae		0	0	1	0	0	0	0	0	0	0	0	0	1
Cluster2915	Acidobact	Acidobacteria_Gp2			Gp2	0	0	0	0	0	0	0	0	0	0	0	1	1
Cluster2916	Proteobac	Alphap	Rhodospirillales			0	0	1	0	0	0	0	0	0	0	0	0	1
Cluster2919	Bacteroid	Sphing	Sphir	Chitinophagaceae		0	0	0	0	0	0	0	0	0	0	0	1	1
Cluster2920	Bacteroid	Sphing	Sphir	Chitinophagaceae		0	0	1	0	0	0	0	0	0	0	0	0	1
Cluster2920	Actinobac	Actinol	Acidimicrobiales			0	0	0	0	0	0	1	0	0	0	0	0	1
Cluster2921	Bacteroid	Sphing	Sphir	Chitinop	Terrimonas	0	0	0	0	0	0	1	0	0	0	0	0	1
Cluster2921	Acidobact	Acidobacteria_Gp1			Gp1	0	0	0	0	0	0	1	0	0	0	0	0	1
Cluster29221	Bacteroidetes					0	0	0	1	0	0	0	0	0	0	0	0	1
Cluster29227;size=1						0	0	0	0	0	1	0	0	0	0	0	0	1
Cluster29231	Actinobac	Actinol	Acidi	Acidimi	Aciditerrimona	0	0	1	0	0	0	0	0	0	0	0	0	1
Cluster2923	Acidobact	Acidobacteria_Gp3			Gp3	0	0	0	0	0	0	0	0	0	0	1	0	1
Cluster2925	Bacteroidetes					1	0	0	0	0	0	0	0	0	0	0	0	1
Cluster2926	Proteobac	Alphap	Caulc	Caulobacteraceae		0	0	0	0	1	0	0	0	0	0	0	0	1
Cluster2926	Proteobac	Gammaproteobacteria				0	0	0	0	0	0	0	1	0	0	0	0	1
Cluster2927	Acidobact	Acidobacteria_Gp3				0	0	0	1	0	0	0	0	0	0	0	0	1
Cluster2927	Acidobact	Acidobacteria_Gp3			Gp3	0	0	0	0	1	0	0	0	0	0	0	0	1
Cluster2927	Proteobac	Alphap	Rhizc	Methylocystaceae		1	0	0	0	0	0	0	0	0	0	0	0	1

Cluster29291	Bacteroid	Flavob	Flavo	Cryomo	Owenweeksia	0	0	0	1	0	0	0	0	0	0	0	0	1
Cluster29292	Actinobac	Actinol	Acidimicrobiales			0	0	0	0	0	0	0	1	0	0	0	0	1
Cluster29293	Bacteroid	Sphing	Sphir	Sphingc	Nubsella	1	0	0	0	0	0	0	0	0	0	0	0	1
Cluster29298	Bacteroid	Sphing	Sphir	Sphingc	Mucilaginibact	0	0	0	0	0	0	0	1	0	0	0	0	1
Cluster29303	Acidobact	Acidobacteria_Gp3			Gp3	0	0	0	0	1	0	0	0	0	0	0	0	1
Cluster29305	Bacteroid	Sphing	Sphingobacteriales			0	0	0	0	0	0	0	0	0	1	0	0	1
Cluster29307	Bacteroid	Sphing	Sphir	Sphingc	Mucilaginibact	0	0	0	0	0	0	0	0	0	1	0	0	1
Cluster29309	Proteobac	Gammaproteobacteria				0	0	0	0	0	0	0	0	0	1	0	0	1
Cluster29315	Actinobac	Actinobacteria				0	0	1	0	0	0	0	0	0	0	0	0	1
Cluster29322	Bacteroid	Sphing	Sphir	Chitinophagaceae		0	0	0	1	0	0	0	0	0	0	0	0	1
Cluster29328	Proteobac	Gammaproteobacteria				0	0	0	0	0	0	1	0	0	0	0	0	1
Cluster29330	Actinobac	Actinol	Actinomycetales			0	0	0	0	0	1	0	0	0	0	0	0	1
Cluster29344	Chlorobi					0	0	0	0	0	0	0	0	0	0	1	0	1
Cluster29361;size=1						0	0	0	0	0	0	0	0	0	0	1	0	1
Cluster29366	Proteobac	Alphap	Caulc	Caulobacteraceae		0	0	1	0	0	0	0	0	0	0	0	0	1
Cluster29370	Proteobac	Deltaproteobacteria				0	0	1	0	0	0	0	0	0	0	0	0	1
Cluster29392	Actinobac	Actinobacteria				0	0	0	0	0	0	0	0	0	0	1	0	1
Cluster29394	Bacteroid	Sphing	Sphir	Chitinop	Terrimonas	0	0	0	0	0	0	0	0	0	0	0	1	1
Cluster29398	Proteobac	Alphap	Rhizc	Beijerin	Methylocapsa	0	0	0	0	0	0	0	1	0	0	0	0	1
Cluster29406	Bacteroid	Sphing	Sphir	Sphingobacteriaceae		0	0	0	0	0	0	1	0	0	0	0	0	1
Cluster29412;size=1						1	0	0	0	0	0	0	0	0	0	0	0	1
Cluster29413;size=5						0	0	0	1	0	0	0	0	0	0	0	0	1
Cluster29415	Actinobac	Actinol	Actinomycetales			0	0	0	0	0	0	0	1	0	0	0	0	1
Cluster29417	Bacteroid	Sphing	Sphir	Chitinophagaceae		0	0	0	0	1	0	0	0	0	0	0	0	1
Cluster29439;size=1						0	0	0	0	0	0	0	0	0	0	0	1	1
Cluster29450	Acidobact	Acidobacteria_Gp4			Gp4	0	0	0	0	0	0	0	0	1	0	0	0	1
Cluster29461	Bacteroid	Sphing	Sphir	Sphingc	Mucilaginibact	0	0	0	0	0	0	0	0	0	1	0	0	1
Cluster29465;size=1						0	0	0	0	0	0	0	0	0	0	1	0	1
Cluster29478	Proteobac	Alphap	Alphaproteok		Rhizomicrobiu	1	0	0	0	0	0	0	0	0	0	0	0	1

Cluster29495	Bacteroid	Sphing	Sphir	Chitinophagaceae		0	0	0	1	0	0	0	0	0	0	0	0	1
Cluster29501	Acidobact	Acidobacteria_Gp1				0	0	1	0	0	0	0	0	0	0	0	0	1
Cluster29505	Actinobac	Actinobacteria				0	0	0	0	0	0	0	0	0	0	1	0	1
Cluster29508	Bacteroid	Sphing	Sphir	Chitinophagaceae		0	0	0	1	0	0	0	0	0	0	0	0	1
Cluster29510	Acidobact	Acidobacteria_Gp2			Gp2	1	0	0	0	0	0	0	0	0	0	0	0	1
Cluster29526	Acidobact	Acidobacteria_Gp3				0	0	0	0	0	0	1	0	0	0	0	0	1
Cluster29527	Proteobac	Alphap	Rhod	Acetoba	Acidisphaera	0	0	0	0	1	0	0	0	0	0	0	0	1
Cluster29530	Actinobac	Actinobacteria				0	0	0	0	0	0	1	0	0	0	0	0	1
Cluster29533	Acidobacteria					0	0	0	0	0	0	1	0	0	0	0	0	1
Cluster29540	Bacteroid	Sphing	Sphir	Sphingo	Mucilaginibact	0	1	0	0	0	0	0	0	0	0	0	0	1
Cluster29542	Acidobact	Acidobacteria_Gp3			Gp3	0	0	0	0	0	0	0	0	0	1	0	0	1
Cluster29543	Bacteroid	Sphing	Sphir	Sphingo	Mucilaginibact	0	0	0	0	0	0	0	0	0	0	0	1	1
Cluster29544	Proteobac	Gamm	Xantl	Sinobac	Steroidobacter	0	0	0	0	0	0	1	0	0	0	0	0	1
Cluster29545	Acidobact	Acidobacteria_Gp1				1	0	0	0	0	0	0	0	0	0	0	0	1
Cluster29547	Proteobac	Alphap	Rhod	Acetobacteraceae		0	0	0	1	0	0	0	0	0	0	0	0	1
Cluster29558	Proteobac	Alphap	Alphaproteob		Rhizomicrobiu	0	0	0	0	1	0	0	0	0	0	0	0	1
Cluster29564	Chlorobi					0	0	1	0	0	0	0	0	0	0	0	0	1
Cluster29575	Bacteroid	Sphing	Sphir	Chitinophagaceae		0	0	1	0	0	0	0	0	0	0	0	0	1
Cluster29576	Verrucom	Subdivision3			Subdivision3_g	0	0	0	1	0	0	0	0	0	0	0	0	1
Cluster29581	Proteobac	Alphap	Rhizobiales			0	0	0	0	0	0	0	0	0	0	0	1	1
Cluster29585	Acidobact	Acidobacteria_Gp1				0	0	1	0	0	0	0	0	0	0	0	0	1
Cluster29590	Proteobac	Deltap	Myxc	Kofleria	Kofleria	0	0	1	0	0	0	0	0	0	0	0	0	1
Cluster29593	Proteobac	Betapr	Burkl	Burkholderiales_incert		0	0	1	0	0	0	0	0	0	0	0	0	1
Cluster29604	Proteobac	Alphap	Alphaproteob		Rhizomicrobiu	0	0	0	0	0	0	0	0	0	0	0	1	1
Cluster29614	Verrucom	Opituta	Opitu	Opituta	Opitutus	0	0	0	0	0	0	0	0	0	0	0	1	1
Cluster29615	Proteobac	Alphap	Rhizc	Bradyrhizobiaceae		0	0	0	0	0	0	0	1	0	0	0	0	1
Cluster29618	Acidobact	Acidobacteria_Gp3			Gp3	0	0	0	0	0	1	0	0	0	0	0	0	1
Cluster29621	Proteobac	Betapr	Burkl	Burkhol	Burkholderia	0	0	0	0	0	0	0	1	0	0	0	0	1
Cluster29625	Planctomy	Plancto	Planc	Planctomycetaceae		0	0	0	0	0	0	0	0	0	0	1	0	1

Cluster29633;size=1						0	0	1	0	0	0	0	0	0	0	0	0	1
Cluster29641;size=1						0	0	0	0	0	0	0	0	1	0	0	0	1
Cluster29643	Proteobac	Alphap	Caulc	Caulobacteraceae		0	0	1	0	0	0	0	0	0	0	0	0	1
Cluster29646	Acidobact	Acidobacteria_Gp3				0	0	0	0	1	0	0	0	0	0	0	0	1
Cluster29649;size=1						0	0	0	0	0	0	1	0	0	0	0	0	1
Cluster29650	Acidobact	Acidobacteria_Gp2			Gp2	0	0	0	0	0	0	0	1	0	0	0	0	1
Cluster29651	Actinobac	Actinol	Actinomycetales			0	0	0	0	0	0	1	0	0	0	0	0	1
Cluster29681	Proteobac	Alphap	Rhodospirillales			0	0	0	0	0	0	0	0	0	0	1	0	1
Cluster29686	Acidobacteria					0	0	0	1	0	0	0	0	0	0	0	0	1
Cluster29691	Acidobact	Acidobacteria_Gp1			Edaphobacter	1	0	0	0	0	0	0	0	0	0	0	0	1
Cluster29692;size=1						0	0	1	0	0	0	0	0	0	0	0	0	1
Cluster29696	Acidobact	Acidobacteria_Gp2			Gp2	0	0	0	0	0	0	0	0	1	0	0	0	1
Cluster29700	Proteobac	Alphap	Rhizobiales			0	1	0	0	0	0	0	0	0	0	0	0	1
Cluster29707	Chlamydia	Chlamy	Chlamydiales			0	0	0	0	0	0	0	0	1	0	0	0	1
Cluster29744	Tenericute	Mollicu	Entor	Spiropla	Spiroplasma	0	0	0	0	0	0	0	1	0	0	0	0	1
Cluster29754	Acidobact	Acidobacteria_Gp1				0	0	0	0	0	0	1	0	0	0	0	0	1
Cluster29757	Actinobac	Actinobacteria				0	0	0	0	0	0	1	0	0	0	0	0	1
Cluster29767	Acidobacteria					0	0	0	0	1	0	0	0	0	0	0	0	1
Cluster29771	Proteobacteria					0	0	0	0	0	0	0	1	0	0	0	0	1
Cluster29772	Proteobac	Deltap	Myxc	Polyangiaceae		0	0	0	0	1	0	0	0	0	0	0	0	1
Cluster29773	Acidobact	Acidobacteria_Gp1				0	0	0	0	1	0	0	0	0	0	0	0	1
Cluster29778	Proteobac	Alphap	Rhizc	Bradyrh	Bradyrhizobiur	0	0	0	0	0	0	0	0	1	0	0	0	1
Cluster29786	Actinobac	Actinol	Actinomycetales			0	0	0	0	0	0	0	0	0	0	1	0	1
Cluster29788	Proteobac	Betapr	Burkl	Burkhol	Burkholderia	0	0	0	1	0	0	0	0	0	0	0	0	1
Cluster29799	Acidobact	Acidobacteria_Gp2			Gp2	0	0	1	0	0	0	0	0	0	0	0	0	1
Cluster29802	Proteobac	Gamm	Xantl	Sinobac	Steroidobacter	0	0	0	0	0	0	0	0	0	0	1	0	1
Cluster29808	Proteobac	Alphap	Rhodospirillales			0	1	0	0	0	0	0	0	0	0	0	0	1
Cluster29814	Proteobac	Alphap	Rhizobiales			0	0	0	0	1	0	0	0	0	0	0	0	1
Cluster29816	Proteobac	Alphap	Rhizc	Xanthol	Labrys	0	0	0	0	0	0	0	0	0	0	0	1	1

Cluster29821;size=1						1	0	0	0	0	0	0	0	0	0	0	0	1
Cluster2982€	Proteobac	Alphap	Rhizobiales			0	0	0	1	0	0	0	0	0	0	0	0	1
Cluster29835;size=1						0	0	0	0	0	0	0	0	0	1	0	0	1
Cluster29837	Actinobac	Actinol	Actinomycetales			0	0	1	0	0	0	0	0	0	0	0	0	1
Cluster29843	Proteobac	Deltaproteobacteria				0	0	0	1	0	0	0	0	0	0	0	0	1
Cluster29844;size=1						0	0	0	0	1	0	0	0	0	0	0	0	1
Cluster2985C	Acidobact	Acidobacteria_Gp1				0	0	0	1	0	0	0	0	0	0	0	0	1
Cluster29863	Proteobac	Alphap	Rhodospirillales			0	0	0	0	0	0	1	0	0	0	0	0	1
Cluster29864;size=1						0	0	0	0	0	0	1	0	0	0	0	0	1
Cluster29867	Acidobact	Acidobacteria_Gp1				1	0	0	0	0	0	0	0	0	0	0	0	1
Cluster2986ε	Proteobac	Gamm	Xantl	Sinobac	Steroidobacter	0	0	1	0	0	0	0	0	0	0	0	0	1
Cluster29874	Acidobact	Acidobacteria_Gp1			Gp1	0	0	0	0	0	0	0	0	1	0	0	0	1
Cluster29877	Proteobac	Alphap	Rhodospirillales			0	0	1	0	0	0	0	0	0	0	0	0	1
Cluster2988€	Proteobac	Alphap	Rhod	Acetobacteraceae		0	0	0	0	0	0	0	0	0	0	0	1	1
Cluster29893	Proteobac	Betapr	Burkl	Burkhol	Burkholderia	0	0	0	0	0	0	0	0	0	0	1	0	1
Cluster29897	Proteobac	Alphaproteobacteria				0	0	0	0	1	0	0	0	0	0	0	0	1
Cluster2989ε	Actinobac	Actinol	Acidimicrobiales			0	0	0	0	0	0	0	1	0	0	0	0	1
Cluster29901	Acidobact	Acidobacteria_Gp1				0	0	0	0	0	0	0	1	0	0	0	0	1
Cluster29902	Acidobact	Acidobacteria_Gp2			Gp2	1	0	0	0	0	0	0	0	0	0	0	0	1
Cluster2990ε	Planctomy	Planctc	Planc	Planctomycetaceae		0	0	0	1	0	0	0	0	0	0	0	0	1
Cluster29913;size=1						0	1	0	0	0	0	0	0	0	0	0	0	1
Cluster29915	Proteobac	Alphaproteobacteria				1	0	0	0	0	0	0	0	0	0	0	0	1
Cluster2991ς	Proteobac	Alphap	Caulc	Caulobacteraceae		0	0	0	0	1	0	0	0	0	0	0	0	1
Cluster29925;size=1						0	0	0	0	0	0	0	0	0	0	0	1	1
Cluster2993€	Actinobac	Actinol	Actinomycetales			0	0	0	0	0	0	0	1	0	0	0	0	1
Cluster2993ε	Proteobac	Betapr	Burkl	Oxaloba	Herbaspirillum	0	0	1	0	0	0	0	0	0	0	0	0	1
Cluster29943	Bacteroid	Flavob	Flavo	Flavoba	Flavobacterium	0	0	0	0	0	0	1	0	0	0	0	0	1
Cluster29955	Proteobac	Alphap	Rhizobiales			0	0	0	0	0	0	0	0	0	0	0	1	1
Cluster29963	Acidobact	Acidobacteria_Gp2			Gp2	0	1	0	0	0	0	0	0	0	0	0	0	1

Cluster29964	Proteobac	Alphap	Rhod	Acetobacteraceae		0	0	0	0	1	0	0	0	0	0	0	0	1
Cluster29965	Actinobac	Actinol	Soliru	Conexib	Conexibacter	0	0	0	0	0	0	0	0	0	0	1	0	1
Cluster29971	Proteobac	Alphap	Rhodospirillales			0	0	1	0	0	0	0	0	0	0	0	0	1
Cluster29982	Proteobac	Alphap	Rhizc	Xanthol	Pseudolabrys	0	0	0	0	1	0	0	0	0	0	0	0	1
Cluster29994	Proteobac	Gammaproteobacteria				0	0	0	1	0	0	0	0	0	0	0	0	1
Cluster29998	Bacteroid	Sphing	Sphir	Chitinophagaceae		0	0	0	0	0	1	0	0	0	0	0	0	1
Cluster30004	Proteobac	Betapr	Burkl	Burkhol	Burkholderia	0	0	0	0	0	0	0	0	1	0	0	0	1
Cluster30006;size=2						0	0	0	0	0	0	0	0	1	0	0	0	1
Cluster30008	Proteobac	Alphap	Rhodospirillales			0	0	0	1	0	0	0	0	0	0	0	0	1
Cluster30011	Acidobact	Acidobacteria_Gp3			Gp3	0	0	0	0	1	0	0	0	0	0	0	0	1
Cluster30023	Actinobac	Actinol	Actinomycetales			0	0	0	1	0	0	0	0	0	0	0	0	1
Cluster30030	Proteobac	Alphap	Rhodospirillales			0	0	0	1	0	0	0	0	0	0	0	0	1
Cluster30033	Proteobac	Gammaproteobacteria				0	0	0	0	0	0	0	0	1	0	0	0	1
Cluster30037	Proteobac	Alphap	Rhodospirillales			0	1	0	0	0	0	0	0	0	0	0	0	1
Cluster30039	Armatimonadetes				Armatimonade	1	0	0	0	0	0	0	0	0	0	0	0	1
Cluster30044	Proteobac	Gamm	Legio	Coxiella	Aquicella	0	0	0	1	0	0	0	0	0	0	0	0	1
Cluster30062;size=1						0	0	0	0	0	0	0	1	0	0	0	0	1
Cluster30063	Proteobac	Gamm	Xantl	Sinobac	Steroidobacter	0	0	0	1	0	0	0	0	0	0	0	0	1
Cluster30070	Acidobact	Acidobacteria_Gp3			Gp3	0	0	0	0	1	0	0	0	0	0	0	0	1
Cluster30079	Planctomy	Planctc	Planc	Planctomycetaceae		0	0	0	0	0	0	0	0	0	0	1	0	1
Cluster30081	Proteobac	Alphap	Rhod	Acetoba	Acidisphaera	0	0	1	0	0	0	0	0	0	0	0	0	1
Cluster30091;size=1						1	0	0	0	0	0	0	0	0	0	0	0	1
Cluster30092	Actinobac	Actinol	Actinomycetales			0	0	0	0	0	0	0	0	0	0	1	0	1
Cluster30093	Proteobac	Deltaproteobacteria				0	1	0	0	0	0	0	0	0	0	0	0	1
Cluster30107;size=1						0	0	0	0	0	0	0	0	0	1	0	0	1
Cluster30109	Proteobac	Alphap	Rhizc	Xanthol	Pseudolabrys	0	0	0	0	0	0	0	0	0	0	1	0	1
Cluster30110	Acidobact	Acidobacteria_Gp2			Gp2	0	0	0	0	0	0	0	0	1	0	0	0	1
Cluster30115	Acidobact	Acidobacteria_Gp2			Gp2	0	0	0	0	1	0	0	0	0	0	0	0	1
Cluster30122	Proteobac	Gamm	Xantl	Sinobac	Steroidobacter	0	0	0	1	0	0	0	0	0	0	0	0	1

Cluster30127	Actinobac	Actinol	Actinomycetales			0	0	0	1	0	0	0	0	0	0	0	0	1
Cluster30135	Verrucom	Subdivision3			Subdivision3_g	0	0	0	0	0	0	0	0	0	0	0	1	1
Cluster30137	Firmicutes	Clostri	Clost	Lachnos	ClostridiumXlV	0	0	0	0	0	1	0	0	0	0	0	0	1
Cluster30139	Actinobac	Actinol	Actinomycetales			0	1	0	0	0	0	0	0	0	0	0	0	1
Cluster30142	Proteobac	Gammaproteobacteria				0	0	0	0	0	0	0	0	0	0	1	0	1
Cluster30144	Actinobac	Actinol	Actinomycetales			0	0	0	0	0	0	0	0	0	0	1	0	1
Cluster30145	Proteobac	Alphap	Sphir	Sphingomonadaceae		0	0	1	0	0	0	0	0	0	0	0	0	1
Cluster30147;size=4						0	0	0	0	1	0	0	0	0	0	0	0	1
Cluster30149	Proteobac	Alphap	Rhizobiales			0	0	0	1	0	0	0	0	0	0	0	0	1
Cluster30155	Actinobac	Actinol	Acidi	Acidimi	Aciditerrimona	0	0	0	0	0	0	0	0	0	1	0	0	1
Cluster30163	Proteobac	Alphap	Caulc	Caulobacteraceae		0	0	0	0	0	0	0	0	0	0	1	0	1
Cluster30170	Acidobact	Acidobacteria_Gp1				0	0	0	0	0	0	1	0	0	0	0	0	1
Cluster30172	Acidobacteria					0	0	0	0	0	0	0	0	0	0	0	1	1
Cluster30178	Actinobac	Actinol	Actinomycetales			0	0	0	0	0	0	0	0	0	0	1	0	1
Cluster30179	Planctomy	Plancto	Planc	Plancto	Zavarzinella	0	0	0	0	0	0	1	0	0	0	0	0	1
Cluster30191	Actinobac	Actinol	Actinomycetales			0	0	0	0	0	0	0	1	0	0	0	0	1
Cluster30192	Acidobact	Acidobacteria_Gp1				0	0	0	1	0	0	0	0	0	0	0	0	1
Cluster30193;size=1						1	0	0	0	0	0	0	0	0	0	0	0	1
Cluster30199	Acidobact	Acidobacteria_Gp2			Gp2	0	0	0	0	0	0	1	0	0	0	0	0	1
Cluster30205	Proteobac	Alphap	Rhizobiales			0	0	0	0	0	0	0	1	0	0	0	0	1

B/ Archaeal OTU

OTU	order	class	family	genus	H1R	E3BS	E4R	E4BS	H1BS	H2R	H2BS	H4R	H4BS	E2R	E2BS	E3R	sum
Otu01	Thaumarcha	unclassified	unclassified	unclassified	364	391	509	457	357	446	395	433	408	122	500	352	4734
Otu05	Thaumarcha	unclassified	unclassified	unclassified	196	296	306	254	186	211	173	266	239	61	217	251	2656
Otu03	Thaumarcha	unclassified	unclassified	unclassified	133	74	43	47	105	144	143	110	143	17	78	76	1113
Otu06	Thaumarcha	unclassified	unclassified	unclassified	35	46	7	20	45	11	27	24	46	2	20	49	332
Otu07	Thaumarcha	Nitrosopumil	Nitrosopumil	unclassified	70	9	31	13	66	30	30	16	15	17	27	7	331
Otu09	Euryarchaeot	Thermoplasn	Thermoplasn	Terrestrial_M	50	20	6	15	21	30	57	18	47	12	10	28	314
Otu08	Thaumarcha	Nitrosopumil	Nitrosopumil	unclassified	12	9	3	5	24	32	50	5	10	9	38	61	258
Otu04	Thaumarcha	unclassified	unclassified	unclassified	6	12	12	15	7	5	12	43	5	4	22	35	178
Otu02	Thaumarcha	unclassified	unclassified	unclassified	12	61	6	4	42	1	0	4	2	0	3	28	163
Otu21	Thaumarcha	unclassified	unclassified	unclassified	1	0	1	46	4	0	17	4	0	0	0	0	73
Otu11	Thaumarcha	unclassified	unclassified	unclassified	4	5	0	2	10	9	10	0	6	0	9	12	67
Otu12	Thaumarcha	unclassified	unclassified	unclassified	3	1	1	41	2	1	2	0	0	0	1	1	53
Otu25	Thaumarcha	unclassified	unclassified	unclassified	16	0	1	1	7	5	2	1	2	0	0	2	37
Otu19	Crenarchaeo	Thermoprote	Desulfurococ	unclassified	3	1	0	0	20	0	0	1	2	0	0	8	35
Otu26	Euryarchaeot	Methanomic	Methanosarc	Methanosarc	7	0	0	1	1	1	0	1	0	13	0	0	24
Otu18	Thaumarcha	Nitrosopumil	Nitrosopumil	unclassified	1	1	0	1	3	0	2	1	1	0	0	4	14
Otu22	Thaumarcha	unclassified	unclassified	unclassified	1	0	1	6	1	0	1	1	0	1	0	0	12
Otu16	Crenarchaeo	Thermoprote	Desulfurococ	unclassified	0	1	0	0	6	0	0	0	0	0	0	2	9
Otu20	Thaumarcha	unclassified	unclassified	unclassified	2	0	1	2	1	0	0	0	0	0	0	0	6
Otu44	Crenarchaeo	Thermoprote	unclassified	unclassified	0	0	0	0	3	0	0	0	1	0	0	1	5
Otu39	Thaumarcha	Nitrosopumil	Nitrosopumil	unclassified	1	0	0	0	0	1	0	0	0	1	1	0	4
Otu10	Thaumarcha	unclassified	unclassified	unclassified	1	1	0	0	0	0	1	1	0	0	0	0	4
Otu15	Thaumarcha	unclassified	unclassified	unclassified	0	1	2	0	0	0	0	1	0	0	0	0	4
Otu45	Crenarchaeo	Thermoprote	unclassified	unclassified	0	0	0	0	3	0	0	0	0	0	0	0	3
Otu57	Euryarchaeot	Thermoplasn	Thermoplasn	unclassified	0	0	0	0	1	0	0	0	0	0	1	1	3
Otu69	Thaumarcha	Nitrosopumil	Nitrosopumil	unclassified	0	0	0	0	0	0	2	0	0	0	0	1	3

Otu56	Thaumarchae	unclassified	unclassified	unclassified	0	0	0	0	1	0	2	0	0	0	0	0	3
Otu60	Thaumarchae	unclassified	unclassified	unclassified	0	0	0	0	0	1	0	0	0	0	1	1	3
Otu73	Thaumarchae	unclassified	unclassified	unclassified	0	0	0	0	0	0	0	0	2	0	0	1	3
Otu40	Crenarchaeo	Thermoprote	Desulfurococ	unclassified	1	0	0	0	1	0	0	0	0	0	0	0	2
Otu81	Euryarchaeot	Thermoplasm	Thermoplasm	unclassified	0	0	0	0	0	0	0	0	0	0	0	2	2
Otu29	Euryarchaeot	unclassified	unclassified	unclassified	2	0	0	0	0	0	0	0	0	0	0	0	2
Otu65	Euryarchaeot	unclassified	unclassified	unclassified	0	0	0	0	0	0	1	1	0	0	0	0	2
Otu33	Thaumarchae	unclassified	unclassified	unclassified	1	0	0	0	0	0	0	0	0	0	1	0	2
Otu43	Thaumarchae	unclassified	unclassified	unclassified	0	0	0	0	2	0	0	0	0	0	0	0	2
Otu48	Thaumarchae	unclassified	unclassified	unclassified	0	0	0	0	2	0	0	0	0	0	0	0	2
Otu62	Thaumarchae	unclassified	unclassified	unclassified	0	0	0	0	0	1	0	0	0	0	0	1	2
Otu64	Thaumarchae	unclassified	unclassified	unclassified	0	0	0	0	0	1	0	0	0	0	1	0	2
Otu84	Thaumarchae	unclassified	unclassified	unclassified	0	0	0	0	0	0	0	0	0	0	0	2	2
Otu34	Crenarchaeo	Thermoprote	Desulfurococ	unclassified	1	0	0	0	0	0	0	0	0	0	0	0	1
Otu49	Crenarchaeo	Thermoprote	unclassified	unclassified	0	0	0	0	1	0	0	0	0	0	0	0	1
Otu86	Crenarchaeo	Thermoprote	Desulfurococ	unclassified	0	0	0	0	0	0	0	0	0	0	0	1	1
Otu88	Crenarchaeo	Thermoprote	Desulfurococ	unclassified	0	0	0	0	0	0	0	0	0	0	0	1	1
Otu50	Euryarchaeot	Methanomic	Methanosarc	Methanosarc	0	0	0	0	1	0	0	0	0	0	0	0	1
Otu52	Euryarchaeot	Methanomic	Methanosarc	Methanosarc	0	0	0	0	1	0	0	0	0	0	0	0	1
Otu14	Euryarchaeot	Thermoplasm	Thermoplasm	unclassified	0	1	0	0	0	0	0	0	0	0	0	0	1
Otu17	Euryarchaeot	Thermoplasm	Thermoplasm	Terrestrial_M	0	1	0	0	0	0	0	0	0	0	0	0	1
Otu31	Euryarchaeot	Thermoplasm	Thermoplasm	unclassified	1	0	0	0	0	0	0	0	0	0	0	0	1
Otu41	Euryarchaeot	Thermoplasm	Thermoplasm	Terrestrial_M	1	0	0	0	0	0	0	0	0	0	0	0	1
Otu24	Euryarchaeot	unclassified	unclassified	unclassified	0	0	1	0	0	0	0	0	0	0	0	0	1
Otu30	Euryarchaeot	unclassified	unclassified	unclassified	1	0	0	0	0	0	0	0	0	0	0	0	1
Otu35	Euryarchaeot	unclassified	unclassified	unclassified	1	0	0	0	0	0	0	0	0	0	0	0	1
Otu77	Euryarchaeot	unclassified	unclassified	unclassified	0	0	0	0	0	0	0	0	0	1	0	0	1
Otu78	Euryarchaeot	unclassified	unclassified	unclassified	0	0	0	0	0	0	0	0	0	1	0	0	1
Otu79	Euryarchaeot	unclassified	unclassified	unclassified	0	0	0	0	0	0	0	0	0	0	1	0	1

Otu13	Thaumarcha	unclassified	unclassified	unclassified	0	1	0	0	0	0	0	0	0	0	0	0	1
Otu23	Thaumarcha	unclassified	unclassified	unclassified	0	0	1	0	0	0	0	0	0	0	0	0	1
Otu28	Thaumarcha	unclassified	unclassified	unclassified	0	0	0	1	0	0	0	0	0	0	0	0	1
Otu36	Thaumarcha	unclassified	unclassified	unclassified	1	0	0	0	0	0	0	0	0	0	0	0	1
Otu37	Thaumarcha	unclassified	unclassified	unclassified	1	0	0	0	0	0	0	0	0	0	0	0	1
Otu38	Thaumarcha	unclassified	unclassified	unclassified	1	0	0	0	0	0	0	0	0	0	0	0	1
Otu42	Thaumarcha	unclassified	unclassified	unclassified	1	0	0	0	0	0	0	0	0	0	0	0	1
Otu46	Thaumarcha	unclassified	unclassified	unclassified	0	0	0	0	1	0	0	0	0	0	0	0	1
Otu47	Thaumarcha	unclassified	unclassified	unclassified	0	0	0	0	1	0	0	0	0	0	0	0	1
Otu51	Thaumarcha	unclassified	unclassified	unclassified	0	0	0	0	1	0	0	0	0	0	0	0	1
Otu53	Thaumarcha	unclassified	unclassified	unclassified	0	0	0	0	1	0	0	0	0	0	0	0	1
Otu54	Thaumarcha	unclassified	unclassified	unclassified	0	0	0	0	1	0	0	0	0	0	0	0	1
Otu55	Thaumarcha	unclassified	unclassified	unclassified	0	0	0	0	1	0	0	0	0	0	0	0	1
Otu58	Thaumarcha	unclassified	unclassified	unclassified	0	0	0	0	1	0	0	0	0	0	0	0	1
Otu59	Thaumarcha	unclassified	unclassified	unclassified	0	0	0	0	1	0	0	0	0	0	0	0	1
Otu61	Thaumarcha	unclassified	unclassified	unclassified	0	0	0	0	0	1	0	0	0	0	0	0	1
Otu63	Thaumarcha	unclassified	unclassified	unclassified	0	0	0	0	0	1	0	0	0	0	0	0	1
Otu66	Thaumarcha	unclassified	unclassified	unclassified	0	0	0	0	0	0	1	0	0	0	0	0	1
Otu67	Thaumarcha	unclassified	unclassified	unclassified	0	0	0	0	0	0	1	0	0	0	0	0	1
Otu68	Thaumarcha	unclassified	unclassified	unclassified	0	0	0	0	0	0	1	0	0	0	0	0	1
Otu70	Thaumarcha	unclassified	unclassified	unclassified	0	0	0	0	0	0	1	0	0	0	0	0	1
Otu71	Thaumarcha	unclassified	unclassified	unclassified	0	0	0	0	0	0	1	0	0	0	0	0	1
Otu72	Thaumarcha	unclassified	unclassified	unclassified	0	0	0	0	0	0	0	1	0	0	0	0	1
Otu74	Thaumarcha	unclassified	unclassified	unclassified	0	0	0	0	0	0	0	0	1	0	0	0	1
Otu75	Thaumarcha	unclassified	unclassified	unclassified	0	0	0	0	0	0	0	0	1	0	0	0	1
Otu76	Thaumarcha	unclassified	unclassified	unclassified	0	0	0	0	0	0	0	0	1	0	0	0	1
Otu80	Thaumarcha	unclassified	unclassified	unclassified	0	0	0	0	0	0	0	0	0	0	1	0	1
Otu82	Thaumarcha	unclassified	unclassified	unclassified	0	0	0	0	0	0	0	0	0	0	0	1	1
Otu83	Thaumarcha	unclassified	unclassified	unclassified	0	0	0	0	0	0	0	0	0	0	0	1	1

Otu85	Thaumarchae	unclassified	unclassified	unclassified	0	0	0	0	0	0	0	0	0	0	0	1	1
Otu87	Thaumarchae	unclassified	unclassified	unclassified	0	0	0	0	0	0	0	0	0	0	0	1	1
Otu27	unclassified	unclassified	unclassified	unclassified	0	0	0	1	0	0	0	0	0	0	0	0	1
Otu32	-	-	-	-	1	0	0	0	0	0	0	0	0	0	0	0	1

C/ Fungal OTUs based on 18S rRNA sequences and associated statistics

OTUId	phylum	class	order	family	genus	specie	H1R	E3BS	E4R	E4BS	H1BS	H2R	H2BS	H4R	H4BS	E2R	E2BS	E3R	sum
Cluster129	Basidiomycot	Agar	Agarica	Amanita	Amanita	Amanita bru	3	14	29	8	26	9	7	10	20	19	18	10	173
Cluster776	Basidiomycot	Agar	Agarica	n	Inocybe	Inocybe aff. a	0	33	9	3	3	8	16	2	1	5	22	39	141
Cluster289	Basidiomycot	Agar	Agarica	Amanita	Amanita	Amanita bru	2	6	13	15	14	4	10	4	15	10	9	13	115
Cluster148	Basidiomycot	Agar	Agarica	n	Inocybe	Inocybe muta	1	18	6	5	3	12	14	0	2	5	12	36	114
Cluster673	Basidiomycot	Agar	Boletal	Sclerode	Scleroderm	Scleroderma	20	1	0	0	12	39	11	14	11	0	1	1	110
Cluster136	Basidiomycot	Agar	Athelia	Atheliac	Athelia	Athelia arach	2	6	28	54	1	0	1	4	0	1	9	1	107
Cluster66	Basidiomycot	Agar	Boletal	Sclerode	Scleroderm	Scleroderma	77	2	0	0	3	5	2	4	4	0	0	0	97
Cluster716	Basidiomycot	Agar	Boletal	Boletace	Xerocomell	Xerocomellus	1	7	11	5	7	4	1	4	1	21	20	5	87
Cluster711	Basidiomycot	Agar	Boletal	Sclerode	Scleroderm	Scleroderma	0	0	0	0	14	38	14	8	8	0	2	0	84
Cluster408	Basidiomycot	Agar	Russula	Russula	Russula	Russula exalb	5	2	2	1	5	8	9	18	21	4	3	0	78
Cluster847	Basidiomycot	Agar	Russula	Russula	Lactarius	Lactarius dec	0	7	5	1	9	3	8	17	21	3	0	1	75
Cluster234	Basidiomycot	Agar	Boletal	Boletace	Xerocomell	Xerocomellus	1	3	14	3	10	3	0	1	1	28	5	4	73
Cluster509	Basidiomycot	Agar	Agarica	Tricholo	Panellus	Panellus styp	3	3	6	1	9	7	1	15	20	3	0	5	73
Cluster351	Basidiomycot	Agar	Boletal	Sclerode	Scleroderm	Scleroderma	19	1	0	0	2	20	8	8	1	0	2	0	61
Cluster424	Basidiomycot	Agar	Agarica	n	Inocybe	Inocybe geop	0	1	0	1	4	24	21	1	3	0	2	1	58
Cluster508	Basidiomycot	Agar	Hymen	Hymeno	Phellinus	Phellinus igni	0	8	22	17	1	1	1	1	0	1	2	1	55
Cluster525	Basidiomycot	Agar	Agarica	n	Inocybe	Inocybe aff. a	0	12	0	8	0	4	4	1	1	2	13	9	54
Cluster59	Basidiomycot	Agar	Agarica	Amanita	Amanita	Amanita bru	30	1	1	7	3	1	1	1	1	4	2	1	53
Cluster450	Basidiomycot	Agar	Russula	Russula	Russula	Russula exalb	1	1	1	1	4	3	2	19	12	5	1	1	51
Cluster653	Basidiomycot	Agar	Agarica	Tricholo	Dendrocolly	Dendrocollyb	0	1	0	0	8	2	1	17	17	1	0	0	47
Cluster600	Ascomycota	Leoti	n	n	n	Leotiomycete	0	1	7	2	4	3	2	0	2	16	5	4	46
Cluster619	Basidiomycot	Agar	Agarica	n	Inocybe	Inocybe aff. a	0	15	4	1	1	4	10	1	1	2	2	5	46
Cluster268	n	n	Mortie	Mortier	Mortierella	Mortierella h	9	5	6	4	4	0	0	3	3	3	4	3	44
Cluster294	Basidiomycot	Agar	Agarica	n	Inocybe	Inocybe geop	0	2	1	0	0	13	15	1	2	1	2	6	43
Cluster522	Ascomycota	Leoti	Helotia	Bulgaria	Bulgaria	Bulgaria sp. F	1	1	6	5	7	2	2	2	1	5	5	1	38

Cluster287	Basidiomycot	Agar	Thelep	Theleph	Thelephora	Thelephora s	0	6	4	13	6	1	0	0	0	5	3	0	38
Cluster721	Basidiomycot	Agar	Russula	Russula	Lactarius	Lactarius dec	0	0	1	2	0	1	2	6	7	8	6	4	37
Cluster826	n	n	Mortie	Mortier	Mortierella	Mortierella h	1	3	4	7	1	1	5	4	2	3	4	2	37
Cluster63	Basidiomycot	Agar	Agarica	n	Inocybe	Inocybe aff. a	1	6	0	2	0	4	2	1	1	1	3	13	34
Cluster533	Basidiomycot	Agar	Agarica	n	Inocybe	Inocybe geop	3	0	0	0	4	8	14	1	3	0	1	0	34
Cluster141	Basidiomycot	Trem	Tremel	Tremella	Asterotrem	Asterotremel	0	3	5	7	4	0	1	0	2	4	5	2	33
Cluster162	Basidiomycot	Agar	Trechis	'Trechis	Trechispora	Trechispora a	2	6	1	2	1	1	2	1	2	1	2	11	32
Cluster64	Basidiomycot	Agar	Boletal	Boletace	Xerocomell	Xerocomellus	1	2	4	0	4	1	1	0	1	10	6	1	31
Cluster114	Basidiomycot	Agar	Russula	Russula	Russula	Russula comp	17	1	1	0	1	0	1	2	5	2	1	0	31
Cluster498	Basidiomycot	Agar	Boletal	n	Leucogyrop	Leucogyroph	0	2	2	2	4	0	0	1	1	6	10	3	31
Cluster503	Basidiomycot	Agar	Agarica	Cortinar	Cortinarius	Cortinarius ic	1	0	0	1	4	1	1	12	6	2	2	1	31
Cluster96	Basidiomycot	Agar	Jaapial	Jaapiace	Jaapia	Jaapia argilla	1	4	2	15	1	0	1	0	0	2	4	0	30
Cluster613	Basidiomycot	Agar	Agarica	n	Inocybe	Inocybe geop	0	0	0	0	0	7	15	4	3	0	1	0	30
Cluster170	Basidiomycot	Agar	Agarica	n	Inocybe	Inocybe aff. a	0	9	2	3	0	2	3	2	0	1	1	6	29
Cluster339	Basidiomycot	Agar	Boletal	Sclerode	Scleroderm	Scleroderma	13	0	0	0	3	6	3	2	1	0	1	0	29
Cluster353	Basidiomycot	Agar	Agarica	Amanita	Amanita	Amanita brur	1	2	4	4	1	1	3	1	2	3	5	2	29
Cluster130	Ascomycota	Doth	n	n	Cenococcur	Cenococcum	3	1	3	0	1	0	0	0	2	14	2	2	28
Cluster227	Basidiomycot	Agar	Russula	Russula	Russula	Russula exalb	3	0	0	0	4	0	3	5	9	0	0	4	28
Cluster823	Basidiomycot	Agar	Agarica	Tricholo	Camarophy	Camarophyllu	0	0	0	0	7	1	0	18	0	2	0	0	28
Cluster608	Chytridiomyc	Chyt	Chytrid	Chytridi	Chytriomyc	Chytriomyces	3	2	6	5	2	1	1	1	0	3	3	0	27
Cluster474	Apicomplexa	Cocc	Eucocc	Eimeriid	n	Eimeriidae sp	0	5	1	0	0	3	2	1	3	0	0	11	26
Cluster634	Ascomycota	Doth	n	n	Cenococcur	Cenococcum	1	0	3	1	3	0	2	2	3	6	3	1	25
Cluster667	Basidiomycot	Agar	Agarica	n	Inocybe	Inocybe aff. a	0	3	1	0	2	1	4	1	0	0	4	9	25
Cluster486	Basidiomycot	Agar	Agarica	Amanita	Amanita	Amanita brur	1	2	3	2	6	0	3	1	2	3	0	0	23
Cluster624	Basidiomycot	Agar	Russula	Russula	Russula	Russula exalb	3	0	3	0	1	3	3	1	6	3	0	0	23
Cluster73	Basidiomycot	Agar	Russula	Russula	Lactarius	Lactarius lign	5	2	1	1	1	1	0	1	4	3	1	2	22
Cluster277	Basidiomycot	Trem	Filobas	n	Cryptococci	Cryptococcus	0	1	0	3	2	1	1	0	0	7	5	2	22
Cluster320	Basidiomycot	Agar	Agarica	n	Inocybe	Inocybe aff. a	0	6	1	3	0	1	0	0	0	1	1	9	22
Cluster733	Basidiomycot	Agar	Agarica	Hygroph	Hygrophoru	Hygrophorus	0	3	3	4	0	1	2	0	0	1	5	3	22

Cluster103	Basidiomycot	Agar	Agarica	n	Inocybe	Inocybe geop	7	0	0	0	2	5	6	0	0	0	1	0	21
Cluster302	Basidiomycot	Agar	Agarica	n	Inocybe	Inocybe aff. a	0	6	0	2	1	3	0	0	0	0	2	7	21
Cluster662	Basidiomycot	Agar	Russula	Russula	Russula	Russula exalk	0	0	0	1	1	2	1	7	6	0	3	0	21
Cluster809	Basidiomycot	Agar	Agarica	n	Inocybe	Inocybe geop	0	0	0	0	4	1	11	2	1	1	1	0	21
Cluster393	Ascomycota	Peziz	Pezizal	Discinac	Gyromitra	Gyromitra es	0	0	0	0	0	0	1	7	12	0	0	0	20
Cluster497	Basidiomycot	Agar	Boletal	Sclerode	Scleroderm	Scleroderma	0	1	0	0	1	7	3	2	3	0	3	0	20
Cluster784	Basidiomycot	Agar	Agarica	Tricholo	Pleurocybel	Pleurocybella	1	1	0	0	2	2	3	1	9	0	0	1	20
Cluster396	Glomeromyc	Glon	Glome	Glomera	Glomus	Glomus sp. c	0	3	0	3	0	4	3	1	0	0	5	1	20
Cluster880	n	n	Mortie	Mortiere	Mortierella	Mortierella p	1	3	1	2	0	1	5	2	1	2	1	1	20
Cluster366	Ascomycota	n	n	n	n	ascomycete s	0	0	4	4	2	1	1	0	0	2	5	0	19
Cluster154	Basidiomycot	Agar	Agarica	Amanita	Amanita	Amanita brui	2	3	3	2	2	0	0	2	1	2	0	2	19
Cluster554	Basidiomycot	Agar	Agarica	Amanita	Amanita	Amanita brui	0	2	1	1	1	1	3	2	3	2	2	1	19
Cluster940	Basidiomycot	Agar	Agarica	Tricholo	Dendrocolly	Dendrocollyb	2	0	0	0	3	1	0	2	10	1	0	0	19
Cluster75	Basidiomycot	Agar	Agarica	Tricholo	Dendrocolly	Dendrocollyb	6	0	0	0	1	0	0	5	5	0	0	1	18
Cluster491	Basidiomycot	Agar	Agarica	Amanita	Amanita	Amanita brui	0	1	1	2	3	1	1	2	1	3	1	2	18
Cluster649	Basidiomycot	Agar	Boletal	Boletace	Xerocomell	Xerocomellus	0	1	0	2	0	1	0	0	0	5	9	0	18
Cluster195	n	n	Mortie	Mortiere	Dissophora	Dissophora d	5	2	0	0	1	0	0	2	0	4	3	1	18
Cluster179	Basidiomycot	Agar	Agarica	Tricholo	Sarcomyxa	Sarcomyxa se	0	1	4	0	0	0	1	4	1	0	2	4	17
Cluster382	Basidiomycot	Agar	Agarica	n	Inocybe	Inocybe geop	0	2	0	3	3	2	1	1	0	0	3	2	17
Cluster647	Basidiomycot	Agar	Athelia	Atheliac	Athelia	Athelia arach	0	0	5	5	0	0	2	1	0	0	4	0	17
Cluster969	n	n	Mortie	Mortiere	Mortierella	Mortierella p	0	2	2	4	4	0	1	1	1	0	1	1	17
Cluster987	n	n	Mortie	Mortiere	Dissophora	Dissophora d	2	0	4	1	3	2	3	0	0	0	2	0	17
Cluster194	Ascomycota	Sacc	Saccha	n	Candida	Candida sp. E	0	5	0	4	1	1	0	0	1	3	0	1	16
Cluster26	Basidiomycot	Agar	Russula	Russula	Russula	Russula exalk	6	0	1	0	1	1	1	1	2	1	1	1	16
Cluster283	Basidiomycot	Agar	Agarica	n	Inocybe	Inocybe aff. a	1	6	0	0	0	1	2	0	0	1	2	3	16
Cluster455	Basidiomycot	Agar	Agarica	Amanita	Amanita	Amanita brui	0	1	2	0	10	1	1	0	1	0	0	0	16
Cluster551	Basidiomycot	Agar	Jaapial	Jaapiace	Jaapia	Jaapia argilla	0	2	5	4	0	0	2	0	1	1	1	0	16
Cluster688	Basidiomycot	Agar	Russula	Russula	Lactarius	Lactarius lign	0	0	1	1	2	1	2	5	4	0	0	0	16
Cluster321	Ascomycota	Doth	n	n	Cenococcur	Cenococcum	1	0	2	0	2	0	0	1	2	1	3	3	15

Cluster164	Basidiomycot	Agar	Boletal	Boletace	Xerocomell	Xerocomellus	1	1	2	0	4	0	1	0	1	3	2	0	15
Cluster178	Ascomycota	Leoti	n	Myxotri	Byssoascus	Byssoascus st	2	0	1	1	0	0	0	2	0	6	1	1	14
Cluster602	Ascomycota	Leoti	Helotia	Helotiac	Hymenoscy	Hymenoscyp	0	2	1	1	2	1	1	0	0	2	3	1	14
Cluster67	Basidiomycot	Agar	Boletal	Boletace	Xerocomell	Xerocomellus	3	0	0	1	1	0	0	1	1	3	2	2	14
Cluster177	Basidiomycot	Agar	Agarica	n	Inocybe	Inocybe geop	0	0	0	1	2	8	2	0	0	0	1	0	14
Cluster183	Basidiomycot	Agar	Russula	Russulac	Russula	Russula exalb	2	1	0	0	1	0	1	7	0	1	1	0	14
Cluster303	n	n	Mucora	n	Umbelopsis	Umbelopsis r	1	4	1	1	3	0	0	1	1	1	0	1	14
Cluster638	n	n	Mortie	Mortiere	Mortierella	Mortierella p	1	1	0	3	4	1	1	0	2	0	1	0	14
Cluster117	Apicomplexa	Cocc	Eucocc	Eimeriid	n	Eimeriidae sp	1	4	2	0	0	1	2	0	2	0	0	1	13
Cluster186	Basidiomycot	Agar	Boletal	Sclerode	Scleroderm	Scleroderma	2	0	0	0	1	1	2	5	1	0	1	0	13
Cluster190	Basidiomycot	Agar	Agarica	Tricholo	Laccaria	Laccaria ame	1	0	0	1	2	0	1	4	3	1	0	0	13
Cluster260	Basidiomycot	Agar	Russula	Russulac	Lactarius	Lactarius lign	0	0	1	2	0	1	0	4	0	3	1	1	13
Cluster539	Basidiomycot	Agar	Boletal	Sclerode	Scleroderm	Scleroderma	0	0	0	0	2	5	4	2	0	0	0	0	13
Cluster757	Basidiomycot	Agar	Agarica	n	Inocybe	Inocybe aff. a	0	6	1	0	1	2	1	0	0	0	1	1	13
Cluster204	n	n	Mortie	Mortiere	Mortierella	Mortierella s	3	3	0	0	1	0	0	1	2	0	1	2	13
Cluster913	n	n	Mortie	Mortiere	Mortierella	Mortierella p	0	0	0	2	1	2	4	0	1	3	0	0	13
Cluster986	n	n	Mortie	Mortiere	Mortierella	Mortierella s	0	1	1	1	1	2	2	0	0	0	2	3	13
Cluster76	Ascomycota	Peziz	Pezizal	Discinac	Gyromitra	Gyromitra es	5	0	0	0	2	0	1	1	2	0	0	1	12
Cluster65	Basidiomycot	Agar	Agarica	Tricholo	Pleurocybel	Pleurocybella	9	0	0	0	1	0	0	0	2	0	0	0	12
Cluster107	Basidiomycot	Agar	Agarica	n	Inocybe	Inocybe aff. a	0	8	0	0	0	1	0	0	0	0	0	3	12
Cluster161	Basidiomycot	n	Leucos	Leucosp	Leucosporic	Leucosporidi	0	5	0	1	0	0	0	0	1	2	2	1	12
Cluster243	Basidiomycot	Agar	Sebacin	Sebacina	Craterocolla	Craterocolla c	0	1	0	3	4	1	0	0	0	2	1	0	12
Cluster401	Basidiomycot	Trem	Tremel	Tremella	Asterotrem	Asterotremel	1	2	0	2	2	1	0	0	1	2	1	0	12
Cluster717	Basidiomycot	Agar	Agarica	n	Inocybe	Inocybe aff. a	0	1	1	0	0	0	0	1	1	1	1	6	12
Cluster217	Chytridiomyc	Chyt	Chytrid	Chytridi	Chytriomyc	Chytriomyces	4	0	4	0	2	0	0	0	0	2	0	0	12
Cluster256	n	n	Mortie	Mortiere	Mortierella	Mortierella h	2	2	0	1	0	0	0	1	0	0	2	4	12
Cluster44	Basidiomycot	Agar	Agarica	n	Inocybe	Inocybe geop	2	0	0	0	0	1	6	1	1	0	0	0	11
Cluster52	Basidiomycot	Agar	Russula	Russulac	Russula	Russula exalb	6	0	0	0	1	0	0	0	4	0	0	0	11
Cluster97	Basidiomycot	Agar	Agarica	Amanita	Amanita	Amanita brun	2	0	1	2	2	0	1	1	0	0	2	0	11

Cluster278	Basidiomycot	Trem	Filobas	n	Cryptococci	Cryptococcus	0	2	0	1	2	0	0	0	0	2	3	1	11
Cluster352	Basidiomycot	Agar	Boletal	Sclerode	Scleroderm	Scleroderma	3	0	0	0	0	5	1	1	1	0	0	0	11
Cluster384	Basidiomycot	Agar	Agarica	Tricholo	Camarophy	Camarophyllu	0	1	0	0	1	1	0	7	1	0	0	0	11
Cluster492	Basidiomycot	Agar	Agarica	n	Inocybe	Inocybe geop	0	0	0	0	2	4	4	0	1	0	0	0	11
Cluster655	Basidiomycot	Trem	Tremel	Tremella	Asterotrem	Asterotremel	0	0	0	1	1	1	1	1	0	3	2	1	11
Cluster781	Basidiomycot	Agar	Agarica	n	Inocybe	Inocybe aff. a	0	3	0	0	2	0	1	1	0	0	3	1	11
Cluster787	Basidiomycot	Agar	Agarica	n	Inocybe	Inocybe aff. a	0	1	0	3	0	0	2	0	0	0	4	1	11
Cluster842	Basidiomycot	Agar	Boletal	Boletace	Xerocomell	Xerocomellus	0	0	1	0	2	1	0	0	0	4	2	1	11
Cluster56	Ascomycota	Leoti	n	Ascodicl	Pseudopha	Pseudophaci	3	0	1	0	0	0	3	1	0	1	0	1	10
Cluster68	Ascomycota	Leoti	n	Myxotri	Byssoascus	Byssoascus st	1	1	2	1	2	0	0	0	0	3	0	0	10
Cluster315	Ascomycota	Sord	Hypocr	Hypocre	Hypomyces	Hypomyces c	0	0	0	2	1	0	0	1	0	6	0	0	10
Cluster964	Ascomycota	Arch	Archae	Archaeo	Archaeorhiz	Archaeorhizo	0	1	6	3	0	0	0	0	0	0	0	0	10
Cluster16	Basidiomycot	Agar	Agarica	Amanita	Amanita	Amanita brur	5	0	1	2	1	0	0	0	0	0	0	1	10
Cluster128	Basidiomycot	Agar	Agarica	Tricholo	Dendrocolly	Dendrocollyb	4	0	0	0	1	0	0	2	3	0	0	0	10
Cluster150	Basidiomycot	Agar	Agarica	Tricholo	Panellus	Panellus styp	0	0	0	1	0	1	0	0	3	0	0	5	10
Cluster212	Basidiomycot	Agar	Agarica	Amanita	Amanita	Amanita mus	0	1	0	1	1	0	0	0	0	7	0	0	10
Cluster265	Basidiomycot	Agar	Agarica	n	Inocybe	Inocybe geop	0	0	0	1	0	4	4	1	0	0	0	0	10
Cluster314	Basidiomycot	Trem	Cystofi	Cystofilo	Mrakia	Mrakia frigid	0	7	0	0	0	0	0	0	1	0	1	1	10
Cluster409	Basidiomycot	Agar	Russula	Russulac	Lactarius	Lactarius lign	0	0	2	1	0	0	0	0	0	5	2	0	10
Cluster510	Basidiomycot	Agar	Agarica	n	Inocybe	Inocybe geop	0	1	0	0	1	4	4	0	0	0	0	0	10
Cluster775	Basidiomycot	Agar	Agarica	Amanita	Amanita	Amanita brur	3	2	1	0	1	0	0	1	1	0	0	1	10
Cluster840	Basidiomycot	Agar	Thelep	Theleph	Thelephora	Thelephora s	0	1	2	5	0	0	0	0	0	0	2	0	10
Cluster370	n	n	Mortie	Mortiere	Mortierella	Mortierella p	1	1	0	2	4	0	0	0	0	0	1	1	10
Cluster400	Apicomplexa	Cocc	Eucocc	Eimeriid	n	Eimeriidae sp	1	3	0	1	1	1	0	0	2	0	0	0	9
Cluster404	Ascomycota	Sord	Ophios	Ophiost	Grosmannia	Grosmannia	0	2	1	0	0	0	0	0	0	1	4	1	9
Cluster635	Ascomycota	Saccl	Saccha	n	Candida	Candida sp. E	0	2	1	0	1	0	0	0	0	3	2	0	9
Cluster751	Ascomycota	Leoti	n	Rhytism	Rhytisma	Rhytisma ace	0	0	0	0	0	0	0	0	0	0	6	3	9
Cluster90	Basidiomycot	Agar	Russula	Russulac	Russula	Russula exalb	2	0	0	0	2	1	1	1	2	0	0	0	9
Cluster99	Basidiomycot	Agar	Agarica	Bolbitiac	Bolbitius	Bolbitius vite	4	0	0	0	0	0	1	1	1	1	1	0	9

Cluster331	Basidiomycot	Agar	Agarica	Hygroph	Humidicutis	Humidicutis r	0	1	0	0	0	0	1	1	0	0	1	5	9
Cluster363	Basidiomycot	Agar	Agarica	n	Inocybe	Inocybe aff. a	0	1	0	0	2	1	1	0	0	0	1	3	9
Cluster388	Basidiomycot	Agar	Agarica	Bolbitia	Bolbitius	Bolbitius vite	0	0	0	0	0	1	3	4	0	1	0	0	9
Cluster440	Basidiomycot	Agar	Agarica	Tricholo	Camarophy	Camarophyllu	0	0	0	1	2	0	0	4	2	0	0	0	9
Cluster502	Basidiomycot	Agar	Agarica	n	Inocybe	Inocybe aff. a	0	2	1	1	0	0	1	0	0	0	0	4	9
Cluster679	Basidiomycot	Agar	Agarica	Amanita	Amanita	Amanita brui	1	2	0	1	1	0	0	1	0	2	0	1	9
Cluster762	Basidiomycot	Agar	Boletal	Boletace	Xerocomell	Xerocomellus	0	0	1	0	0	2	0	0	0	3	3	0	9
Cluster1013	n	n	Mortie	Mortier	Dissophora	Dissophora d	0	0	0	0	2	0	2	1	2	0	2	0	9
Cluster241	Ascomycota	Sord	Hypocr	Hypocre	Hypocrea	Hypocrea kor	2	0	0	0	0	3	0	0	1	1	0	1	8
Cluster819	Ascomycota	Sord	Hypocr	Cordycip	Isaria	Isaria javanic	0	0	1	1	2	0	0	0	0	3	1	0	8
Cluster19	Basidiomycot	Agar	Agarica	Amanita	Amanita	Amanita brui	2	0	0	0	3	1	1	0	0	1	0	0	8
Cluster70	Basidiomycot	Agar	Athelia	Atheliac	Athelia	Athelia arach	0	1	3	3	0	0	0	0	0	0	1	0	8
Cluster120	Basidiomycot	Agar	Agarica	Tricholo	Melanoleuc	Melanoleuca	0	0	0	2	0	0	0	0	0	1	0	5	8
Cluster296	Basidiomycot	Agar	Agarica	Tricholo	Collybia	Collybia tube	0	1	0	0	0	1	0	5	1	0	0	0	8
Cluster585	Basidiomycot	Agar	Boletal	Boletace	Boletus	Boletus eduli	0	1	2	0	0	0	1	0	0	3	0	1	8
Cluster637	Basidiomycot	Agar	Agarica	Tricholo	Dendrocolly	Dendrocollyb	0	0	0	0	1	0	1	3	2	0	0	1	8
Cluster651	Basidiomycot	Agar	Agarica	Tricholo	Dendrocolly	Dendrocollyb	0	0	0	0	0	0	0	8	0	0	0	0	8
Cluster390	n	n	Mortie	Mortier	Dissophora	Dissophora d	4	0	0	2	1	0	0	1	0	0	0	0	8
Cluster444	n	n	Mortie	Mortier	Mortierella	Mortierella h	1	1	0	1	0	1	3	0	0	1	0	0	8
Cluster246	Annelida	Poly	Phylloc	Syllidae	Typosyllis	Typosyllis bel	0	0	1	4	1	1	0	0	0	0	0	0	7
Cluster45	Ascomycota	Leoti	n	Myxotri	Byssoascus	Byssoascus st	2	0	1	1	0	0	0	0	2	0	0	1	7
Cluster527	Ascomycota	Sord	Hypocr	Ophiocc	Ophiocordy	Ophiocordyc	0	0	2	0	0	0	1	1	0	3	0	0	7
Cluster9	Basidiomycot	Agar	Agarica	Tricholo	Dendrocolly	Dendrocollyb	3	0	0	0	1	0	0	0	3	0	0	0	7
Cluster38	Basidiomycot	Agar	Athelia	Atheliac	Athelia	Athelia arach	0	1	0	4	0	0	0	0	0	0	1	1	7
Cluster71	Basidiomycot	Agar	Athelia	Atheliac	Amphinema	Amphinema	0	1	3	1	0	0	0	1	0	0	1	0	7
Cluster102	Basidiomycot	Agar	Boletal	Sclerode	Scleroderm	Scleroderma	4	0	0	0	0	0	1	0	1	0	0	1	7
Cluster106	Basidiomycot	Agar	Agarica	n	Inocybe	Inocybe aff. a	1	1	0	0	0	0	1	0	0	1	0	3	7
Cluster108	Basidiomycot	Agar	Agarica	Tricholo	Laccaria	Laccaria ame	3	0	0	0	1	1	0	1	1	0	0	0	7
Cluster132	Basidiomycot	Agar	Agarica	Amanita	Amanita	Amanita brui	1	0	3	0	2	0	0	0	0	0	0	1	7

Cluster206	Basidiomycot	Agar	Russula	Russulac	Lactarius	Lactarius lign	0	1	0	0	0	0	2	1	2	0	0	1	7
Cluster215	Basidiomycot	Agar	Boletal	Sclerode	Scleroderm	Scleroderma	4	0	0	0	0	3	0	0	0	0	0	0	7
Cluster248	Basidiomycot	Agar	Boletal	Boletace	Xerocomell	Xerocomellus	0	0	2	0	1	0	0	0	0	3	0	1	7
Cluster313	Basidiomycot	Agar	Polypo	Polypora	Trichaptum	Trichaptum b	0	1	3	0	0	0	0	0	0	0	1	2	7
Cluster494	Basidiomycot	Agar	Boletal	Sclerode	Scleroderm	Scleroderma	0	0	0	0	1	4	0	2	0	0	0	0	7
Cluster595	Basidiomycot	Agar	Agarica	n	Inocybe	Inocybe geop	0	2	0	0	1	0	2	0	0	0	1	1	7
Cluster603	Basidiomycot	Agar	Agarica	n	Inocybe	Inocybe aff. a	0	1	1	0	0	1	2	0	0	0	1	1	7
Cluster620	Basidiomycot	Agar	Agarica	Lyophyll	Asterophora	Asterophora	0	4	0	0	0	0	0	0	0	0	0	3	7
Cluster656	Basidiomycot	Agar	Agarica	Tricholo	Camarophy	Camarophyllu	1	0	1	0	1	0	0	3	1	0	0	0	7
Cluster185	n	n	Mortie	Mortiere	Mortierella	Mortierella a	2	1	0	0	0	0	1	2	0	0	1	0	7
Cluster207	Ascomycota	Leoti	n	Myxotri	Byssoascus	Byssoascus st	0	2	0	0	1	0	0	0	1	0	2	0	6
Cluster293	Ascomycota	Euro	Eurotia	Trichocc	Penicillium	Penicillium h	0	1	1	0	1	1	0	0	0	2	0	0	6
Cluster299	Ascomycota	Leoti	n	Myxotri	Byssoascus	Byssoascus st	0	1	1	1	0	0	0	2	0	0	1	0	6
Cluster505	Ascomycota	Doth	n	n	Cenococcum	Cenococcum	0	0	0	1	0	0	0	0	0	2	2	1	6
Cluster85	Basidiomycot	Agar	Agarica	n	Inocybe	Inocybe geop	2	1	0	0	1	2	0	0	0	0	0	0	6
Cluster133	Basidiomycot	Agar	Boletal	Boletace	Boletus	Boletus eduli	0	2	3	1	0	0	0	0	0	0	0	0	6
Cluster171	Basidiomycot	Agar	Russula	Russulac	Russula	Russula exalb	3	0	0	0	0	1	0	1	1	0	0	0	6
Cluster203	Basidiomycot	Agar	Russula	Russulac	Russula	Russula exalb	0	1	0	1	1	1	0	1	1	0	0	0	6
Cluster239	Basidiomycot	Agar	Boletal	n	Leucogyrop	Leucogyroph	0	0	1	1	0	1	0	0	0	2	0	1	6
Cluster326	Basidiomycot	Agar	Boletal	Boletace	Xerocomell	Xerocomellus	0	0	2	2	0	0	0	0	0	1	1	0	6
Cluster520	Basidiomycot	Agar	Agarica	Amanita	Amanita	Amanita aff.	0	0	1	0	2	0	0	1	0	1	0	1	6
Cluster562	Basidiomycot	Agar	Agarica	n	Inocybe	Inocybe geop	0	1	0	0	0	1	3	0	1	0	0	0	6
Cluster682	Basidiomycot	Agar	Agarica	Tricholo	Dendrocolly	Dendrocollyb	0	0	0	1	1	1	0	2	1	0	0	0	6
Cluster811	Basidiomycot	Agar	Agarica	Tricholo	Melanoleuc	Melanoleuca	0	0	0	0	0	1	0	0	0	1	1	3	6
Cluster812	Basidiomycot	Agar	Sebacin	Sebacina	Craterocolla	Craterocolla	0	1	0	0	0	1	0	0	0	2	1	1	6
Cluster879	Basidiomycot	Agar	Russula	Russulac	Russula	Russula exalb	0	0	0	0	0	0	1	4	1	0	0	0	6
Cluster1030	Basidiomycot	Agar	Agarica	n	Inocybe	Inocybe aff. a	0	0	0	0	0	1	1	0	0	1	1	2	6
Cluster274	Glomeromyc	Glon	Diversi	Acaulos	Acaulospora	Acaulospora	0	0	0	0	1	1	0	0	0	4	0	0	6
Cluster292	n	Chry	Chrom	Chromu	n	Spumella-like	0	0	1	3	0	1	0	0	0	1	0	0	6

Cluster454	n	n	Mortie	Mortier	Gamsiella	Gamsiella mu	1	1	0	0	0	2	1	0	1	0	0	0	6
Cluster36	Ascomycota	n	n	n	n	ascomycete s	1	0	0	2	0	0	0	1	0	1	0	0	5
Cluster54	Ascomycota	Doth	n	n	Cenococcur	Cenococcum	0	0	1	0	2	0	0	0	0	2	0	0	5
Cluster109	Ascomycota	Sacc	Saccha	n	Candida	Candida sp. E	0	1	0	0	1	0	0	0	0	1	2	0	5
Cluster173	Ascomycota	Doth	n	n	Cenococcur	Cenococcum	0	0	0	0	0	1	0	0	0	4	0	0	5
Cluster295	Ascomycota	Peziz	Pezizal	Pyroner	Scutellinia	Scutellinia sc	0	2	0	3	0	0	0	0	0	0	0	0	5
Cluster298	Ascomycota	Sord	Sordari	Chaetor	Chaetomiur	Chaetomium	0	0	0	0	2	0	0	0	0	2	1	0	5
Cluster358	Ascomycota	Arch	Archae	Archaec	Archaeorhi	Archaeorhizc	0	0	1	3	0	0	0	0	0	0	0	1	5
Cluster786	Ascomycota	Arch	Archae	Archaec	Archaeorhi	Archaeorhizc	0	0	4	1	0	0	0	0	0	0	0	0	5
Cluster22	Basidiomycot	Agar	Agarica	Amanita	Amanita	Amanita brui	4	0	0	0	0	0	1	0	0	0	0	0	5
Cluster30	Basidiomycot	Agar	Agarica	n	Inocybe	Inocybe aff. a	1	1	0	0	0	0	0	0	0	0	1	2	5
Cluster78	Basidiomycot	Agar	Sebacir	Sebacin	Sebacina	Sebacina ver	0	0	2	3	0	0	0	0	0	0	0	0	5
Cluster153	Basidiomycot	Agar	Agarica	Amanita	Amanita	Amanita aff.	0	2	1	1	1	0	0	0	0	0	0	0	5
Cluster191	Basidiomycot	Agar	Cantha	Botryob	Botryobasic	Botryobasidi	0	1	1	0	0	0	0	0	0	2	1	0	5
Cluster360	Basidiomycot	Agar	Agarica	Tricholo	Melanoleuc	Melanoleuca	0	1	1	0	0	0	0	0	0	0	0	3	5
Cluster367	Basidiomycot	Agar	Agarica	Lyophyll	Asterophor	Asterophora	0	3	0	0	0	0	0	0	0	0	0	2	5
Cluster402	Basidiomycot	Agar	Boletal	Boletace	Xerocomell	Xerocomellus	0	1	0	0	1	0	1	0	0	2	0	0	5
Cluster410	Basidiomycot	Agar	Boletal	Boletace	Xerocomell	Xerocomellus	0	1	1	0	0	1	0	0	0	2	0	0	5
Cluster456	Basidiomycot	Agar	Agarica	Tricholo	Melanoleuc	Melanoleuca	0	1	0	1	0	0	0	0	0	0	0	3	5
Cluster478	Basidiomycot	Agar	Agarica	Tricholo	Dendrocolly	Dendrocollyb	0	0	0	0	0	0	0	2	3	0	0	0	5
Cluster496	Basidiomycot	Agar	Agarica	Lyophyll	Asterophor	Asterophora	0	3	0	0	0	0	0	0	0	0	0	2	5
Cluster511	Basidiomycot	Agar	Russula	Russulac	Lactarius	Lactarius lign	0	0	0	0	0	0	0	2	1	2	0	0	5
Cluster559	Basidiomycot	Agar	Agarica	Amanita	Amanita	Amanita brui	0	0	0	2	0	0	1	0	1	0	0	1	5
Cluster561	Basidiomycot	Agar	Agarica	n	Inocybe	Inocybe aff. a	0	1	1	0	0	0	0	0	0	0	1	2	5
Cluster700	Basidiomycot	Agar	Boletal	Boletace	Xerocomell	Xerocomellus	0	1	0	2	0	0	0	0	0	0	2	0	5
Cluster703	Basidiomycot	Agar	Boletal	Coniopł	Coniophora	Coniophora p	0	0	1	2	0	0	0	0	0	0	1	1	5
Cluster865	Basidiomycot	Agar	Agarica	Tricholo	Laccaria	Laccaria ame	0	0	0	0	1	0	0	3	1	0	0	0	5
Cluster941	Basidiomycot	Agar	Agarica	Tricholo	Panellus	Panellus styp	0	0	0	1	0	0	0	2	2	0	0	0	5
Cluster140	n	n	Endogo	Endogoi	Endogone	Endogone pis	0	1	0	0	0	1	0	0	1	1	0	1	5

Cluster174	n	n	Mortie	Mortier	Mortierella	Mortierella h	0	1	0	1	0	0	0	0	1	1	1	0	5
Cluster349	n	n	Mortie	Mortier	Mortierella	Mortierella s	1	0	0	0	0	0	0	1	2	0	0	1	5
Cluster696	Apicomplexa	Cocc	Eucocc	Eimeriid	n	Eimeriidae sp	0	0	1	0	0	1	1	0	0	0	0	1	4
Cluster1019	Apicomplexa	Cocc	Eucocc	Eimeriid	n	Eimeriidae sp	0	1	0	0	0	0	2	0	0	0	0	1	4
Cluster113	Ascomycota	Leca	n	Briganti	Brigantiaea	Brigantiaea c	0	0	0	0	0	0	0	0	0	1	2	1	4
Cluster172	Ascomycota	Leoti	Helotia	n	Amylocarpu	Amylocarpus	0	1	0	0	0	1	0	2	0	0	0	0	4
Cluster181	Ascomycota	Euro	Chaeto	Herpotr	Capronia	Capronia fun	0	0	0	0	0	0	0	0	0	2	1	1	4
Cluster245	Ascomycota	Leoti	n	Myxotri	Pseudogym	Pseudogymn	0	0	2	0	0	0	0	0	0	0	1	1	4
Cluster264	Ascomycota	Doth	n	n	Cenococcur	Cenococcum	1	0	1	0	0	0	1	1	0	0	0	0	4
Cluster345	Ascomycota	Euro	Eurotia	Trichocc	Byssochlam	Byssochlamy	0	1	0	1	2	0	0	0	0	0	0	0	4
Cluster898	Ascomycota	Leoti	n	Myxotri	Byssoascus	Byssoascus st	0	0	0	0	1	0	0	0	0	1	2	0	4
Cluster28	Basidiomycot	Agar	Agarica	n	Inocybe	Inocybe aff. a	0	2	0	0	0	1	1	0	0	0	0	0	4
Cluster39	Basidiomycot	Agar	Boletal	Sclerode	Scleroderm	Scleroderma	3	0	0	0	0	1	0	0	0	0	0	0	4
Cluster48	Basidiomycot	Agar	Boletal	Sclerode	Scleroderm	Scleroderma	3	0	0	0	0	1	0	0	0	0	0	0	4
Cluster57	Basidiomycot	Agar	Agarica	Tricholo	Laccaria	Laccaria ame	3	0	0	0	0	0	0	0	1	0	0	0	4
Cluster77	Basidiomycot	Agar	Agarica	n	Inocybe	Inocybe geop	1	0	0	0	0	2	1	0	0	0	0	0	4
Cluster202	Basidiomycot	Agar	Russula	Russula	Russula	Russula exalb	1	0	1	0	0	1	0	0	1	0	0	0	4
Cluster205	Basidiomycot	n	Helicob	n	Helicobasid	Helicobasidiu	0	0	2	0	0	0	0	0	0	2	0	0	4
Cluster209	Basidiomycot	Agar	Agarica	Tricholo	Laccaria	Laccaria ame	0	0	0	0	1	1	0	2	0	0	0	0	4
Cluster216	Basidiomycot	n	n	n	Marchandic	Marchandior	0	0	0	0	0	0	1	1	0	0	0	2	4
Cluster412	Basidiomycot	Agar	Agarica	n	Inocybe	Inocybe aff. a	0	1	0	0	1	0	0	0	0	1	0	1	4
Cluster484	Basidiomycot	Agar	Athelia	Atheliac	Athelia	Athelia arach	0	0	1	3	0	0	0	0	0	0	0	0	4
Cluster604	Basidiomycot	Agar	Polypo	Pterulac	Pterula	Pterula echo	1	1	0	0	0	0	0	2	0	0	0	0	4
Cluster628	Basidiomycot	Agar	Agarica	n	Inocybe	Inocybe geop	0	0	0	1	0	2	1	0	0	0	0	0	4
Cluster764	Basidiomycot	Agar	Boletal	Boletace	Chalciporus	Chalciporus p	0	1	1	0	1	0	0	0	0	1	0	0	4
Cluster857	Basidiomycot	Agar	Jaapial	Jaapiace	Jaapia	Jaapia argilla	0	2	1	0	0	0	0	0	0	0	1	0	4
Cluster900	Basidiomycot	Agar	Jaapial	Jaapiace	Jaapia	Jaapia argilla	0	1	0	1	0	0	0	0	0	0	2	0	4
Cluster924	Basidiomycot	Agar	Boletal	Boletace	Xerocomell	Xerocomellus	0	0	0	0	0	0	0	1	0	2	1	0	4
Cluster927	Basidiomycot	Agar	Boletal	Boletace	Xerocomell	Xerocomellus	0	0	0	0	0	1	0	0	0	2	1	0	4

Cluster961	Basidiomycot	Agar	Jaapial	Jaapiace	Jaapia	Jaapia argilla	0	0	1	3	0	0	0	0	0	0	0	0	4
Cluster836	Cryptomycot	n	n	n	Rozella	Rozella allom	0	0	0	0	0	1	3	0	0	0	0	0	4
Cluster144	n	n	Kickxel	Kickxella	Mycoemilia	Mycoemilia s	0	0	0	0	2	1	0	1	0	0	0	0	4
Cluster262	n	n	n	Nuclear	Nuclearia	Nuclearia the	0	0	0	3	0	1	0	0	0	0	0	0	4
Cluster420	n	Spiro	Sporad	Oxytrich	Protogastro	Protogastros	0	0	1	2	0	0	0	0	0	0	1	0	4
Cluster566	n	n	Mortie	Mortier	Mortierella	Mortierella s	0	0	0	0	2	0	1	0	0	0	1	0	4
Cluster878	n	Spiro	Sporad	Oxytrich	Protogastro	Protogastros	0	0	2	1	0	0	1	0	0	0	0	0	4
Cluster197	Nematoda	Chro	Araeola	Rhabdol	Rhabdolaim	Rhabdolaimu	0	0	0	1	0	1	0	1	0	0	1	0	4
Cluster980	Apicomplexa	Cocc	Eucocc	Eimeriid	n	Eimeriidae sp	0	0	0	1	1	1	0	0	0	0	0	0	3
Cluster127	Ascomycota	Leoti	n	Myxotri	Byssoascus	Byssoascus st	2	0	0	0	0	0	0	0	0	0	0	1	3
Cluster145	Ascomycota	Sord	Glomer	Plectosp	Plectosphae	Plectosphaer	2	0	0	0	0	0	0	1	0	0	0	0	3
Cluster229	Ascomycota	Sord	Ophios	Ophiost	Grosmannia	Grosmannia	0	0	0	0	0	0	1	0	1	1	0	0	3
Cluster270	Ascomycota	Sord	Hypocr	Hypocre	Hypocrea	Hypocrea lac	1	0	0	0	2	0	0	0	0	0	0	0	3
Cluster305	Ascomycota	Doth	n	n	Cenococcum	Cenococcum	0	0	0	0	1	0	0	0	0	1	1	0	3
Cluster421	Ascomycota	Sacch	Saccha	n	Blastobotry	Blastobotrys	0	0	0	0	0	0	0	0	1	0	2	0	3
Cluster904	Ascomycota	Arch	Archae	Archaeo	Archaeorhiz	Archaeorhizo	0	0	2	1	0	0	0	0	0	0	0	0	3
Cluster957	Ascomycota	Doth	n	n	Cenococcum	Cenococcum	1	0	0	0	1	0	0	0	0	1	0	0	3
Cluster25	Basidiomycot	Agar	Agarica	Amanita	Amanita	Amanita brun	1	1	0	0	0	1	0	0	0	0	0	0	3
Cluster32	Basidiomycot	Agar	Russula	Russula	Russula	Russula exalb	1	0	0	0	1	1	0	0	0	0	0	0	3
Cluster49	Basidiomycot	Agar	Russula	Russula	Russula	Russula exalb	0	0	0	0	0	0	0	0	2	1	0	0	3
Cluster53	Basidiomycot	Agar	Agarica	n	Inocybe	Inocybe geop	0	0	0	0	2	0	0	1	0	0	0	0	3
Cluster58	Basidiomycot	Agar	Agarica	Amanita	Amanita	Amanita brun	1	0	0	1	0	0	0	0	0	1	0	0	3
Cluster60	Basidiomycot	Agar	Agarica	Tricholo	Laccaria	Laccaria ame	2	0	0	0	0	0	0	1	0	0	0	0	3
Cluster80	Basidiomycot	Agar	Boletal	Boletace	Xerocomell	Xerocomellus	0	0	1	0	1	0	0	0	0	0	1	0	3
Cluster110	Basidiomycot	Agar	Trechis	'Trechis	Trechispora	Trechispora a	0	1	0	1	0	0	0	0	0	0	0	1	3
Cluster121	Basidiomycot	n	n	n	Marchandio	Marchandior	3	0	0	0	0	0	0	0	0	0	0	0	3
Cluster126	Basidiomycot	Agar	Russula	Stereace	Amylostere	Amylostereu	0	0	0	0	0	0	0	2	1	0	0	0	3
Cluster139	Basidiomycot	Agar	Thelep	Theleph	Thelephora	Thelephora s	0	0	0	0	0	0	0	0	0	1	0	2	3
Cluster163	Basidiomycot	Agar	Agarica	Amanita	Amanita	Amanita brun	0	0	0	0	0	0	0	2	0	0	1	0	3

Cluster192	Basidiomycot	Agar	Agarica	Amanita	Amanita	Amanita bru	0	1	0	0	2	0	0	0	0	0	0	0	3
Cluster319	Basidiomycot	Ento	Entorrh	Entorrhi	Entorrhiza	Entorrhiza as	0	0	0	0	0	0	3	0	0	0	0	0	3
Cluster328	Basidiomycot	Agar	Agarica	Marasm	Chaetocalat	Chaetocalath	0	0	1	0	0	1	0	0	0	0	0	1	3
Cluster356	Basidiomycot	Agar	Russula	Russula	Lactarius	Lactarius dec	1	0	1	0	0	1	0	0	0	0	0	0	3
Cluster375	Basidiomycot	Agar	Boletal	Gyropor	Gyroporus	Gyroporus cy	1	0	0	0	0	0	0	1	1	0	0	0	3
Cluster422	Basidiomycot	Agar	Agarica	Marasm	Moniliophtl	Moniliophthc	0	0	0	0	0	1	0	0	2	0	0	0	3
Cluster442	Basidiomycot	Agar	Agarica	Tricholo	Dendrocolly	Dendrocollyb	0	0	0	0	0	1	0	1	1	0	0	0	3
Cluster452	Basidiomycot	Agar	Agarica	n	Inocybe	Inocybe geop	0	1	0	0	0	0	2	0	0	0	0	0	3
Cluster519	Basidiomycot	Agar	Boletal	Hygroph	Hygrophorc	Hygrophorop	0	2	0	1	0	0	0	0	0	0	0	0	3
Cluster581	Basidiomycot	Agar	Agarica	Amanita	Amanita	Amanita mus	0	1	0	0	0	0	0	0	0	1	1	0	3
Cluster607	Basidiomycot	Agar	Agarica	Tricholo	Camarophy	Camarophyll	0	0	0	0	1	0	1	0	1	0	0	0	3
Cluster664	Basidiomycot	Agar	Agarica	n	Inocybe	Inocybe aff. a	0	0	0	0	0	1	0	0	0	0	1	1	3
Cluster666	Basidiomycot	Agar	Boletal	Sclerod	Scleroderm	Scleroderma	0	0	0	0	1	0	2	0	0	0	0	0	3
Cluster706	Basidiomycot	Agar	Athelia	Atheliac	Athelia	Athelia arach	0	1	1	1	0	0	0	0	0	0	0	0	3
Cluster719	Basidiomycot	Agar	Athelia	Atheliac	Piloderma	Piloderma fa	0	0	1	0	0	0	0	0	0	0	0	2	3
Cluster729	Basidiomycot	Agar	Agarica	n	Inocybe	Inocybe geop	0	0	0	0	0	0	3	0	0	0	0	0	3
Cluster732	Basidiomycot	Agar	Agarica	Bolbitia	Bolbitius	Bolbitius vite	0	0	0	0	0	0	1	1	0	0	1	0	3
Cluster736	Basidiomycot	Agar	Agarica	Tricholo	Dendrocolly	Dendrocollyb	0	0	0	0	0	0	0	1	2	0	0	0	3
Cluster747	Basidiomycot	Agar	Russula	Russula	Russula	Russula exalb	0	0	1	0	0	0	0	0	2	0	0	0	3
Cluster780	Basidiomycot	Agar	Polypo	Auriscal	Lentinellus	Lentinellus u	0	0	0	0	0	0	0	1	0	0	1	1	3
Cluster818	Basidiomycot	Agar	Boletal	Boletac	Xerocomell	Xerocomellus	0	0	0	0	1	0	0	0	0	1	1	0	3
Cluster845	Basidiomycot	Agar	Athelia	Atheliac	Amphinema	Amphinema	0	0	0	1	0	0	0	0	0	0	1	1	3
Cluster931	Basidiomycot	Agar	Boletal	Boletac	Xerocomell	Xerocomellus	0	0	0	0	0	0	0	0	0	3	0	0	3
Cluster1055	Basidiomycot	Agar	Agarica	Tricholo	Pleurocybel	Pleurocybella	0	0	0	0	0	0	0	0	0	0	2	1	3
Cluster324	Blastocladior	Blast	Blastoc	Catenar	Catenomyc	Catenomyces	0	0	1	0	0	0	1	0	0	0	0	1	3
Cluster236	Glomeromyc	Glon	Diversi	Acaulos	Acaulospor	Acaulospora	0	0	0	1	0	0	0	0	0	2	0	0	3
Cluster31	n	n	Mortie	Mortier	Mortierella	Mortierella h	0	1	1	0	0	0	0	0	0	0	1	0	3
Cluster196	n	n	Kickxel	Kickxella	Spiromyces	Spiromyces n	0	2	0	0	0	0	0	0	0	0	0	1	3
Cluster233	n	n	Mortie	Mortier	Mortierella	Mortierella p	0	1	0	0	1	0	0	0	0	1	0	0	3

Cluster261	n	n	Mucora	n	Umbelopsis	Umbelopsis r	0	0	1	0	1	1	0	0	0	0	0	0	3
Cluster383	n	n	Endogo	Endogo	Endogone	Endogone pis	0	1	2	0	0	0	0	0	0	0	0	0	3
Cluster403	n	n	n	Nuclear	Nuclearia	Nuclearia the	0	0	1	0	1	0	0	0	1	0	0	0	3
Cluster465	n	n	Mucora	n	Umbelopsis	Umbelopsis r	0	1	0	0	0	0	0	1	0	0	0	1	3
Cluster849	n	n	Mortie	Mortier	Mortierella	Mortierella s	0	0	0	2	0	1	0	0	0	0	0	0	3
Cluster919	n	n	Mortie	Mortier	Mortierella	Mortierella p	0	0	0	1	0	0	0	0	1	0	1	0	3
Cluster990	n	Spirc	Sporad	Oxytrich	n	Oxytrichidae	0	0	0	0	1	0	0	1	0	0	1	0	3
Cluster1049	n	n	Mortie	Mortier	Dissophora	Dissophora d	1	0	0	0	1	0	0	0	0	1	0	0	3
Cluster232	Platyhelmint	Turb	Lecitho	Prorhyn	Geocentrop	Geocentroph	0	1	1	1	0	0	0	0	0	0	0	0	3
Cluster4	Ascomycota	Doth	n	n	Cenococcur	Cenococcum	1	0	0	0	0	0	1	0	0	0	0	0	2
Cluster33	Ascomycota	Leoti	n	Myxotri	Byssoascus	Byssoascus st	0	0	0	0	0	0	0	0	1	1	0	0	2
Cluster98	Ascomycota	Saccl	Saccha	n	Candida	Candida sp. E	0	0	0	2	0	0	0	0	0	0	0	0	2
Cluster111	Ascomycota	Leoti	n	n	Xylogone	Xylogone gar	0	0	0	0	2	0	0	0	0	0	0	0	2
Cluster159	Ascomycota	n	n	n	Eladia	Eladia saccul	0	0	0	0	1	0	0	0	0	1	0	0	2
Cluster228	Ascomycota	Leoti	n	Myxotri	Byssoascus	Byssoascus st	0	0	0	2	0	0	0	0	0	0	0	0	2
Cluster276	Ascomycota	Doth	Botryo	Botryos	Lasiodiplod	Lasiodiplodia	0	0	2	0	0	0	0	0	0	0	0	0	2
Cluster279	Ascomycota	Doth	Pleosp	Melano	Herpotrichi	Herpotrichia	0	1	0	0	0	0	0	0	0	0	1	0	2
Cluster284	Ascomycota	Leoti	Helotia	Hyalosc	Lachnum	Lachnum scle	0	0	0	0	0	0	2	0	0	0	0	0	2
Cluster371	Ascomycota	Doth	n	n	Cenococcur	Cenococcum	0	0	0	0	0	0	0	0	1	1	0	0	2
Cluster397	Ascomycota	Sord	Hypocr	Clavicipi	Metarhiziur	Metarhizium	0	0	0	0	0	0	0	0	0	0	2	0	2
Cluster439	Ascomycota	Leoti	n	Myxotri	Pseudogym	Pseudogymn	0	1	0	0	0	0	0	0	0	0	1	0	2
Cluster453	Ascomycota	Doth	Dothid	Dothior	Aureobasid	Aureobasidiu	0	1	0	0	0	0	0	0	0	1	0	0	2
Cluster458	Ascomycota	Leoti	n	Myxotri	Byssoascus	Byssoascus st	0	0	0	0	0	0	0	0	0	0	1	1	2
Cluster544	Ascomycota	Peziz	Pezizal	Discinac	Gyromitra	Gyromitra es	0	0	0	0	0	0	0	0	2	0	0	0	2
Cluster588	Ascomycota	Leoti	n	Erysipha	Cystotheca	Cystotheca w	0	0	0	0	0	0	0	0	2	0	0	0	2
Cluster640	Ascomycota	Sord	Ophios	Ophiost	Grosmannia	Grosmannia	0	0	0	0	1	0	0	0	0	0	1	0	2
Cluster669	Ascomycota	n	n	n	n	ascomycete s	0	0	0	0	0	0	0	0	0	0	1	1	2
Cluster672	Ascomycota	Euro	Eurotia	Trichocc	Penicillium	Penicillium sp	0	1	0	0	0	0	0	0	0	1	0	0	2
Cluster724	Ascomycota	Leoti	Helotia	Leotiace	Neobulgaria	Neobulgaria	0	0	0	1	0	0	0	0	1	0	0	0	2

Cluster748	Ascomycota	Doth	Botryo:	Botryos|	Lasiodiplod	Lasiodiplodia	0	0	0	0	0	0	0	0	0	0	0	2	2
Cluster825	Ascomycota	Leoti	n	Myxotri	Pseudogym	Pseudogymn	0	0	0	0	0	0	0	0	1	0	0	1	2
Cluster892	Ascomycota	Sord	Hypocr	Hypocre	Hypocrea	Hypocrea kor	1	0	0	0	0	0	0	1	0	0	0	0	2
Cluster17	Basidiomycot	Agar	Boletal	Sclerode	Scleroderm	Scleroderma	2	0	0	0	0	0	0	0	0	0	0	0	2
Cluster23	Basidiomycot	Agar	Agarica	Tricholo	Dendrocolly	Dendrocollyb	0	0	0	0	0	0	0	0	2	0	0	0	2
Cluster27	Basidiomycot	Agar	Trechis	'Trechis|	Trechispora	Trechispora a	2	0	0	0	0	0	0	0	0	0	0	0	2
Cluster62	Basidiomycot	Agar	Agarica	n	Inocybe	Inocybe aff. a	0	0	0	0	0	0	1	0	0	0	1	0	2
Cluster69	Basidiomycot	Agar	Thelep	Theleph	Thelephora	Thelephora s	0	0	0	1	0	0	0	0	0	1	0	0	2
Cluster83	Basidiomycot	Agar	Agarica	Tricholo	Dendrocolly	Dendrocollyb	0	0	0	0	1	0	0	1	0	0	0	0	2
Cluster91	Basidiomycot	Agar	Agarica	n	Inocybe	Inocybe geop	0	0	0	0	0	0	1	1	0	0	0	0	2
Cluster123	Basidiomycot	Agar	Agarica	Tricholo	Dendrocolly	Dendrocollyb	0	0	0	0	0	0	0	0	2	0	0	0	2
Cluster160	Basidiomycot	Agar	Russula	Stereace	Amylostere	Amylostereu	0	0	0	0	0	0	0	0	2	0	0	0	2
Cluster240	Basidiomycot	Agar	Agarica	n	Inocybe	Inocybe geop	0	1	0	1	0	0	0	0	0	0	0	0	2
Cluster253	Basidiomycot	Agar	Agarica	Tricholo	Laccaria	Laccaria ame	0	0	0	0	0	0	0	1	1	0	0	0	2
Cluster258	Basidiomycot	Agar	Russula	Russula	Russula	Russula exalb	0	0	0	0	1	1	0	0	0	0	0	0	2
Cluster286	Basidiomycot	n	Leucos	Leucosp	Leucosporic	Leucosporidi	0	0	0	0	0	0	1	1	0	0	0	0	2
Cluster306	Basidiomycot	Agar	Agarica	n	Inocybe	Inocybe aff. a	0	0	0	0	0	0	1	1	0	0	0	0	2
Cluster310	Basidiomycot	Agar	Boletal	Sclerode	Scleroderm	Scleroderma	0	0	0	0	0	2	0	0	0	0	0	0	2
Cluster311	Basidiomycot	Agar	Agarica	n	Inocybe	Inocybe aff. a	0	0	0	1	1	0	0	0	0	0	0	0	2
Cluster341	Basidiomycot	Agar	Agarica	Amanita	Amanita	Amanita wes	0	0	0	1	0	0	0	0	0	0	0	1	2
Cluster348	Basidiomycot	Agar	Agarica	Amanita	Amanita	Amanita brui	0	0	0	1	0	0	0	0	0	1	0	0	2
Cluster389	Basidiomycot	Agar	Agarica	Amanita	Amanita	Amanita brui	0	0	0	0	0	0	0	0	0	1	0	1	2
Cluster417	Basidiomycot	Agar	Boletal	Boletace	Boletus	Boletus eduli	0	1	0	0	0	0	0	0	0	0	0	1	2
Cluster437	Basidiomycot	Agar	Agarica	n	Inocybe	Inocybe aff. a	0	1	0	0	0	0	0	0	0	0	0	1	2
Cluster447	Basidiomycot	Agar	Russula	Russula	Russula	Russula exalb	0	0	0	0	0	2	0	0	0	0	0	0	2
Cluster457	Basidiomycot	Agar	Agarica	Amanita	Amanita	Amanita brui	0	0	0	1	1	0	0	0	0	0	0	0	2
Cluster468	Basidiomycot	Agar	Agarica	Tricholo	Dendrocolly	Dendrocollyb	0	0	0	0	0	0	0	0	2	0	0	0	2
Cluster470	Basidiomycot	Agar	Agarica	Tricholo	Panellus	Panellus styp	0	0	1	0	0	0	0	0	0	0	0	1	2
Cluster471	Basidiomycot	Agar	Sebacin	Sebacin	Sebacina	Sebacina ver	0	1	1	0	0	0	0	0	0	0	0	0	2

Cluster495	Basidiomycot	Agar	Russula	Russula	Russula	Russula exalb	1	0	0	0	0	0	0	1	0	0	0	0	2
Cluster549	Basidiomycot	Agar	Agarica	n	Inocybe	Inocybe geop	0	0	0	0	0	0	2	0	0	0	0	0	2
Cluster573	Basidiomycot	Agar	Agarica	Bolbitia	Bolbitius	Bolbitius vite	0	0	0	0	0	0	1	0	0	0	1	0	2
Cluster576	Basidiomycot	Agar	Russula	Russula	Russula	Russula exalb	0	0	0	0	0	0	0	1	1	0	0	0	2
Cluster596	Basidiomycot	Agar	Agarica	Tricholo	Laccaria	Laccaria ame	0	0	1	0	1	0	0	0	0	0	0	0	2
Cluster611	Basidiomycot	Agar	Athelia	Atheliac	Athelia	Athelia arach	0	0	0	1	0	0	0	0	0	0	1	0	2
Cluster654	Basidiomycot	Agar	Boletal	Tapinell	Pseudomer	Pseudomeru	0	0	1	0	0	0	0	0	0	0	0	1	2
Cluster660	Basidiomycot	Agar	Agarica	Amanita	Amanita	Amanita brun	1	0	0	0	0	0	0	0	1	0	0	0	2
Cluster689	Basidiomycot	Agar	Boletal	Boletace	Xerocomell	Xerocomellus	0	0	2	0	0	0	0	0	0	0	0	0	2
Cluster726	Basidiomycot	Agar	Agarica	Tricholo	Sarcomyxa	Sarcomyxa se	0	0	0	1	0	0	0	0	1	0	0	0	2
Cluster731	Basidiomycot	Agar	Boletal	n	Leucogyrop	Leucogyroph	0	0	1	0	0	0	0	0	0	0	0	1	2
Cluster767	Basidiomycot	Agar	Boletal	Boletace	Xerocomell	Xerocomellus	0	0	0	0	1	0	0	0	1	0	0	0	2
Cluster770	Basidiomycot	Agar	Athelia	Atheliac	Athelia	Athelia arach	0	0	0	0	0	0	0	0	0	0	2	0	2
Cluster788	Basidiomycot	Agar	Agarica	Amanita	Amanita	Amanita brun	0	0	0	2	0	0	0	0	0	0	0	0	2
Cluster808	Basidiomycot	Agar	Agarica	Amanita	Amanita	Amanita brun	0	0	1	0	1	0	0	0	0	0	0	0	2
Cluster843	Basidiomycot	Trem	Tremel	Tremella	Asterotrem	Asterotremel	0	0	0	1	0	0	0	0	0	0	1	0	2
Cluster872	Basidiomycot	Agar	Boletal	Boletace	Xerocomell	Xerocomellus	0	0	0	0	0	0	0	2	0	0	0	0	2
Cluster883	Basidiomycot	Agar	Russula	Russula	Russula	Russula exalb	0	0	0	0	0	0	0	1	0	0	1	0	2
Cluster887	Basidiomycot	Trem	Filobas	Filobasi	Filobasidiur	Filobasidium	0	0	2	0	0	0	0	0	0	0	0	0	2
Cluster895	Basidiomycot	Agar	Boletal	n	Leucogyrop	Leucogyroph	0	0	1	0	0	0	0	0	0	1	0	0	2
Cluster955	Basidiomycot	n	Leucos	Leucosp	Leucosporic	Leucosporidi	0	0	0	0	0	0	0	0	0	0	0	2	2
Cluster1053	Basidiomycot	Agar	Agarica	n	Inocybe	Inocybe aff. a	0	0	0	0	0	0	1	0	0	0	1	0	2
Cluster82	Chytridiomyc	Chyt	Chytrid	Chytridi	Chytriomyc	Chytriomyces	2	0	0	0	0	0	0	0	0	0	0	0	2
Cluster429	Chytridiomyc	Chyt	Chytrid	n	Entophlycti	Entophlyctis	0	1	0	0	0	0	0	0	1	0	0	0	2
Cluster534	Chytridiomyc	Chyt	Chytrid	n	n	Chytridiales s	0	0	2	0	0	0	0	0	0	0	0	0	2
Cluster175	Glomeromyc	Arch	Archae	Archaec	Archaeospc	Archaeospor	0	0	0	0	1	0	0	0	0	0	1	0	2
Cluster3	n	n	Mortie	Mortier	Dissophora	Dissophora d	0	1	0	0	0	0	0	0	0	0	0	1	2
Cluster180	n	n	Mucora	n	Umbelopsis	Umbelopsis r	0	0	0	1	0	0	1	0	0	0	0	0	2
Cluster271	n	n	Mucora	n	Umbelopsis	Umbelopsis r	0	0	0	1	0	1	0	0	0	0	0	0	2

Cluster273	n	n	Mortie	Mortier	Mortierella	Mortierella s	0	0	0	0	0	0	0	0	0	1	0	1	2
Cluster308	n	n	Endogo	Endogo	Endogone	Endogone pi	0	0	1	0	0	0	1	0	0	0	0	0	2
Cluster317	n	n	Entomo	Basidiob	Basidiobolu	Basidiobolus	1	0	0	0	1	0	0	0	0	0	0	0	2
Cluster332	n	Spiro	Urostyl	Pseudou	Pseudouros	Pseudourosty	0	0	0	0	0	0	0	0	0	1	1	0	2
Cluster334	n	n	Cercon	Heteron	Heteromita	Heteromita g	0	0	1	0	0	0	0	0	0	0	1	0	2
Cluster394	n	n	Endogo	Endogo	Endogone	Endogone lac	0	0	0	0	0	0	0	0	1	0	1	0	2
Cluster426	n	n	n	n	n	Cercozoa sp.	0	0	0	0	0	2	0	0	0	0	0	0	2
Cluster432	n	n	Mortie	Mortier	Dissophora	Dissophora d	0	0	0	1	0	0	0	0	1	0	0	0	2
Cluster469	n	Spiro	Sporad	Oxytrich	Protogastro	Protogastros	0	0	1	1	0	0	0	0	0	0	0	0	2
Cluster473	n	n	Mucora	n	Umbelopsis	Umbelopsis r	0	0	1	0	0	0	1	0	0	0	0	0	2
Cluster477	n	n	n	Nuclear	Nuclearia	Nuclearia the	0	0	1	0	0	1	0	0	0	0	0	0	2
Cluster631	n	n	Mortie	Mortier	Dissophora	Dissophora d	0	0	0	0	0	0	0	0	0	0	0	2	2
Cluster659	n	n	n	Vampyr	Arachnula	Arachnula im	0	0	0	1	0	0	0	1	0	0	0	0	2
Cluster661	n	n	Cercon	n	Spongomor	Spongomona	0	0	0	0	0	0	0	1	0	0	1	0	2
Cluster687	n	n	Euglyp	Euglyph	Assulina	Assulina mus	0	0	1	0	0	0	0	0	1	0	0	0	2
Cluster745	n	n	Mortie	Mortier	Mortierella	Mortierella p	0	0	1	0	1	0	0	0	0	0	0	0	2
Cluster796	n	n	Cercon	Cercom	Paracercom	Paracercomo	0	0	2	0	0	0	0	0	0	0	0	0	2
Cluster797	n	n	Euglyp	Euglyph	Assulina	Assulina mus	0	1	1	0	0	0	0	0	0	0	0	0	2
Cluster804	n	n	Mucora	n	Umbelopsis	Umbelopsis r	0	0	0	0	0	0	0	0	1	0	1	0	2
Cluster835	n	n	Choano	Codono	Monosiga	Monosiga ov	0	0	0	0	0	0	0	0	0	0	2	0	2
Cluster852	n	n	Mortie	Mortier	Mortierella	Mortierella h	0	0	0	1	0	0	0	0	0	0	0	1	2
Cluster866	n	n	Mortie	Mortier	Mortierella	Mortierella p	0	0	1	0	0	0	0	0	0	0	1	0	2
Cluster973	n	n	Endogo	Endogo	Endogone	Endogone pi	0	1	0	0	0	0	0	0	0	0	0	1	2
Cluster415	Platyhelmint	Turb	Lecitho	Prorhyn	Geocentrop	Geocentroph	0	0	0	2	0	0	0	0	0	0	0	0	2
Cluster517	Platyhelmint	Turb	Rhabdo	Typhlop	Phaenocora	Phaenocora u	0	0	1	0	1	0	0	0	0	0	0	0	2
Cluster249	Annelida	n	Haplota	Megasco	Achaeta	Achaeta cam	0	0	1	0	0	0	0	0	0	0	0	0	1
Cluster364	Annelida	Polyo	Phylloc	Syllidae	Typosyllis	Typosyllis bel	0	0	1	0	0	0	0	0	0	0	0	0	1
Cluster406	Annelida	Polyo	Phylloc	Syllidae	Typosyllis	Typosyllis bel	0	0	0	0	0	0	0	0	0	0	0	1	1
Cluster834	Annelida	n	n	n	Hrabeiella	Hrabeiella pe	0	0	0	0	0	0	0	1	0	0	0	0	1

Cluster996	Annelida	Poly	Phylloc	Syllidae	Typosyllis	Typosyllis bel	0	0	1	0	0	0	0	0	0	0	0	0	1
Cluster189	Apicomplexa	Cocc	Eucocc	Eimeriid	n	Eimeriidae sp	0	0	0	0	0	0	0	0	1	0	0	0	1
Cluster998	Apicomplexa	Cocc	Eucocc	Eimeriid	n	Eimeriidae sp	0	0	0	0	0	0	0	0	0	0	0	1	1
Cluster1041	Apicomplexa	Cocc	Eucocc	Eimeriid	n	Eimeriidae sp	0	0	0	0	0	0	0	0	0	0	0	1	1
Cluster218	Arthropoda	Ellip	Collem	Tomoce	Tomocerus	Tomocerus vi	0	0	1	0	0	0	0	0	0	0	0	0	1
Cluster536	Arthropoda	Ellip	Collem	Neanuri	Neanura	Neanura latic	0	0	0	1	0	0	0	0	0	0	0	0	1
Cluster614	Arthropoda	Ellip	Collem	Tullberg	Tullbergia	Tullbergia yo	0	0	1	0	0	0	0	0	0	0	0	0	1
Cluster907	Arthropoda	Arac	Oribati	Brachyc	n	Brachychthon	0	0	0	0	1	0	0	0	0	0	0	0	1
Cluster938	Arthropoda	Ellip	Collem	Isotomi	Folsomia	Folsomia oct	0	0	0	0	0	0	1	0	0	0	0	0	1
Cluster965	Arthropoda	Ellip	Collem	Onychiu	Onychiurus	Onychiurus y	0	0	0	0	0	0	1	0	0	0	0	0	1
Cluster14	Ascomycota	Peziz	Pezizal	Discinac	Gyromitra	Gyromitra es	1	0	0	0	0	0	0	0	0	0	0	0	1
Cluster15	Ascomycota	Leoti	n	Myxotri	Byssoascus	Byssoascus s	0	0	0	0	0	0	0	0	0	1	0	0	1
Cluster35	Ascomycota	Leoti	n	Myxotri	Byssoascus	Byssoascus s	0	0	0	0	1	0	0	0	0	0	0	0	1
Cluster41	Ascomycota	Leoti	Helotia	Leotiace	Leotia	Leotia lubrica	0	0	0	1	0	0	0	0	0	0	0	0	1
Cluster79	Ascomycota	Sacc	Saccha	n	Candida	Candida sp. E	0	0	0	1	0	0	0	0	0	0	0	0	1
Cluster93	Ascomycota	Euro	Pyrenu	Pyrenul	Anthracoth	Anthracothe	0	0	0	0	0	0	0	0	1	0	0	0	1
Cluster124	Ascomycota	Leoti	Helotia	Scleroti	Botryotinia	Botryotinia fu	1	0	0	0	0	0	0	0	0	0	0	0	1
Cluster149	Ascomycota	Leoti	n	Myxotri	Byssoascus	Byssoascus s	0	0	1	0	0	0	0	0	0	0	0	0	1
Cluster193	Ascomycota	Sord	Chaeto	Chaetos	Chaetospha	Chaetosphae	0	0	1	0	0	0	0	0	0	0	0	0	1
Cluster199	Ascomycota	Peziz	Pezizal	Discinac	Gyromitra	Gyromitra es	0	0	0	0	0	0	0	0	1	0	0	0	1
Cluster213	Ascomycota	Sord	Ophios	Ophiost	Grosmanni	Grosmannia	0	1	0	0	0	0	0	0	0	0	0	0	1
Cluster220	Ascomycota	Doth	Dothid	Dothior	Aureobasid	Aureobasidiu	0	0	0	0	0	1	0	0	0	0	0	0	1
Cluster230	Ascomycota	Sord	Magna	Magnap	Phialophor	Phialophora	0	0	1	0	0	0	0	0	0	0	0	0	1
Cluster250	Ascomycota	Leoti	Helotia	Leotiace	Neobulgari	Neobulgaria	0	0	0	1	0	0	0	0	0	0	0	0	1
Cluster252	Ascomycota	Peziz	Pezizal	Tuberac	Labyrinthor	Labyrinthom	0	0	0	0	0	0	0	1	0	0	0	0	1
Cluster254	Ascomycota	Leoti	n	Myxotri	Pseudogym	Pseudogymn	0	0	0	0	0	0	0	0	1	0	0	0	1
Cluster290	Ascomycota	Leoti	n	Myxotri	Pseudogym	Pseudogymn	0	0	0	0	0	0	0	0	0	0	0	1	1
Cluster336	Ascomycota	Sacc	Saccha	n	Candida	Candida sp. E	0	0	0	0	0	1	0	0	0	0	0	0	1
Cluster350	Ascomycota	Labo	Pyxidic	Pyxidiop	Pyxidiophor	Pyxidiophora	0	0	0	0	0	1	0	0	0	0	0	0	1

Cluster369	Ascomycota	Doth	Capnoc	Mycosp	Mycosphae	Mycosphaere	0	0	0	0	0	0	1	0	0	0	0	0	1
Cluster374	Ascomycota	Doth	n	n	Cenococcur	Cenococcum	0	0	0	0	0	0	0	0	0	1	0	0	1
Cluster459	Ascomycota	Peziz	Pezizal	Discinac	Gyromitra	Gyromitra es	0	0	0	0	0	0	0	0	1	0	0	0	1
Cluster530	Ascomycota	Peziz	Pezizal	Tuberac	Reddellomy	Reddellomyc	0	0	0	1	0	0	0	0	0	0	0	0	1
Cluster531	Ascomycota	Peziz	Pezizal	Tuberac	Labyrinthor	Labyrinthom	0	0	1	0	0	0	0	0	0	0	0	0	1
Cluster548	Ascomycota	Sord	Ophios	Ophiost	Grosmannia	Grosmannia	0	0	0	0	0	0	0	0	0	0	1	0	1
Cluster564	Ascomycota	Peziz	Pezizal	Pyroner	Leucoscyph	Leucoscypha	0	0	0	0	0	0	0	0	0	0	0	1	1
Cluster575	Ascomycota	Leoti	n	Myxotri	Byssoascus	Byssoascus st	0	0	0	0	0	0	0	0	0	0	0	1	1
Cluster580	Ascomycota	Doth	n	n	Cenococcur	Cenococcum	0	0	1	0	0	0	0	0	0	0	0	0	1
Cluster589	Ascomycota	Euro	Eurotia	Trichocc	Paecilomyc	Paecilomyces	0	0	0	0	1	0	0	0	0	0	0	0	1
Cluster601	Ascomycota	Sord	Ophios	Ophiost	Grosmannia	Grosmannia	0	0	0	0	0	0	0	0	0	0	1	0	1
Cluster616	Ascomycota	Geog	Geoglo	Geoglos	Trichogloss	Trichoglossur	0	0	0	0	0	0	0	0	0	1	0	0	1
Cluster617	Ascomycota	Doth	n	n	Cenococcur	Cenococcum	0	0	0	0	0	0	0	0	0	1	0	0	1
Cluster629	Ascomycota	Saccl	Saccha	Dipodas	Geotrichum	Geotrichum l	0	0	0	0	0	1	0	0	0	0	0	0	1
Cluster630	Ascomycota	Sord	Hypocr	Hypocre	Hypocrea	Hypocrea lac	0	0	0	0	1	0	0	0	0	0	0	0	1
Cluster639	Ascomycota	n	n	n	n	Ascomycota s	0	0	1	0	0	0	0	0	0	0	0	0	1
Cluster642	Ascomycota	Sord	Hypocr	Hypocre	Hypomyces	Hypomyces c	0	0	0	0	0	0	0	0	0	1	0	0	1
Cluster678	Ascomycota	Leoti	n	Myxotri	Byssoascus	Byssoascus st	0	0	0	0	1	0	0	0	0	0	0	0	1
Cluster680	Ascomycota	Sord	Xylaria	Xylariac	Ascotricha	Ascotricha lu	0	0	0	0	0	0	0	0	0	0	0	1	1
Cluster691	Ascomycota	Sord	Magna	Magnap	Phialophora	Phialophora s	0	0	0	0	0	0	0	0	0	0	1	0	1
Cluster710	Ascomycota	Pneu	Pneum	Pneumo	Pneumocys	Pneumocysti	0	0	0	0	0	1	0	0	0	0	0	0	1
Cluster720	Ascomycota	Doth	Dothid	Dothide	Dothidea	Dothidea hip	0	0	0	0	0	0	0	0	0	0	1	0	1
Cluster765	Ascomycota	Leoti	n	Myxotri	Pseudogym	Pseudogymn	0	0	0	0	0	0	0	0	1	0	0	0	1
Cluster779	Ascomycota	Arch	Archae	Archaec	Archaeorhiz	Archaeorhizo	0	1	0	0	0	0	0	0	0	0	0	0	1
Cluster789	Ascomycota	Arch	Archae	Archaec	Archaeorhiz	Archaeorhizo	0	0	0	1	0	0	0	0	0	0	0	0	1
Cluster792	Ascomycota	Euro	Eurotia	Trichocc	Penicillium	Penicillium fr	0	0	1	0	0	0	0	0	0	0	0	0	1
Cluster805	Ascomycota	Leoti	n	Ascodicl	Pseudophac	Pseudophaci	0	0	0	0	0	0	1	0	0	0	0	0	1
Cluster817	Ascomycota	Sord	Hypocr	Ophiocc	Ophiocordy	Ophiocordyc	0	0	0	0	0	0	0	0	0	0	1	0	1
Cluster821	Ascomycota	Peziz	Pezizal	Discinac	Gyromitra	Gyromitra es	0	0	0	0	0	0	1	0	0	0	0	0	1

Cluster838	Ascomycota	n	n	n	Tetracladiu	Tetracladium	0	1	0	0	0	0	0	0	0	0	0	0	1
Cluster848	Ascomycota	Sacc	Saccha	n	Candida	Candida sp. E	0	0	0	0	0	0	0	0	0	0	0	1	1
Cluster870	Ascomycota	Euro	Eurotia	Trichoc	Penicillium	Penicillium s	0	0	1	0	0	0	0	0	0	0	0	0	1
Cluster896	Ascomycota	Leoti	n	Myxotri	Byssoascus	Byssoascus s	0	0	0	0	1	0	0	0	0	0	0	0	1
Cluster902	Ascomycota	Sord	Conioc	Conioch	Coniochaet	Coniochaeta	0	0	0	0	1	0	0	0	0	0	0	0	1
Cluster909	Ascomycota	Sacc	Saccha	n	Candida	Candida oreg	0	0	1	0	0	0	0	0	0	0	0	0	1
Cluster929	Ascomycota	Sord	Sordari	n	Kionochaet	Kionochaeta	0	0	0	0	0	0	1	0	0	0	0	0	1
Cluster971	Ascomycota	Sacc	Saccha	Lipomy	Lipomyces	Lipomyces kc	0	0	0	1	0	0	0	0	0	0	0	0	1
Cluster975	Ascomycota	Doth	Capno	Mycosp	Passalora	Passalora zar	0	0	0	0	0	0	0	0	0	0	1	0	1
Cluster997	Ascomycota	Doth	n	n	Cenococcu	Cenococcum	0	0	0	0	0	0	0	0	1	0	0	0	1
Cluster1038	Ascomycota	n	n	n	n	ascomycete s	0	0	0	0	0	0	0	0	1	0	0	0	1
Cluster1046	Ascomycota	Doth	Pleosp	Melano	Herpotrichi	Herpotrichia	0	0	0	0	0	0	0	0	1	0	0	0	1
Cluster1073	Ascomycota	Pezi	Pezizal	Discinac	Gyromitra	Gyromitra es	0	0	0	0	0	0	0	1	0	0	0	0	1
Cluster1079	Ascomycota	Sacc	Saccha	n	Candida	Candida sp. E	0	0	0	0	0	0	0	0	0	1	0	0	1
Cluster6	Basidiomyco	Agar	Boletal	Boletac	Boletus	Boletus satar	0	0	1	0	0	0	0	0	0	0	0	0	1
Cluster7	Basidiomyco	Agar	Agarica	n	Inocybe	Inocybe aff. a	0	1	0	0	0	0	0	0	0	0	0	0	1
Cluster8	Basidiomyco	Agar	Agarica	Amanita	Amanita	Amanita aff.	0	0	0	0	0	0	0	0	0	0	0	1	1
Cluster21	Basidiomyco	Agar	Agarica	Tricholo	Laccaria	Laccaria ame	0	0	0	0	0	0	0	0	1	0	0	0	1
Cluster34	Basidiomyco	Agar	Boletal	Sclerod	Scleroderm	Scleroderma	1	0	0	0	0	0	0	0	0	0	0	0	1
Cluster37	Basidiomyco	Agar	Boletal	Sclerod	Scleroderm	Scleroderma	0	0	0	0	0	0	0	1	0	0	0	0	1
Cluster42	Basidiomyco	Agar	Agarica	n	Inocybe	Inocybe aff. a	0	0	1	0	0	0	0	0	0	0	0	0	1
Cluster43	Basidiomyco	Agar	Agarica	n	Inocybe	Inocybe aff. a	0	0	0	0	0	1	0	0	0	0	0	0	1
Cluster50	Basidiomyco	Agar	Boletal	Boletac	Xerocomell	Xerocomellu	0	0	0	0	0	0	0	1	0	0	0	0	1
Cluster51	Basidiomyco	Agar	Agarica	n	Inocybe	Inocybe geop	0	0	0	0	0	0	1	0	0	0	0	0	1
Cluster61	Basidiomyco	Agar	Boletal	Boletac	Boletus	Boletus eduli	0	1	0	0	0	0	0	0	0	0	0	0	1
Cluster86	Basidiomyco	Agar	Agarica	Amanita	Amanita	Amanita brur	1	0	0	0	0	0	0	0	0	0	0	0	1
Cluster92	Basidiomyco	Agar	Agarica	n	Inocybe	Inocybe geop	0	0	0	0	0	0	1	0	0	0	0	0	1
Cluster105	Basidiomyco	Agar	Athelia	Atheliac	Piloderma	Piloderma fa	0	0	0	0	0	0	0	0	0	0	1	0	1
Cluster112	Basidiomyco	Agar	Hymen	Hymeno	Fomitiporia	Fomitiporia r	0	0	0	0	1	0	0	0	0	0	0	0	1

Cluster115	Basidiomycot	Agar	Agarica	Marasm	Crinipellis	Crinipellis sp.	1	0	0	0	0	0	0	0	0	0	0	0	1
Cluster147	Basidiomycot	Agar	Boletal	Sclerode	Scleroderm	Scleroderma	1	0	0	0	0	0	0	0	0	0	0	0	1
Cluster152	Basidiomycot	Agar	Boletal	Boletace	Xerocomell	Xerocomellus	0	0	0	0	0	1	0	0	0	0	0	0	1
Cluster156	Basidiomycot	Agar	Agarica	n	Inocybe	Inocybe geop	0	0	0	0	0	0	1	0	0	0	0	0	1
Cluster157	Basidiomycot	Agar	Agarica	Amanita	Amanita	Amanita aff.	0	0	0	0	0	0	1	0	0	0	0	0	1
Cluster176	Basidiomycot	Agar	Agarica	n	Inocybe	Inocybe aff. a	0	0	0	0	0	0	1	0	0	0	0	0	1
Cluster219	Basidiomycot	Agar	Agarica	Tricholo	Dendrocolly	Dendrocollyb	0	0	0	0	0	0	0	0	1	0	0	0	1
Cluster224	Basidiomycot	Agar	Agarica	n	Inocybe	Inocybe geop	0	0	0	0	0	0	1	0	0	0	0	0	1
Cluster226	Basidiomycot	Agar	Agarica	Tricholo	Camarophy	Camarophyllu	0	0	0	0	0	0	0	0	0	0	1	0	1
Cluster242	Basidiomycot	Agar	Agarica	Amanita	Amanita	Amanita brur	0	0	0	0	0	0	0	1	0	0	0	0	1
Cluster247	Basidiomycot	Trem	Tremel	Tremella	Asterotrem	Asterotremel	0	0	0	1	0	0	0	0	0	0	0	0	1
Cluster255	Basidiomycot	Agar	Agarica	Tricholo	Laccaria	Laccaria ame	1	0	0	0	0	0	0	0	0	0	0	0	1
Cluster257	Basidiomycot	Agar	Phallal	Phallace	Phallus	Phallus hadri	0	0	0	0	0	0	0	0	1	0	0	0	1
Cluster259	Basidiomycot	Agar	Gomph	Gompha	Ramaria	Ramaria stric	0	0	1	0	0	0	0	0	0	0	0	0	1
Cluster316	Basidiomycot	Agar	Agarica	n	Inocybe	Inocybe aff. a	0	0	0	0	0	0	0	0	0	0	1	0	1
Cluster318	Basidiomycot	Agar	Agarica	n	Inocybe	Inocybe aff. a	0	0	0	0	0	1	0	0	0	0	0	0	1
Cluster333	Basidiomycot	Agar	Agarica	n	Inocybe	Inocybe aff. a	0	0	0	0	0	0	0	0	0	0	0	1	1
Cluster340	Basidiomycot	Agar	Agarica	Amanita	Amanita	Amanita brur	0	0	0	0	0	0	0	0	1	0	0	0	1
Cluster342	Basidiomycot	Agar	Agarica	n	Inocybe	Inocybe geop	0	0	0	0	1	0	0	0	0	0	0	0	1
Cluster346	Basidiomycot	Trem	Cystofi	Cystofilc	Mrakia	Mrakia psych	0	1	0	0	0	0	0	0	0	0	0	0	1
Cluster355	Basidiomycot	Agar	Thelep	Theleph	Thelephora	Thelephora s	0	0	0	1	0	0	0	0	0	0	0	0	1
Cluster357	Basidiomycot	Agar	Agarica	n	Inocybe	Inocybe aff. a	0	0	0	0	0	0	0	0	0	0	0	1	1
Cluster365	Basidiomycot	Agar	Agarica	Amanita	Amanita	Amanita brur	0	0	0	0	1	0	0	0	0	0	0	0	1
Cluster368	Basidiomycot	Agar	Athelia	Atheliac	Athelia	Athelia arach	0	0	0	1	0	0	0	0	0	0	0	0	1
Cluster372	Basidiomycot	Agar	Agarica	Tricholo	Laccaria	Laccaria ame	0	0	0	0	0	0	0	0	1	0	0	0	1
Cluster376	Basidiomycot	Trem	Cystofi	Cystofilc	Cystofilobas	Cystofilobasi	0	0	0	0	0	0	0	0	0	0	0	1	1
Cluster385	Basidiomycot	Agar	Agarica	n	Inocybe	Inocybe aff. a	0	0	0	0	0	0	0	0	0	0	0	1	1
Cluster392	Basidiomycot	Agar	Russula	Russulac	Russula	Russula exalb	0	0	0	0	0	0	0	1	0	0	0	0	1
Cluster407	Basidiomycot	Agar	Agarica	Amanita	Amanita	Amanita aff.	0	0	0	1	0	0	0	0	0	0	0	0	1

Cluster411	Basidiomycot	Agar	Agarica	n	Inocybe	Inocybe aff. a	0	0	0	0	0	0	0	0	0	0	0	1	1
Cluster414	Basidiomycot	Agar	Agarica	n	Inocybe	Inocybe aff. a	0	0	0	0	0	0	0	0	1	0	0	0	1
Cluster423	Basidiomycot	Agar	Athelia	Atheliac	Piloderma	Piloderma fa	0	0	0	0	0	0	0	0	0	1	0	0	1
Cluster436	Basidiomycot	Trem	Cystofi	n	Guehomyce	Guehomyces	0	0	0	0	0	0	0	1	0	0	0	0	1
Cluster443	Basidiomycot	Agar	Agarica	Tricholo	Dendrocolly	Dendrocollyb	0	0	0	0	0	0	0	1	0	0	0	0	1
Cluster449	Basidiomycot	Agar	Agarica	n	Inocybe	Inocybe aff. a	0	0	0	0	0	0	0	0	0	0	0	1	1
Cluster460	Basidiomycot	Agar	Boletal	Sclerode	Scleroderm	Scleroderma	0	0	0	0	1	0	0	0	0	0	0	0	1
Cluster493	Basidiomycot	Agar	Hymen	Hymenc	Fomitiporia	Fomitiporia r	0	0	0	0	0	0	0	0	0	0	1	0	1
Cluster500	Basidiomycot	Agar	Boletal	Boletace	Xerocomell	Xerocomellus	0	0	0	0	0	0	0	0	0	0	1	0	1
Cluster501	Basidiomycot	Agar	Agarica	n	Inocybe	Inocybe aff. a	0	0	0	0	0	0	0	0	0	0	0	1	1
Cluster514	Basidiomycot	Agar	Boletal	Sclerode	Scleroderm	Scleroderma	0	0	0	0	0	0	0	1	0	0	0	0	1
Cluster521	Basidiomycot	Agar	Hymen	n	Resinicium	Resinicium b	0	0	0	0	0	0	0	0	0	0	0	1	1
Cluster528	Basidiomycot	Agar	Russula	Stereace	Amylostere	Amylostereu	0	0	0	0	1	0	0	0	0	0	0	0	1
Cluster540	Basidiomycot	Agar	Boletal	Boletace	Xerocomell	Xerocomellus	0	0	1	0	0	0	0	0	0	0	0	0	1
Cluster542	Basidiomycot	Agar	Agarica	n	Inocybe	Inocybe geop	0	0	0	0	0	0	1	0	0	0	0	0	1
Cluster545	Basidiomycot	Agar	Boletal	Gyropor	Gyroporus	Gyroporus cy	0	0	0	0	0	0	0	1	0	0	0	0	1
Cluster546	Basidiomycot	Trem	Filobas	n	Cryptococc	Cryptococcus	0	0	0	1	0	0	0	0	0	0	0	0	1
Cluster547	Basidiomycot	n	n	n	Marchandic	Marchandior	0	0	1	0	0	0	0	0	0	0	0	0	1
Cluster571	Basidiomycot	Agar	Boletal	Tapinell	Tapinella	Tapinella atrc	0	1	0	0	0	0	0	0	0	0	0	0	1
Cluster572	Basidiomycot	Agar	Russula	Russulac	Russula	Russula exalb	0	0	0	0	0	0	0	0	1	0	0	0	1
Cluster574	Basidiomycot	Agar	Athelia	Atheliac	Athelia	Athelia arach	0	0	0	1	0	0	0	0	0	0	0	0	1
Cluster583	Basidiomycot	Agar	Agarica	Tricholo	Camarophy	Camarophyllu	0	0	0	0	0	0	0	1	0	0	0	0	1
Cluster591	Basidiomycot	Agar	Agarica	Amanita	Amanita	Amanita brui	0	0	0	0	0	0	0	0	0	0	1	0	1
Cluster592	Basidiomycot	Agar	Agarica	n	Inocybe	Inocybe aff. a	0	0	0	0	0	0	0	0	0	0	1	0	1
Cluster636	Basidiomycot	Agar	n	n	Lepidostror	Lepidostroma	0	0	1	0	0	0	0	0	0	0	0	0	1
Cluster644	Basidiomycot	Trem	Tremel	n	Fellomyces	Fellomyces h	0	0	0	0	0	1	0	0	0	0	0	0	1
Cluster652	Basidiomycot	n	n	n	n	basidiomycet	0	0	0	0	0	0	0	1	0	0	0	0	1
Cluster677	Basidiomycot	Agar	Russula	Russulac	Russula	Russula exalb	1	0	0	0	0	0	0	0	0	0	0	0	1
Cluster684	Basidiomycot	Agar	Agarica	Tricholo	Collybia	Collybia tube	0	0	1	0	0	0	0	0	0	0	0	0	1

Cluster693	Basidiomycot	Agar	Boletal	Sclerode	Scleroderm	Scleroderma	0	0	0	0	0	0	0	1	0	0	0	0	1
Cluster695	Basidiomycot	Agar	Polypo	Merulia	Climacodor	Climacodon s	0	0	1	0	0	0	0	0	0	0	0	0	1
Cluster697	Basidiomycot	Agar	Agarica	Tricholo	Camarophy	Camarophyllu	0	1	0	0	0	0	0	0	0	0	0	0	1
Cluster702	Basidiomycot	Agar	Jaapial	Jaapiace	Jaapia	Jaapia argilla	0	0	1	0	0	0	0	0	0	0	0	0	1
Cluster704	Basidiomycot	Agar	Russula	Russulac	Lactarius	Lactarius dec	0	0	0	0	0	1	0	0	0	0	0	0	1
Cluster707	Basidiomycot	Agar	Phallal	Phallace	Phallus	Phallus hadri	0	1	0	0	0	0	0	0	0	0	0	0	1
Cluster708	Basidiomycot	Agar	Athelia	Atheliac	Amphinema	Amphinema	0	0	0	1	0	0	0	0	0	0	0	0	1
Cluster725	Basidiomycot	Agar	Agarica	Amanita	Amanita	Amanita brur	0	0	0	0	0	0	1	0	0	0	0	0	1
Cluster728	Basidiomycot	Agar	Boletal	Tapinella	Tapinella	Tapinella par	0	0	0	0	0	0	0	0	0	1	0	0	1
Cluster734	Basidiomycot	n	Helicob	n	Helicobasid	Helicobasidiu	0	0	1	0	0	0	0	0	0	0	0	0	1
Cluster740	Basidiomycot	Agar	Agarica	n	Inocybe	Inocybe aff. a	0	0	0	0	0	1	0	0	0	0	0	0	1
Cluster743	Basidiomycot	Agar	Russula	Russulac	Lactarius	Lactarius lign	0	0	1	0	0	0	0	0	0	0	0	0	1
Cluster749	Basidiomycot	Agar	Sebacir	Sebacina	Craterocolla	Craterocolla c	0	1	0	0	0	0	0	0	0	0	0	0	1
Cluster760	Basidiomycot	Agar	Polypo	Lentinac	Phyllotopsis	Phyllotopsis r	0	0	0	1	0	0	0	0	0	0	0	0	1
Cluster782	Basidiomycot	Agar	Agarica	Tricholo	Panellus	Panellus styp	0	0	0	0	0	0	1	0	0	0	0	0	1
Cluster785	Basidiomycot	Agar	Gomph	Gompha	Ramaria	Ramaria stric	0	0	1	0	0	0	0	0	0	0	0	0	1
Cluster798	Basidiomycot	Agar	Russula	Russulac	Lactarius	Lactarius lign	0	0	0	0	1	0	0	0	0	0	0	0	1
Cluster815	Basidiomycot	Agar	Polypo	Coriolac	Fomes	Fomes fomer	0	0	0	0	0	0	0	1	0	0	0	0	1
Cluster828	Basidiomycot	Agar	Agarica	n	Inocybe	Inocybe aff. a	0	0	0	0	0	0	0	0	0	0	0	1	1
Cluster830	Basidiomycot	Agar	Agarica	Tricholo	Panellus	Panellus styp	0	0	0	0	0	0	0	1	0	0	0	0	1
Cluster850	Basidiomycot	Agar	Boletal	Boletace	Boletus	Boletus eduli	0	0	0	0	1	0	0	0	0	0	0	0	1
Cluster854	Basidiomycot	Agar	Hymen	Hymenc	Porodaedal	Porodaedalea	0	0	0	0	1	0	0	0	0	0	0	0	1
Cluster859	Basidiomycot	Agar	Agarica	n	Inocybe	Inocybe geop	0	0	0	0	0	1	0	0	0	0	0	0	1
Cluster868	Basidiomycot	Agar	Agarica	n	Inocybe	Inocybe aff. a	0	0	0	0	0	0	0	0	0	0	1	0	1
Cluster884	Basidiomycot	n	n	n	Sporobolon	Sporobolomy	0	0	0	0	0	0	0	0	0	0	0	1	1
Cluster886	Basidiomycot	Agar	Boletal	Boletace	Xerocomell	Xerocomellus	0	1	0	0	0	0	0	0	0	0	0	0	1
Cluster889	Basidiomycot	Agar	Agarica	Tricholo	Pleurocybel	Pleurocybella	1	0	0	0	0	0	0	0	0	0	0	0	1
Cluster911	Basidiomycot	Trem	Tremel	Tremella	Asterotrem	Asterotremel	0	0	1	0	0	0	0	0	0	0	0	0	1
Cluster921	Basidiomycot	Trem	Filobas	n	Cryptococc	Cryptococcus	0	0	1	0	0	0	0	0	0	0	0	0	1

Cluster923	Basidiomycot	n	Leucos	Leucosp	Leucosporid	Leucosporidi	0	0	0	0	1	0	0	0	0	0	0	0	1
Cluster944	Basidiomycot	Agar	Russula	Russulac	Lactarius	Lactarius lign	0	0	1	0	0	0	0	0	0	0	0	0	1
Cluster956	Basidiomycot	n	Helicob	n	Helicobasid	Helicobasidiu	0	0	0	0	0	1	0	0	0	0	0	0	1
Cluster966	Basidiomycot	Agar	Boletal	Sclerode	Scleroderm	Scleroderma	0	0	0	0	0	0	1	0	0	0	0	0	1
Cluster977	Basidiomycot	Agar	Boletal	Boletace	Xerocomell	Xerocomellus	0	0	0	0	0	0	0	0	0	0	1	0	1
Cluster984	Basidiomycot	Agar	Boletal	Boletace	Xerocomell	Xerocomellus	0	0	0	1	0	0	0	0	0	0	0	0	1
Cluster1006	Basidiomycot	Agar	Agarica	Amanita	Amanita	Amanita brui	0	0	0	0	0	0	0	0	0	1	0	0	1
Cluster1018	Basidiomycot	Agar	Thelep	Theleph	Thelephora	Thelephora s	0	0	1	0	0	0	0	0	0	0	0	0	1
Cluster1026	Basidiomycot	Agar	Agarica	Tricholo	Dendrocolly	Dendrocollyb	0	0	0	0	0	0	0	0	1	0	0	0	1
Cluster1028	Basidiomycot	Agar	Agarica	Cortinar	Cortinarius	Cortinarius b	0	0	1	0	0	0	0	0	0	0	0	0	1
Cluster1033	Basidiomycot	Trem	Cystofi	n	Guehomyce	Guehomyces	0	0	0	0	0	0	1	0	0	0	0	0	1
Cluster1039	Basidiomycot	Agar	Russula	Russulac	Russula	Russula exalb	0	0	0	0	1	0	0	0	0	0	0	0	1
Cluster1044	Basidiomycot	Agar	Boletal	Melanog	Melanogast	Melanogaste	0	0	0	0	0	0	0	0	1	0	0	0	1
Cluster1057	Basidiomycot	Agar	Sebacin	Sebacina	Craterocolla	Craterocolla	0	1	0	0	0	0	0	0	0	0	0	0	1
Cluster1067	Basidiomycot	Agar	Agarica	n	Inocybe	Inocybe geop	0	0	0	0	0	0	1	0	0	0	0	0	1
Cluster1069	Basidiomycot	Agar	Boletal	Sclerode	Scleroderm	Scleroderma	0	0	0	0	0	0	0	1	0	0	0	0	1
Cluster1075	Basidiomycot	Trem	Filobas	n	Cryptococci	Cryptococcus	0	0	0	0	0	0	0	0	0	0	1	0	1
Cluster398	Blastocladior	Blast	Blastoc	Catenar	Catenomyc	Catenomyces	0	1	0	0	0	0	0	0	0	0	0	0	1
Cluster1082	Blastocladior	Blast	Blastoc	Catenar	Catenomyc	Catenomyces	0	0	0	1	0	0	0	0	0	0	0	0	1
Cluster118	Chytridiomyc	Chyt	Chytric	Chytridi	Chytriomyc	Chytriomyces	1	0	0	0	0	0	0	0	0	0	0	0	1
Cluster155	Chytridiomyc	Chyt	Chytric	Chytridi	Rhizidium	Rhizidium en	1	0	0	0	0	0	0	0	0	0	0	0	1
Cluster198	Chytridiomyc	Chyt	Spizello	Spizello	Spizellomyc	Spizellomyce	0	0	0	1	0	0	0	0	0	0	0	0	1
Cluster387	Chytridiomyc	Chyt	Chytric	Chytridi	Chytriomyc	Chytriomyces	0	0	0	1	0	0	0	0	0	0	0	0	1
Cluster395	Chytridiomyc	Chyt	Chytric	n	n	Chytridiales s	0	0	0	0	0	0	0	0	0	1	0	0	1
Cluster434	Chytridiomyc	Chyt	Chytric	Chytridi	Chytriomyc	Chytriomyces	0	0	1	0	0	0	0	0	0	0	0	0	1
Cluster448	Chytridiomyc	Chyt	Chytric	n	n	Chytridiales s	0	0	0	0	0	0	0	0	0	1	0	0	1
Cluster481	Chytridiomyc	Chyt	Chytric	Chytridi	Rhizidium	Rhizidium en	0	0	0	0	0	0	0	0	0	1	0	0	1
Cluster518	Chytridiomyc	Chyt	Rhizop	Rhizoph	Rhizophydiu	Rhizophydiur	0	1	0	0	0	0	0	0	0	0	0	0	1
Cluster593	Chytridiomyc	Chyt	Spizello	Spizello	Rhizophlyct	Rhizophlyctis	0	0	0	0	1	0	0	0	0	0	0	0	1

Cluster605	Chytridiomyc	Chyt	Chytrid	Chytridi	Chytriomyc	Chytriomyces	0	0	0	0	0	0	0	0	0	1	0	0	1
Cluster712	Chytridiomyc	Chyt	Chytrid	Chytridi	Chytriomyc	Chytriomyces	0	0	0	0	0	1	0	0	0	0	0	0	1
Cluster773	Chytridiomyc	Chyt	Spizello	n	n	Spizellomyce	0	0	0	0	0	0	0	0	0	0	0	1	1
Cluster853	Chytridiomyc	Chyt	Chytrid	Chytridi	Rhizidium	Rhizidium en	0	0	0	0	1	0	0	0	0	0	0	0	1
Cluster862	Chytridiomyc	Chyt	Rhizop	Rhizoph	Rhizophydiu	Rhizophydiur	0	0	0	0	0	1	0	0	0	0	0	0	1
Cluster917	Chytridiomyc	Chyt	Rhizop	Rhizoph	Rhizophydiu	Rhizophydiur	0	0	0	0	0	1	0	0	0	0	0	0	1
Cluster1009	Chytridiomyc	Chyt	Chytrid	Chytridi	Rhizidium	Rhizidium en	0	0	0	1	0	0	0	0	0	0	0	0	1
Cluster1045	Chytridiomyc	Chyt	Chytrid	Chytridi	Rhizidium	Rhizidium en	0	0	1	0	0	0	0	0	0	0	0	0	1
Cluster441	Cryptomycot	n	n	n	Rozella	Rozella allom	0	0	0	0	0	0	0	1	0	0	0	0	1
Cluster960	Glomeromyc	Glon	Diversi	Acaulos	Acaulospora	Acaulospora	0	0	0	1	0	0	0	0	0	0	0	0	1
Cluster24	n	n	Cercon	Cercom	Paracercom	Paracercomo	1	0	0	0	0	0	0	0	0	0	0	0	1
Cluster169	n	n	Mortie	Mortier	n	Mortierellace	0	0	0	0	0	0	1	0	0	0	0	0	1
Cluster182	n	n	Mortie	Mortier	Dissophora	Dissophora d	0	0	0	0	0	0	0	0	0	1	0	0	1
Cluster200	n	n	Protost	Protoste	Schizoplasn	Schizoplasmo	0	0	0	0	0	0	0	0	0	1	0	0	1
Cluster201	n	n	Mortie	Mortier	Mortierella	Mortierella p	0	0	0	0	1	0	0	0	0	0	0	0	1
Cluster214	n	n	Bicoso	Bicosoe	Bicosoeca	Bicosoeca pe	1	0	0	0	0	0	0	0	0	0	0	0	1
Cluster231	n	n	Entom	Basidiob	Basidiobolu	Basidiobolus	0	0	1	0	0	0	0	0	0	0	0	0	1
Cluster263	n	n	Endogo	Endogo	Endogone	Endogone pis	0	0	0	0	0	0	1	0	0	0	0	0	1
Cluster267	n	n	Mortie	Mortier	Gamsiella	Gamsiella mu	1	0	0	0	0	0	0	0	0	0	0	0	1
Cluster281	n	n	Endogo	Endogo	Endogone	Endogone pis	1	0	0	0	0	0	0	0	0	0	0	0	1
Cluster291	n	n	Mortie	Mortier	Mortierella	Mortierella s	0	0	1	0	0	0	0	0	0	0	0	0	1
Cluster297	n	n	Endogo	Endogo	Endogone	Endogone pis	0	1	0	0	0	0	0	0	0	0	0	0	1
Cluster344	n	n	Cercon	Heteron	n	Rigidomastix	0	0	0	0	0	0	1	0	0	0	0	0	1
Cluster354	n	n	Mortie	Mortier	Mortierella	Mortierella s	1	0	0	0	0	0	0	0	0	0	0	0	1
Cluster377	n	n	Entom	Entomo	Furia	Furia neopyra	0	0	0	0	0	0	0	0	0	1	0	0	1
Cluster391	n	n	Mortie	Mortier	n	Mortierellace	0	0	0	1	0	0	0	0	0	0	0	0	1
Cluster418	n	n	Mucora	n	Umbelopsis	Umbelopsis r	0	0	0	0	0	0	1	0	0	0	0	0	1
Cluster431	n	n	Mortie	Mortier	Mortierella	Mortierella p	0	0	1	0	0	0	0	0	0	0	0	0	1
Cluster464	n	n	Mortie	Mortier	Mortierella	Mortierella n	0	0	0	0	1	0	0	0	0	0	0	0	1

Cluster479	n	n	Mortie	Mortier	Mortierella	Mortierella v	0	0	0	0	0	0	0	0	0	0	0	1	1
Cluster482	n	n	Kickxel	Kickxella	Mycoemilia	Mycoemilia s	0	0	0	0	0	0	1	0	0	0	0	0	1
Cluster487	n	Colp	Bryom	Bryome	Bryometop	Bryometopus	0	1	0	0	0	0	0	0	0	0	0	0	1
Cluster488	n	n	Protost	Cavoste	Ceratiomyx	Ceratiomyxel	0	0	0	0	0	0	1	0	0	0	0	0	1
Cluster504	n	n	n	n	n	eukaryote SC	0	0	0	1	0	0	0	0	0	0	0	0	1
Cluster513	n	n	Mortie	Mortier	Mortierella	Mortierella s	0	0	0	0	0	0	1	0	0	0	0	0	1
Cluster516	n	n	Mortie	Mortier	Mortierella	Mortierella h	0	0	0	0	0	0	1	0	0	0	0	0	1
Cluster529	n	n	n	n	n	Mucoromyco	0	0	0	0	0	1	0	0	0	0	0	0	1
Cluster550	n	n	Mortie	Mortier	Mortierella	Mortierella a	0	0	0	0	1	0	0	0	0	0	0	0	1
Cluster565	n	Dino	Proroc	Proroce	Prorocentru	Prorocentrur	0	0	0	0	0	0	1	0	0	0	0	0	1
Cluster579	n	n	n	Nuclear	Nuclearia	Nuclearia the	0	0	0	0	0	0	0	0	0	1	0	0	1
Cluster582	n	Colp	Bryom	Bryome	Bryometop	Bryometopus	0	0	0	0	0	0	0	0	0	1	0	0	1
Cluster597	n	n	n	Vampyr	Arachnula	Arachnula im	0	0	0	1	0	0	0	0	0	0	0	0	1
Cluster599	n	Spirc	Sporad	Oxytrich	Protogastro	Protogastros	0	0	1	0	0	0	0	0	0	0	0	0	1
Cluster626	n	n	n	n	n	fungal endop	0	0	0	0	0	0	0	0	1	0	0	0	1
Cluster646	n	n	Mortie	Mortier	Mortierella	Mortierella h	0	0	0	0	1	0	0	0	0	0	0	0	1
Cluster657	n	n	Mortie	Mortier	Dissophora	Dissophora d	0	0	0	0	0	0	0	0	0	0	1	0	1
Cluster658	n	n	Euglyp	Euglyph	Assulina	Assulina mus	0	0	0	0	0	0	0	0	1	0	0	0	1
Cluster699	n	n	Endogc	Endogon	Endogone	Endogone pis	0	0	0	1	0	0	0	0	0	0	0	0	1
Cluster709	n	n	Cercon	Heteron	Heteromita	Heteromita g	0	0	0	1	0	0	0	0	0	0	0	0	1
Cluster750	n	n	Endogc	Endogon	Endogone	Endogone pis	0	0	1	0	0	0	0	0	0	0	0	0	1
Cluster752	n	Flori	Corallir	Corallina	Mesophyllu	Mesophyllum	0	0	1	0	0	0	0	0	0	0	0	0	1
Cluster756	n	n	Mortie	Mortier	Mortierella	Mortierella h	0	0	0	0	1	0	0	0	0	0	0	0	1
Cluster769	n	Nass	Nassuli	Furgaso	Furgasonia	Furgasonia b	0	1	0	0	0	0	0	0	0	0	0	0	1
Cluster793	n	n	n	n	n	soil flagellate	0	1	0	0	0	0	0	0	0	0	0	0	1
Cluster802	n	n	n	n	n	Mucoromyco	0	0	1	0	0	0	0	0	0	0	0	0	1
Cluster827	n	n	Mortie	Mortier	Mortierella	Mortierella p	0	0	0	0	0	0	0	0	0	0	0	1	1
Cluster841	n	n	Cercon	n	Spongomor	Spongomona	0	0	0	0	1	0	0	0	0	0	0	0	1
Cluster844	n	n	Mucora	Mucora	Ambomuco	Ambomucor	0	0	0	0	0	0	0	0	0	0	0	1	1

Cluster856	n	Katal	n	n	Roombia	Roombia trui	0	0	0	0	1	0	0	0	0	0	0	0	1
Cluster863	n	n	Choano	Salpingo	Salpingoeca	Salpingoeca t	0	0	0	0	0	0	1	0	0	0	0	0	1
Cluster885	n	n	Mucora	n	Umbelopsis	Umbelopsis r	0	0	0	0	0	0	0	1	0	0	0	0	1
Cluster891	n	Hete	n	n	Stygamoeba	Stygamoeba	0	0	0	0	0	0	0	0	0	0	0	1	1
Cluster903	n	n	Mortie	Mortiere	Mortierella	Mortierella p	0	0	0	1	0	0	0	0	0	0	0	0	1
Cluster905	n	Spiro	Sporad	Oxytrich	Protogastro	Protogastros	0	0	0	1	0	0	0	0	0	0	0	0	1
Cluster914	n	n	Mortie	Mortiere	Mortierella	Mortierella p	0	0	0	0	1	0	0	0	0	0	0	0	1
Cluster922	n	n	Zoopag	Cochlon	Cochlonem	Cochlonema	0	0	1	0	0	0	0	0	0	0	0	0	1
Cluster932	n	n	Choano	Codono	Monosiga	Monosiga ov	0	0	0	0	0	0	0	0	0	0	1	0	1
Cluster936	n	Dino	Blastoc	n	Dissodinium	Dissodinium	0	0	0	0	0	0	0	0	1	0	0	0	1
Cluster939	n	n	Cercom	Cercom	Paracercom	Paracercomo	0	0	0	0	0	0	0	0	0	0	1	0	1
Cluster951	n	Synu	Ochron	Ochrom	Poteriochr	Poteriochro	0	0	0	0	0	0	0	0	0	1	0	0	1
Cluster953	n	n	Zoopag	Piptocep	Piptocepha	Piptocephalis	0	0	0	0	1	0	0	0	0	0	0	0	1
Cluster978	n	n	Choano	Stephan	Acanthocor	Acanthocorb	0	0	0	0	0	0	1	0	0	0	0	0	1
Cluster985	n	n	Mortie	Mortiere	Mortierella	Mortierella p	0	0	0	0	0	1	0	0	0	0	0	0	1
Cluster988	n	n	Mortie	Mortiere	Mortierella	Mortierella p	0	0	1	0	0	0	0	0	0	0	0	0	1
Cluster992	n	n	n	n	n	Mucoromyco	0	0	0	1	0	0	0	0	0	0	0	0	1
Cluster1001	n	Colp	Bryome	Bryome	Bryometop	Bryometopus	0	0	0	0	0	0	0	0	1	0	0	0	1
Cluster1029	n	Nass	Microth	Microth	Leptophary	Leptopharyn	0	0	1	0	0	0	0	0	0	0	0	0	1
Cluster1032	n	Colp	Colpod	Colpodi	Colpoda	Colpoda hen	0	0	0	1	0	0	0	0	0	0	0	0	1
Cluster1034	n	Colp	Grossg	Grossglo	Pseudoplat	Pseudoplatyc	0	0	0	0	0	0	0	0	0	0	1	0	1
Cluster1035	n	Oligo	Philast	Loxocep	Paratetrahy	Paratetrahym	0	0	0	0	0	0	0	0	0	0	1	0	1
Cluster1037	n	n	Entomo	Basidiob	Basidiobolu	Basidiobolus	0	0	0	0	1	0	0	0	0	0	0	0	1
Cluster1043	n	n	Mortie	Mortiere	Mortierella	Mortierella h	0	0	0	0	0	0	0	0	0	1	0	0	1
Cluster1059	n	n	Mortie	Mortiere	Mortierella	Mortierella s	0	1	0	0	0	0	0	0	0	0	0	0	1
Cluster1060	n	n	Mortie	Mortiere	Gamsiella	Gamsiella mu	0	0	0	0	0	0	0	0	1	0	0	0	1
Cluster1065	n	n	Mortie	Mortiere	Mortierella	Mortierella a	0	0	0	0	0	0	0	0	0	0	1	0	1
Cluster1080	n	n	Mortie	Mortiere	Mortierella	Mortierella a	0	0	0	0	0	0	0	0	0	0	1	0	1
Cluster1081	n	n	Endogo	Endogor	Endogone	Endogone lac	0	0	1	0	0	0	0	0	0	0	0	0	1

Cluster187	Platyhelmintl	Turb	Lecitho	Prorhyn	Geocentrop	Geocentroph	0	0	0	0	0	0	0	1	0	0	0	0	1
Cluster949	Streptophyta	Zygn	Desmic	Closteria	Closterium	Closterium m	0	0	0	0	0	0	0	1	0	0	0	0	1

D/ fungal OTUs based on ITS and associated statistics

specie	OTUId	H1R	E3BS	E4R	E4BS	H1BS	H2R	H2BS	H4R	H4BS	E2R	E2BS	E3R	sum of the re
Scleroderma citrinum	Cluster425	526	0	5	4	245	535	187	189	108	0	0	1	1800
Atheliales sp	Cluster1214	0	3	11	11	2	0	2	2	0	256	249	1	537
Gloniaceae sp	Cluster1222	46	9	13	7	37	32	23	36	26	168	85	18	500
uncultured Russula	Cluster386	60	0	2	0	84	64	61	90	112	0	0	1	474
Inocybaceae sp	Cluster839	0	179	12	0	9	0	1	0	1	4	8	237	451
Laccaria laccata	Cluster185	24	0	1	0	55	1	2	150	153	0	2	0	388
Atheliales sp	Cluster828	0	0	189	183	0	0	1	4	0	0	0	0	377
Russulaceae sp	Cluster361	3	0	0	1	18	2	3	148	159	1	0	0	335
Amanita rubescens	Cluster72	56	32	9	13	85	6	39	22	33	2	1	11	309
Scleroderma citrinum	Cluster400	81	2	2	1	37	79	45	40	16	0	2	0	305
Inocybaceae sp	Cluster160	15	0	0	1	33	63	167	4	11	0	3	0	297
Inocybaceae sp	Cluster1007	1	84	6	0	5	0	0	0	0	5	45	124	270
Atheliales sp	Cluster545	0	71	42	58	7	0	5	0	0	10	7	67	267
Russulaceae sp	Cluster431	4	0	1	0	8	23	31	78	71	0	0	1	217
Cryptococcus podzolicus	Cluster1574	3	23	15	15	4	4	6	7	7	42	27	17	170
Inocybaceae sp	Cluster1071	0	12	2	0	0	1	0	0	0	25	78	20	138
uncultured fungus	Cluster874	3	22	6	6	5	8	40	6	12	0	4	18	130
Xerocomus badius	Cluster744	0	6	7	4	0	0	1	5	8	53	33	7	124
uncultured fungus	Cluster1154	8	7	18	16	7	2	15	6	10	10	18	3	120
Inocybe assimilata	Cluster1003	0	14	11	12	1	27	40	0	0	0	0	1	106
Archaeorhizomyces finlayi	Cluster1657	0	1	67	24	2	11	0	1	0	0	0	0	106
Inocybaceae sp	Cluster636	0	45	0	0	3	0	0	0	0	0	13	44	105
uncultured fungus	Cluster369	0	0	35	59	0	0	3	1	1	0	0	0	99
Xerocomus badius	Cluster376	8	3	34	7	10	0	0	1	0	8	6	13	90
Xerocomus badius	Cluster732	10	0	0	0	2	0	0	0	1	35	38	1	87

Amanita sp	Cluster116	0	0	12	13	0	0	0	1	0	34	17	0	77
Amanita rubescens	Cluster127	10	8	4	5	13	3	7	9	9	1	0	4	73
uncultured fungus	Cluster1234	3	3	6	3	30	0	6	0	1	5	13	2	72
Mortierella humilis	Cluster838	2	6	20	7	2	0	12	5	4	4	1	7	70
Atheliales sp	Cluster601	0	29	9	17	2	0	0	0	0	3	1	7	68
Inocybe assimilata	Cluster855	0	0	15	11	0	16	23	0	0	0	0	3	68
Scleroderma citrinum	Cluster716	16	0	0	0	14	9	5	6	5	0	0	0	55
Amanita rubescens	Cluster113	11	3	3	3	14	1	6	5	7	0	0	1	54
Atheliales sp	Cluster416	0	25	9	10	0	0	0	0	0	0	0	10	54
Cryptococcus terricola	Cluster876	1	4	1	9	6	2	4	0	2	12	11	2	54
Thelephoraceae sp	Cluster399	0	0	15	28	0	0	1	0	0	8	0	0	52
Atheliales sp	Cluster1054	0	13	5	14	0	0	0	0	0	3	0	14	49
Mortierellales sp GD9A	Cluster1239	0	7	1	0	6	2	12	3	11	0	2	5	49
Atheliales sp	Cluster367	0	27	1	0	1	0	1	0	0	0	0	18	48
Atheliales sp	Cluster850	0	15	5	15	0	0	0	0	0	2	2	9	48
Gloniaceae sp	Cluster1192	2	1	0	3	7	5	2	4	1	13	9	1	48
uncultured fungus	Cluster546	0	0	18	26	0	0	0	0	0	2	0	0	46
uncultured fungus	Cluster861	3	5	2	2	2	4	15	2	4	0	3	4	46
Inocybaceae sp	Cluster541	0	17	4	0	0	0	0	0	0	0	2	22	45
Inocybe assimilata	Cluster939	1	3	7	8	0	13	10	0	1	0	0	2	45
Atheliales sp	Cluster1081	0	0	2	4	0	0	0	0	0	23	16	0	45
Gloniaceae sp	Cluster1289	6	1	2	0	11	2	2	2	9	5	1	4	45
Amanita sp	Cluster75	2	0	11	7	0	0	0	0	0	11	11	0	42
Laccaria laccata	Cluster79	2	0	0	0	9	0	1	10	20	0	0	0	42
Amanita rubescens	Cluster539	5	4	6	3	14	0	3	2	4	0	0	1	42
Meliniomyces bicolor	Cluster984	0	0	5	1	0	0	0	19	16	0	0	1	42
Gloniaceae sp	Cluster1190	0	0	29	10	0	0	0	1	0	2	0	0	42
Laccaria laccata	Cluster128	1	0	0	1	9	1	0	17	11	0	0	0	40
Inocybe assimilata	Cluster791	0	3	3	9	0	10	14	0	0	0	0	1	40

uncultured fungus	Cluster1567	1	3	3	1	3	1	3	3	1	11	10	0	40
Atheliales sp	Cluster739	0	19	4	6	0	0	0	0	0	0	1	9	39
Hypocrea koningii	Cluster432	5	0	0	0	6	0	2	4	3	16	2	0	38
Sebacinaceae sp	Cluster690	0	6	9	8	0	0	2	0	0	3	5	4	37
Oidiodendron chlamydosporicum	Cluster974	0	10	3	2	6	0	0	1	1	2	10	2	37
uncultured fungus	Cluster1707	0	2	4	0	1	0	2	0	2	11	13	2	37
Laccaria laccata	Cluster134	3	0	0	0	13	0	1	12	6	0	0	0	35
Amanita rubescens	Cluster223	8	1	1	0	7	1	7	4	2	2	0	1	34
uncultured Trechisporales	Cluster337	0	6	0	2	0	0	0	0	0	2	0	24	34
Atheliales sp	Cluster945	0	0	2	3	0	0	0	0	0	17	11	0	33
Amanita rubescens	Cluster46	4	2	1	2	7	3	5	2	4	0	0	1	31
Amanita sp	Cluster138	0	10	0	0	0	0	0	3	9	0	0	8	30
Helotiales sp	Cluster424	0	2	2	6	0	0	0	2	0	3	4	11	30
Scleroderma citrinum	Cluster646	7	0	0	0	5	8	9	0	1	0	0	0	30
Amanita sp	Cluster74	0	0	3	7	0	0	0	0	0	13	6	0	29
Russulaceae sp	Cluster422	0	0	0	1	0	9	13	5	1	0	0	0	29
Inocybaceae sp	Cluster787	0	9	1	0	0	0	0	0	0	2	4	13	29
uncultured Basidiomycota	Cluster669	0	18	4	0	0	0	0	0	0	0	5	1	28
Xerocomus pruinatus	Cluster313	1	4	11	4	0	0	0	1	0	0	0	6	27
Atheliales sp	Cluster1123	0	0	0	5	1	0	0	0	0	8	13	0	27
Amanita rubescens	Cluster230	9	3	0	0	3	0	6	2	1	0	0	2	26
Atheliales sp	Cluster494	0	5	2	9	0	0	0	0	0	0	1	9	26
uncultured fungus	Cluster685	0	8	3	9	0	0	0	0	0	0	2	3	25
Atheliales sp	Cluster1250	0	0	1	1	0	0	0	0	0	11	12	0	25
Cryptococcus podzolicus	Cluster1718	1	10	2	2	1	0	1	1	2	4	1	0	25

:ads
